# Fluxomics reveals cellular and molecular basis of increased renal ammoniagenesis

**DOI:** 10.1038/s41540-022-00257-2

**Published:** 2022-12-20

**Authors:** Liliane Mpabanzi, Jessica Wainwright, Bas Boonen, Hans van Eijk, Dipok Dhar, Esther Karssemeijer, Cees H. C. Dejong, Rajiv Jalan, Jean-Marc Schwartz, Steven W. M. Olde Damink, Zita Soons

**Affiliations:** 1 Department of Surgery, Maastricht University Medical Centre, and NUTRIM School of Nutrition, Toxicology and Metabolism, Maastricht University, PO Box 616, 6200 MD Maastricht, the Netherlands; 2 Hepato-Pancreato-Biliary and Liver Transplant Surgery, Royal Free Hospital, University College London, Pond Street, London, NW3 2QG UK; 3 Liver Failure Group, UCL Hepatology, Royal Free Hospital, University College London, Pond Street, London, NW3 2QG UK; 4 School of Biological Sciences, Faculty of Biology, Medicine and Health, University of Manchester, Manchester, M13 9PT UK; 5 Department of General, Visceral and Transplantation Surgery, University Hospital RWTH Aachen, Aachen, Germany; 6 Research Center for Computational Biomedicine, University Hospital RWTH Aachen, Aachen, Germany

**Keywords:** Pathogenesis, Nephrology

## Abstract

The kidney plays a critical role in excreting ammonia during metabolic acidosis and liver failure. The mechanisms behind this process have been poorly explored. The present study combines results of in vivo experiments of increased total ammoniagenesis with systems biology modeling, in which eight rats were fed an amino acid-rich diet (HD group) and eight a normal chow diet (AL group). We developed a method based on elementary mode analysis to study changes in amino acid flux occurring across the kidney in increased ammoniagenesis. Elementary modes represent minimal feasible metabolic paths in steady state. The model was used to predict amino acid fluxes in healthy and pre-hyperammonemic conditions, which were compared to experimental fluxes in rats. First, we found that total renal ammoniagenesis increased from 264 ± 68 to 612 ± 87 nmol (100 g body weight)^−1^ min^−1^ in the HD group (*P* = 0.021) and a concomitated upregulation of NKCC2 ammonia and other transporters in the kidney. In the kidney metabolic model, the best predictions were obtained with ammonia transport as an objective. Other objectives resulting in a fair correlation with the measured fluxes (correlation coefficient >0.5) were growth, protein uptake, urea excretion, and lysine and phenylalanine transport. These predictions were improved when specific gene expression data were considered in HD conditions, suggesting a role for the mitochondrial glycine pathway. Further studies are needed to determine if regulation through the mitochondrial glycine pathway and ammonia transporters can be modulated and how to use the kidney as a therapeutic target in hyperammonemia.

## Introduction

Hyperammonemia remains the major cause of hepatic encephalopathy in cirrhotic patients^1–3^. The kidney has a pivotal role in systemic hyperammonemia and has been shown able to switch from an organ of net ammonia production into an organ of net ammonia removal in an experimental model of liver failure^4^. Renal ammoniagenesis and urinary ammonia excretion are highly regulated processes as they are central in the regulation of the acid-base balance^5^. In the kidney, ammonia is produced by proximal tubular cells mainly by degradation of glutamine^6,7^. After production, ammonia is released in the tubule through passive diffusion and through the NHE-3 ammonia transporter^8^. About 40–80% of ammonia is reabsorbed in the thick ascending limb (TAL) of Henle’s loop^9^ predominantly via the co-transporter Na-K-2Cl (NKCC2)^10,11^. Ammonia is transported from the cytoplasma into the interstitium by the NHE4 exchanger located on the basolateral side of TAL^12^ cells. After accumulation in the TAL, ammonia is either passively diffused by transepithelial gradient or actively transported by the Rhesus transporters into the collecting tubules to be excreted in the urine^13^. Though transport of ammonia and its substrates has been extensively studied various conditions such as metabolic acidosis and altered potassium balance^14^, it has yet to be understood in hyperammonemic situations.

This study was designed to enlighten renal ammonia handling related to metabolic pathways and transporters in an experimental rodent model of increased renal ammoniagenesis. The diet was aimed to induce increased renal ammoniagenesis in animals with normal liver function avoiding harmful effects associated with hyperammonemia, such as systemic alterations of pH.

Systems biology has become a promising tool to understand metabolic diseases. Several methods exist to predict metabolic fluxes but they are difficult to calibrate and validate against in vivo data. Constraint-based methods such as Flux Balance Analysis have been widely applied and rely on a stoichiometric model of the metabolic network, a set of constraints on exchange fluxes, and the definition of a metabolic objective. The application of Flux Balance Analysis to animal/human cells and to whole organs is challenging due to the difficulty to precisely characterize exchange fluxes between cells and their environment, which is required a priori knowledge for accurate predictions. The lack of tissue-specific metabolite uptake, secretion, and growth rates urges the development of techniques to understand substrate usage of human cells and organs. Elementary Mode Analysis identifies all feasible paths inherent to a metabolic network. It has the advantage that it does not require parameters beyond stoichiometry, such as constraints on substrate uptake rates and that it also computes non-optimal paths. Alternative methods based on elementary modes (EMs) are based on a more precise computation of the efficiency of individual metabolic routes, such as control-effective fluxes^15^ and structural flux analysis^16,17^. Here, we developed a method based on structural flux analysis and gene expression measurements to study changes in amino acid flux distributions in the kidney in an experimental model of increased renal ammoniagenesis. Like flux balance analysis, structural fluxes rely on the assumption that cells optimize certain objectives; living organisms do not behave randomly but are expected to have evolved to maximize the production of certain compounds that are essential for their survival and growth or for their function. In some cases, an objective other than growth is more relevant, e.g., ATP production to optimize energetic availability. In this paper we investigate the suitability of different objectives to model kidney metabolism and for the first time we compare these predictions with in vivo data.

## Results

The experimental model characterizes the changes in fluxes across the kidney and gene expression associated with increased total renal ammoniagenesis.

The reconstructed kidney metabolic model contains 106 reactions of central metabolism representing glycolysis, TCA cycle, pentose phosphate pathway, urea cycle, amino acid metabolism, growth, and protein breakdown; it contains 85 internal metabolites and 23 external metabolites. 6,625,064 elementary modes were obtained in the kidney metabolic model. In the following, we first present the results related to the healthy kidney, followed by the changes in rats fed with a pre-hyperammonemic diet.

### Ammonia transport is the best objective in a healthy kidney

**Fig. 1:**
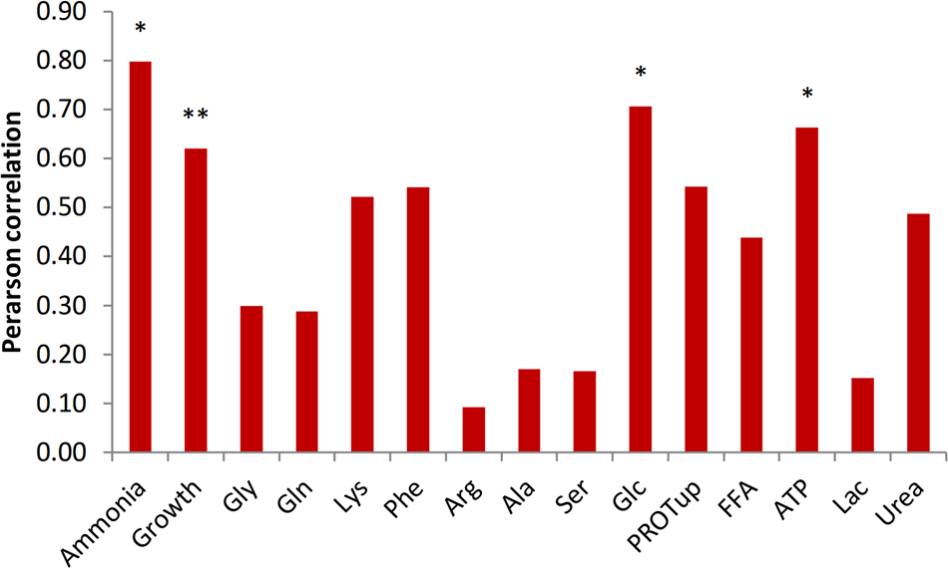
Ammonia transport is the best objective in metabolic modeling of a healthy kidney. Correlation coefficients between measured fluxes in the AL group and predicted values using structural fluxes. The asterisk * indicates a significant correlation between predictions and measurements (*P* < 0.05), **0.05 < *P* < 0.1.

Of the 15 tested objectives, three of the predicted flux distributions gave significant correlations with the measured fluxes across the kidney: ammonia excretion, glucose production, and energy production (ATP maintenance reaction). These objectives reflect relevant biological renal processes. The best predictions in terms of Pearson correlation coefficient were obtained when ammonia transport was used as the objective with a correlation coefficient of 0.80 (Fig. 1 and Supplementary Fig. 2). Other objectives that resulted in a fair correlation with the measured fluxes (correlation coefficient >0.5) were growth, protein uptake, urea excretion, and lysine and phenylalanine transport. These are related to metabolism in the postabsorptive state and with the kidney’s function to redistribute (essential) amino acids into the circulation.

### The pre-hyperammonemic diet did not affect weight growth or arterial pH

Weight gain in HD animals compared with AL animals after 7 days of diet was not statistically different [AL: 66 (16–121) vs HD 45 (30–72) grams, *P* = 0.08]. Changes in ammonia transporters were not due to altered systemic pH, as pH was normal and not significantly different between both groups [AL: 7.29 (7.02–7.47) vs HD 7.37 (7.31–7.44), *P* = 0.39]. Renal flow [AL: 3.0 (2.5–3.8) vs. HD 3.1 (2.1–3.6) ml min^−1^, *P* = 0.35] and urinary flow were not significantly altered [AL: 7.1 (5.0–10.0) vs. HD 9.0 (4.3–14.3, *P* = 0.45].

### The pre-hyperammonemic diet led to increased renal ammoniagenesis

**Fig. 2:**
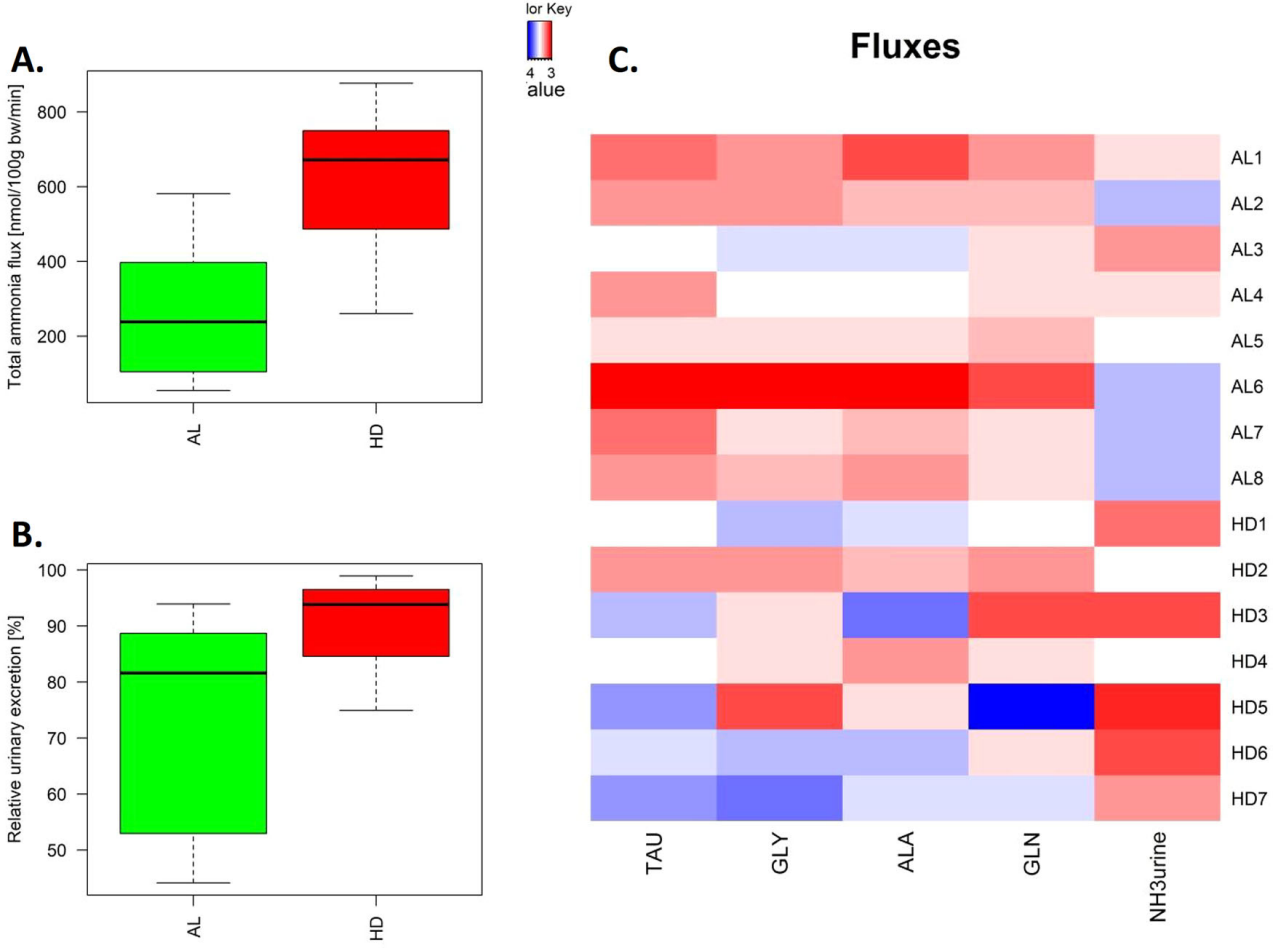
Pre-hyperammonemic diet increased total renal ammoniagenesis. **A** Total renal ammoniagenesis. **B** Percentage urinary excretion relative to total renal ammoniagenesis. **C** Scaled fluxes (*P* < 0.2), red indicates higher than the mean value, blue lower. This corresponds to clearance from the systemic circulation in kidney (blue) and addition to the systemic circulation (red) for the fluxes in the veins. In the boxplots, the center lines represent the median; box limits represent upper and lower quartiles; whiskers represent 1.5× interquartile range.

Total renal ammoniagenesis in the HD group more than doubled compared to the AL controls (Fig. 2A, 612 ± 87 vs 264 ± 68 nmol (100 g body weight bw)^−1^ min^−1^, *P* = 0.021). The HD diet resulted in a 2.6-fold increase in ammonia efflux to urine (201 ± 28 vs. 80.2 ± 19.6 nmol (100 g bw)^−1^ min^−1^) (*P* = 0.029) and no significant increase in ammonia efflux into the systemic circulation (49.2 ± 17.6 in HD vs. 44.8 ± 7.21 nmol (100 g bw)^−1^ min^−1^ in AL). Relatively, urinary ammonia excretion increased from 73% of total renal ammoniagenesis in the AL group to 90% in the HD group (*P* = 0.028) (Fig. 2B).

### The pre-hyperammonemic diet led to elevated amino acid clearance

Four main amino acids were predominantly taken up from the systemic circulation in the pre-hyperammonemic diet (glutamine, alanine, glycine, and taurine) (blue), whereas the kidney released amino acids into the systemic circulation in a normal diet (red) (each with a *P* < 0.2 and a combined *P* of 0.0025) (Fig. 2C). Note that the clearance of amino acids is not necessarily reflected in blood concentrations (Supplementary Table 1).

### The pre-hyperammonemic diet led to increased transcription for transport and metabolic reactions

**Fig. 3:**
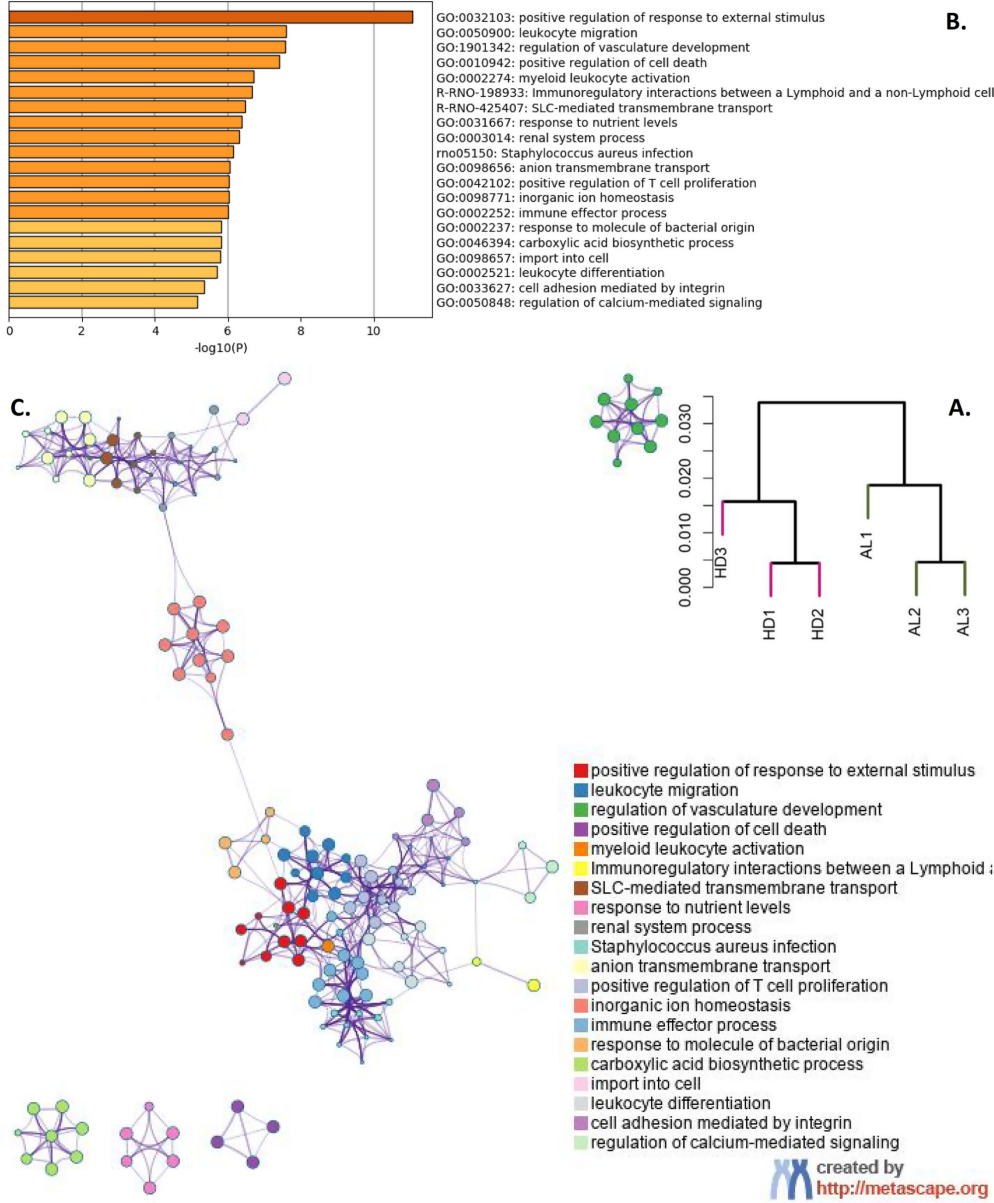
Gene expression analysis. **A** Dendrogram of expressed genes. **B** Functional enrichment of the differentially expressed genes. The *x* axis represents −log10 of the *P* value. **C** Each node represents a functionally enriched term, where the size is proportional to the number of input genes that fall into that term, and the color represents cluster identity. Terms with a membership similarity >0.3 are linked by an edge.

Gene expression profiles were analyzed for three animals in each group. RNA of 17,051 genes was measured, of which 11,512 were expressed in at least one sample (read count of at least 30). Hierarchical clustering of these expression levels revealed distinct clusters of the AL and HD groups (Fig. 3A). In total, 449 genes were differentially expressed between these groups. These genes were enriched for processes related to inflammatory response and metabolic reactions (Fig. 3B, C). Genes involved in inflammatory response were highly over represented (fold enrichment of up to 35). Interestingly in this study, many genes involved in transport of ions were upregulated in the HD group, amongst others ammonia and glutamate transporters (Supplementary Fig. 1). The two most significantly upregulated genes were podocin (Nphs2) and nephrin (Nphs1), which play a role in the regulation of glomerular permeability (Supplementary File Section 4). In addition, four genes in the serine–glycine pathway showed elevated and high expression levels in the HD group (mean log2 expression level of 12.6, compared with a mean level of all expressed genes of 8.49). None of the genes from the urea cycle, which may also be involved in ammonia detoxification, was differentially expressed (adjusted *P* value >0.4).

### Integrated modeling of fluxes and gene expression points to altered pathway activities

**Fig. 4:**
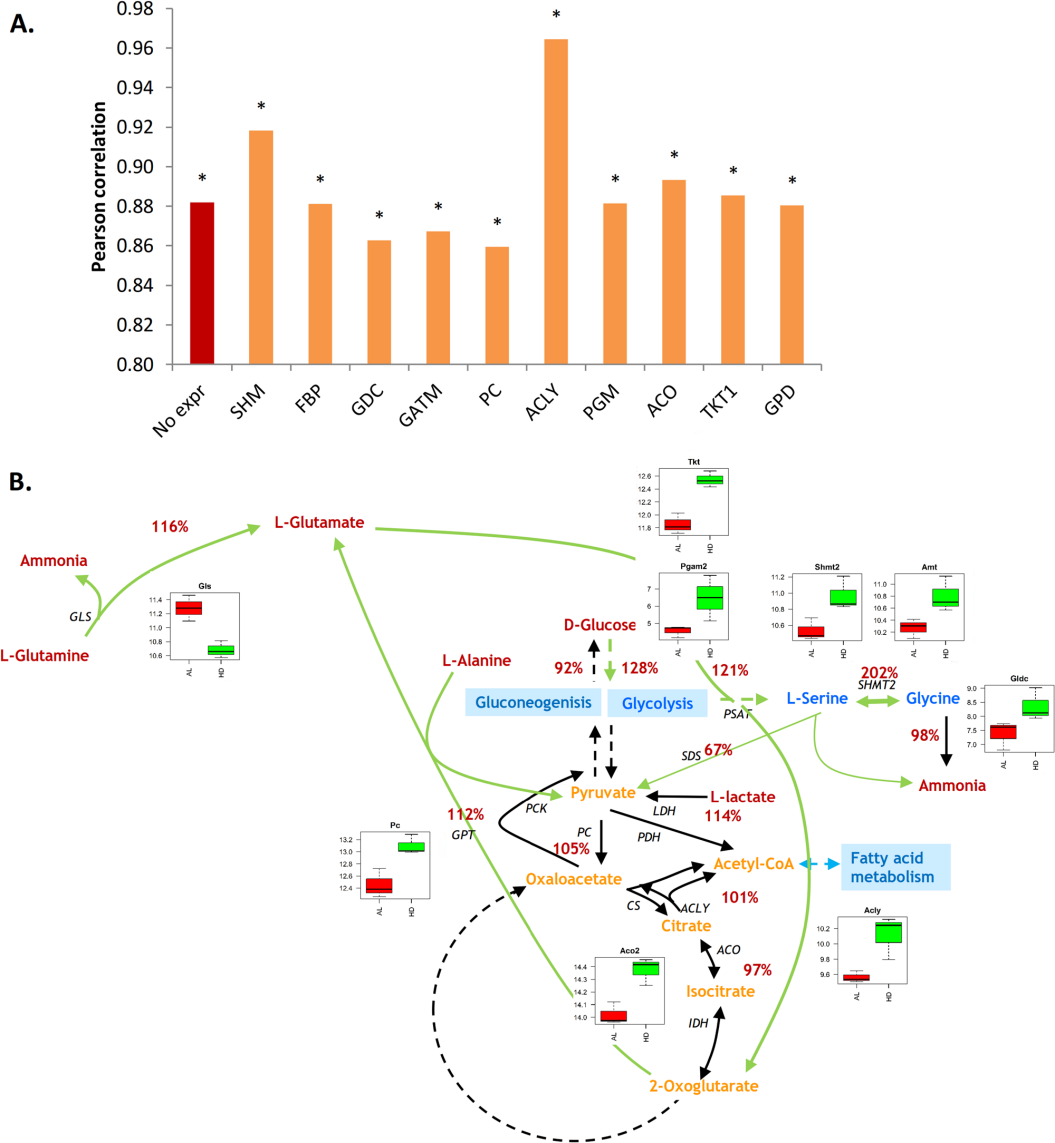
Effect of gene expression alterations on flux predictions in HD. **A** Pearson correlation coefficients between measured and predicted exchange fluxes. The objective in all simulations in this figure is ammonia. The reactions on the *x* axis—SHM, FBP, etc.—represents the effect of overexpression of the respective genes on the predicted fluxes and the resulting Pearson correlation with the measured exchange fluxes. The asterisk * indicates significance. **B** Relative changes in predicted fluxes (%) for activity of SHM and AMT genes in all pathways. Boxplots show the gene expression levels of significantly altered genes in the rat.

Flux predictions with ammonia transport as the biological objective best represented the measured amino acid fluxes over the kidney in the AL group (Fig. 1). Hence, the predicted fluxes with ammonia as the objective were used as a starting point for integration with gene expression. The correlation coefficient of predicted fluxes with ammonia as the objective improved from 0.80 in the AL conditions to 0.88 in HD conditions. All correlations of predicted structural fluxes for upregulated genes with measured fluxes were significant (Fig. 4A and Supplementary Fig. 3). The modeling predictions improved most when the genes serine hydroxymethyltransferase 2 (Shmt2) (or other upregulated genes in the same pathway) (*r* = 0.92) or ATP citrate lyase (Acly) (*r* = 0.96) were considered for HD conditions. The highest correlation between measured and predicted fluxes is through increased activity of Acly and increased cellular turnover, and thus higher anabolic demand. Although this hypothesis is supported by the increased activity of many genes in fatty acid metabolism, it would need follow-up experiments for validation, in which also cellular turnover and fatty acid fluxes over the kidney are measured.

In this work, measurements were focused on amino acid metabolism and we studied Shmt2 in more detail. Figure 4B shows relative changes in flux predictions in HD compared with AL condition when only the pathways containing Shmt2 are active. The structural fluxes predicted an increase of flux through the mitochondrial glycine synthesis pathway. It involves an increased flux from glutamine to glutamate, transamination of glutamate and glycolysis intermediate to oxoglutarate and serine, and glycine cleavage. It is supported by upregulated genes in glycolysis phosphoglycerate mutase 2 (Pgam2), the glycine synthesis pathway (Shmt2, aminomethyltransferase Amt), and glycine cleavage system (glycine decarboxylase Gldc, Amt), and contradicted by downregulation of glutaminase (Gls). The elevated activity of the serine–glycine pathway is in accordance with the measured elevated glycine and glutamine clearance by the kidney (Fig. 2B).

### The pre-hyperammonemic diet led to increased expression of ammonia transporters

**Fig. 5:**
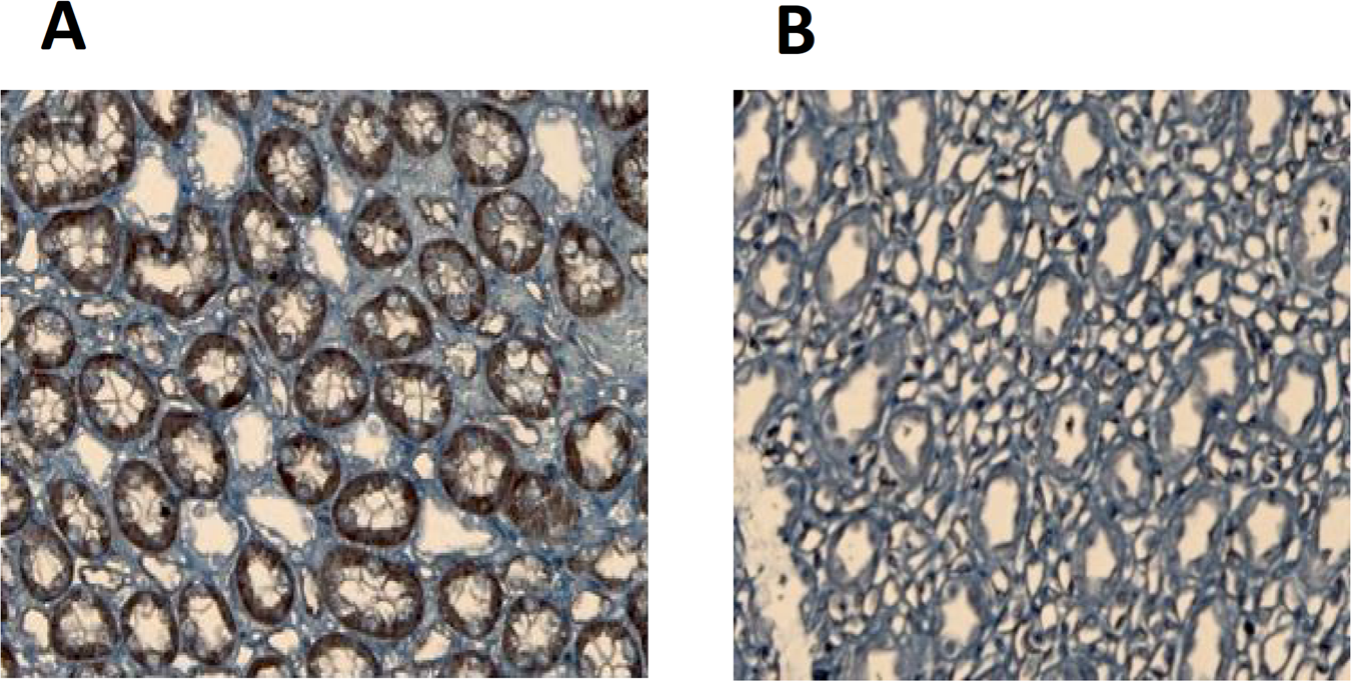
The pre-hyperammonemic diet affected the expression of transporters. Immunohistochemistry showing overexpression of NKCC2 in the HD (**A**) vs AL (**B**) group.

Although the expression levels of most genes support the predicted and measured flux changes, the downregulation of Gls is apparently contradictory. However, regulation of metabolic activity may take place at the post-transcriptional level. In fact, Gls is known to be regulated by ADP. NKCC2 (Slc12a1 in Supplementary Fig. 1) was also downregulated on a gene expression level, whereas immunohistochemistry showed overexpression of NKCC2 in the HD group (Fig. 5). The pre-hyperammonemic diet had no effect on the gene expression (qPCR) of any of the three isoforms of the NKCC2 transporter (0.37 < *P* < 0.96). The pre-hyperammonemic diet did not increase the expression of Rhesus transporters in the collecting ducts (*P* = 0.61).

## Discussion

This paper had two main goals: first, to characterize experimentally the changes in metabolic processes and gene expression associated with increased total renal ammoniagenesis without liver failure, in order to better understand the mechanisms behind this pathology; second, to construct a predictive kidney metabolic model of the pathways involved in renal ammoniagenesis in order to investigate how the development of hyperammonemia can be explained by fundamental shifts in cellular metabolic objectives and gene expression.

This study showed that increased total renal ammoniagenesis, without metabolic acidosis or liver failure, is associated with increased expression of renal ammonia and amino acids transporters. It also showed that increased total renal ammoniagenesis greatly influences renal ammonia handling by altering gene expression in several important ammoniagenic pathways. In particular, the mitochondrial serine–glycine pathway might play a role. Although it was not possible to measure the intracellular fluxes in the kidney (and we are not aware of any publications that has been able to achieve that in tissue), this approach of combining gene expression with the metabolic restrictions imposed by stoichiometry, shed light on the changes that might occur during a pre-hyperammonemic diet.

### Computational model and simulation

In contrast to FBA, EMs analysis has the advantage that it does not require constraints substrate uptake rates, which are often lacking. It has the disadvantage that the enumeration of the complete set of elementary modes for large networks has been infeasible so far, even undesirable due to the hardly manageable number of modes that would be generated. We hence reconstructed a medium-scale model for the kidney using specific information about protein abundances contained from the Human Proteome Atlas and RNA abundances from our own dataset as the main sources of evidence to represent central metabolism of the kidney. We also assumed an artificial biomass reaction based on overall composition. Although we defined the model to our best possible knowledge and data of the kidney, choices on the presence/absence of specific reactions might influence the results of the simulations as in any tissue-specific or reduced model.

### Biological objectives

Although a biological objective function had been suggested in metabolic flux analysis of kidney metabolism by Chang et al.^18^, this study is the first to systematically compare a range of biological objectives with experimental data. It is important to note that our modeling approach considers all possible pathways (elementary modes), whereas for instance in classical Flux Balance Analysis, using ATP production as an objective would lead to a single optimal pathway being highlighted and thus disregard cell renewal and other cellular fluxes. The present approach does predict growth for all objectives, since growth is included in most pathways. Hence, whereas growth must explicitly be included in the objective function for Flux Balance Analysis, a single objective is sufficient for structural fluxes. We found that using ammonia transport as the objective led to the best prediction for measured fluxes.

### Regulation

Though the use of gene expression profiles to construct tissue or cell line-specific metabolic models has been established^19,20^, the validity of genome-wide gene expression profiles for flux prediction has been questioned^21^, since an up- or downregulation of a specific gene might not relate with a (linear) change in the respective flux. It might be regulated at a translational level, by post-translational modifications, or depend on other enzymes in the network. As Machado et al.^21^ concluded in their review, it is known that in particular the central metabolic pathways are more heavily regulated at post-transcriptional levels, hence genome-wide transcript levels are in general not suitable for estimation of fluxes of the central carbon metabolism. Albeit the importance of post-transcriptional regulation, transcriptional regulation of specific genes undoubtedly plays an important role. Hence, rather than using all genome-wide gene expression levels, as for example done by Kurata et al.^22^, we like to argue that using only genes, known to be regulated at a transcriptional level, is more promising in the search for biologically meaningful flux predictions. This, however, is often unknown. Here, we investigated one-by-one which overexpressed gene contributes to enhanced flux predictions and found that in particular fluxes through the Shmt2 pathway and Acly might be activated at a transcriptional level in rat kidneys at hyperammonemic conditions. The activity of the mitochondrial glycine synthesis pathway has also been implicated in cancer cell lines by isotope labeling experiments^23,24^. Shmt2 is also a drug target in other diseases, for which approved drugs are available, such as the antimalarial agent Artenimol^25^.

### Metabolism

Metabolism of ammonia in the kidney is solely limited to the proximal tubules. Glutamine is considered the main substrate of renal ammoniagenesis^26^. However, pathophysiological changes can create a shift in the substrates used by the kidney to generate ammonia^27,28^. Several glutamine transporters were overexpressed at RNA level. Of particular interest are the transporters from the SLC family. The most acknowledged glutamine transporters belong to the SLC1, 6, 7, and 38 families^29^. They have been shown to be of crucial importance in drugs development^30^ and in cancer research^31,32^ and are known to be expressed in the proximal tubule, where glutamine transport occurs. The results of the present study show an upregulation of glutamine transporters at RNA levels.

### Ammonia transport

After production in the proximal tubular cells, ammonia is transported into the lumen through the NHE-3 (Slc9a3) transporter. The apical NHE-3 transporter is thought to be the major transporter of ammonia in the proximal tubule^33^. It has been shown that NHE-3 expression is upregulated in metabolic acidosis^34^. The present study also demonstrated an increase in gene expression pre-hyperammonemic suggesting that the activity of this transporter is closely linked to the amount of ammonia produced in the proximal tubule. Following excretion in the lumen, the accumulation of ammonia in the TAL is mainly regulated by the NKCC2 co-transporter. It has been shown that the expression of this transporter is upregulated during chronic metabolic acidosis^10,11^ mainly through stabilization of mRNA^35^. Our results show that the protein expression of NKCC2 is also upregulated in conditions where ammonia levels are high but the pH is still neutral. Interestingly, it has been shown that the expression of NKCC2 is increased in a rodent model of liver failure known to induce hyperammonemia^36–39^, again suggesting a role for NKCC2 in medullary ammonia accumulation in liver failure. In these studies, the authors did not report on ammonia levels but the used model (bile duct ligation for 28 days in rodents) has been well documented and has repetitively shown its value as a model of hepatic encephalopathy and hyperammonemia^36,40^. These results and ours suggest an increased accumulation of ammonia following the increase in renal ammoniagenesis. NHE4 (Slc9a4), an ammonium transporter in the thick ascending limb has been shown to be critical in the renal handling of ammonia in rodents^41^. After accumulation in the ascending loop, ammonia is either passively diffused into the collecting tubules or actively transported through the Rh glycoproteins transporters family. In the present study their added value could not be shown. It remains unclear whether this was due gene expression below the detection limit or the factual lack of effects of hyperammonemia on the Rhesus transporters.

A limitation of this study is the fact that amino acids in urine were not measured. This study suggests that the kidney can be used as a target therapy organ by altering renal ammonia handling through transporters (NKCC2 and the SLC family) and the mitochondrial glycine pathway (particularly Shmt2).

## Materials and methods

### Animals

Animal experiments were conducted according to Home Office guidelines under the UK Animals in Scientific Procedures Act 1986. Male-specified pathogen-free Sprague–Dawley rats were obtained from Charles Rivers (Margate, UK). All rats were housed in the comparative biological unit at the Royal Free and University College Medical School, University College London at least a week before start of the experiments. They were given free access to standard rodent chow (RM1 Expanded, SDS Diets, Essex, UK) and water ad libitum, with a light/dark cycle of 12 h, at a temperature of 19 °C to 23 °C and a humidity grade of approximately 50%. All procedures and experiments were approved by the Animal Ethical Committee of UCL under license number PPL 70/6615.

### Pre-hyperammonemic diet

Naive male Sprague–Dawley rats (*n* = 8) were fed a high-protein/ammoniagenic diet (mixture of amino acids mimicking the hemoglobin molecule^39^) for 7 consecutive days. The amino acids preparation was mixed with standard rodent chow powder. Similarly to previously conducted studies, animals were fed 3 g kg^−1^ day^−1^ of the amino acids mixture^42^. One rat deceased before the end-point. The control group (*n* = 8) was fed standard laboratory powder chow ad libitum. Rats were randomly assigned to the groups.

### Experimental design

The animals were randomized for pre-hyperammonemic diet (HD group) or ad libitum standard laboratory chow (AL group). After 7 days of diet, surgery was performed. Briefly, following induction anesthesia (1 L min^−1^ oxygen containing 5% isoflurane), anesthesia was maintained with 2% isoflurane in air, delivered at 0.5 L min^−1^ rate through a facemask. Rectal temperature was maintained at 36 ± 1 °C using a heating pad.

### Renal blood flow measurements

Para-amino hippuric acid (PAH) (Sigma-Aldrich, Gillingham, UK) was used to determine renal blood flow. Micro-catheters were inserted in the left jugular vein and left ureter. A primed, constant 1 mM PAH infusion was infused in the jugular vein at a rate of 0.08 ml h^−1^ after a prime infusion of 0.032 ml (100 g)^−1^ of 50 mM PAH to reach a steady state. The final concentrations of PAH have no influence on renal ammonia excretion^43^. An equilibration period of 30 min was observed before blood and urine collection.

### Blood and urine collection

After the equilibration period, urine was collected on ice for 10 (±5) min. Urine quantity was measured using a Hamilton syringe. Immediately after urine collection, renal venous and arterial blood were collected by direct puncture. Blood was immediately centrifuged (1500×*g* for 10 min at 4 °C). Plasma was processed with 5% trichloric acid (for ammonia measurements) or with 5% sulfuric acid (for amino acids measurements) before being stored at −80 °C till further analysis.

### Antibodies and primers

The NKCC2 antibody was kindly provided by the group of Prof Wagner in Zurich^44^ (1/1000).

### Immunohistochemistry

Formalin-fixed tissue sections (5 mm thick) were used for immunohistochemistry. Immunohistochemistry was performed using the streptavidin ABC duet kit (Dako, Cambridgeshire, UK) as described in ref. ^45^ and in Supplementary File Section 3.

### Quantitative polymerase chain reaction (qPCR)

RNA was extracted from half kidney using TRI reagent according to the manufacturer’s protocol and further processed as described in ref. ^46^ and in Supplementary File Section 3. The geometric mean of the expression levels of two reference genes, cyclophilin A (PPIA) and β2-microglobulin (B2M) was calculated and used as a normalization factor.

### Biochemistry

Ammonia, hematocrit, and PAH concentrations were determined using an enzymatic method (CobaS Integra 400, Roche-diagnostics, Burgess Hill, West Sussex, UK). Amino acids were determined using High-performance liquid chromatography^47^.

### RNAseq

Illumina TruSeq RNA Sample Prep Kit v2 (Illumina, Inc.) was used with 100–1000 ng of total RNA for the construction of sequencing libraries. In short, total RNA samples were poly(A)-selected and subsequently reverser transcribed into double-stranded cDNA. The following steps were cDNA fragmentation, end-repair, and polyadenylation before ligation of the TruSeq adapters containing the index for multiplexing. We checked the quality and quantity of the libraries with a Qubit (1.0) fluorometer and the Caliper LabChip® GX (Caliper Life Sciences, Inc.). The product had an average fragment size of ~260 bp. We normalized the libraries to 10 nm in 10 mm Tris-Cl, pH 8.5 with 0.1% Tween 20.

The TruSeq SR Cluster kit v3-cBot-HS (Illumina, Inc.) was used for cluster generation. Sequencing was performed on the Illumina HiSeq 2000 single-end 100 bp using the TruSeq SBS kit v3-HS (Illumina, Inc.). Isoform expression was quantified using RSEM v1.1.15. As a reference, we used the gene definitions from the University of California Santa Cruz for genome build rn5. RSEM was run with default parameters. Raw and processed data are deposited at GEO accession number GSE106682.

### Gene expression analysis

Gene expression was analyzed in the R statistical computing environment (version 3.3.0). The reads were mapped to Entrez gene identifiers and official gene symbols using Biomart^48^. Genes with read counts below 30 in all samples were excluded from further analysis. For all subsequent analyses, we normalized the read counts using variant stabilization by the DESeq2 R-package^49^. We used size factor correction to account for differences in sequencing depth between the samples. DESeq2 was also applied to obtain dispersion estimates and to measure differential gene expression between different conditions using default parameters. Results are reported as log2 fold changes and associated adjusted *P* values (Benjamini Hochberg correction). Functional gene enrichment analysis was performed using METASCAPE^50^ with default settings. Dendrograms were obtained by hierarchical clustering using Ward’s method and 1 − Pearson’s correlation was used as the distance measure.

### Flux calculations

Blood flow rates of the kidneys were calculated using a formula based on the method of indicator dilution and Fick’s principle^51^ as described in detail before^28^ and in Supplementary File Section 3. Positive flux values represent substrate release and negative values reflect substrate uptake^28^. Renal plasma flow and substrate fluxes represent metabolism of two kidneys. Total ammoniagenesis was calculated as renal flux plus urinary ammonia excretion.

### Stoichiometric model

The metabolic model was reconstructed to represent kidney metabolism in the postabsorptive state. As a starting point, we used a model for central carbon metabolism of cancer cells^17^ and manually adapted it for the reactions present or absent in kidney cells using the Human Protein Atlas^52^. All reactions were checked against the human Recon2 model^53^ and RNA abundance data from our own dataset. Since the metabolic model was aimed to simulate renal metabolism in postabsorptive state, important modifications were the addition of breakdown of proteins and free fatty acids, reactions which are typically absent in stoichiometric models of metabolism. Elementary modes were calculated using efmtool^54^. The kidney metabolic model is provided in Supplementary Files Sections 1 and 2.

### Computational fluxes

Whereas the experimental model describes the fluxes across the kidney, the kidney metabolic model predicts also the fluxes in central and ammonia metabolism giving rise to these exchange fluxes by using structural fluxes. In summary, required inputs to the model are: the stoichiometrix matrix, the definition of a biological objective function, and for the HD simulation: gene expression data. Outputs are: all fluxes including exchange and intracellular fluxes. Structural fluxes estimate the efficiency of direct metabolic routes, or elementary modes (EM), toward a cellular objective. The contributions of all EMs are then weighed accordingly to predict the flux distribution, under the assumption that more efficient routes are favored. Structural fluxes are computed according to ref. ^17^ with slight modifications (Eq. (2)) and are summarized below. The efficiency ε_*i*_ of each elementary mode *i* is defined as the ratio of the EM’s objective to the investment required to establish the EM:

1 $$\varepsilon _i = \frac{{e_i^{objective}}}{{\mathop {\sum}\limits_k {\left| {e_i^k} \right|} }}$$

Where *e*_*i*_^*objective*^ is the yield of production of the cellular objective by EM *i* normalized for substrate uptake. Since the system possesses more than one substrate uptake (glucose, lactate, amino acids, choline, ethanolamine, proteins, and free fatty acids), normalization is needed to quantify yields with respect to different substrates. Here, we chose to use the molecular weight of each substrate:

2 $$e_i^k = \frac{{c_i^k}}{{\mathop {\sum}\limits_{s \in \{ substrates\} } {c_{s,i}MW_{s,i}} }}$$

Where *c*_*i*_^*k*^ is the coefficient of reaction *k* in EM *i*, *c*_*s,i*_ is the coefficient of the input reaction of substrate *s* in EM *i* and *MW*_*s,i*_ the molecular weight of each substrate *s* for EM *i*.

The structural flux of reaction *k* is obtained by weighting each EM containing the reaction by its efficiency and summing up all these contributions:

3 $$aSF_k = \frac{{\mathop {\sum}\limits_i {\varepsilon _i \cdot \left| {e_i^k} \right|} }}{{\mathop {\sum}\limits_i {\varepsilon _i} }}$$

*aSF* values are not comparable across networks^16,55^. Appropriate normalization is necessary if one aims to compare structural fluxes across metabolic networks of different sizes, for example, when integrating with gene expression measurements:

4 $$aStrufF_k = \frac{{aSF_k}}{{aSF_{Total\;Sub}}}$$

### Biological objectives

Although the assumption of optimality toward maximum growth of microorganisms is justifiable, the choice of the most relevant biological objective for human renal metabolism has not been widely studied. Chang et al.^18^ compiled an objective function consisting of the metabolic functions of the organ in relation to blood pressure. The objective function consisted of exchange fluxes, which are all equally weighted, such as absorption of glucose and amino acids, and secretion of urea. Note that ammonia removal was not in this list and that the predicted fluxes were not compared with experimental data. Here, we tested 15 objectives to account for a range of biological processes in the kidney, amongst others ammonia excretion, growth, and uptake of amino acids. Maximum growth is frequently used to simulate microorganisms and cancer cells and might be relevant for an organ with high cellular turnover. In addition, we tested maximum ATP production, which is commonly used to simulate a “normal” human cell (as opposed to a cancer cell). Other investigated objectives in the present study are related to the postabsorptive state of the kidney: lactate uptake, free fatty acid uptake, and protein breakdown.

### Integration with gene expression data

In healthy conditions, we assumed that all genes, and consequently reactions, in the kidney metabolic model might be active and contribute to the fluxes according to their efficiency and flexibility in the pathways (Eqs. (1)–(4)). Differential gene expression analysis identified genes that were upregulated in hyperammonemic conditions. In hyperammonemia, we developed a kidney metabolic model in which we assumed that only the pathways containing significantly upregulated gene(s) were active. The underlying idea is that the cells need to perform a specific task to handle the pre-hyperammonemic diet and that only pathways with increased enzymatic activity are relevant. We predicted the structural fluxes for each upregulated gene separately.

### Statistics

Statistical analyses were performed using R (version 3.3.0). Results are expressed as median and range unless otherwise specified. Nonparametric statistics were used. A *P* of 0.05 or less was considered statistically significant. Combined *P* values were computed using Stouffer’s test. The performance of the computational models was evaluated by the Pearson correlation coefficient between predicted and measured fluxes.

## Supplementary information

Supplementary materials

## Data Availability

Gene expression data are deposited in NCBI GEO entry GSE106682. The kidney metabolic model is available in METATOOL format^56^ in supplementary files sections 1 and 2. All data supporting the findings of this study are openly available within the paper and the Supplementary Information deposited at the *npj Systems Biology* *and Applications* website.

## Code availability

The implementation of Structural Fluxes (Eqs. (2)–(4)) and integration with gene expression data were done in Java (Oracle, JDK 8, https://www.oracle.com/index.html) and run in Eclipse (The Eclipse Foundation, neon release 4.6.0, https://eclipse.org). The code is available on request.

## References

[1] Albrecht J, Jones EA (1999). Hepatic encephalopathy: molecular mechanisms underlying the clinical syndrome. J. Neurological Sci..

[2] Butterworth RF (2002). Pathophysiology of hepatic encephalopathy: a new look at ammonia. Metab. Brain Dis..

[3] Weiss N, Dam G, Rose CF (2018). Ammonia: this is not the end but rather the end of the beginning. J. Hepatol..

[4] Dejong CH, Deutz NE, Soeters PB (1993). Renal ammonia and glutamine metabolism during liver insufficiency-induced hyperammonemia in the rat. J. Clin. Investig..

[5] Hamm LL, Simon EE (1987). Roles and meNchanisms of urinary buffer excretion. Am. J. Physiol..

[6] Owen EE, Robinson RR (1963). Amino acid extraction and ammonia metabolism by the human kidney during the prolonged administration of ammonium chloride. J. Clin. Investig..

[7] Darmaun D, Matthews DE, Bier DM (1986). Glutamine and glutamate kinetics in humans. Am. J. Physiol..

[8] Girardi AC, Di Sole F (2012). Deciphering the mechanisms of the Na+/H+ exchanger-3 regulation in organ dysfunction. Am. J. Physiol. Cell Physiol..

[9] Karim Z, Szutkowska M, Vernimmen C, Bichara M (2005). Renal handling of NH3/NH4+: recent concepts. Nephron.

[10] Attmane-Elakeb A, Karim Z, Bichara M (2002). Role of the Na(+)-K+(NH4+)-2Cl cotransporter of the medullary ascending limb in the regulation of renal acid-base equilibrium. Nephrologie.

[11] Attmane-Elakeb A (1998). Stimulation by in vivo and in vitro metabolic acidosis of expression of rBSC-1, the Na+-K+(NH4+)-2Cl- cotransporter of the rat medullary thick ascending limb. J. Biol. Chem..

[12] Blanchard A (1998). NH4+ as a substrate for apical and basolateral Na(+)-H+ exchangers of thick ascending limbs of rat kidney: evidence from isolated membranes. J. Physiol..

[13] Weiner ID, Verlander JW (2011). Role of NH3 and NH4+ transporters in renal acid-base transport. Am. J. Physiol..

[14] Abu Hossain S, Chaudhry FA, Zahedi K, Siddiqui F, Amlal H (2011). Cellular and molecular basis of increased ammoniagenesis in potassium deprivation. Am. J. Physiol..

[15] Stelling J, Klamt S, Bettenbrock K, Schuster S, Gilles ED (2002). Metabolic network structure determines key aspects of functionality and regulation. Nature.

[16] Soons ZI, Ferreira EC, Patil KR, Rocha I (2013). Identification of metabolic engineering targets through analysis of optimal and sub-optimal routes. PLoS ONE.

[17] Schwartz JM, Barber M, Soons Z (2015). Metabolic flux prediction in cancer cells with altered substrate uptake. Biochem Soc. Trans..

[18] Chang RL, Xie L, Xie L, Bourne PE, Palsson BO (2010). Drug off-target effects predicted using structural analysis in the context of a metabolic network model. PLoS Comput Biol..

[19] Agren R (2012). Reconstruction of genome-scale active metabolic networks for 69 human cell types and 16 cancer types using INIT. PLoS Comput. Biol..

[20] Wang Y, Eddy JA, Price ND (2012). Reconstruction of genome-scale metabolic models for 126 human tissues using mCADRE. BMC Syst. Biol..

[21] Machado D, Herrgard M (2014). Systematic evaluation of methods for integration of transcriptomic data into constraint-based models of metabolism. PLoS Comput. Biol..

[22] Kurata H (2007). Integration of enzyme activities into metabolic flux distributions by elementary mode analysis. BMC Syst. Biol..

[23] Jain M (2012). Metabolite profiling identifies a key role for glycine in rapid cancer cell proliferation. Science.

[24] Kim D (2015). SHMT2 drives glioma cell survival in ischaemia but imposes a dependence on glycine clearance. Nature.

[25] Wishart DS (2018). DrugBank 5.0: a major update to the DrugBank database for 2018. Nucleic Acids Res..

[26] Deferrari G (1994). Renal ammoniagenesis and interorgan flow of glutamine in chronic metabolic acidosis. Contributions Nephrol..

[27] Olde Damink SW (2006). Kidney plays a major role in ammonia homeostasis after portasystemic shunting in patients with cirrhosis. Am. J. Physiol. Gastrointest. Liver Physiol..

[28] Olde Damink SW (2003). The kidney plays a major role in the hyperammonemia seen after simulated or actual GI bleeding in patients with cirrhosis. Hepatology.

[29] Pochini L, Scalise M, Galluccio M, Indiveri C (2014). Membrane transporters for the special amino acid glutamine: structure/function relationships and relevance to human health. Front. Chem..

[30] Nigam SK (2015). Handling of drugs, metabolites, and uremic toxins by kidney proximal tubule drug transporters. Clin. J. Am. Soc. Nephrol..

[31] Bhutia YD (2016). SLC transporters as a novel class of tumour suppressors: identity, function and molecular mechanisms. Biochemical J..

[32] Bhutia, Y. D. & Ganapathy, V. Glutamine transporters in mammalian cells and their functions in physiology and cancer. *Biochim. Biophys. Acta*10.1016/j.bbamcr.2015.12.017 (2015). 10.1016/j.bbamcr.2015.12.017 PMC4919214 26724577

[33] Aronson PS, Suhm MA, Nee J (1983). Interaction of external H+ with the Na+-H+ exchanger in renal microvillus membrane vesicles. J. Biol. Chem..

[34] Ambuhl PM (1996). Chronic metabolic acidosis increases NHE3 protein abundance in rat kidney. Am. J. Physiol..

[35] Karim Z, Attmane-Elakeb A, Sibella V, Bichara M (2003). Acid pH increases the stability of BSC1/NKCC2 mRNA in the medullary thick ascending limb. J. Am. Soc. Nephrol..

[36] Wright, G. et al. Endotoxemia produces coma and brain swelling in bile duct ligated rats. *Hepatology* **45**, 1517–1526 (2007). 10.1002/hep.21599 17523148

[37] Jonassen TE (2003). Effects of renal denervation on tubular sodium handling in rats with CBL-induced liver cirrhosis. Am. J. Physiol..

[38] Jonassen TE (1997). Functional and structural changes in the thick ascending limb of Henle’s loop in rats with liver cirrhosis. Am. J. Physiol..

[39] Jonassen TE, Nielsen S, Christensen S, Petersen JS (1998). Decreased vasopressin-mediated renal water reabsorption in rats with compensated liver cirrhosis. Am. J. Physiol..

[40] Butterworth RF (2009). Experimental models of hepatic encephalopathy: ISHEN guidelines. Liver Int..

[41] Bourgeois S (2010). NHE4 is critical for the renal handling of ammonia in rodents. J. Clin. Investig..

[42] Olde Damink SW, Dejong CH, Deutz NE, van Berlo CL, Soeters PB (1999). Upper gastrointestinal bleeding: an ammoniagenic and catabolic event due to the total absence of isoleucine in the haemoglobin molecule. Med. Hypotheses.

[43] Welbourne TC, Dass PD (1981). Role of hippurate in acidosis induced adaptation in renal gamma-glutamyltransferase. Life Sci..

[44] Wagner CA (2008). Mouse model of type II Bartter’s syndrome. II. Altered expression of sodium- and water-transporting proteins. Am. J. Physiol. Ren. Physiol..

[45] Shah N (2013). Increased renal expression and urinary excretion of TLR4 in acute kidney injury associated with cirrhosis. Liver Int..

[46] Mookerjee RP (2015). Hepatic dimethylarginine-dimethylaminohydrolase1 is reduced in cirrhosis and is a target for therapy in portal hypertension. J. Hepatol..

[47] van Eijk HM, Rooyakkers DR, Soeters PB, Deutz NE (1999). Determination of amino acid isotope enrichment using liquid chromatography-mass spectrometry. Anal. Biochem..

[48] Durinck S, Spellman PT, Birney E, Huber W (2009). Mapping identifiers for the integration of genomic datasets with the R/Bioconductor package biomaRt. Nat. Protoc..

[49] Love MI, Huber W, Anders S (2014). Moderated estimation of fold change and dispersion for RNA-seq data with DESeq2. Genome Biol..

[50] Zhou Y (2019). Metascape provides a biologist-oriented resource for the analysis of systems-level datasets. Nat. Commun..

[51] Owen OE (1981). Hepatic, gut, and renal substrate flux rates in patients with hepatic cirrhosis. J. Clin. Investig..

[52] Uhlen M (2015). Proteomics. Tissue-based map of the human proteome. Science.

[53] Thiele I (2013). A community-driven global reconstruction of human metabolism. Nat. Biotechnol..

[54] Terzer M, Stelling J (2008). Large-scale computation of elementary flux modes with bit pattern trees. Bioinformatics.

[55] Çakir, T., Kirdar, B., Onsan, Z. I., Ulgen, K. O. & Nielsen, J. Effect of carbon source perturbations on transcriptional regulation of metabolic fluxes in *Saccharomyces cerevisiae**BMC Syst. Biol*. **1**, 1–10 (2007). 10.1186/1752-0509-1-18 PMC1855933 17408508

[56] von Kamp A, Schuster S (2006). Metatool 5.0: fast and flexible elementary modes analysis. Bioinformatics.

