# Supplementary material for: Fluxomics reveals cellular and molecular basis of increased renal ammoniagenesis
Source: NPJ Syst Biol Appl. 2022 Dec 20;8:49. doi: 10.1038/s41540-022-00257-2 (PMC9768161; doi:10.1038/s41540-022-00257-2)
Supplement: Supplementary file 1 — Supplementary materials [file 41540_2022_257_MOESM1_ESM.pdf]

Supplementary materials

Fluxomics reveals cellular and molecular basis of increased renal ammoniagenesis

Liliane Mpabanzi[1,2,3], Jessica Wainwright[4], Bas Boonen[1], Hans van Eijk[1], Dipok Dhar[2], Esther Karssemeijer[1], Cees HC Dejong [1,5], Rajiv Jalan[3], Jean-Marc Schwartz[4*], Steven WM Olde Damink[1,2,5*], Zita Soons[1,6*]

[1] Department of Surgery, Maastricht University Medical Centre, and NUTRIM School of Nutrition, Toxicology and Metabolism, Maastricht University, PO Box 616, 6200 MD Maastricht, the Netherlands

[2] Hepato-Pancreato-Biliary and Liver Transplant Surgery, Royal Free Hospital, University College London, Pond Street, London NW3 2QG, United Kingdom

[3] Liver Failure Group, UCL Hepatology, Royal Free Hospital, University College London, Pond Street, London NW3 2QG, United Kingdom

[4] School of Biological Sciences, Faculty of Biology, Medicine and Health, University of Manchester, Manchester M13 9PT, UK

[5] Department of General, Visceral and Transplantation Surgery, University Hospital RWTH Aachen, Germany

[6] Research Center for Computational Biomedicine, University Hospital RWTH Aachen, Germany

* These authors contributed equally

Correspondence

Zita Soons, PhD, Research Center for Computational Biomedicine, University Hospital RWTH Aachen, Pauwelsstrasse 19, 52074 Aachen Germany.

T: +31 6 37306199

E-mail: zsoons@ukaachen.de

Section 1. Metabolic model of the kidney in METATOOL format

```
-ENZREV
FBA TPI GAPD PGK PGM ENO CS1 ACO IDH1 SUCLG2 SDH FH MDH1 RPI RPE TKT1 TKT2 TALA GOT1 SHM ACACT
PRPS GPD ADK CO2TR PROt OXCT SDS GLYt GLNup ALAt GPI  LYSup PHEup

-ENZIRREV
G6PC HEX1 PFK FBP PYK PDH PC KGD ZWF SOL GND ME2 GLTt GLNA GLS CAD1 PHGDH PSAT PSPH GDC LYSmetab
OGDH PHHA TYRdeg PROTsyn OTC ASS1 ASL ARG1 IMPsyn ATPRNsyn GTPRNsyn UMPS CAD2 CAD3 UTPsyn RNAsyn
DA DG DC DU DNAsyn PCsyn PEsyn PSS2 SMsyn CHOLsyn MLsyn Growth NADHX FADHX MAINT CHOLup O2_UPT
ARGt EAup PROTup GPT NH3t BIOMr GLUD2a SERt CLNt FFAup FFAmetab PROTmetab LDH PEPCK ACLY GATM
GLCt CREAt

-METINT
ATP ADP AMP NADP NADPH NAD NADH FAD FADH2 G6P F6P FDP GA3P DHAP P13G P3G P2G PEP PYR ACCOA CO2
OAC CIT ICIT AKG SUCCOA SUC MAL GLT ALA GLY G15L P6G RBL5P RB5P X5P S7P E4P PRO ASP NH3 GLN
CarbP ORN SER PHP P3SER AKA LYS AACOA ACAC PHE TYR ARG Protein CLN ARGSUCC FUM PRPP IMP ATPRN
GTPRN NCarbP ORO UTPRN CTPRN RNA dATP dGTP dCTP dTTP DNA GOH3P Choline PC EA PE PS SM Cholesterol
MembraneLipid O2 BIOM FFA GLUC

-METEXT
BIOMext GLNext GLText LYSext PHEext CHOLext CO2EX O2ext ARGext EAext PROext ALAext NH3ext Urea
LAC GLYext SERext CLNext FFAext PROText CREA GLUCext CREAext

-CAT
G6PC : 1 G6P = 1 GLUC .
HEX1 : 1 ATP + 1 GLUC = 1 ADP + 1 G6P .
GPI : 1 G6P = 1 F6P .
PFK : 1 ATP + 1 F6P = 1 ADP + 1 FDP .
FBP : 1 FDP = 1 F6P .
FBA : 1 FDP = 1 GA3P + 1 DHAP .
TPI : 1 DHAP = 1 GA3P .
GAPD : 1 NAD + 1 GA3P = 1 NADH + 1 P13G .
PGK : 1 ADP + 1 P13G = 1 ATP + 1 P3G .
PGM : 1 P3G = 1 P2G .
ENO : 1 P2G = 1 PEP .
PYK : 1 ADP + 1 PEP = 1 ATP + 1 PYR .
PEPCK : 1 ATP + 1 OAC = 1 ADP + 1 PEP + 1 CO2 .

PDH : 1 NAD + 1 PYR = 1 NADH + 1 ACCOA + 1 CO2 .
PC : 1 ATP + 1 PYR + 1 CO2 = 1 ADP + 1 OAC .
CS1 : 1 ACCOA + 1 OAC = 1 CIT .
ACO : 1 CIT = 1 ICIT .
IDH1 : 1 NAD + 1 ICIT = 1 NADH + 1 AKG + 1 CO2 .
KGD : 1 NAD + 1 AKG = 1 NADH + 1 SUCCOA + 1 CO2 .
SUCLG2 : 1 ADP + 1 SUCCOA = 1 ATP + 1 SUC .
SDH : 1 SUC + 1 FAD = 1 FUM + 1 FADH2 .
FH : 1 FUM = 1 MAL .
MDH1 : 1 NAD + 1 MAL = 1 NADH + 1 OAC .

LDH : 1 NAD + 1 LAC = 1 NADH + 1 PYR .
GPT : 1 AKG + 1 ALA = 1 PYR + 1 GLT .

ZWF : 1 NADP + 1 G6P = 1 NADPH + 1 G15L .
SOL : 1 G15L = 1 P6G .
GND : 1 NADP + 1 P6G = 1 NADPH + 1 CO2 + 1 RBL5P .
RPI : 1 RBL5P = 1 RB5P .
RPE : 1 RBL5P = 1 X5P .
TKT1 : 1 X5P + 1 RB5P = 1 GA3P + 1 S7P .
TALA : 1 GA3P + 1 S7P = 1 F6P + 1 E4P .
```

TKT2 : 1 X5P + 1 E4P = 1 GA3P + 1 F6P .

ME2 : 1 NAD + 1 MAL = 1 NADH + 1 PYR + 1 CO2 .

GOT1 : 1 GLT + 1 OAC = 1 AKG + 1 ASP .
GLNA : 1 ATP + 1 NH3 + 1 GLT = 1 ADP + 1 GLN .
GLS : 1 GLN = 1 GLT + 1 NH3 .
CAD1 : 1 GLN + 2 ATP + 1 CO2 = 1 CarbP + 1 GLT + 2 ADP .
GLUD2a : 1 NAD + 1 GLT = 1 NADH + 1 AKG + 1 NH3 .
SHM : 1 SER = 1 GLY .
PHGDH : 1 NAD + 1 P3G = 1 NADH + 1 PHP .
PSAT : 1 GLT + 1 PHP = 1 AKG + 1 P3SER .
PSPH : 1 P3SER = 1 SER .
GDC : 1 GLY + 1 NAD = 1 CO2 + 1 NH3 + 1 NADH .
SDS : 1 SER = 1 PYR + 1 NH3 .
LYSmetab : 1 LYS + 2 AKG + 1 NADP + 2 NAD + 1 FAD = 1 AKA + 2 GLT + 1 NADPH + 2 NADH + 1 FADH2 .
OGDH : 1 AKA + 2 NAD = 1 AACOA + 2 NADH + 2 CO2 .
ACACT : 1 AACOA = 2 ACCOA .
OXCT : 1 ACAC + 1 SUCCOA = 1 AACOA + 1 SUC .
PHHA : 1 PHE + 1 NADH = 1 TYR + 1 NAD .
TYRdeg : 1 TYR + 1 AKG = 1 FUM + 1 ACAC + 1 GLT + 1 CO2 .

PROTsyn : 0.059 LYS + 0.039 PHE + 0.051 ARG + 0.072 GLY + 0.052 PRO + 0.032 TYR + 0.078 ALA + 0.053 ASP + 0.042 GLN + 0.063 GLT + 0.068 SER + 4 ATP = 1 Protein + 1 AMP + 3 ADP .

OTC : 1 CarbP + 1 ORN = 1 CLN .
ASS1 : 1 CLN + 1 ASP + 1 ATP = 1 ARGSUCC + 1 AMP .
ASL : 1 ARGSUCC = 1 ARG + 1 FUM .
ARG1 : 1 ARG = 1 ORN + 1 Urea .

PRPS : 1 RB5P + 1 ATP = 1 PRPP + 1 AMP .
IMPsyn : 4 ATP + 1 CO2 + 1 PRPP + 2 GLN + 1 ASP + 1 GLY = 4 ADP + 1 FUM + 1 IMP + 2 GLT .
ATPRNsyn : 1 IMP + 1 ASP + 3 ATP = 1 ATPRN + 1 FUM + 3 ADP .
GTPRNsyn : 1 IMP + 1 GLN + 3 ATP + 1 NAD = 1 GTPRN + 1 GLT + 2 ADP + 1 AMP + 1 NADH .
CAD2 : 1 CarbP + 1 ASP = 1 NCarbP .
CAD3 : 1 NCarbP + 1 NAD = 1 ORO + 1 NADH .
UMPS : 1 ORO + 1 PRPP + 2 ATP = 1 UTPRN + 1 CO2 + 2 ADP .
UTPsyn : 1 UTPRN + 1 ATP + 1 NH3 = 1 CTPRN + 1 ADP .
RNAsyn : 0.285 ATPRN + 0.285 UTPRN + 0.215 GTPRN + 0.215 CTPRN = 1 RNA .
DA : 1 ATPRN = 1 dATP .
DG : 1 GTPRN = 1 dGTP .
DC : 1 CTPRN = 1 dCTP .
DU : 1 UTPRN = 1 dTTP .
DNAsyn : 0.285 dATP + 0.285 dTTP + 0.215 dGTP + 0.215 dCTP = 1 DNA .

GPD : 1 NADH + 1 DHAP = 1 NAD + 1 GOH3P .
PCsyn : 1 Choline + 18 ACCOA + 1 GOH3P + 23 ATP + 33 NADH = 1 PC + 17 ADP + 6 AMP + 33 NAD .
PEsyn : 1 EA + 18 ACCOA + 1 GOH3P + 23 ATP + 33 NADH = 1 PE + 17 ADP + 6 AMP + 33 NAD .
PSS2 : 1 PE + 1 SER = 1 PS + 1 EA .
SMsyn : 16 ACCOA + 1 SER + 1 Choline + 16 ATP + 29 NADPH = 1 SM + 2 CO2 + 14 ADP + 2 AMP + 29 NADP .
CHOLsyn : 18 ACCOA + 18 ATP + 14 NADPH = 1 Cholesterol + 6 CO2 + 18 ADP + 14 NADP .
MLsyn : 0.5 PC + 0.2 PE + 0.075 PS + 0.075 SM + 0.15 Cholesterol = 1 MembraneLipid .

Growth : 0.9226 Protein + 0.013 RNA + 0.0052 DNA + 0.0297 MembraneLipid = 1 BIOM .
BIOMr : BIOM = BIOMext .

FFAmetab : 1 FFA + 7 NAD + 2 ATP + 7 FAD = 8 ACCOA + 7 NADH + 2 ADP + 7 FADH2 .

```
PROTmetab : 1 Protein + 1 ATP = 0.059 LYS + 0.039 PHE + 0.051 ARG + 0.072 GLY + 0.052 PRO +
0.032 TYR + 0.078 ALA + 0.053 ASP + 0.042 GLN + 0.063 GLT + 0.068 SER + 1 ADP .

NADHX : 1 NADH + 3 ADP + 0.5 O2 = 1 NAD + 3 ATP .
FADHX : 2 ADP + 1 FADH2 + 0.5 O2 = 2 ATP + 1 FAD .
MAINT : 1 ATP = 1 ADP .
ADK : 1 AMP + 1 ATP = 2 ADP .

ACLY : 1 ACCOA + 1 OAC + 1 ADP = 1 CIT + 1 ATP .
GATM : 1 ARG + 1 GLY = 1 ORN + 1 CREA .

FFAup : 1 FFAext = 1 FFA .
PROTup : 1 PROText = 1 Protein .

CREAt : 1 CREA = CREAext .
GLCt : 1 GLUC = GLUCext .
GLNup : 1 GLNext = 1 GLN .
GLTt : 1 GLT = 1 GLText .
GLYt : 1 GLY = 1 GLYext .
SERt : 1 SER = 1 SERext .
LYSup : 1 LYSext = 1 LYS .
PHEup : 1 PHEext = 1 PHE .
CHOLup : 1 CHOLext = 1 Choline .
CO2TR : 1 CO2 = 1 CO2EX .
O2_UPT : 1 O2ext = 1 O2 .
ARGt : 1 ARG = 1 ARGext .
CLNt : 1 CLNext = 1 CLN .
EAup : 1 EAext = 1 EA .
PROt : 1 PRO = 1 PROext .
ALAt : 1 ALA = 1 ALAext .
NH3t : 1 NH3 + 1 ATP = 1 NH3ext + 1 ADP .
```

Section 2. Nomenclature metabolites metabolic kidney model

**Metabolite**	**Name**
AACOA	AcetoacetylC oA
ACAC	Acetoacetate
ACCOA	Acetyl Coenzyme A
ADP	Adenosine diphosphate
AKA	α – ketoadipate
AKG	α − K etoglutarate
ALA	Alanine
AMP	Adenosine monophosphate
ARG	Arginine
ARGSUCC	Argininosuccinate
ASP	Aspartate
ATP	Adenosine triphosphate
ATPRN	Adenosine triphosphate
BIOM	Biomass
CarbP	Carbamoyl phosphate
Cholesterol	Cholesterol
Choline	Choline
CIT	Citrate

CLN	Citruline
CO2	Carbon dioxide
CTPRN	Cytidine triphosphate
dATP	Deoxyadenosine triphosphate
dCTP	Deoxycytidine triphosphate
dGTP	Deoxyguanosine triphosphate
DHAP	glycerone phosphate
dTTP	Deoxythymidine triphosphate
DNA	Deoxyribonucleic acid
E4P	Erythrose 4 – phosphate
EA	Ethanolamine
F6P	Fructose 6 – phosphate
FAD	Flavin adenine dinucleotide
FADH2	Reduced form of flavin adenine dinucleotide
FDP	Fructose-bisphosphate
FFA	Free fatty acid
FUM	Fumarate
G15L	6-phospho-D-glucono-1,5-lactone
G6P	Glucose 6 – phosphate
GA3P	Glyceraldehyde 3 − phosphate
GLN	Glutamine
GLT	Glutamate
GLUC	Glucose
GLY	Glycine
GOH3P	Glycerol 3-phosphate
GTPRN	Guanosine triphosphate
H2O2	Peroxide
ICIT	Isocitrate
IMP	Inosine monophosphate
LYS	Lysine
MAL	Malate
MembraneLipid	MembraneLipid
NADH	Reduced form of nicotinamide adenine dinucleotide
NAD	Nicotinamide adenine dinucleotide
NADPH	Reduced from of mitochondrial Nicotinamide adenine dinucleotide phosphate
NADP	Nicotinamide adenine dinucleotide phosphate
NCarbP	carbamoyl phosphate
NH3	Ammonia
O2	Oxygen
OAC	Oxaloacetate
ORN	Ornitine
ORO	Orotate
P13G	1,3-bisphosphoglycerate
P2G	2 – Phosphoglycerate
P3G	3 – Phosphoglycerate
P3SER	Phosphoserine

P6G	6-phospho-D-gluconate
PC	Phosphatidylcholine
PE	Phosphatidylethanolamine
PEP	Phosphoenolpyruvate
PHE	Phenylalanine
PHP	3-phosphonooxypyruvate
PRO	Proline
Protein	Protein
PRPP	Phosphoribosyl – pyrophosphate
PS	Phosphatidylserine
PYR	Pyruvate
RB5P	ribose 5-phosphate
RBL5P	ribulose 5-phosphate
RNA	Ribonucleic acid
S7P	Sedoheptulose 7-phosphate
SER	Serine
SM	Sphingomyelin
SUC	Succinate
SUCCOA	Succinate C oenzyme A
TYR	T yrosine
UTPRN	Uridinetriphosphate
X5P	Xylulose 5 – phosphate

Section 3: Detailed methods

Immunohistochemistry

Sections were dewaxed in xylene, rehydrated in graded alcohol and rinsed with PBS. Antigen retrieval was done by heating the sections in citrate buffer solution at 958°C for 10 min (Sigma Aldrich, Gillingham, UK). Endogenous peroxidase activity was blocked by 3% hydrogen peroxide for 20 min. The sections were incubated with a rabbit polyclonal antibody. Streptavidin-peroxidase labelled secondary antibody was applied for 30 min at room temperature. Colour was developed with the chromagen 3,30-diaminobenzidine (Dako, Cambridgeshire, UK), and counterstained with Mayer's haematoxylin solution.

Quantitative polymerase chain reaction (qPCR)

RNA samples were treated with DNAse (Promega, Madison, WI, USA) to ensure the removal of contaminating genomic DNA. RNA concentration was determined by Nanodrop (Nanodrop, Wilmington, DE, USA) and 750 ng of RNA was used as the template for reverse transcription in a cDNA synthesis reaction using iScript cDNA synthesis kit (Bio-Rad). qPCR reactions were conducted in a volume of 20 µl containing 10 ng of cDNA, 1× Absolute qPCR SYBR Green Fluorescein Mix (Westburg, Leusden, The Netherlands), and 150 nM of gene-specific forward and reverse primers. The sequences of the primers used were extracted from earlier published material[1].

Flux calculations

The blood flow determined using PAH was converted to plasma flow using the hematocrit levels. Renal urinary ammonia excretion was calculated as the urinary ammonia concentration times the urinary volume. The renal flux was calculated as renal plasma flow times the venous arterial difference divided by bodyweight.

References

1 Hao, S., Zhao, H., Darzynkiewicz, Z., Battula, S. & Ferreri, N. R. Differential regulation of NFAT5 by NKCC2 isoforms in medullary thick ascending limb (mTAL) cells. American journal of physiology 300, F966-975 (2011).

Supplementary Table 1. Renal fluxes and blood concentrations.

Data are means ± standard error. Fluxes are in nmol (100g body weight)^-1 min^-1 per kidney; concentrations in µM. BCCA: sum of the branched chain amino acids, TEAA: sum of the essential amino acids, TNEA: sum of the non-essential amino acids, TAA: sum of all measured amino acids.

AA	**AL flux**	**HD flux**	**p-value**	**AL arterial**	**HD arterial**	**p-value**	**AL venous**	**HD venous**	**p-value**
NH3	44.8±7.2	38.4±21.6	0.46	58.9±10.2	80.4±10.1	0.19	102±12	129±22	0.28
GLN	78.7±37.2	-64.9±114	0.12	790±53	892±98	0.87	875±24	818±53	0.54
ALA	66.0±54.9	-91.9±53.2	0.09	1098±115	1070±102	0.78	1175±89	966±115	0.19
GLY	33.5±32.1	-35.5±44.0	0.19	503±38	506±27	0.87	543±32	465±39	0.34
GLU	5.33±5.6	35.3±46.7	0.46	58.0±8.1	62.4±6.4	0.64	64.6±5.9	1001±47	0.49
SER	94.5±25.8	82.8±25.4	0.87	437±34	453±43	1.00	532±14	531±41	0.87
HIS	18.6±6.0	15.0±9.21	0.87	121±11	127±8	1.00	140±6.5	140±10	0.96
CIT	-22.1±11.9	-47.0±13.3	0.34	127±9	130±10	1.00	109±6.8	78.9±8.4	0.024
TAU	13.4±18.7	-91.2±18.7	0.0012	195±21	308±29	0.0037	211±21	208±47	0.40
ARG	55.2±11.1	36.6±19.0	0.61	170±13	147±14	0.19	225±11	183±13	0.029
TYR	17.4±4.8	13.1±6.9	0.69	75.6±6.6	79.1±9.7	0.86	93.0±6.4	90.4±15.7	0.87
VAL	23.7±12.0	13.8±24.1	0.46	196±13	251±14	0.014	221±10.2	263±31	0.19
MET	10.2±3.2	10.7±4.7	0.96	58.8±4.5	56.1±5.4	0.30	69.0±3.2	66.9±9.1	0.95
ILE	13.4±4.8	16.8±17.7	0.40	113±8	127±13	0.38	126±5	143±23	0.54
PHE	9.44±2.7	15.8±9.4	0.78	60.0±4.5	61.7±5.5	0.45	69.5±1.7	77.6±15.3	1.00
TRP	14.5±3.4	13.0±6.1	0.54	66.6±5.7	70.7±5.4	0.68	80.6±6.2	81.9±9.6	0.78
LEU	14.4±9.7	9.8±29.4	0.34	156±13	214±17	0.0093	171±10	222±36	0.23
ORN	16.8±3.5	34.1±25.8	0.96	73.5±5.7	92.3±11.1	0.25	90.4±4.7	126±35	0.45
LYS	43.2±64.2	84.6±50.0	0.54	288±46	327±42	0.53	306±43	410±74	0.35
THR	27.6±11.3	16.7±86.1	0.46	200±17	378±52	0.0014	228±10	391±66	0.0022
ASN	16.5±4.9	3.10±9.86	0.28	91.0±7.9	93.3±9.5	0.95	108±6	94.4±11.4	0.27
AAB	0.93±0.4	2.09±1.82	0.96	7.63±0.89	10.6±2.2	0.27	8.50±0.65	12.7±1.3	0.029
BCAA	51.5±25.5	40.3±70.1	0.46	465±33	518±83	0.13	518±23	549±110	0.38
TEAA	191±53.2	190±207	0.40	1313±47	1492±227	0.065	1518±65	1652±312	0.23
TNEAA	359±186	-127±167	0.054	3534±266	3281±508	0.80	3927±159	3133±498	0.19
TAA	551±215	73±297	0.28	4848±298	4773±719	0.50	5445±215	4785±794	0.72

Supplementary Figure 1: Boxplots transporters

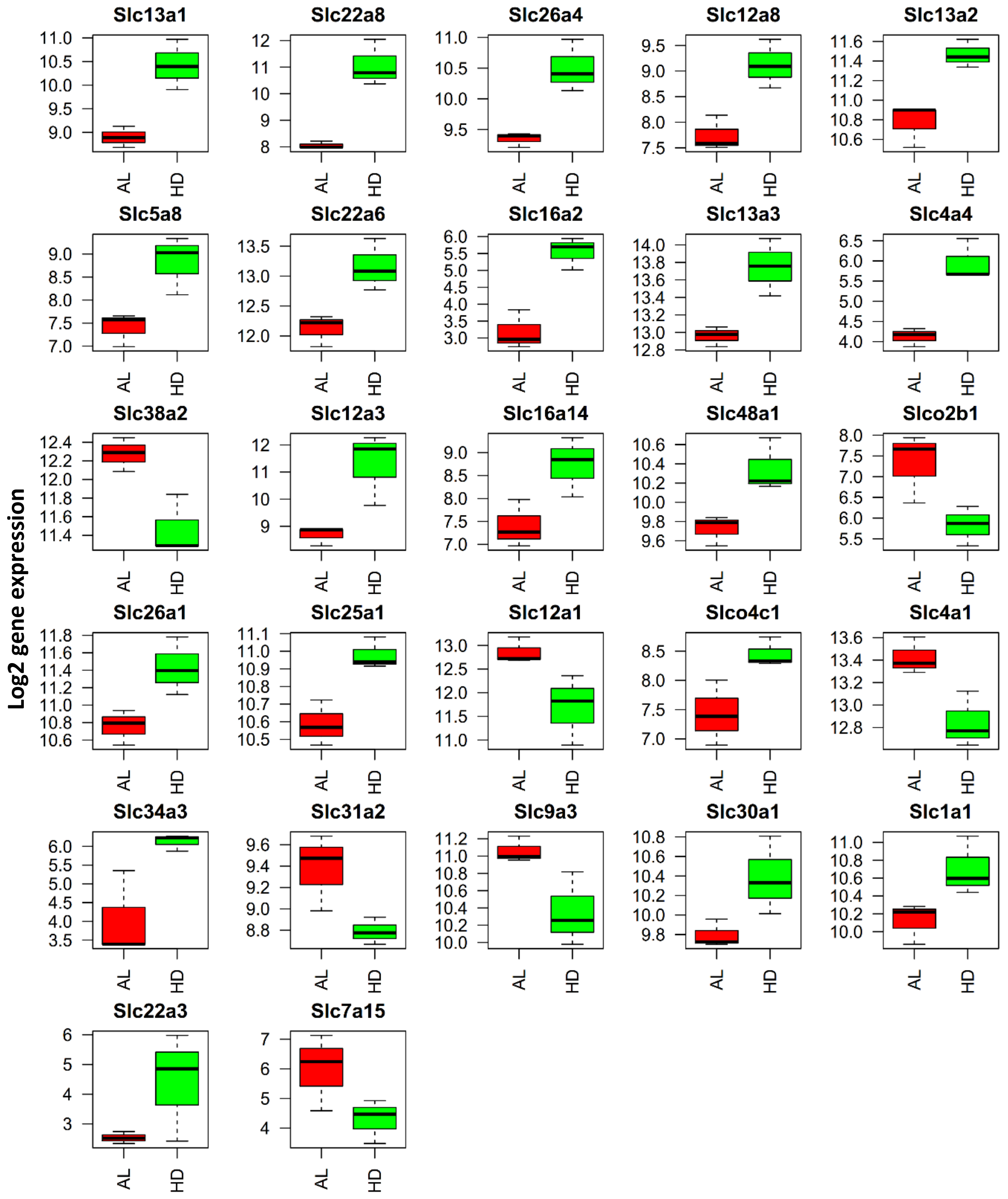

Supplementary Figure 1. Boxplots for gene expression levels of differentially expressed transporters with a gene name containing “slc”. The centre lines represent the median; box limits represent upper and lower quartiles; whiskers represent 1.5x interquartile range.

Supplementary Figure 2. Scatter plots for biological objectives

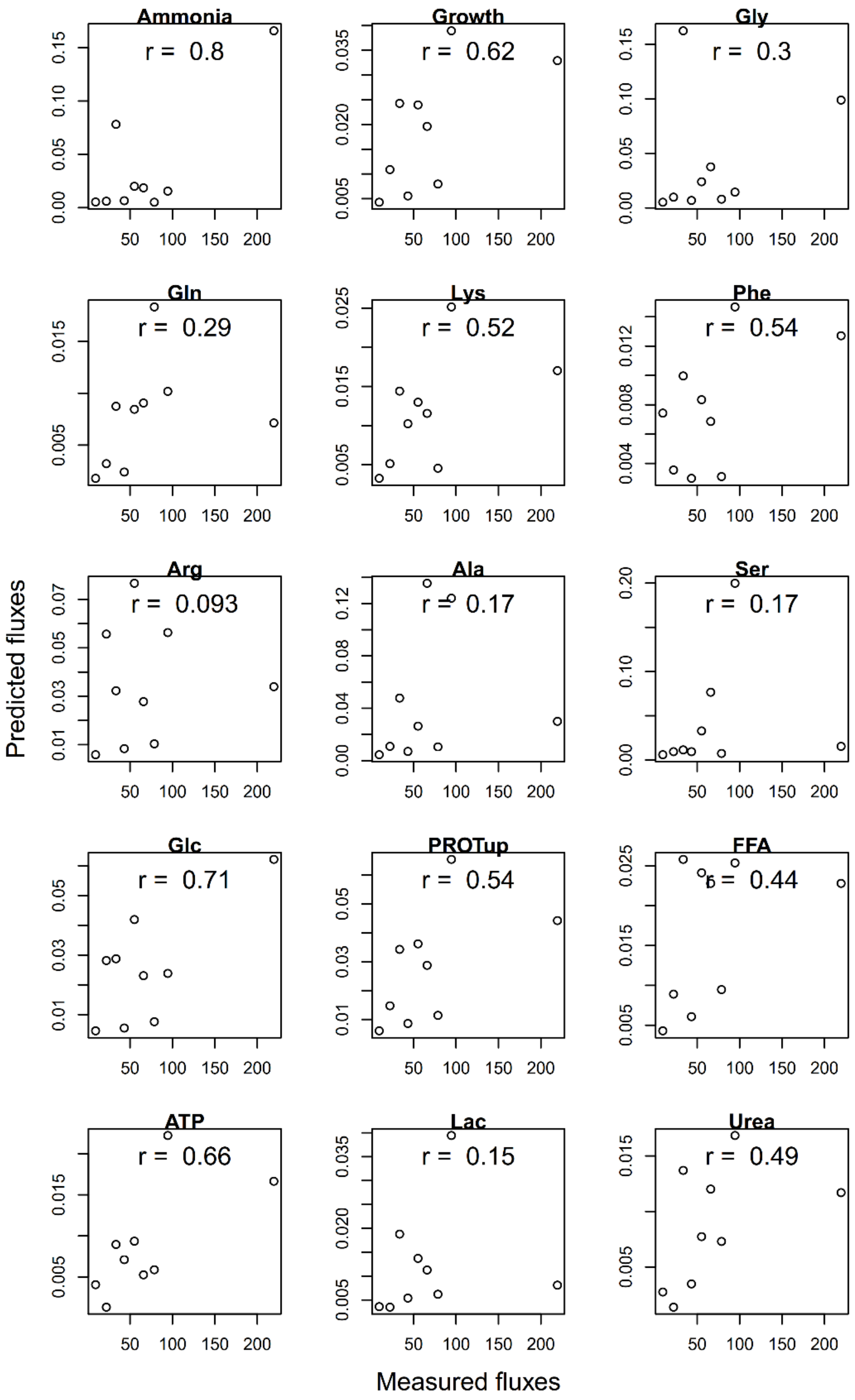

Supplementary Figure 2. Scatter plots for biological objectives in the AL group. The measured flux units are nmol (100g bw)^-1 min^-1. The predicted structural fluxes are relative to the total substrate uptake (Eq. 3).

Supplementary Figure 3: Scatter plots for differentially expressed genes

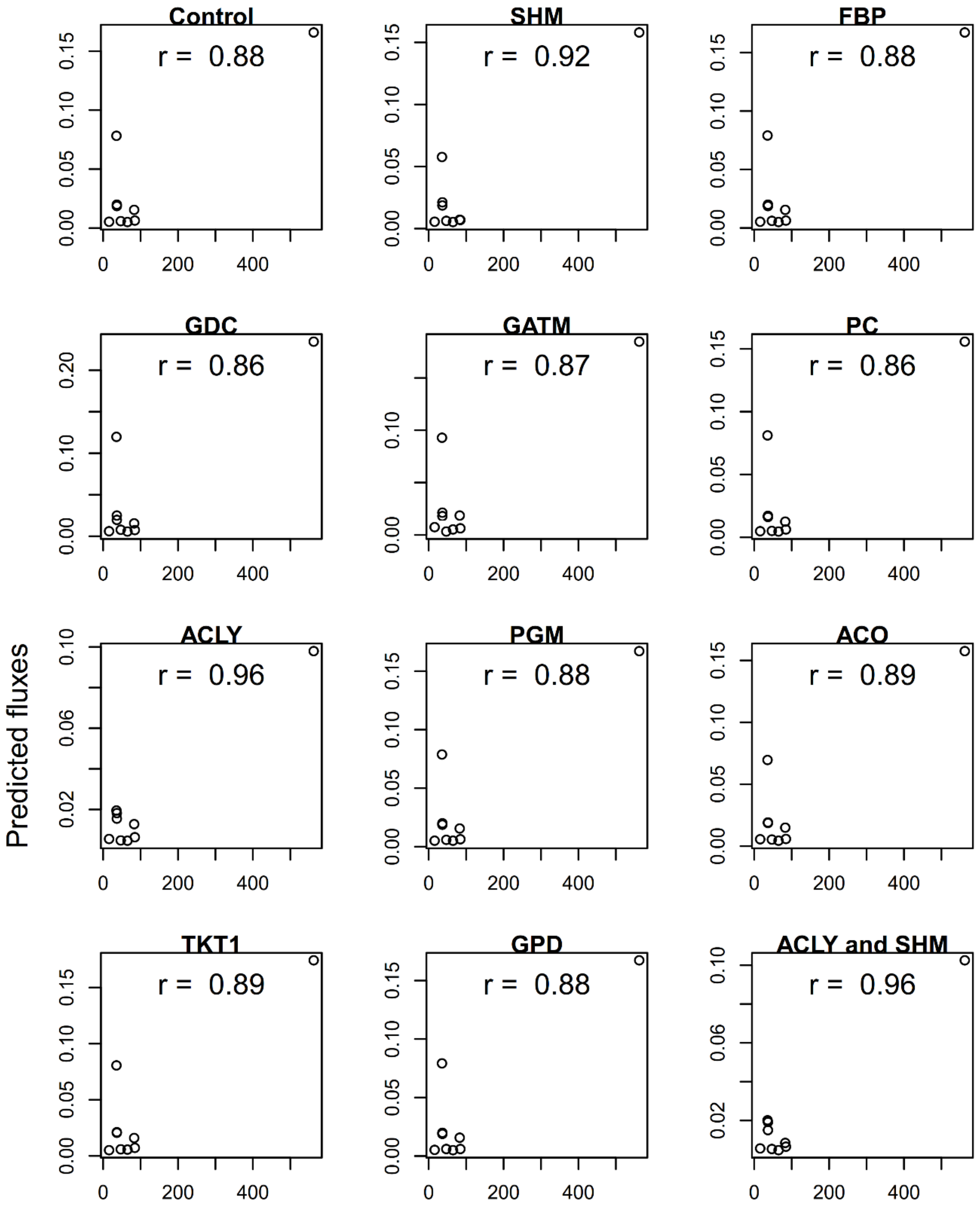

Supplementary Figure 3. Scatter plots for differentially expressed genes in the HD group. The measured flux units are nmol (100g bw)^-1 min^-1. The predicted structural fluxes are relative to the total substrate uptake (Eq. 3).

Section 4. Differential gene expression ranked according to adjusted p-value

	baseMean	log2FoldChange	lfcSE	stat	pvalue	padj
Nphs2	923.264535422646	3.33493842613402	0.273111911081695	12.2108860537263	2.71911672168582e-34	3.0905480658681e-30
Nphs1	597.130065953015	2.34349462136977	0.224920013988321	10.4192356198745	2.02574688000985e-25	1.1512319519096e-21
Tnnc1	179.786451750288	3.41263267063502	0.36041798081904	9.4685416717554	2.83777723587893e-21	1.0751392021e-17
Clic3	175.650655368688	2.73120930003185	0.313609510560943	8.70894921249877	3.06704648835851e-18	8.71501259667071e-15
Sema3g	1080.99892928076	2.24459709081291	0.280019017042831	8.01587375927964	1.09356782801912e-15	2.48589838665307e-12
Ptpro	341.039043436389	2.50715005615662	0.345802413827447	7.25023873722197	4.16037654572488e-13	7.88113996978483e-10
Gdf15	1327.77094545802	1.56689441893863	0.221782485076112	7.06500523880816	1.60609934328434e-12	2.46643205941312e-09
Wt1	83.3394396924292	2.74206409870395	0.388713871951891	7.05419666382093	1.73600708035412e-12	2.46643205941312e-09
Ppm1k	2261.79448713114	0.914677998356528	0.136170088773875	6.71717266686552	1.8528437110761e-11	2.33993573556566e-08
Tdrd5	59.9952422306135	3.09870534371653	0.463478512329487	6.68575837128272	2.29731811255101e-11	2.61113176672548e-08
Mmp9	180.815272509434	3.12098350145517	0.47340597757243	6.59261532239033	4.32145261475085e-11	4.46523912902347e-08
Slc13a1	958.164753729708	1.57523878749591	0.244172546350153	6.45133456255542	1.10869436266786e-10	1.05011834384024e-07
Ddn	167.969428454025	3.18482313688804	0.497777029921021	6.39809180707545	1.57330686758212e-10	1.37555429668757e-07
Sfrp2	143.253412081551	2.75519798858518	0.434366611365999	6.3430243404773	2.25297817767758e-10	1.82909642624881e-07
Fam151a	112.455323239646	2.94582694356244	0.46931458393338	6.27687066289976	3.45455214690819e-10	2.61762931345057e-07
Aqp4	480.512720886447	-1.14234156689737	0.183305306516975	-6.2319066949193	4.60791915752332e-10	3.27335057152563e-07
Slc22a8	1353.03470669187	3.19258371141368	0.514757634525074	6.20211046380929	5.57109501207822e-10	3.72476858278124e-07
RGD1309362	223.919281603027	-1.2795845403331	0.213501953134302	-5.99331538446483	2.05605551823272e-09	1.2982848344574e-06
Slc26a4	1072.62381691724	1.19731215653622	0.202997964129253	5.89814859312515	3.67602861409776e-09	2.19903901199132e-06
LOC257642	12759.2424876757	-1.02230754944753	0.179289662556041	-5.70198825115185	1.18417992018664e-08	6.72969448642067e-06
Ren	223.148184536545	4.02401570097329	0.709803862202405	5.66919386503114	1.43470954689586e-08	7.76519462381824e-06
Igfbp2	324.010743215016	1.27832443726738	0.23507077584229	5.43804066110292	5.38696825005545e-08	2.66209917957088e-05
Sim1	688.656623248672	-0.673886505340284	0.12385665763933	-5.44085815154676	5.30245228093641e-08	2.66209917957088e-05
Aoc3	259.651162026288	1.31190895447692	0.242200347139667	5.41662706090341	6.07338353831061e-08	2.87625322068493e-05
Tkt	4854.93390854253	0.690604772618424	0.127956508449208	5.39718362894029	6.76950956320192e-08	3.07768982781412e-05
Dgkg	155.853120005367	1.77699260536648	0.329904990971156	5.38637684787816	7.18921483376802e-08	3.14279291540797e-05
Areg	155.572814828995	2.27921235665765	0.425389886640697	5.35793733757185	8.41774228787242e-08	3.41700210156993e-05
Sesn2	688.900933087082	0.699933593444978	0.130479456191881	5.36432028361357	8.12546143189577e-08	3.41700210156993e-05
Slc12a8	398.288588836563	1.40968169653017	0.263959053383206	5.34053171680246	9.26743614337143e-08	3.63219583467447e-05
Fetub	27.7197552339064	3.22084189412885	0.606474044581962	5.31076626098476	1.09165266375221e-07	4.13590805873588e-05
Slfn13	261.729636402644	-1.49267070502456	0.285065076842674	-5.23624542703404	1.63876010011772e-07	6.00843461223805e-05
Aldh1l1	3543.07807407326	0.545677959027062	0.104375215014469	5.22804153219148	1.71315032808782e-07	6.08489582157692e-05
Abcc5	605.351552077888	-1.12870026496122	0.2172968490313	-5.19427810386078	2.0551573240798e-07	6.87027004279147e-05
Cpox	1229.79516103864	-0.913825276305794	0.175829541305123	-5.197222659644	2.02288013878302e-07	6.87027004279147e-05
Tmem150c	143.489355610208	1.59692247218073	0.309124905351817	5.16594568905167	2.39226253668922e-07	7.76870171200277e-05
Mal2	2041.6078040452	-0.514557891375371	0.0999345732807539	-5.14894770131037	2.6195190108022e-07	8.27040363243828e-05
Irf4	122.479512354688	1.36972204089453	0.266678053201411	5.13623833851837	2.80292147906558e-07	8.61027176515119e-05
Cercam	172.714949526171	-1.14965248652473	0.224095886716893	-5.1301811174121	2.89463530371029e-07	8.65800654262399e-05
Acox1	3773.97505099962	0.739749152520175	0.144568078623985	5.11696053209801	3.10498561595337e-07	9.04904269510923e-05
Ptprv	68.0247091455739	2.30750027299019	0.452601231251954	5.09830754681631	3.42703710372816e-07	9.73792593024357e-05
Tmem100	80.3235448619504	-2.32168745136151	0.456040307709739	-5.09096983777851	3.56236697027117e-07	9.8755763375859e-05
Ndrg4	115.696677668397	-1.71603890590911	0.341916773635165	-5.01887897357201	5.19738790297399e-07	0.000140651216440958
Gpx3	30210.3869101957	1.26439826061381	0.253774164033456	4.98237582785267	6.28082950416622e-07	0.000165034967333809
Klk1c9	54.9171066488466	2.94326786240147	0.591127213310176	4.97907691632035	6.38882505955269e-07	0.000165034967333809
RGD1305733	94.9968425256964	3.02323506338585	0.608743977100628	4.96634903524655	6.82251169342794e-07	0.000172321484238893
Suox	1940.25601623684	0.456583144144379	0.0922172587005458	4.95116804140781	7.37693690564249e-07	0.00018227448884681
Ak4	1201.85645847819	1.09556877111878	0.223883089725594	4.89348602639699	9.90653512610079e-07	0.000239569528177152
C1qtnf5	102.855021757643	-1.9737622872728	0.405388738919476	-4.86881380211417	1.12270130203236e-06	0.000260420877528567
Trpv5	76.3712373478112	2.72302619646982	0.559013951981905	4.87112385445071	1.10965250115365e-06	0.000260420877528567
Fxyd4	2084.40598389595	-0.441695972398796	0.090954577711297	-4.8562258603498	1.19644226872092e-06	0.000271975256525639
Slc13a2	2300.33578208207	0.689564884208171	0.14224113387632	4.8478584599006	1.24801389588563e-06	0.000272787037319925
Slc5a8	326.644673744042	1.48936784067362	0.307200769749771	4.84819045826863	1.24592753049386e-06	0.000272787037319925
Prss23	3012.43390953241	-0.5732895297898	0.11932934768616	-4.80426266384669	1.5532281818901e-06	0.000333094179535148
Ptprn	1875.8747641975	0.57556547625388	0.121369308810453	4.74226542026999	2.11341480078e-06	0.000444834678253064
Cd180	53.5444066603782	-2.9360840892657	0.620734584248285	-4.73001531374528	2.24502901812571e-06	0.000463945451273032
Ifi27l2b	147.201395997595	-1.74266381780831	0.370227221190067	-4.70701158117616	2.51374632263149e-06	0.000510200726839813
Col4a3	1618.99491413199	-0.705764918231735	0.15013865914069	-4.7007541047132	2.59202488332849e-06	0.000516858856559852
Slc22a6	6969.91099777107	1.06742900331615	0.229697135305724	4.64711500165386	3.36609528696006e-06	0.000659638603992897
Mpst	2330.61197545269	0.547317398243559	0.118142056314334	4.63270587391286	3.60917130648379e-06	0.000695285441855843
Slc16a2	28.0646383490971	2.44073750313339	0.529301268214593	4.61124438897782	4.00265625911838e-06	0.000758236517352325
Gls	2071.68122667794	-0.597697064250047	0.129953955307816	-4.59929875034821	4.23915490564659e-06	0.000789872699304575
Gatm	10984.8878904387	1.36841572038324	0.29825759508435	4.58803310606807	4.47441694127092e-06	0.000820261660556215
Pygl	1213.14548236119	0.478961177448321	0.104597101629834	4.57910563471777	4.66968333649132e-06	0.000842470171469212
Nfat5	3301.0772538236	-0.991548992546049	0.216727357680731	-4.57509842392272	4.75996158009242e-06	0.000845339426864538
Ugt1a6	457.184052221684	1.11318078461335	0.24380965807011	4.56577804761632	4.97645093206153e-06	0.000870189866058635
Prodh2	456.219750875983	1.3472918246131	0.295800893473814	4.55472533835456	5.24540693113748e-06	0.000903322654231948
Tmem52b	455.349403576986	1.48906455592504	0.327579429086493	4.5456595369176	5.47634979157869e-06	0.000929017787031095
Rxrg	22.7595232324	2.61816349155586	0.578229030402308	4.52790045794527	5.95726310130583e-06	0.000995739006021207
Entpd5	1892.2351639071	0.751421989895078	0.166888460273248	4.50254013168295	6.71460704968903e-06	0.00109026033895379
Slc13a3	10993.4551086406	0.811634924478214	0.18017893774927	4.50460489231907	6.64965467852018e-06	0.00109026033895379
Cxcl10	290.956642912343	-0.812878116781028	0.180886369699477	-4.49386052764249	6.99434475186618e-06	0.00111968623168607
Alx1	43.7283945837422	-2.95747848466502	0.65879603538197	-4.48921718684945	7.14853849482901e-06	0.00112847622961426
Agxt2	26067.5321750093	0.543345251536537	0.121505798360292	4.47176397232826	7.75770230909767e-06	0.00118113492434894
Bnc2	67.2150716821511	1.69120169000473	0.378279784260951	4.47076941557649	7.7938693758728e-06	0.00118113492434894
Slc4a4	40.3685564613872	1.93380130824553	0.432192433907339	4.47439880139162	7.66266073049737e-06	0.00118113492434894
C2cd2l	1152.93344434897	0.414417635305847	0.092805518746446	4.46544172053041	7.99037216895602e-06	0.00119498118516255
A4galt	401.550135727443	0.852252083115977	0.191055182241384	4.46076402177472	8.16679774198537e-06	0.00120550419656371
Hmx2	85.3328742924136	1.33291419242026	0.300014485792482	4.44283278155517	8.87821651775148e-06	0.00129371549924056
Fam84a	367.992927693753	-0.803688736396705	0.182023578348295	-4.41530016984326	1.00869923077574e-05	0.00145125005784773
Olfm1	137.163087826683	1.61814411659973	0.368420459361075	4.39211253198577	1.12254567797544e-05	0.00159485677198361
B3galt2	11.9753619710432	3.93923275310023	0.898948565354106	4.38204465185227	1.1757070508567e-05	0.00164976374568361
Rassf6	460.774078252129	-0.657456661598995	0.150978342210347	-4.354642208768	1.33284432583917e-05	0.00184745226920586
Snca	389.391554964961	-1.38296398162603	0.31848281478818	-4.34235041079321	1.40966463024084e-05	0.00193039134786957
Aif1	181.94999718621	-1.48415772764395	0.342627623509127	-4.33169314383787	1.47967066487215e-05	0.00200213533058773
Slc38a2	3934.16574258014	-0.785390003567149	0.18146859232266	-4.32796658371982	1.50492294982318e-05	0.0020123475585518
Fam55b	253.237123600202	-0.918383909128696	0.213252855331273	-4.30654917938638	1.6582107613839e-05	0.00219153761789411
Cpne8	165.772827641446	-1.40332766881021	0.326404359048292	-4.2993533324798	1.71297192158133e-05	0.00223788952421763
RT1-CE5	1504.94373680215	-0.438114641828909	0.1020430647786	-4.29342888494651	1.7593468420082e-05	0.00224682429283879
Slfn2	372.133722839807	-0.670000749817738	0.155992592918126	-4.29508053737778	1.74629928918825e-05	0.00224682429283879
Serpine2	208.933678558065	-1.92396794516322	0.452293161163142	-4.25380728776761	2.10166191989616e-05	0.00265416548683775
Hnrph1	3112.41892822397	-0.632637849270111	0.149132829799332	-4.24210987025035	2.2142823722049e-05	0.00276566301565725
Ccdc39	328.544886439025	-0.788175676404382	0.18618481158972	-4.23329738701361	2.30289658224555e-05	0.00284507853845684
Slc12a3	1786.34781379511	2.92299783187665	0.691711139033657	4.2257492570673	2.3814705371785e-05	0.00288950244872107
Tes	295.321265085841	-0.705660261595191	0.167021266592907	-4.22497251990758	2.3896993681135e-05	0.00288950244872107
Rbm47	1076.92935482651	0.877616726966865	0.208125114308353	4.21677475053112	2.47821296399941e-05	0.0029649861630334
Klk1	3291.40306460442	2.67156603093792	0.635139602615233	4.20626586649228	2.59624787892141e-05	0.00307384931164799
Fgl2	326.350636978085	-0.881004664324375	0.209602199634928	-4.20322241779357	2.63141794101828e-05	0.00308337075439317
Gpm6b	55.3770443011257	-1.85175116833271	0.440970554410548	-4.19926262606803	2.67785589301296e-05	0.00310576633469237
Cd24	7176.77297314825	-0.473952724548355	0.113149754965951	-4.1887207328993	2.80531364708428e-05	0.00322072675886464
Enpp5	2138.65452325035	0.607645586728445	0.145506596111277	4.1760690097083	2.9658987600252e-05	0.00337104053064464
Ccdc62	210.814864736003	-0.713644842863838	0.171012018187236	-4.17306836343216	3.00524719123657e-05	0.00338194451243514
Tsc22d1	5062.74058113591	0.641952176231045	0.154239178512498	4.16205650485247	3.1539427873265e-05	0.00351448173732873

Clec10a	90.8317012242046	-2.30420043991372	0.555727175263901	-4.14627994180689	3.37920641111465e-05	0.0037289378707504
RGD1309079	717.18177944204	-0.616258500811073	0.148762907851384	-4.14255481901928	3.43458237130927e-05	0.00375360223387512
Pm20d2	281.702877289516	1.02920613309696	0.249213025004307	4.12982480782927	3.63039771271407e-05	0.0039298190859722
Ctss	303.969086367555	-1.21584944072189	0.296066933164123	-4.10667083867786	4.01402606047523e-05	0.00430409624560014
Fcer1g	211.949081337005	-1.56964550918931	0.383172695931592	-4.09644404691493	4.19544832282957e-05	0.00441532089234082
Nfia	207.145200962841	0.826390936282648	0.2016573233634	4.09799615753819	4.16742197361498e-05	0.00441532089234082
Trim34	324.608249836907	-0.63543050504103	0.155941650438836	-4.07479658739576	4.6054584675899e-05	0.0048023523800575
Hfe2	11.5921655666602	2.99043957532339	0.73677166311355	4.05884173488159	4.93167223388332e-05	0.00501002898715229
Lgals9	244.515729031178	-1.01859419373729	0.250751191017043	-4.06217090976073	4.86184598782544e-05	0.00501002898715229
Vcam1	257.334581760593	-1.3556723179306	0.334024924335867	-4.0585962877652	4.93685770333501e-05	0.00501002898715229
Folh1	89.3048203723261	1.36613813966815	0.337229104715584	4.05106831102357	5.09843189017945e-05	0.00512821034192741
Ccnt2	748.711610477569	-0.455636470549302	0.112808264098278	-4.03903449974509	5.36716713177186e-05	0.00534078352811182
Gbp2	284.219019976668	-1.14152494764891	0.28273478714748	-4.03744073789361	5.40374895066742e-05	0.00534078352811182
Col11a1	124.655787750864	-1.07199335245366	0.265940192797014	-4.03095651386507	5.55503217675912e-05	0.00539645262573027
Gpc1	421.515737057522	0.763818971396001	0.1894555783733	4.03165205244569	5.5386145015574e-05	0.00539645262573027
Slfn3	738.812177820176	-2.10252553976	0.521959418775033	-4.02813985940582	5.62198978281599e-05	0.00541521490436326
Ms4a6bl	32.8128757742434	-2.4783539288471	0.617482673653623	-4.0136412479119	5.97891968840352e-05	0.00571062194776423
Mapk4	34.7407481375217	1.55211368737259	0.387383377155445	4.00666053037629	6.15832747766819e-05	0.00583296250926472
Gpd1	5417.76685577709	0.5290236281384	0.132199002434814	4.00172178605701	6.28832132064132e-05	0.00590686447358754
Col7a1	110.047834592905	-1.61137940376771	0.403352488741452	-3.99496581462921	6.47035883902159e-05	0.00601772717672682
Zap70	15.9825936815532	-2.40075092340428	0.601174077794307	-3.99343719578291	6.51223335155199e-05	0.00601772717672682
Slc16a14	314.804782677806	1.36196084954907	0.342466712493435	3.97691454341034	6.98152401883231e-05	0.00639935499984258
Atp2c2	73.0909321308119	-1.26070885665979	0.317601974896782	-3.96946164163341	7.20352015583996e-05	0.00655001680730216
Pc	7235.41201555549	0.635649447586923	0.160765497687017	3.95389220157439	7.68900619695925e-05	0.00693597178052689
Cldn8	1691.42581571028	-0.475921745213112	0.120593074257199	-3.94650976554486	7.92986889172566e-05	0.00704147576744952
Pla2g4a	276.600963739823	-1.30126242369093	0.329659926210412	-3.94728725037805	7.90417021705981e-05	0.00704147576744952
Dusp9	33.4890048892681	1.7044180626027	0.43255391558462	3.94035980531833	8.13594873943512e-05	0.0071524234969566
Hba2	4038.23195563977	0.837024261279496	0.212494230794483	3.93904464205918	8.18067090097095e-05	0.0071524234969566
Itgb8	235.135348902816	1.18026566664965	0.300573340472841	3.92671440784781	8.6114129392871e-05	0.00735919695247648
Mx2	699.161737165476	-1.09847964672128	0.279610414558562	-3.92860776826043	8.54390611439567e-05	0.00735919695247648
Slc48a1	1086.19953684586	0.642315927463515	0.163512341871393	3.92824125758479	8.5569347149919e-05	0.00735919695247648
Dpep1	62.8226864620722	-1.55878012580489	0.397768103182338	-3.91881629857672	8.89849006171307e-05	0.00754777895831573
Bst2	650.855236059266	-1.11592124022392	0.285068356247187	-3.9145742267384	9.05638751074406e-05	0.00762480744052718
Clic2	215.580161581436	-1.36332633039141	0.349356359130537	-3.9023944884942	9.52457607851647e-05	0.00796002439032487
Insrr	471.807417243302	0.885959928976747	0.227195967988136	3.89954072170422	9.63753278435273e-05	0.00797480397734484
Nox4	385.49950672941	0.814629167394292	0.209058197668983	3.89666215665052	9.75275165274444e-05	0.00797480397734484
Pdk2	5078.67213871119	0.377537645214271	0.0968679959447488	3.89744457425969	9.72130616956639e-05	0.00797480397734484
Fyb	20.5856932151681	-2.28908922864558	0.588625827878592	-3.88886983925843	0.00010071210946877	0.00811839600157478
Ivd	4558.76501504517	0.350568091848458	0.0901301280990989	3.8895772062257	0.000100419018218287	0.00811839600157478
Pgam2	68.5355155980865	2.30757232748975	0.594056091743798	3.884435088808	0.000102568089997845	0.00815237000640218
Tll1	26.2528093536876	-2.6010765837376	0.669351288154634	-3.88596635244945	0.000101923624320316	0.00815237000640218
Kcnj15	223.737347393316	1.19671915545392	0.309194224588682	3.8704447246578	0.000108636988257335	0.00845731512693748
LOC498222	363.377508242553	-1.07462832556127	0.27753466386583	-3.87205082994888	0.000107923437761497	0.00845731512693748
Zc3h11a	1266.60114476157	-0.608021011521601	0.157083050262026	-3.87069776470075	0.000108524274573452	0.00845731512693748
Lpar3	600.707829299174	0.794021625149839	0.205326356265878	3.86711983590484	0.000110128318362396	0.00845755720612836
Shpk	543.344726877963	0.503157819328346	0.130103462033497	3.86736687451714	0.000110016851981689	0.00845755720612836
Fhl2	157.666836344967	-2.01179407400824	0.52125412010132	-3.859526469771	0.000113606951633186	0.00866615176015297
Trps1	226.304631249503	0.726727872911012	0.188481966090827	3.85568915681203	0.000115404054399302	0.00874454988201642
Srsf11	1952.01131753392	-0.468889179982646	0.121727223583847	-3.85196643920551	0.000117173084653842	0.00881979655745406
C3	131.818817426929	-1.73926315380923	0.45307098696685	-3.83883144990806	0.000123621253051961	0.00924394185650388
Tnip1	2707.34275100058	0.556684734082193	0.145217877086407	3.8334449260056	0.000126361101670973	0.00938706066400186
Sept4	1416.91861068031	-1.07662752960975	0.28101573538345	-3.83120015731744	0.000127519711948126	0.00941161718183377
Hsd11b2	5289.65078617658	0.63763854826053	0.166791383866992	3.8229705484608	0.000131853533696323	0.00960671323072058
Klk1c7	1676.32565187624	2.65864999040926	0.6952434453812	3.82405617495829	0.000131273984879993	0.00960671323072058
Csmd1	60.9218885643303	2.43156905976988	0.637049194342929	3.81692509991771	0.000135125167899772	0.00965932489527553
Icam1	323.260698176494	-0.527602274525013	0.138192827910864	-3.81787016374918	0.000134608730250548	0.00965932489527553
Lepr	40.0263629474455	-2.31874475199741	0.607395943197226	-3.81751768013454	0.000134801129895655	0.00965932489527553
Slco2b1	116.849219238568	-1.58947461330381	0.416774869949113	-3.81374868762572	0.000136874657640919	0.00972323349216681
Ankrd10	577.697844132187	-0.57314769192106	0.150501493853661	-3.80825251128974	0.000139952321440005	0.00988011233221802
Ptbp2	153.814873652079	-0.71964086975359	0.18905319997672	-3.80655217601292	0.000140917574187159	0.00988684659389659
Nrip3	471.390633687558	-4.5804938643428	1.20434751708934	-3.80329913031491	0.000142781774083861	0.00991659799407031
Rxfp2	12.7577241401253	2.9123925651167	0.765860612546268	3.80277104920414	0.000143086580241732	0.00991659799407031
Tspan2	64.8738094664551	1.64394175120246	0.433170034877663	3.79514190464895	0.000147559033418504	0.0101645816596044
Gsg1	11.6060226947109	3.04012147562073	0.80288107435279	3.78651530436361	0.000152774690692735	0.0104604646651423
Susd2	1301.50332896325	0.846514405447352	0.223857627511499	3.78148564718381	0.000155895211743422	0.0106102094411721
Rtn4	2644.81475122967	-0.452831140158585	0.11980426260934	-3.77975816799763	0.00015698075200848	0.0106204954007642
Fam13b	1235.38430307571	-0.424676653730378	0.112660660188367	-3.76952037224284	0.000163561558531766	0.011000240676166
Lrrc8d	2309.17883102054	0.413739204838665	0.109884900385401	3.76520525920807	0.000166412320709515	0.0111261319834374
Lhfp	240.80431030568	-1.24922697034433	0.331964283523491	-3.76313667568376	0.000167795436569362	0.0111529996026162
Acly	941.070402555677	0.576009888677378	0.153168435688843	3.76063048556246	0.000169485636883877	0.0111715025640903
Tnfaip2	443.794031604425	-1.12012559362442	0.297920463516375	-3.75981421485285	0.000170039586801656	0.0111715025640903
Myom2	14.7997505177343	2.8184402932975	0.750119120636601	3.75732362468722	0.000171740332589289	0.011187743590771
Slc26a1	2277.79090853629	0.68957395392715	0.183564591735145	3.7565738980974	0.000172255422170061	0.011187743590771
Tmed6	600.934613263244	-0.717265055648445	0.191461666500135	-3.74625933618905	0.000179491036259938	0.0115914495348321
Myc	743.037065958291	1.03279103617785	0.276655426951361	3.73313130907576	0.000189113939757144	0.0120756687599983
Pdpn	115.965737261632	1.74342808546844	0.466916539271557	3.73391803209281	0.000188523872063261	0.0120756687599983
Nckap1l	87.3930035478874	-1.67309743109293	0.4491887111331	-3.72470943642475	0.000195540315295734	0.0124162638192811
Jun	3995.70841061569	0.642703050099983	0.172696825487368	3.72156840918303	0.000197989211484476	0.0125019187651809
Lyz2	2263.31251346937	-2.31990733727935	0.624743023044264	-3.71337854398893	0.000204510634896594	0.012771801517773
RT1-N3	295.633753601176	-1.32764695660044	0.35743712164792	-3.71435107377623	0.000203725803254746	0.012771801517773
Trpm2	52.3531741041701	-1.35565951603102	0.365598559180684	-3.70805486506591	0.000208857410659164	0.0129719854073883
Clec4a1	44.5844260389587	-2.07366887022766	0.560717696167948	-3.69824045932473	0.000217099179001423	0.0133653748330537
Ctsk	131.082665507556	-1.27464268742629	0.344710265343219	-3.69772187131464	0.000217543053327022	0.0133653748330537
Kcnt1	1522.86934557766	-1.03595645206117	0.28112876375128	-3.68498917804691	0.000228712358073763	0.0139760465691741
Gcom1	145.848456157007	-1.07783491636654	0.292796675834408	-3.68117197128328	0.000232164342413503	0.0141111225447694
Erc2	37.6316487862381	1.39347220231144	0.37903911630578	3.67632822673449	0.00023661505013334	0.0141545613674502
Lamc2	225.414962321005	-0.917381879894371	0.249468667318582	-3.67734308983514	0.000235675958330768	0.0141545613674502
Rab31	238.517769380922	-1.15655604805591	0.314364634621491	-3.67902722088463	0.000234125280537075	0.0141545613674502
Lig4	468.854248065615	-0.924152748388607	0.251998256104256	-3.66729818958061	0.000245126877907074	0.0145869743156639
Ly6i	1024.09469738622	0.792033834931643	0.216057810497524	3.66584217949724	0.000246525932113636	0.0145938215854353
Cldn6	303.940744352793	-0.994913175436503	0.271773302888452	-3.6608201205284	0.000251409223337361	0.0148057887691837
Ogn	214.584563476155	-1.05367322527037	0.288562584164188	-3.6514547730514	0.000260759024220493	0.0152772529344852
Adora2a	89.7617964743025	-2.0375360429891	0.558634873324628	-3.64734845653839	0.000264960487686251	0.0154437995027791
Ccl5	29.9037906745215	-1.71752392107199	0.471701938964811	-3.6411211809755	0.000271453320922314	0.0157205765287751
Slamf8	13.5227879210654	-3.16082335912233	0.868321377031784	-3.64015379873187	0.000272475239852956	0.0157205765287751
Acot12	32.7673435141388	1.91696203425385	0.52731178519133	3.63534836142208	0.000277605248199576	0.0159356628840221
Abat	1468.22182776793	0.608296684497402	0.167450332947248	3.6326991639308	0.000280471943498514	0.016019317134694
Adamts9	265.054975747669	-0.593681935840702	0.164048296495426	-3.61894605749383	0.000295805283948263	0.0164809943988037
C1s	541.764856848824	-2.60384615762142	0.719323189062353	-3.61985571605938	0.000294767334584182	0.0164809943988037
Cd53	78.2831131479645	-2.01752600634366	0.557184441643273	-3.62093026214709	0.000293545638483799	0.0164809943988037
Ryr2	20.0512305243702	-2.0043051470267	0.553507402252604	-3.62109908353492	0.000293354130099572	0.0164809943988037
S100b	62.7321124017594	2.37618357218043	0.656169963956155	3.6212928093417	0.000293134514729978	0.0164809943988037
Bend7	139.116225749697	0.790637435257083	0.218580327414782	3.61714818807439	0.000297866787036637	0.0165148970802849
Cfb	231.519254935696	-1.08270668483896	0.300132720504658	-3.60742635131033	0.00030924927787002	0.017062753845974
Akna	112.740685212546	-1.2062972365882	0.335731233210939	-3.59304442738663	0.0003268368358389	0.0173281435505077

Btg1	1547.48317327881	-0.505146234605908	0.140360639601102	-3.59891659115767	0.000319545648989295	0.0173281435505077
C1qc	220.998631291047	-1.58136367856585	0.439665256249306	-3.59674469630859	0.000322224461470646	0.0173281435505077
Clec12a	26.6220257147482	-2.61113534885015	0.72579317129372	-3.5976300854358	0.000321129895714416	0.0173281435505077
Cryl1	5255.80236307146	0.536920958577584	0.149428416549275	3.59316501490553	0.000326685554851356	0.0173281435505077
Ewsr1	1526.1353367161	-0.336611115206395	0.0934591414237883	-3.60169278337406	0.000316151845961518	0.0173281435505077
Pik3r3	104.670088243728	-0.955834063989257	0.265582290205859	-3.59901280785089	0.000319427459007421	0.0173281435505077
Slc25a1	1779.78169655237	0.39331710987697	0.109442116734789	3.59383683002126	0.000325843939663882	0.0173281435505077
Supt20	1118.01009455342	-0.389301905859507	0.108371406508622	-3.59229356157275	0.00032778029767369	0.0173281435505077
Ddc	52.840937423946	1.71573911700195	0.478113129399248	3.58856306489174	0.000332505566569678	0.01746940385187
Il2rb	16.4089185985702	-2.09024961749336	0.582605154339094	-3.58776368853882	0.000333526362471915	0.01746940385187
Csf2rb	3451.36684327454	0.663857206329983	0.185173073161946	3.58506339498613	0.000336996336032001	0.0174899559604554
Pcsk9	84.3971934180838	-5.34016165390429	1.48915551735185	-3.5860335550451	0.000335745776288222	0.0174899559604554
Esm1	235.460322882304	-1.24318595351408	0.346946362547016	-3.58322232977903	0.000339381504817395	0.0175336826534296
Chst8	15.7065476140287	2.47354506484457	0.694456805469817	3.56184149303737	0.000368262705237698	0.0188230517189145
Igfals	106.238588083437	1.15135233505154	0.323256648997004	3.56172823861144	0.000368421632251164	0.0188230517189145
Zhx2	378.464433724389	0.639910221782704	0.17969462346243	3.56109832032061	0.000369306751127744	0.0188230517189145
Hs3st1	75.3228404637258	-1.76511961646455	0.4966275243099	-3.55421222155842	0.000379113160126029	0.0192366079374663
Cebpa	123.180119835388	0.734106661841882	0.206819196511039	3.54950930196995	0.000385949829642806	0.019239937560176
LOC287167	76.7739833784386	1.26433157369242	0.356094060601373	3.55055507400827	0.000384419695215579	0.019239937560176
Ndufa10l1	3619.53824510333	0.444277831818446	0.125114576445074	3.55096779641408	0.000383817377031954	0.019239937560176
Pi16	92.5519842871354	1.52697616577042	0.429902764724651	3.55191055062982	0.00038244484863564	0.019239937560176
Fcgr2a	76.4759223762132	-1.83278053423559	0.516717982625371	-3.54696487419209	0.000389696545253949	0.0192876984870446
Zfp202	175.618297847521	-0.758236135159581	0.213795057236064	-3.5465559632764	0.000390301834596187	0.0192876984870446
Dbt	2026.52162631207	0.375510043970556	0.106001118688459	3.54251019816304	0.000396338097619609	0.0193338146675728
Pdcd4	1133.71701190917	-0.32073846388772	0.0905390049458513	-3.54254460913883	0.00039628639081959	0.0193338146675728
Stard13	438.839776766125	-0.768541244641741	0.216869158235124	-3.54380148332807	0.000394402093633961	0.0193338146675728
Idua	1517.17745960531	-0.476174633568915	0.134563671616258	-3.53865666601938	0.00040216855652498	0.0195343923652262
Lrrc59	1661.05312713101	0.317043559198772	0.0896856199084413	3.53505455526134	0.000407690977456683	0.0196422421282688
Srpx2	57.023803570956	-2.34649311690464	0.663797246186181	-3.53495458196959	0.000407845252707324	0.0196422421282688
Nfxl1	193.431986760457	-0.720216836229078	0.20397196941798	-3.53095985827939	0.000414054580312666	0.0198571491976108
Clec7a	48.50746170699	-1.39813507829706	0.396566271335513	-3.52560260253241	0.000422520471196276	0.0201760439403436
Ttc3	1434.33231372173	-0.411180699794416	0.11666296589754	-3.52451779903778	0.000424254311256564	0.0201760439403436
Ch25h	73.4627437946828	-1.7560674364726	0.499466705472684	-3.51588487727265	0.000438290986680058	0.0206973409116146
Ephb1	71.5886957314146	-1.22711809982354	0.349055202106566	-3.5155416461861	0.000438857923605412	0.0206973409116146
Cxxc1	652.134782158096	-0.412667904828984	0.117568298368487	-3.51002702731636	0.000448061148357444	0.0210440620340112
Ifit3	126.059774501369	-0.99700287353798	0.284217777966857	-3.50788356966975	0.000451686686376865	0.0211270406475697
Cd3g	16.4032152122151	-2.51338300530244	0.717203136690293	-3.50442277330388	0.000457598267075066	0.0212354085502705
Prss35	158.427725161856	-1.81148757547714	0.516926860321167	-3.50434019689296	0.000457740198382568	0.0212354085502705
Olfm4	173.202698433563	-0.885791500582039	0.253014224047291	-3.50095534714473	0.000463593513082715	0.0214195279256022
Bmp7	1152.96337237262	0.460327958218757	0.131761590493778	3.49364299940274	0.000476477723787765	0.021752915715709
Kng1l1	24.6611084188088	2.28841532005956	0.655111903313168	3.49316705815619	0.000477327794128215	0.021752915715709
RGD1562618	395.891886712455	0.67256879612604	0.192550494983643	3.49294763528484	0.000477720177724176	0.021752915715709
Slc12a1	5567.07391941226	-1.06915171932475	0.306125121000078	-3.49253179821356	0.000478464625103577	0.021752915715709
Trim63	71.456121450025	-1.13700098943597	0.325712324358951	-3.49081353207543	0.000481552218730602	0.0218060658091315
Ccnl1	981.511697193854	-0.543072947481782	0.155708094495708	-3.48776310724649	0.000487079425811559	0.0219688283879928
Pde9a	221.290185588145	0.980776039561794	0.281337483639391	3.48611932855317	0.000490082340341055	0.0220169007127132
Csf1r	418.990955556866	-1.20211431295509	0.345166663376959	-3.48270687903094	0.000496371530811925	0.022211648894521
Cdc42ep2	376.341233773479	-0.970272654776689	0.278956316679359	-3.47822435543537	0.000504747266898582	0.0224943052795303
Heph	146.579792606345	-1.20018271286069	0.345671920822317	-3.47202836147519	0.000516541762973554	0.0224943052795303
Hnf4g	267.931612494414	0.915179766920776	0.263460127008815	3.47369363748222	0.000513346803756246	0.0224943052795303
Igfbp4	967.236680512832	0.741433950716813	0.213526893371811	3.47232116296361	0.000515978661391606	0.0224943052795303
Leprel2	461.910770456658	-0.59141868379228	0.170234011061648	-3.47415114114949	0.000512472279055203	0.0224943052795303
Lpar1	172.737292148241	-1.04433939080486	0.300720689255528	-3.4727886311722	0.000515080835096903	0.0224943052795303
Nipal1	72.9113145127788	-0.967157147967515	0.278403185509672	-3.47394425892413	0.00051286756519483	0.0224943052795303
Gas7	92.4666814280494	-1.87942333678949	0.541820232821901	-3.46872121589315	0.00052294180033129	0.0226101914154495
Tia1	125.466730652762	-0.704532145707551	0.203117260180139	-3.4685981146197	0.0005231814483779	0.0226101914154495
Frmd6	354.423871115721	-0.693816205946076	0.200622774470958	-3.45831228670658	0.000543570915505441	0.0234023750971017
Hgf	31.880219540585	-2.25248368876743	0.65185786350806	-3.45548288187457	0.00054930807087682	0.0234217938056909
Resp18	1249.67240753094	0.819478610566528	0.23717276201018	3.45519697802125	0.000549890922428132	0.0234217938056909
Slco4c1	266.617970191153	0.960762042363605	0.278075236873627	3.45504350968234	0.000550204024821351	0.0234217938056909
Slc4a1	9229.34341957763	-0.569149215964714	0.164788743097263	-3.45381125717298	0.000552724063241188	0.0234412750104453
Arhgef37	122.310956162777	-0.906468388921615	0.263002170613858	-3.44661942069101	0.000567647621323398	0.0238958624591176
Mrc2	140.961001845399	-1.90069382629282	0.551369627129653	-3.44722257587445	0.000566381762069078	0.0238958624591176
Ddah1	6877.74100606283	0.663549374448936	0.192653490013457	3.44426345145674	0.000572617449677299	0.0240161252141409
Adamtsl4	229.039398305972	-0.882101201661278	0.256812762667792	-3.43480282092658	0.000592985128059393	0.0245175456054739
C1r	794.635736181639	-2.27516253596113	0.661967901991155	-3.43696806010924	0.000588264969041583	0.0245175456054739
Mecr	524.919001524186	0.486685088842094	0.141625392653858	3.43642534521748	0.000589444775949608	0.0245175456054739
Mrc1	73.7798150305993	-2.11079425841085	0.614549087491187	-3.434704080398	0.000593201217799167	0.0245175456054739
Akr1d1	273.435502138551	-0.555975076375622	0.162092751713842	-3.42998110956337	0.00060362327158827	0.0246790723196845
Nfkbiz	180.974624900849	-0.844564505008169	0.246204054178689	-3.43034361406252	0.000602817343952341	0.0246790723196845
Vasp	872.546326179958	-0.540579532357338	0.157552683659396	-3.43110329701514	0.000601131648228989	0.0246790723196845
Dixdc1	236.130860886664	-0.697761965206184	0.203708435584958	-3.42529735306508	0.000614126880036352	0.025018516553739
Mpeg1	47.3019918600669	-2.4369099744881	0.712146040582465	-3.42192448685799	0.000621795801837947	0.0252404681560361
Pla1a	77.9666802795608	-1.65211094902211	0.484101742745034	-3.41273497520178	0.000643144548836984	0.0260141670536696
Fam133b	804.205630165397	-0.545618328297789	0.160061321158881	-3.40880810146628	0.000652473598366993	0.02629792524482
F2	1240.51354176694	-0.515189437680783	0.151298778732558	-3.40511299560094	0.00066136681736753	0.0265576261704958
Gde1	3357.38319268266	0.407211186584962	0.119620355619802	3.40419642188016	0.0006635901664984	0.0265576261704958
Ppp1r18	179.150534111022	-1.15358946082975	0.33898404105	-3.4030789687223	0.000666310193551735	0.0265729181049439
Ifitm3	527.61552094283	-0.953046213285867	0.280209317224654	-3.4011938743699	0.0006709222657219	0.0266632953573256
Pgf	36.2191638347351	-2.50169768200028	0.737541088615458	-3.39194347354473	0.000693987558212695	0.0274838417653153
Homer2	105.412304153789	0.851295953039288	0.251065838006688	3.39072794529939	0.000697072597455967	0.0275101636898768
Tnfrsf26	56.0682162590237	-1.29845828046514	0.383192378811016	-3.38852845793554	0.000702687367788863	0.0276357945407897
Fbn1	171.782447282682	-1.5330309324571	0.45267513822223	-3.3866028924798	0.000707637353107555	0.0277345039842085
Car6	84.6050656586092	1.15504797073249	0.341160714654984	3.38564178440237	0.000710120144952366	0.0277361703351498
C1qa	187.54389877372	-1.4295836676798	0.422667477578527	-3.38228925459305	0.000718844100299884	0.0278858656956991
Fads2	572.769863107803	0.795140510891746	0.235089859807544	3.38228331729274	0.000718859638293141	0.0278858656956991
Ctnnal1	1328.23875706345	-0.462727118295767	0.136927943920412	-3.37934759733719	0.000726580792154266	0.0280895145701544
Siglec1	19.5426563894578	-2.28769195217516	0.677235894735085	-3.37798390481066	0.000730193543827056	0.0281334909123333
Acbd5	600.764126908594	0.734468018024121	0.217684173395602	3.37400742813468	0.000740823686101591	0.0284466284332118
Eltd1	440.122151446279	-1.15357454938445	0.342269325528685	-3.37037082596456	0.000750670910841025	0.028727695530704
Pf4	50.57432710891	-1.47921733896261	0.439018963977771	-3.36937002802801	0.000753402131479327	0.0287354651892417
Atp2c1	1722.01951158965	-0.391377706792418	0.116244751274566	-3.3668419649159	0.000760342473948253	0.0288068418629861
Tra2a	856.777122150381	-0.622337876155676	0.184801117198061	-3.36760884128576	0.000758230905430231	0.0288068418629861
Cntn1	20.6560487713955	-3.74939671295535	1.11564525853222	-3.36074274889866	0.000777331891373223	0.0292554777395631
Mxra8	251.897186971931	-1.30593478246488	0.388561385676057	-3.36094843854	0.000776753245409266	0.0292554777395631
Tnfrsf11a	35.3214846994198	-2.25027654053329	0.669902484585512	-3.35911060536759	0.000781937641871869	0.029331693853187
Marcksl1	215.828141246291	-1.19298522796989	0.35541142875346	-3.35663158653638	0.000788981670524372	0.0294985712736185
Ccdc64	886.554889512268	-0.795846669750227	0.237462308209539	-3.35146523147566	0.000803851322903996	0.0299559807741863
P2ry12	7.52716221379931	-5.87794213636713	1.75561651468076	-3.34807863062052	0.000813739200646822	0.0302253586750058
Hnf4a	7017.91093458403	0.359154901923696	0.1073141549076	3.34676168519369	0.000817614677799675	0.0302703857585378
A1cf	89.307917597186	1.0440539801862	0.312459335298834	3.34140754408146	0.000833547725684699	0.0307185505542768
Dkk3	60.0024414480594	-1.9791576964378	0.592405631130784	-3.34088265275262	0.000835125120646799	0.0307185505542768
Ddx19a	3207.16203519417	0.694150397450168	0.208143175283542	3.33496592672119	0.000853098517251293	0.0312784443454135
Cd8a	21.5855082225029	-1.48286199403328	0.445349511285159	-3.32965896774903	0.000869524126123713	0.0317781711174345
LOC100910940	12.8566173302971	-2.47367472185261	0.744191351175124	-3.32397671371284	0.000887436056447791	0.0321383041247758

Ndrg1	21557.2326367237	0.941722901675852	0.283323502102502	3.32384322051459	0.000887860944499349	0.0321383041247758
Slc34a3	44.4622128225325	1.82036297867144	0.547645983388053	3.3239775948134	0.000887433252668221	0.0321383041247758
Tead2	50.7833810512817	-1.19584836265468	0.359880703012839	-3.32290215241693	0.000890861572246224	0.0321445480322241
Cpe	214.18500284808	0.818425486712625	0.246439681150349	3.32099718232194	0.000896964428852974	0.0321863871574202
Lcp2	41.2058203134357	-1.46819414174452	0.442169971083976	-3.32042933206263	0.000898791105488741	0.0321863871574202
Tmem200c	17.8067286686322	-3.38180711759404	1.018649145482	-3.31989393265908	0.000900516550770685	0.0321863871574202
Fam198b	94.6174078505951	1.13847964486654	0.343906397809598	3.3104346185989	0.000931512110394038	0.0331898640963594
Cd3e	9.0153501930541	-3.64245135763141	1.10124329124795	-3.30758097377713	0.000941055016541297	0.0334250978687762
Zfp189	298.727529005724	-0.732613375408538	0.221609591260448	-3.30587395266449	0.000946806694818457	0.033524625835846
Slc31a2	560.20038505969	-0.619244640681727	0.187451176501587	-3.30349828813417	0.000954865523996788	0.0337049737445574
Cd55	88.864311226227	1.20204604692628	0.364172444718685	3.30076057197253	0.000964231295256903	0.03388228797498
Tpst1	64.9385251220119	-1.34500322665085	0.407540965098786	-3.30028964407254	0.000965850897755896	0.03388228797498
Abcb1a	659.390452593261	0.789141255713979	0.239247179681528	3.29843493563619	0.00097225409279055	0.0340019692881766
Ulk1	1043.00439096104	-0.350679601352845	0.106425131794258	-3.29508261291874	0.000983927493308024	0.0343046622360092
LOC652956	1760.63591047369	0.39296009201564	0.119610072378247	3.28534281605456	0.00101858357302112	0.0354043452322876
Alox5ap	35.4919802453792	-2.08400289266279	0.634579775181096	-3.28406762107737	0.00102320368754442	0.0354565033921643
St6galnac3	576.081347128376	0.456971362489122	0.139202126250695	3.28279010383896	0.00102785165761774	0.0355093068099795
F2r	670.974053509375	-0.922147714791432	0.280996254352136	-3.28170820966114	0.00103180316852026	0.0355378024648525
Frem1	87.8726371929924	-0.987293119524184	0.301213463173407	-3.277719093704	0.00104649481724719	0.0358266870266009
Tyrobp	116.374468756455	-1.02836494604428	0.313708799225254	-3.27808766787533	0.00104512931079637	0.0358266870266009
Elmo1	74.5725073045854	-1.30649153798233	0.398785672770362	-3.27617471537064	0.0010522344535285	0.0359150054018165
Plod2	294.232896250612	-0.950325888210082	0.290209483157848	-3.27462038066202	0.00105804048897548	0.03596202223572
Tmem106b	536.90355548859	-0.499176732042243	0.152461641439517	-3.27411358902542	0.00105993994799984	0.03596202223572
Alox15b	53.0636226288658	-1.80167786953685	0.55053243229955	-3.27260986607332	0.00106559449693858	0.036046270988702
Slc9a3	1742.76914374317	-0.667675427690021	0.204344375193021	-3.26740301542112	0.00108539047930237	0.0365683452162775
Spon1	1649.05569301731	-1.42742468799792	0.436940471399798	-3.26686306586563	0.00108746266787804	0.0365683452162775
March2	1431.41113983657	0.303447624532529	0.0929418185801367	3.26492023900834	0.00109494904780031	0.0367114775141545
Coro1a	196.268791083396	-1.20263729452519	0.368944935083361	-3.2596660915089	0.0011154344887086	0.0367479084019187
Igfbp7	15767.5761007396	-0.440482144044411	0.135116968029689	-3.26000613000452	0.00111409805882456	0.0367479084019187
LOC685157	47.7342715501718	-1.86537427409878	0.572048662493822	-3.26086641994189	0.00111072352880334	0.0367479084019187
Nes	631.461861874801	1.54630331445275	0.474245785093459	3.26055257222374	0.00111195351552286	0.0367479084019187
Nostrin	184.711707928808	0.867325386149718	0.266005945849011	3.26054886999415	0.00111196803228011	0.0367479084019187
Sphk1	107.29545854273	-1.24673135904545	0.38226701482383	-3.26141495525064	0.00110857680873665	0.0367479084019187
Dok4	292.0153628907	0.509714009419989	0.156728207699393	3.25221615752556	0.00114508902170682	0.0376158434124847
Acrbp	145.533055672499	-0.852165897535902	0.26232590782132	-3.24850070895912	0.00116014919713914	0.0380007371028341
Htatip2	842.72656853878	0.922728501818605	0.284315600314928	3.24543746736558	0.00117270317061587	0.0383015639000573
Ablim3	433.589159236344	0.851616933550861	0.262485712370739	3.24443157632908	0.00117685287896621	0.0383269622416331
Vstm4	62.2715383006411	-1.76673871008998	0.54480915629685	-3.24285796167372	0.0011833719019546	0.0384291572503313
Arhgap30	45.776174036363	-1.8443679055939	0.569315443528853	-3.23962387909547	0.0011968746336417	0.0387569147748478
Fam20a	48.8096759471426	-1.57002653232052	0.485319821808048	-3.23503484047164	0.00121627888346475	0.0392733687200579
Ppip5k1	770.8911085759	-0.356538593025766	0.110272172761063	-3.23325988867845	0.00122386169346964	0.0394062663115465
Amt	1524.18624796288	0.542075633052754	0.167743544445925	3.23157373860967	0.00123110554961559	0.0394081341348752
Epn1	2847.50882583971	0.315499045611238	0.0976749571055898	3.23009146827854	0.0012375062028597	0.0394081341348752
Gcm1	294.496977082508	0.47492113864047	0.147033212822089	3.23002626090424	0.00123778848197699	0.0394081341348752
Serpini1	20.143864243868	-2.01361160561961	0.623271422592286	-3.23071383129468	0.00123481501761098	0.0394081341348752
Atm	202.029019914953	-0.595818620580817	0.184542047437331	-3.22863341365687	0.00124383225881594	0.0394899370215137
Pecr	3362.95264586935	-0.400975785767738	0.124257390210936	-3.22697736599049	0.00125105355905302	0.0396085647693498
Adh1	270.537084653254	0.835925494574819	0.259409712574811	3.22241401942013	0.0012711531040403	0.0397324689018498
Cyb5r2	81.4063275982796	0.950641245587717	0.294798265947406	3.22471790168982	0.00126096854355488	0.0397324689018498
Olfml3	168.197097484005	-1.36048513226714	0.422169088427381	-3.22260717224719	0.00127029634355735	0.0397324689018498
Rcn3	186.22789883419	-1.42708345909783	0.442687415083123	-3.22368201686934	0.00126553842662523	0.0397324689018498
Tlr7	20.2145327151836	-2.07175753521802	0.642979070340429	-3.22212281983162	0.00127244577514282	0.0397324689018498
Sned1	216.873341645062	-0.970664007992918	0.301331438611999	-3.22125036957317	0.00127632595819009	0.0397444406596946
Myo1b	195.750465518637	-0.89124719596887	0.276960626199103	-3.21795631458518	0.00129107480128724	0.040093869375494
Cdca4	186.72907916543	-0.690463570023659	0.21468948679575	-3.2161033142743	0.00129944042559954	0.0402437053879137
Gjb4	22.9155478905881	-2.91722582262243	0.908006210937596	-3.21278179321057	0.00131456121503169	0.0406013662229626
Morc3	522.505614812368	-0.356778287173689	0.111166491586702	-3.20940493921611	0.00133010020818596	0.0408592404493017
Wnt2b	89.7544954046731	0.916785233429564	0.28565204725806	3.20944744569372	0.00132990356080836	0.0408592404493017
Thoc1	379.274905757208	-0.388072930671644	0.12098301351525	-3.20766460840991	0.00133817456016476	0.0409964745305461
Thbs4	48.2284417177977	-1.06306933326486	0.332038670022607	-3.20164314955387	0.00136646161442727	0.0417505449182268
Fbln1	93.1153116493825	-1.86570791770317	0.583741991221051	-3.19611736993693	0.00139290437872484	0.0424443731061301
Tfap2b	351.159833791897	-0.536730670400547	0.167997602335724	-3.19487101564671	0.00139893346624823	0.0425141117042175
Vash2	207.798745182404	-1.1845795861606	0.3709174965707	-3.19364709702986	0.00140487743311784	0.0425808984128463
Calb1	2975.19342180599	2.05108401046809	0.642711563107904	3.1912978203626	0.001416352003548	0.0427009466109457
Tmbim6	8371.4099958561	0.429099695340309	0.134438555684173	3.19179042914119	0.00141393881940513	0.0427009466109457
Ctsa	5264.51096728802	0.504891405605754	0.158306803570726	3.1893222162128	0.0014260682624984	0.0428568316762773
Parvg	18.7636078096283	-1.87032548151738	0.58661237506699	-3.18834985590576	0.0014308729778381	0.0428568316762773
Trem2	14.5668395566678	-2.62096043696922	0.822145057643496	-3.18795377117713	0.001432834421695	0.0428568316762773
Ifit2	260.933375690374	-0.868202739438284	0.272552990519344	-3.18544565511441	0.00144531246661716	0.0430037211925933
Kl	655.301811901685	0.741357020886187	0.232722388463669	3.18558530522268	0.00144461507329123	0.0430037211925933
Nme4	201.829452695507	0.612463869304403	0.192376528462585	3.18367253115039	0.00145419422681056	0.0431550171851926
Usp53	765.567682969591	-0.405471619004585	0.127394991791835	-3.18279088762869	0.00145862916948169	0.043173904011273
Crb1	21.5346496671388	2.49792597852861	0.785567918904412	3.17977086184009	0.00147391549636373	0.0435130481342081
Ap1m1	1021.00629899001	0.274966086608379	0.0865436336629381	3.17719599894882	0.0014870650033829	0.0437875150996116
LOC498276	44.7561714795601	-1.61123954587649	0.507420975065011	-3.1753506950911	0.00149655513977604	0.0439038222602775
Slc30a1	1129.99545519449	0.620278245623384	0.195367699390528	3.17492731684108	0.00149874036925811	0.0439038222602775
Slc1a1	1408.12223176775	0.593511829777965	0.18702784570439	3.17338751105571	0.00150671275249223	0.0440239001152356
Lck	34.9877269064042	-1.72938030057028	0.545369493773781	-3.17102500289029	0.00151902068042662	0.0442697155223819
Cd9	2111.48953338449	-0.310083670970493	0.0978437561430455	-3.16917178156118	0.0015287401271654	0.0444146295758608
Fam102b	122.76096967124	-1.04778980479625	0.330680251276828	-3.16858899420365	0.00153180844569219	0.0444146295758608
Slc22a3	18.1508198221699	2.72605922195879	0.861091795240445	3.16581720674459	0.0015464794269947	0.0447259164560351
Lysmd2	69.1920216058166	1.21220909417371	0.383130163536977	3.16396151893354	0.00155637372166915	0.0448978267017553
RGD1563986	28.544997596039	1.3249517024967	0.419019830163558	3.16202625059421	0.00156675439939913	0.0449690164231578
Spry1	172.346710196975	-0.973665779674084	0.30785408580642	-3.16275087635618	0.00156286010128132	0.0449690164231578
Fam65a	1056.99033403494	0.384042611902522	0.121590251044059	3.15849838786304	0.00158584186470154	0.0454022131843772
L3hypdh	405.501409075143	1.07555047449041	0.340733989879009	3.15656936624468	0.0015963691255676	0.0455887725658324
Zmynd8	1064.2073239968	-0.463606983094775	0.146945724458724	-3.15495387703504	0.0016052348000575	0.0457270645048962
Donson	47.6958762140943	-0.97021747742338	0.307840415133917	-3.15168973833834	0.00162328658582442	0.0458962072997023
MGC105649	21.499231454626	-1.69512928363039	0.53775885267702	-3.15221083798409	0.00162039225272664	0.0458962072997023
Rrm2	182.037563733015	0.864049839568565	0.274121428330615	3.15206966792265	0.00162117588127657	0.0458962072997023
Aida	417.513402789417	-0.402852645357503	0.127951111222763	-3.14848883692879	0.00164116991479124	0.0462866929318045
Pdia5	128.381273014825	-0.871508674303403	0.276903290981171	-3.14733953220753	0.00164763515217159	0.0463540127217384
Kcnj12	344.301660715369	0.945533059446122	0.300752217436783	3.14389389213687	0.00166715878673914	0.0467874735063633
Agrn	2927.53434088312	-0.51171937323014	0.162843425889013	-3.14240117730578	0.00167568267865825	0.0469108604079549
Ttc38	220.745586051074	0.691426488257657	0.220099488484969	3.14142705654164	0.00168126682807346	0.0469515448842332
Shmt2	1752.37111096157	0.438895739561509	0.139844230558116	3.13846154260268	0.0016983721976247	0.0473129862701037
C1qb	161.752151163403	-1.39052359956378	0.443407226127628	-3.13599670376942	0.00171271128659235	0.047381794069228
Pnisr	2274.71720665795	-0.440021419124927	0.140317980193548	-3.13588763548323	0.00171334835143874	0.047381794069228
Zfp53	200.045288266266	-0.517275498847728	0.164951237971493	-3.13592977663571	0.00171310218030056	0.047381794069228
Aco2	18957.4961628542	0.356029546346354	0.113705945771031	3.13114273780624	0.0017412750551736	0.0475449233163185
Acpl2	640.98756725834	0.376613674385762	0.120239170315893	3.13220453365006	0.00173498961341966	0.0475449233163185
Dse	137.693584783641	-1.4524665209811	0.463741469527275	-3.1320608925954	0.00173583869373289	0.0475449233163185
Ptplad2	62.9753092690675	-1.57636770034621	0.503352408844661	-3.13173767056052	0.00173775069720824	0.0475449233163185
Pycard	38.7993289362436	-1.36822889670457	0.436796200360345	-3.13241941110251	0.00173372015877422	0.0475449233163185
Snhg11	172.903739471823	-0.821840931976923	0.262516543298408	-3.13062529946054	0.00174434568211374	0.0475449233163185

Acss3	290.240339901258	0.790014969177487	0.253502222197516	3.11640254010061	0.00183072183056874	0.0486877286189772
Apmap	6584.28868402479	0.621096271232639	0.199279986433921	3.11670169366751	0.00182886528567839	0.0486877286189772
Asrgl1	5012.53025490148	0.435955774670607	0.139780799096522	3.11885307201292	0.00181556472179941	0.0486877286189772
Clec4a3	34.3555880248486	-2.18675235558459	0.701799951538038	-3.11591978710199	0.00183372144472554	0.0486877286189772
Dcn	1024.84946350041	-1.36049263656911	0.436768836683481	-3.11490317601353	0.001840052991642	0.0486877286189772
Hrg	68.8179227908055	1.03594425860558	0.33209872455288	3.11938644148759	0.0018122810274828	0.0486877286189772
Kdm2a	1560.38712482438	-0.31105557558361	0.099914153607051	-3.11322835007891	0.0018505277814005	0.0486877286189772
Mtmr3	1884.42569484326	-0.266600786997269	0.0855340731061321	-3.11689572723219	0.00182766203990296	0.0486877286189772
Plekhs1	23.377712625079	-2.02280472344923	0.649483442947069	-3.11448235580977	0.00184267977193111	0.0486877286189772
Pnn	1619.83980558557	-0.530984519545748	0.170445816196561	-3.11526871937652	0.00183777404490812	0.0486877286189772
Rasl10b	54.3729325773186	0.972268624546236	0.311982405246606	3.11642133721518	0.00183060512484107	0.0486877286189772
Slc7a15	49.6105280825176	-1.93291569035312	0.619010784356163	-3.12258806987274	0.00179268461554444	0.0486877286189772
Sult1c2	11871.1489024158	-0.7035327738891	0.225818696053201	-3.11547620363265	0.0018364816573361	0.0486877286189772
Tmem229a	104.933404248559	-1.40603464341904	0.451154725874089	-3.1165242494023	0.00182996629465674	0.0486877286189772
Tmsb4x	6650.09458090932	-0.60691389425689	0.19491116211431	-3.11379752536159	0.00184696188381738	0.0486877286189772
Npr3	34.6793044792736	1.65615620710105	0.532354743562096	3.11100112684141	0.00186454227811408	0.0489431582749299
Akap8	1764.15856229715	-0.29422904088281	0.0948426477180522	-3.10228623896596	0.00192032178993512	0.0494614286985941
Apcs	57.7989121394827	1.42648925036555	0.459379781824119	3.10525039805018	0.00190118004444878	0.0494614286985941
Fnip2	635.135963095759	0.81722586765243	0.263320287682148	3.10354312174722	0.00191218366818666	0.0494614286985941
Ghdc	231.043903277202	-0.647600241868131	0.20882818791601	-3.10111507613424	0.00192793350154906	0.0494614286985941
Hmgcr	726.008342420934	-0.331449345207383	0.106680917946455	-3.10692250861343	0.00189045946674123	0.0494614286985941
Igsf10	120.889190965543	-1.19158149520584	0.383704197853226	-3.10546900938948	0.00189977527293403	0.0494614286985941
Kcnq1	514.274653750563	0.617196666671493	0.19904273703887	3.1008248572816	0.00192982399260445	0.0494614286985941
Nat8l	24.1911841830973	2.63328587207247	0.849202837449974	3.10089151371635	0.00192938964081875	0.0494614286985941
Npy	47.0501214753316	-1.43931663860259	0.4641668036733	-3.10086078369284	0.00192958987500706	0.0494614286985941
Sgca	32.7518732774662	1.36730782949507	0.440964527348765	3.10072068090332	0.00193050301455525	0.0494614286985941
Vav1	28.0691174542065	-1.75240473715334	0.565206638782687	-3.10046736345415	0.00193215505386027	0.0494614286985941
Hs3st6	51.6160503370863	1.59790295434325	0.515658312482027	3.09876310662391	0.00194330335145776	0.0496350244779077
Rac2	114.037585852197	-1.21747614052841	0.393063425990063	-3.09740377767732	0.00195223762854537	0.0497514190270106
RT1-CE15	184.438540876495	-0.867230388222452	0.280136652450187	-3.09574052747939	0.0019632207425946	0.0497749360921475
Sardh	1761.14645639472	0.555401330277652	0.179420121816902	3.09553535385756	0.00196457951016273	0.0497749360921475
Scnn1b	1121.64989744165	0.52335461271603	0.169081727038332	3.09527600577075	0.00196629828482969	0.0497749360921475
Srek1	509.7797149169	-0.489649858560162	0.158470742246273	-3.08984391452661	0.00200261706943236	0.0505816569137072
Fzd5	96.900273278601	1.12666941966161	0.365003517625879	3.08673578542446	0.00202367382070871	0.0509026115795173
Hspb8	85.3261834773819	0.855082168322865	0.277026264341304	3.08664656889492	0.00202428122769152	0.0509026115795173
Klrk1	15.2841142677471	-1.91591437729132	0.620873899297421	-3.085834948867	0.00202981461496671	0.0509290792797167
Git2	748.002406509057	-0.444332640451935	0.144051739251548	-3.08453506191984	0.00203870578420977	0.0509615972755767
Was	31.3469834602317	-1.48640592918375	0.481973923393517	-3.08399657541254	0.00204239946465027	0.0509615972755767
Zfp692	628.773313663676	-0.455267628488158	0.147637682195426	-3.08368176550975	0.00204456170663936	0.0509615972755767
Sec31a	1650.22594694019	0.397562470844576	0.129040356720298	3.08091577665361	0.00206365012883313	0.0513248301188564
Gigyf1	606.600018755738	-0.401322714195051	0.13037684049748	-3.07817487111762	0.00208272659112501	0.0516861799884865
Arl13b	256.479982611195	-0.477121766280213	0.155070543211617	-3.07680463612685	0.00209232382816235	0.0518112257753666
Tmem63c	11.336498028589	-2.4271741747914	0.790070921698824	-3.07209657782677	0.00212560940042622	0.0525210357505314
Oas2	19.4657107235948	-1.57010684434967	0.51122955040729	-3.07123647899225	0.00213174242837036	0.0525583176591269
Flrt3	20.5206237192176	-1.55749848311296	0.508049242768372	-3.06564472889698	0.00217201228981615	0.0534042436976845
Nkd1	39.9900652224042	-1.75528918283501	0.572897033619633	-3.06388247770263	0.00218484720389084	0.0534042436976845
RT1-N2	124.926225990666	-2.95294582793268	0.96362059281245	-3.06442789823963	0.002180867360904	0.0534042436976845
Serinc2	418.909011985351	-0.76671935855641	0.250170841925752	-3.06478306046539	0.00217827937487434	0.0534042436976845
Fhod1	277.963594826055	-0.671107973414061	0.219277015983686	-3.06054864165058	0.0022093187081073	0.0538865159578275
RGD1311723	171.075712463713	-0.879962433591322	0.287782595029612	-3.05773333338931	0.00223017940085005	0.0542593745959526
Tanc1	1509.64708144374	-0.408015067882738	0.133460388870477	-3.0571997529448	0.00223415337945678	0.0542593745959526
Slc34a1	39285.9722300247	0.697260129863451	0.228275743921131	3.05446438542482	0.00225462782422307	0.0546398717486554
Gpr171	48.1872141441771	-1.19356730784921	0.390851562037328	-3.05376113025544	0.00225991945380642	0.0546515840680079
Echs1	4263.80075593967	0.368817685587413	0.120822065342784	3.05256895370097	0.00226891596364325	0.0547526514708476
Scube3	108.606543305339	1.42662205006687	0.467747380392772	3.0499840509399	0.0022885351892505	0.0551090910191128
L1cam	996.065395468627	-0.602583221071361	0.197641666268958	-3.0488673387895	0.00229705892413365	0.0551974032382729
Luc7l	571.39240826533	-0.46631469069768	0.152999215438808	-3.04782406472001	0.00230504837840162	0.0552725313690145
Ogt	2875.98041853971	-0.438234221776853	0.143827250745562	-3.04694847120532	0.00231177336100007	0.0553170863602669
Magi3	501.511472343959	-0.481159017901273	0.157949810680409	-3.04627790200291	0.00231693580316696	0.0553241435688986
LOC687105	934.316233487767	0.27837319067323	0.0914124400593489	3.04524406626164	0.00232491557815911	0.0553983028539968
Naglt1	3637.52389781943	0.849054164071594	0.279251535992235	3.04046372047602	0.00236214147854339	0.0561675733161594
Entpd3	29.1564639111223	1.54961216783008	0.509808102206352	3.03959894149124	0.00236893376499417	0.0562114847033899
Parp9	372.676227097525	-0.459487222982624	0.15127729003413	-3.03738401764705	0.00238641220976737	0.0565082524504499
Ak2	1436.44876054014	0.374763457975475	0.123453902782727	3.03565500586111	0.00240013817897274	0.0566562989926253
Tsku	1617.88210583976	1.2663989537304	0.417217933367129	3.03534161034262	0.0024026338302345	0.0566562989926253
Chst9	293.88344517954	-0.476826910647005	0.157152716019836	-3.03416270951893	0.00241204301734971	0.0567604160149002
Hpd	7345.85871731897	0.657300177235244	0.216689493825113	3.03337354124672	0.00241836045639579	0.0567914978251954
Fnbp4	1186.6864410331	-0.458084543282523	0.151053732295873	-3.03259334489835	0.00242462095724025	0.056821117113387
Lcat	84.8381249723898	-0.928798938243503	0.306587113512671	-3.02947807428024	0.00244976684877787	0.0572922839572208
Cb707485	51.7936816548331	1.00218579552235	0.331165532819197	3.0262382289328	0.00247617124716665	0.0575545243257589
Gldn	27.1457706109607	-2.96892590688579	0.980805736088778	-3.02702747103128	0.00246971512553538	0.0575545243257589
Wbp2	1259.36908206912	0.381843730449771	0.126152502977038	3.0268422856364	0.00247122858601606	0.0575545243257589
Ftl	57376.7628943235	0.550740343084205	0.182036516053564	3.0254388241651	0.00248272623905999	0.0575891151697058
Epsti1	19.8328336533553	-1.90874184237077	0.63112838157854	-3.02433212969562	0.00249182716249674	0.0576825000589368
Agap2	28.9910042163299	-1.42088465077622	0.470506522171808	-3.0199042602376	0.00252854600017795	0.0580711751412228
Dpyd	1027.34064500094	0.819671787359075	0.271442341770195	3.01969022965848	0.00253033336051684	0.0580711751412228
Fam108c1	1634.16751616609	0.501037717969486	0.165922954937198	3.01970103027112	0.00253024313737673	0.0580711751412228
Slc7a8	222.960582617458	0.804051041935275	0.266309786919008	3.01923204264291	0.00253416354654641	0.0580711751412228
Zfp958	1263.96245537227	-0.411780477280971	0.136358529795238	-3.01983658740908	0.00252911100823293	0.0580711751412228
Gsto1	3949.07954818583	-0.348012558954547	0.115311993352171	-3.01800835140965	0.00254441892584333	0.0581453299541617
Slc25a23	1021.53940780031	0.607734017808417	0.201394744386276	3.01762600439453	0.00254763103265639	0.0581453299541617
Tlr8	13.2182695603078	-2.65172858202985	0.8799246617531	-3.01358593217144	0.002581799127924	0.0588070719198081
Slc7a6	209.136308574284	-0.822030844858625	0.272902776012375	-3.0121747270953	0.00259383251038812	0.0589630006261428
S1pr3	276.416914338906	-2.46693163174041	0.819194897606796	-3.01140990861555	0.00260037554381984	0.0589937493633857
Alox5	18.2705210773354	-2.24838289634485	0.747311883980334	-3.00862724726055	0.00262430878647906	0.0591986470268915
Hmha1	192.343026446009	-1.06248105247655	0.353154627906575	-3.0085434778944	0.00262503238620036	0.0591986470268915
Krt16	11.9673497430058	-3.234870874549	1.07497944140785	-3.00923975840174	0.00261902345418141	0.0591986470268915
Eps8l1	283.268378688943	-0.829146369260872	0.275679159277194	-3.00764980361526	0.0026327633039628	0.059255421213547
Ubc	35218.8154010161	0.362229699087183	0.120501000694221	3.00603063045396	0.00264682333907982	0.0594541384821763
Gldc	259.867097130218	0.991190649590645	0.330445486274081	2.99955874951344	0.00270370976348115	0.0602569054298239
Spink1	568.537008114863	2.17602992528548	0.725131578397959	3.00087596528758	0.00269204196645695	0.0602569054298239
Tcf7l2	275.405002332973	-0.52578775649023	0.175248587545115	-3.00023962449839	0.00269767286762115	0.0602569054298239
Tspyl2	368.910414955774	-0.531909398961478	0.177329599975913	-2.99955224076369	0.00270376753204383	0.0602569054298239
Clec2l	67.1644517354808	-2.14164197477506	0.715174855848995	-2.99457112796937	0.0027483096907523	0.0609027050687152
Exoc4	466.735283243814	0.501633276671753	0.167476888331866	2.99523881574475	0.00274230043105601	0.0609027050687152
Gpr182	80.1975079013821	-1.55037685250586	0.517794706910107	-2.99419216113196	0.00275172578094784	0.0609027050687152
Zfp94	161.478612595427	-0.587160479171767	0.196117601503117	-2.99392035529475	0.00275417828658452	0.0609027050687152
Pogz	693.452654395131	-0.316545765440481	0.105773502016868	-2.99267547547023	0.00276543637942886	0.061032912405026
Pdgfra	1098.11236439235	-0.739690029329597	0.247222647974507	-2.99199946036446	0.00277156753019625	0.0610496832329662
Dot1l	573.654377078501	-0.425507196342343	0.142483181474816	-2.98636787821552	0.0028231280021589	0.0618289353467476
Tmem171	250.42430397441	-0.559882932303264	0.187434111535388	-2.98709198510836	0.0028164496584998	0.0618289353467476
Tmem47	458.677375692555	-0.419504492120803	0.140473838707781	-2.98635315998925	0.00282326389626623	0.0618289353467476
Mlxipl	1881.06070864039	-0.383454481219118	0.128488047379533	-2.98435916055643	0.00284172990954454	0.0621136579843908
Cwh43	203.783105230614	0.656168855710581	0.21992079809542	2.98365985115187	0.00284823214299763	0.0621362889391767
Aars	3161.88843351221	0.343899099480747	0.115448996961678	2.97879677200573	0.00289382642812144	0.0625425569030274

Bcorl1	241.433938206928	-0.52540523439453	0.176339370061944	-2.97951180278101	0.00288708107214111	0.0625425569030274
Gp2	415.316468612988	-0.827549466703296	0.277818681940218	-2.97873944590008	0.00289436784541549	0.0625425569030274
Myo1f	57.7523027606619	-1.27398901946211	0.427646332924872	-2.97907153967322	0.00289123266383038	0.0625425569030274
Plcb1	226.061730929591	0.769235198376726	0.258211460042569	2.97909007698538	0.00289105775090693	0.0625425569030274
Trpc6	22.4296053603806	-1.96169750213066	0.65890292632534	-2.97721777177546	0.00290877316443401	0.0627345650606393
Aqp11	371.912538608052	0.744491349801352	0.250143201501816	2.97626057926642	0.00291786816994588	0.0627355986085147
Nln	449.456778932626	0.390877175505421	0.131340873406087	2.97605128829078	0.00291986025549044	0.0627355986085147
Alg11	654.724243887471	-0.41281224250753	0.138839724914199	-2.97330063685045	0.00294615727564175	0.0631811765942342
Atp6v0e2	1461.44996988367	0.475408964752661	0.160011805405703	2.97108681167169	0.00296747885399817	0.0635185775038479
Adamts12	57.3926013944781	-1.59572237209191	0.537413816389842	-2.96926190474859	0.00298516048204966	0.0637769436822865
Ugt2b7	38.1910149537651	2.70327479579328	0.910820453926388	2.96795574159532	0.00299787493352757	0.0639284174380382
Gsta3	20182.8133511437	0.696614825509096	0.234812696758867	2.9666829567758	0.00301031198033559	0.064073419416656
Cybb	31.5373928272784	-2.29990176338085	0.776563590806145	-2.96163996176197	0.00306005378666461	0.064289410978244
Lamp1	11329.5165136068	0.327112090756812	0.110441761832769	2.96185143489584	0.00305795294546695	0.064289410978244
Pah	7228.41244384031	0.954411045989183	0.321955580753035	2.96441839509932	0.00303255662757034	0.064289410978244
Prkar2a	435.543107036579	0.833363649637677	0.281342252768248	2.962099156589	0.0030554936725305	0.064289410978244
Sema3d	140.392752850568	-1.1258014816787	0.379834934538019	-2.96392295523839	0.00303744324732218	0.064289410978244
Tpcn2	238.728639671053	-0.479847737777365	0.16196504025891	-2.96266241782981	0.0030499085726163	0.064289410978244
Wdfy1	239.321252436252	0.490865764664481	0.165637767617038	2.96348937640469	0.00304172561144964	0.064289410978244
Lnx1	274.98998790491	-0.533061592184031	0.180030966429489	-2.96094390179708	0.00306697796688028	0.0643159992095226
Zcchc24	126.687670983429	-1.46920088377011	0.496335624768213	-2.96009556931607	0.00307543622252247	0.0643746005620449
Akr1c12	647.893034763025	-0.838004619143568	0.283596331654183	-2.95492051767944	0.00312749612229839	0.0653439722905212
LOC498592	965.474609000836	-0.480961756522855	0.162847821714391	-2.95344298412774	0.00314250653137683	0.0655371178635396
Esr1	130.498501644959	0.908287833617998	0.30772397176004	2.95163171209253	0.00316099701064801	0.0658020000421708
Gria3	20.666224719564	-2.00859522028795	0.680667226825345	-2.95092100975114	0.00316827930830519	0.0658330212398478
Bcat1	88.8575213715401	-2.91833136297294	0.990276750770941	-2.94698563881359	0.00320888105882752	0.0663828009102188
Nr1h4	822.83231460606	0.567900631220564	0.192726879139513	2.94666023626868	0.00321225941409646	0.0663828009102188
Scarf2	174.616201755148	-0.981695803680711	0.33308534127031	-2.9472801172719	0.00320582655114431	0.0663828009102188
Ppp1cb	4187.03685254758	-0.262831175685185	0.0893436810668152	-2.94179926937002	0.00326311370111225	0.0673113436508836
Ccl2	82.4538796419133	-1.50708040443108	0.512821804518251	-2.93879938636159	0.00329486266257962	0.0673550521994244
Frzb	668.436989227913	-1.43874282699253	0.489424682784557	-2.93966135669103	0.00328571139736216	0.0673550521994244
Nab2	978.687861569157	-0.741407359562887	0.252214454122713	-2.93959107990758	0.00328645663583938	0.0673550521994244
Slc22a23	255.291512294299	0.721199189238539	0.245376556399579	2.93915278550129	0.00329110793176376	0.0673550521994244
St3gal5	1769.16475672708	0.358878161682386	0.122108660068107	2.93900663132508	0.00329266029114326	0.0673550521994244
Sgce	273.053874006473	-0.705023398734162	0.240095196716498	-2.93643274990898	0.00332010799676211	0.0677492773630128
Fbln5	880.198692776635	-1.71241433198572	0.583336667777006	-2.93555064609847	0.00332956253645658	0.0678204440669633
Sord	8382.79490350616	0.585039322126656	0.199374108193899	2.93437963146992	0.00334215155330751	0.0679550886491828
Tmem144	279.854020568032	0.594164217789283	0.202538289471433	2.93358959108365	0.00335066937008843	0.0680066215364734
Tmem237	187.618564259665	-0.594024081750363	0.202553608038787	-2.93293652164415	0.00335772535490174	0.0680283536253355
Gna12	3772.04466550838	0.322195901055232	0.109919560172684	2.93119714588614	0.00337658422214892	0.0681674178844487
Plagl2	177.773497359651	-0.595727759407299	0.203208008015925	-2.93161556586201	0.00337203879026891	0.0681674178844487
Vac14	618.964788735616	0.320554929642815	0.109516303653452	2.92700647254462	0.00342241758128127	0.068970209625608
Tnfaip8l2	79.3999020962756	-1.11682220362328	0.38217735319357	-2.92226159998974	0.0034749953541757	0.0699058357443558
Defb29	48.9759816496878	-1.35910599390193	0.465565716614542	-2.91925703590238	0.00350866786349775	0.0704585140221121
Nckap5l	130.639311104825	-0.783375898519787	0.268441041059138	-2.9182419179607	0.00352011133964789	0.0705636428332239
Cfh	647.071084566534	-1.72942749845798	0.593327374102201	-2.9147947220114	0.00355922568230761	0.0710969404307704
Dusp8	118.682697431495	0.980950284775145	0.336495557565039	2.9151953501958	0.00355465965082329	0.0710969404307704
Hba1	7132.08885032209	0.713875300445153	0.244971979635148	2.91411001988215	0.0035670417093793	0.0711280632786056
Chd2	472.880689766499	-0.465838752097433	0.160183894826254	-2.90814974003918	0.00363574202784695	0.0719344384334055
Cpxm1	29.5243903416194	-1.42228705864666	0.489009726585209	-2.90850463973099	0.00363161787612266	0.0719344384334055
Ilf2	872.768494876803	-0.357520042588526	0.122962068518834	-2.90756366491806	0.00364256190478477	0.0719344384334055
Irf2bpl	765.698224687099	0.564086637009687	0.194023201092705	2.90731538204115	0.00364545456076382	0.0719344384334055
Ncf1	157.059490863111	-1.04656008523912	0.359823311255301	-2.9085388647779	0.00363122038512879	0.0719344384334055
Tnmd	5.62911164814756	8.11290297150848	2.78859837384244	2.90931209298871	0.00362225062131668	0.0719344384334055
Bhlhe22	12.7345495043414	-2.62587627679838	0.904095856641879	-2.90442242103817	0.00367931373645764	0.0724767416439818
Adra2a	53.7923169620143	-1.69696227977268	0.585792032945732	-2.89686814489312	0.00376908130926928	0.0739345314602769
Il10ra	18.6290749535895	-2.26039315878002	0.780372693217211	-2.89655593849804	0.00377283373631538	0.0739345314602769
Zmat4	14.7026393999163	2.39146326554596	0.825366009270425	2.89745790193114	0.00376200223876413	0.0739345314602769
C6	18.9196507216852	2.2872187948521	0.789859609813667	2.89572826162319	0.00378279807070692	0.074002208040714
Gadd45a	3404.41121291707	0.654306949058519	0.226111422569029	2.89373682065428	0.00380687093841548	0.0743451805601895
Aldh4a1	6854.00321356081	0.365050892210107	0.126318813753387	2.88991703898359	0.00385343505309136	0.0751254593712459
Cdv3	216.110115102959	0.570411674141627	0.197781126316534	2.88405514097805	0.00392590004164356	0.0762765467920011
Lamb3	210.243825206966	-1.00398703595258	0.348059978563796	-2.88452306437341	0.00392007042351208	0.0762765467920011
Bscl2	2370.4478482661	0.342188592819103	0.118686577621973	2.88312798022539	0.00393747432549093	0.0763708757398121
Epb41l3	126.566454664566	1.06019057988194	0.367981172762167	2.88110006260336	0.00396289808185203	0.0767330487194722
Tcp11l2	612.539246116256	-0.509007474819893	0.176724216712187	-2.88023613452401	0.00397377425069631	0.0768127859411808
Pnkd	883.00284751174	0.391633895160012	0.13600747984717	2.87950262441511	0.00398302982843601	0.0768609796774256
Ap2a2	3189.57002065197	0.3551900136041	0.123620276698808	2.87323425484231	0.00406292731689263	0.0776643985189325
Casp3	219.978354087264	-0.696830289009627	0.242386045047841	-2.87487791994002	0.00404183741988735	0.0776643985189325
Nsf	394.174065993385	0.639449445415629	0.222568083074081	2.87305096302955	0.00406528531717047	0.0776643985189325
Pitrm1	988.394966529933	0.272344842424332	0.094765934034711	2.8738686026624	0.00405477618110219	0.0776643985189325
Ppm1d	294.184560779119	-0.743393942446509	0.258771138977755	-2.87278537082304	0.00406870429404206	0.0776643985189325
Psmb8	213.470653301287	-0.62027739895495	0.215708473062091	-2.87553562523436	0.00403342626543606	0.0776643985189325
Zfp219	1026.49823866148	-0.327273842116273	0.113933804810136	-2.8724911158866	0.00407249529449971	0.0776643985189325
Pde8a	351.254282155064	-0.436332492993711	0.152061937182107	-2.86943926323368	0.00411200297162048	0.0782864753357426
Ap1b1	1780.4510062553	0.304637882840595	0.106245941403655	2.86728960010999	0.00414003980727622	0.0786884489122098
Asb13	613.864485449645	0.449562488639695	0.156943354765443	2.86448884256094	0.00417682869013469	0.078991405810434
Nlgn2	90.1400787336196	-1.03174828364981	0.360169737100266	-2.86461681083046	0.00417514133964246	0.078991405810434
Setd4	208.294881158439	-0.560465022933059	0.195628522031723	-2.86494534187695	0.00417081226108376	0.078991405810434
Sgms1	970.694083543159	0.403434736293995	0.14091670434014	2.86293053868202	0.00419742569978945	0.0792490706043303
Lat	40.271069227801	-1.07856610831178	0.377065711075775	-2.86041948824945	0.00423080964552261	0.0797469028706634
Myrf	62.1756330140395	1.26058283676539	0.440812713764665	2.85967894618931	0.00424070089505526	0.0798010039291358
Trpa1	26.8893290705658	-1.70734535099085	0.597180756198012	-2.85900932551941	0.00424966291575008	0.0798374689263064
Rspry1	280.248669338917	0.566041707421012	0.198214590187631	2.85570152472224	0.00429418613066873	0.0805407913550837
Zdhhc7	814.462860570564	0.319012425727217	0.111819977930335	2.85291082713286	0.00433207759871308	0.0811176177709603
Cyp2c23	15108.9426049432	0.982995228239495	0.344657563439815	2.85209243177147	0.00434324692861372	0.0811930009714203
Mlec	695.745059910711	0.458936491564527	0.160950135471045	2.85142034967153	0.00435243891473962	0.081231232684615
Gabarapl1	8682.06181065546	0.346827824163015	0.121664766660688	2.8506841683286	0.00436252781459027	0.081286051050218
Mb	5.85757238560707	4.95426065865705	1.74258982270712	2.84304464200335	0.00446848110434233	0.083123987286342
Cpn1	359.580819876586	0.599463545520366	0.210934555596035	2.84194092251252	0.00448397998039048	0.0832411258816944
Gimap5	35.6007642205143	-1.19377530981902	0.42011354696994	-2.84155395232811	0.00448942549405936	0.0832411258816944
Avpr2	388.793516676926	-0.552012038541868	0.194366638576894	-2.84005548783252	0.00451056872434474	0.0833793867678515
Hoxb5	187.956562982592	-0.580364349464757	0.204354664944327	-2.83998581399092	0.00451155400864233	0.0833793867678515
Bcar1	2265.03154666806	0.386928796652782	0.136294154338338	2.83892437303119	0.00452658838031811	0.0833860673106899
Klf5	131.40106124244	-0.575551992612907	0.202701311920683	-2.83940931195414	0.00451971402760896	0.0833860673106899
F13b	22.81583053796	1.55768253666998	0.548864867724833	2.83800736441179	0.00453961352274466	0.0834279001225832
Nr2f1	35.0210846535656	-1.77058330798542	0.623943227754012	-2.83773143008368	0.00454353951925734	0.0834279001225832
Slc6a20	1326.99997403628	0.52525880611542	0.185449084551273	2.83236127795604	0.00462056102707542	0.0847053171511923
Adap2	145.006728338893	0.8448292270759	0.298448241832314	2.83073950072246	0.00464405275832235	0.0848347034925004
Rell1	165.436187133468	1.09075554004718	0.385381132301746	2.83032937687552	0.00465001058207177	0.0848347034925004
Tnfaip6	21.3231651944393	-1.47390206610984	0.520711330295476	-2.8305550126468	0.00464673194037117	0.0848347034925004
Herc2	1103.98892061656	-0.3403456851692	0.12032625502332	-2.82852387538562	0.00467632128333325	0.0851779931191759
Atp1b3	3702.03598961402	-0.325744582307317	0.115203090284815	-2.82756809302583	0.00469030393087206	0.085295991165267
Gpc5	17.5369634381259	-1.70517158940516	0.603177267738838	-2.82698251510278	0.00469888934890886	0.0853156171560672
Gfpt2	19.8393439249599	-1.77379848652958	0.627979834503111	-2.82461058312978	0.00473381100994835	0.0854521312830392

Ifngr1	810.559856698368	-0.499674445028631	0.176911612210112	-2.82442988781982	0.00473648097028987	0.0854521312830392
Irf1	188.272968836845	-0.622693122334157	0.220353046039331	-2.82588842553604	0.00471496841386382	0.0854521312830392
Sim2	741.032695399284	-0.71722543507716	0.253892072219864	-2.82492253029493	0.00472920487335408	0.0854521312830392
Aldh7a1	8920.94726403354	0.45108013284623	0.159742643637658	2.82379283686709	0.00474590493528832	0.0854864587868257
Fcgr3a	58.0779197005235	-1.08636930652843	0.385135866959813	-2.82074301493656	0.0047912568187988	0.0861668117127644
Klk1c3	34.8135790673624	1.74103594333586	0.617691539648905	2.81861711158527	0.00482310130419868	0.086329715627594
Loxl2	56.5158485056347	-1.81486803549555	0.643709063447149	-2.81939176959337	0.00481147537993215	0.086329715627594
Phf11b	20.6037066818574	-1.38879495882005	0.492698876108441	-2.81875000363179	0.00482110508044671	0.086329715627594
Tsc22d3	2167.33618123634	-0.686651283402463	0.243661384202752	-2.81805541591726	0.00483154701975536	0.086344911048018
Tbc1d2b	270.952269774286	-1.15528330369241	0.410096462526282	-2.81710136336124	0.00484592292674653	0.0864658712486674
Reep1	22.1714329944511	-2.03829009071224	0.723899592810018	-2.81570829843964	0.00486698349945396	0.0867055398977958
Tmsb10	1351.70028161459	-0.552063830818295	0.196148587696001	-2.8145185101914	0.00488503642846769	0.0868909609483001
Tpd52l1	219.183654664167	-0.464277656396275	0.165004950361877	-2.81371956040139	0.00489719304949307	0.086971087813341
Rapgef5	270.537727635648	-1.113119894815	0.395706129235531	-2.81299634394202	0.00490822092145084	0.0870309500674107
Cp	338.048627918885	-1.85078533556975	0.658425870059722	-2.81092438758804	0.00493993935056484	0.0874569324899065
Immt	3195.71712707447	0.326203349272416	0.116071749162458	2.81035955455319	0.00494861816356551	0.0874743297777381
Wdyhv1	77.7414598190207	-0.728256865660449	0.259198717659924	-2.80964686953406	0.00495958843834074	0.0875321152021441
Atp6v1f	1864.23842470934	0.244762338558908	0.0872319957472512	2.80587800912053	0.00501796857583945	0.0883438549372495
LOC686539	467.495362191991	-2.33995292019558	0.834007047064944	-2.80567523791363	0.00502112707104198	0.0883438549372495
Pou6f1	177.254904628797	-0.465558857913587	0.16610952851926	-2.80272216810011	0.00506733015191038	0.089018971416713
Adamts4	32.9260990966051	-1.50031744111538	0.535493111710875	-2.80174928174508	0.0050826356737131	0.0890690507411727
Fzd6	453.063306908131	-0.442130370691032	0.157816619340509	-2.80154506248218	0.00508585376834604	0.0890690507411727
Plekhb2	6673.34496791186	0.303994980906098	0.108553007637122	2.80042891047583	0.00510347469646961	0.089240143692421
Slc43a3	94.358411123836	-1.55465281799177	0.555377655533837	-2.7992714552	0.00512180595378076	0.0894231128581753
Ces1a	73.5951485273189	0.774226564246974	0.276776727030499	2.79729647992282	0.00515322217425854	0.0895736078599911
Inpp5j	564.08692809114	0.341790090380259	0.122159103572297	2.79790928703057	0.00514345557793927	0.0895736078599911
Nr1i2	248.533069542976	0.417136282070889	0.14912405392808	2.79724344318098	0.00515406823336567	0.0895736078599911
Nudt4	10187.5177384697	-0.264407643993988	0.0945545930088918	-2.79634902525701	0.0051683551961683	0.0896847712361052
Col5a1	178.188667195795	-1.32102307557922	0.472753088043459	-2.79431929476424	0.00520090989074883	0.0900733357250658
Slc25a15	405.661243184735	0.768345331946304	0.275001686452047	2.79396589111566	0.00520659700610314	0.0900733357250658
LOC499742	360.99114787676	-0.514782065971702	0.184363154986309	-2.79221770754537	0.00523481221574993	0.0902866094752863
RGD1307100	594.70149754105	-0.397656201208108	0.142410073773544	-2.79233196550722	0.00523296391263121	0.0902866094752863
Sh3tc1	1766.9029623055	0.262310457119721	0.0939873057999118	2.79091367591864	0.00525594880484098	0.0905138092663979
Pde2a	196.855637279997	-1.24100015357681	0.444794048345334	-2.79005566327477	0.0052698980492942	0.0906167340821148
Stard7	480.757576968664	0.366797079436657	0.131501889430415	2.78929132520753	0.00528235252307135	0.0906936839535181
Pla2g2a	34.3273707997517	-1.39328578712416	0.499949690321516	-2.78685198550306	0.00532227826263413	0.0911535946102121
Sypl1	1815.00308970108	0.389995352112706	0.13995004702549	2.78667539169654	0.00532517920298969	0.0911535946102121
Svil	457.94579857147	-0.451147856227261	0.161981913188296	-2.78517426635667	0.00534989619735633	0.0914389777130107
Mier1	1076.36301341906	-0.30668151535089	0.110260284130676	-2.78143229694043	0.00541196176311453	0.0920591709517368
Oasl2	263.4028181603	-0.620441465447787	0.223028908213271	-2.78188809880417	0.00540436706655021	0.0920591709517368
Ogdh	5305.07434771447	0.462687732608566	0.166361914665007	2.78121187496702	0.00541563795042543	0.0920591709517368
Rhoj	115.498278675772	-1.1428388767901	0.410940047172874	-2.78103554192987	0.00541858044753756	0.0920591709517368
RGD1305235	265.975384024418	-0.405216750000629	0.145756779482578	-2.78008852445223	0.00543440818343339	0.0921902737506028
Gpsm3	119.489888442192	-0.782172651300827	0.28148666818115	-2.77872005930122	0.00545735341468497	0.0924415483029946
Cldn4	1118.36463309385	-0.675109452359239	0.243028280919411	-2.77790489981332	0.00547106282527122	0.0925358632024295
Cdh11	138.452498428885	-2.34472654557299	0.844520395820868	-2.7764001404536	0.00549645165978077	0.0928271464562678
Pof1b	28.6434577678731	-1.87652899975015	0.676309863955102	-2.77465862877719	0.00552596778131117	0.0929405951509771
Prosc	785.731179386441	0.494222305924099	0.17808369567035	2.77522489671907	0.00551635469617607	0.0929405951509771
Vdr	269.302522226782	0.946311505632869	0.341067644397233	2.77455666398752	0.00552770036266589	0.0929405951509771
Arhgap22	372.460100409505	-0.479956186497895	0.17331920197952	-2.76920376401577	0.00561934792807873	0.0935654030229421
Cpsf7	2236.00808838762	-0.384513691110319	0.138884190863802	-2.76859222578756	0.00562990491773061	0.0935654030229421
Gpr88	43.4093113776884	-1.98767315283028	0.717948602159312	-2.76854519509075	0.00563071754950664	0.0935654030229421
Hist1h4b	1427.91389548195	0.6263451988607	0.22610486462748	2.77015357406235	0.00560298675640366	0.0935654030229421
Sfxn1	3411.04807985872	0.502704926341522	0.181502784124449	2.76968162646386	0.00561111101855898	0.0935654030229421
Slc11a1	84.6166799489676	-0.775890523809529	0.28011052095138	-2.76994423906057	0.00560658900779059	0.0935654030229421
Sp100	73.7242699164284	-1.15913843661494	0.418519192027198	-2.7696183560911	0.0056122009832325	0.0935654030229421
Stx16	566.473924540446	-0.330539900677464	0.119389572724133	-2.76858265873205	0.00563007021594338	0.0935654030229421
Zeb1	263.076092941323	-0.621243035411543	0.224474528142783	-2.76754356296669	0.0056480496474745	0.0937163975083142
Ppl	824.945526217732	-0.618640224560865	0.223754005154256	-2.76482302131027	0.00569536865066924	0.0943637902091933
Gprasp1	400.735212983233	-0.499522912097942	0.180868977499572	-2.76179430548903	0.005748468165237	0.0949583149764108
Map3k9	118.19541134369	0.73951940214089	0.267856873611618	2.76087520984496	0.00576466983404218	0.0949583149764108
Phf23	561.744102983823	-0.49910202249725	0.180769124545691	-2.7609915340996	0.00576261701559955	0.0949583149764108
Robo1	30.8995190711668	-1.89117515040644	0.68464385048233	-2.76227581548292	0.0057399965866901	0.0949583149764108
Gpr137b	321.052617357581	0.709836712366301	0.257205828066778	2.75980026464256	0.00578367102613609	0.095133436878528
Tnfrsf1b	44.7719217500835	-1.25353608114131	0.454412421940271	-2.75858673886797	0.00580518967456326	0.0953494015044596
Dhtkd1	3750.0389318916	0.332302951948457	0.120581575038069	2.75583522477249	0.00585424806107442	0.0960164263523404
Acap1	146.616051303162	-0.582750272111913	0.211523515081185	-2.75501412638801	0.00586896014509562	0.0961190216270272
Glb1	1898.27864232462	0.474330702966602	0.172270074306906	2.75341323717991	0.00589774004884404	0.0964513861800884
Casp1	77.1898910451946	-1.05688931137321	0.384029790512145	-2.75210240841923	0.0059214000559927	0.0966991853971452
Pdgfrl	30.452038267649	-1.31347281658612	0.477556807931567	-2.75040119787033	0.0059522338581738	0.0970632568608371
Tacc1	615.126365565618	-0.791049434257002	0.287731754858241	-2.74926010390037	0.00597299675117338	0.0972622938020583
Gsto2	89.6647888960979	-1.1293173272561	0.411061959494526	-2.74731655696091	0.00600851104317941	0.0977006244875211
Il15	65.5278126821104	-0.749082631266917	0.272720031760829	-2.74670924035331	0.00601964744364631	0.09774187549212
Rgs19	52.918159109239	-1.10991271132198	0.404160949917787	-2.7462146245147	0.00602873097941381	0.0977497236975997
Klhl6	18.1363493367585	-2.05294030156563	0.747805378693896	-2.74528688888446	0.00604580199591306	0.0977940822551309
Mll5	1300.73514776968	-0.343401898304908	0.125117551235003	-2.74463410540949	0.00605783977771486	0.0977940822551309
Slc6a12	206.459762250537	-1.12345183199476	0.409391594281439	-2.74419858074184	0.00606588315941116	0.0977940822551309
Txnip	6419.85849276461	0.486896325282947	0.177398971436581	2.74464006944374	0.00605772969911055	0.0977940822551309
Zfp697	50.6404569148612	1.18877159556366	0.433398797937453	2.74290469013996	0.00608983584003381	0.0980411815266633
Bach1	210.444370701731	0.735878135750963	0.26840364789595	2.74168455428831	0.0061125011744638	0.0982668859249725
Cflar	363.6448841634	0.405336074968828	0.148034362400934	2.73812153066882	0.00617912368676355	0.0989691945764245
Jund	2968.90473405038	0.490705226096426	0.179232928629189	2.73780733177459	0.00618502993177274	0.0989691945764245
Ptges	561.291710698719	0.539237700711211	0.196911595534912	2.73847611282803	0.00617246442559504	0.0989691945764245
Sars	2016.7257546966	0.267744087765246	0.0978064482660473	2.73748911765965	0.0061910168347561	0.0989691945764245
Krit1	542.379398124758	-0.415930720721825	0.152136385482932	-2.73393323629663	0.00625827325452359	0.0999038396220718
Nxpe3	53.3431925046078	0.923646183456367	0.337934743222357	2.73320870961356	0.0062720574440318	0.099983457095183
Ncf4	40.8653995180756	-1.38869539137466	0.508454956511682	-2.73120632140549	0.00631029532316768	0.100452124150033
Mapt	3153.76715046964	0.3040372384039	0.111397178257703	2.72930825680835	0.00634673459890715	0.100890888742907
Daxx	477.307822900619	-0.43519933926557	0.159534318694579	-2.72793554908231	0.00637320589340492	0.101170192995028
Zfp612	1084.06300276232	-0.55984441478581	0.205292325900274	-2.7270596323107	0.00639014895452265	0.101297675058723
Abcc6	1497.28436233106	-0.382322508998647	0.140269278104182	-2.72563254167947	0.00641784032494036	0.101370770666682
Colec12	272.931112970577	-0.925425784220357	0.339542862567529	-2.72550504293491	0.00642031956614834	0.101370770666682
Reck	203.327286227451	-0.956746492210426	0.351042495632168	-2.72544351215225	0.00642151635403931	0.101370770666682
Id3	756.625487714419	-0.727228672337858	0.266962374861347	-2.72408676584316	0.00644795644092896	0.101506195162879
Rn28s	323415.233552023	0.557121757758027	0.204513312520306	2.72413443844987	0.00644702574595527	0.101506195162879
Btbd9	169.409164107025	0.620111100364881	0.227804629254366	2.7221180815973	0.00648649606780249	0.101971665707667
Bckdk	2911.58649075412	0.301075998907559	0.110687057572556	2.72006506912701	0.00652690713073754	0.102465229900501
Hectd2	62.5343345317306	-0.784294099690279	0.288470327273135	-2.71880337608408	0.00655185423138364	0.102649563800589
Lilra5	19.5620289803811	-2.34486396593082	0.862539542877365	-2.71855822181617	0.00655671153609251	0.102649563800589
Ddx39a	484.341740381453	-0.374140239256069	0.137710973178383	-2.71685131998472	0.00659062069063502	0.102775497525064
PVR	124.067864394577	-0.816903355435662	0.300687197603047	-2.71678795089274	0.00659188260564593	0.102775497525064
Samd4b	656.934896300806	0.293794864055899	0.108121295820321	2.71727102257576	0.00658226832896313	0.102775497525064
Banp	295.794360768759	-0.494769601958067	0.182209057541095	-2.71539520940926	0.00661967220428109	0.102926394355484
Pink1	1937.08866269054	0.340296437981041	0.125305967489305	2.71572411752924	0.00661309996259675	0.102926394355484
Mgll	2806.79108681517	0.658460281875678	0.242564419358753	2.71457901210901	0.00663600683492833	0.103039417603546

Sulf1	28.7206812734435	2.10852567127231	0.777245362346482	2.7128185942554	0.0066713616766645	0.103447062506096
LOC500959	525.752890843925	0.309258075927747	0.114128448450243	2.70973696854011	0.00673365848056723	0.104128928285887
Snai1	38.5281954229955	-1.0418099072669	0.384406772526558	-2.71017573498896	0.00672475676295424	0.104128928285887
Lztr1	1055.6948584146	0.261747168171923	0.0966460767641654	2.70830619240381	0.00676275977207721	0.104436858110638
Mapk8ip3	567.603015775807	-0.341414853740489	0.126124795275666	-2.7069606178093	0.00679023119245797	0.104718816463334
Prkaa2	155.267292300112	0.763527308922681	0.282246309256077	2.70518084340989	0.0068267213283919	0.104996636831532
Tnfsf15	16.7918735037809	-1.60630794815434	0.593775067125566	-2.7052465438308	0.00682537116796999	0.104996636831532
Zbtb16	56.0015588390603	-2.18981611062786	0.80980926621388	-2.70411342767903	0.00684869064289897	0.105192186279986
Agbl4	186.340673169934	-0.543927039813234	0.201254624003739	-2.70268095705036	0.00687827326038397	0.105205332813922
Cxcl16	931.877099857106	-0.436282423998506	0.16144973894868	-2.7022801451366	0.00688657114319535	0.105205332813922
Eif2b2	803.334493296952	0.259865781339505	0.0961552974186782	2.70256333572554	0.00688070740588272	0.105205332813922
Lrrc8e	690.469706070868	-0.283386606369336	0.104830827861639	-2.70327547869187	0.00686598160958043	0.105205332813922
Naaa	56.3319550923469	-1.21908374972223	0.451469211398866	-2.70025888574977	0.00692855382841821	0.105462733849519
Rnf152	350.048304678886	0.683880014900211	0.253302497297414	2.69985500418196	0.00693697018162049	0.105462733849519
Serpinc1	1348.62317421878	0.696587324952653	0.258025499104335	2.69968405204394	0.00694053536155554	0.105462733849519
Slamf9	30.8329512833949	-1.31338116333012	0.486407288432914	-2.70016752331471	0.00693045689651878	0.105462733849519
Grhl2	296.522149189197	-0.507085011854407	0.188023630246333	-2.69692171771212	0.00699837201912434	0.106199594618648
Abcg1	257.160316927052	-1.05217855135817	0.390760575179189	-2.69264254940684	0.0070888224521937	0.107143026584619
Cyba	793.14273409753	0.523346914638258	0.194343055823775	2.69290257076546	0.00708329647327821	0.107143026584619
RGD1309730	284.359434236372	-0.361432466729938	0.13420571257408	-2.69312281718582	0.00707861881794553	0.107143026584619
Zfp386	676.891013886968	-0.327615216299622	0.121705047976984	-2.69187861757039	0.00710507994405999	0.107246133657617
Sez6l	18.2422719852803	-2.69551220274869	1.00165544791216	-2.69105729756394	0.00712259606668218	0.107367940177599
Fgd6	38.1763268290144	-1.32738473310871	0.493359428096616	-2.69050241571296	0.00713445185031671	0.107404211563841
Meis1	50.7661847791448	-1.33421726883181	0.496254883440532	-2.68857257299251	0.00717582356530522	0.107884141062512
Hcls1	101.60144523565	-1.34777912898743	0.501407983563878	-2.68798896939728	0.00718837712115895	0.107930111438696
Fgd2	57.24052278586	-0.923773587928121	0.343792545967162	-2.687008775392	0.00720950586368498	0.10810454306945
Gpkow	566.866218579888	-0.301033122404503	0.112099180182634	-2.68541769809604	0.00724392129803673	0.108477482837267
Klf9	858.649526149105	0.650196327196196	0.242629011288987	2.67979630194252	0.00736669735067091	0.109881735023262
Lrch1	118.44642104534	-0.776009765546646	0.289520479840749	-2.68032771282188	0.00735501150842617	0.109881735023262
Thoc2	849.035034519782	-0.345841815578715	0.129036435282244	-2.68018730385916	0.00735809751481279	0.109881735023262
Lime1	192.564891396167	-0.533350223765053	0.199103701677193	-2.6787559411114	0.00738962334850585	0.110079238504741
Nhlrc2	542.336681698241	0.324161836226586	0.121051981328508	2.67787303164318	0.0074091298393065	0.110225353080573
Man2a1	2559.23593550214	0.409043401636292	0.152826688788448	2.67651811917821	0.00743915432307207	0.110239149981796
Mbd6	1177.40861951835	-0.393116071098983	0.146855864638409	-2.67688370544085	0.0074310423018844	0.110239149981796
Slc22a18	2706.72276069402	0.544165097614564	0.20328453142406	2.67686426410583	0.00743147348731538	0.110239149981796
C5ar1	24.9603634285619	-2.43344678436417	0.909942363820864	-2.67428672531091	0.00748883929870153	0.110543048661093
LOC688553	1504.85497413957	0.529691102900625	0.198021676056327	2.67491475402903	0.00747482539322394	0.110543048661093
Macf1	3475.4220554205	-0.350048195533652	0.130889665664377	-2.67437611485107	0.00748684321312284	0.110543048661093
Ndufs1	5522.91977903463	0.321016369533182	0.120226327394705	2.67010043881055	0.0075828560718829	0.111785657734139
Foxp4	564.306851367464	0.303292166570256	0.113620368093208	2.66934680515603	0.0075998933379093	0.111866585834441
Ppfia4	2186.89011280633	-0.576585251906633	0.216031461746486	-2.66898741158017	0.00760803016452777	0.111866585834441
Naga	990.810504810273	0.271734486933018	0.101828798904934	2.66854259163662	0.0076181118953853	0.111870103104586
Adhfe1	3455.70622649572	0.337061859624376	0.126406476007092	2.66649202059447	0.00766474259061051	0.112322092242075
Copg1	1713.51166549339	0.301534463492269	0.11309010683961	2.6663204405662	0.00766865595458827	0.112322092242075
Fam43a	2956.93600448638	-0.535736674078065	0.201004284624659	-2.66529977248227	0.00769197218789696	0.112516689616235
Siglec5	13.9723754525748	-3.22893541858054	1.21186224885807	-2.6644409639983	0.00771164008543438	0.112516689616235
Upf3b	478.205694485404	-0.411506021836777	0.154437583647267	-2.66454584511404	0.00770923574990954	0.112516689616235
Sidt1	13.2669865220853	-2.2876002366787	0.858789513628752	-2.66374961544722	0.00772750565022946	0.11260362720578
LOC361346	25.779839647673	-1.17932601631112	0.442953963573609	-2.66241215406834	0.00775828173145765	0.112907336952302
Clca2	14.4487017316808	-2.886967305357	1.08486333089452	-2.66113456243061	0.00778778267972376	0.113191736493274
Map4k3	1637.5475378995	-0.247440085369501	0.0930022950680883	-2.66058042103527	0.00780060961540409	0.113233370228203
Gng2	42.8433402435053	-1.49940709012775	0.564303755676438	-2.65709216896916	0.00788178909913129	0.114265835332559
Csad	4566.23005279998	0.643164945265602	0.242105165044155	2.65655193745371	0.007894428952522	0.114273273686839
Pde8b	14.2499745540068	-2.16949953148471	0.816764689241809	-2.6562112197806	0.00790241009307193	0.114273273686839
D2hgdh	884.86091685319	0.419821493482724	0.158211951739506	2.65353842656561	0.00796527006827212	0.114309671207047
Lrrc71	568.250576014069	-0.333961004917769	0.12583127967903	-2.65403805611479	0.00795348563298339	0.114309671207047
Rarb	251.387317587628	-0.655972952827	0.247166090120213	-2.65397633028041	0.00795494067402916	0.114309671207047
RGD1304982	2958.5600175163	0.585611363731677	0.220662250367118	2.65388104561332	0.00795718725348133	0.114309671207047
Srgn	334.352913189409	-0.909903186503112	0.342877600789106	-2.65372594887809	0.0079608452703922	0.114309671207047
Usp15	766.116071057701	0.289947799132717	0.109251742013413	2.65394211377537	0.00795574735017523	0.114309671207047
Slc15a3	65.7379216975703	-1.23851073778717	0.466834089722794	-2.65299978097701	0.00797799226283344	0.114327640044443
Spn	65.0619596326619	-1.24897998833479	0.470845183384192	-2.65263409802298	0.00798663964413932	0.114327640044443
Nfic	242.676308393085	0.747152671381383	0.281856332263633	2.65082804910175	0.00802947087140834	0.114492194160396
Plek	42.5956718257037	-1.46284205278476	0.551921843506035	-2.65045145430771	0.0080384278497269	0.114492194160396
Sema6b	116.154398000389	-1.25953659376801	0.475214227739351	-2.65046061385781	0.00803820989172452	0.114492194160396
Vmac	166.98132302071	0.519927747526206	0.196077509730851	2.6516439760985	0.00801009540779206	0.114492194160396
Abtb2	148.854744090875	0.641025796891118	0.24203476472735	2.64848645860121	0.00808530883113521	0.114968629829242
Mrpl4	1381.54912233014	0.216472953844193	0.0817433597736705	2.64820230589444	0.00809210838143529	0.114968629829242
Ssfa2	2007.05142964747	0.372593535163264	0.140720815873641	2.64774996399844	0.00810294312745476	0.114978840932148
Phf20l1	838.41318613845	-0.271705891687463	0.102696935716602	-2.64570592872654	0.00815206510238336	0.11553163585248
Pir	160.056940074394	0.960472065412099	0.363263750612981	2.64400745681718	0.00819308511885947	0.115968375418377
Lypd6b	205.274636326025	-0.532323385438561	0.201477040408464	-2.64210445199788	0.00823926418406269	0.116476961089623
Adcy6	1367.58308076274	-0.328717021102158	0.124479642021858	-2.6407291647291	0.0082727822860821	0.116660074670887
Dapk2	480.364125130746	-0.564662336493038	0.213893860508061	-2.63991839294405	0.00829259929484627	0.116660074670887
Hmcn1	36.3820041296188	-1.92728203307743	0.730061253901979	-2.63989086227578	0.00829327294862543	0.116660074670887
Vcan	55.0688838237566	-1.26102784368988	0.477635127642113	-2.64014887245638	0.00828696156291618	0.116660074670887
Pcca	2217.64061374292	0.304520295754556	0.115433892322298	2.6380492732959	0.0083384465120063	0.117150535297236
Eif4ebp2	486.298475981871	0.572048040909565	0.21695470192629	2.63671649349143	0.0083712763228544	0.117466576155016
Aass	5416.36868571992	0.735116920594583	0.279048465907412	2.63437004824278	0.00842935639698655	0.1181357149299
Sult1c2a	69882.1859110574	-0.555099895356261	0.210749198399063	-2.6339359749552	0.00844014015414207	0.118141173635442
Dcxr	3870.53619105076	0.370750099302723	0.140788290261365	2.63338732656279	0.00845378797223009	0.118186659400206
Zfp512b	1085.50783409984	-0.241373300755397	0.0917657537984086	-2.63032003513692	0.00853045212665999	0.119111939645722
Dtx3l	177.372471195657	-0.534158232837383	0.203292274986904	-2.62753827154423	0.00860051665830802	0.119801580008176
Pip4k2c	2586.04024850433	0.325638686593588	0.123933752570217	2.62752220311485	0.00860092286527115	0.119801580008176
Ctsl1	32883.9506456811	0.476201873338987	0.181391530067335	2.6252707232924	0.00865800970885378	0.120256172501822
Gclm	29823.7075721999	0.415446806947546	0.158236301014601	2.6254835602433	0.00865259871667056	0.120256172501822
Rgs10	81.0602916187276	-0.9049172786654	0.344732475418132	-2.62498413463312	0.00866530048205107	0.120256172501822
Akr1c19	1208.23625602083	-0.568074322696395	0.21649408760606	-2.62397153186965	0.00869110491350378	0.120467193227907
Irx2	500.333356147602	-0.438325954312384	0.167081391251242	-2.62342772603125	0.00870499119092772	0.120512703868556
RT1-T24-4	1066.31375365151	-0.375810957923715	0.14329056796005	-2.6227194383687	0.00872310730052937	0.120616590727271
Tnrc6b	1090.80776277845	-0.337753383189424	0.12883183744212	-2.62166084017211	0.00875024623014656	0.120844834327881
Arc	141.853009850135	-0.944031105998126	0.360338863780697	-2.6198426006379	0.00879703595801148	0.12094763137762
Desi2	246.675969365742	0.514021401827333	0.196158559280291	2.62043830110339	0.00878168191682312	0.12094763137762
Mal	5434.29092304836	-0.440159910016769	0.168042860255609	-2.61933121911425	0.00881023582635113	0.12094763137762
Ostm1	252.831466961413	0.492427427221847	0.187909543510595	2.62055571006207	0.00877865855443342	0.12094763137762
Plxna2	427.052113628161	0.307608029436557	0.117467971567585	2.61865447518667	0.00882773126062042	0.12094763137762
Setd5	828.474665998632	-0.269675587427437	0.102989258263664	-2.61848266483322	0.00883217790281756	0.12094763137762
Xpot	1777.40660097513	0.344262595533159	0.131470947351573	2.61854502814641	0.00883056363992536	0.12094763137762
Il28ra	28.5336686611301	-1.05261810687095	0.402194098065019	-2.6171893419997	0.00886571480567579	0.121115041443883
Slc41a1	826.559285055322	0.266386733565906	0.101776367732275	2.61737316335205	0.00886094124522307	0.121115041443883
Arrdc4	12.7870507367382	1.80285943437911	0.689336959896415	2.61535292500495	0.00891353001680714	0.121362842500642
Runx2	22.5755228839428	-1.40263217224078	0.536325552901545	-2.61526262295815	0.00891588716241738	0.121362842500642
Tbc1d10c	23.2795130040862	-1.18002866866626	0.45107097113525	-2.61605987566962	0.00889509577938903	0.121362842500642
Snx19	1217.01465480359	0.261808878448244	0.100254718655466	2.61143696735087	0.00901626071565589	0.122582319729838
Dhx57	494.083712311437	-0.326259664086254	0.124997795033478	-2.61012335456696	0.00905095791633825	0.122874789336812

Slc44a1	565.090821571566	-0.459302652280476	0.17599120036575	-2.60980464549329	0.0090593941108788	0.122874789336812
Opa3	734.654728416176	0.363616452990755	0.139399618691411	2.60844653955399	0.00909542178004683	0.123142941879451
Ppm1h	378.837351414757	0.577504178351774	0.22141502418786	2.60824296124454	0.00910083329040464	0.123142941879451
B4galt4	229.760415794727	-0.827777489075638	0.317777747132287	-2.6048944475997	0.00919025674354527	0.123827588070938
Fn1	1492.5943893027	-1.59988044154779	0.614163930026154	-2.60497297762124	0.00918815061843837	0.123827588070938
LOC365238	238.141563582665	-0.402180543775872	0.154379833362666	-2.60513653250991	0.00918376556347632	0.123827588070938
Ugt2b15	3553.23518322735	1.30790313167025	0.502128636246865	2.60471727214385	0.00919501005911238	0.123827588070938
Dmgdh	5293.7435659641	0.469029371130222	0.180118558968849	2.60400357306512	0.00921417961864368	0.12386515107677
Mmp2	153.013926395438	-1.08622840046923	0.417170092434101	-2.6038021904478	0.0092195950915843	0.12386515107677
Prss22	29.7689250875955	-1.3829331078245	0.531225325371632	-2.60328911626538	0.00923340524623773	0.123904231438888
Vps26b	1052.04442346924	0.267856733588154	0.102917891407872	2.60262554861931	0.00925129353282451	0.123997880063778
Cttnbp2nl	978.334664338051	-0.387990335150544	0.149142066826597	-2.60148154981416	0.0092822057892405	0.124216847315589
Idh3g	5069.10926020254	0.261258437377671	0.100437174096666	2.60121254632496	0.00928948796570916	0.124216847315589
Col5a2	416.579390731609	-1.63159567125383	0.627648420030445	-2.59953760606088	0.00933494496610311	0.124678007620127
Rnf123	1269.83217290284	-0.292195277158392	0.112435631181415	-2.59877828841405	0.00935561774829564	0.124807454609305
Rab40b	225.701538486903	0.458560845693606	0.176525747988527	2.59769949097403	0.00938505875476874	0.125053432364246
C1qtnf2	52.452671842778	-1.08095588205404	0.416413006578686	-2.59587444430552	0.00943505355498039	0.125278993815312
Dzip1l	306.229983808389	-0.579334785872288	0.223160777935435	-2.59604215056062	0.00943044956699817	0.125278993815312
Tuba8	35.7603852304772	0.976021585964394	0.375927073004025	2.59630565621419	0.00942321967557107	0.125278993815312
Crb2	7.50844735296973	2.2962859218146	0.885856650474954	2.59216422948729	0.00953742305630504	0.12649049061606
Agphd1	1202.00464787551	0.67912093984771	0.262092565925497	2.59114918978953	0.00956560128490394	0.126716345226361
Isg15	214.975885352535	-0.571779200539687	0.220904502867186	-2.58835466510819	0.00964356318852141	0.12760039487862
Aspn	20.6596704630842	-1.83777247848774	0.710462248900551	-2.58672783998266	0.0096892089168044	0.127980228072711
Mybpc2	139.173538857343	0.67288011042153	0.260147866673646	2.58652941892333	0.00969478940441707	0.127980228072711
Ccbl1	5516.03340791596	0.407267202321418	0.157549292767793	2.58501447494071	0.00973749093020915	0.128394805003199
Aqp3	5681.49019153534	-0.416322124560417	0.161106355149022	-2.5841446426823	0.00976208446180602	0.128569932784342
Akr1b1	1701.98619554433	-2.64445879989455	1.02361420840856	-2.58345261151265	0.00978169039207792	0.128679042819858
Psca	368.26464076687	-2.91077328099543	1.12692941173587	-2.58292422815711	0.0097966836342778	0.128727290389828
Them6	457.222040726163	-0.525907027513206	0.203744368167554	-2.58121013230027	0.00984546336648329	0.129218864461258
Stat5a	509.174641859214	-0.415619902162895	0.16105578326776	-2.58059595085707	0.00986299433376228	0.129299646594628
Fblim1	41.8843456667036	-1.19359273337555	0.463044416628558	-2.57770678257204	0.00994583538082073	0.130176653071958
Phf12	651.273658201531	-0.306836472856607	0.119045839392665	-2.57746490278024	0.00995279883156177	0.130176653071958
Slc38a3	11543.3259557076	0.552038779667555	0.214260622018975	2.5764826708039	0.00998112084703427	0.13024732439425
Tm6sf1	112.583067658205	-1.03668954933333	0.402318811408709	-2.57678617040896	0.00997236198046534	0.13024732439425
Cyp24a1	387.144751971758	2.66440103501604	1.03449545491459	2.57555605716608	0.0100079049264186	0.130447072699167
Ppdpf	1584.01139314733	0.264280128854133	0.102635217199136	2.57494587205256	0.0100255774721237	0.13052773602309
Tpbg	198.571664194673	-0.581915201245566	0.226126484067508	-2.57340578059773	0.0100703061872029	0.130960068791474
Chst5	20.3864094738519	-2.54966142220903	0.99159969311879	-2.57126080201761	0.0101328986763177	0.131500176916211
Efna1	395.395993041279	-0.54356788453062	0.211407143986677	-2.57118976341157	0.0101349775627838	0.131500176916211
Baz2b	363.193681754591	-0.408390429698705	0.158902033768288	-2.57007679520466	0.0101675972821524	0.131772988265615
Qsox1	448.221121412689	-0.855677671246865	0.333229110613561	-2.56783589426308	0.0102335590130155	0.132476801528399
Abcb7	226.403912565855	0.513091884571251	0.199914006933489	2.56656295595113	0.0102711978237809	0.132661857346697
Irgm	91.7169651855521	-0.733965709967396	0.285940202306308	-2.5668503555899	0.0102626891094395	0.132661857346697
Chn1	84.5631534219683	-0.622056170418797	0.242429647062349	-2.5659244979176	0.0102901223971509	0.132755426976183
Inpp4a	334.783673315328	-0.456565623114254	0.178006763553121	-2.56487795183134	0.0103212102686495	0.133005528246565
Lrrc19	433.280020561425	0.636162543195386	0.248342102598069	2.56163790408503	0.0104179872167478	0.13396198799879
Steap4	276.97383220578	-2.48500450267151	0.970097053199827	-2.56160401113973	0.0104190038176078	0.13396198799879
Igsf6	34.958072866339	-1.30787670952353	0.510928587973805	-2.55980334690254	0.0104731408586422	0.134505897174381
Alkbh5	302.562147703813	0.401826362013987	0.157015560854796	2.55914993282472	0.0104928475739359	0.134606891112139
Pmp22	107.038693099718	-1.35145098942741	0.528822119099781	-2.55558710692359	0.010600882439654	0.135338284838447
Sf3b1	6223.25103627414	-0.352221320451027	0.137796149851814	-2.55610422228638	0.0105851409297404	0.135338284838447
Sfpq	1676.98694945856	-0.39817952289116	0.15576839941402	-2.55622786386109	0.010581380237359	0.135338284838447
Tc2n	186.184374604018	-0.597889922747213	0.233947532649083	-2.55565816821004	0.010598718029648	0.135338284838447
Zmym6	552.499592938634	-0.410649351730901	0.16070446497239	-2.5553076686541	0.0106093974829365	0.135338284838447
Ntrk1	25.2647971757377	1.29054843669931	0.505785599369889	2.5515721252386	0.0107238125508682	0.136644454543911
Tmem119	42.1078713312816	-1.87339180820893	0.734466668022106	-2.5506832233162	0.0107511995804776	0.136840016160928
Dlgap1	38.1538109882799	1.2335260754692	0.483768512999085	2.54982712252613	0.0107776347738992	0.136869940603507
Luc7l2	1643.27647072286	-0.359766699641463	0.141091952424194	-2.54987398969306	0.0107761860876895	0.136869940603507
Fbxl14	427.229551848002	-0.377830745140321	0.148257309049758	-2.54847971787693	0.010819357794215	0.137246451661884
Duox2	8.63252727297815	-2.93224455244677	1.15142991257859	-2.5466114093563	0.0108774484030336	0.137676034018798
Fn3k	233.969056356127	0.625031785378587	0.245400324477097	2.54698842273504	0.0108657037926119	0.137676034018798
RGD1310127	690.175637938288	-0.364789324517807	0.143313405435715	-2.54539569001755	0.0109153970244316	0.138002672502435
Cyfip2	2876.8588682926	-0.520166111166682	0.204413409475647	-2.54467704687765	0.0109378847271285	0.13813333089838
Slc5a3	179.912738314684	-2.39861147916836	0.943622007772619	-2.54191981472558	0.0110245462108676	0.139073243321556
Irf5	38.9307496920521	-1.02164939781281	0.402014842969196	-2.54132257970159	0.0110433978578736	0.139156607597107
Cox8b	54.90191215451	1.7687599515563	0.696356712208347	2.54001996469173	0.0110846141181494	0.139239412876311
Ltbp4	666.207215776595	-0.592335437240508	0.233157304713208	-2.54049701753544	0.0110695037768085	0.139239412876311
Rnf126	507.468080619348	0.294304700577237	0.115870113605346	2.53995349982687	0.0110867208035423	0.139239412876311
Lurap1l	120.462860660765	-1.02932205579218	0.405531270592126	-2.53820637380008	0.0111422258155915	0.139782051456968
Zfx	266.143538192931	-0.465766986017234	0.183751042377911	-2.53477193919432	0.0112520554393878	0.141004258130189
Klhl14	411.711012779544	-0.388403933099065	0.153315640826906	-2.53336144312617	0.0112974394040793	0.14141706637309
Bhlhe40	2347.49662960974	0.670595513220313	0.264836070773078	2.53211547529304	0.011337664650911	0.141764462510731
Scand1	1468.29646339876	0.525080889502529	0.207444465465417	2.53118774860766	0.0113676982302962	0.14185490022543
Sumf2	542.325344041692	0.314645907418926	0.124310886550987	2.5311210960584	0.0113698587106605	0.14185490022543
Efna5	99.8671646922603	-0.805748447784757	0.318550010065993	-2.52942527805237	0.0114249498337071	0.14238594277403
Zc2hc1c	92.7846433294299	0.568387899307342	0.224835338389965	2.52801851958655	0.011470830106942	0.14280115552629
Nrbp2	652.448623993612	-0.672836294009626	0.266279540889504	-2.52680431910774	0.0115105616669396	0.143138997709448
Dok2	30.3829111214558	-1.05254249684023	0.416805211413411	-2.52526232402661	0.0115611954687792	0.143611527538955
Ssh2	184.39231571761	-0.736424727008855	0.291901369709922	-2.52285464689899	0.0116406505654095	0.144440648827996
Actr2	1011.52729581683	0.542721960707818	0.215290347360249	2.52088385458207	0.0117060483372422	0.144971520669233
Clint1	744.64039363222	0.556674878829578	0.220900021797839	2.52003089134609	0.0117344535470434	0.144971520669233
Lrrfip1	932.902776526409	-0.470594415794396	0.18672140731555	-2.52030242573694	0.0117254043369488	0.144971520669233
Tfec	328.157207757302	0.576545838779409	0.228779105906713	2.52009831271262	0.0117322060710008	0.144971520669233
Letm1	1216.87826927255	0.422791950422039	0.167809507242566	2.51947554920651	0.011752980284601	0.145042751264685
Chac1	27.9381721366531	1.58806873297572	0.630540379508452	2.51858371737228	0.0117827869269405	0.14505258332477
Fam189b	602.977427638026	-0.778510836505597	0.309094798601481	-2.51867983553272	0.0117795712628087	0.14505258332477
Hspa1b	439.977263600733	-0.823246255464168	0.326904698984419	-2.51830658299411	0.0117920629062192	0.14505258332477
Tnrc6a	840.867966149002	-0.268097305360789	0.106482961685673	-2.51774839013387	0.0118107658706212	0.145125583659978
Dock9	789.157380141268	-0.399061687444607	0.158583287914622	-2.51641703670221	0.0118554808104397	0.145517705066369
Snapc5	283.349338423652	0.56845627360727	0.225992766361535	2.51537375624614	0.0118906253541883	0.145791637298494
Kank4	60.0889124877938	-1.20385638510182	0.478793413477878	-2.51435452371243	0.0119250489846881	0.146056149525824
Cit	90.2507557410961	-0.958115234288064	0.381245521917588	-2.51311865768006	0.0119669077158981	0.146411058233475
Pstpip2	159.225038706386	0.583781448946527	0.232343618172665	2.51257793752998	0.0119852628127047	0.146477953902367
Npepl1	1335.23312661622	0.225157369256639	0.0896325535195606	2.51200440482266	0.0120047590253187	0.146555601326098
Poldip3	1180.81717562884	0.214905450928778	0.0855640391333918	2.5116328437201	0.012017404578209	0.146555601326098
Adarb1	120.444944495711	-0.537900785617443	0.214319475441924	-2.5098082407504	0.0120796738611788	0.14684232417771
Anxa1	1547.44040594795	-0.618800477726707	0.246489842049171	-2.51045021807944	0.012057732195883	0.14684232417771
Ddx39b	2675.12601420484	-0.299909265856863	0.119477473208443	-2.51017415922109	0.0120671630731323	0.14684232417771
Adcy7	16.902162870068	-1.65598526349124	0.660384620463095	-2.50760725216462	0.0121551687029404	0.14744466112873
Irf8	32.8692049460477	-1.45614859458108	0.580620820485421	-2.50791660099906	0.0121445327156073	0.14744466112873
Dopey2	156.798464413754	-0.542251910664683	0.216296532770811	-2.50698383241888	0.012176628114272	0.147547500156519
Slc7a7	162.637403390276	0.586948615020731	0.234298406020996	2.50513277059228	0.0122405434935492	0.148164022734483
Armcx3	346.228737827824	-0.491640141013224	0.196361972931667	-2.5037441500158	0.0122886861788838	0.148246292392185
Clic4	546.058396863496	0.602946955196053	0.240878634999046	2.50311512765937	0.0123105491754836	0.148246292392185
Csdc2	81.2513463173333	1.02210168534716	0.408129231146861	2.50435795170812	0.0122673853753475	0.148246292392185

Plvap	1533.88418725792	-2.00728891313464	0.80193480932778	-2.50305746774759	0.0123125549901656	0.148246292392185
Prc1	164.52044129741	0.638881645586713	0.255190880437631	2.50354418814294	0.012295632542529	0.148246292392185
RGD1565410	194.029057687239	-0.653138699771443	0.261224386989773	-2.5002975690666	0.0124089027709215	0.149248242216184
Marveld3	111.791484925666	0.642029527091068	0.256938991308599	2.49876254211628	0.0124627788797786	0.149737785145416
Rgs1	77.3052379258399	-1.14609013637999	0.459119360855671	-2.49627925566893	0.0125503755350343	0.150631011965365
Clec14a	460.720546415634	-0.63572147689021	0.255077874218015	-2.49226428924548	0.0126931549062941	0.1520915012366
Dgcr8	376.167450698468	-0.344708825238725	0.13834306948562	-2.4916956557376	0.0127134923306656	0.1520915012366
Foxs1	35.9307981334462	1.00823954367591	0.404674641268506	2.49148189892859	0.0127211448911979	0.1520915012366
Slc38a4	17.2892580494126	-1.40730710383353	0.564875530110423	-2.49135788119274	0.0127255866334688	0.1520915012366
Arrb2	226.233218592367	-0.682415427866226	0.274146726466296	-2.48923427488062	0.0128018576771172	0.152521922807248
Itga6	2110.30996997384	0.449335337088993	0.180494064926981	2.48947430637551	0.012793216520763	0.152521922807248
RGD1304770	1340.51409112816	0.439201904692948	0.176406903351553	2.48970928205504	0.0127847623743075	0.152521922807248
Habp4	438.423429411446	0.409840170707819	0.164696067804213	2.48846360554903	0.012829636769116	0.152692828814421
Cog4	1237.95083738669	0.224369708042911	0.0901835022193096	2.4879240938912	0.0128491154150739	0.152745021188174
Zfp39	84.7241419485968	-0.655101653083772	0.263347068047876	-2.48759805051133	0.0128608996372587	0.152745021188174
Gorab	250.020162508101	-0.457694509262063	0.184168356541764	-2.4851962511718	0.0129480032148309	0.153329138633833
Mapk1	560.76741313584	0.338368196444711	0.136157350076348	2.48512618859707	0.0129505519169875	0.153329138633833
Pea15	1347.17992322088	-0.397895702510894	0.160082077071701	-2.48557308718999	0.0129343024682143	0.153329138633833
RGD1560108	279.423784341987	-0.551448319165762	0.222070786918207	-2.48320964147738	0.0130204434697566	0.153996212775498
Gca	708.891146920177	-0.255935280030053	0.103132956370423	-2.48160519233841	0.0130792099524625	0.154530457712775
Cebpg	308.269141104552	0.361560683846582	0.145727340320347	2.48107652998933	0.0130986246895103	0.154599136262694
Plekhf2	2170.25221383708	0.379763582092179	0.153165327724091	2.47943570346598	0.0131590452464973	0.155042108361703
Smndc1	660.249468072918	0.289944706546999	0.116945410610813	2.47931667461426	0.0131634378470037	0.155042108361703
Cldn15	34.4186303102066	-1.4243288335511	0.574770522820849	-2.47808260340284	0.0132090560549617	0.155159267544362
Col9a2	86.8030853896065	-0.638944140601647	0.257912162570307	-2.47737111051314	0.0132354203066812	0.155159267544362
F13a1	23.2217767969571	-1.8673941110298	0.754088903247886	-2.47635803018301	0.013273040110813	0.155159267544362
Fam105a	44.5367639782468	-1.5570210690661	0.628309094016319	-2.47811321512667	0.0132079227851525	0.155159267544362
Lrp3	211.751779749041	0.564428916524449	0.227839029390155	2.4773144357015	0.0132375223815996	0.155159267544362
Proser1	335.72548957549	-0.453971186608666	0.18327867703287	-2.47694491229468	0.0132512352823633	0.155159267544362
Sash3	27.2611954784183	-1.3688854975126	0.552834927013944	-2.47611977938231	0.0132819010521616	0.155159267544362
Slc11a2	3756.4178282061	0.399091369951483	0.161201206446486	2.47573438654114	0.013296245520694	0.155159267544362
Tle2	849.612823702101	-0.577759977516966	0.233349734253124	-2.47594015637595	0.0132885849859103	0.155159267544362
Aplf	214.648772960204	-0.427456925964072	0.172885243926506	-2.47248935916003	0.0134175707980914	0.156414471478058
Txnrd1	3788.18431448943	0.470972748992378	0.19055842277874	2.47153992001304	0.0134532530025092	0.156669747568155
Fkbp10	71.253942451556	-1.00688828835693	0.407498586572215	-2.4709000755724	0.0134773471671728	0.156787896080811
Rnf145	513.773032410529	-0.293163226494915	0.118663704640991	-2.47053829460206	0.0134909873629275	0.156787896080811
Spata22	181.262207186457	1.07162199652021	0.433873637013455	2.46989423901545	0.013515300323623	0.156910013767415
Bin1	515.580025003109	0.312811176334571	0.126684075690097	2.46922254932648	0.0135406976999278	0.157044459242224
Ccr10	146.757074220689	0.550859981077102	0.223352470474825	2.46632589246059	0.0136507073588473	0.157516690193562
Dyrk3	48.2599576709082	-1.18869844165485	0.481898271054538	-2.46669995111959	0.0136364570538804	0.157516690193562
Gcat	703.882035418897	0.375510109263636	0.152222527770909	2.4668497807944	0.0136307527629864	0.157516690193562
Man2b2	653.788094733721	0.470028703411553	0.190524389915916	2.4670264191319	0.0136240305244762	0.157516690193562
RGD1564927	96.3700494070593	-0.634140075850124	0.257086573913373	-2.46664019126803	0.0136387328093801	0.157516690193562
Cd37	51.7859038179606	-1.41912597130265	0.575786557827646	-2.46467367466304	0.0137138084724588	0.157764318925068
Stat6	404.661500432565	-0.633419382294112	0.256927822816483	-2.46535924116925	0.0136875942739786	0.157764318925068
Zfp292	480.771975916455	-0.35189622537257	0.142761083859312	-2.46493102923873	0.013703962739588	0.157764318925068
C1qtnf4	69.9793457524699	1.14317010071463	0.464061040319609	2.46340459851424	0.0137624515266387	0.158163826139308
Pde7a	85.0418176044509	-0.7312585196322	0.297110820091158	-2.46123153444173	0.0138460978922442	0.158964392568937
Agpat9	564.763513928744	0.369092702817621	0.150029183435155	2.4601393833297	0.0138883066176359	0.159288085788142
Clptm1	4141.07561131838	0.229811750588831	0.0935485190001426	2.45660490454671	0.0140256845277706	0.159919450625523
Crtac1	86.671777801252	-1.35523371821671	0.551691162328877	-2.45650793551923	0.0140294703592871	0.159919450625523
Fgfr2	82.7595256195758	-1.34663102262017	0.548181394476797	-2.45654273601431	0.0140281115865432	0.159919450625523
Gem	135.419363724908	-0.927496679439748	0.377615840575694	-2.45619113336382	0.0140418451279493	0.159919450625523
Kcnh2	22.8448361700441	1.14982709712693	0.467849390873264	2.45768642549843	0.0139835212308514	0.159919450625523
Klk1c2	79.0708426456949	2.64000979789064	1.07389301661956	2.45835456328878	0.0139575296988959	0.159919450625523
Matk	22.0981172015385	-1.13551208509308	0.462304635522429	-2.45619878721288	0.0140415460435663	0.159919450625523
Liph	229.803601193316	-0.813723907995037	0.331534702200219	-2.45441548831777	0.0141113829756769	0.160389978901544
Stk39	981.655512824	-0.357749354054608	0.145745632736522	-2.45461457292065	0.0141035713233084	0.160389978901544
Abcb8	848.717729469554	0.278344440600792	0.113447132345369	2.45351676015417	0.0141466946829323	0.160630701065143
Higd1b	33.2521965658464	-1.43599829510872	0.585377796310965	-2.45311370564162	0.0141625563081707	0.160650314369928
Ctsc	1126.73397701635	0.502510365584927	0.205126585374262	2.44975737624684	0.0142952503587567	0.161477146802222
Cxcr4	55.6692112838166	-0.93270680917896	0.380746450537646	-2.44967959087182	0.0142983386000126	0.161477146802222
Dlst	6880.77652220659	0.221672121184084	0.090496135309939	2.44952030741293	0.0143046643213438	0.161477146802222
Marc1	79.0333057847671	1.04882151944616	0.42800248139796	2.45050336161709	0.0142656630550623	0.161477146802222
Nr4a3	171.064978533059	-1.07682413721345	0.439614344895934	-2.44947452173873	0.0143064830925424	0.161477146802222
Tbc1d19	272.837865733365	0.448144773251991	0.183040796502638	2.44833273136206	0.014351905123377	0.161829120666967
Etv4	20.6384174672333	1.46410799605851	0.598345926897441	2.44692564993338	0.014408055739899	0.162301250287108
Amd1	2593.29874054701	-0.204022914756454	0.0834286805473026	-2.44547694411608	0.0144660697530534	0.162632392495752
Wisp3	99.9733063007078	-0.588909827172559	0.240813240183693	-2.44550435317982	0.014464970236987	0.162632392495752
Cd2	11.4023177873004	-1.99978215042492	0.818153371453144	-2.44426316654181	0.0145148344631145	0.162858448674984
Sh3gl3	18.9929206453224	-2.21530463408044	0.906309058193242	-2.44431478870655	0.0145127575442624	0.162858448674984
Mab21l3	91.7372391377981	0.798288932858269	0.326684672875877	2.44360693702204	0.0145412594510014	0.162994038382724
Zfp846	184.557787171249	-0.470762943027075	0.19275445246414	-2.44229348276485	0.0145942769444333	0.163427144581704
Slc29a1	131.679514087222	0.676571070089298	0.277114881106882	2.44148227401887	0.0146271063948936	0.163633554413741
Hoxd9	1281.06249000103	0.359372404683496	0.147279388155983	2.44007263462342	0.0146843090874415	0.164111953872036
Ipp	407.466023563629	0.546126069708567	0.224090346179432	2.43707986095606	0.0148064089578319	0.165313992352375
Ctsz	250.684617389068	-0.640211359623087	0.262957749205501	-2.43465485066486	0.0149060003777813	0.16610109394143
Cyth3	511.609982312101	-0.67305541936551	0.276521184594811	-2.43401032854587	0.0149325689838452	0.16610109394143
Fbxo31	902.198444014689	0.225572495272061	0.0927047755698637	2.43323489955559	0.0149645891427084	0.16610109394143
Fcgr1a	56.5298601122316	-1.19090002433897	0.489333073926042	-2.43372068596156	0.0149445222637034	0.16610109394143
Gmcl1	341.316672639317	-0.311548184166403	0.127980646639688	-2.43433825618591	0.0149190458829428	0.16610109394143
Oat	34909.6827689073	0.374603421113182	0.153950995727675	2.43326403536759	0.0149633849309236	0.16610109394143
Mcoln2	667.030239222739	-0.547884483728075	0.225521638057139	-2.42940982713713	0.0151234263943161	0.167700355510045
Ntn1	244.360465067716	0.936887593392281	0.38570981861479	2.42899596581946	0.0151407008051238	0.167728270322648
Pgk1	8660.2409016624	0.279148849144025	0.114947259760059	2.42849503091001	0.0151616328769946	0.167796610788628
Dab2	2519.66687701275	0.5220177097261	0.215121842796843	2.42661415939562	0.0152404546473514	0.168448992729852
RGD735065	1374.61409761463	0.305032632340947	0.125715023216159	2.42638170472643	0.0152502211436757	0.168448992729852
Hipk2	261.467455075985	0.369102289626045	0.152179179054221	2.42544539877255	0.015289615510906	0.168621146528013
Tsfm	252.115592756127	0.470460983095437	0.194008184466018	2.42495430999635	0.0153103134978805	0.168621146528013
Zfp347	517.675728496043	-0.401235758358168	0.165443481824891	-2.42521345617499	0.0152993881559357	0.168621146528013
Gpd1l	166.256508068197	0.718419017840223	0.296583658802995	2.42231490682846	0.0154219798919952	0.169522459818585
Ip6k2	669.497598697954	-0.487070213008254	0.201068334563014	-2.42241133625946	0.0154178876271908	0.169522459818585
Apbb1ip	29.5215803185823	-1.26818527487244	0.523957833156101	-2.42039567809002	0.0155036270778719	0.16972375942531
Dnttip1	914.587411825717	0.439558789326277	0.181596766697327	2.42052101103157	0.0154982836194901	0.16972375942531
Egflam	71.2080385090863	-1.00119113017604	0.413472724385541	-2.42142001425585	0.0154600029015835	0.16972375942531
Itk	52.1777822738815	-1.08922200611957	0.450067544639007	-2.42013008734772	0.0155149556609974	0.16972375942531
Pan2	774.05812106975	-0.277148710511547	0.114489092286964	-2.42074336493891	0.0154888077467891	0.16972375942531
Slc12a2	735.72852948625	-0.332925002954662	0.137630547505725	-2.41897608480279	0.015564263447062	0.170099440710872
Rev1	776.106153248868	-0.257172793997158	0.106390401756254	-2.41725559591695	0.015638031879981	0.170741470074797
Cacna1d	152.822816233917	-0.56258496846878	0.232830342858802	-2.41628716240801	0.0156796900169023	0.171032012218917
LOC100294508	2068.04621011927	0.269715493431971	0.111664349308288	2.41541275351301	0.0157173874272574	0.171278835568752
Hepacam2	198.608894669193	0.604324321592986	0.250406466677063	2.4133734628042	0.0158056150218931	0.171746290954911
Isyna1	1522.46419487789	-0.56566903006421	0.234334202015742	-2.41394139309724	0.0157810005068795	0.171746290954911
Slco4a1	446.658011440109	-1.58519054662111	0.656784552234906	-2.41356247071741	0.0157974195341769	0.171746290954911
Slc36a2	18.129376006605	2.78826496229611	1.1560400320198	2.411910388107	0.0158691813567697	0.172272316428886

Ccnb1	71.4262806573809	0.811605999836657	0.336653771454226	2.41080323066278	0.0159174334500461	0.172631248657656
Tead3	414.312338115207	-0.362935217564279	0.150656125208709	-2.4090306123398	0.0159949562823668	0.173306647383585
Col16a1	132.015236259608	-1.04336675705401	0.433263623802627	-2.40815683508505	0.0160332916314487	0.173556564460044
Ddr1	728.511703845199	0.436340127949857	0.181227522526037	2.40769239609932	0.0160537008850964	0.173612144871556
Rpusd1	264.526698030612	0.397329271827101	0.165066739231275	2.40708257567506	0.0160805334429489	0.17373701816783
Abhd4	1433.94536048194	0.213349836063732	0.0887305785115389	2.40446799336474	0.0161960244061995	0.174561643173353
Ggt1	39965.6520160762	0.22702244664705	0.0944153524141946	2.40450775050986	0.0161942628154058	0.174561643173353
Ugt8	367.539877979753	-0.837668774395416	0.348402675601985	-2.40431211657045	0.0162029327422037	0.174561643173353
Hoxb7	1535.37521035721	-0.296786200862958	0.123465371224895	-2.40380114617204	0.0162255967071468	0.174640276679385
Akr1a1	20298.8233868265	0.495812716977871	0.206324300562035	2.40307474993135	0.0162578637840447	0.174822024379803
Aldh6a1	32540.0223201844	0.396763873983524	0.165186772806539	2.40191068111853	0.0163096902029536	0.174895741754981
Nrg4	4.34230861684814	3.50438725120809	1.45870353221822	2.40239855036139	0.0162879517499387	0.174895741754981
Pcdh9	383.498873886511	-2.28267032546137	0.950614218793894	-2.40125834469165	0.0163387968062712	0.174895741754981
Sytl3	50.0093928136387	1.12651176265228	0.46914644429909	2.4011942887797	0.0163416573767191	0.174895741754981
Tmem26	12.9639798263296	2.04091979309535	0.849848340479828	2.40151059416441	0.0163275362779243	0.174895741754981
Aqp7	2699.69710407051	0.360693892301862	0.15035559455877	2.39893895109354	0.0164426557849748	0.175173098600289
Ifrd1	1245.25347709776	0.399323243227627	0.166434065940031	2.39928791604184	0.0164269926866531	0.175173098600289
Pdxk	380.330810611973	0.43744422599281	0.18226524265806	2.40004193675851	0.0163931936356958	0.175173098600289
Polr3g	357.692589503895	0.853322820140443	0.355603701667216	2.39964549339536	0.0164109566232048	0.175173098600289
Ttll10	74.1831804474201	-0.64034092737772	0.26693162780081	-2.39889492546592	0.016444632782554	0.175173098600289
Mapkapk3	400.696958466036	0.409843959608881	0.170975140500349	2.39709678500334	0.0165255580059243	0.175541581584426
Sorl1	3020.32493749589	-0.582339256010349	0.242903664441952	-2.39740827849682	0.0165115142586008	0.175541581584426
Zfp52	115.366249486614	-0.577526087123573	0.240925131694124	-2.39711848682019	0.0165245792352207	0.175541581584426
Gdf11	306.54903681617	-0.407940777374912	0.170256538441621	-2.39603589447339	0.016573467213764	0.175876114925932
Rasl11b	147.007376725691	-1.12205628126824	0.468402337857318	-2.39549675691421	0.0165978610701043	0.175876114925932
Sec3l1	309.444305433011	0.424053139195328	0.177030119893049	2.39537282950222	0.0166034727534335	0.175876114925932
Kif11	1005.00769835298	0.995633048498269	0.415841702093989	2.39425974712184	0.0166539501038699	0.175994372899692
Prox1	25.1946393969695	-1.69575871717926	0.708202690190151	-2.3944539334127	0.0166451342270853	0.175994372899692
Tcf7l1	173.600705473303	-0.903535304073962	0.377400418283334	-2.39410255077044	0.0166610896744738	0.175994372899692
Lyst	220.749326028446	-0.747865005815131	0.312450698909824	-2.39354563271747	0.0166864055052985	0.176098129037347
Sdha	11360.3411462791	0.337064066158479	0.140852116371768	2.39303515517526	0.0167096399505964	0.176179747382633
Fam107a	696.230161541686	-0.374990212107935	0.156763636660327	-2.39207395347978	0.0167534662661763	0.176348014721617
Zfp467	482.607596175384	-0.365176479936629	0.152665455509726	-2.39200465303274	0.0167566299401149	0.176348014721617
Lasp1	6132.27548497338	0.207254274593344	0.0867124249899229	2.39013353181426	0.0168422480431952	0.177085098296908
Kif21b	9.14428381517657	-2.27369652257272	0.951945954559012	-2.38847227795196	0.0169185846759746	0.17772332109716
Ehd4	664.067055863959	-0.693766145744877	0.290598743588566	-2.3873680153522	0.016969494740407	0.177929222527183
Mylk3	13.0597422356545	1.81700229472994	0.761071777341001	2.38742566578687	0.0169668335471971	0.177929222527183
Gxylt1	693.688029588878	-0.372401684304009	0.156014290593031	-2.38697162220499	0.0169878025060334	0.177957016851222
Gsr	3939.67706561503	0.397536424941752	0.166595304781359	2.38624026927699	0.01702162617852	0.178147148384031
Map1a	38.3481043066248	0.872516535297187	0.365716843066303	2.38577071808258	0.0170433731589158	0.178210652552196
Bag3	1046.90464419336	0.350587628552192	0.147149483429772	2.38252707641691	0.0171942674703049	0.179293618410537
Epha4	158.366560682577	-1.17497610880733	0.493159728686145	-2.38254674999042	0.0171933487340001	0.179293618410537
Rab9b	48.049888516142	-1.48915323179205	0.624897832353063	-2.38303472134737	0.0171705747334595	0.179293618410537
Fbp1	13704.1327964181	0.449893200774977	0.188882725410144	2.38186525421036	0.0172251990109869	0.17945152333536
F8	254.202507839404	-0.577473803271287	0.24256474602397	-2.38069963890887	0.0172797949907302	0.179674482412624
Fibin	21.4357169707349	-1.94959456640084	0.819020979617763	-2.38039636946872	0.0172940246137085	0.179674482412624
Sema3a	13.1938705536534	-2.22241878806783	0.933579528014827	-2.38053504964232	0.0172875163642057	0.179674482412624
Cs	1671.92976428194	0.246706956083899	0.103664793951466	2.3798528572719	0.0173195523169671	0.179775371355843
Dmbt1	45.1049063842855	-1.82564077422012	0.767291539204799	-2.3793312983904	0.0173440799815653	0.17986570535627
Pfkfb4	328.601878030241	0.546335023947972	0.229713195936861	2.37833539218242	0.0173909996933944	0.180187878318251
Ercc6	332.624306732069	-0.329351394781642	0.138512536830464	-2.37777317720172	0.0174175362140686	0.180298466857107
Fli1	67.9731141529459	-1.13674229107564	0.478191275765305	-2.37717070278286	0.0174460123816187	0.180412594518712
Twsg1	1043.97152075029	-0.39144532518957	0.164689511029045	-2.37686858588422	0.017460307405471	0.180412594518712
Akr1cl	31.5126456028102	1.27570285338993	0.536902569215715	2.37604162567786	0.0174994885616178	0.180653212526202
Ces2h	226.32756632688	0.494239617464541	0.20805938599356	2.37547378650747	0.0175264372492191	0.180758117929889
Phf2	697.770602817764	-0.238127095274412	0.10025738604305	-2.37515762850791	0.017541457335621	0.180758117929889
Ccr5	133.775893895279	-0.489940847421872	0.206489638849386	-2.37271395384267	0.0176579330075054	0.18179353855372
Aldh1a1	4750.80354770602	0.539258415512833	0.227334564048948	2.37209162526084	0.0176877038490841	0.181935241582525
Mpi	622.711762626828	0.376390631195319	0.15871361865962	2.37150809347075	0.0177156586892943	0.182058025915478
Cdc42bpa	434.197030755533	-0.403293085548732	0.170201051278612	-2.36950995613158	0.0178116755292844	0.182879407466889
Msn	2374.32042447043	0.477490856710008	0.20154522628295	2.36914991992743	0.0178290248106922	0.182892324908238
Mycbp2	1089.95580748998	-0.30965414458473	0.130741283065361	-2.3684496382824	0.0178628121159889	0.183073690270812
Gosr2	2025.16269196565	0.398309060740047	0.168351332130736	2.3659394653957	0.0179843847313778	0.184153618789946
Akap8l	1722.79768653346	-0.295334057410395	0.124881866783172	-2.36490745228188	0.0180345770216475	0.184501352320473
Acacb	958.376831961384	-1.03307415041846	0.43699169475425	-2.36405900345412	0.0180759334591004	0.184592147076492
Dnaja3	1228.1811709284	0.382409939515005	0.16174085706123	2.36433728906381	0.0180623596764759	0.184592147076492
Cd99l2	554.454051183736	0.275866402920233	0.1167409858075	2.36306384610392	0.0181245468194687	0.184756591165992
Slc22a25	2755.01536819707	0.302928769965105	0.128180915873279	2.3632907278068	0.0181134536011271	0.184756591165992
MGC94207	1803.00876983938	0.31908037738111	0.13509813391111	2.36184148621219	0.0181844156636593	0.185034976215892
RGD1309808	49.1362836860333	-0.816920001702197	0.34583424093968	-2.36217211888132	0.0181682048488753	0.185034976215892
Adamts7	25.2602320418484	-1.66287731283668	0.704442307157302	-2.36055855240585	0.0182474374805278	0.185498921650975
Kin	191.230330208925	-0.417540643544369	0.176905304350506	-2.36024942879659	0.0182626511813691	0.185498921650975
Sept2	2463.35532802991	-0.210395665383142	0.0892430121630545	-2.35755898734942	0.0183955324561133	0.186183100530885
Adcy2	41.5490148873992	-0.964570424730031	0.409045973034084	-2.35809783818519	0.0183688509657396	0.186183100530885
Gdi1	3647.16915533276	-0.214327738593074	0.0908808334594037	-2.35833817136826	0.0183569616661354	0.186183100530885
Med25	1251.71737608275	0.251556820981986	0.106702091333042	2.35756223555936	0.0183953715176678	0.186183100530885
Akt3	40.5378282290542	-1.2626123278144	0.535978221234491	-2.3557157320801	0.0184870588991059	0.186883493305384
Apol9a	90.9000250755799	-0.726596367771978	0.308467527311081	-2.35550358932668	0.0184976183326198	0.186883493305384
Epas1	718.389460322109	0.674881222103931	0.286594633359611	2.35482854020197	0.0185312541200742	0.187057046473147
Tifa	46.7997689854402	-0.815475736474837	0.346388160702756	-2.35422519874926	0.0185613622014436	0.187194714091932
Agrp	7.42761530890535	2.09328328090709	0.88984240176215	2.35242024516	0.0186516891188491	0.187772452192063
Kcna5	7.90883673184272	2.09141740089805	0.88901259151774	2.35251718687982	0.0186468280220538	0.187772452192063
Sgta	1631.5196004177	0.264542864546756	0.11250645198829	2.35135727659683	0.0187050639492018	0.188143147651883
LOC100361645	832.094117242901	-0.404988745053562	0.172314601076618	-2.35028687367873	0.0187589470588663	0.188518295553558
S100a1	551.736977183603	-0.315327652818429	0.134424611267665	-2.34575833878033	0.018988414504033	0.190655759057278
Nptx1	18.6145720821673	1.55689679470691	0.663868355726254	2.34518904430036	0.0190174344425557	0.190778605361067
Pak1	646.357029529626	0.344308954753814	0.146843433371685	2.34473511581761	0.0190406013478931	0.190842570476325
C1qtnf3	1517.71440641339	0.330978935272646	0.141278034629173	2.34274872340489	0.0191422702735994	0.191187209077092
Elovl1	3444.74514603323	0.217445768770294	0.092803577915696	2.34307527418635	0.0191255239990317	0.191187209077092
RGD1564171	59.3011366798872	-0.813313953249027	0.347081001466862	-2.34329724131178	0.0191141483272073	0.191187209077092
Zfp46	272.691546488709	-0.547385009497682	0.233556211059012	-2.34369707838502	0.0190936718585716	0.191187209077092
Marf1	1031.47115847576	-0.312134829125161	0.133254784169113	-2.34239116495084	0.0191606213926476	0.191202478269387
Dap	2122.1663894853	0.271724415720726	0.116032739394951	2.34179092157632	0.0191914625070925	0.191335553695954
Gzf1	610.106530130093	-0.243397127819512	0.103950267089813	-2.34147669490082	0.0192076250894848	0.191335553695954
Hmmr	57.8372107847667	0.847393372235313	0.362026331297904	2.34069541073799	0.0192478628445856	0.191568484318353
Xdh	778.478526804956	0.390917786787046	0.167118231964171	2.33916899546219	0.0193266890133491	0.192016737172837
Yipf1	786.888026968773	0.28965877663501	0.123815805892116	2.33943295484743	0.0193130376501911	0.192016737172837
Kifc3	1305.30980345472	-0.450227339708007	0.192555109519571	-2.33817394319649	0.0193782266307051	0.192283367558958
Map3k8	87.3703300153235	-0.676726019991771	0.289446812143282	-2.33799783449257	0.0193873604806058	0.192283367558958
Dpt	484.804177131637	-1.53749336514209	0.657920352442229	-2.33689892619198	0.0194444402023947	0.192681349032623
Gprc5b	263.087512960288	-0.607860537977003	0.260363625576807	-2.33465998420614	0.019561190267232	0.193507519606951
Rhcg	3145.70179692811	0.268473405244635	0.114995283141093	2.33464710822297	0.0195618634548994	0.193507519606951
Clk4	795.760496278551	-0.479285207126221	0.205344856292456	-2.33405022058899	0.0195930924111005	0.19364790290832
Pcsk1n	191.183115897741	0.484997294338446	0.207829834392195	2.33362691048105	0.0196152662348259	0.193698623827134
Dram	188.270758233524	-0.913390714850646	0.391630511421593	-2.33227669502843	0.0196861397194655	0.194112862953914

Etv1	63.5609163620794	1.27502625124315	0.546710712081436	2.33217718816753	0.0196913717214379	0.194112862953914
Lonp2	2152.57326152576	0.324668012358267	0.139252665269376	2.33150303967409	0.0197268499765328	0.194294087377186
Akr1c14	191.660315645932	-0.547239858539204	0.23483083940742	-2.33035771587806	0.0197872526486275	0.194383676408211
Cdca2	36.1976307335944	0.991789103279723	0.425550811828984	2.33060089585329	0.0197744142115397	0.194383676408211
Phf11	68.5620906200652	-0.87404430060195	0.375013109028553	-2.33070332625466	0.0197690086811544	0.194383676408211
Lhfpl2	173.400770704952	-0.604198453136984	0.259361972202125	-2.32955682749868	0.0198295861982741	0.194631327054908
Klf7	78.1938461647486	-0.822784650341031	0.353254643195667	-2.32915452405049	0.0198508810724594	0.194672229740789
Akr1b10	33.3291613956645	1.68098848990907	0.722229606676667	2.32749872667796	0.0199387366834826	0.195365242365917
Nap1l5	13.7608646160175	-1.58442981006076	0.681326821730644	-2.32550629085192	0.0200449039268095	0.196236329054364
Cenpj	335.086200265837	-0.414320897846626	0.178213273790107	-2.32485992224462	0.0200794516077173	0.196236497827441
Lca5	151.673829573257	0.721305734004292	0.31023488376207	2.32503103860328	0.0200703005754011	0.196236497827441
Hmgn3	224.036422642257	-0.5478607082211	0.235911918003155	-2.32231043203919	0.0202162262329276	0.197403459934239
Trim47	345.804502500665	0.516213625784217	0.222360175096752	2.32152014433162	0.0202587881771043	0.197649258730444
Crebbp	930.639130722614	-0.295060767886164	0.127151746932488	-2.32054041729233	0.0203116611364202	0.197995146206305
Cd209a	23.4103905704012	-1.57537749583665	0.679075537162779	-2.31988550554872	0.0203470718747393	0.198170367547804
Hps4	855.802498618419	0.326972361393391	0.141061334374393	2.31794462205758	0.0204523309141545	0.199024994152637
Ctsw	20.7224544750304	-1.2776202339353	0.55182906638521	-2.31524635391976	0.0205994537800777	0.200285193895948
LOC100365744	163.212824325584	0.886018961342828	0.383095235931407	2.31279034099361	0.0207341691036903	0.201250696868099
Napa	1977.23875724384	0.283266913963217	0.122466910833555	2.3130077507075	0.020722213007215	0.201250696868099
Cpped1	1260.1679847903	0.224272888691829	0.0969884029660491	2.31236809590871	0.0207574069736614	0.201304341009075
Bai1	37.4485991739007	1.21087858604373	0.523807356658834	2.31168686474253	0.0207949457673312	0.201348870831388
Ldb2	122.909615249749	-1.09682729092501	0.474479770159578	-2.31164184419522	0.0207974286781673	0.201348870831388
Snn	2092.89558827385	-0.364976995368477	0.157932023185953	-2.31097524115641	0.0208342224969264	0.201533423744736
Loxl4	89.6453212938134	-1.18899349169863	0.515176165474596	-2.30793575359469	0.0210027101014285	0.202990478752412
Lynx1	226.01715497275	-0.727810960518142	0.315595047510943	-2.30615456819837	0.0211019971272536	0.203776804883912
Acy3	24730.0626601179	0.331217468858146	0.143709437488314	2.30477186917581	0.0211793533750043	0.204176870619422
Ifitm2	2209.33024968607	-0.524256839426016	0.227455155727957	-2.30488000040343	0.0211732949921141	0.204176870619422
Dlat	1502.88566485557	0.273813250876107	0.118834335627725	2.30415939492344	0.0212136976279059	0.204334650202355
Sipa1l1	1627.5016363081	0.193398327373245	0.0839535109315855	2.30363596742067	0.0212430870544696	0.204444477105081
Psmd11	1534.05405118011	0.333122935878638	0.144718428010177	2.30186950244657	0.0213425325555625	0.20522777075002
Gbp5	136.967152481429	-0.862504701237554	0.374792545521565	-2.30128563533003	0.0213754911636028	0.205256480508093
Snta1	937.685634865167	0.356884454279507	0.155087798429447	2.30117686815872	0.0213816358368451	0.205256480508093
Wwp1	4149.44125655111	0.38915830368036	0.169168803823756	2.3004141123194	0.0214247700627577	0.205496992855109
Hnrnph3	489.016299021611	-0.373521715583526	0.162466302589873	-2.29907192832743	0.0215008552350208	0.20587929284014
Serpinb1a	1156.50260892013	0.56825518961781	0.24716425499913	2.29909939695693	0.0214992957497477	0.20587929284014
Map3k12	896.896884649792	-0.232524594677284	0.10118367911038	-2.29804447438233	0.0215592579896092	0.20615811719641
RGD1563547	841.384242448544	-0.324601030885089	0.141258530125341	-2.29792162354419	0.0215662503384243	0.20615811719641
Lamc3	267.285439758414	-2.06875836019578	0.90058699208463	-2.29712218628334	0.0216118004633231	0.206419936190025
Dpysl2	470.165311076633	-0.735835675612956	0.320774713456504	-2.29393292159462	0.0217943520400762	0.207988753390014
Sptssb	63.9023758560617	-1.23470936772418	0.538858298526396	-2.29134332922165	0.021943564441969	0.209237041482734
Etnk1	13683.6314856495	-0.49044093193015	0.214184540685148	-2.28980546570399	0.0220325961820843	0.209419007062117
Kdelc2	348.294031028445	-0.59795797982188	0.261146582986779	-2.28974077693429	0.02203634809487	0.209419007062117
Ndel1	2165.98357291236	0.445243327019399	0.194446725115449	2.2897959672761	0.0220331470508278	0.209419007062117
Pygo2	474.133316172618	-0.311693659518696	0.136084365503993	-2.29044430169718	0.0219955738800842	0.209419007062117
Tarsl2	71.6450250248577	0.599593519406698	0.26193462249055	2.28909608705253	0.0220737701312949	0.209599391238344
Epha1	629.311853446673	-0.343209416098209	0.149963391796163	-2.28862132276065	0.0221013639021862	0.209686228808221
Mkln1	948.595526129048	0.284522686143198	0.12440761514211	2.28701985660757	0.0221946641168691	0.21012283700434
Nxf1	1996.83329907624	-0.334346558401926	0.146163636146548	-2.28748112195773	0.0221677560867689	0.21012283700434
Ranbp2	2905.68713379946	-0.211242622300229	0.0923715487780043	-2.28687972752202	0.0222028442057199	0.21012283700434
Aadac	90.0843527036204	1.04170867976137	0.455771232156571	2.2855955055178	0.0222779333567492	0.210482951398846
Cd68	54.2384500799757	-1.23746862677962	0.541357022498939	-2.28586418084573	0.0222622055245675	0.210482951398846
Sorcs1	187.569513502675	0.46688954030655	0.204369330799173	2.28453818623768	0.0223399210334097	0.210893307695793
Hpse2	23.7945194227563	-1.1227446205342	0.491545272680118	-2.28411233498903	0.0223649298352075	0.210954184653086
Ppp6r2	1083.19939965671	0.227136677617806	0.0994711372914114	2.28344305496764	0.0224042835740592	0.211150155143248
Ar	30.3713037402938	1.27265763000677	0.558245143146284	2.27974689190135	0.0226227044404571	0.211878314272326
Dapp1	42.1230522892895	-1.04161032639814	0.456969780162056	-2.27938557781381	0.0226441548811284	0.211878314272326
Gata5	33.5150612248598	0.974773391378426	0.427663742910264	2.27929864885217	0.0226493183037899	0.211878314272326
Gstt3	18039.5635638241	0.504775096845549	0.221303492685159	2.28091789569573	0.0225533057791294	0.211878314272326
Ifi44	247.44426468855	-0.601251517030721	0.263694283603189	-2.28010827089257	0.0226012678129054	0.211878314272326
Mfsd1	2449.01508202637	0.315254075297145	0.138249576091792	2.28032580069417	0.0225883726838111	0.211878314272326
Plagl1	440.929940555555	-0.592112881248162	0.259716882248413	-2.27983978600906	0.0226171923715643	0.211878314272326
Tcerg1	1029.88704905797	-0.233343529778225	0.102357376528307	-2.27969431898926	0.0226258244829968	0.211878314272326
Wnt7b	66.3917373617418	-1.42081035811091	0.623268579760158	-2.27961171836652	0.0226307273342558	0.211878314272326
Zfp641	326.469521418995	-0.395602336007327	0.173670557514898	-2.27788948033631	0.0227331632020661	0.212487773811417
Acmsd	1346.19469346768	0.565749242001369	0.248405920002877	2.27751915894282	0.02275524186021	0.212519374678017
Slc25a10	7451.18429850134	0.355439049679262	0.156124228855951	2.27664246788504	0.0228075846880878	0.212833339544176
Actn4	7404.10030211593	0.215823776109738	0.0949017001402627	2.27418239916413	0.0229550220459804	0.213307539352677
Atp6v0a4	6445.10827867272	0.277948854192664	0.122233532591539	2.27391656200815	0.0229710037099839	0.213307539352677
Ctbp2	680.300399112556	-0.243051276727821	0.106857650115333	-2.27453323618378	0.0229339451236926	0.213307539352677
Elk3	294.524372108406	-0.863464930155454	0.379596512349208	-2.27469142119281	0.0229244474839457	0.213307539352677
Fnbp1	337.925120903207	0.403490240885239	0.177373066512187	2.27481121468639	0.0229172571958191	0.213307539352677
Klk1c6	19.3996105121753	1.73721585231597	0.76377751577299	2.27450509662856	0.0229356350183157	0.213307539352677
Ptgfr	45.7085975830977	-0.835814667010305	0.367709256339553	-2.27303134908981	0.0230242908524187	0.213477127958012
Vps45	551.838762497797	0.263977085764395	0.116136533254016	2.27298919959164	0.0230268308003276	0.213477127958012
Gypc	56.7714987608792	-1.3299732873562	0.585717644365625	-2.27067307968272	0.0231667758475122	0.214076076652702
Kazn	851.748081781322	-0.648964558275146	0.285691448563836	-2.27155751961592	0.0231132491241357	0.214076076652702
Mrgbp	228.140587253389	-0.359380786198137	0.158229145405321	-2.27126794673349	0.0231307623748448	0.214076076652702
RT1-O1	52.401588924589	-0.939319178342961	0.413667034001928	-2.27071316091043	0.0231643477862311	0.214076076652702
Dkc1	1778.92639732666	0.264099339390942	0.116341309109305	2.2700392613153	0.0232052010121134	0.214252778647892
Inhbb	44.1011954045788	-0.870236474444006	0.383685585730799	-2.26809790830813	0.0233232398391345	0.214252778647892
Mmgt2	174.461508611201	-0.536845530621433	0.236524105946006	-2.26972861169586	0.023224054308494	0.214252778647892
Morf4l2	2323.27547167816	-0.246071608443459	0.108477334224313	-2.26841496615896	0.0233039264366505	0.214252778647892
Thumpd3	105.619333577976	0.584082389566843	0.257535903949678	2.26796489580331	0.0233313463583151	0.214252778647892
Tmem173	92.5872580480836	-0.708087898217057	0.312210966567729	-2.26797894385766	0.023330490076524	0.214252778647892
Trim39	407.145241202521	-0.317814558324407	0.14006278326796	-2.26908641188703	0.0232630715241327	0.214252778647892
Trim45	77.5736947157928	-0.550170956447364	0.242592936490998	-2.26787706355077	0.0233367006832738	0.214252778647892
B4galnt3	13.3237801946657	-1.88947905272776	0.833465120707012	-2.26701634631688	0.0233892271688686	0.214388674194645
Shc2	70.4997765982845	-0.668042023965269	0.29465937233715	-2.26716706367274	0.0233800220243076	0.214388674194645
LOC100302465	22.6773140154057	-0.993783450736703	0.438447589139487	-2.26659576960416	0.0234149307601462	0.214451332006303
Lrrc40	431.350454772819	-0.323445013488906	0.142738733007401	-2.26599330591042	0.0234517931080527	0.214616006816528
Ednra	58.4272793309748	-1.1507551813361	0.507999835193348	-2.26526683989595	0.0234963096400942	0.214677697242211
Ppara	68.2151533108351	0.801111610038477	0.35362868221132	2.26540337460453	0.0234879374464518	0.214677697242211
Dpp4	22735.1352771332	0.259617907955664	0.11469026883321	2.26364373016875	0.0235960359371842	0.215242812569852
Nav2	800.021788907902	0.472193829731908	0.208582564545418	2.26382215004883	0.0235850556137741	0.215242812569852
Coq9	2645.07047405447	0.250312800011574	0.110625185842275	2.26271077517972	0.0236535241604899	0.215594190543808
Phlda1	329.683617229814	0.39719845499438	0.175594578529139	2.26202003684566	0.0236961653718697	0.215809788154384
Top3b	753.557520589279	-0.306268362774097	0.135447322005151	-2.26116218645098	0.023749215714013	0.216119764455942
LOC679149	39.6219288200207	-0.992835179973669	0.439215350275257	-2.26047468366362	0.023791805932505	0.216168384440535
Rpp25	21.8386204848444	1.12160447599183	0.496183748100844	2.26046193629839	0.0237925962462704	0.216168384440535
Fgfrl1	500.614806239115	0.356852057332119	0.158204683308723	2.25563523069511	0.0240934849278138	0.21872727610985
Rab27a	111.826158808446	-0.744685360699542	0.330231923216262	-2.25503747017172	0.0241309769396503	0.218892804386325
Ncoa5	515.353929531113	-0.331742729660905	0.147213697544452	-2.25347732714025	0.0242290687258776	0.21925973668946
Sh2b1	813.264554773834	-0.346147979600142	0.153591064086604	-2.25369868786743	0.0242151299746074	0.21925973668946
Ttc27	507.115316209095	0.345940699059617	0.153514419586992	2.25347364755909	0.0242293004823123	0.21925973668946
Csrnp1	729.08670610257	-0.650905835243441	0.288919235447726	-2.25289892600872	0.0242655226082288	0.219412832112274

Lsp1	104.78028988476	-1.04709577175151	0.464852640558765	-2.25253269615263	0.0242886289141932	0.21944718301965
Polr3b	512.535048999414	-0.41187357849886	0.182969310099311	-2.25105280374783	0.0243821931663474	0.220117559593888
Aifm2	143.015950530781	0.674134206590356	0.299610193467701	2.25003761984163	0.0244465573298055	0.220523468738547
Cep290	188.273532231125	-0.522819216203881	0.232419549784466	-2.24946316559307	0.0244830437720261	0.220559760563124
Slc39a6	170.226392639741	-0.386032084201298	0.171618378705961	-2.24936330894198	0.0244893909757753	0.220559760563124
Ptk2b	821.48492114029	-0.45604121982211	0.202834720992722	-2.24833902987681	0.0245545797530676	0.22097177630512
Sectm1b	1741.97246073826	0.951192788628611	0.423190743789453	2.24766917185186	0.0245972931788209	0.221181039770948
Aldh1b1	588.98462456231	-0.562196593877366	0.250504101504555	-2.24426103405394	0.0248156110889133	0.221814589176001
Cygb	380.148928107585	-1.28393113134565	0.572282669138487	-2.24352614640328	0.0248629057373065	0.221814589176001
Fads1	753.939867152294	0.40094237544614	0.178555430811819	2.24547846919704	0.0247374329246714	0.221814589176001
Il33	4620.88973693749	0.457067901722852	0.203663048156444	2.24423578975286	0.0248172344254322	0.221814589176001
Kcnip3	12.130925062739	-2.29877600939911	1.02461298410312	-2.24355541561998	0.024861020587353	0.221814589176001
LOC100911576	1162.63564982825	-0.255106967230625	0.113622152245923	-2.24522209963487	0.0247538780600904	0.221814589176001
Pcdhga3	21.198121576693	-1.66627192458561	0.742623853448293	-2.24376299905862	0.0248476542593618	0.221814589176001
Slc4a9	994.564535922099	0.556133084506691	0.247877916871752	2.24357656190255	0.0248596586903275	0.221814589176001
Tmem123	3130.11619993409	-0.209208877072512	0.0932064556316773	-2.24457496698769	0.0247954312918263	0.221814589176001
Tmem79	269.207010664965	0.398160148097277	0.177429801884908	2.24404324339802	0.0248296191583506	0.221814589176001
Mccc2	3543.17605011041	0.312240195061518	0.139249947616094	2.24230026945755	0.0249419723591471	0.222345457124757
Decr2	3993.17227172454	0.346406989279097	0.15451731488572	2.24186518860554	0.0249700865110978	0.222421632668603
Tmem165	185.221715692743	-0.369205602706102	0.164716819960368	-2.24145659681224	0.0249965139677302	0.222482676395631
Sox6	83.0879921789754	0.580817931215126	0.259177298168016	2.24100619660986	0.0250256736240401	0.222567923639155
Idh3a	2450.27439504175	0.215207242157959	0.0960639804267972	2.24024906319545	0.0250747580666916	0.222656015770326
Pde12	184.933462041919	0.470125977783629	0.209849500369416	2.24030067718067	0.0250714093219624	0.222656015770326
Eno1	10855.2492132127	0.236803152573214	0.105724534366325	2.23981267917165	0.0251030863912392	0.222733551852322
Zfp36	3391.03051887313	0.620202089385513	0.276936896433658	2.23950689623651	0.0251229531151863	0.222735947821535
Lpgat1	945.796506770931	0.464436519533203	0.207462815048215	2.2386494631593	0.0251787331694395	0.223056493533787
Hspa2	1109.93560200035	-0.498249897094017	0.222601306227363	-2.23830625946601	0.0252010902087204	0.223080678592147
Ube2c	70.8458183522138	0.728543627662634	0.325634881743862	2.23730217033595	0.0252665974725642	0.223486495621139
Limd2	164.558626453685	-0.764809197823871	0.342060548714028	-2.23588835572872	0.0253590852519432	0.223608505022178
Loxl1	258.205418409022	-1.41505908048289	0.632805011649247	-2.23616920604799	0.0253406895294973	0.223608505022178
Lrif1	247.106197778988	-0.481007407856504	0.215072645199629	-2.23648808248039	0.0253198170936357	0.223608505022178
Ucp1	249.57092910287	3.97646953328147	1.77815696627751	2.23628712689298	0.0253329691460082	0.223608505022178
Folr2	36.2845581399963	-1.29967968776325	0.581574837953015	-2.2347591452508	0.0254331653341724	0.22391429681503
Ugt1a7c	179.518592365385	0.652542726772127	0.291966758502023	2.23498979856505	0.0254180184857912	0.22391429681503
Ankra2	388.131333055645	0.425624229074249	0.190495579249199	2.23429977090157	0.0254633554079134	0.224006577063734
Acsf2	293.54024260547	0.707876388402919	0.31701089863202	2.23297177307651	0.0255508058495675	0.224602056679184
Hbb	6999.8693933755	0.554283794500082	0.248320294162618	2.23213248183855	0.0256062082008473	0.224657445162396
P2ry6	26.9720460825583	-1.29016623774641	0.578033656995463	-2.23199154951031	0.0256155214503441	0.224657445162396
Zfp532	352.066100916709	-0.366286716511012	0.164108555043683	-2.23197819524712	0.0256164040938294	0.224657445162396
Cacna1a	42.6720334439896	0.886196833515126	0.39723606129493	2.23090731145168	0.0256872693320577	0.225025441317459
Txk	67.3789345020244	-0.80445867284404	0.36062316566752	-2.23074596817699	0.0256979608331921	0.225025441317459
Gltp	653.868593602801	-0.373269660036449	0.167446560664989	-2.22918678385548	0.025801479517731	0.225659700726941
LOC680549	21.0988871797857	-1.24366015068522	0.557931024943396	-2.22905716851182	0.0258101012620995	0.225659700726941
Gng7	897.591732387253	1.0625892356707	0.476795409769425	2.22860626150861	0.0258401140751147	0.225748452404115
Grhpr	10659.8870824502	0.451873498166703	0.20279432851809	2.22823538246236	0.0258648227644993	0.225790764624654
Nudt12	1824.05967662854	0.325030822312454	0.145891699006466	2.22789113106461	0.0258877757479686	0.225817696969617
Anxa5	1858.55114851348	-0.521383064990253	0.234234427288546	-2.22590278903783	0.0260206935200865	0.226503384897388
Dmp1	9.6955185270833	1.70435434466435	0.765673431081009	2.22595466354118	0.0260172183038442	0.226503384897388
LOC100302372	305.187388273903	-0.810257182326053	0.364026195078747	-2.22582109002012	0.0260261675766311	0.226503384897388
Enpp1	299.851157830298	0.411347360450284	0.184871885156987	2.22504011413624	0.026078545350384	0.226785574944502
Dact2	1070.32995372973	0.560913235105371	0.252127615483255	2.2247195493848	0.0261000710557608	0.226799241299524
Kif3c	38.0778519231456	-1.32315439863081	0.594990875122987	-2.22382300965087	0.0261603546519672	0.226976023644473
Mpp7	59.7111989243817	-0.756199288892375	0.340020396187477	-2.2239821415755	0.0261496457978011	0.226976023644473
Ccdc115	157.030672779262	0.411306565090973	0.184985204010852	2.223456558541	0.0261850295595466	0.227016816150882
Abhd6	791.601771842689	-0.228457378317643	0.102907754232712	-2.22002102777422	0.0264173402504184	0.228542221275855
Flt3	22.5545775328808	-1.4431624034628	0.650103127743352	-2.21989764681233	0.0264257162945319	0.228542221275855
Gclc	181903.662785426	0.388330595184492	0.174890677376313	2.22041906984509	0.0263903337431711	0.228542221275855
RT1-CE4	584.685584272233	-0.443880118551954	0.199976029584181	-2.2196666244196	0.0264414060335869	0.228542221275855
Acadsb	411.670790702789	0.467932272527645	0.210906063094967	2.21867624695523	0.0265087581296872	0.228950262083605
Gabbr1	610.976558105678	-0.313127125665331	0.141250182612886	-2.21682634226035	0.0266349606857766	0.229052917853334
Gcnt2	813.267887749832	-0.228269991409875	0.102982447582863	-2.21659124217455	0.0266510365823366	0.229052917853334
Olfml2b	121.114362753328	-1.89035776461723	0.852278176600068	-2.21800559549501	0.0265544508991582	0.229052917853334
Oscp1	292.871338146046	0.361046350923107	0.162931206786004	2.21594351410722	0.0266953709038522	0.229052917853334
Pars2	315.783836542361	0.309001713756922	0.139407396547754	2.21653743925326	0.0266547167476537	0.229052917853334
Rbm5	1534.72956427813	-0.284601500050322	0.128386197910631	-2.21676087213387	0.0266394366225021	0.229052917853334
Sfxn5	86.9898935689118	0.708212711821963	0.319541331882763	2.21634149062695	0.0266681235097936	0.229052917853334
St8sia1	17.3264482383129	-1.66100519782746	0.749700709503323	-2.21555772426556	0.0267218068858837	0.229052917853334
Tspan18	496.737766471508	0.305350605921028	0.137792626708621	2.2160155678484	0.0266904359751218	0.229052917853334
Ubox5	269.654990177331	0.359187071186993	0.162120771134276	2.215552446944	0.026722168667387	0.229052917853334
Ap1s1	1668.69748676999	0.210800916068199	0.0951815875053596	2.21472368336291	0.0267790362449361	0.229258014900358
Med16	963.270523310419	0.320392981191824	0.144675415360544	2.21456410125643	0.0267899983401295	0.229258014900358
Prex1	114.576490876704	-1.1196546779795	0.505642118396773	-2.2143224174631	0.0268066075842492	0.229258014900358
Usp29	83.5423892867322	-0.632147370535107	0.285525016106946	-2.21398243542461	0.0268299872338928	0.229285439774756
Cbs	4389.56093716088	0.236341087534334	0.106849856291114	2.21189897429922	0.0269736461516083	0.229510826036195
Cyb5r3	3006.66938648745	0.398539050112323	0.180121869291442	2.21260778427452	0.026924697897843	0.229510826036195
Dbnl	1383.72804268936	0.191478992799878	0.0865594081125812	2.21211069917247	0.0269590170380875	0.229510826036195
Gpc4	3802.50921453178	0.273668241330569	0.123725709842601	2.21189469576468	0.0269739418472461	0.229510826036195
Lrat	29.8164019762619	-1.22276678401995	0.552827124301475	-2.21184296187524	0.0269775174717892	0.229510826036195
Rps6ka5	180.980382673222	-0.541575434247201	0.24485062643299	-2.21186052140005	0.0269763037868812	0.229510826036195
Gpx8	222.643769222459	-0.432681522820549	0.195659812943399	-2.21139699722454	0.027008357606076	0.229601340725998
Arhgap4	204.10783898796	-0.460487041526983	0.208348618637727	-2.21017563993391	0.0270929749171587	0.230148544774609
Pkd2l2	228.063011927287	-0.623729708722896	0.282296679739574	-2.20948297832728	0.0271410650739205	0.230384873510217
Tnfrsf14	794.672067654003	-0.353162553659557	0.159866117785585	-2.20911446747724	0.0271666800811803	0.230430213285594
Jam2	359.51305468579	-1.32878285714975	0.601939081168078	-2.20750387991293	0.0272788762336986	0.230946830907809
Lrrn3	8.01215446454824	-2.45036434906271	1.10986715527227	-2.20779967892787	0.0272582404695952	0.230946830907809
Nipsnap1	5443.89332384669	0.336399424870484	0.152398615392875	2.20736536223288	0.0272885442468052	0.230946830907809
Acat1	7693.41200074887	0.213683496295756	0.0968342113143882	2.20669423951827	0.0273354279648733	0.230999609106877
Top2a	149.386154542959	0.768954909581501	0.348418749789352	2.20698487106793	0.0273151163159085	0.230999609106877
Plcxd2	135.639765244905	0.490332268599026	0.222309419853254	2.20562974309722	0.0274099347790177	0.23142748812082
Vtcn1	36.3253840987377	-1.13134378439495	0.512990497002394	-2.20538936102294	0.0274267839608257	0.23142748812082
Pfn3	270.92275473506	0.72737375465975	0.329878690273166	2.20497345268779	0.0274559574690361	0.231501789757466
Hoxb8	494.418041334137	0.319523328170737	0.145060661642386	2.20268765186284	0.0276167711844233	0.232647694933837
LOC246295	1184.96310584021	0.211346005722311	0.0959703798485887	2.20220036698562	0.0276511581571787	0.232647694933837
Rgl1	1284.61770765394	0.388872283184588	0.176585910948938	2.20217049647316	0.0276532672757007	0.232647694933837
Cdh1	2483.31453943538	-0.214173421168503	0.0973484808155999	-2.20006947590889	0.0278019661445324	0.233048579722601
Gnpda1	1265.3341474102	0.362823083383964	0.164910756577674	2.20011775407186	0.0277985415535737	0.233048579722601
Hmox2-ps1	2363.53459816769	-0.339625815623073	0.154328077236118	-2.20067418518701	0.0277590975961094	0.233048579722601
Vapb	358.262571532579	0.340220456340543	0.154642236595491	2.20004873073896	0.0278034378060749	0.233048579722601
Ythdc1	1054.3253262116	-0.242001749877398	0.109983803188435	-2.20033989425494	0.027782788820892	0.233048579722601
Klhdc10	194.59805640497	0.551288851021835	0.250614859841271	2.19974526399192	0.0278249734044035	0.233057220128556
Zbtb7b	654.927096775503	0.441933014393524	0.200958117891724	2.19912994324339	0.0278686839545888	0.233251444644961
St3gal6	1036.696104373	-0.225124558870311	0.102399790784717	-2.19848651198524	0.0279144546980971	0.233462613759066
Mob1a	1108.05451597474	-0.309261443064214	0.140756592383768	-2.19713647387131	0.0280107008856544	0.233483489679968
P4ha2	393.547905448116	-0.576859626631191	0.262599331450426	-2.19672922792681	0.0280397901442994	0.233483489679968
Plcg2	171.018961405	0.608501696044179	0.276931095788247	2.19730360836569	0.0279987701304019	0.233483489679968

Rhobtb3	140.57712799385	-0.480331559371664	0.218642671403878	-2.19687930213948	0.0280290674325602	0.233483489679968
Syngr1	95.3029791969327	0.656307370835685	0.298766499978831	2.19672343078018	0.0280402044178388	0.233483489679968
Zc3h7a	963.731036768951	-0.303079378827253	0.137879400439763	-2.1981483663302	0.0279385347943752	0.233483489679968
Klhl22	757.436338141271	0.246390733599444	0.112304215798735	2.19395800813934	0.0282384285127708	0.234618405318825
Stx3	168.555387198737	0.512153169195232	0.233381189138706	2.19449207147044	0.0282000533150003	0.234618405318825
Wbscr16	666.069692730994	0.247572193132001	0.112840551972709	2.19400019588595	0.0282353954700567	0.234618405318825
Kcnc4	32.8175031329974	1.12666945039114	0.513665268112557	2.19339231272351	0.0282791257065932	0.234784910723987
Rmdn3	1571.32166534455	0.311514563690896	0.142098398078219	2.19224542925122	0.0283617897789399	0.235299344983527
LOC687713	188.376465358297	0.452279584417401	0.206378884895922	2.19150125094187	0.0284155393149635	0.235511610024794
Wnt4	21.7486654759001	-1.30300626162584	0.594622274487443	-2.19131761040909	0.0284288165541103	0.235511610024794
Ahnak	3036.94100915454	-0.468584283291312	0.213866757028345	-2.19101037394609	0.0284510417485684	0.235524064467755
Tbc1d10a	1310.32700074347	0.252475316982502	0.115250547109077	2.19066480216835	0.0284760579674453	0.235559588688488
Ntmt1	500.981962487985	0.421660625482506	0.192613316355205	2.18915614694524	0.0285854927760024	0.236292880648759
Clec1b	14.6962775378765	-1.56882100412293	0.717101346104378	-2.18772564386538	0.0286895929513039	0.236465492011979
Cmip	562.88109638673	0.603993984993641	0.27599805462297	2.18839942846239	0.0286405198839083	0.236465492011979
Srsf2	4700.53355982825	-0.30491756983644	0.139361911978429	-2.1879548400831	0.0286728920168016	0.236465492011979
Ubp1	702.110125017111	-0.25387013078231	0.116028648379966	-2.18799524364833	0.0286699487818947	0.236465492011979
Atp6v1b1	2513.56644119575	0.357695104048916	0.163560016985847	2.18693486733906	0.0287472791139821	0.236737450439475
March5	1527.97018761301	0.324775054702585	0.148522740718043	2.18670254219953	0.0287642459138585	0.236737450439475
Cdh13	111.452162423324	-0.867941065029181	0.397420026599461	-2.18393892339989	0.0289667363700392	0.237372693281807
Lphn3	42.2926302573941	-0.820326966600841	0.375496388885577	-2.18464675262381	0.028914757152269	0.237372693281807
Psmb10	253.278761220965	-0.432625150537106	0.198069520866847	-2.18420859829282	0.0289469234015876	0.237372693281807
Scly	3697.64164560969	0.35319722815614	0.161663507690387	2.18476781310828	0.0289058751591142	0.237372693281807
Slc18a2	159.523439429309	-0.381641653547685	0.174680146053643	-2.18480269320639	0.028903316504042	0.237372693281807
Stxbp2	1060.26769863208	0.197624807111375	0.0904752034091155	2.18429801387398	0.028940356629841	0.237372693281807
RT1-Da	774.990221705411	-0.986554519987587	0.451984020441711	-2.18271990904337	0.0290564428599217	0.237936260479733
Dync1li1	444.731365280836	0.3070935186788	0.140807965150685	2.18093854527452	0.0291879622466724	0.238047566347217
Elf5	118.236074278373	-0.905722003947564	0.415196016539338	-2.18143230635198	0.02915145628773	0.238047566347217
Fgf1	196.170486700393	0.505731661799909	0.231958462394199	2.18026821086807	0.0292375860127323	0.238047566347217
Gstm7	362.462516881098	0.734925371214516	0.337032463929697	2.18057739199811	0.0292146888237234	0.238047566347217
Leprel4	43.7392061822828	-0.801768756943249	0.367697208720998	-2.18051357999733	0.0292194133184185	0.238047566347217
LOC498154	241.985265321332	0.391064502606702	0.179271457113241	2.18140973975392	0.0291531238797115	0.238047566347217
RGD1305939	70.5521001895551	-0.582567043784486	0.267109531234007	-2.18100432842329	0.0291830963347105	0.238047566347217
RGD1311783	623.519103848403	0.260268925325417	0.119314148622308	2.18137520428783	0.0291556760877646	0.238047566347217
Slc25a37	160.900690719846	0.444713039529299	0.204064583010318	2.17927595748848	0.0293111741717155	0.238475880913184
Mrpl37	1229.14834029981	0.239879130482709	0.11012264416608	2.17829068943295	0.0293844019003243	0.238900652359861
Oasl	72.6383014609333	-0.634270531410174	0.291251312982862	-2.17774308007153	0.0294251696821217	0.239061099790561
Csgalnact2	211.673327973863	-0.466806707881643	0.214477177019957	-2.17648662840339	0.029518892458732	0.239192223088019
Ctdspl	1643.39051759408	-0.456047893870351	0.209525673548244	-2.17657285690741	0.0295124522008424	0.239192223088019
Faim3	33.5291645958691	-1.65801438674356	0.761660735233007	-2.17684109216466	0.0294924258999739	0.239192223088019
Pdlim2	297.692892644287	0.507514359013109	0.233190013132195	2.17639834655098	0.0295254873299746	0.239192223088019
Rgs9	22.1311872568465	-1.1121674529243	0.511544478208561	-2.17413636604805	0.0296948955735636	0.240393292798521
Spetex-2G	7.29654968839961	8.447353107119	3.8869899854901	2.17323768228178	0.029762433029323	0.240768550755363
Irf3	2420.5222017058	-0.257043291413831	0.118311597106953	-2.17259590521347	0.0298107443546849	0.240987852301101
Sec24a	470.681119752184	0.309091579486659	0.142304016108561	2.17205099293093	0.0298518168606041	0.241148365627311
Dnaja4	953.474289105949	0.509308912398416	0.234516955604803	2.17173598849151	0.0298755823553321	0.241168941087149
Kcnn1	73.9732010102144	-0.778520457185657	0.358576316244686	-2.17114299499471	0.0299203648536689	0.241187848884256
Spatc1	705.799299992041	0.394655696036027	0.181751330624253	2.17140471368502	0.0299005929127264	0.241187848884256
Rasd2	67.5480652883907	0.895149370843254	0.412517450080734	2.16996728421565	0.0300093244431739	0.24173350929916
Rps6ka3	1108.41833960131	0.201156118373732	0.0927713295626259	2.16830047949179	0.0301358319831813	0.242580641870283
Tomm40	963.907821703392	0.307229714638127	0.141753053914342	2.16735869989636	0.0302075138269937	0.242985564159667
Kif22	96.367059935244	0.644901876597488	0.297600712507313	2.16700380575077	0.0302345639334018	0.243031155351517
Cd83	56.7800139362049	-0.986779493567613	0.455589347024741	-2.1659406656715	0.0303157212940607	0.2432911503909
Lilrb4	27.5033309033795	-1.55291232415752	0.716937277278893	-2.16603651863596	0.0303083964581366	0.2432911503909
Myef2	1266.00336670321	-0.228977998108433	0.105727412520542	-2.16573916498661	0.0303311244152653	0.2432911503909
Slc40a1	598.619119212351	0.349102001850774	0.161248836063913	2.16498928223211	0.0303885060609341	0.243579520372762
Cacna1c	24.6467711119924	-1.38968571109427	0.642326692950795	-2.16351854336019	0.0305013190707107	0.243967623193313
Ifi204	27.0103528988242	-1.07635389941687	0.4974435450472	-2.16377096483328	0.0304819315509287	0.243967623193313
Pcdh12	55.5735024083424	-1.54826696411895	0.715542236295061	-2.16376739986108	0.0304822052889836	0.243967623193313
Ccl6	23.5078247348097	-1.21322862869707	0.561377062851843	-2.16116530043777	0.0306825727919931	0.245072468273924
LOC684035	1989.22212417161	0.247724776142872	0.114624664500732	2.16118212621937	0.0306812735447319	0.245072468273924
Cacna1g	94.5502613618203	-1.14199133580311	0.528502059514039	-2.16080773053785	0.0307101946709389	0.245120837520991
Moap1	289.125873583161	0.432105717708947	0.200234675457139	2.15799644453411	0.0309281082321582	0.246686932046814
Gucy1b3	43.3228751328282	-1.10913150174978	0.51426093210876	-2.15674851519775	0.031025264607936	0.247115036814156
P2rx5	7.27458835433983	2.34649422106691	1.08784471828916	2.15701210073181	0.0310047216079584	0.247115036814156
Amigo2	61.5088515398709	-1.15419772120597	0.53542245154	-2.15567673317813	0.0311089161918873	0.2474345286473
Shank3	570.729327630272	-0.677904719731684	0.31447334737851	-2.15568258926483	0.031108458604395	0.2474345286473
Tmie	18.6468714551073	1.121738301806	0.520498634307019	2.15512246886	0.031152251886679	0.247605940520275
Rabl2a	247.213346418697	-0.37620572102725	0.174668398902518	-2.15382818753156	0.0312536483075088	0.248064920854151
Slc27a1	549.018043164432	0.387397331840316	0.179858316893861	2.15390279710518	0.0312477955717655	0.248064920854151
Ap1s2	61.5109145259393	-0.879481236111224	0.408464867662577	-2.15313801929654	0.0313078330501615	0.248321584402049
Ctsb	11050.713699666	0.220693622574774	0.102516577089412	2.15276035194086	0.0313375176197338	0.248383699627541
Dld	5486.02492453865	0.297986415072462	0.138511192393975	2.15135260856671	0.0314483787852714	0.248569035656046
Igsf11	2238.86596886624	0.48278058794036	0.224390523676032	2.15151950283508	0.0314352181448487	0.248569035656046
Irf7	666.588551027344	-1.21232915993657	0.563452204559785	-2.15160957775246	0.0314281171463877	0.248569035656046
Lrrc10b	26.5110690955926	1.00546107147855	0.46723201897717	2.15195241473312	0.0314011023968397	0.248569035656046
RGD1561157	35.8136487059646	-0.787800776510264	0.366282151942915	-2.15080306897684	0.0314917467737843	0.248738842134004
Nuak1	78.651278609963	-1.00106500677554	0.465699034136014	-2.14959648484725	0.0315871467128523	0.249319103846027
Rbak	75.502219179525	-0.611974565447901	0.284784450755502	-2.14890442165786	0.0316419772469193	0.249578565849052
Gjb5	20.9556996106737	-1.82934034864925	0.851860781470827	-2.14746398524264	0.0317563614159363	0.250307076181368
Picalm	2120.54298631287	0.345409993188824	0.160899103938748	2.14674901682682	0.0318132681639807	0.250581847506448
Zfp266	798.198119255716	-0.282008694019124	0.131385513110018	-2.14642152961704	0.0318393631580044	0.250613713056702
Iqub	85.3459635693272	-0.74979867287658	0.34966277846064	-2.14434798058148	0.0320050150163463	0.251569156760575
Pcdh17	197.927674938938	0.607913918317799	0.283471263507546	2.14453454927228	0.031990080217697	0.251569156760575
Pygb	1200.40995651965	-0.463027671500491	0.215997027215479	-2.14367613049866	0.032058846047732	0.251818136958204
RGD1311892	233.269795860547	0.440210469089225	0.205397374452676	2.14321371079965	0.0320959418394931	0.251935410875469
Ptgs2	31.8780834760813	-2.00962588377726	0.937792920247718	-2.14293138750334	0.0321186081916029	0.251939337961186
Adra1b	104.854094793433	1.02043902940543	0.476736082919387	2.14046946720829	0.0323168457546944	0.253116174628649
Cyp7b1	94.9044326476915	-0.712763043435145	0.333029629355948	-2.14023912771236	0.0323354465564665	0.253116174628649
Tmtc2	39.8898594287915	-1.15454187455025	0.539312272709298	-2.14076692293738	0.032292838623096	0.253116174628649
Sarnp	1754.5201202806	-0.324794402396208	0.151865886054628	-2.13869230828691	0.0324605959713913	0.253920945499541
Col6a2	335.047918396682	-1.26320414213223	0.591284473007281	-2.13637293011865	0.0326490286868642	0.25521929852469
Slc2a5	3018.12136456381	-0.459288107520411	0.215071041689486	-2.13551812420901	0.0327187113495731	0.255588229002919
Nenf	433.832365477853	0.31239746216748	0.146328592301875	2.13490376182261	0.0327688719957047	0.25580425762581
Endog	455.506661817746	0.502758699892934	0.235546341802806	2.13443645970028	0.0328070697387092	0.255926667570465
Serpina3n	30.1642715129415	-1.00712872575471	0.47198348061622	-2.13382198131132	0.0328573558387745	0.256143145722573
RGD1559548	33.7954081997604	0.833857677472321	0.390984283099817	2.13271405914657	0.0329481898894107	0.256675206499686
Pdgfrb	497.133698602361	-1.20434084839637	0.564884411843785	-2.13201289174434	0.0330057867687283	0.25677191814741
Sptbn2	2522.05500695837	-0.371431219229351	0.174196233515768	-2.13225746465826	0.0329856867262578	0.25677191814741
Erg	101.366932028687	-1.1285999266258	0.529502035634587	-2.1314364264402	0.0330532046234475	0.256793375971204
Gltscr1	327.654932008131	-0.3644407423923	0.170984145628743	-2.13143002851041	0.033053731219943	0.256793375971204
Pld4	161.532941816256	-1.63046838046793	0.765525197569255	-2.12986899143895	0.0331824308201686	0.257617150752757
LOC367975	19.9339967838573	1.20542682548735	0.566273283659361	2.12870156560744	0.0332789592340404	0.25801408639434
Metrn	681.225550843834	0.514610100511987	0.241729115527633	2.12887098597421	0.0332649358510292	0.25801408639434
Emr1	81.6626639590281	-2.31720782717616	1.08917884211767	-2.1274814911671	0.0333800975699487	0.258621805712363

Zmym3	834.832771517933	-0.219010244011488	0.103005893874146	-2.12619138356373	0.0334873272614655	0.259275859437205
Capn2	1820.22676055919	-0.277195044617935	0.130477273699552	-2.124469930727	0.0336308678391154	0.25954903941127
Eya2	51.8640797000883	-1.19107692826254	0.560610815723981	-2.12460568875106	0.0336195287920282	0.25954903941127
Srsf6	3010.64859309453	-0.27687079459286	0.130317319697247	-2.12458938870203	0.0336208900638316	0.25954903941127
Stk10	161.587054942584	-0.562351168393287	0.264660996403798	-2.12479804744367	0.0336034678324153	0.25954903941127
Tk1	513.363774163592	0.367735615160445	0.173101006041586	2.12439906370099	0.0336367882326941	0.25954903941127
Fdft1	2378.12238794767	0.285741788811938	0.134526167538939	2.12406102128219	0.0336650413391444	0.259590814016767
Rin2	4612.68318016734	0.411019427758419	0.193619354348735	2.12282201405401	0.0337687693971686	0.26021412404625
B2m	15775.1829425729	-0.418627662443049	0.197247566349329	-2.12234640047042	0.0338086596716674	0.260345003948626
Glrx5	2021.34037964546	0.313128834202225	0.14757834741626	2.12178032675086	0.0338561894558916	0.260534495162941
Nek2	409.250970741358	0.363369009315052	0.171319533019456	2.12100163309332	0.0339216649939783	0.260685357891519
Tmem50a	1730.60571486198	-0.249605996851563	0.117673764019624	-2.12116948013949	0.0339075426276159	0.260685357891519
Gpr153	11.3404823339531	-1.98945557649174	0.938189455533399	-2.12052647230048	0.0339616714905276	0.26081645821712
Ring1	1099.42681868481	0.253762445051402	0.119820069804421	2.11786260403296	0.0341867054936021	0.262367383281756
Acbd3	890.318097570995	-0.231490703052549	0.109426833081414	-2.11548389489001	0.0343887261371569	0.262433717546811
Chst1	55.2543368266513	-1.29440734256869	0.612626723388117	-2.1128809651169	0.0346109574698812	0.262433717546811
Dclre1b	279.661394773718	-0.454714070429626	0.214908867415927	-2.11584601369467	0.0343579061961859	0.262433717546811
Ddx20	370.606172685966	-0.275323412256812	0.130128938492165	-2.11577390430637	0.0343640415454414	0.262433717546811
Fbxo11	1008.64241108172	-0.311083087162571	0.147187158368382	-2.11352057211396	0.0345562361232316	0.262433717546811
Itgal	50.7038983891598	-1.22703296949442	0.580531440276976	-2.11363740938645	0.0345462481398605	0.262433717546811
Klhl25	428.195359848792	0.502583817899272	0.237839575115536	2.11312107186169	0.0345904065521575	0.262433717546811
Maged2	88.2204878905746	-0.484450507587741	0.228918523012096	-2.11625735311137	0.0343229257224163	0.262433717546811
Mamdc4	51.8382451354607	-0.754207970839251	0.356679716617846	-2.11452441980973	0.034470501321796	0.262433717546811
Mark1	289.954404869646	0.399426413475965	0.188799984773949	2.11560617419647	0.0343783163016797	0.262433717546811
Plekhg1	74.8249323510718	-0.798391361294777	0.377506804247917	-2.11490588331345	0.0344379696619849	0.262433717546811
Prrg4	147.384320130222	0.717639017674437	0.339000393682696	2.11692679727725	0.0342660609821053	0.262433717546811
Rab43	117.932057339596	0.52397687780221	0.247777710287571	2.11470546399869	0.0344550583868277	0.262433717546811
RGD1565685	286.438158238689	-0.32149707097065	0.152064604389156	-2.11421370714181	0.0344970186650356	0.262433717546811
Slc2a8	211.744659622613	0.440122897216328	0.207889121622095	2.11710403017812	0.034251019735325	0.262433717546811
Slc3a2	1104.31721346701	0.309395135763149	0.146342476537995	2.11418545785523	0.0344994304253785	0.262433717546811
Snx12	255.476826772837	0.391489838313247	0.1851905585559	2.11398378711123	0.0345166520897039	0.262433717546811
Tbc1d9	362.216570203481	-0.490511447159478	0.232152852937607	-2.11288140960864	0.0346109194158642	0.262433717546811
Gcsh	4652.09605412439	0.347541963205306	0.164547906020016	2.11210201096712	0.0346777005603082	0.262589436754472
Tlr6	292.915219597764	-0.318942212752179	0.150989597429381	-2.11234560646703	0.0346568167755439	0.262589436754472
Mum1	250.323311442406	-0.339769552508601	0.160998440523093	-2.11039033300367	0.0348247484484803	0.263527357433706
Bmpr2	297.732440258354	-0.414979502799696	0.196702349898133	-2.10968248734499	0.0348857139385191	0.263813056969533
G3bp2	656.279268880119	0.458587769019441	0.217525136465671	2.10820586747138	0.0350131859820875	0.26459858954141
Gtf2f1	1220.41613510495	0.220037815171328	0.104385219196434	2.10794034696863	0.035036149679731	0.26459858954141
Ptprcap	22.7606589384573	-1.21428150630146	0.576721619257415	-2.10548983383867	0.0352486913350056	0.265850448383327
Zbtb7c	58.4377615110584	0.595874913335377	0.282975420301992	2.10574795754154	0.0352262516256514	0.265850448383327
Laptm5	262.871877633095	-1.48811908050957	0.707554404537713	-2.10318679520036	0.0354494441170689	0.267187255858491
RGD1309104	80.6257412608499	-1.19141798379608	0.566690104403845	-2.10241536694813	0.0355169062339298	0.267518327538003
Exosc8	225.024417894073	-0.354534557118017	0.16875229601127	-2.10091693860177	0.0356482582318754	0.268329869578474
Rftn1	93.3711692300953	-0.932044857601654	0.443718030135835	-2.10053411017877	0.0356818832948531	0.26840521874871
Aldh2	21479.7677746983	0.360142152509662	0.171635916875887	2.09829130793228	0.0358794198913636	0.269179880916185
Dmtf1	664.589962984241	-0.279390101432163	0.133120294248249	-2.09877917570662	0.0358363713643124	0.269179880916185
Ocln	1126.45673696012	-0.273758064353438	0.130467265276856	-2.09828928177896	0.0358795987672023	0.269179880916185
Pltp	335.519505110566	1.43661551376954	0.684607041033081	2.09845272932289	0.0358651714949857	0.269179880916185
Baz2a	732.99350166263	-0.292690798158626	0.139529718285208	-2.09769504128393	0.0359320932069112	0.269395891418043
Slc16a6	41.4564065284369	0.765837231049116	0.365224791684389	2.09689278626769	0.0360030672621298	0.269750074160427
Abcc2	250.415312698986	0.556675742888937	0.265694488525542	2.09517233864421	0.0361556752704422	0.270715023138239
Miox	62315.3652466514	0.400264009792714	0.1910871874912	2.0946669164365	0.0362006121440117	0.270873046496931
Mboat2	355.479299237491	-0.558522018811768	0.266729846377703	-2.09396146099403	0.0362634134813827	0.270986165436815
Sema4f	92.2921746316629	-0.958938061566975	0.457910037525408	-2.09416257120978	0.0362455007110612	0.270986165436815
Dym	650.194237114415	0.223228377327385	0.106765942170068	2.09082009478083	0.0365441940688096	0.272666454849198
Ralgapa1	1001.46107891314	-0.21928424015555	0.10487832558202	-2.09084421341242	0.0365420312686848	0.272666454849198
Usp40	902.632667234715	-0.252069169652242	0.120570267608374	-2.09064120576553	0.0365602390630106	0.272666454849198
Leng8	3651.51057756552	-0.327778339992272	0.156832036323654	-2.08999607271461	0.0366181524910245	0.272740446404315
Timp4	34.8944222427707	0.778633631492444	0.372537082057014	2.09008356213323	0.036610294010382	0.272740446404315
Zcchc7	446.961292694008	-0.25622312871514	0.122631361179108	-2.08937686291287	0.0366738122867525	0.272976129961512
Cdc37	2592.7252726605	0.17638253292245	0.0844348415611491	2.08897807660018	0.0367096967038035	0.273064406240465
Ankrd12	483.958834246782	-0.332981641358618	0.159517921849845	-2.08742464481235	0.0368497661498758	0.273431671872814
Caskin2	1399.77168270441	-0.269617396608604	0.129142868755831	-2.0877451399842	0.0368208306117658	0.273431671872814
Lhpp	2109.313448834	0.305382262837989	0.146300478303652	2.08736339333189	0.0368552983731437	0.273431671872814
Ptdss2	1086.77247792094	0.224486544458547	0.107526775435552	2.08772692707684	0.0368224744243869	0.273431671872814
Lpcat3	1236.71427963413	0.311367650711662	0.149224553465995	2.08657116727521	0.0369269158616319	0.273784295944754
Bpgm	982.021988247341	0.25772162191036	0.123674747650157	2.08386616352261	0.0371723429241535	0.274049714193963
Camk2n1	715.79825839572	0.340577276835765	0.163441648624117	2.08378512883839	0.0371797166362037	0.274049714193963
Cml1	4142.67054993873	-0.620678024043236	0.297786034641613	-2.08430870436965	0.0371320960947428	0.274049714193963
Ephx1	1916.73960963173	0.256002672779089	0.122832016755459	2.08416892876353	0.037144803961751	0.274049714193963
Gpx1	7391.48039860983	0.460759771187056	0.221013694401949	2.0847566592371	0.0370913946776597	0.274049714193963
Kat2a	568.593578818944	-0.308090232069972	0.147779302026062	-2.08479961568392	0.0370874936288435	0.274049714193963
Kdelr3	125.90355969152	-0.528541104994221	0.253575251780667	-2.08435602955208	0.0371277943078552	0.274049714193963
Samd8	222.796052011402	0.359323266107607	0.172287338181507	2.08560460623668	0.0370144537660615	0.274049714193963
Spc25	49.5108722348022	0.846562657146874	0.406209929548492	2.08405210105977	0.0371554283332722	0.274049714193963
Tspan1	816.015848561849	-0.541192538304362	0.25976552734383	-2.08338859985848	0.037215816557541	0.274138023974732
Gna14	56.4827276719732	-0.844790500019432	0.405548914760279	-2.08307917805375	0.0372440069817305	0.274167994400485
Kank3	436.796097419801	-1.22229986720786	0.587023791655393	-2.08219817421881	0.0373243719856249	0.274581755332435
Nr4a1	1497.79356792677	-1.28570560331214	0.617565643896955	-2.08189301982393	0.0373522425080811	0.274609048089813
Cldn5	427.633288887573	0.680535481024166	0.326987103559245	2.08123034094176	0.0374128276527749	0.274677253319364
Il17rd	14.4399161561305	-1.40616487805228	0.675887756212867	-2.08047100295366	0.0374823526217081	0.274677253319364
Letm2	91.330425209905	-0.469534226895371	0.225680576106116	-2.08052564822678	0.0374773456327721	0.274677253319364
Mapk6	1972.87005456315	0.303523160652412	0.1458865510496	2.0805424384131	0.037475807310694	0.274677253319364
Srpr	2706.17622528792	0.200030942912685	0.0961356958433198	2.08071456869352	0.0374600397800827	0.274677253319364
Fzd1	1852.93253219861	0.44406180465805	0.213541453764884	2.07951101216617	0.0375704067013728	0.27491322806143
Limch1	427.652135681878	0.522057910384143	0.251070366764314	2.07932906265362	0.0375871156438028	0.27491322806143
Ngef	339.813251696129	0.333567269944832	0.160392309136749	2.07969616336427	0.0375534102277385	0.27491322806143
Trrap	1162.08473302729	-0.224105593394291	0.107862025463442	-2.07770614756579	0.0377364324914558	0.275827840320184
Dsn1	27.2497820211023	-0.793526181236066	0.382311171692435	-2.0756029119506	0.037930691904602	0.276891614764102
Rbp2	20.0533162635959	-1.14420638284538	0.55120066914692	-2.07584360268691	0.0379084181738158	0.276891614764102
Camsap2	751.39442737204	-0.231769932095401	0.111708530335262	-2.07477380106791	0.0380075037822843	0.27727425416524
Cdc42se2	1146.98996941505	0.296280194448533	0.142826277787874	2.07440954870062	0.0380412912616537	0.277342730262961
Bok	1237.30177825246	-0.232334165233062	0.112049386124428	-2.07349788578994	0.0381259676722328	0.277603938861369
Hipk1	1964.73891784177	-0.403514006096291	0.194588638121049	-2.0736771169819	0.0381093078055165	0.277603938861369
Ahsa2	1192.7161656244	-0.413483771332135	0.199525807402039	-2.07233227979866	0.038234464046845	0.278088545477737
Ifitm1	215.928410810996	-1.46878239248842	0.708783810818312	-2.07225725259253	0.0382414566762012	0.278088545477737
Ssna1	310.448091863806	0.314955650063198	0.152009610140029	2.07194564720654	0.0382705103241469	0.278121880015507
Mtpn	622.973822104542	0.346830244018454	0.167453966373355	2.07119754479367	0.0383403389591782	0.278320010505418
St3gal3	260.513736192231	0.404851490359833	0.195473822155799	2.0711289414352	0.0383467478841707	0.278320010505418
Mbl1	664.277940045454	0.397436845432774	0.19194426239914	2.07058466069864	0.038397626866441	0.278475022578193
Pear1	272.679169919775	-1.34034513258711	0.647391995692492	-2.07037643576886	0.0384171067572239	0.278475022578193
Azi2	575.654646810362	-0.230990540531497	0.111623783839486	-2.06936669396246	0.0385116894567713	0.278982703865942
Ankef1	28.4268491113033	1.74826378699428	0.846673609409454	2.06486155652551	0.0389361014599551	0.28018850657354
Cd4	94.5718262693064	-2.06617892372008	1.00015881617511	-2.06585083319241	0.0388425665522287	0.28018850657354
Copb2	891.585325622487	0.280459646664303	0.135728216865055	2.06633265463985	0.0387970800905455	0.28018850657354

Lrrn2	36.1048398934602	-1.15798137604614	0.56039426676376	-2.06636906321937	0.0387936447702721	0.28018850657354
Mtdh	595.789483298314	0.368916766041468	0.178656024695183	2.06495564127155	0.0389271976362219	0.28018850657354
Prkx	293.448448341646	-0.285721671720159	0.138208246835388	-2.06732722729972	0.038703330346272	0.28018850657354
Ptpn13	2586.73487617694	-0.223710924845854	0.108310161940709	-2.06546570365501	0.0388789573656793	0.28018850657354
S100a11	1253.3807665043	-0.350144741087214	0.169584445396307	-2.06472203431718	0.0389493084978175	0.28018850657354
Slc25a16	947.016501332852	0.285852273749428	0.138330137566855	2.06644971788072	0.0387860355449924	0.28018850657354
Vom2r52	270.205816384768	-0.380094696127415	0.18404666935843	-2.06520822926267	0.0389033022206362	0.28018850657354
Wdr72	405.030765561565	0.331050624313954	0.160261489667189	2.06569042258023	0.038857720201232	0.28018850657354
Slc5a1	470.321764025034	0.37394684409838	0.181162014515207	2.06415702043869	0.0390028310822732	0.280396064567436
Kcng2	11.8921132362299	1.57189311491688	0.762534787755784	2.06140511902823	0.0392644069005041	0.282098134532952
Crat	3722.0928167687	0.273217608047152	0.132592081909161	2.06058766189624	0.0393423948359145	0.282479886105499
Poll	523.255775707839	0.3298643057427	0.160170648323073	2.05945539458232	0.0394506338468528	0.283078222413717
Ncaph2	731.685753821721	0.205202007941577	0.0996555586922142	2.059112513486	0.0394834613669401	0.283135029587786
Arid1a	1736.83334143372	-0.214938350256778	0.104493860900813	-2.05694715846321	0.039691309043332	0.283552117276249
Atp6v1g3	2184.20178331297	0.283533362258656	0.137784907656044	2.05779694657449	0.0396096293106627	0.283552117276249
Gatsl3	737.46513030409	0.27197803136378	0.132179367965156	2.05764360619031	0.0396243574877268	0.283552117276249
Gjd2	59.3384364221482	-1.39207563402415	0.676361868940463	-2.05818171891457	0.0395726928020438	0.283552117276249
Notch4	352.772977542421	-0.546646136114027	0.26572015139228	-2.05722499121648	0.0396645886672413	0.283552117276249
Rrn3	1196.71732434776	0.212604061090747	0.103358147066826	2.0569647108059	0.0396896205067658	0.283552117276249
Coq4	210.670926687478	0.383708524314513	0.186577701057058	2.05656154053034	0.0397284209001129	0.283638964793143
Kdm3a	663.397923661505	-0.298470396290703	0.145162456905993	-2.05611287279322	0.0397716377268875	0.283769262023731
Lmna	1828.06659875266	0.206284949553168	0.10038796752707	2.05487723912274	0.0398908633989349	0.284441376030298
Dhcr7	1877.20348067584	0.346889118295745	0.168906825723254	2.05373061041421	0.040001772158148	0.284874775908214
RGD1304595	481.951434464642	-0.281487661668807	0.137045076832805	-2.05397864829702	0.0399777583087034	0.284874775908214
Fbxl17	335.588031114316	0.324017219386637	0.157824427824099	2.05302324775582	0.0400703228704004	0.285184276609249
Kbtbd11	1136.29157865966	0.395014810912777	0.192478516349725	2.05225403023708	0.0401449810301042	0.285358257903792
Tyms	114.32639063357	0.624288140734863	0.304169456301996	2.0524353376068	0.0401273732110617	0.285358257903792
Cd300lf	52.2270559242783	-0.867765550780733	0.423234493785721	-2.05031859057328	0.0403333516077784	0.286043256241871
Giot1	228.627985468123	-0.362635052221901	0.176892675149871	-2.05002865107139	0.0403616350062999	0.286043256241871
Prkcz	855.434485731843	0.286433764541934	0.13972570866354	2.04997181464771	0.0403671813313356	0.286043256241871
Srgap3	101.733946227911	-0.955761673782362	0.466064589580746	-2.0507064796365	0.0402955395810912	0.286043256241871
Xpr1	175.633588446288	0.499574218971349	0.243586341990683	2.05091227565812	0.0402754904745105	0.286043256241871
Cacnb3	103.180254782931	-0.845942327234021	0.412829528892414	-2.049132312564	0.0404491785943816	0.286388833879847
Fktn	252.075580293085	0.621562653243672	0.303393814187957	2.04869916318928	0.0404915410914054	0.286388833879847
Pstpip1	59.8002865658274	-0.860139921911671	0.41981128772612	-2.04887278417539	0.0404745562485255	0.286388833879847
Ddx24	1262.48781415161	0.25002657559364	0.122116837617343	2.04743735976121	0.0406151613364156	0.287073679220991
Fgd3	253.834354041853	-0.447369773273383	0.218528104373585	-2.04719559781923	0.0406388835004905	0.287073679220991
Armc1	712.448292681659	0.324479873686252	0.158592828969456	2.04599335162086	0.0407570247910094	0.287729406071188
LOC498350	35.782382574977	-1.02373188874165	0.500599747356961	-2.04501079784138	0.0408537935772612	0.288233530601583
Echdc2	1606.99335141255	0.428443583003705	0.20965730146559	2.04354239040906	0.040998775526903	0.288372530058369
Exoc3l4	134.386539771693	-0.686069829168113	0.335599850356132	-2.04430910335648	0.0409230204871608	0.288372530058369
Gpatc2	87.7155077364362	0.508816035206078	0.248918916189415	2.04410352975703	0.0409433205308575	0.288372530058369
Ppp1r12c	949.445648893863	-0.338595776678071	0.165691899016461	-2.04352644086982	0.0410003526811828	0.288372530058369
Tifab	16.7509623635368	-1.24379039533557	0.608636715131301	-2.04356780393579	0.0409962626418655	0.288372530058369
Agtr1a	264.428531985106	-0.79301706462042	0.388287048271312	-2.04234745441807	0.0411170780095827	0.28901466212549
LOC100151767	454.980590240229	-0.227169769640137	0.111265165561034	-2.04169713400125	0.0411815832436328	0.289281765137108
Tnc	77.6729794358737	-2.40543735595931	1.17829763998926	-2.04145139082282	0.041205980798608	0.289281765137108
Abcc3	296.530099572113	-0.289262692911534	0.141883777212241	-2.0387298575991	0.0414769961404816	0.290299953824442
Acot5	22.4458949266471	1.60058539913122	0.784717876867415	2.0396953431477	0.0413806790389302	0.290299953824442
Ccl19	57.3673409644095	-0.959607559092041	0.470561986423747	-2.03927981175237	0.0414221093205111	0.290299953824442
Esrp1	154.051251620496	-0.519569213662337	0.254851622296063	-2.0387126006157	0.0414787194273178	0.290299953824442
Ms4a7	6.32684408445753	-2.97029066577885	1.45680650102876	-2.0389054165267	0.0414594682258972	0.290299953824442
Dnmt3b	33.6416317606641	-0.814942757926214	0.399791341577388	-2.03842022868938	0.041507924980164	0.290325584815104
Zfp281	89.7502843891954	-0.485772388778203	0.23837725491087	-2.03783028275845	0.0415669087491599	0.290559338771803
Elovl5	386.946035971823	0.369127231610205	0.181213556634836	2.03697360432053	0.0416526872184074	0.290666058147278
Gtpbp2	787.503721246898	-0.355292745046537	0.174416922105839	-2.03703138867993	0.0416468966109397	0.290666058147278
Ksr1	119.285843371293	-0.614650633662363	0.301805343602613	-2.03657969181511	0.0416921796057503	0.290666058147278
Lrpap1	11662.7739744471	0.168706925632418	0.0828456054430518	2.03640162601488	0.0417100423049631	0.290666058147278
Rara	1070.07481306945	-0.297100800244935	0.145860744245451	-2.03687977722766	0.0416620911431463	0.290666058147278
Abcg3l4	408.010978818158	-0.629665080299138	0.309322366823363	-2.03562738370842	0.0417877858927885	0.291029396113624
Mmp13	4.92739887097764	-8.26198749602185	4.05950076430864	-2.03522254969422	0.0418284850918386	0.291134452880489
Fgd1	112.483079601686	0.440226467164234	0.216385671513563	2.03445294729989	0.0419059480919144	0.29130489148529
Ifi47	108.689065584774	-0.620525506785211	0.305018530366848	-2.03438625856239	0.0419126662442936	0.29130489148529
Rnf222	60.8008380614719	0.567113354478308	0.278821031300916	2.03396907267822	0.0419547137963785	0.29130489148529
Tnfsf10	77.5680017715476	-0.609893787933953	0.29985514849011	-2.03396136769707	0.0419554907057382	0.29130489148529
Dnajc16	358.245193841161	0.355721774750223	0.174959912252032	2.0331615978282	0.0420361994239015	0.291687083426169
Nptn	2574.16637470223	-0.17647660257323	0.0868287133090662	-2.03246824521127	0.0421062753388767	0.291817027744922
Rgcc	361.895164313197	-1.18527269397218	0.583107141208938	-2.03268423623622	0.0420844349179922	0.291817027744922
Prss12	198.67095790843	0.517039474192561	0.254454077602272	2.03195593902302	0.0421581166922337	0.291998265889048
Anxa2	1985.2913013846	-0.436579625711637	0.214884943065275	-2.03169016629992	0.0421850320681045	0.292006744510399
Lpxn	125.396858343373	-0.494181057517834	0.243410814048346	-2.03023460337994	0.0423326980450032	0.29285054533141
Adam19	63.5873609141605	-1.47815009586464	0.728331280907664	-2.02950241821624	0.0424071429312242	0.293187096445434
Sh2d3c	246.248525371269	-0.835143799120372	0.411656188184661	-2.02874102974918	0.0424846744799685	0.293544565434239
Celf3	24.9844838985755	-1.60996031768977	0.794108535172739	-2.02738069971703	0.0426234941942678	0.294324808634294
Cpg1	372.014935958367	-0.249384010031074	0.123077284973062	-2.02623912353654	0.0427402862215042	0.294952090585074
Acads	3095.91250036497	0.254612259967226	0.125753624923699	2.02469121762264	0.0428990809975194	0.295868297704979
Adnp	2104.99275967123	-0.192599283563957	0.0951412777537492	-2.02435039880855	0.0429341114379477	0.295930327837303
Htra1	802.809262222853	0.250423633913466	0.123751341085576	2.02360339465165	0.0430109754843387	0.296280452942421
Limk2	556.895192944176	0.318313440046555	0.15732248703281	2.0233181285786	0.0430403590129305	0.296303283186534
Ephx2	2741.70715712497	0.399260765048611	0.197372896552355	2.02287533913099	0.0430860016747258	0.296437950989669
Gaa	1173.2676879556	0.292253512759416	0.144495523654435	2.02257831500959	0.043116641789863	0.296469298598659
Mier3	312.502744558421	-0.500011677609178	0.247286485202272	-2.02199354809134	0.0431770184032509	0.296704952340598
Emp3	68.5303588746058	-0.806036764581199	0.398709006237405	-2.02161664765922	0.0432159708576927	0.29679318717132
Pcgf5	182.809588137894	0.358104658425128	0.17718670640315	2.02105827064892	0.0432737333998182	0.296948204204166
Prkd2	191.462273020975	-0.524394497454718	0.259583623505229	-2.02013705785317	0.0433691728821851	0.296948204204166
Prkd3	386.550419120153	-0.375953342587825	0.186079724180925	-2.02038854175367	0.0433431010178272	0.296948204204166
Rbm39	1685.03578443882	-0.218977992502179	0.108393606629224	-2.02021133267781	0.0433614712766454	0.296948204204166
Vamp1	196.207225524313	-0.56124605509469	0.277775599122303	-2.02050164545798	0.0433313796369917	0.296948204204166
Erlin1	1546.4993124247	0.373198113130899	0.184767551740054	2.01982496177654	0.0434015469620413	0.296990958922674
RGD1305347	2174.34869659854	0.189943373741946	0.094109266653204	2.01832806159136	0.0435571060521621	0.297876093495111
Ado	47.7088025644596	0.661062996993076	0.327627151080035	2.01772958930253	0.0436194314333756	0.297943784658502
Hck	35.4507157794343	-0.867348592297549	0.429823557500855	-2.0179177645372	0.0435998266010257	0.297943784658502
Rnf10	3160.49658374864	0.196313638273696	0.0973075141021709	2.01745610382741	0.0436479374941294	0.297959433968934
Chrd	1769.1463326556	-0.373416942350696	0.185148544470334	-2.01685054246013	0.0437111126321071	0.298211588341254
Atic	1057.8886052068	0.219431353832414	0.108845661175437	2.01598622731259	0.0438014159901836	0.298648406805295
Lfng	91.8332919345601	-1.14508951858363	0.568157448217172	-2.01544399739337	0.0438581483685337	0.298803806101309
Tmem45b	485.424717850643	-0.55665463187224	0.276218937909789	-2.01526599184173	0.0438767862381739	0.298803806101309
Dynll1	1577.88163259411	-0.307700855558977	0.152716433263742	-2.01485098219637	0.04392026532579	0.298920799816126
Ap3b1	1184.17855831366	0.271675864622631	0.134877583918387	2.01424029649745	0.0439843108438864	0.299177544614969
Gnl3l	204.273883335658	0.514589930192974	0.255561018512658	2.01356972666583	0.0440547275017562	0.299254516563613
Josd2	437.637636178534	-0.311718185068764	0.154787926537329	-2.0138404334371	0.0440262890890256	0.299254516563613
Mpp3	18.6709085387219	1.17554460070571	0.583866085796932	2.0133805153303	0.0440746138243434	0.299254516563613
Fhdc1	197.031957152572	-0.518442322109738	0.257571302331469	-2.01281088932242	0.044134527905439	0.299303725640346
Slc25a11	3260.58981712154	0.227868581897649	0.113197804566052	2.01301237927001	0.0441133270502275	0.299303725640346
Yy1	346.538118794828	0.276165997706376	0.137243064357929	2.01224010115467	0.0441946331685589	0.299532618123936

Casp4	79.3377573312904	-0.593384899991174	0.29494693245625	-2.01183614641997	0.044237212237416	0.29964252341506
Pla2g4b	262.99333625505	-0.312806209158943	0.155502915192648	-2.01157778149314	0.0442644634830673	0.299648536002706
Ctla2a	151.081299179219	-0.567127339657793	0.281981762458016	-2.01121992682854	0.0443022318888594	0.299720485725164
Fam180a	41.3594781395061	-1.28303526048676	0.638094910052779	-2.0107279344708	0.0443542017411338	0.299720485725164
Kctd18	426.807233374862	-0.282983788730382	0.14073303215195	-2.01078442213086	0.0443482322561772	0.299720485725164
Zbtb1	255.419368865416	-0.348192428513804	0.173193347655361	-2.01042611178505	0.0443861091548477	0.299757882741532
Pik3r5	14.9537490078912	-1.21892826857886	0.606805616785302	-2.00876233650642	0.0445623442881908	0.300718878825931
Spi1	49.3810354066523	-0.918452257383951	0.457263666983149	-2.00858350160105	0.0445813224372421	0.300718878825931
Got2	6626.14986855111	0.241774743017374	0.120397873577235	2.00813133848479	0.0446293368821539	0.300864201069135
Fxyd5	80.9169783766054	-0.844613497910976	0.420705550282578	-2.00761196837947	0.0446845417499704	0.300879444034457
Trap1	2041.53018675004	0.207572307945958	0.10338251983061	2.00780855686301	0.0446636392026947	0.300879444034457
Mtch1	3121.79977575318	0.231908714781442	0.115554723980089	2.00691678188251	0.0447585246305586	0.301199165749514
Foxc2	46.3564520432578	1.01586865331371	0.506486577016141	2.00571683320511	0.0448864682033007	0.301385130572523
Sort1	2207.18980934129	-0.456404372068033	0.227557865914663	-2.00566291230376	0.0448922247104771	0.301385130572523
Sytl4	23.6288841449411	-0.965849134293603	0.481417843076539	-2.00625952731886	0.0448285657225229	0.301385130572523
Uck2	49.4258763512559	0.647635192783802	0.322873172628022	2.00585012223959	0.0448722411554411	0.301385130572523
Adra2b	3131.60731771053	0.393131642540706	0.196072802047332	2.00502894045347	0.044959953213375	0.301547417607734
Crip2	1210.74029310542	-0.527811997786754	0.263288331255995	-2.0046919484387	0.0449959898172371	0.301547417607734
Selplg	24.6158329585111	-1.44351545124517	0.720054426741769	-2.00473102814887	0.0449918095382707	0.301547417607734
Mpzl1	664.121394854243	-0.448346279235495	0.223974650673232	-2.00177242329807	0.0453092131255095	0.30346759951947
Rpl38	4116.72557889477	-0.293045851830326	0.146499991331931	-2.00031310013091	0.045466465322986	0.304341486961754
Mthfd1	4087.93312154714	0.187914686035442	0.0939696782717523	1.99973746309963	0.0455286205820507	0.304399000903287
Slc9a3r1	10502.2913473715	0.235785393608503	0.117895023499813	1.9999605293677	0.0455045261797638	0.304399000903287
Dock11	115.085792090188	-0.742993668058009	0.371732313729259	-1.998733068439	0.0456372430486284	0.30494585801923
Hspb7	54.2145824517183	-1.05275308679488	0.526829737552401	-1.99827954224048	0.045686362149762	0.305094707517154
Dclk1	53.8700683847886	-0.689488477971783	0.345098692954325	-1.99794578202891	0.0457225384553102	0.305157000635969
Csk	666.559088728783	-0.204005091488425	0.102221541583715	-1.9957152702628	0.0459649239764657	0.306594674833632
Ippk	68.72671156028	0.658888204238708	0.330246944824647	1.99513792501112	0.0460278390312321	0.306834263008202
Abcb6	1464.68845198516	0.197968719410791	0.0992524567774995	1.99459767383482	0.0460867775053193	0.307013407218057
Parp14	680.624537890441	-0.380248794727936	0.190658570340849	-1.99439654901507	0.0461087353617124	0.307013407218057
Phldb1	1473.79501324596	-0.249343985465163	0.125087481300953	-1.99335683212981	0.0462223872746679	0.307589961220067
Ddo	251.986627657542	0.418716934278332	0.210122333895541	1.9927293139933	0.046291095637667	0.307796908089864
Mipep	1214.64772678869	0.316006631534026	0.15859182872227	1.99257826888058	0.046307646738841	0.307796908089864
Opa1	1551.44600463516	0.204951535744506	0.102889287833634	1.99196184617295	0.0463752442824468	0.30788611361816
Sec61a2	354.695573386408	-0.2871274220963	0.14412528470664	-1.9922071458921	0.0463483345198799	0.30788611361816
Alas2	95.7608635656218	0.723482500777445	0.36333439348056	1.99123043058723	0.0464555598074021	0.308059447357603
Dnaaf1	28.6979882994361	0.946015636584277	0.475055469968315	1.99137931544578	0.0464392015248485	0.308059447357603
Rfx7	392.573184193042	-0.280136862144108	0.140796489433404	-1.98965800405564	0.0466286218448303	0.309026773112735
Cdc20	210.715071211534	0.408743073913886	0.205567456301003	1.98836470163537	0.0467713697272617	0.30943154151342
Gnai1	340.19020866427	0.427366707577889	0.214921150007802	1.98848139218674	0.0467584749661543	0.30943154151342
Kbtbd2	545.113272001908	-0.29764608744025	0.149659867101306	-1.98881699686912	0.0467214060129455	0.30943154151342
Axin2	74.3769169948632	-0.814853333943345	0.409956356247266	-1.9876587386094	0.0468494452246631	0.309767768716417
Pgm1	2086.10572756505	0.272434290454141	0.13708910719106	1.9872789022869	0.0468914983176465	0.309865563882773
Gosr1	408.376593005907	0.272698678131945	0.137284307201435	1.98637909671511	0.0469912458900641	0.310116614228008
Klf11	280.754382271268	-0.352400616951362	0.177424716686804	-1.98619799728034	0.0470113431563309	0.310116614228008
Usp28	306.059288366228	-0.340597222166573	0.17146678909541	-1.98637429419089	0.0469917787504215	0.310116614228008
Bhmt2	10756.2006793815	0.284081508054218	0.143100955878458	1.98518246304029	0.0471241747094997	0.310675759944403
Hnf1a	1515.50298447461	0.335788882385578	0.169167991583788	1.98494336453279	0.0471507729987766	0.310675759944403
Nubp2	1184.27992346875	0.202403120797988	0.102005350093678	1.98424024438041	0.0472290641185492	0.310831234957401
Tars2	1075.64909456911	0.192454028664201	0.0969804366580725	1.98446238536483	0.0472043173274457	0.310831234957401
Tomm34	643.889386876649	-0.200360812615341	0.10099836065126	-1.98380262138287	0.0472778478044302	0.31097223272289
Suv420h1	819.202378956093	-0.230225514495422	0.116119298890376	-1.9826636631071	0.0474050110028045	0.311405765682824
Tgfbrap1	832.5011680924	0.24819050246105	0.125171820494323	1.98279853629121	0.0473899375922276	0.311405765682824
Xbp1	1132.11736122362	-0.23778767320272	0.119944772294071	-1.98247634019207	0.0474259528767348	0.311405765682824
Hadhb	4523.79595928948	0.255330108425663	0.12883349546632	1.98186121940945	0.047494775381854	0.311677607961982
Tmem136	28.6053008166424	-1.00687218309088	0.508195814553153	-1.98126815345026	0.0475612097828403	0.311933473971012
Nt5dc2	245.757931734581	-0.470384482370685	0.237490128809372	-1.9806485630746	0.0476306988380255	0.312209067470011
Pitpnm1	1155.38892587348	-0.199199846914546	0.100701993112468	-1.97811225734203	0.0479160440994363	0.313898419155154
Hsph1	2210.53457269429	-0.36763222601614	0.186119337512065	-1.97525002469079	0.0482397815079627	0.315462163602779
Kcnab1	35.9485381800568	0.969224216209831	0.490683960751745	1.97525147291333	0.0482396172409656	0.315462163602779
Nek6	234.026637285427	0.516718640654402	0.261626911507439	1.97502098571274	0.04826576654102	0.315462163602779
Prkch	224.678581815369	-0.587006346905471	0.297178611694437	-1.97526444974795	0.0482381453433612	0.315462163602779
Npr2	195.447631938596	-0.431786374920439	0.218685060671784	-1.97446672211638	0.0483286977632545	0.315691941825949
Gpr37	44.0235796221592	-0.988714078614341	0.500930915982335	-1.97375336013241	0.0484097944790481	0.315827708060703
Taf15	1122.47106246498	-0.207369062606336	0.10506482238321	-1.97372496238546	0.0484130251676964	0.315827708060703
Zbtb11	412.460034712373	-0.332567761089519	0.168512389725558	-1.97355079725084	0.0484328431418093	0.315827708060703
Cyp4f17	361.279095302376	-0.344897944353495	0.174803263269805	-1.97306353383776	0.0484883242714975	0.316008195911606
Xpnpep3	72.0121580196476	0.564968320546939	0.286447425321765	1.97232815031349	0.0485721580855172	0.316373151174778
Pip5k1a	606.046843437873	-0.21913286265111	0.111170685573832	-1.97113889799282	0.0487079905651495	0.317076186004289
Gys2	19.6841363175155	1.97538663064458	1.00277753029025	1.96991513169708	0.0488480979242059	0.317627956907771
Masp2	80.4384657910782	-0.528768671485369	0.268454104224106	-1.96967996825242	0.048875060187743	0.317627956907771
Zfp317	368.99308326412	-0.302331047717375	0.153493510196607	-1.96966664799134	0.0488765877733319	0.317627956907771
Cmtm3	61.7204407760051	-1.0920998801627	0.554747628216845	-1.96864272078656	0.0489941331450731	0.318209895615372
Cdkn3	38.3916876384749	0.806683924952862	0.409974006809832	1.96764651308015	0.0491087239913895	0.318705724551868
Gns	519.680413395685	0.510594257661864	0.25951533405689	1.9674916687194	0.049126555464972	0.318705724551868
S100a9	20.9806083658832	1.7862949156631	0.908652001468288	1.96587352779351	0.0493132216531524	0.319734214095682
Aqp1	70738.9644550545	0.207323384881922	0.105525720900467	1.96467158066109	0.0494522612614839	0.320452908493744
Mtus1	1414.17291032046	-0.20415561117608	0.103952857401436	-1.96392495867323	0.0495387949572359	0.320830737027888
Crym	7490.0757333817	0.280823410967034	0.143118925832746	1.96216824108375	0.0497429001227834	0.321237387952021
LOC100365921	419.647183201335	0.329360351932263	0.167842837114015	1.96231401706186	0.0497259362734094	0.321237387952021
Pck1	44259.5286562513	0.756335976395694	0.385383613898583	1.96255354176717	0.0496980734849932	0.321237387952021
Smim3	185.060880376367	-0.787880300146203	0.401509737325831	-1.9622943776998	0.0497282214095131	0.321237387952021
Zcchc11	138.349705029426	-0.589176907681004	0.300199974221553	-1.96261478439096	0.0496909515193308	0.321237387952021
LOC686295	1083.83299297123	-0.221030333369172	0.112663627720678	-1.96186060968285	0.049778714903302	0.321286129239597
Cnnm2	392.578667537968	0.31653321933173	0.16138121859418	1.96140060218349	0.0498323098158111	0.321449508153524
Tcaim	1341.08669457675	0.391705838592255	0.199816532439276	1.96032747546199	0.0499575266277529	0.322074445633034
Enpp6	604.452423290508	-0.3573190629632	0.182379662822622	-1.95920453757347	0.0500888379541574	0.322647721887612
LOC291863	36.3796789492441	1.166077453048	0.595216355154904	1.95908167332621	0.0501032226932637	0.322647721887612
Cmtm4	110.784059961905	0.744157803059396	0.379984630542167	1.95838921694707	0.0501843590923424	0.322762417150837
Crispld2	410.114300653773	-1.37091502253437	0.70016635762229	-1.95798470979081	0.0502318068849174	0.322762417150837
F3	1459.32730020639	0.860587787712697	0.43952972825741	1.95797401719479	0.0502330616126388	0.322762417150837
Map7d1	532.383879132985	-0.341996233923119	0.174669609409007	-1.95796071841152	0.0502346222012872	0.322762417150837
Slc51a	357.265220352282	0.602732120575731	0.308026550233504	1.95675379320004	0.0503764220238281	0.323490628656966
Epn2	177.142329891659	-0.494967675618288	0.253026996622924	-1.95618523803576	0.0504433368490934	0.323554721572684
Mtfr1	323.100283526643	0.429611261510429	0.219614288359075	1.95620815348773	0.050440638426621	0.323554721572684
Abcb1b	385.182501043115	1.74449878376656	0.892325920258176	1.95500180389451	0.0505828573544849	0.323824217607405
Clk2	1870.91753539585	-0.278380031563393	0.142456620654848	-1.95413895320363	0.0506847864792868	0.323824217607405
Cryba4	13.703747467007	1.20423204323733	0.616129843067238	1.95451016825022	0.0506409135294072	0.323824217607405
Hk1	2055.01470532509	0.313177835230358	0.160245625344132	1.95436121615052	0.0506585139709304	0.323824217607405
Lman1	339.747051942883	0.446392422359025	0.228325394728087	1.95507128276569	0.0505746572458719	0.323824217607405
Psma3	752.908034775973	-0.242909456192265	0.124283966351564	-1.9544713877665	0.0506454954060901	0.323824217607405
Tmem5	349.338677126569	0.254431146001437	0.130117688774274	1.95539244816145	0.0505367667967738	0.323824217607405
Egr2	38.1911521544938	-0.674752147355085	0.345344313676837	-1.95385335919125	0.0507185617680328	0.323857962390709
Clk3	1045.28300823858	-0.284551404547156	0.145744525582925	-1.95239857832776	0.0508909017094039	0.324347333578722
Gabpb1	262.814213539524	-0.339948215785551	0.174167603495621	-1.95184528559066	0.0509565758919081	0.324347333578722

Prnp	390.719747203651	-0.610644493436765	0.312868254140885	-1.95176239632734	0.0509664207083933	0.324347333578722
Prpf38b	1347.60024288935	-0.295474473018208	0.151366793626136	-1.95204288827051	0.0509331129053381	0.324347333578722
Sla	25.3054155910398	-1.13590077218368	0.581742062698588	-1.95258490836035	0.0508688008583359	0.324347333578722
Ttpal	223.946800407054	-0.299582798117316	0.153487524788501	-1.95183809583304	0.0509574297614265	0.324347333578722
Zfr	1429.99338791506	-0.190240914959288	0.0975051571545697	-1.95108567086056	0.0510468554745653	0.324677425475047
Metrnl	381.986606296302	0.456803272014653	0.234251929055166	1.95005127111281	0.0511700083749582	0.325179014842454
Zw10	193.742862770721	0.326754764879404	0.167571461201434	1.94994280372491	0.0511829366138615	0.325179014842454
Marcks	325.616740439176	-1.13392239117141	0.581783030079817	-1.9490468654884	0.0512898282060843	0.32567608234098
Cacnb2	117.98770590621	-0.614575625533066	0.315460101368304	-1.94818813177119	0.0513924563844165	0.326001229260255
Id2	2619.54748372381	0.372559679092617	0.191238809954978	1.94813845150117	0.0513983989824369	0.326001229260255
Ly86	36.906759060075	-1.0741560796677	0.551463944143132	-1.94782649178766	0.0514357277717311	0.326056041189903
Slc25a24	171.378150237698	-0.585678147809548	0.300801742619596	-1.94705703068422	0.0515278977455278	0.326458241792458
Pgm2l1	71.1526455139509	-0.710445425588648	0.364999525397655	-1.94642835443317	0.0516033063629386	0.326753860791733
Adcy3	87.9885923900137	-1.12666652934353	0.578917656099598	-1.94616024830602	0.0516354933036046	0.326775621875707
Cxcl13	49.0586798067944	-1.47888770050094	0.760634324264139	-1.94428209893323	0.0518614421305544	0.327068265610156
Efemp2	141.914142919735	-0.688890122540323	0.354318598546818	-1.94426746257661	0.0518632061853987	0.327068265610156
Fam20c	5731.85384864365	0.25148752847332	0.129273850469003	1.94538591958797	0.0517285481209763	0.327068265610156
Pdia4	1287.83891345466	-0.307195965490717	0.158011355159203	-1.94413854106373	0.0518787466895265	0.327068265610156
Ppp2r5c	543.172774106906	0.209843373306849	0.107951715736409	1.94386325289385	0.0519119436178709	0.327068265610156
Rasal3	140.279021507042	-0.472228908200722	0.242920398761468	-1.94396563898456	0.0518995948309593	0.327068265610156
RGD1566243	39.1550953976319	-0.655625204278991	0.337149601571361	-1.94461212833503	0.0518216784829561	0.327068265610156
Samsn1	13.0198992928145	-1.45839227520688	0.750001984460992	-1.94451788851598	0.0518330303804741	0.327068265610156
Arhgap12	464.721117452924	-0.326795441330289	0.168229459032489	-1.94255776134415	0.0520696145609022	0.32760459594739
H2afj	647.627382709366	0.498078425625654	0.256411370277021	1.94249742157511	0.0520769117800143	0.32760459594739
Inpp5b	918.064179155074	-0.30457013845341	0.156816728735179	-1.94220438667451	0.052112362262263	0.32760459594739
Wdr47	283.831769422773	-0.281493483532404	0.144919634453368	-1.94241094103078	0.0520873718378994	0.32760459594739
Papd7	538.00741978105	-0.308481279740578	0.158853534639157	-1.94192266757744	0.0521464628203253	0.327637753684808
Arhgap25	49.2772204243245	-0.91571241505871	0.471897087437178	-1.94049177127124	0.0523199532545304	0.328546181597234
LOC308990	16.3075630771284	-1.22901043040148	0.633430361538362	-1.9402455345157	0.0523498570943367	0.328552443806864
Epb42	12.1021775928871	1.13033967934156	0.582655447311566	1.93997959610103	0.0523821696227198	0.328569184196238
Rbm43	101.554708445426	-0.449818844226703	0.231895523395857	-1.93974785558426	0.0524103405725655	0.328569184196238
Clic5	576.409896063415	1.06401702704123	0.548694936449808	1.93917777686392	0.0524796946634607	0.328641437765782
Ehbp1l1	117.1337716732	-0.75607000778546	0.38984730613251	-1.93940036494306	0.0524526061308515	0.328641437765782
Anxa7	770.287034363211	-0.292029680383702	0.150632084447909	-1.93869507584681	0.0525384786153858	0.328828385430878
Dao	33596.7689641679	0.302672871992365	0.156166241573495	1.93814533117211	0.0526054942659094	0.328984990045742
Gsn	1982.01743688901	0.239393990177535	0.123525353006078	1.93801502567457	0.0526213893984831	0.328984990045742
Slc52a3	639.310317858792	-0.223763930075771	0.1155890446313	-1.93585759610284	0.0528851444464353	0.330452199988006
Babam1	1232.61018554256	0.171718248559316	0.0887381031398125	1.9351129050929	0.0529764422302603	0.330840792521504
Gk	3396.82170654987	0.38005873124423	0.196489095934403	1.93424846013395	0.0530825867645031	0.331321626120452
Ivns1abp	5297.79349254786	-0.215231043577426	0.111300547395653	-1.93378243516017	0.0531398832920598	0.331497208286252
Lrrc49	81.7303959266386	-0.760192971434661	0.393239918255703	-1.93315311122699	0.0532173389795713	0.331798285705873
Psap	5341.58901742421	0.192265628913191	0.099512483113768	1.93207548336808	0.0533501898529214	0.33226205910592
Smpdl3a	616.080384689751	-0.357238989717917	0.184890759193911	-1.9321624903019	0.0533394532947102	0.33226205910592
Loxl3	31.8831872297502	-0.817244182544203	0.423765709706043	-1.92852834437951	0.0537894421585175	0.334814238539819
Pxmp4	1236.77990304643	-0.403445342563311	0.20924720054583	-1.92808000064473	0.0538451761590183	0.334977707839848
Msln	101.42793003116	-1.15701321679248	0.600161264837757	-1.92783720739668	0.0538753781157492	0.334982247080747
Cx3cr1	14.217756328165	-1.44100728256221	0.747617772662313	-1.92746525732086	0.0539216737964203	0.335086792985299
Tnfrsf25	21.8028609564389	-0.951093043566166	0.493513730323314	-1.92718659102571	0.05395638043695	0.33511924592698
Mrgprf	35.6808140621029	1.00971487604185	0.524142134488963	1.92641424835331	0.0540526697285058	0.335533939996831
Ghr	2630.58064307466	0.357202781314495	0.185494065345407	1.92568307050336	0.0541439590404399	0.335917160727969
Pkd1	677.656031404655	-0.299930919361157	0.155790547432313	-1.9252189834654	0.0542019681441901	0.336093600614765
Tacstd2	243.749416765314	-0.83580445735971	0.434190484114826	-1.92497184516525	0.054232880642455	0.336101920055695
Cpt2	728.573343726055	0.268270622105021	0.139425494949151	1.92411454019124	0.054340227923658	0.336421034158873
Fa2h	273.223903202448	-0.631316123416599	0.328114995675563	-1.92406970646608	0.0543458466446029	0.336421034158873
RGD1311084	59.1746904102042	0.772160479885029	0.401361737636296	1.9238517463883	0.0543731690788184	0.336421034158873
Nlrp1a	186.182873848762	-0.596511047404073	0.310464410178243	-1.92135081461223	0.0546874949204368	0.338119440104241
Taf1	834.753206518428	-0.237469999359846	0.123605379194449	-1.92119469967623	0.0547071661403924	0.338119440104241
Pgap2	240.400882874533	0.31493968231492	0.163980344939804	1.92059409577734	0.0547829001017987	0.338219686342772
Samd5	61.330626308657	-0.68132535346141	0.354733953304157	-1.9206657471472	0.0547738605348151	0.338219686342772
Enpp2	198.407410012603	0.542627234611981	0.282613936699023	1.92002999197405	0.0548541111368447	0.33829182158512
Tmem151a	32.3689264468722	0.904464029055636	0.471039875457856	1.92014323240997	0.0548398097622328	0.33829182158512
Gstz1	4363.84806813871	0.343350062906915	0.178900400523927	1.91922467418397	0.0549559061524972	0.33842390885391
Lactb2	9371.74080766028	0.458263758040435	0.238784262160778	1.91915394211314	0.0549648544557731	0.33842390885391
Tspan13	574.954973460829	-0.445958798088331	0.23234965911131	-1.91934345758902	0.0549408816150388	0.33842390885391
Tmem65	338.781930050562	-0.34350574159431	0.179098249581117	-1.91797375126623	0.055114339541239	0.339160575650093
Itpk1	333.334068328578	0.313691868301155	0.163599506227425	1.91743774498366	0.0551823428861221	0.33921174107283
Trappc9	461.012902449586	0.211792049435579	0.110452076724891	1.91750174116781	0.0551742199880255	0.33921174107283
Tnpo3	1053.50360136049	0.179882578988865	0.0938264607430128	1.91718388996422	0.0552145739783432	0.339226404236675
Muc1	1419.97083443645	-0.363537532041898	0.189711479119677	-1.91626534002492	0.0553313302238897	0.339576619505794
Uba7	181.053721983612	-0.524712329391713	0.273812549972714	-1.91631950195125	0.0553244400348489	0.339576619505794
Mthfd1l	40.6610086029184	-0.781344784103135	0.407931367158636	-1.91538294675753	0.0554436843246487	0.340082523493771
Dars2	577.164282722373	0.23080633863846	0.12057108216591	1.91427608090024	0.0555848890088202	0.340339226367795
Myo5a	499.407171432539	0.470469768522838	0.245786327012192	1.91414133667208	0.0556020989903018	0.340339226367795
RT1-S3	1398.78095335993	-0.37437718950841	0.195587492606929	-1.91411620711756	0.0556053091118244	0.340339226367795
Tmf1	727.648859433756	0.312728747951137	0.163324143298907	1.91477353950542	0.0555213903745664	0.340339226367795
Fermt3	90.142826037373	-0.997243918414138	0.521076903411736	-1.91381331984725	0.0556440129506945	0.340392815499243
Mxd3	16.7275550985412	1.37026265627738	0.716569271798417	1.91225427911295	0.0558435870824257	0.341429914351184
Fgfbp1	33.8546681040706	-1.32733957340871	0.694731169418054	-1.91058013781153	0.0560585588577123	0.342559989234816
Igf2r	4428.02892090517	0.307988751859826	0.161255878181507	1.90993813889474	0.056141178737045	0.342696368166087
Ss18	1586.93443766141	-0.161699454525627	0.0846531891202333	-1.91014014009519	0.0561151719502421	0.342696368166087
Atl1	29.9867985839475	-0.749420637546404	0.392463774428651	-1.90952818164533	0.056193989825576	0.342834615328769
Pawr	460.301741528808	-0.242362913513106	0.126979358782501	-1.90867961404848	0.0563034345506667	0.343133960913071
Vopp1	205.692569444918	-0.783343739238241	0.410398860280038	-1.90873760883186	0.0562959489786934	0.343133960913071
Abcc1	275.107228097329	-0.466783120824918	0.244633642041725	-1.90809046919762	0.0563795243362451	0.3432382976485
Mfap2	14.6583891681815	-1.44341978503487	0.756477832867811	-1.90807942059963	0.0563809521124185	0.3432382976485
MGC109340	9.26774345129856	1.92804087804985	1.01095464737957	1.90714873614499	0.0565013296914398	0.343603056860837
MGC112830	504.316060626026	-0.224242197325212	0.11757332407306	-1.90725403992039	0.0564876986540493	0.343603056860837
B3gat3	1524.33174924307	0.208884128485739	0.109559731238448	1.90657759127868	0.0565753091897107	0.343868964839707
Gna15	23.8041694046411	-1.01851202343092	0.534631744433379	-1.90507210624086	0.0567706987143573	0.344645378899821
Pebp1	11834.27391115	0.307025521439125	0.161197060217481	1.90465955784118	0.0568243392625607	0.344645378899821
Stk40	794.387510639081	0.238291368860393	0.125085722409299	1.905024524547	0.0567768832517628	0.344645378899821
Tmem51	909.473509516086	0.259398287499401	0.136180903940372	1.90480662114699	0.0568052128980436	0.344645378899821
Coa5	4769.23691708158	0.190677296293142	0.100181066689278	1.90332667233968	0.0569979325911438	0.345310275113464
Csrnp3	28.3452920699855	0.992512207736681	0.521451067313342	1.90336595310922	0.056992810411147	0.345310275113464
Sbno1	1074.38078664609	0.183939972831875	0.0966518852243683	1.90311831378018	0.0570251087795154	0.345310275113464
Hic1	21.5657813692791	-1.08412238885688	0.570098219138738	-1.90164142328789	0.057218048621563	0.346294111093017
Zfp868	546.132835814367	-0.235461406894139	0.123835355096919	-1.90140696661189	0.0572487277430587	0.346295390914106
Cept1	787.165171461384	-0.234124736982181	0.123173467959424	-1.90077247040984	0.0573318214469622	0.34642928366091
Elmo3	1234.59044920868	-0.20883289346509	0.109866420140293	-1.90078909641747	0.057329642823913	0.34642928366091
Tff3	46590.9264326803	-0.443995719878117	0.233669642570552	-1.90010013707305	0.057419979730792	0.346777624665346
Ak3	1822.73377510167	0.40671351294802	0.21437955633259	1.89716556888961	0.0578060895449508	0.348738860810993
Rsph1	68.313641482453	-0.597924362172008	0.315155592051411	-1.8972354521143	0.0577968697814223	0.348738860810993
Inpp5a	524.317087719336	0.25965557589213	0.136894610862113	1.89675527953154	0.0578602440898851	0.348880389562671
Rem1	94.4179283424623	-1.02644388361348	0.541360885294685	-1.89604367713921	0.0579542692614577	0.349076960479983
Ucp2	430.479426222047	-0.37130105450069	0.195820497675366	-1.89612966420011	0.0579429009118157	0.349076960479983

Kcnj8	44.1202851681506	-1.10919934472439	0.585390494409632	-1.89480245292165	0.0581185781231863	0.349881228256428
Cln6	642.607759831021	0.249230802835078	0.131744258931457	1.89177733327072	0.0585206545740915	0.351975065519625
Gstm1	460.96371379737	0.453970336314742	0.239977573679605	1.89171983595784	0.0585283190068706	0.351975065519625
Apoo	814.631893614755	0.242079600621006	0.12798856349472	1.89141587350491	0.0585688512736055	0.352032556095082
RGD1564894	79.8012119164965	0.85098132832907	0.450074111710924	1.89075822444914	0.058656626124167	0.352373790976365
Gpnmb	49.4617065196159	-0.917194264785898	0.485430676572612	-1.88944438217575	0.0588323086473064	0.353055976813772
Pcdhga9	21.9511677844161	-0.955083684021668	0.505482686969186	-1.88944885481288	0.0588317098411936	0.353055976813772
Cggbp1	1068.0122785864	-0.278414851058569	0.147421781906539	-1.88855980071572	0.0589508377931688	0.353394104618753
Kifap3	1021.0206859338	0.297946796710522	0.157759415710832	1.88861498610422	0.0589434374550298	0.353394104618753
Nacc2	255.315487046097	0.353774061479369	0.187355814163879	1.88824703977388	0.0589927934828446	0.353459193846079
Pola2	176.531692209474	-0.538268811945669	0.285143468347045	-1.88771222804426	0.0590645938727587	0.353702936753306
Cdh2	1408.00137836971	0.235011023058464	0.124525452596965	1.88725291221461	0.059126316555883	0.353886105304985
Arhgap9	32.9207747943389	-0.875048376008346	0.464173960495963	-1.8851733412046	0.0594064387834107	0.354328716399773
Cacnb4	234.487998155662	-0.379268458042309	0.201101423987528	-1.88595610375106	0.0593008701874778	0.354328716399773
Gimap9	87.673164331786	-0.690220840157677	0.36606540458082	-1.88551234703003	0.0593606990604822	0.354328716399773
Pkig	1532.21208891774	-0.162942575361357	0.0863850936752348	-1.88623486331959	0.0592633124198452	0.354328716399773
Purb	593.262334925996	0.248379593989266	0.131695394185421	1.88601579824089	0.0592928257821256	0.354328716399773
Rprm	26.4646565289959	1.02009925347005	0.54095845060494	1.88572570098369	0.05933192766615	0.354328716399773
Zfp394	181.125615805066	-0.35192465730231	0.186689104329822	-1.8850840736831	0.0594184878988182	0.354328716399773
Fh	5831.58990825928	0.310529121253844	0.164819126404356	1.88405998762555	0.0595568617006494	0.354781598579445
Klhl7	959.460868793364	-0.252550309596668	0.134036879229673	-1.88418524101805	0.059539923212099	0.354781598579445
Slit2	579.941723324416	0.338156503822924	0.179510840275736	1.88376648063984	0.0595965694179803	0.354832167629525
Fam91a1	1872.21756030991	0.197854296283352	0.105088928176339	1.88273207955221	0.0597366857070892	0.355294175691667
Zfp638	1129.33869667514	-0.195661334269404	0.10391507329174	-1.88289656227338	0.0597143872097366	0.355294175691667
Abcc12	6.2546203222394	-2.92341508500604	1.55325948673263	-1.88211635594489	0.0598202190938331	0.355419033047834
Gal3st1	259.586250022219	-0.540552425933101	0.287176307741795	-1.88230160831764	0.0597950762731161	0.355419033047834
Hist1h1a	160.814476319613	0.844155447266579	0.448577087160736	1.88185146194081	0.0598561862682021	0.355446924307411
Bbc3	49.4142011338599	-0.679149639680215	0.36105606401168	-1.88100881656497	0.059970719525585	0.355567509728755
Dusp5	664.82693802163	0.353497472074261	0.187940137047563	1.88090462009613	0.0599848946382937	0.355567509728755
LOC683788	90.7830059296747	-0.747783247683683	0.397640328723907	-1.88055182954768	0.0600329096577055	0.355567509728755
Slco1a6	23264.6211138856	-0.472963176889022	0.251473900272885	-1.88076447049093	0.0600039652751865	0.355567509728755
Suclg2	7143.22448553113	0.205386389917747	0.109178294380923	1.88120167183738	0.059944490406603	0.355567509728755
Grk5	126.739166251893	-0.551386994657113	0.293246848529808	-1.88028276321294	0.0600695511767467	0.355599228476512
Hs6st2	46.4157601703536	0.725930156278273	0.386311078482704	1.87913367416092	0.0602262431730975	0.356341218066333
Atp5f1	5296.87439370575	0.265979687593028	0.141596119024648	1.87843910853749	0.0603211196871485	0.356615546191742
LOC690911	670.736544123777	-0.317656129676925	0.169115807137282	-1.87833494132848	0.0603353594339892	0.356615546191742
Npl	129.922931993181	0.602009687812733	0.320606859770752	1.8777192984679	0.0604195752531258	0.356742281728326
Ttc7b	636.820170409077	0.193862771153665	0.1032372333038	1.87783772336458	0.0604033679563811	0.356742281728326
LOC361635	934.23555914643	0.185670551024112	0.0989269355828289	1.87684526898798	0.0605393039646634	0.3572636183086
Ccdc120	54.723611896307	-0.547090734203896	0.291633277642971	-1.87595441310942	0.0606615399168124	0.357613621729507
Ptpru	245.131120682284	-1.45884797117657	0.777574490815848	-1.87615204511907	0.0606344048313632	0.357613621729507
Rnase4	70.5732583617484	-0.96486461967549	0.514440299516485	-1.87556188848804	0.0607154638068316	0.357745962482347
Eif2ak4	358.810327793408	-0.279306574488562	0.148957890808389	-1.87507068590174	0.0607829997350532	0.357958329009645
Cep112	86.8747778857863	-0.514984316053652	0.274715296570679	-1.87461099721164	0.0608462591569084	0.357959928352702
Mocs1	266.698568908438	0.374733995696978	0.199877405980851	1.87481918658119	0.0608176027108815	0.357959928352702
Nsrp1	422.764437047488	-0.257918665708389	0.137643290375441	-1.87381938491066	0.0609553236268516	0.358230717860804
Scamp1	1391.9257822488	-0.162752092772788	0.0868542143735304	-1.87385372082058	0.0609505896355345	0.358230717860804
Mfn1	1444.25141250658	0.261404314639364	0.139552404486466	1.87316238370307	0.0610459649760842	0.358578004092079
Fn3krp	477.027248027221	0.333442880306376	0.178073918485404	1.87249701215346	0.0611378748771317	0.358932379056549
Chrnb1	162.868665622737	-0.354265432980632	0.189376858226376	-1.87069020100203	0.0613880338105134	0.359472638995515
Gpatch1	202.781418322269	-0.347389843844634	0.185629978333313	-1.87141024829981	0.0612882395041704	0.359472638995515
Mtss1l	258.662630291924	-0.531492508328598	0.284111577185962	-1.87071753144616	0.0613842435171509	0.359472638995515
Plin1	79.7836342203311	2.6052293209521	1.39264764490804	1.87070242101629	0.0613863390669761	0.359472638995515
Rras	681.318183327537	0.260699614917302	0.139314106314471	1.87130809516755	0.0613023891424122	0.359472638995515
Apitd1	158.020649072437	-0.394727988971442	0.211077305742868	-1.87006361286559	0.0614749846804993	0.359796434540965
Ceacam20	84.7763321340915	0.729302112163891	0.390096622058663	1.86954223883081	0.0615474128196423	0.359828144524201
Gcdh	13406.9274044957	0.392297922157432	0.209892925910854	1.86903832253998	0.0616174828910636	0.359828144524201
Itm2b	28055.7630122638	-0.193046245960062	0.103280861641035	-1.8691386079932	0.0616035328360117	0.359828144524201
Paqr6	72.2839164069265	-0.511161416461544	0.273544598129017	-1.86865841971573	0.0616703524136146	0.359828144524201
Stx4	884.78415960279	-0.237540092574188	0.127062818093857	-1.86946973266976	0.0615574908099735	0.359828144524201
Vamp3	1477.7886371434	0.172751466187517	0.0924359551280804	1.86887738594955	0.0616398751957425	0.359828144524201
Prss36	141.833256375226	0.527219353879851	0.28222797463184	1.86806199692854	0.061753429883145	0.360128006183595
Ddb1	5077.55594789217	0.179870800512558	0.0963094399207837	1.86763416608491	0.0618130807291242	0.360291013111398
Hibadh	7586.77709193077	0.258624692120918	0.138586087693759	1.86616634053784	0.0620180967153172	0.36130071105397
Ggt7	31.5785932857852	-0.761162364428249	0.40805523399518	-1.86534150530523	0.0621335508692564	0.361787878678262
Rexo4	1135.58695884809	0.393493804256105	0.211046270262202	1.86449068143792	0.0622528289810334	0.362296801944918
Ddi2	2233.32183819086	0.363195899939838	0.194856239571693	1.86391721783283	0.0623333304129349	0.362394185919907
Gpihbp1	26.7943820688728	1.96773883274876	1.05560405320536	1.86408798523811	0.0623093494997819	0.362394185919907
Npap60	822.707868669014	0.20991641721009	0.112721461817615	1.86225776196674	0.0625667660674231	0.363565369694443
Mtmr10	806.934643560225	-0.315779859196134	0.169595385807174	-1.86196020424264	0.0626086998580466	0.363623138776984
Ppme1	899.860866507299	0.177165082406259	0.0951938788075855	1.8610974216563	0.0627304202682017	0.364144002435333
RGD1308428	549.860223529068	-0.260756398093246	0.140183372623563	-1.86010932119218	0.0628700606856287	0.364360068621518
Smc3	1046.63319869834	-0.202506996569211	0.108892269714302	-1.85970039104267	0.0629279266852051	0.364360068621518
Stat1	940.395824337183	-0.212813616597185	0.114416242932274	-1.85999479744459	0.0628862620267001	0.364360068621518
Sucla2	4647.25606762313	0.269858447662297	0.145098512741241	1.8598291778741	0.0629096978500364	0.364360068621518
Tfap4	114.76388154642	-0.497501460693439	0.267457757440209	-1.86011228634734	0.0628696412595957	0.364360068621518
Aifm3	95.8459558185489	0.494532322463942	0.266015435174135	1.85903619517498	0.0630220081031048	0.36453340666661
Tspan6	611.455913789813	-0.283247865032653	0.152351757005674	-1.85917032136429	0.0630030001542057	0.36453340666661
Folr1	5612.11835299322	0.236525234039859	0.127300396138472	1.85800862538226	0.0631677894462518	0.365005132102744
Mier2	237.954005477345	-0.347930963589305	0.187245830369284	-1.8581506616362	0.0631476221760538	0.365005132102744
Arf1	3632.57387129381	0.249867694529577	0.134508592664615	1.85763369893104	0.0632210496178922	0.365127261156993
Cdc42se1	1792.52670731588	-0.221495757419644	0.11925557483215	-1.85731994274812	0.0632656487671628	0.365199270638686
Stam	913.712311722105	0.214642155690271	0.115626010014065	1.85634837407398	0.0634039181943669	0.365761418308343
Zbp1	31.1843872481064	-0.78827357177076	0.424674355435445	-1.85618359498655	0.0634273935848799	0.365761418308343
Cdk13	431.609450695128	-0.214783576858969	0.115743265121669	-1.8556896302622	0.0634978097196244	0.365796302723391
Large	768.540589097595	0.275082813207995	0.148234801162236	1.85572356188429	0.0634929706012653	0.365796302723391
LOC100364769	1407.81096907078	0.298004045207383	0.160646813415812	1.85502618365694	0.0635924876179611	0.365970741400378
Prdx6	5894.54872542262	0.19536117889277	0.105306295183182	1.8551709425627	0.0635718196936192	0.365970741400378
Nhlrc1	28.7027248088397	0.781941297608011	0.42162959748817	1.85456927660291	0.0636577588472308	0.366160975231591
Sec23a	250.728639597445	0.446068605233647	0.240573044979261	1.85419195767382	0.063711702267301	0.366285891740083
Ptk7	229.854688676225	-0.706979834284712	0.381706341558314	-1.85215637602056	0.0640033710063659	0.367776701141737
Hspb1	290.900636756444	-0.531341378448866	0.287111376893492	-1.85064550279376	0.0642205689711103	0.367911214568156
Larp4b	3008.85897373534	0.177678395584309	0.0959556900401092	1.85167128192231	0.064073040263429	0.367911214568156
Ly6c	142.187301432539	-0.608604540532003	0.328971859401945	-1.8500200644469	0.0643106577612099	0.367911214568156
Plp1	79.33453490271	-0.662527765499372	0.358120469108153	-1.85001367598256	0.0643115785002335	0.367911214568156
Ptprc	36.3879505651467	-1.34372431879723	0.72609753472629	-1.85061132221549	0.0642254896843279	0.367911214568156
Pum2	2264.9787710487	-0.177556250220599	0.0959779912183061	-1.84996839344907	0.0643181051686544	0.367911214568156
Rqcd1	573.487686112112	-0.211915672114751	0.1145407264824	-1.85013382246457	0.0642942641804576	0.367911214568156
Sowahc	350.152197899965	-0.340822126468346	0.184156980603589	-1.85071521780643	0.0642105336053078	0.367911214568156
Zfp217	220.90861247605	0.411624763866147	0.222338362555566	1.85134386677546	0.0641200990424199	0.367911214568156
Gpat2	33.0122574965942	-0.686993372089556	0.371545102756063	-1.84901743285956	0.064455295513675	0.368510507448909
Psmb9	204.509558403873	-0.394364567923117	0.213345582934288	-1.84847777253764	0.0645332569840895	0.368770738502344
Lpcat2	31.974770066974	-0.809587662447249	0.438152987571727	-1.84772827165698	0.0646416619399568	0.369204587743492
Tubb4a	28.2904305894135	-0.714106108926965	0.38707111514939	-1.84489640528034	0.0650526096979791	0.371365123971487
Ltb	30.4676863415767	-0.92878040335608	0.503556531277881	-1.84444118120979	0.0651188703972467	0.371370336645814

Thrsp	1575.70825878174	2.77364348005263	1.50374577328545	1.84448962672237	0.0651118162052548	0.371370336645814
Arhgef25	105.644397751488	-0.698063113178168	0.378700325991518	-1.84331268094499	0.0652833707208049	0.371935233891062
Spon2	42.7900834104821	-0.870902014126276	0.47246014655035	-1.84333434361635	0.0652802097524333	0.371935233891062
Magi2	22.9118713830081	0.853284064266943	0.463043722353013	1.84277212512649	0.0653622882940058	0.372198280936708
Pdpr	187.91037687081	0.490127573885244	0.266048347777158	1.84225001951816	0.0654385869276892	0.37244615874818
LOC684352	108.83382365582	-0.489660187035982	0.266024418824401	-1.84065879816544	0.0656715753764924	0.373524710659788
Odz3	135.720238842357	-0.576454916370038	0.313204387377237	-1.84050715635644	0.0656938145881503	0.373524710659788
Akap2	839.203926175516	0.802516160883431	0.436563725664509	1.83825662487622	0.0660245993710864	0.374468860504874
Fry	220.802980285684	-0.522214156392107	0.284016342127105	-1.83867643840861	0.06596279094417	0.374468860504874
Mmp23	73.0219509354277	0.932973673275453	0.507317503958475	1.83903308282424	0.0659103202663738	0.374468860504874
Nr4a2	375.109014223685	-1.09321411798547	0.594579583556244	-1.83863379809788	0.0659690666276039	0.374468860504874
Oxa1l	2321.83596764592	0.215435625498775	0.117188636825491	1.83836617042987	0.0660084665668546	0.374468860504874
Ccne2	123.147939052611	-0.386729099927321	0.210787999533726	-1.83468271809964	0.0665527143877217	0.376546704787363
Clvs1	272.567220202139	-0.27260018179233	0.148531441753166	-1.8353028730802	0.0664608256180019	0.376546704787363
Ptgs1	662.56459575943	-0.903187911791506	0.492097757379527	-1.83538310883813	0.0664489446715822	0.376546704787363
Spsb4	1113.77395034637	0.363585899111173	0.198148614231262	1.83491517476285	0.0665182588793739	0.376546704787363
Ucp3	11.1186684413434	4.82507350410752	2.62996015920292	1.83465650124901	0.0665566012597054	0.376546704787363
Atmin	456.681368800725	0.269794252794501	0.147145765157279	1.83351693816079	0.0667257319851991	0.377128130156028
Odf2	217.031165125371	0.342948038067921	0.187023735076073	1.83371398249759	0.0666964619417149	0.377128130156028
Msra	2703.20962490702	0.253624366173195	0.138349274287879	1.83321790069856	0.0667701728518475	0.377191741865854
Pcyt1a	235.158945173449	0.284778708262757	0.155364664976574	1.83296960287396	0.0668070916639164	0.377212818605104
Adm	74.7068003175622	0.798572925636834	0.43598734641843	1.83164243686655	0.067004709940017	0.377462395354844
Atp8b2	1302.87902195406	0.244598121805227	0.133595207488986	1.83088994285512	0.0671169717589425	0.377462395354844
Kdelc1	187.982221598203	-0.31541261515322	0.172207873293158	-1.83158068862669	0.0670139161077035	0.377462395354844
Neurl2	122.649066370411	0.540693874534004	0.295193719422076	1.83165778591958	0.0670024216807979	0.377462395354844
Plbd2	3090.36123709432	0.235196847494131	0.128382930288881	1.83199469715251	0.0669522106639967	0.377462395354844
Rnd1	51.4279596993259	-0.542907112001152	0.296523672174216	-1.83090647711316	0.0671145034112094	0.377462395354844
Rora	57.2569387348127	0.547395529336746	0.298918371510079	1.83125422024551	0.067062607234585	0.377462395354844
Slc23a2	423.411185184531	-0.445168847538511	0.243078470786749	-1.8313791677958	0.0670439684932217	0.377462395354844
Mras	1070.56848956448	-0.340386952034144	0.186046038080848	-1.82958452405327	0.0673120896290565	0.378205874730123
Pomc	202.08495273864	0.590332692949526	0.322663705793664	1.82956025840424	0.067315720972993	0.378205874730123
Cnksr1	576.097866083589	-0.375775952075766	0.20561924099726	-1.82753301808353	0.0676196664531613	0.379725854202881
Il1rn	55.4180349972819	-0.845693536782095	0.463027059771636	-1.82644516974707	0.0677832332105452	0.380454017060012
Zdhhc4	462.936896888016	0.259159982020752	0.141910164703901	1.82622564466397	0.0678162800073539	0.380454017060012
Slc25a19	296.76174402337	0.31208617047339	0.170945106830791	1.82565138165848	0.0679027909094332	0.38075141661402
Caprin1	3126.68042825029	0.236793535891355	0.129753008208644	1.8249560388657	0.0680076635668858	0.38097320184003
RT1-Ba	463.076893785708	-1.02752900302001	0.563046705940773	-1.82494452445672	0.0680094013080501	0.38097320184003
Taf9b	437.01476945848	0.249344941226409	0.136648134992178	1.82472260774494	0.0680428998535335	0.38097320184003
Ect2	115.814997361758	0.61631674200362	0.337817598367827	1.82440685441305	0.0680905865157905	0.381052489580736
Dnajb14	105.573119928146	0.418093436950302	0.229306875540505	1.82329219725664	0.068259147460644	0.381807810057913
Rarg	135.778071991397	-0.504165177438802	0.276567641973502	-1.82293624026742	0.0683130482886157	0.381921351130549
Acin1	2622.27941723997	-0.234333059346357	0.128646010830524	-1.82153381852674	0.0685257507772422	0.382389605617517
Fem1a	537.344614589148	0.349985668056246	0.192087037458465	1.82201606462864	0.0684525481530795	0.382389605617517
Gpi	8126.37886247152	0.240126222110152	0.131819739792691	1.82162567220806	0.0685118028745216	0.382389605617517
LOC100360552	32.6425713573504	0.818149949567441	0.449163545743567	1.82149677399361	0.068531376618237	0.382389605617517
Abcc9	170.908695382421	-0.564193118640439	0.309869802264727	-1.82074250061462	0.0686460084132828	0.382841281464854
Gucy2g	51.3103785818467	-2.84222694503521	1.56148909512491	-1.82020287807892	0.0687281149930268	0.383111208931213
Chst15	21.2201174120962	-1.19155050437542	0.654839612807381	-1.81960663507678	0.0688189305248024	0.383267801004793
Coro7	718.704992208954	0.383200036126121	0.210625022543568	1.81934715779962	0.0688584830563259	0.383267801004793
Tppp3	18.9836161352938	0.92680195936995	0.509474449559962	1.81913334450911	0.0688910889893359	0.383267801004793
Trank1	13.0209754005507	-1.42843471367561	0.785160918259772	-1.81928911698966	0.0688673328645411	0.383267801004793
Ankrd6	120.229188611923	-0.438103387115418	0.241030582059491	-1.81762572770656	0.0691213566107085	0.384206870597502
Dock6	364.180798915614	-0.512389162005346	0.281906477540834	-1.81758562795396	0.0691274899148242	0.384206870597502
Cdh23	44.3570280835005	-0.711745537717114	0.391720655168761	-1.8169721926215	0.069221371308447	0.384329155920345
Ghrhr	206.812998400276	-0.354655640754607	0.195165281115592	-1.81720661957571	0.0691854817737207	0.384329155920345
Hps3	160.030333943192	-0.374533681448262	0.206152562580702	-1.81677916956111	0.069250933602399	0.384329155920345
Man1c1	572.282332366862	-0.913243761391546	0.502869361589356	-1.81606562488749	0.0693603060032192	0.384748286009073
Camkv	30.9152894742007	1.23292693304841	0.679133502778717	1.81544118793111	0.069456136295864	0.38490416632803
Egln3	298.941722087037	-0.502238990173655	0.276622431543242	-1.81561194213979	0.0694299204130259	0.38490416632803
Gpam	186.347512801662	0.58297328980476	0.321203907690896	1.81496325494823	0.0695295565350855	0.385123264901453
Bcl2l14	112.351513385632	0.511720476145728	0.282038423786046	1.81436440211395	0.069621642464692	0.385445488676907
Myl12a	3014.83613448719	-0.234403574343527	0.129227722265607	-1.81387994954943	0.0696962102480772	0.385670460408786
Bcl2a1	24.2348360680224	-0.925622173677666	0.510369340377553	-1.81363201205018	0.0697343985779989	0.385694002062061
Fyttd1	1415.50219489069	-0.201870570165436	0.111335977990875	-1.81316564338242	0.0698062770977093	0.38590376726292
Dguok	430.671077892441	0.340303211608434	0.187758499968188	1.81245169548165	0.0699164312810922	0.385949566751284
Rgs14	20.4141338098128	-0.871659426532404	0.480849707453627	-1.81274816854592	0.0698706714906465	0.385949566751284
Tbc1d2	186.814133877699	-0.317605011965999	0.17521931942828	-1.81261411699524	0.0698913589278768	0.385949566751284
As3mt	921.764869471398	0.281645802119699	0.155504123023697	1.81117900055151	0.0701131477203528	0.386847590771617
Acad11	1654.94283966671	0.242315037506598	0.13382279008693	1.81071577830049	0.070184859168833	0.386928222390387
Ola1	833.118696556548	0.243961967681419	0.134737615346619	1.81064483777462	0.0701958467859386	0.386928222390387
Usp42	312.267201968497	-0.324849456219857	0.179451347576612	-1.81023692831937	0.070259053200613	0.387088899019955
Lep	15.4656622447772	2.17918864009226	1.20412710611164	1.80976628549563	0.0703320382947351	0.387135568404223
Pcdh18	32.2026591851704	-1.16614888363121	0.644391996531173	-1.80968865210727	0.0703440832929665	0.387135568404223
Pld2	305.37040470649	-0.478904641912886	0.264657867920103	-1.80952353949085	0.0703697065214785	0.387135568404223
Cyp2d5	5442.16952901671	-0.385326309365202	0.213053497538378	-1.80858945671986	0.0705148076703292	0.387746155772115
Clpb	1541.99473625689	0.206177754567985	0.114069895842683	1.80746859673064	0.0706892467910804	0.388329617702958
Tax1bp3	748.495559707287	-0.285042327987245	0.15768930692958	-1.80761989216262	0.0706656800844453	0.388329617702958
Clk1	2342.98806551746	-0.41872408598547	0.231763292749753	-1.80668854423633	0.0708108548744811	0.388420014296074
Irak1bp1	271.931842985622	0.451006088630149	0.249679274700696	1.8063417124661	0.0708649800838514	0.388420014296074
Mbd1	347.608390003404	-0.29850983158174	0.165283386088512	-1.80604862137737	0.0709107451754666	0.388420014296074
Mical1	62.1631345414351	-0.785025419386903	0.434658708635091	-1.80607314150458	0.0709069155194255	0.388420014296074
Prtfdc1	26.7955896943908	-0.868203511645792	0.480636178837451	-1.80636321166205	0.0708616240174467	0.388420014296074
Sfxn2	140.935061868918	0.486264642715396	0.269113879137035	1.80691030977182	0.0707762647837991	0.388420014296074
Nrp2	29.5498461141506	-1.58294856283836	0.876852928894314	-1.80526119110352	0.0710338195336983	0.388587674584614
Rpl23	6190.72869867537	-0.24864209223239	0.137736847732819	-1.80519662185608	0.0710439193900077	0.388587674584614
Wdtc1	1148.98247139006	0.250862042258539	0.138953902269978	1.80536162108734	0.0710181127167573	0.388587674584614
Atg7	244.47261736949	0.309677006903387	0.171772412564454	1.80283319236252	0.0714144156306418	0.389264287494123
Dnajc22	1274.17686431205	0.265920272560371	0.147490987133389	1.80295947385501	0.0713945795102607	0.389264287494123
Fam69a	249.806934025651	-0.262476541840148	0.145602369024652	-1.80269417042045	0.0714362582260449	0.389264287494123
Kcnj13	39.3751042696498	-0.619769855241306	0.343798095913916	-1.8027146240987	0.0714330442789463	0.389264287494123
Letmd1	983.166019850564	0.208556184655575	0.115646011270565	1.80340145210575	0.0713251897403484	0.389264287494123
Pgpep1	1480.22893462125	0.377455952088975	0.209383617525157	1.80270050040388	0.0714352635643212	0.389264287494123
Robo4	31.3063275475867	-1.09193842803867	0.605528571866559	-1.80328142844316	0.0713440277644387	0.389264287494123
Slc9a2	60.1444084946026	0.779503343942443	0.432522904799554	1.80222442625019	0.071510103139867	0.389264287494123
Syne4	262.753286294078	0.611186577098862	0.339055580297471	1.80261471161346	0.0714487449483898	0.389264287494123
Tmprss13	184.836053231189	0.503392660309205	0.279314402074389	1.80224383909548	0.0715070501552105	0.389264287494123
Ifi27	1204.01624964417	0.378650041209575	0.210129365548404	1.80198536373704	0.0715477083536581	0.389282552966816
Usp19	1705.08955914776	-0.177484355843897	0.0985731518090781	-1.80053445168984	0.0717762887180033	0.390339376827189
Tprn	536.524635249518	0.233383434551507	0.129635233706611	1.80030866515578	0.0718119134377363	0.39034634535309
Ppa1	495.639013051849	0.257008182255692	0.14277808464648	1.80005343881764	0.0718522006254443	0.390378638770937
Slc14a2	289.51075473481	-1.44223863310395	0.801374205278761	-1.79970683309211	0.0719069415888394	0.390489392307094
Kcnk1	1464.33064385482	-0.340705741562076	0.189402389344913	-1.79884605859766	0.0720430352605273	0.390942331685991
Scn3b	47.9985374205068	-0.833124318807799	0.463337815333598	-1.79809264695557	0.07216232728112	0.390942331685991
Sdccag1	931.177192096692	-0.228622351803602	0.127136835727942	-1.79823849236603	0.0721392221164874	0.390942331685991
Sike1	372.259038391631	-0.247450516910976	0.137602269399117	-1.79830258608048	0.0721290701596144	0.390942331685991

Usp18	82.3843087800204	-0.615691237079584	0.34230222269155	-1.7986772982026	0.0720697420224892	0.390942331685991
Zfp445	797.233265680359	-0.187421835413252	0.104251378051868	-1.79778760641421	0.0722106720970587	0.391017865200176
Fasn	2769.84098329434	1.3366339350496	0.743731201923429	1.79720029439778	0.0723038278310175	0.391335860536831
Aldh1a3	204.326545931356	-1.46594892772773	0.815858433297522	-1.7968177662915	0.0723645549315026	0.391371477896087
Arih2	1143.02955492622	0.219574765603236	0.12222307125215	1.79650832984098	0.0724137091338617	0.391371477896087
Ep400	845.731191984693	-0.2127937563276	0.118445478902212	-1.79655448481307	0.0724063756492638	0.391371477896087
Atp5a1	39135.3246978437	0.217002039849073	0.120813929493423	1.79616738532528	0.0724679000644621	0.391371609774798
Cmas	1111.23341823569	0.232529200390007	0.129465200043674	1.79607493219463	0.0724826006137559	0.391371609774798
Fmod	34.2709947122336	0.845773284799425	0.471095417852373	1.79533328652427	0.0726006146619723	0.391822690526105
Tspan12	762.331678396347	-0.443759020744056	0.247241695871281	-1.79483892949466	0.0726793663403761	0.392061546191132
Ptges3l1	299.984406628937	0.237117837088292	0.132152569955527	1.79427337030289	0.0727695463474139	0.392361794964282
Bex1	163.769906491159	-0.633687104533409	0.353294914600067	-1.79364909696124	0.0728691948031296	0.392377509984475
Cdc42ep3	68.2434269502163	-0.730669556114718	0.407284117555182	-1.79400454037033	0.0728124441377466	0.392377509984475
Ddx42	1072.3157554875	-0.170806848052628	0.0952417150669257	-1.79340374049967	0.07290838992292	0.392377509984475
Pabpc4	2128.9256471946	0.209560137499333	0.116851388109125	1.79339022745395	0.0729105491014615	0.392377509984475
Pde1a	345.036304925628	-0.459005273083571	0.256002206122367	-1.79297389673341	0.0729770980440119	0.392549785313885
Scap	1060.56425496245	0.230624636325299	0.128683126522713	1.79219018497031	0.0731025063448702	0.392852523459005
Tmem163	13.9390336597105	1.019115289807	0.568611406535788	1.79228780515653	0.0730868757119337	0.392852523459005
Hdhd2	355.119000786873	0.294048796469295	0.164114476584101	1.79172978880147	0.073176260381304	0.393063031896929
Ampd3	661.699993150724	-0.306413717505222	0.171121525120225	-1.79062053876591	0.0733542088397745	0.393275442298527
Cpeb4	683.82731776759	0.379134616073921	0.211721559454538	1.79072276366513	0.0733377948920004	0.393275442298527
Med15	944.294846995224	-0.242954530798915	0.135636773302467	-1.79121432103912	0.0732589089391246	0.393275442298527
Pard3	607.624135483054	0.215892159981578	0.120556726576495	1.79079314868915	0.0733264951247006	0.393275442298527
Gon4l	516.700067575342	-0.1985503867381	0.110961564623447	-1.78936181561507	0.0735565646732278	0.393654090152971
RGD1309313	468.100648649193	0.270339013448573	0.151031564643226	1.78995042584099	0.0734618813036758	0.393654090152971
Schip1	135.900747095263	-0.617798902053485	0.345270308108914	-1.7893195202253	0.0735633721172716	0.393654090152971
Stt3b	1105.27228157969	0.259161898523061	0.144810754731553	1.78965919350043	0.0735087162385686	0.393654090152971
Yif1b	243.224071530035	0.289298497019488	0.161700503082852	1.78910078511788	0.0735985857608471	0.3936571885919
Cyyr1	242.914996113707	-0.552376320785241	0.308898196844911	-1.78821477893759	0.0737413628097146	0.394235338520798
Fam213b	486.292052234942	0.4563088714832	0.255304344208889	1.78731338433415	0.0738868519780316	0.394827437509312
Ipmk	4078.25106002958	0.217948783609525	0.121971306568684	1.78688570075122	0.0739559640837371	0.394877643477459
Mkrn1	613.404196728945	0.2150868896149	0.120373766523923	1.78682528449547	0.0739657313886601	0.394877643477459
Tinag	2357.65336781214	0.244213271009158	0.136730358059496	1.78609399167149	0.0740840408371241	0.39532357190364
Ier2	976.164642051425	0.411164329434281	0.230241571871587	1.7857953543837	0.0741323992023363	0.395395987486511
Avil	30.6199649444238	-0.697592052345968	0.390731125849807	-1.78535060606898	0.0742044651593124	0.395594723733933
Sephs1	1409.4598847222	-0.160411031565267	0.0898707157164719	-1.78490880245506	0.0742761106420157	0.395791033078833
Basp1	1724.86610339124	0.363415142429823	0.20367408260452	1.78429743137951	0.0743753474147713	0.396134113737718
Kdm4a	960.548602159314	-0.322073875786555	0.180534440537923	-1.78400240323618	0.0744232746507892	0.39620371882008
Rexo2	580.086548312404	0.184879419236947	0.103689733515782	1.78300602160201	0.0745853231147508	0.396880516162106
Adamts10	691.93325531029	-0.290872785934961	0.163180431069959	-1.78252247544473	0.0746640694122655	0.397113623275531
Fam126a	27.0377362843772	0.866666802231205	0.4865515465224	1.78124354639433	0.0748726725513121	0.39782584407853
Prkcsh	4082.69954530307	0.15366570109914	0.0862777656986827	1.78105795687621	0.0749029831363765	0.39782584407853
Spag5	54.6036043627274	0.668490832195055	0.375333501122279	1.78105825937789	0.0749029337234788	0.39782584407853
S100a6	1277.34513955727	-0.439825741158258	0.247003256209905	-1.7806475424943	0.0749700478307674	0.397996059619104
Esrra	1756.87661728567	0.235327260566326	0.132178606348735	1.78037329237266	0.0750148896210569	0.398048195813694
Pik3c2b	45.6190020635104	-0.807221381314282	0.453623706877156	-1.77949557987472	0.0751585489186725	0.398624389645185
Cpsf6	978.573153325871	-0.332664214387493	0.187017728663431	-1.7787843792402	0.0752751191267511	0.398741240127464
Psmd1	2139.04456381594	0.181312791848608	0.101953918679943	1.77837982292561	0.0753414942584692	0.398741240127464
Rgs6	83.7746846635992	-0.469640993166878	0.264092823218955	-1.77831789384714	0.0753516591131529	0.398741240127464
Seli	111.638348862744	-0.413907968766463	0.232755972006146	-1.77829150933894	0.0753559901279072	0.398741240127464
Zbtb46	29.340497329987	-0.907596820144178	0.510321975013011	-1.77847881255954	0.075325248719177	0.398741240127464
Csrnp2	136.515657537812	-0.339083956830264	0.190812455489923	-1.77705357839269	0.0755594249404128	0.39916009147685
MGC125239	302.75780222502	0.396330678459136	0.223035909525617	1.77698147039239	0.0755712885773941	0.39916009147685
Net1	2427.8141736053	0.301193816933766	0.169487304602341	1.77708777445273	0.0755537993326613	0.39916009147685
Zfp131	377.971314224375	-0.306898268744693	0.172710195457067	-1.77695513534974	0.0755756217541951	0.39916009147685
Slc30a4	892.76523271646	0.306672478136763	0.172624773845236	1.77652645854706	0.0756461849000915	0.399347207419619
Pvrl3	188.37073393687	0.333783798296856	0.187920585045596	1.77619603629836	0.0757006113580161	0.399449001251258
Fabp5	1823.64646681225	-0.265895930936926	0.149780252401637	-1.77524023810512	0.0758582283633947	0.40004317685775
Mtm1	912.528485518634	0.189178283534133	0.106574121914686	1.77508648568151	0.0758836080683889	0.40004317685775
Gphn	789.043069696842	0.237627069084967	0.133888856640186	1.77480841235031	0.0759295268533198	0.400099676502009
Plcl2	58.221547934148	-0.784104082857098	0.441990567483246	-1.77402899641477	0.0760583541434485	0.400592795734215
Gareml	30.446241306784	-0.665952994256885	0.375652351877854	-1.77279069577987	0.0762633957272182	0.401486686352738
Ptprr	108.868509912079	-0.565979255855205	0.319384362239781	-1.77209445035474	0.0763788800253356	0.401887930953618
Ufd1l	1168.09901300728	0.18558124508527	0.104735438187605	1.77190498552028	0.076410330704801	0.401887930953618
Slc21a4	14730.9732804026	-0.421243505525003	0.237810175435716	-1.77134348752403	0.0765035999726911	0.402192376174656
Atp1a2	120.236555127864	1.86785138076874	1.05461017898554	1.77112967235484	0.0765391407639663	0.402193191827666
Gulp1	208.976140872078	-0.543905493972562	0.307249280779993	-1.77024171575532	0.0766868827879034	0.402597279338249
Parg	1019.47438811142	0.265774996738458	0.150122784012663	1.77038414579388	0.0766631690240127	0.402597279338249
Fcgrt	2447.0102910013	0.16836484490725	0.0951339124312786	1.76976685394781	0.076765987695307	0.402826507915448
Unc13d	66.7939824677734	-0.579638256693105	0.327654467981718	-1.76905341857095	0.0768849604408916	0.403264633304649
Sypl2	265.918507973242	0.34839812945263	0.197095874317738	1.76765815448261	0.0771180695708883	0.40430072820236
Plxna3	131.837896986988	-0.395459526441188	0.22374883870914	-1.76742605111467	0.0771569032905811	0.404317825173234
Cdc42ep4	588.73273651974	-0.335762950989565	0.190120033325953	-1.76605771162428	0.0773861671599161	0.405226851725931
Nefm	175.952876739236	-0.51279962315612	0.290379227398617	-1.76596524396759	0.0774016800190917	0.405226851725931
Cpq	3161.53710616505	0.255536580968876	0.144781806362071	1.76497715693524	0.0775676049125555	0.405835309938501
Fbxl19	302.720241149752	-0.274012513888848	0.155261252957682	-1.7648480137123	0.0775893127306319	0.405835309938501
Slc35g1	71.6527696760853	-0.55715604195949	0.315981074640477	-1.76325763368398	0.0778570472702221	0.407048389730149
Ndc80	22.208168834227	0.815178474382488	0.462778807939152	1.76148617956955	0.0781561501446815	0.408236582051677
Ppp1ca	5758.40158095923	0.183097022460161	0.103939712496226	1.76156945274222	0.0781420688828568	0.408236582051677
Bbs4	438.832298719415	-0.310247947711441	0.176179823107641	-1.76097320475732	0.0782429383215425	0.408502175913024
Prmt3	474.636790325209	0.270528137491802	0.153660953375134	1.76055225188769	0.0783142162591414	0.408686584940956
Rere	1625.19521784927	-0.220072890244365	0.125074919791887	-1.75952853386231	0.078487778114089	0.409404353393637
Bphl	2555.99979830467	0.285047315765097	0.162046329370881	1.75904827262517	0.0785693097425713	0.409641639694526
Armc7	148.359249420319	-0.442917475454476	0.251851217317802	-1.75864734811098	0.078637425530244	0.409674006542226
Nrgn	126.427731429495	-0.88339180194965	0.502330321728576	-1.75858745478433	0.0786476053383018	0.409674006542226
Arhgdia	6957.12384796218	0.162536928711681	0.0925370207150729	1.75645301151571	0.0790110879595957	0.409869317055511
Clcnka	1001.59437983311	-0.921634031533527	0.524282003473693	-1.75789751589246	0.0787649485884156	0.409869317055511
Coq6	813.456548698315	0.253609263953346	0.144390639522518	1.75641069789565	0.0790183074995942	0.409869317055511
Erlin2	1154.70361984472	0.319461344113469	0.181918028730825	1.7560730310362	0.0790759393688586	0.409869317055511
Flt4	50.7185978381408	-0.858999536900215	0.489213535613355	-1.75587851595978	0.0791091540793823	0.409869317055511
Kcnk3	15.1542408309552	1.19046354035753	0.678170069614363	1.75540560354496	0.0791899542718549	0.409869317055511
Lcp1	407.227589064818	-1.49208547006782	0.849463036960906	-1.75650429170645	0.0790023392645386	0.409869317055511
LOC306766	13318.2935031114	0.248551859232423	0.141502326682397	1.75652135947064	0.0789994275807253	0.409869317055511
Lonrf3	108.415823763682	0.534834408038197	0.304378670299552	1.75713497766398	0.0788948050452302	0.409869317055511
Mesdc1	347.596828220596	0.268708316215215	0.153047955919102	1.75571319853006	0.0791373920216977	0.409869317055511
Mgst2	36.5808742608084	-0.842936512923652	0.479899474190277	-1.75648559387551	0.0790055291306031	0.409869317055511
Mpo	30.7316444578815	2.80543960457642	1.59592124698643	1.75788097932396	0.0787677628377095	0.409869317055511
Pggt1b	146.710715661624	-0.410095906612418	0.23341399751326	-1.75694650270115	0.0789269282206069	0.409869317055511
Rpa1	468.56612158287	0.290338800770772	0.165387338761132	1.75550802706914	0.0791724488483466	0.409869317055511
Cdk1	124.437628573831	0.625599944850758	0.356578855587473	1.75445048142315	0.0793533478234627	0.410142451228155
Nrn1l	54.7939141943917	-1.18046111366291	0.672803714810004	-1.75454012467257	0.079338000828887	0.410142451228155
Slc6a8	3123.21304977062	0.251648201988269	0.143450299841306	1.75425357957883	0.0793870660480328	0.410142451228155
Tank	68.3393872784257	-0.489556650234965	0.279048266799971	-1.75437987772311	0.0793654454933128	0.410142451228155
Arhgef1	1219.84796104621	-0.240609138991746	0.137311108988228	-1.75229186308861	0.0797236344797163	0.411238586178713
Card9	29.5404034485515	-0.745935456509731	0.425902310514599	-1.75142383146138	0.0798729309264399	0.411238586178713

Gins4	74.0497864804094	-0.497954245040875	0.284270000197532	-1.75169467300404	0.0798263233700122	0.411238586178713
LOC100125362	10.917706332698	-4.74108480040611	2.70711927333541	-1.75133945781586	0.079887454816896	0.411238586178713
Pdzk1	13313.3991550345	0.289224973342107	0.16513128953482	1.75148497996268	0.0798624063016092	0.411238586178713
RT1-M3-1	130.369390962095	-0.610193305530581	0.348324177472252	-1.75179716193887	0.0798086924071758	0.411238586178713
Syngr2	974.763755687688	0.186740112278174	0.106627457998732	1.75133230954822	0.079888685402305	0.411238586178713
Usp4	789.645049244031	0.180219368458438	0.102900140909859	1.75140059930832	0.0798769298431423	0.411238586178713
Epb41l1	1220.87714504933	0.186733689316801	0.106641647593625	1.75103905022529	0.0799391837335372	0.411312250934986
Dus2l	277.990101599543	-0.275095479365791	0.157212526176392	-1.74983181083888	0.0801473398186815	0.411637896240006
Lrsam1	422.158284933203	-0.234875176588752	0.134223127722781	-1.74988603360409	0.0801379811189211	0.411637896240006
Mgat1	1288.80586628465	-0.187359661892614	0.107061679273013	-1.75001609506644	0.0801155364883887	0.411637896240006
Vwa9	483.234847877529	0.193882434287534	0.110774324968882	1.75024703912208	0.0800756951949237	0.411637896240006
Cep89	791.186034622035	0.190008175840065	0.10860207053013	1.74958152190432	0.080190550509285	0.411673801756338
Sema3f	361.253608193019	-0.503544188574878	0.287873095262879	-1.74918808621227	0.0802585127789475	0.411836684535222
Clcn6	1105.3938371011	-0.220505385635476	0.12612712274348	-1.74827888592959	0.0804157474985906	0.412407613363563
Lima1	756.032278164924	-0.420958218821515	0.240805526832211	-1.74812523765217	0.080442343729282	0.412407613363563
Asic2	23.3700597943181	-0.943914083406167	0.540255314428745	-1.74716297683114	0.0806090718225522	0.412783855032239
Dnase2	295.004061412526	-0.271023702933789	0.155102292439533	-1.74738682885328	0.0805702606143266	0.412783855032239
Rsad2	150.505536281697	-0.777105053919827	0.444804008724995	-1.74707295500181	0.0806246839848294	0.412783855032239
Bmpr1b	90.632880939965	-0.700784832845789	0.401305093012854	-1.74626448815924	0.0807650035681122	0.412831156826733
Col1a2	265.506999399411	-1.32759477064865	0.760075521107951	-1.7466616589802	0.0806960448184545	0.412831156826733
Ireb2	501.281176013122	0.354900027622195	0.203243352958244	1.74618270391903	0.0807792092893415	0.412831156826733
Orc2	300.341351772069	-0.274680985977953	0.157295953329689	-1.74626861126063	0.0807642874492664	0.412831156826733
Kansl3	1896.67285310281	-0.242193924159532	0.138743423861885	-1.74562453064895	0.0808762167983096	0.413141159608803
Bola3	626.954190735998	0.342060269427871	0.195990894677277	1.74528653482151	0.0809350046188206	0.413247198366585
Nupr1	422.326033381812	-0.952721629046467	0.546010349462823	-1.74487833423629	0.0810060494422621	0.413247198366585
Tst	4907.28907544619	0.235132677898379	0.134751516536776	1.74493529973896	0.0809961319051458	0.413247198366585
Ghitm	6362.2026576786	0.274038765680491	0.15709721517864	1.74438971033875	0.0810911580177211	0.413380395377398
Loh12cr1	188.488590822949	0.291017928224291	0.166838339936075	1.74431086005648	0.0811048989698749	0.413380395377398
Bbs1	769.137502447293	0.215203055916859	0.123397741595837	1.74397888594842	0.0811627716232887	0.413489942747781
Ncam1	57.851516611265	-1.0801549935077	0.619442390320654	-1.74375375399891	0.0812020376801774	0.413504641699326
Upk3bl	154.327168770816	-1.32750817703847	0.761675085491611	-1.74287987401038	0.0813546002809327	0.414096008416069
Fam221a	24.1572880778583	0.843026983324628	0.483917177243314	1.74208939663399	0.0814928026958259	0.414613784888432
Mfap4	20.9501137003955	0.925283826541019	0.531334299843914	1.7414343979164	0.0816074630949104	0.414825771706955
Polg	677.172927031255	0.250731312458528	0.143966551792817	1.74159420598861	0.0815794759256612	0.414825771706955
Cyp2d1	5582.95546520453	-0.363004265553867	0.208636105441509	-1.73989187914378	0.0818780049161867	0.415829045521616
Phf13	217.643417732935	-0.351759027710794	0.202169474663426	-1.73992155985173	0.0818727923711778	0.415829045521616
Ltbp1	382.027954849124	-1.17539481627376	0.676087241549251	-1.73852536187541	0.0821182852103651	0.416491044043289
Rnf2	346.592102723911	0.285650144371003	0.164282504528069	1.73877398078136	0.0820745270315973	0.416491044043289
Slc5a6	2543.37233566525	0.148488432874912	0.0854032682178618	1.73867389355781	0.0820921406121113	0.416491044043289
Nlrp3	15.1584631395296	-1.14793955864136	0.660398315304409	-1.73825331173387	0.0821661890808054	0.416548128943994
Smtn	963.521029527445	-0.487346320232507	0.280448849772504	-1.73773691932712	0.0822571802184861	0.416823499939061
Htra3	59.9552596515391	-1.01540538023465	0.58445132407059	-1.73736518066645	0.0823227331489908	0.416969779399033
Adam1a	150.109950345337	-0.370413777840102	0.213266708118063	-1.73685701396508	0.0824124125171211	0.417182184843742
Mllt10	940.905985876723	-0.245965928161203	0.141627382839994	-1.73671166711516	0.0824380773499072	0.417182184843742
Cic	1203.36101568061	-0.155023315493415	0.0893655034279129	-1.73471092923978	0.0827920194911419	0.41756673019135
Ecsit	1577.2999356091	0.200385692919224	0.115444577118213	1.73577397848695	0.0826038068749581	0.41756673019135
Frem2	135.150174781846	-0.715384492106926	0.412610181670281	-1.73380232453545	0.0829531630799285	0.41756673019135
Glyctk	5751.68740324281	0.246653213687306	0.14230075552337	1.73332328967711	0.0830382235172668	0.41756673019135
Marc2	1216.32393816032	0.258271690464509	0.148781814740111	1.73590899476292	0.0825799271170769	0.41756673019135
Mettl7a	1258.82234903142	-0.311800637985175	0.179773074658154	-1.73441233387189	0.082844948205717	0.41756673019135
Phf8	466.271519693344	-0.329181502620242	0.189836881849638	-1.73402291173837	0.0829140179796296	0.41756673019135
Plch1	86.21539030958	-0.437898399943344	0.252603560471636	-1.73354009391532	0.0829997176450593	0.41756673019135
Rbm25	2043.59736256238	-0.227824640454451	0.131463790869311	-1.73298395663132	0.0830985203741253	0.41756673019135
Rfx5	391.605029912096	-0.303481347032473	0.175051879707213	-1.73366517137701	0.0829775096393019	0.41756673019135
Sdc3	255.77731466537	-0.635945884657376	0.366850989601253	-1.73352642539853	0.0830021448370015	0.41756673019135
Son	2146.83728074213	-0.156849113829634	0.0904074108479376	-1.73491434339879	0.0827559781983869	0.41756673019135
Tjp1	1135.95303541465	-0.226007890046669	0.130384136946373	-1.73340020756994	0.0830245607324756	0.41756673019135
Ugt1a9	19.2253526286652	-1.25476441298189	0.723054262930933	-1.73536686983304	0.0826758443467641	0.41756673019135
Wwtr1	349.953503805619	0.255180397918728	0.147250745380026	1.73296506758018	0.0831018778543757	0.41756673019135
Zfp263	252.656063690958	-0.291747195156906	0.168239815717805	-1.73411504234089	0.0828976730423567	0.41756673019135
tGap1	82.8737630746888	-0.433229773116702	0.250032346991874	-1.73269490259507	0.0831499110142532	0.417623459384888
Pepd	13709.2379725251	0.289313575792351	0.167021734230667	1.73219118532676	0.0832395279532405	0.417888902259952
Aknad1	32.0740144842038	-0.725515310307567	0.41901508770363	-1.73147777156112	0.0833665861124242	0.418341994593295
Acox3	654.464683501742	0.28441026851998	0.164301917570755	1.73102221036162	0.0834478031837802	0.418467803544236
LOC499136	499.654193713679	-0.304086753796072	0.175692503145584	-1.73078958038464	0.0834893009729228	0.418467803544236
Mau2	746.599714139783	-0.213614573505495	0.12342542153377	-1.73071779582335	0.0835021096637627	0.418467803544236
Sfswap	654.369946750918	-0.262883379586071	0.151940683056246	-1.73017110558043	0.0835997091307518	0.418599682517556
Sostdc1	2047.39446907232	-0.345474235272795	0.199677874840334	-1.73015781317305	0.0836020833463709	0.418599682517556
Irf9	196.340077268078	-0.425505163814587	0.246028568181955	-1.72949494019693	0.0837205512915741	0.419008272118023
Hist1h1d	437.970311945054	0.876159170307138	0.506822083522473	1.72873124276221	0.0838572069523692	0.419322927505776
Shisa2	41.0251570028143	-1.29355783814984	0.74824537628214	-1.72878828142879	0.0838469942437379	0.419322927505776
Nfs1	7552.01440684054	0.212448581352894	0.122944052255786	1.72801024087683	0.0839863883670842	0.419599687991332
Wfdc10	50.3427763408064	0.777062239782871	0.449670705007923	1.72806952093795	0.0839757611246556	0.419599687991332
LOC689064	796.647480078501	0.505019543913379	0.292315258872808	1.72765371832034	0.0840503257679307	0.419713424806648
Rhoh	27.8114628732991	-0.668746468373289	0.38712444392217	-1.72747156340181	0.0840830079434047	0.419713424806648
Dusp16	144.717223706485	0.488179370790479	0.282653327841921	1.72713116281972	0.0841441100757091	0.419834045268002
Foxc1	740.870863252168	-0.434214050755851	0.251483601513418	-1.72660979937765	0.0842377648328536	0.420116908771485
Grip1	44.2741679696526	-0.633882332059598	0.367263888388063	-1.72595877814651	0.0843548289991435	0.420506093014267
Wdr31	421.069445000261	0.304075313220531	0.176197458125416	1.72576447160828	0.0843897939614238	0.420506093014267
Wdr5	613.891801931923	-0.210094751897966	0.121832911232053	-1.72444990252102	0.0846266554625343	0.421501562658705
Ddx54	979.388348561406	0.163504968102289	0.0948428683951957	1.72395638036788	0.0847157179454463	0.421510703252493
Prom2	972.394596230489	-0.771747470356256	0.447695229970924	-1.72382330364873	0.0847397463427721	0.421510703252493
Tma7	733.042369704872	-0.172717530045283	0.10018440194969	-1.7239962178146	0.0847085259486641	0.421510703252493
Rnpc3	166.958090294771	-0.389920699681526	0.22625502188702	-1.72336815523252	0.0848219698387468	0.421735130878039
Akip1	227.008842819171	-0.29146550719476	0.169346369012642	-1.72112049932999	0.0852289601707305	0.422991799429959
Anp32e	1363.24002052358	0.195685915354911	0.113725968815838	1.72067925551635	0.085309042727108	0.422991799429959
Ass1	9085.88341032567	0.391908888232215	0.227841201952513	1.72009665009534	0.0854148745725047	0.422991799429959
Inha	182.512756813407	-0.475304639417098	0.27618155036261	-1.72098620922741	0.0852533264146651	0.422991799429959
P2ry1	34.5363639502758	0.731924184270393	0.42525878565045	1.7211265445131	0.0852278634360072	0.422991799429959
Pgm3	1084.18368149078	0.315304303743977	0.183317084225708	1.71999410243598	0.0854335136116498	0.422991799429959
Qprt	3227.37969687605	0.258076281387136	0.149906620852674	1.72158027390111	0.0851455790932859	0.422991799429959
Rad50	321.529420203524	-0.286414130654591	0.166527530098163	-1.71992060703572	0.0854468741414029	0.422991799429959
Robo2	100.68095389687	0.747355489956454	0.434444085375912	1.72025702527355	0.085385731407544	0.422991799429959
Sp5	34.4944090167697	-1.27906719118113	0.743370109215712	-1.72063306732981	0.0853174290646395	0.422991799429959
Cspp1	730.495141318604	-0.260854242713091	0.151694188629033	-1.71960603811269	0.0855040778605232	0.423000132735281
LOC257650	148.957073950814	-0.360792846979743	0.209824025295951	-1.71950207546945	0.0855229900603269	0.423000132735281
Smox	78.0167962227854	-0.452645642335704	0.263553777391609	-1.71746975822368	0.0858933754220082	0.424647283621812
Arhgdib	633.7079307025	-1.03313005163082	0.601943186632871	-1.71632485352962	0.0861026018912428	0.425496596998202
Angptl4	119.235899817577	-0.848570109854943	0.494643398739905	-1.71551892134144	0.086250129353507	0.425855330248462
Ttll3	316.147886983466	-0.271739143713733	0.158393644235356	-1.71559373499834	0.0862364259759765	0.425855330248462
Smoc2	186.365086362244	-1.89777713198633	1.10672528264912	-1.71476802937374	0.0863877652700177	0.426349691732098
Cxcl9	20.9268577779095	-0.912934430284702	0.5327636370614	-1.71358247218266	0.0866054349125501	0.427238443236131
C1qtnf7	28.9142349986835	-1.2850854068247	0.750209230887291	-1.71296933430797	0.0867181813460467	0.427609045197036
Anapc5	6259.50134789142	0.160431955503107	0.0937001832689662	1.71218400974294	0.0868627632793319	0.427980910461837
Tgfbr3	189.439869667029	-0.76993923844525	0.449691284501937	-1.71215067976692	0.0868689037863328	0.427980910461837

Calcrl	44.6245782428598	-1.08330457529374	0.633200025286667	-1.7108410171072	0.0871104654162772	0.428985073622793
Dynll2	6150.59576057649	0.239076752751022	0.139834735879216	1.70970933114593	0.0873196363882708	0.429828924724593
Zcchc4	137.237115104504	-0.347102134649981	0.203051755267289	-1.70942691036121	0.087371899728686	0.429900005331708
Nexn	38.582520978948	-0.958710745013401	0.561265624647225	-1.70812303998839	0.0876135146333527	0.430902296548112
Nab1	685.012159478624	-0.285834608316636	0.167360122364464	-1.70790152563445	0.0876546160694696	0.430917978479927
Zdhhc8	242.820625442678	-0.279388521400938	0.163606498642099	-1.70768596430952	0.0876946278671586	0.430928292407317
Heyl	335.846183886544	0.534741546310366	0.31324398652554	1.70710873731894	0.0878018434289641	0.431082398450802
Trmt13	130.59995033108	-0.37410234550347	0.219120365614863	-1.70729153565311	0.087767878583149	0.431082398450802
Odf2l	218.32096105762	-0.33308575470238	0.195175493613648	-1.7065961947135	0.0878971329491516	0.431363908937848
Afap1l1	166.189994194673	-0.818594422893426	0.480194617606856	-1.70471386575104	0.0882478025552809	0.432139759620242
Guca2b	169.526165798733	0.769803169244457	0.451606632513537	1.70458782892517	0.0882713228940415	0.432139759620242
Hexdc	981.526496320236	-0.213118910028116	0.125013223372925	-1.70477093765005	0.0882371537556128	0.432139759620242
Nfkbia	1395.34488303135	-0.416698352682922	0.24434956048976	-1.70533702556173	0.088131585943016	0.432139759620242
Slc30a2	84.1070154363186	0.803165471945082	0.471036376185824	1.70510285946203	0.0881752424270315	0.432139759620242
Ush1c	5101.98315330838	0.178569761731887	0.104762281954615	1.70452340670898	0.0882833469855888	0.432139759620242
St6galnac2	807.328342988864	-0.329852846071393	0.193605136061629	-1.7037401630006	0.0884296412383078	0.432669523165995
Egfl6	687.972367667104	-0.219223450427785	0.128737276274154	-1.70287469777545	0.0885915200210348	0.43327505015451
Mef2b	20.5812167777978	0.774566790542299	0.455039137750706	1.70219817655915	0.0887182244866899	0.433708103017513
Ralgps2	72.6971533814002	0.447952975533852	0.263218555728293	1.70182901541429	0.0887874255877192	0.433859793306112
Tubgcp3	445.333664613422	-0.220384530418405	0.1295171893502	-1.70158518358911	0.0888331569292154	0.433896717515025
Atad3a	859.038017874293	0.263823225601214	0.15507599222439	1.70125124989992	0.0888958179214064	0.43401626567642
Gng13	1816.420317853	-0.306602367129227	0.180306858097369	-1.70044761671603	0.0890467616678096	0.434193690740594
Mon1a	547.01533070962	0.22962969126062	0.135020836219705	1.70069818621896	0.0889996758948749	0.434193690740594
Pak4	574.389759544583	0.176299221602153	0.103667702562362	1.70061858461751	0.0890146320568535	0.434193690740594
Ppm1b	807.574548722086	0.199957490867996	0.117640967995386	1.69972667069382	0.0891823502011451	0.434482037027953
Tyro3	1708.14506395218	0.206368452195947	0.121406173064732	1.69981844404167	0.0891650811283546	0.434482037027953
Ociad2	670.76074875207	-0.237668768164647	0.139907598908513	-1.69875525002799	0.0893653085280064	0.435016198124261
Usp3	545.499939877979	0.176227240376996	0.103740087726455	1.69873810827765	0.0893685397343085	0.435016198124261
H19	5.68122302580037	2.32316150137059	1.36833142220755	1.69780614817907	0.0895443551386894	0.435428298590301
Hivep2	375.093516639899	-0.291388888910449	0.171639442154248	-1.69768023743975	0.0895681296941866	0.435428298590301
MGC108823	20.8280173555766	-0.935381979186503	0.550895369013637	-1.69793037262462	0.0895209039705435	0.435428298590301
Cd59	7566.07484102407	-0.23476956329262	0.138392400046369	-1.69640502812263	0.0898092021513419	0.436088463845233
Luc7l3	1838.28950274478	-0.280649416150497	0.165431314236273	-1.69647093385032	0.0897967301892952	0.436088463845233
Ralb	1159.12897034171	0.295329246246328	0.174096564077787	1.6963531004229	0.0898190299016093	0.436088463845233
Ube2v1	2799.67903353803	0.190513230513298	0.112331372617821	1.69599307899024	0.0898871907716562	0.436128316980051
Zfp142	716.145671924198	-0.34549598908302	0.203723737783549	-1.69590442842798	0.0899039808801917	0.436128316980051
Pxk	2058.23912379406	-0.186260715979502	0.109862460785396	-1.69539908944278	0.0899997385459913	0.43640658204511
Creld2	859.74967707303	-0.281258746522453	0.165970519710672	-1.69463075136932	0.0901454896994759	0.436926923635072
Tmem40	14.5285173282582	-1.01120847632324	0.597111415732949	-1.69350049200114	0.0903602412962484	0.437781117891372
Gba3	2577.67337894982	0.436004657185447	0.257504623027475	1.6931915709293	0.0904190085080509	0.437879186494464
Gmfb	351.984136178113	0.383372863307616	0.226494101827557	1.69263950016456	0.0905241075750802	0.438201450893681
Efnb1	791.886671062254	0.297503842701689	0.17583420035697	1.69195663925284	0.0906542414204879	0.438506443804435
Wnk1	801.01268666487	0.242958867537998	0.1436008567421	1.69190402515731	0.09066427440968	0.438506443804435
Arglu1	1222.24326809517	-0.189517884165856	0.112095826435304	-1.69067743369764	0.0908984265388617	0.438779202254696
B3galnt1	188.832102723035	0.478013262721803	0.282705479849274	1.69085248356932	0.0908649803952625	0.438779202254696
Bsx	27.1943553042919	-1.40529754954566	0.831192383464755	-1.69070070599998	0.0908939794139757	0.438779202254696
Dalrd3	833.62605588904	-0.186757315994995	0.110477844182793	-1.69045040095091	0.0909418196082957	0.438779202254696
Igfbp5	1255.65001973256	-1.53620878898981	0.908895103433818	-1.69019371232829	0.0909909009074713	0.438779202254696
Lum	12.9966729839343	1.7967471082011	1.06297828073707	1.69029522122995	0.0909714888971118	0.438779202254696
Pex16	670.125555676668	0.23279773651078	0.137663549207985	1.69106301450258	0.090824768124579	0.438779202254696
Cmah	102.10370416802	-0.525023354032334	0.310760130309733	-1.68948105894101	0.0911272786645772	0.438793223378235
Gatad2a	1676.15393472782	0.152757337117084	0.0903941457285533	1.6899029896892	0.0910465155555874	0.438793223378235
Gpr68	52.5744940341144	-0.709062545347351	0.419680639573373	-1.68952884285572	0.0911181293018591	0.438793223378235
Tomm70a	1074.35835608145	0.203062821366793	0.120200207061437	1.68937164361958	0.0911482316026758	0.438793223378235
Scd1	3256.01362180462	2.78638117169105	1.65000768935807	1.68870799188523	0.091275403341072	0.439219404900349
Col18a1	6152.71338871853	-0.423263177131824	0.250773110236524	-1.68783318407867	0.0914432555913496	0.439468939979399
RGD1562310	152.483796282203	-0.7386161187514	0.437602150812371	-1.68787131731465	0.0914359336736488	0.439468939979399
Spag8	103.758277032956	-0.482605693063148	0.285903946676263	-1.68799940914987	0.0914113423613315	0.439468939979399
Cdc42ep1	715.993887568911	0.275079540613031	0.163053363282937	1.68705223292881	0.0915933087836766	0.440004035348803
LOC303448	293.053924417026	-0.328336034439895	0.194664292880421	-1.68667827869996	0.0916652309757936	0.440163504550431
Pdgfd	24.5927672509449	-0.841030942540149	0.4987742477889	-1.68619560105297	0.0917581309085955	0.440423528676983
Bhlha15	73.9179434482651	0.930045527603162	0.551696639971864	1.68579153871699	0.0918359580836601	0.440611017129118
Tmem50b	1789.59495246411	-0.228596182906078	0.135663007588264	-1.68502959627627	0.0919828614967607	0.441129621844803
Cln8	307.130549008298	0.347554736911681	0.206410407402345	1.68380432598154	0.0922194909100195	0.441705323928901
Hspb2	203.372192003562	-0.377831643594146	0.224358653499738	-1.68405201983701	0.0921716158291755	0.441705323928901
Usp54	563.970913179645	-0.337999045382867	0.200725693001364	-1.68388530799876	0.0922038362430033	0.441705323928901
Defb1	3611.44469960768	0.348742007906845	0.207314945252915	1.68218459832404	0.0925330497310265	0.44208938345643
Idh3B	5075.36753408871	0.189987879439364	0.11289781613328	1.68283042087436	0.0924079241732499	0.44208938345643
Pex19	769.790942939869	0.218298769839468	0.129731276685427	1.68269961891148	0.0924332555556521	0.44208938345643
Snrnp70	1654.27348431201	-0.225823456769427	0.134233697247034	-1.6823157031415	0.0925076376929852	0.44208938345643
Tpd52	472.30926819598	0.23885052161377	0.141940076586733	1.68275604295464	0.0924223276733107	0.44208938345643
Traf3	769.842300659321	-0.330152170097056	0.196234033270801	-1.68244093338004	0.0924833695574003	0.44208938345643
Agpat1	3444.51178557659	0.196174556896024	0.116648114951341	1.68176362710925	0.0926146844509602	0.442107729302652
Serping1	4608.44953856718	0.168829250525351	0.100383669597977	1.68183979726473	0.0925999092542962	0.442107729302652
LOC294154	9703.08498847532	0.224749970727997	0.133659704874071	1.6815088058121	0.0926641274748468	0.442158049067636
Jmjd1c	1357.86449258436	-0.165699039773956	0.0985580188450279	-1.68123346751217	0.0927175752347636	0.442227427661906
Alg3	152.709526473331	0.300754817106741	0.178973823788012	1.68044024953601	0.0928716904512896	0.442256504004055
Bcl10	647.947743424784	-0.260535089883712	0.155043399315021	-1.68040104277094	0.0928793133079077	0.442256504004055
RGD1307235	766.648082800403	-0.188123384017886	0.111920323990136	-1.68086882981563	0.0927883955872328	0.442256504004055
RGD1560065	306.233271640596	-0.29512769935469	0.175625310426527	-1.68043944598837	0.0928718466776645	0.442256504004055
Plekhg2	368.903329136801	-0.481318266390122	0.286603396383239	-1.6793878665224	0.0930764764486709	0.443009728356614
Pddc1	165.888056967424	0.360683202017594	0.214975743805754	1.67778557539727	0.0933889662988034	0.444136958964666
Plekha8	385.587238759759	-0.25840368133547	0.154015895478807	-1.67777280735953	0.093391459785813	0.444136958964666
Asl	1754.34975041889	0.272986577865577	0.162824067556953	1.67657387486706	0.0936258390049261	0.445065364337093
Cbx7	852.379793025328	-0.167429009079562	0.0999268602782799	-1.67551555821227	0.0938331211011363	0.445545737039028
Cplx2	16.2410679436795	1.08385245405995	0.64691620109447	1.67541399060072	0.0938530334929748	0.445545737039028
Spta1	151.314321669255	-1.59600833777255	0.952807919520372	-1.67505780029196	0.0939228916017519	0.445545737039028
Tmem143	310.048847715129	0.260652947283499	0.155601863672509	1.67512741256164	0.0939092355623887	0.445545737039028
Wdfy3	2932.70444678084	-0.232995925564855	0.139060739105502	-1.67549753484402	0.0938366543461144	0.445545737039028
Trim8	1723.79599489662	0.181545775271096	0.108408509248331	1.67464506734642	0.0940038913268107	0.445743941935974
Uhrf2	760.672379878772	0.193577504909209	0.115619627096603	1.67426162642326	0.0940791926129889	0.445914972159813
LOC100363776	313.201735041997	-0.309882687628286	0.185172704954335	-1.6734792943955	0.0942329793430032	0.446106655386986
Pzp	3576.92129714175	0.247862914795508	0.148114309156799	1.67345691450454	0.0942373816280269	0.446106655386986
Timm44	1856.37379195162	0.191534811617019	0.114449239694085	1.67353502853299	0.0942220167550754	0.446106655386986
Acad10	1914.34863235552	0.402073802589832	0.240415562632212	1.67241171157013	0.0944431642672891	0.446894673214825
Apobr	64.3025364847	-0.62561991005136	0.37415183273961	-1.67210168521815	0.0945042723853276	0.446929861927725
Hhex	22.3819601717512	-0.851075005281341	0.50904399463126	-1.67190854672167	0.0945423571995611	0.446929861927725
Prrx1	13.4956337243613	-1.36111497050607	0.814173212941096	-1.67177567238944	0.0945685657167147	0.446929861927725
Slc6a9	65.660897488546	-0.666643569464988	0.398885670319115	-1.67126477351684	0.0946693911268583	0.447220407127129
Myo1g	37.7002338159695	-0.814195634899998	0.487664301567214	-1.66958219472577	0.0950020556155402	0.448605469101051
Rhobtb1	4223.87424528321	0.43514227368479	0.260746980676543	1.66882957783732	0.095151159140365	0.449122954646756
Cd300a	23.7446778155878	-0.951810747304488	0.570528672015981	-1.66829608044278	0.0952569656344306	0.449153432139937
Klhl36	273.767287167911	0.243536722829625	0.146040057852022	1.66760220730941	0.0953947197946339	0.449153432139937
Lhx1	98.3046996016715	0.597344758267327	0.358092685936525	1.66812889993853	0.0952901412881676	0.449153432139937
Ptger1	354.984677229013	-0.364697305070289	0.218614459098461	-1.66822133620189	0.0952717968964733	0.449153432139937

RT1-CE1	420.344490636977	-0.868964553884864	0.52102459968488	-1.66779947513116	0.095355540127798	0.449153432139937
Ufl1	614.111345254361	0.2244463897327	0.134571812546247	1.66785588665209	0.0953443385183286	0.449153432139937
Asb8	879.692007157671	0.172000182695208	0.103169475443215	1.66716155099459	0.0954822857895276	0.449379569475682
Aox1	51.0578124943707	-0.774648403706135	0.464987329496889	-1.66595594022808	0.0957221903804299	0.449637382578017
Bace2	92.6156714660909	-0.714164759821712	0.428503571520455	-1.6666483252115	0.0955843537123677	0.449637382578017
Dpagt1	655.110903496682	-0.172741488544813	0.103705326581065	-1.66569542992359	0.0957740926989943	0.449637382578017
Gucy1a3	30.1558493081163	-0.948099660779547	0.569222923983877	-1.66560344081716	0.0957924253703656	0.449637382578017
Irs2	775.298882114023	-0.359591753446122	0.215827702959835	-1.66610564128109	0.0956923751402958	0.449637382578017
Stim1	292.663845751374	-0.421113797584757	0.25284598629935	-1.66549528330733	0.0958139838645043	0.449637382578017
Tmbim4	961.708074382581	-0.195144120863105	0.117127908547879	-1.66607705441386	0.0956980680750123	0.449637382578017
Umps	692.962233096083	0.20373095269549	0.12235028431028	1.66514490623356	0.0958838494420835	0.449779543028775
Epha2	379.398694263349	-0.419978975034889	0.252263158928401	-1.66484466784185	0.0959437497491675	0.449874859591187
Ndufa12	1846.70731772173	0.200927540855821	0.120762538023691	1.66382343518155	0.0961477192493779	0.450006125213677
Rcan1	1048.58847463646	0.274790958879321	0.165186897903246	1.66351546258997	0.0962092982816502	0.450006125213677
Steap2	836.362060430461	0.243065247989098	0.146085128616687	1.66386031412463	0.096140347430317	0.450006125213677
Tmem72	443.106819139189	-0.37556325209462	0.225738446054063	-1.66370974310984	0.0961704482730838	0.450006125213677
Ttc9	95.8379349028589	-0.437452717981315	0.262814996690422	-1.66448917866207	0.0960147118175511	0.450006125213677
Wasf1	38.21246109221	-0.620758961137863	0.373052721122509	-1.66399794449967	0.0961128401632968	0.450006125213677
Arhgap17	295.737715537703	-0.26306714005965	0.158190691215274	-1.66297484408646	0.0963174711207348	0.450141602285473
Trafd1	1014.38806833804	-0.215918570300261	0.129827993100445	-1.66311259339278	0.0962898995042364	0.450141602285473
Ctsd	8611.35791540409	0.284934603358005	0.171447096873567	1.66193892199953	0.0965250215819225	0.450740918364886
Dlg2	15.5842036739257	-0.926642165025442	0.557515098660217	-1.66209339846093	0.0964940490259236	0.450740918364886
Abcd4	156.089150112078	0.345531359652028	0.207934442025415	1.66173220889397	0.0965664800350227	0.450749327342122
Cdc34	1113.13801181833	0.219626933381235	0.132275968123111	1.66036912447184	0.0968402175008472	0.451841507436219
Ces1f	16242.9106737832	0.29825758448268	0.179669828511635	1.66003155317403	0.0969081050613075	0.451972721430784
Exoc3l1	138.283531820654	-0.653265172732542	0.393883162440945	-1.65852525577426	0.0972114941997457	0.453114530681102
RGD1303003	4112.09280261351	0.207131058525722	0.124896597859066	1.65842034191716	0.0972326535572065	0.453114530681102
Nrg1	37.9202315533465	0.638557698406337	0.385195933239034	1.65774776757594	0.0973683879475517	0.453450707768443
Psmd3	3020.99531397963	0.158416951087404	0.0955661763956595	1.65766756672918	0.0973845836409263	0.453450707768443
Tmem167b	243.159717750672	0.283636430971955	0.171185435413726	1.65689581176374	0.0975405412898768	0.453990905938059
Cachd1	531.998058402072	-0.228803073367348	0.138132329106107	-1.65640494769036	0.0976398398109801	0.454081186289525
Pxmp2	1155.38501599778	0.229487882185334	0.138544235957669	1.65642316765493	0.0976361525908213	0.454081186289525
Angel1	282.348941160579	0.252972453352282	0.152798116988296	1.65559928576646	0.0978029949293038	0.454653922440273
RGD1563714	59.9249384213519	-0.811066866558718	0.490080668402349	-1.65496604712603	0.097931385335928	0.4550646466591
Dhx32	458.455223442659	0.208071609018399	0.125809700445668	1.6538598238556	0.0981559973853149	0.455549639151282
Emilin1	644.801184528517	-0.491029397526192	0.296838333885246	-1.65419806498448	0.0980872759048196	0.455549639151282
Pcbd1	2788.39150305556	0.362227159625297	0.218997345838366	1.65402534098584	0.0981223639620769	0.455549639151282
Bcl7c	737.810048324052	0.234777529715081	0.142033812765653	1.65296928346526	0.0983371151258806	0.456203938988065
Gng11	137.82742721408	-0.612449568957809	0.370624888987045	-1.65247825269289	0.0984370948629996	0.456295277411441
LOC298795	222.63990483378	-0.517769199091508	0.313291665304024	-1.65267466847245	0.0983970925247852	0.456295277411441
Evc	2557.22898049005	0.27254160970534	0.165004618970929	1.65172109365833	0.0985914205685732	0.456638176928445
Ndufv1	6901.89565253251	0.181294680583605	0.109757798044186	1.65177038729056	0.0985813675639143	0.456638176928445
Cyp2c11	1646.43678184105	-1.11834471829208	0.677743597836999	-1.65010001106797	0.0989224825063383	0.457041693449379
Gba2	816.845770680018	-0.233224996212277	0.141272654581879	-1.65088563602452	0.0987619293731899	0.457041693449379
MGC112715	147.1438559669	-0.636278006349649	0.385518401939733	-1.65044782077385	0.0988513771858353	0.457041693449379
Naa60	1070.79423607215	0.160532976703834	0.0972959001955007	1.64994595230907	0.0989539909262837	0.457041693449379
Nkx1-2	23.4949925324816	1.04992638246656	0.636195087645152	1.65032142318611	0.098877212862301	0.457041693449379
Slc2a13	728.894043443032	-0.37068040327731	0.224534779323584	-1.65088190076385	0.0987626922319678	0.457041693449379
Vof16	43.3958962805132	-0.544064446117909	0.329752721587759	-1.64991646922045	0.0989600217824144	0.457041693449379
Atp2b2	78.820394602377	0.474636796114587	0.287824253655027	1.64905073178255	0.0991372418535532	0.457271765562536
Atp2b3	47.5780122940961	-0.877907972121473	0.532370431255999	-1.64905471938076	0.0991364249953047	0.457271765562536
Coa4	426.236757948321	0.204014951462909	0.12375822810783	1.64849606027933	0.0992509184866604	0.457271765562536
Hn1	974.147857894223	-0.219105213659332	0.132840524062747	-1.64938534536221	0.0990687150502035	0.457271765562536
Mertk	178.749507188013	0.425098068882766	0.257828260859253	1.64876444291274	0.0991959020770039	0.457271765562536
Phactr2	122.197905395875	-0.362946914549925	0.220168706819252	-1.64849455580391	0.0992512269613563	0.457271765562536
Agfg2	626.985258781536	-0.331728722394501	0.201255681422944	-1.64829494526103	0.0992921615013778	0.457275003089409
Elf2	306.715594304954	-0.228700634860707	0.138785317725603	-1.64787341059289	0.0993786507363311	0.457302730473336
Zdhhc5	1059.80098383251	0.217261334407665	0.131835986549582	1.64796684193625	0.0993594755867578	0.457302730473336
Aldh8a1	9454.88791672656	0.300089424256921	0.182150719687701	1.64747866366615	0.0994596982332546	0.457445180725047
Rffl	183.734925747648	0.369181499563401	0.224108923828988	1.64733065179107	0.0994901008932181	0.457445180725047
Sec23ip	541.59294283851	-0.214857132672469	0.130463722146655	-1.64687262587026	0.0995842295500508	0.457507822581195
Sncg	122.120421016768	0.816547856588901	0.495763044117512	1.64705269236517	0.0995472157118453	0.457507822581195
Axl	53.6407944403758	-0.853981645537836	0.51889203881955	-1.64577904775836	0.0998092575013717	0.457616789334647
Dleu7	119.657303557488	0.594617709787331	0.361251612702927	1.64599323263454	0.099765152305012	0.457616789334647
Lage3	1444.02228779489	0.224796940033475	0.136571813836787	1.64599805566121	0.0997641593207979	0.457616789334647
LOC498750	788.350519407036	-0.179924553194794	0.109324328987428	-1.64578694295472	0.0998076314374354	0.457616789334647
Pafah1b1	2500.71570172757	0.148085402412566	0.0899596865195375	1.64613070745199	0.0997368515240913	0.457616789334647
Tmem178a	515.935989419919	0.229967069485214	0.139791345581968	1.64507372418395	0.0999546084793237	0.458098419345159
Slc29a3	1769.98238985398	0.233870207775203	0.142180946022561	1.64487728009696	0.0999951211302965	0.458099373948791
Etfdh	2802.88682961954	0.280771878809587	0.170778535345776	1.64407007145897	0.100161729177086	0.45830845967261
Ggt6	16.1409097815511	1.111760537437	0.676219417727354	1.64408253932343	0.100159154125186	0.45830845967261
Piwil2	18.3146208267163	-0.930331717512323	0.565765729889965	-1.64437622917398	0.100098512120483	0.45830845967261
Cmpk2	83.8638685609489	-0.758435143529699	0.461480747785666	-1.64348165588471	0.100283317700362	0.458667457671385
Tgfb1	152.207115669887	-0.665793634103964	0.405156494875181	-1.64329991626835	0.100320895633562	0.458667457671385
Dnajc11	929.111845615501	0.164034027189292	0.099853793928719	1.64274206052088	0.100436312453534	0.458870470292464
Rassf5	185.342631617136	-0.469450318465632	0.285780564367124	-1.64269504997743	0.100446043470671	0.458870470292464
B3galt5	1868.91443563125	0.225861036771352	0.137528110116044	1.64228997679655	0.100529923333862	0.459069147694926
Eml4	555.801375129047	-0.213516888617031	0.13006020902271	-1.6416772679471	0.100656905090652	0.459095659414265
Mcc	37.0816886354768	-0.78276549785622	0.476789485127112	-1.64174236696419	0.100643407481959	0.459095659414265
Zfp111	196.933660673868	-0.280494608448481	0.170839125740346	-1.64186398890145	0.100618194294505	0.459095659414265
Pygm	43.2639138513014	-0.707570344128977	0.431136252029748	-1.641175709066	0.100760946523961	0.459232579631565
Tmlhe	6874.23007668851	0.31079526055518	0.189377317622254	1.64114300729043	0.100767733028429	0.459232579631565
Brd9	559.990769205718	-0.185125351899535	0.112903886036197	-1.63967209986186	0.101073362996806	0.460056535733546
Gdpd1	65.5946554231597	-0.523562632261052	0.319290499410566	-1.63976890395295	0.101053226050701	0.460056535733546
Ica1	358.58294816192	-0.275571779597482	0.168083441535294	-1.63949391492926	0.101110436940208	0.460056535733546
Nipal4	24.7107580768885	-0.970440332298337	0.591800090682947	-1.63981105710618	0.101044458455609	0.460056535733546
Baat	20.4671716787875	-0.761919912331794	0.4649177632888	-1.63882727762867	0.101249236526738	0.460062705048436
Dclk3	1194.83941646933	0.185861834167454	0.113419611114833	1.63870985220779	0.101273701216891	0.460062705048436
Scx	78.318908526215	-0.598621809266282	0.365294444260805	-1.63873778720514	0.101267880746647	0.460062705048436
Uqcrc2	8402.39674623331	0.169168408189233	0.103196036980705	1.63929171253798	0.101152521182748	0.460062705048436
Grpel1	3082.77664858201	0.2152970617835	0.131411044069287	1.63834830860931	0.101349055632267	0.460191408207918
Lrrc16b	44.0687763351553	-0.550439999439923	0.33600590971078	-1.6381854709452	0.101383009515452	0.460191408207918
Ahi1	197.300370048333	0.368579988173293	0.225036387683859	1.6378683997145	0.101449149203112	0.460307796344338
Psat1	3446.63642983278	0.261226438912888	0.159525897031857	1.63751744245464	0.101522397430884	0.460456332481815
Il21r	18.8399658535053	-0.852047851744827	0.521036562708286	-1.63529378306195	0.101987476215805	0.462241509746894
Oxct1	21364.1341833505	0.216246259659334	0.132240753090939	1.63524673449663	0.101997334721556	0.462241509746894
Cdc6	66.6893864485796	-0.425622984756422	0.260483122815074	-1.63397528468125	0.102264040287031	0.462847915578593
Htatsf1	1114.01057060716	-0.212879135908796	0.130322938523629	-1.63347403243366	0.102369337820754	0.462847915578593
Kidins220	1594.82831909874	-0.151119959822367	0.092516109187135	-1.63344482544864	0.102375475960284	0.462847915578593
Lman2	2183.8015478195	0.226502358704864	0.138620538690031	1.6339740188959	0.102264306080642	0.462847915578593
Rsph10b	98.9208753688091	-0.383932595036426	0.234997700501099	-1.63377171018161	0.102306794570007	0.462847915578593
Wdr86	46.7472353216895	-1.3317573297572	0.814957388697953	-1.63414351256442	0.102228720155564	0.462847915578593
Cltb	1002.86042109378	0.149694012542009	0.0916892161429951	1.6326239751963	0.102548105638875	0.463444043217276
Tmem110	216.545336819624	0.291470239681353	0.178590692373334	1.63205728029796	0.102667420008084	0.463798845712193
Adcy5	118.959718600384	-0.719723438986117	0.44110533840559	-1.63163620188233	0.102756147150343	0.464015243746841

Plcb4	307.575859957344	-0.334133116334282	0.204834079355556	-1.63123791404987	0.102840128115033	0.464210046130052
Tmcc3	355.968375126861	0.300108805035925	0.184022432622386	1.63082728969108	0.102926767440095	0.464416688655862
Dpysl5	17.7026011725902	-0.891668865987448	0.546973611334551	-1.63018626037896	0.103062136887576	0.464686823264398
Znfx1	420.420915970606	-0.337846916322136	0.207248135920757	-1.63015659861623	0.103068404139499	0.464686823264398
Pwwp2a	271.557375326586	-0.303635255266895	0.186453745564963	-1.62847495686861	0.103424215148562	0.466106117913779
Galm	5926.94830088321	0.154636473285131	0.0950131614160761	1.62752687080852	0.103625246425027	0.466642056603351
Lgalsl	1286.00962565348	0.247095502826362	0.151815552670015	1.62760335473301	0.103609017337948	0.466642056603351
Prkca	284.865460100256	0.308909571563158	0.189831972565402	1.62727894246966	0.103677868109625	0.466694118389702
Synj2	29.6289775203311	-0.866221096851602	0.532496475621852	-1.6267170516763	0.103797205529383	0.467046333351929
Triqk	39.0977021854178	-0.547726245955239	0.336779682115865	-1.62636368831419	0.103872310650737	0.467199320481314
Fam57b	16.689660571142	1.2455494946747	0.766188220428269	1.62564427573486	0.104025350635413	0.467604273065159
Scrn2	1331.64801045533	-0.283471280632325	0.174384442230055	-1.62555373063819	0.104044624897219	0.467604273065159
Homer3	168.720317643163	-0.316428414958129	0.194697465608739	-1.62523129907617	0.104113283685771	0.467646741856759
Ide	1118.08170112897	0.250504608094841	0.15416546547034	1.62490741574699	0.104182287846428	0.467646741856759
Tmem176a	547.586713866897	-0.263058222946224	0.161908207171326	-1.62473680329166	0.104218651867251	0.467646741856759
Usp22	250.241669202426	0.484109974431539	0.297935708999859	1.62488066991583	0.104187987736782	0.467646741856759
Icos	15.5311411687325	-1.25348012002039	0.771612764579223	-1.62449375847734	0.104270471396636	0.467694624267625
Brd4	1330.90223368789	-0.150842554352227	0.0928690110890232	-1.62425067935342	0.104322318709168	0.467742593470771
Selenbp1	6407.0128951829	0.238999749904871	0.147314035318236	1.62238275116536	0.104721420093581	0.469346869394183
Rab33a	21.0306862730805	-0.911103714503373	0.561808573393601	-1.62173337619228	0.104860449131255	0.469784731898241
Mbnl2	1527.13984344113	-0.187040617814599	0.115349322253712	-1.62151466658123	0.104907307109624	0.469809476992903
Dnajc5	196.892947255979	0.297662645795345	0.183596790968442	1.62128457815208	0.104956620911208	0.469845196249227
Dclre1c	121.462861658811	-0.520646909756359	0.321415195317243	-1.61985779559199	0.105262827661928	0.470548897533458
Irs3	37.4710172057363	-0.877820616181047	0.542251235318787	-1.61884484350687	0.105480650828653	0.470548897533458
Osbpl1a	2310.37975080839	0.1430746700403	0.0883724922324399	1.61899553159574	0.105448224541493	0.470548897533458
Procr	48.8834172104417	-0.74616824038643	0.460933972836155	-1.61881806150068	0.105486414826258	0.470548897533458
Rab9a	951.947392249006	-0.259278806470894	0.16015844815297	-1.61888935276928	0.10547107214077	0.470548897533458
Rpap3	1453.95844164105	-0.187774710160562	0.115884815202992	-1.6203564706182	0.105155724796014	0.470548897533458
Sectm1	17.5422076708992	-1.12905938392842	0.696890250380088	-1.62013944564818	0.105202325672224	0.470548897533458
Slc10a2	3718.76762673553	-0.259290369164991	0.160148379477503	-1.61906333370932	0.105433636912155	0.470548897533458
Tmem134	680.67630555508	0.178467654361348	0.110200249565245	1.61948502898521	0.10534294509082	0.470548897533458
Lancl1	585.839699263147	0.322073114676344	0.199054397295363	1.61801557289108	0.105659241612093	0.470793609196076
Postn	49.2789709255012	1.16733657028428	0.721472324957427	1.61799216671708	0.105664285817899	0.470793609196076
Tlr3	374.143561033946	-0.299039128568253	0.184821785907454	-1.61798635967078	0.105665537309448	0.470793609196076
Lxn	141.401976908663	-0.324845647421444	0.200802455487281	-1.61773742573591	0.1057191967618	0.470848115358395
Hoga1	14358.0947645986	0.267762373246623	0.165537306292565	1.61753491852398	0.105762864551731	0.470858095767713
Bub1	71.181771293183	0.497975597590802	0.308067002401166	1.61645224483451	0.105996570455594	0.471529166261558
Spata2L	272.231185565662	-0.345139030018254	0.213507635237993	-1.61651844269426	0.105982269242767	0.471529166261558
Fus	3528.4032925752	-0.156239851697172	0.0966701144481151	-1.61621668277873	0.106047473080235	0.471571040309057
Matn4	38.4921845597279	0.623227547264856	0.385967683176299	1.61471432565556	0.106372573776902	0.472831706510858
Bcl2l2	1829.68642624922	0.19065957211688	0.11811597219696	1.61417265227224	0.106489982121529	0.473065539479116
Notch1	437.878714529193	-0.363163655914986	0.224996245741306	-1.6140876249666	0.106508421214768	0.473065539479116
Ppp1r9b	407.199168449321	-0.487821504389523	0.302323839997574	-1.613572731788	0.106620135456939	0.473240578858862
Rab13	301.841375618872	-0.305915914968314	0.18959510716476	-1.61352220288296	0.106631103506735	0.473240578858862
Clcn5	51.8820837640783	0.716276882442443	0.444275601247064	1.61223546922649	0.10691070963413	0.474155871796491
Glipr1	59.9676987529997	-0.627222267289128	0.389050034084528	-1.61218921048302	0.106920772427803	0.474155871796491
Pofut2	686.828472644382	-0.208857205247289	0.129594242211406	-1.6116241098627	0.107043760937316	0.474516141502937
Scn3a	53.3487926054145	-0.514845455552169	0.319786726380556	-1.60996505821035	0.107405484636546	0.47593401106393
Bgn	2622.40922438035	-0.983776637700081	0.61120300304551	-1.60957428677233	0.107490825405638	0.476126547763245
Kctd9	829.351234578582	0.230159275283978	0.143044288829386	1.609007092611	0.107614790755259	0.476489953924533
Golgb1	1962.0915535533	-0.250301294065487	0.15560352569244	-1.60858369340693	0.107707402209487	0.476528740176347
Stat2	644.828831680237	-0.254515113308427	0.158208937057744	-1.60872778770729	0.107675876923558	0.476528740176347
Cdca3	72.178919610958	0.453541320286039	0.282216427877475	1.60706916920848	0.108039195610939	0.476622421585852
Cpne7	300.364414014167	-0.513096066900212	0.319225804157575	-1.60731388320645	0.107985530315576	0.476622421585852
Gyltl1b	468.022433540082	-0.472716770109094	0.294014766796594	-1.60779941517743	0.107879116585572	0.476622421585852
Igsf5	864.834800330527	0.18926914726848	0.117777755575938	1.60700249671889	0.108053820414794	0.476622421585852
Mapk10	6.61229984405819	-1.99686103170035	1.24242518512066	-1.60722839138715	0.108004276103518	0.476622421585852
Mpdz	650.334069307451	-0.285072465067852	0.177278737099921	-1.60804656966376	0.107824979791417	0.476622421585852
Rasal1	141.137433825668	-0.364356216242368	0.226585347603359	-1.60803079323637	0.107828434822092	0.476622421585852
Stc1	208.764745235139	-0.469008775362477	0.291861639756226	-1.60695587043988	0.10806404895537	0.476622421585852
RGD1312026	95.6247318786703	-0.601165243123193	0.37416568044084	-1.60668194478688	0.108124156290423	0.476702544762197
Rexo1	677.853768841169	-0.174050393037953	0.108351468981626	-1.6063501000385	0.108197008220318	0.476838772947705
S100a4	344.778922981348	-1.0696565943834	0.66640233185485	-1.60512132574047	0.108467106962164	0.477723512351364
Tmem30b	600.21106952975	-0.261140974476726	0.162699137102715	-1.60505445282025	0.108481821694428	0.477723512351364
Actr1a	2291.16372422897	0.122099345392162	0.0760842079382397	1.60479222562551	0.108539537465961	0.47779255725721
A3galt2	113.161065046583	-0.644611973121839	0.401807470794758	-1.60428070649564	0.108652191898425	0.477918271330303
Acaca	125.325273976953	0.561693534861534	0.350092985089389	1.60441242408247	0.108623174231912	0.477918271330303
Arhgap10	289.355705739508	0.277804254119807	0.173284668387337	1.60316695472932	0.108897799029424	0.478302396339005
Oxr1	672.875572529755	-0.263352469090494	0.164271724252072	-1.60315154838446	0.10890119954916	0.478302396339005
Pdp1	74.8369172779097	-0.414961479434278	0.258808188304319	-1.60335529626422	0.10885623469221	0.478302396339005
Pik3r2	922.867159990225	0.177179413391331	0.11052151779921	1.60312142756872	0.108907848119421	0.478302396339005
Exoc5	475.160227325083	0.244043190370555	0.152288552903638	1.60250515037053	0.109043949535176	0.478564246280102
Phactr1	74.8419222069343	-0.610279385473418	0.380836660449587	-1.60247016333188	0.109051680262666	0.478564246280102
Rdh10	252.889110911002	0.390383515570447	0.243697702782774	1.60191709282719	0.109173944177508	0.478915881714224
Ankrd13a	2414.64908567866	-0.224047855441322	0.140190932397598	-1.59816224637052	0.110006873477584	0.479118416083341
Arpc3	1836.09797516403	-0.16028136820738	0.100277470905256	-1.59837864637428	0.109958733979559	0.479118416083341
Atp5b	78815.9087672422	0.196265409341851	0.122745459532801	1.59896268333578	0.109828894489831	0.479118416083341
Cd6	17.0821341903317	-0.907736908144552	0.568079827511572	-1.59790378778423	0.110064390984132	0.479118416083341
Cyld	474.661418175652	0.386559368889553	0.242043686053997	1.59706446051774	0.110251339194239	0.479118416083341
Fam193b	836.607002078341	-0.248316331037818	0.155312084606849	-1.59882169933135	0.109860226083444	0.479118416083341
Fars2	575.109587516552	0.205725618522152	0.128823033899011	1.59696299874003	0.110273955347002	0.479118416083341
Gdap1	28.6208586554818	-0.622627683819051	0.389528917730084	-1.59841196758205	0.109951322953251	0.479118416083341
Igfbp3	248.05882287123	-0.534523263846706	0.334139986053921	-1.599698587886	0.109665465290551	0.479118416083341
Il1r1	45.2046619180537	-0.686782372238197	0.428903149865529	-1.60125280603213	0.109320937252609	0.479118416083341
Irak3	100.603191743303	-0.432309869024338	0.270270273762552	-1.5995464947216	0.109699226272933	0.479118416083341
Itgae	90.2294887949844	-0.3899148442294	0.244081263116781	-1.59747962318126	0.110158836286931	0.479118416083341
Kansl2	876.429301350395	-0.162053728308028	0.101337868556421	-1.59914285366879	0.109788864598067	0.479118416083341
Lpar6	130.337519370994	-0.652094915275605	0.407884228094081	-1.59872549699371	0.109881609673244	0.479118416083341
Mfap1a	402.137804315333	0.249735104674033	0.156087964421356	1.59996387677835	0.109606597266496	0.479118416083341
Ppap2a	981.300555724677	-0.385129040218405	0.241152901026916	-1.59703258214347	0.110258444590377	0.479118416083341
Prkcb	17.9773634981353	-0.992133435983757	0.620172378890841	-1.59977043440431	0.109649519967698	0.479118416083341
Prkce	53.3962011774554	0.566761402192736	0.354234212911717	1.5999623456303	0.109606936958982	0.479118416083341
Qdpr	7864.3985085578	0.296555272106414	0.185572979397038	1.59805200665516	0.110031403375078	0.479118416083341
Rarres2	286.763268542886	-0.472861027547602	0.295925030463323	-1.59790818237732	0.110063412810589	0.479118416083341
Sde2	567.012744751778	-0.189708363877826	0.118551578669375	-1.60021794738726	0.109550242016469	0.479118416083341
Sertad1	664.365129226122	-0.418618056046821	0.262121074183464	-1.59704082302754	0.110256607738518	0.479118416083341
Setd8	452.905129213546	0.261429381093888	0.163510530576598	1.59885348161977	0.109853162326934	0.479118416083341
Slc2a12	341.266531032206	0.352121274458956	0.219966584212018	1.60079439211348	0.109422466093734	0.479118416083341
Slc30a6	262.543514039491	-0.237820848320229	0.148520357548101	-1.60126767970651	0.109317644301043	0.479118416083341
Clec2g	40.4912771335937	-0.601242218662052	0.376640466098163	-1.59632931875501	0.110415287563819	0.479549162571789
Dbf4	96.4510486686303	0.370573666116415	0.232232905973897	1.59569835533155	0.110556156014907	0.47994062986858
Pgp	905.017937797569	0.18995906982988	0.119055734179555	1.59554742271708	0.110589874153247	0.47994062986858
Pdcd11	1002.66268300811	-0.161079295888819	0.100994999587064	-1.59492347687926	0.110729348985149	0.480362511666109
Nova1	35.562243645403	-0.740385757595071	0.464362663431438	-1.59441276377377	0.11084361548661	0.480491431586884
Rasa3	178.001086493903	0.417572575286286	0.261872930105339	1.59456181713137	0.110810256802365	0.480491431586884

Nucks1	828.278870145145	0.177068719257105	0.111103658809749	1.59372536560937	0.110997560314884	0.480975322355689
Fam219a	101.64657900208	-0.542200871133689	0.340478706431045	-1.59246631549188	0.111279965936212	0.482015279280102
Degs2	1944.24538381424	0.353009782048617	0.221737151853685	1.59201910504179	0.11138041186145	0.482071588163769
Hexim1	2071.61858783395	-0.314412252222387	0.197519623452039	-1.59180261043142	0.111429063437977	0.482071588163769
Ilkap	803.761231732574	-0.209274654559399	0.131472881093878	-1.59177050672501	0.111436279345098	0.482071588163769
Ppp1r7	527.856789576355	0.208834121069398	0.131205782558917	1.59165333262368	0.111462619540242	0.482071588163769
Adam17	510.517560134171	-0.307721606390828	0.193509156073995	-1.5902172932487	0.111785833773128	0.482760619703273
Agt	3567.23386860798	0.310898401992901	0.195556485481845	1.58981381377793	0.111876779192189	0.482760619703273
Gpx2	195.090965175785	-0.614229952487959	0.386145666150141	-1.59066903071012	0.111684080081184	0.482760619703273
Hnrnpf	587.110765427962	0.199149756426998	0.125265235601894	1.58982462668188	0.111874341172026	0.482760619703273
Rnf115	210.946027156024	0.272824231402453	0.171592688632516	1.58995254154876	0.111845502976743	0.482760619703273
Trim5	472.513226747095	-0.248150593612617	0.156022016053691	-1.59048447064818	0.111725643332669	0.482760619703273
Agtpbp1	697.583451666092	0.174297643623827	0.109733523617898	1.58837188378956	0.112202271393863	0.48361434078978
Ces2c	335.35743545279	-0.350105688515389	0.220368971810014	-1.58872497175884	0.112122498574878	0.48361434078978
Dnlz	434.638271166374	0.252506635758082	0.158967243124064	1.58841929189787	0.11219155792576	0.48361434078978
Csf1	214.506126307993	-0.522857018906783	0.32935670031736	-1.58750988943893	0.11239720894986	0.484087410732893
Map7	1799.23292103637	0.256252786693079	0.161415386823883	1.58753630453255	0.112391231288404	0.484087410732893
Dnajc4	396.410884508326	0.246541651858226	0.155472844244174	1.58575378907345	0.112795172124098	0.485345075199091
Klf4	350.571340084874	-0.286201546030774	0.180521616400994	-1.58541426637258	0.112872241893715	0.485345075199091
LOC501194	48.7229808374032	0.751004076346658	0.473718662466336	1.58533774548101	0.112889617441248	0.485345075199091
Sbp	10.7531613106056	-1.60026007718163	1.00944946808035	-1.58528002419459	0.112902725569804	0.485345075199091
Tbccd1	219.937203091277	0.256339496492573	0.161692955611936	1.58534733639101	0.112887439524202	0.485345075199091
Birc3	112.451533811317	-0.445999565181111	0.281502388958182	-1.58435445905706	0.11311307926296	0.486001576942697
Msrb1	9368.23080068404	0.229171544103601	0.144674992196856	1.58404393616121	0.113183721112733	0.486001576942697
Rpl22l1	1835.81590586482	-0.174871502039307	0.110390780340346	-1.584113288267	0.113167940965688	0.486001576942697
Mpp5	346.605200433452	0.328913204868415	0.207697574104465	1.58361601615522	0.113281126959613	0.486236136337975
Acsm2a	19751.6544890059	0.335150129282643	0.211772402795189	1.58259586640652	0.113513606264531	0.486436838248487
B3galnt2	496.36153470761	-0.237301839996213	0.149956428367711	-1.58247193921104	0.113541873295199	0.486436838248487
Fam217a	32.4069793354373	-0.656340138026843	0.414721175693068	-1.58260579998113	0.113511340717276	0.486436838248487
Mrs2	326.414316330335	0.215217235038647	0.135948035033134	1.58308455864179	0.113402192596999	0.486436838248487
Tgm3	74.9354629683476	0.864945317270637	0.546413556054765	1.58294996104369	0.113432870002251	0.486436838248487
Ednrb	424.599866535188	-0.400979502635704	0.253724204451921	-1.580375445464	0.114020911358774	0.487631545594237
Eml5	76.1111127787502	-0.472936051267846	0.299267367146007	-1.58031280115185	0.114035249708735	0.487631545594237
Mx1	44.2003930249699	-1.19469798065326	0.755691696214979	-1.58093305330351	0.113893345769571	0.487631545594237
Slc25a34	207.846692510094	0.371992994132983	0.23538498646599	1.58035990195463	0.11402446890414	0.487631545594237
Tulp2	7.8191938282615	1.25336764449384	0.793065149080073	1.58040943540099	0.114013132162514	0.487631545594237
Gak	1761.13263188349	0.162841663487721	0.103088266331939	1.57963334996225	0.114190857178157	0.488113306764546
Flnb	3043.76327569497	-0.140170606280263	0.0887883586611315	-1.57870478060346	0.114403787782301	0.488839643584072
LOC100192314	309.720193876698	0.257005958631415	0.162902314058268	1.57766917012295	0.11464163250079	0.489465174216813
Lrrc14	363.507214101453	-0.244733518271476	0.155120352325388	-1.57770089226016	0.114634341226063	0.489465174216813
Mapk8ip1	1757.0882725547	0.180059314287262	0.114141834578254	1.57750499589015	0.114679373476982	0.489465174216813
Gpr116	1321.91042383108	-0.400526030356109	0.253962901512433	-1.57710448246907	0.11477148599152	0.489674440607964
Ptrf	214.696177276769	-0.493148691524129	0.312782546957664	-1.57665028410578	0.114876015723276	0.48993650833424
Txnrd2	762.768158117285	0.255908739159419	0.162333888612528	1.57643447924939	0.114925707540008	0.489964588109428
Mybl1	653.928835884892	0.375469295824558	0.23824370397408	1.57598832439831	0.115028493992531	0.490218921154522
Cxcl12	1267.53065851754	-0.702765168831592	0.446113207904582	-1.57530679742148	0.11518564574155	0.490704666228805
Haao	15638.2879274242	0.366781044341507	0.232964133907629	1.5744099239196	0.115392711019806	0.491402605264561
Atg13	526.110175375215	0.209151732090609	0.133007923447309	1.57247573430062	0.115840262434968	0.491499997352741
Cep85	485.393747642982	-0.219746698911089	0.139717352952404	-1.57279460473279	0.115766385341233	0.491499997352741
Ces4a	159.93441323693	-0.372563788838448	0.236681893804082	-1.57411191388744	0.115461578706763	0.491499997352741
Chka	874.041864356914	-0.735053155988494	0.46709645804451	-1.57366458967764	0.115565012339599	0.491499997352741
Dcun1d3	51.0727210118047	0.477768850989104	0.303693073091106	1.57319640558867	0.11567334736	0.491499997352741
Ganc	359.619229967711	-0.215422187272999	0.137006200261741	-1.57235356400987	0.115868577121567	0.491499997352741
Glyr1	906.574439264194	0.345903396502243	0.220062617486321	1.57184078083478	0.115987481187446	0.491499997352741
H2afy2	182.692264009177	0.324510944742649	0.206245839512183	1.57341813784069	0.115622029884521	0.491499997352741
Il6r	79.4874158539567	-0.762505073828332	0.484960705266748	-1.57230279803582	0.115880344451255	0.491499997352741
Kifc1	1682.27880862225	-0.27235307785808	0.173305964321864	-1.57151589631541	0.116062864961076	0.491499997352741
Mycbpap	399.244193587972	-0.363627058985328	0.231364890044284	-1.57166050093309	0.11602930723377	0.491499997352741
Sapcd2	15.1318711807122	0.937927043976111	0.59653097977243	1.57230232088519	0.115880455057129	0.491499997352741
Satb2	265.608630117117	0.556956210556661	0.354373534865832	1.57166423493653	0.116028440801802	0.491499997352741
Trim44	187.810088950652	0.295486139781968	0.18793066740032	1.57231464065702	0.115877599299705	0.491499997352741
Zdhhc2	188.967634988771	0.346430988743088	0.220444656208671	1.57151003204704	0.116064226015727	0.491499997352741
Crls1	1177.39162323411	0.221119306290287	0.140761897093583	1.57087472431037	0.116211750688321	0.491768242515089
Syap1	933.053407873647	0.23155879253918	0.147408499052126	1.57086459755144	0.116214103413297	0.491768242515089
Hormad2	240.494219363273	0.481833501021537	0.306806242213258	1.57048141376022	0.11630315506501	0.491961913088537
Anxa6	2599.82656876595	0.350993293121825	0.223589828567307	1.5698088565606	0.116459586526028	0.492084967366807
Cenpw	32.6832501495919	0.574949539914892	0.366287767577212	1.56966623187517	0.116492781128027	0.492084967366807
LOC100158225	263.546710240648	-0.282297821293747	0.179862594097578	-1.56951934731129	0.116526974946614	0.492084967366807
Lrrk1	406.649996249217	-0.414650551341558	0.264205232404146	-1.56942596317427	0.116548718295922	0.492084967366807
Spata1	25.0280676804948	-0.643331145706267	0.409734841887419	-1.5701157918199	0.116388175331226	0.492084967366807
Gatad2b	89.6248795931119	0.428010240847929	0.272789425274163	1.56901331647209	0.116644836170929	0.492307912335231
Ces2g	425.334279818556	0.329961632183266	0.210383726994703	1.56838001159459	0.116792473089263	0.492565213036201
Fbxo23	30.7348869859901	0.751095263734492	0.478881472123068	1.56843667474666	0.116779257721111	0.492565213036201
Rufy3	614.93810142502	0.263917152029854	0.168374895758045	1.56743765655602	0.117012427818194	0.493309812530263
Fads3	253.169828711258	0.315267772476613	0.201289143018682	1.56624330427674	0.117291668335601	0.494135477747003
Tox4	1254.19187816455	0.168075582973774	0.107312326958107	1.56622810946395	0.117295224261958	0.494135477747003
Psme1	3368.23379023493	-0.198980176142664	0.127071170667927	-1.56589551427566	0.117373080183156	0.494221268159521
Wnt5a	235.443274019242	-0.5187661256	0.331317014117443	-1.56576965110554	0.117402553583557	0.494221268159521
Aatk	19.336773504901	0.928506631480207	0.593387869359606	1.56475499319234	0.117640368437026	0.494323863429511
Alb	10.6885671896469	1.42580510492907	0.91114014768229	1.56485817089276	0.117616168462587	0.494323863429511
Bcl3	134.039620918011	-0.357776317666052	0.228649361665658	-1.56473788100581	0.117644382419217	0.494323863429511
Mob3b	174.034908712372	0.560587903218446	0.358195358831253	1.56503396651362	0.117574945204021	0.494323863429511
Proc	1752.60952278284	-0.244836987962132	0.156444858751002	-1.56500501145783	0.117581734250315	0.494323863429511
Fbxl22	162.241042085354	-0.334087193277063	0.21365659597935	-1.56366430788476	0.117896423959048	0.495199835446614
Pex13	1410.76957043893	0.237072372377013	0.151642380757923	1.56336487987133	0.117966795838442	0.495312375877257
Slc22a12	21652.961653046	0.271606506638497	0.173759425834746	1.56311811767155	0.118024814910102	0.495372986066553
Erich1	137.190799636249	0.308298711758207	0.197276719122549	1.56277290665347	0.118106018965902	0.49553082745162
Tsen34	712.677508052569	0.174231132196765	0.111515554274118	1.5623930969171	0.118195412313778	0.495722899025239
Mrm1	330.897316341165	-0.325114538170493	0.208192091880431	-1.56160848970773	0.118380248262398	0.496314976669278
Ajuba	697.317715540872	-0.290310116962251	0.186040863640115	-1.56046425114344	0.118650211592749	0.496531040118992
Cyp4a3	26113.9445086133	-0.549525753945216	0.352135640952548	-1.56055136156827	0.118629642431945	0.496531040118992
Dram2	1015.96066734703	0.26168221841256	0.167666739650128	1.56072825748634	0.118587881057008	0.496531040118992
Lats2	755.776643692574	-0.29972963272092	0.1920064064627	-1.56103974988536	0.118514372328572	0.496531040118992
Metap2	1727.41660823244	0.152113756605551	0.0974678405535897	1.56065586086229	0.118604970971011	0.496531040118992
Fam178a	350.768296221136	-0.240329029053642	0.154043074084345	-1.56014173621368	0.118726390557126	0.496666969110158
Btrc	219.331950691655	0.256820185376722	0.164681358429949	1.55949761299769	0.118878649097659	0.496755413839701
Gab2	42.6938001082416	-0.770464946219597	0.493993867363892	-1.55966500218119	0.118839066749599	0.496755413839701
Pkp1	42.7959973569777	-0.966320020206818	0.619595678808223	-1.55959773971554	0.11885497099381	0.496755413839701
C1qtnf9	13.4194001964778	1.32584563971451	0.850649223186296	1.55862793214371	0.119084467993945	0.497262989437663
March7	403.55513941453	0.306233741397929	0.196483275852785	1.55857408254621	0.119097221229731	0.497262989437663
RGD1307621	26.0988120486067	-0.651440348904503	0.418060212575083	-1.55824527020137	0.119175117299683	0.497262989437663
Slc31a1	1448.63567808387	0.208875413066619	0.134029869837091	1.55842435212763	0.119132687609932	0.497262989437663
Tcf21	151.788125528425	-1.25334601939667	0.804928579910923	-1.55708972283649	0.119449184718868	0.49822364532648
Cdo1	6498.19734018234	0.268075290903886	0.172247972324943	1.55633350735861	0.119628807593881	0.498484213437754
Cldn7	552.46147933193	-0.398372726683693	0.256009067721676	-1.55608834573309	0.119687085911634	0.498484213437754

Naa38	393.818168847299	-0.229670477523306	0.147543143205006	-1.5566326739033	0.11955772167497	0.498484213437754
Nrtn	103.493652673724	0.505252841889713	0.324656223771106	1.55627030962429	0.119643828444595	0.498484213437754
Rgma	43.7390175681648	-0.694541519613471	0.446393125304662	-1.55589654105773	0.119732696046423	0.498491510352985
B3gntl1	36.5568789249329	-0.547853264436332	0.352241382237606	-1.55533475639945	0.119866363797164	0.498500216216086
Elf4	19.2654996913688	-0.867552793270723	0.55775361742667	-1.55544090825154	0.119841097695821	0.498500216216086
Rab1b	3557.87716968754	0.165795071418236	0.106581931975781	1.55556451590605	0.119811682047829	0.498500216216086
Pcdhgb7	84.3310946148392	-0.524245408555947	0.337160293638847	-1.55488477868482	0.119973513132979	0.498763332212672
Pias4	470.263752722225	0.208760178872932	0.134310084756823	1.55431499615912	0.120109298168721	0.498962822728686
RT1-T24-2	47.6263845357029	-0.717505773625157	0.461607556764292	-1.55436314486405	0.120097819184371	0.498962822728686
Eno2	39.6496585536903	-0.569768344225792	0.366820503586707	-1.55326198687013	0.120360557778888	0.499276717805937
Gprc5c	2886.25005868587	0.220583668169759	0.141973285387568	1.55369841282179	0.120256371857089	0.499276717805937
Lypla2	1177.62070849444	0.15081085056406	0.0970943374181576	1.55324043166968	0.120365705381509	0.499276717805937
Pcbp3	197.809331459557	0.31350964656979	0.201856307839566	1.55313277016325	0.120391418632066	0.499276717805937
Pnldc1	29.6007546883097	-0.634312587380494	0.408422871006141	-1.55307802870559	0.120404494413696	0.499276717805937
Eif2s3x	2923.97322828239	0.162009607539714	0.104390431125758	1.55195841029283	0.120672175290667	0.500204210194648
Odc1	36645.3964446437	0.389426049699268	0.251072707703759	1.55104891033697	0.120889963231588	0.500559315879866
Zfp426	169.520477573364	-0.340386035105607	0.21941404470738	-1.55134114390699	0.120819951765763	0.500559315879866
Zmym1	209.945190002998	-0.310441106019137	0.200138049287465	-1.55113486478146	0.120869367516131	0.500559315879866
Ctbp1	3864.57843435894	0.177083510767907	0.114205325133422	1.55057139902212	0.121004430675331	0.500668496197964
Spice1	225.907798592737	-0.325687947253262	0.210037039282077	-1.55062149212582	0.120992418541075	0.500668496197964
Klk1c12	7.1494452889205	1.74155881356441	1.12362115901181	1.54995195631245	0.121153047783347	0.501100997491091
Pigt	1649.67576249338	0.196378388187557	0.12675039785627	1.54933153275181	0.121302043313831	0.501534748746818
Alms1	369.156507085328	-0.250812702284589	0.161995220767675	-1.54827223356355	0.121556766624898	0.50167545731975
Dnajb1	1083.06490014327	-0.269836243305826	0.174244866324178	-1.54860369202386	0.121477017873511	0.50167545731975
Gfra2	45.2646725531318	-1.02607459635166	0.662643719423375	-1.54845592929567	0.121512564461036	0.50167545731975
MGC94335	782.989935575899	0.186609137360846	0.120523009326105	1.54832789526462	0.121543371585822	0.50167545731975
Rasgrp2	71.0675084303642	-0.619185722359668	0.399796496760344	-1.54875224614796	0.121441289102083	0.50167545731975
Fam107b	6186.68419077828	0.489090547621839	0.316060005473936	1.54746104901328	0.121752109959992	0.502148162151526
RGD1311847	425.555621436329	0.189238521185613	0.122301610389954	1.5473101342021	0.121788479189623	0.502148162151526
Tbx19	43.4273279710854	-0.555464780379993	0.359002149613314	-1.54724639108232	0.121803843309146	0.502148162151526
Smarcad1	749.089301072499	-0.266066391170386	0.172040490378538	-1.54653355489143	0.121975762755214	0.502674590092736
Phc2	923.347933155031	-0.178307336663862	0.115321248403363	-1.54617938265973	0.122061251336551	0.502844575096498
Mgrn1	900.603306414563	0.297512582449251	0.192476761253787	1.54570650769092	0.122175464879785	0.502944300040268
Pi4k2a	161.753389333932	0.366723570198975	0.237280194337736	1.54552962678796	0.122218208403239	0.502944300040268
Sall1	992.89950456694	-0.29257722461625	0.189299349064308	-1.54557966555319	0.122206115278653	0.502944300040268
Mcart1	808.532361834436	0.331228041181284	0.214368913370211	1.54513094260668	0.122314593866094	0.503158767239242
Acer2	2725.06659306435	-0.32025645861989	0.207390010680533	-1.54422316469821	0.122534278898444	0.503515767881313
Ccdc28a	141.402523801868	-0.312485908987703	0.202347590442768	-1.54430259487615	0.122515044253107	0.503515767881313
RGD1305350	1038.78486859698	0.14351653696496	0.0929357847189416	1.54425485725424	0.122526604012647	0.503515767881313
Tmem30a	1214.64320178883	0.251665713487852	0.163045944235811	1.54352636410184	0.122703115824195	0.504027327234476
Ddx19b	757.393787433843	0.153126272886123	0.0992305232006144	1.54313680858608	0.122797585495968	0.504060265118715
Smim1	427.184583030252	0.222076535108492	0.143913271833779	1.54312755369076	0.122799830557252	0.504060265118715
Myo7b	805.718133654392	0.198300963203645	0.128539655340058	1.54272207031387	0.122898224579005	0.504282029084826
Smad4	452.084204540031	0.302888640077596	0.196470668883457	1.5416481340391	0.123159121974085	0.505170184178076
Tube1	153.513532675898	-0.296338321441274	0.192283393640247	-1.5411540010351	0.123279309682289	0.505480748141737
Dusp3	2335.14703933583	0.230027920806288	0.149284293179609	1.54087155391179	0.123348050269969	0.505579803164363
Pfkfb3	166.889573835874	-0.690263536117513	0.448022552510226	-1.5406892627392	0.123392431284352	0.505579803164363
Rnf19b	177.965064969157	-0.549572348473091	0.356812358301679	-1.54022789762354	0.12350481196905	0.505857907329809
RT1-S2	31.157947939915	1.389371124896	0.902517042740167	1.5394403197946	0.123696837116131	0.506461905858048
Foxj2	559.351041284345	-0.208936279122631	0.135780286366583	-1.53878213630025	0.123857492362979	0.506497914844935
Man1a2	457.630449369078	0.253213432617379	0.164569654758538	1.53863987251418	0.123892238758955	0.506497914844935
Olr1	38.546803941649	0.666775828254102	0.433383857775179	1.53853406464437	0.123918086122844	0.506497914844935
Padi1	24.9496281297626	-1.12481143678678	0.730928488925062	-1.53888027875474	0.123833526559825	0.506497914844935
Wdr44	109.189675491008	-0.388284609885685	0.252380054022279	-1.53849166642705	0.123928444587697	0.506497914844935
Atp6v1b2	4034.70996690928	0.187776172479988	0.122180149281565	1.53687954699791	0.124322808962255	0.507566748530521
Mylip	454.526715791998	-0.358723113309559	0.23343059227412	-1.53674421940509	0.124355957934688	0.507566748530521
Nfil3	243.215538227141	-0.37202326281552	0.242165090534232	-1.53623820012543	0.124479970242061	0.507566748530521
Pamr1	413.111955936374	0.477311827078498	0.310685571485067	1.53631797188702	0.124460413833311	0.507566748530521
Pdhx	580.1420066167	0.2387744781539	0.15543735268364	1.53614606805531	0.12450255981903	0.507566748530521
Prep	6891.4382678159	0.309284793575489	0.201324559952611	1.53624969376955	0.124477152375351	0.507566748530521
Slc25a25	8525.62508179868	0.788603544576835	0.513089046246175	1.53697209158208	0.124300143808226	0.507566748530521
Cxcl1	27.2912496552343	-1.0325976225284	0.672319612995884	-1.53587312130774	0.124569501554849	0.507597587614106
Them4	204.496140664109	0.286799529913816	0.186748708474255	1.53575107564053	0.124599443026866	0.507597587614106
Mmgt1	935.649748092822	0.181104900736335	0.117970892101945	1.53516598467216	0.124743061394169	0.508000586100367
Rps14	7189.17586769433	-0.247625640799816	0.161339036442804	-1.53481541888098	0.124829174259426	0.508169195785327
Ckap2	75.0419516117329	0.58979976247167	0.384494809014795	1.53396027369767	0.1250394262816	0.508478754603456
Lemd2	822.202154257488	0.168193895156978	0.109645716526048	1.53397597722864	0.125035562813963	0.508478754603456
Sptbn1	5760.13955275561	0.276699351007796	0.180382095886444	1.53396239049127	0.12503890549116	0.508478754603456
Mtss1	1997.90646765754	-0.131230005036266	0.085574030245332	-1.53352605527685	0.125146291915987	0.508613312009732
Smarca4	1628.88985840384	0.133882618735942	0.0873074135237251	1.53346220363704	0.125162012466234	0.508613312009732
Mapkapk2	3110.98439390824	0.146709118602166	0.0957056337529595	1.53292040237526	0.125295468239505	0.508791815652095
Slc28a2	153.578246778262	-0.64151406424525	0.41846388209514	-1.53302134710732	0.125270595262426	0.508791815652095
Frk	291.660128200513	-0.296232912298877	0.193304914984507	-1.53246446073355	0.125407861102257	0.508910461492496
Rgl2	540.794699745198	-0.249041535808272	0.162513221781645	-1.53243861070509	0.125414235671343	0.508910461492496
Cwc25	308.144485674456	-0.29223488058181	0.190906960242969	-1.53077122075528	0.12582594466463	0.509489735325322
Dusp14	25.6595540576604	-0.87340886134547	0.570415715520296	-1.53117951974518	0.125725030975448	0.509489735325322
Fat4	93.1669715789016	-0.688535966340537	0.449790921317518	-1.53079116030998	0.125821015011863	0.509489735325322
Fes	126.106865560696	-0.381847629841629	0.249394890197597	-1.53109644523627	0.125745558256611	0.509489735325322
Mfng	66.1566026401908	-0.632210079088813	0.413000860455257	-1.53077182064928	0.125825796350741	0.509489735325322
Snx10	71.921601581065	-0.534479103924471	0.349062543535639	-1.53118435026215	0.125723837460031	0.509489735325322
Gfra3	611.157751012481	0.320839877447979	0.209640440583459	1.53042932248681	0.125910495528313	0.509650531401284
Fam116a	1007.94564975545	-0.214933933189795	0.140545245002827	-1.5292864101199	0.126193457118937	0.509706763899729
Grifin	48.0285438213674	3.05862545463226	1.99972482722402	1.52952316888428	0.1261347998857	0.509706763899729
Nabp2	565.117428127718	0.187348349922698	0.122489286651183	1.52950804959961	0.126138545069547	0.509706763899729
Sirt3	561.162471763246	0.183566598345562	0.120022819146656	1.52943081699541	0.126157677636825	0.509706763899729
Tdp2	607.883357104646	-0.161378884513528	0.10551314156405	-1.52946715566768	0.126148675301065	0.509706763899729
Ube2d1	70.3440884601213	-0.400530563280901	0.261888241029195	-1.52939498813255	0.126166554165164	0.509706763899729
Gmfg	160.375790559803	-0.378750545817211	0.247742089483652	-1.52880984658929	0.126311590651014	0.509910849629764
Snrpg	733.628556895918	-0.234903594490773	0.153660248023871	-1.52872065164362	0.126333710413287	0.509910849629764
Leprot	927.26807513291	-0.193904253411563	0.126902977433219	-1.52797245055659	0.126519378177598	0.510428245978897
Nfib	74.6631566445351	0.470343676475301	0.307848329153632	1.52784222597023	0.126551715394029	0.510428245978897
Vldlr	266.874963458152	0.375286350833361	0.245670051388255	1.52760317634428	0.126611092664138	0.510486583618514
Cog1	817.236447093583	0.241277159657062	0.157969875388408	1.5273618407549	0.126671059741174	0.510547257098648
Col4a3bp	1718.54480396608	-0.206750079082162	0.135389305726539	-1.52707836097304	0.126741527045796	0.510650193691073
RGD1565033	262.149424279289	-0.296359062082177	0.194128835414615	-1.52661020939636	0.126857966804802	0.510938217825436
Dok3	42.2770514411987	-0.715124344088632	0.46852864230014	-1.5263193741537	0.126930345949569	0.511048640475663
Nup88	565.456531092767	-0.220043603167605	0.144208317183407	-1.52587317753489	0.127041451809624	0.511314851723864
Acaa1b	148.284599133974	0.725662272229367	0.475661022129233	1.5255870009719	0.127112751463483	0.511420719693433
Afg3l1	1303.45104167409	0.224919238969317	0.147521812598376	1.5246507279683	0.127346237441708	0.512091822757764
Pax2	293.8251732462	-0.334572685501416	0.219481677887501	-1.52437637948488	0.127414717117628	0.512091822757764
Plscr4	66.9001119984115	-0.566876276670871	0.371849674237259	-1.52447700225542	0.127389597506538	0.512091822757764
Ei24	1876.71218568803	-0.134010709683547	0.0879485318055133	-1.52374015725349	0.127573633757895	0.512549282888738
Oas1b	465.910680841234	-0.233455284493959	0.153334713973765	-1.52252075504509	0.127878648942342	0.513593188649703
Adamts1	613.221951273714	-0.468103274651096	0.307595329777165	-1.52181528565538	0.128055370313118	0.51412127833942
Trpc3	31.6096146189052	0.818260835875633	0.537784628077888	1.52154002393152	0.128124375355308	0.514216684423881

Chpt1	4375.48188497044	0.271698117704918	0.178760459260222	1.51990053521516	0.128535975939757	0.514332021645468
Eif3c	3390.18625663809	0.152909526984554	0.100611382394944	1.5198034590591	0.128560379508602	0.514332021645468
Gsta2	16039.2899331107	0.201469882468067	0.132505337975174	1.52046616043358	0.128393857376756	0.514332021645468
Hlx	69.5402426729371	0.692919029166791	0.455595985825834	1.52090679181638	0.128283229330702	0.514332021645468
Iqch	729.76406180056	-0.334866154202836	0.220281239200595	-1.52017555111852	0.128466860389072	0.514332021645468
Mapk7	141.050820401452	-0.295368916652852	0.194274336670528	-1.52037022344218	0.128417953827197	0.514332021645468
Rnf26	515.44323692554	0.252307291068891	0.165860788858864	1.52119915023187	0.128209868651854	0.514332021645468
Tex36	47.8119889720317	0.932933240124558	0.613467888185753	1.52075317729112	0.128321788468498	0.514332021645468
Tspan8	1050.05589527714	-0.425971412683268	0.280249923532801	-1.51996977309937	0.128518572717371	0.514332021645468
Tmem120a	990.082922055719	0.264016230223575	0.173771177928648	1.51933268434184	0.128678776672603	0.514624551604788
Aacs	198.000335248685	-0.61972225848998	0.408259664198212	-1.5179610253858	0.129024224220954	0.51467201349468
Acbd4	1588.89632894773	0.326952368002415	0.215372791243708	1.5180764762084	0.128995120592804	0.51467201349468
Hpn	8517.57619871728	0.221443612350862	0.145846318328716	1.51833529216528	0.128929895039398	0.51467201349468
Ldb1	1129.16828010467	-0.163202139268078	0.107506813935346	-1.51806321193954	0.128998464080522	0.51467201349468
Map3k4	823.005953110399	0.23084757547681	0.152088800724784	1.51784729958221	0.129052897981686	0.51467201349468
Nle1	191.002459439705	0.332387412955027	0.218838939442026	1.51886777464064	0.1287957819572	0.51467201349468
Slc43a2	779.683304991519	0.305666872721974	0.201370361161379	1.51793377614798	0.129031094138076	0.51467201349468
Tamm41	195.369004750041	0.27420321414556	0.180568911652445	1.5185516246193	0.128875395636526	0.51467201349468
Kpna6	484.139140808528	0.210373282153686	0.138813663359418	1.51550846697983	0.129643687753988	0.514680459312548
Lilrb3l	19.3058268337167	-1.14926930519255	0.757482644038225	-1.51722196440788	0.129210652599478	0.514680459312548
Ovol1	480.753836090302	-0.245067891333554	0.16169627766554	-1.51560626423611	0.129618942143832	0.514680459312548
Plrg1	764.263723247469	-0.16355507101481	0.107817193846453	-1.51696649838369	0.129275142628336	0.514680459312548
Ppp2cb	1718.33284157528	-0.14846082275938	0.09793915861823	-1.51584743889913	0.129557933465607	0.514680459312548
Psme3	1988.74370391487	0.16367563601627	0.107891007574338	1.51704613476239	0.129255036481666	0.514680459312548
Rtn3	2162.96744656556	-0.265544328535135	0.175137246461845	-1.51620705418014	0.129467004905505	0.514680459312548
Sez6	19.1311698979849	1.03824937402075	0.684845159282463	1.51603520875954	0.129510449757414	0.514680459312548
Slc26a2	24.0238802666071	-0.8045657431189	0.530632474647213	-1.5162391703482	0.129458886758185	0.514680459312548
Slc39a5	911.981326658554	0.320592272580229	0.211432562962346	1.51628617696567	0.129447005398145	0.514680459312548
Synm	504.060399141575	-0.250500256233355	0.165138025919493	-1.51691444074472	0.129288287162245	0.514680459312548
Tatdn2	1416.38871730105	0.192201608320854	0.126712861032835	1.51682794275357	0.129310130167212	0.514680459312548
Ypel3	844.825914602134	-0.190773357448641	0.125834669832327	-1.5160635594534	0.129503281538006	0.514680459312548
Bdh1	1308.03329974577	0.212987170636278	0.140567596270428	1.51519394431791	0.129723296196187	0.514816684555119
Mterf	486.695411239041	-0.217835956392567	0.143792078419684	-1.51493711466339	0.129788330176923	0.514894995040456
Abcd2	67.6752182537039	0.529681571814559	0.349745075377205	1.51447899943492	0.129904396195241	0.515175634038768
Nt5c3b	370.709060083689	0.282626326915383	0.18665733455738	1.51414530581171	0.129988990020949	0.515331308189086
Zfp275	124.831458075255	-0.431362783817244	0.284941051674679	-1.51386674991899	0.130059638759676	0.51543160883629
Cpt1b	581.787437514235	-0.777179810026967	0.513503953867732	-1.51348359476732	0.130156865170851	0.51545746673585
Tbxas1	26.8128151468525	-0.969427994109322	0.640471994146696	-1.51361496360336	0.13012352370486	0.51545746673585
Lrrc57	400.833321413263	-0.178881888262559	0.118213280998852	-1.51321312420299	0.13022553159138	0.515549770835118
Myo3b	113.466285163384	-0.404471382555549	0.267397816248747	-1.51262036552792	0.130376118063972	0.515966210973225
Pigl	189.66810961588	-0.273561337589932	0.180883428264347	-1.51236263164	0.130441635807188	0.516045817119561
Ppp4r2	1912.08766653351	-0.194186337626293	0.128415232215006	-1.51217526361021	0.130489282094951	0.516054690428397
Zfand3	1752.46318926742	0.131574274640272	0.0870294433580771	1.51183633450254	0.130575503533077	0.516216060228504
RGD1565616	383.48457830778	0.342158944723003	0.226424843786705	1.51113693621589	0.130753565768073	0.516609445770021
Shc1	491.120403537934	-0.243406360690017	0.161080152926348	-1.51108846290524	0.130765913732215	0.516609445770021
Poldip2	2815.12400405159	0.182551271751692	0.120824047903982	1.51088524940635	0.130817689651104	0.516634419935526
Arg1	26.3044964080283	-0.616754121250019	0.408410934260346	-1.51013126611557	0.131009932860557	0.516989520124696
Cecr2	92.8755136607376	-0.365800579384451	0.242263054792334	-1.50993134177233	0.13106094432453	0.516989520124696
Cst7	16.8402394367531	-0.852948531685729	0.564797525480342	-1.51018461166296	0.130996324141954	0.516989520124696
Eftud1	500.704602668076	0.267672936301874	0.177361598565565	1.50919330039149	0.131249391808867	0.516989520124696
Hps1	1038.81868114832	0.190521106136903	0.126217341335589	1.50946854149258	0.131179088688678	0.516989520124696
Nkiras2	345.807734440907	0.240730398374746	0.159518462875664	1.50910680829706	0.131271489977114	0.516989520124696
Slc17a4	65.5275150576265	-0.427191779703009	0.282946738745535	-1.50979573610565	0.131095553397058	0.516989520124696
Ube2l6	92.1660198731877	-0.479272617599271	0.31757380123065	-1.50916925685309	0.131255534487265	0.516989520124696
Ces1c	66.9273206175696	0.458345319489427	0.304029124270266	1.50757043618618	0.131664503970237	0.517998875779062
Rbm28	557.087797569717	0.21380875658708	0.141801176972858	1.50780664273333	0.131604021540433	0.517998875779062
RGD1307461	42.5079272756932	0.649222836689193	0.430609857314432	1.50768224568331	0.131635871638468	0.517998875779062
Sh3glb1	1005.57443092609	0.138352947803745	0.0917858462880545	1.5073451234469	0.131722217034248	0.518046615505627
Arid4b	215.540207100038	-0.277968264437505	0.184500740721688	-1.50659701066897	0.131913984115622	0.51844202747516
Atp6v1c1	2145.47346312949	0.18406196921254	0.122158938342221	1.50674172279479	0.131876872545355	0.51844202747516
Nacc1	385.176103244554	0.302741691941022	0.200994793151894	1.50621658996031	0.132011581988545	0.518646263699204
Nob1	1028.51198212632	0.218263429157899	0.144933742018028	1.50595317638835	0.132079194167397	0.518732591882046
Akr7a2	10857.1005687768	0.218403955279868	0.145178566098473	1.5043815430146	0.132483153863244	0.519959781356917
Hdac11	1253.16560533905	0.187069986644651	0.124335485308813	1.5045583019202	0.132437673508722	0.519959781356917
Gga2	1234.69901928524	0.238284736004082	0.158500780827208	1.50336632261674	0.132744606019341	0.520037179498945
Pom121	496.422885427898	-0.21448991010234	0.142617280453409	-1.50395456581722	0.132593065693237	0.520037179498945
Prkcg	32.8883445181954	-0.623209443238142	0.41457772188527	-1.50323910412776	0.132777397053136	0.520037179498945
RGD1309821	61.2449434872904	0.444385845752822	0.295566626076467	1.50350481599317	0.132708915970937	0.520037179498945
Synpo	77.9389011352259	0.694603940029554	0.461995332353386	1.50348692159133	0.13271352697969	0.520037179498945
Tmem70	1499.23147911428	0.208893200187908	0.138923185153649	1.50365973798306	0.132669001037881	0.520037179498945
Tek	616.018948435946	-0.905719095971311	0.602983860502365	-1.50206192122079	0.133081117876281	0.521047187661664
Susd3	1837.95133849456	0.235695274816687	0.156935285218934	1.50186285058761	0.133132532510659	0.521068996045508
Rchy1	1003.97669889189	0.208824979301089	0.139113052554686	1.50111708043355	0.133325281750063	0.521643770179419
Chd8	954.603675465375	-0.171680612539854	0.114395889571606	-1.5007585778018	0.133418015962589	0.521826968145487
Srfbp1	301.748469081882	0.22348461678857	0.148981941391817	1.50007856456114	0.133594052750712	0.522335742540279
Cep95	373.070228456028	-0.24588018845951	0.163989335410007	-1.49936694264148	0.133778464543915	0.522456344491263
Faf2	483.932423731877	0.169955261945533	0.113337884953752	1.49954502869789	0.133732296336087	0.522456344491263
Fchsd1	123.857600070237	-0.308809154271224	0.205975744889076	-1.49925009101206	0.133808764632594	0.522456344491263
Uimc1	722.766627620476	-0.192248007298953	0.128203734194993	-1.49955076196573	0.133730810210623	0.522456344491263
Kdm5b	876.172649947456	-0.139660766080564	0.0931814057177967	-1.49880509963042	0.133924201260906	0.522727497091847
Hnrnpa1	3240.0887557433	-0.221679360253822	0.147950959738383	-1.49832999154456	0.134047535571669	0.522763811725582
Ppm1j	27.6338177253845	0.590809466871847	0.394336252415339	1.49823776853661	0.134071486114734	0.522763811725582
Zkscan1	172.274884786746	0.408570647336956	0.272661923983833	1.49845142059946	0.13401600519323	0.522763811725582
Dchs1	129.562776254809	-0.736571985037634	0.491721105384965	-1.49794665506777	0.134147110715158	0.522769309614863
Gpc2	32.5686175096955	-0.634663818436822	0.423758613228884	-1.49770128234307	0.134210878537407	0.522769309614863
Irak1	1983.24531089266	0.192810233640269	0.128730549739156	1.49778148256925	0.134190033407186	0.522769309614863
Dapl1	95.9506374584023	0.541945309773455	0.362207090615027	1.49623053721348	0.134593589383841	0.523005380149312
Gna11	295.402860447002	0.302511226095698	0.202155995773633	1.49642470379379	0.134543015924113	0.523005380149312
Kpnb1	1059.74818031112	0.209292109621388	0.139817897532167	1.49689069364841	0.134421702147081	0.523005380149312
Nxn	206.519939556769	-0.448160419185492	0.299519584538289	-1.49626415874052	0.134584831124922	0.523005380149312
Pmpca	2460.13292623627	0.208802967967309	0.139522040365658	1.49655901977982	0.134508040003902	0.523005380149312
Sumo2	2601.45908102074	-0.18284982170635	0.122155186162036	-1.49686499158378	0.134428391106598	0.523005380149312
Tsta3	682.961389347488	0.17040005921342	0.113805646534458	1.49729002384628	0.134317809562719	0.523005380149312
Fam3c	677.926558663042	0.295070027303863	0.197403043700503	1.49475925888731	0.13497728254564	0.524264304384901
RGD1311756	1620.12229498893	0.236773586015437	0.15841569544213	1.49463464055512	0.135009820423597	0.524264304384901
Slc35d1	974.314692407262	0.209003475983362	0.139873898096285	1.49422786401142	0.135116072066887	0.524497703248715
Dapk1	682.419317731122	-0.235755772068072	0.157813726840122	-1.49388634809259	0.135205327262011	0.524664987934454
Hbegf	128.571542705797	-0.777128874738005	0.520681884733971	-1.49252143683673	0.135562501777788	0.52587146594073
Apoe	343.336944757072	-0.89694134906542	0.601142076382007	-1.49206216684031	0.135682848810911	0.526158737490553
Bcas3	517.667294752061	0.190616616111406	0.127789618024659	1.49164399313427	0.135792498732639	0.526404345359883
Ipo4	872.956034663057	0.191768098027369	0.128580210046619	1.4914277862654	0.13584921747279	0.526444666142424
Hpcal1	1367.4348427435	0.312701804322465	0.209697491691854	1.49120431436526	0.135907861313312	0.526481311151928
Uros	166.745516605306	0.390717988209055	0.262044149971157	1.49103877438997	0.135951315170764	0.526481311151928
Ehf	578.314653180151	-0.777945027570316	0.521991115176452	-1.4903415114783	0.136134462822081	0.52701100287322
Kif12	4283.7177012241	0.185817289539403	0.124742353525733	1.48960865566057	0.13632716478866	0.527218290230661

Lrp1	500.298661514401	-0.967170359007272	0.649263222311673	-1.48964291487774	0.136318151754227	0.527218290230661
Rasal2	68.15987431491	0.448800244964045	0.301266390099788	1.48971229354655	0.136299900780748	0.527218290230661
Slc4a7	23.0661442359258	-1.38557365087533	0.930775846098158	-1.48862226784644	0.136586864463183	0.527863414310963
Vdac1	17253.4044361437	0.264341103517221	0.177561182731326	1.48873250026277	0.136557823147828	0.527863414310963
Adipoq	561.174200054703	0.931826173393921	0.626187225875238	1.48809514932452	0.136725802528594	0.528175414985901
Atxn2l	1849.462189124	-0.19830080675826	0.133288023476613	-1.48776162768333	0.136813768580187	0.528175414985901
Lrpprc	2323.30817150096	0.161900740054199	0.108832702428706	1.48761113563505	0.136853475024941	0.528175414985901
Wnt5b	55.7852565244725	-0.64259779621954	0.431965768041345	-1.48761277805244	0.136853041634761	0.528175414985901
Abcd3	4358.84869533982	0.225042385464944	0.151356133307897	1.48684021285844	0.137057017922769	0.528183048420335
Atf2	189.363710398208	0.292197287083943	0.196498116710715	1.48702334645841	0.137008644936094	0.528183048420335
Nt5e	1948.61732821743	0.302265453217716	0.20329943853528	1.48679925235142	0.137067839051078	0.528183048420335
Pex14	476.976243647015	0.165441368575043	0.111279164043943	1.48672368269869	0.137087805106457	0.528183048420335
Piwil4	9.75453181260974	1.0187089209428	0.685200169639782	1.48673185746341	0.137085645165575	0.528183048420335
Synj1	330.088554759896	-0.267015968868663	0.179628991712571	-1.48648593037766	0.137150635641911	0.528246060557765
Bloc1s4	464.555681505891	0.362618992671767	0.243982202391085	1.48625182131325	0.137212525079386	0.52828451127885
RGD1310553	274.577720386404	-0.287997453517583	0.193817492192155	-1.48592085399627	0.137300056864132	0.52828451127885
Sptb	232.501224909158	-0.546467617902321	0.36775866861826	-1.48594082079834	0.137294774971859	0.52828451127885
Tp53il1	241.774260882496	0.488668607057433	0.32893347173649	1.48561532664243	0.137380898696226	0.528416681753403
Syde1	99.2687689785023	-0.613698112122723	0.413206539988567	-1.48520909698018	0.137488442969414	0.528472655661265
Uspl1	354.405908067058	-0.208937979973065	0.140674383941496	-1.48525960533055	0.137475067976204	0.528472655661265
Gtf3a	576.506275785034	0.194580300944237	0.131056217314404	1.48470866114988	0.137621016389127	0.528604371765585
Nfrkb	667.80726003628	-0.249463156370208	0.168039221666757	-1.48455315310211	0.137662233013033	0.528604371765585
Tbc1d14	734.157763805971	-0.152099421175625	0.102433910065205	-1.48485419602557	0.1375824517236	0.528604371765585
Prmt2	309.847095366969	-0.23443545092844	0.157967907639149	-1.48407011545641	0.137790320399434	0.528917521668343
Lace1	572.863168019145	0.231788411894907	0.156258315927026	1.4833668884743	0.137976959739468	0.529455139905062
Hmbs	1123.63116966668	0.173384533987247	0.116932739482503	1.48277150398235	0.138135129508937	0.529883186634686
Prkaa1	120.410658609496	-0.310530265195932	0.209487681614613	-1.48233186220087	0.138252014348497	0.53015262992072
Tyk2	600.209911818562	-0.178385233805253	0.120401014229825	-1.48159245124586	0.138448768821226	0.530728062874217
Kctd17	391.788724521757	0.184962396487594	0.124923011030305	1.48061109768418	0.138710236392706	0.531551094686276
Serpinb5	28.5679472333588	-0.752809078244082	0.508553951716332	-1.48029343927701	0.138794953351788	0.531597188675542
Setdb1	797.804588697921	-0.182603229302885	0.123376436773996	-1.48004946550193	0.1388600462613	0.531597188675542
Ypel4	107.237656445481	-2.02247702546956	1.36650161591061	-1.48003998086882	0.138862577263565	0.531597188675542
Eif4ebp1	88.6430055887574	0.489734887699079	0.330974370150938	1.47967616790309	0.138959688646648	0.531789838773671
Amacr	6838.26667307216	0.306415484693496	0.20713863675972	1.47927730667136	0.139066215437775	0.531842525122938
Rnaseh2b	884.156454119265	0.234174478716962	0.15830363020733	1.47927421759225	0.139067040706086	0.531842525122938
Lcn2	124.979383609703	-0.524003038258916	0.354352657078293	-1.47876141970946	0.13920409039917	0.532187585427841
Fntb	224.723525996455	-0.34050204260351	0.230320289272212	-1.47838492075301	0.139304779203996	0.532393450044592
Bap1	1743.7254620665	0.131901330939211	0.089267774926524	1.47759178547666	0.139517074388852	0.533025568908804
Col15a1	203.19083867291	-0.624464400071289	0.42267889918564	-1.47739667457832	0.139569337048723	0.533046063473048
Tfcp2l1	4835.47302849948	0.21104868421196	0.142891801368835	1.47698245938686	0.139680339221127	0.533290808057552
Comt	6885.00949928837	0.97098153420053	0.657519139535073	1.47673501167906	0.139746683137463	0.533364943096173
Igtp	312.471592288123	-0.311461518553298	0.21099488302574	-1.47615673938074	0.139901819938375	0.533777806451684
Aagab	312.982085082555	0.294068042383829	0.199268906006305	1.47573471585439	0.140015122505188	0.533851688156314
Polrmt	619.669338710009	0.160352755214735	0.108649045044706	1.47587818327123	0.139976597240214	0.533851688156314
Bnip3	3170.43071571192	0.2441925012856	0.165504379547448	1.47544434747477	0.140093120007922	0.533969953725703
Dus3l	613.968767771088	-0.187347301097616	0.127076966377216	-1.47428213340798	0.140405643633338	0.534944065709693
Gjb2	3570.24050555129	0.286550183834822	0.194407172595339	1.47396919573168	0.140489885284483	0.534944065709693
Il16	160.427460919927	-1.31844010085423	0.894405992572424	-1.47409578178499	0.140455804112199	0.534944065709693
Cds1	388.164508209514	0.265658690856118	0.180320418982299	1.47325905843306	0.140681195773258	0.535493124969476
Ubac1	559.518023076535	0.198588582529094	0.134816909454901	1.47302429147826	0.140744485865309	0.535554679057617
Atp6v0d1	7552.37538008926	0.14158425712926	0.0961538412502071	1.47247634923743	0.140892289084704	0.535828925794747
Rab5c	3919.99629598905	0.149050269310206	0.10122894654691	1.47240759085776	0.140910844553977	0.535828925794747
Fam108a1	2195.61657322976	0.238976056966212	0.162323297223097	1.47222278658967	0.140960726039196	0.53583933517107
Zbtb40	133.24743424031	-0.333811012591794	0.226792924301736	-1.47187578104366	0.141054424791076	0.53601624613018
Ankrd13d	51.3702952883476	-0.477280268665188	0.324690822647305	-1.46995306111141	0.141574467168578	0.536498073466583
Dhdds	974.32308984705	0.188349907353921	0.128111453984923	1.47020349465464	0.141506648522864	0.536498073466583
Eaf1	944.825900927562	0.151733262732925	0.103209439264645	1.47014908533566	0.141521380713365	0.536498073466583
Ephb2	104.676963328507	-0.694496977410295	0.472128285670823	-1.47099209788611	0.14129325391785	0.536498073466583
Jkamp	408.370997026662	0.187691571347956	0.12764432631323	1.47042627564483	0.141446339308476	0.536498073466583
Lmod1	44.6509384893974	1.09550932688784	0.744964089933679	1.47055320073934	0.141411988150868	0.536498073466583
N4bp2l1	46.5274530267307	-0.502813561918837	0.342045361035309	-1.47002011779055	0.141556305439537	0.536498073466583
Nr1i3	39.9041439438893	-0.476405006061422	0.323856737137777	-1.47103626829522	0.141281308803284	0.536498073466583
Slc33a1	957.466633111027	0.205881822365356	0.140071239533679	1.4698365135539	0.141606037339411	0.536498073466583
Ctdspl2	34.9233294537633	0.601328329922314	0.409416982963751	1.46874300516145	0.141902508225463	0.537348612602007
Rae1	556.680721521043	0.190008946219021	0.129391085308183	1.46848560522125	0.141972363509047	0.537348612602007
Slc20a2	703.520190859834	0.191726743661175	0.130558870488452	1.46850798374618	0.141966289195293	0.537348612602007
Slc22a9	2891.86404981707	0.32803667960096	0.223429509343986	1.46818869434083	0.142052974393781	0.537474736005232
Stradb	1873.94514561374	0.297323371812136	0.202600315636108	1.46753656764366	0.142230149258285	0.537966015464116
Slc39a9	133.246832367871	0.520664060672229	0.354855507062324	1.46725653205315	0.142306283569848	0.538074923171952
Glod5	1015.9376967513	0.201212383985534	0.137187633947588	1.46669476100452	0.14245910869103	0.538115729272929
Katnb1	226.061772497025	0.258732843586765	0.176374062658928	1.4669551729219	0.142388250144761	0.538115729272929
Timp1	77.9748214220796	-0.501350694666943	0.341784808942306	-1.46686067241676	0.142413960771568	0.538115729272929
Il13ra1	869.038143307813	-0.224723270895351	0.153313300607069	-1.46577805060306	0.142708762626383	0.538531468343224
Itm2a	20.0400882547753	0.674963374104482	0.460476618888555	1.46579293370775	0.142704706729879	0.538531468343224
Slc13a4	54.2716277605308	0.608032990240856	0.414821924744289	1.46576869247128	0.142711312920094	0.538531468343224
Tmem206	451.278982080626	0.299264576851641	0.204259317704571	1.46512080924739	0.142887960200549	0.539019102435925
Clcnkb	8703.43794831567	0.283979104641321	0.193869669631278	1.46479387508847	0.142977163448152	0.539176655524784
Nup214	824.427562182101	0.185719563924468	0.126832774627765	1.46428684911709	0.14311558886186	0.539340776858056
Scin	536.191939163875	-0.362008751631421	0.247223940229032	-1.46429488704068	0.143113393590724	0.539340776858056
Chml	23.3754818120676	-0.691120632954742	0.472260792035696	-1.46343004672406	0.143349741971632	0.540044138962403
Bcl2	23.8475765495001	0.687634093511806	0.470100673294947	1.4627379465172	0.143539098575882	0.540578328168812
Uncx	19.140963190814	0.720986380014668	0.493106100775402	1.46213234612374	0.143704946415301	0.541023657156778
Bms1	512.370968542335	-0.17692707715976	0.121050681559617	-1.46159505159518	0.14385221121702	0.541398752547234
Tor4a	132.122273371508	-0.513034884050593	0.351055599305694	-1.46140635576033	0.143903957514062	0.541414227442845
Grin3b	78.443108574393	-0.591419978436757	0.404813740407776	-1.46096814269262	0.144024184312612	0.54168725310958
Arf3	3165.82291962961	0.119313803589395	0.0817531330601646	1.45944013548188	0.144444006116525	0.541831872448984
Chpf2	436.515214446704	0.264882957265504	0.181484336725061	1.45953618943318	0.144417587582084	0.541831872448984
Notch2	163.079122070446	-0.561451112942098	0.3845335857065	-1.46008342004912	0.144267148738996	0.541831872448984
RGD1308147	480.302292577337	0.164933575431673	0.113005567909318	1.45951724753974	0.144422797038834	0.541831872448984
Slc25a40	92.1017547562543	0.381083218276614	0.260955655614009	1.46033707290212	0.14419745794141	0.541831872448984
Spata2	108.12723480144	0.377255814015358	0.258291844169264	1.46057966030137	0.144130831508816	0.541831872448984
Zfp710	207.129384399879	-0.280543701807678	0.192218999661189	-1.45950037354358	0.144427437897524	0.541831872448984
Zpbp	29.0332591452332	0.606383399577382	0.41526090971018	1.4602467638974	0.14422226726614	0.541831872448984
Cradd	28.3722453762225	0.575905189987543	0.394798807823442	1.45873082333393	0.144639208941805	0.542015028138871
Evpl	311.673626144956	-0.46608415622786	0.319548774936195	-1.4585696857105	0.144683582207754	0.542015028138871
Slc52a2	212.782262220153	0.233510178130119	0.160063050519276	1.45886372509187	0.144602618974052	0.542015028138871
Tgm2	2137.84435663541	-0.354717785538158	0.243170472220713	-1.45872063453576	0.144642014372771	0.542015028138871
Cd40	29.9356850440477	-0.689728786557458	0.473052845340134	-1.45803749697675	0.144830207773805	0.542385549112707
Ptrhd1	101.614475992293	-0.313265746211092	0.215013324293991	-1.45695968954351	0.145127507773581	0.543319912172107
Akap12	327.545114132113	-0.934467903213213	0.641778413307975	-1.45606004165301	0.145376022385646	0.544071080156487
Nudt9	934.148708694435	0.164947814291291	0.113323456514205	1.45554873955521	0.145517407298957	0.5442418069628
Rxra	144.397705223922	0.531650123971857	0.365225886086411	1.45567482105053	0.145482533552112	0.5442418069628
Btd	1926.27426553271	0.16319710467984	0.112149338315633	1.45517670572909	0.145620348000281	0.544268620641631
Golga4	2059.996147199	0.185047467400637	0.127162837513112	1.45520083555509	0.145613669655717	0.544268620641631
Scarf1	367.128548778642	-0.360549108806002	0.247856014849369	-1.45467161256958	0.1457601950595	0.544480106432312

Smpx	237.075438233742	-0.432055566362096	0.297021688720588	-1.45462632113891	0.145772740090931	0.544480106432312
RGD1562726	95.3383321051979	-0.424090351385536	0.291781569875866	-1.45345146907654	0.146098444963179	0.545517386810607
Ldhc	27.0418157454268	-0.654818647416198	0.450853951960869	-1.45239637928921	0.146391422483293	0.545536035391839
Mrpl15	1624.35587105389	0.140257676575267	0.0965637422227981	1.45248799753074	0.146365964105184	0.545536035391839
Pomt2	390.060486448126	0.364339244608515	0.250805878969247	1.4526742598932	0.146314216978277	0.545536035391839
RT1-CE6	149.090768366501	0.634748010357648	0.436966555612198	1.45262378139754	0.146328239454351	0.545536035391839
Ube2s	1121.6923936751	0.181052355168986	0.124654522253826	1.45243310788453	0.146381216134376	0.545536035391839
Yes1	113.687547805066	0.361345789549585	0.248706229013644	1.45290204826258	0.146250952188366	0.545536035391839
Nin	102.773891561986	-0.355719770454407	0.245007075473344	-1.45187550101225	0.146536225693052	0.545896670346517
Isca1	2900.84785896957	0.152140222474657	0.10482943029979	1.45131211759491	0.146692968578438	0.546301533703317
Hif1an	98.6736406578998	0.33557049891413	0.231282312974401	1.45091293233163	0.146804106337314	0.546536348715987
Nrbp	1145.01069443649	0.126584324245661	0.0872737839500373	1.45042782054836	0.146939253696544	0.546860365918443
Cog6	263.979417296545	0.260584389063564	0.179705114445634	1.45006662646988	0.147039940583879	0.547055962250858
Fam160a2	420.415120979969	0.205148725664823	0.141518105415896	1.44962883061449	0.147162051790147	0.547331112777098
Sigmar1	997.50671317557	0.146831923366574	0.101321534179038	1.44916798345273	0.147290676281242	0.54763029984056
Atp1a1	57599.5099285296	0.222843798238305	0.153825199948175	1.44868199952533	0.147426409644205	0.547955713543505
Fip1l1	603.178138537746	0.152262877284076	0.105139806747723	1.44819438035893	0.147562695800774	0.548103791003789
Tspan5	1457.52246271914	0.129925553850773	0.0897073589635092	1.44832659607807	0.147525732917593	0.548103791003789
Rnf138	337.527096795767	-0.192914008119581	0.133260559924184	-1.44764518646278	0.147716306944947	0.548315984564423
Tmtc4	215.373392623421	-0.337812634904368	0.233338192514272	-1.44773828606607	0.147690258114016	0.548315984564423
Rnasel	43.9375056930183	-0.65224242622276	0.450745242371579	-1.44703119392012	0.14788818711164	0.548774774636273
Kit	127.334608180007	-0.552470518137105	0.381877764778563	-1.44672083345168	0.147975127089911	0.54891817705742
Cutc	333.665113578688	0.204234801711046	0.141213013385523	1.44628881442724	0.148096211775092	0.549188105395008
LOC500035	239.777625895769	0.45746855995963	0.316360038380504	1.4460377559109	0.14816661228959	0.549269965845884
Gtf2a2	234.184682680312	-0.259156748308385	0.17938682899797	-1.4446810267844	0.148547501624622	0.549785380483703
Shisa5	1130.88631755724	-0.249464095048561	0.172621140556652	-1.44515378732937	0.148414693619632	0.549785380483703
Tp53inp2	2583.7017533489	0.275416159886712	0.190556254192111	1.4453273184571	0.148365967973082	0.549785380483703
Zfyve27	631.883414250007	-0.186324285005194	0.128964906289411	-1.4447673430403	0.148523246871387	0.549785380483703
Zhx3	775.65168565385	0.327424986834408	0.226608794372094	1.44489090876488	0.148488530330923	0.549785380483703
Rps29	9211.80445389059	-0.18983862334956	0.131538941966136	-1.44321233326047	0.148960666308114	0.551135069419929
Casp2	266.069171448789	-0.333922372848159	0.231491686685643	-1.44248105678893	0.149166711799643	0.551179728970982
Ccdc50	103.766636099018	-0.355940654571476	0.246713160758684	-1.44273071398745	0.149096343593008	0.551179728970982
Ocel1	663.204744045198	0.176746417718303	0.12252043150415	1.44258729379611	0.149136764812018	0.551179728970982
Socs5	477.942814207576	-0.325613898233983	0.225721521923478	-1.44254697318748	0.149148130186405	0.551179728970982
Chst7	14.7352797614764	-0.931535718599518	0.646112412721418	-1.44175487153373	0.14937153797913	0.551592362000273
Slc25a39	6599.87970335774	0.200407293997632	0.139003669500979	1.44174103257196	0.149375443448605	0.551592362000273
Nav3	36.1884799370043	-0.782466126616934	0.54295869066909	-1.4411153925038	0.149552085654965	0.552065282739308
Copg2	1202.40266572734	0.199582196783269	0.138524336697997	1.44077352428251	0.149648675492236	0.552073770996968
Mthfs	1194.12649867965	0.165110264335424	0.114599150401677	1.44076342413275	0.149651529864654	0.552073770996968
Gstm4	735.804943482853	0.301309903548862	0.209196350905778	1.44032102971323	0.149776594356418	0.552355863548037
Efha1	2172.02619958817	0.154702574087505	0.107457682395368	1.43966043784853	0.149963491458294	0.55260339242615
LOC688090	128.016861677436	-5.28623411394419	3.67253483357979	-1.43939658941001	0.150038190130838	0.55260339242615
Ngly1	987.577761446333	0.161418814359798	0.112130292484281	1.43956473120255	0.149990583883959	0.55260339242615
Scamp5	71.9238845964888	0.480961060342516	0.334048419588854	1.43979444936301	0.149925562047657	0.55260339242615
C4a	3551.53504264068	0.252524403154454	0.175496224793212	1.43891644080666	0.150174198791692	0.552636946814845
Cbr1	133.338649078551	-0.393644005947571	0.273615095555084	-1.4386779543321	0.150241788285929	0.552636946814845
Pde1c	82.7810659280504	-0.404093567995423	0.280874962085603	-1.43869558537677	0.15023679067449	0.552636946814845
Tshr	191.670799007308	-0.42505423302956	0.295371132951631	-1.43905136829727	0.15013596923939	0.552636946814845
Dnajc30	399.038957486551	0.212058114081505	0.147417589482373	1.43848583351623	0.150296254148997	0.552658435670494
Apobec3b	15.9376686262725	-0.9588126503813	0.667000088059086	-1.4375000356767	0.150575962694826	0.553276588217911
Astl	16.7251084111501	-0.732543962573752	0.509824638866841	-1.43685476677223	0.150759264932158	0.553276588217911
Dgat1	754.911963666475	0.150291074218206	0.104613097326144	1.43663726683911	0.150821088684583	0.553276588217911
Fitm2	469.337857932123	0.261156777307533	0.18172412113122	1.43710573853295	0.150687950911403	0.553276588217911
LOC500684	450.109638253968	-0.222360712273732	0.154734563296566	-1.43704617466463	0.150704873750732	0.553276588217911
Tcf4	96.0344621427958	-0.353816008040929	0.246286036297052	-1.43660604295967	0.150829965571531	0.553276588217911
Tlr2	32.1123389284091	-0.874003047543818	0.608151495199434	-1.43714691888935	0.150676251904463	0.553276588217911
Zmiz1	1384.44555950872	-0.144421268061545	0.100535350024916	-1.43652225834747	0.150853787338317	0.553276588217911
Hnrnpm	1285.09558706282	-0.13752514356054	0.0957869482983935	-1.43573989988829	0.151076367087227	0.553566315468268
Sh3bp1	321.223714930274	-0.291980902580007	0.203391646937245	-1.43555995035575	0.151127597826679	0.553566315468268
Tec	254.517956772923	-0.278925871393689	0.194264091130763	-1.43580766661574	0.151057077666474	0.553566315468268
Vom2r44	17.2660446937197	-0.974352669517566	0.678669292039886	-1.43568109084314	0.151093108274828	0.553566315468268
LOC679038	934.248731973514	0.159078530123257	0.11088738658063	1.43459535866675	0.151402437973065	0.554215816425719
Mettl23	305.746920511161	-0.241423851916683	0.1682760123411	-1.43468964208227	0.151375557115985	0.554215816425719
Ccdc8	156.231424687856	-0.493984448439584	0.344560085870873	-1.43366706910071	0.151667293959927	0.554988020229302
Ifrd2	416.999773087684	0.242334098387974	0.169049012874426	1.43351383286684	0.151711048640898	0.554988020229302
Tex10	240.314290048019	-0.289697991540777	0.202254724356903	-1.43234227265599	0.152045890701043	0.555855128243181
Tshz3	32.0011206965899	-0.723406420357202	0.50503519670324	-1.43238812874715	0.152032774061044	0.555855128243181
Cdk20	441.008988890659	0.271397169374964	0.18954509000724	1.43183434276561	0.152191236194986	0.556028798004566
Pttg1	52.0978784490865	0.508661610263152	0.355245970604675	1.43185750818607	0.152184605046998	0.556028798004566
Nrep	100.866787357493	-0.467022976467601	0.326243821461558	-1.43151516057947	0.152282625130063	0.556183906564364
Esrp2	1369.00831333569	-0.199935053927793	0.139687638995538	-1.43130097527226	0.152343974547953	0.556229236977844
Abi1	821.036313377704	-0.169240632827556	0.118304310368243	-1.43055339489123	0.152558252539915	0.5566539641633
Tnfaip8	2052.26224122754	0.28863978287603	0.201748183490616	1.43069334197726	0.152518122249091	0.5566539641633
Chuk	1642.96498752488	0.262428012825368	0.183600052058367	1.42934606980363	0.152904790758792	0.5575604272584
Ntan1	1245.57429226704	0.152601049326603	0.106751328076065	1.42950024207537	0.152860505405635	0.5575604272584
Dtnb	604.773180493874	0.210704267653189	0.147453410647621	1.42895485921802	0.153017208203338	0.557791401038852
Ly6e	1361.33037338179	-0.639713781399311	0.447846892931354	-1.42842072033168	0.153170798731213	0.558023766854006
Slc2a9	404.785736466872	0.31532152987244	0.220752842397409	1.4283917092437	0.15317914416545	0.558023766854006
Efs	41.6352177504701	-0.739085588782421	0.517666257116114	-1.42772602738262	0.153370731636305	0.558542690092355
Herc3	819.962585840898	0.335428268030512	0.234971450213177	1.42752776018617	0.153427829401254	0.558571655661325
Pcdh7	21.534604086426	-0.896860658755469	0.628461956809162	-1.42707231366721	0.153559051888262	0.558870375844375
Slc17a5	1670.93474623174	0.243463744876281	0.170647939119092	1.42670193459747	0.153665827784964	0.559079961140815
Nup98	1562.20926585761	0.25191306760092	0.176749674556176	1.42525336034412	0.154083976727243	0.560421913434189
Cbx8	75.8064182266755	0.355138586530105	0.249250061510924	1.42482848099333	0.154206787297674	0.560509863903218
Pds5b	292.990347775677	-0.223389824623507	0.156775009421095	-1.42490710380687	0.154184055915587	0.560509863903218
Rpl34	5531.4519441714	-0.161803146063194	0.1135928895316	-1.42441262591689	0.154327061435564	0.560588488423336
Senp2	241.071314579651	0.263840301419392	0.185214717891277	1.42451045156287	0.154298761767326	0.560588488423336
Calcoco1	594.963057526852	-0.193020449437162	0.135638986474313	-1.42304550081341	0.154722965473622	0.560871121760565
LOC100360501	486.504748142665	0.226129556455321	0.158915886715358	1.42295122991921	0.154750293668937	0.560871121760565
Mtif2	420.90852168207	-0.191296437370826	0.134426417381053	-1.42305687451719	0.15471966859776	0.560871121760565
Tm9sf4	672.651071065756	0.189811851168966	0.133361522079112	1.42328797849479	0.15465269044416	0.560871121760565
Tpm1	403.576029075054	-0.212850832669728	0.149561433865652	-1.42316656886914	0.15468787442453	0.560871121760565
Zbtb25	33.0958111327532	-0.51332423753516	0.3607394202542	-1.42297794117826	0.154742549968196	0.560871121760565
Zc3h12d	14.9641937682315	-1.04194312221653	0.731821153566075	-1.42376742888514	0.154513807212043	0.560871121760565
Cat	39939.232606681	0.26862723825672	0.18880908591149	1.42274529300273	0.154810005486672	0.560882916017338
E2f3	26.7854742939583	0.584074856175743	0.410568674162912	1.42259965976845	0.154852242694211	0.560882916017338
Lonp1	4275.80255004781	0.146664514658752	0.103124541606755	1.42220767601595	0.154965971071266	0.56111603287544
Asgr2	815.271434223531	0.355848725512356	0.250391345823425	1.42117022592026	0.155267278200339	0.56131294021153
Mmd	382.362163882787	-0.361129327669553	0.254095869216225	-1.42123257959085	0.155249156240114	0.56131294021153
RGD1308601	207.67374318122	0.263459129836189	0.185324809629821	1.42160744890249	0.155140241139223	0.56131294021153
Rhbdd2	376.090187068963	0.345632853721925	0.243187435837456	1.42126114587985	0.155240854504182	0.56131294021153
Xkr5	14.7074348475808	0.900774675245522	0.633685971025413	1.42148432572669	0.155176007134045	0.56131294021153
Pik3cb	519.959016967481	0.265553793340147	0.186951969663687	1.42043859616916	0.155480033071709	0.561724747582024
Tusc5	77.7960531530868	1.83438213459091	1.2914102060721	1.42044884419048	0.155477051462638	0.561724747582024
Abr	406.973564339522	-0.269113950618255	0.189707796740491	-1.41857085076154	0.156024169108375	0.5619977201962

Ak1	305.822702429498	0.371731049718282	0.261788714619236	1.41996590746455	0.155617606593871	0.5619977201962
Bcl7b	954.425699732636	0.140522747448557	0.0989893292732952	1.41957470042649	0.155731535169766	0.5619977201962
Chp	2976.52957500815	0.195826763964227	0.138136208658889	1.41763528813649	0.156297271998961	0.5619977201962
Coq3	618.957790902631	0.184855788717318	0.130365642746969	1.41797934503426	0.156196795194141	0.5619977201962
Lsm4	956.459081902531	0.157775974132646	0.111241783776246	1.41831575130079	0.156098600029515	0.5619977201962
Morc2	562.818699538757	-0.161936225354107	0.114204998884246	-1.41794340822365	0.156207287723873	0.5619977201962
Npr1	276.887350985147	-0.531403212290627	0.374463140886474	-1.41910686064488	0.155867864079684	0.5619977201962
P2rx1	16.397404944937	1.13413805076054	0.799015508856542	1.41941932063821	0.155776802932233	0.5619977201962
Rab32	225.598519105316	0.382539826748227	0.269548029571292	1.41918984663566	0.155843675286592	0.5619977201962
RGD1563349	16.776821822649	0.766444384858357	0.540245114658893	1.41869748390466	0.155987231084894	0.5619977201962
Slc29a2	100.845349327608	-0.531406562002745	0.374831365775344	-1.41772170240749	0.156272031360689	0.5619977201962
Slc39a3	469.316875527918	0.199639560780945	0.14073677512791	1.41853158564633	0.156035623822268	0.5619977201962
Smarcc1	659.468390158978	0.179219100710739	0.126372169055426	1.41818489031501	0.156136792074384	0.5619977201962
Tlr4	21.7951979075496	-0.846493482690492	0.596854062526287	-1.41825872660992	0.156115241930132	0.5619977201962
Kif20a	191.451496663584	0.301374949600057	0.212758926206975	1.4165090742509	0.156626509263485	0.563003448541674
Fxyd1	63.1389679376275	0.609207383392883	0.43013711782865	1.41630972576416	0.156684841526549	0.563035064429578
Cog8	785.103717777567	0.137230052550126	0.0969506247824409	1.41546331298094	0.156932697673069	0.563569365482498
Rapgef2	448.623219084418	-0.193733193992798	0.136859991690226	-1.41555754607454	0.15690508857961	0.563569365482498
Serpinf1	5950.92794624235	0.304293750968107	0.215054771426547	1.4149593098986	0.157080426673006	0.563743646847297
Sf1	1397.33208312867	-0.192862047474176	0.136300028566534	-1.41498170985365	0.157073858755396	0.563743646847297
G6pc	2399.34713690164	0.881730661610024	0.623397587779036	1.41439536965702	0.157245848868104	0.563981167003745
Neil1	294.362724459108	0.206681209758836	0.14611498047769	1.4145107440944	0.15721199501779	0.563981167003745
Slc37a4	4034.53218794221	0.180120073992505	0.127364780002065	1.41420629776603	0.157301339407399	0.564002215679651
Gpd2	157.074229862767	0.534556756449901	0.37809123926943	1.41383005192821	0.157411807608595	0.56422031071564
B4galt1	160.666306866287	-0.401653260481912	0.284293347436162	-1.41281273059724	0.157710794077742	0.564402042030105
Eml1	219.327139445839	0.429853320695671	0.304171641217315	1.41319328447376	0.157598900562194	0.564402042030105
Hspd1	16040.6207700356	0.210118701359037	0.148676035041946	1.41326543514397	0.157577693025769	0.564402042030105
Shisa4	26.7575984511682	0.75968153891769	0.537683350190764	1.41287904609314	0.157691291132555	0.564402042030105
Thada	2005.7482778593	0.244377034597936	0.172932923997589	1.41313191813806	0.15761693991764	0.564402042030105
Acvr1c	21.0437749549308	0.912767710952372	0.646660125726852	1.41151073746261	0.158094071813823	0.564511805943071
Adrb2	24.1580392088486	0.640116378188876	0.454250162934092	1.40917148835839	0.158784466267816	0.564511805943071
Agpat5	343.374558200951	-0.324332825936846	0.229970421134861	-1.41032409444799	0.158444007131804	0.564511805943071
Capn6	50.8459829679535	0.546709082855363	0.387943703670597	1.40924850096181	0.15876170087936	0.564511805943071
Ccna2	39.0813892993047	0.544243807209822	0.385500838914926	1.41178371684407	0.158013654381709	0.564511805943071
Chst14	77.8750827818786	-0.473134973443892	0.335461841827151	-1.41039878296405	0.15842196458059	0.564511805943071
Clybl	500.693173784258	0.26944483920589	0.190986704607656	1.41080416963794	0.158302364698171	0.564511805943071
Cnpy4	186.149577016286	-0.445752689837893	0.316215428609726	-1.40964876950404	0.158643418879383	0.564511805943071
Erbb3	993.569949288536	0.231524330963729	0.164187957708265	1.41011761273692	0.1585049574492	0.564511805943071
Frs2	384.857080386588	-0.200709244748618	0.142340439491646	-1.41006480987012	0.158520546916606	0.564511805943071
Klhdc2	852.12038334199	0.23600814186999	0.167447255513534	1.40944765649094	0.15870284076308	0.564511805943071
LOC100365935	19.1388792667146	0.713325017988506	0.505279974985151	1.41174210992523	0.158025909428771	0.564511805943071
Mtmr7	144.476980560121	-0.378536792449997	0.26849341932002	-1.40985501025936	0.158582499419919	0.564511805943071
Pdgfa	485.11725754388	-0.206718934272167	0.146498344408158	-1.41106669230492	0.158224949998272	0.564511805943071
Slc17a2	614.780842141535	0.279508234374337	0.197989224691642	1.41173457701881	0.158028128274278	0.564511805943071
Slc22a15	743.509071567609	0.196006474685862	0.139042104338184	1.40969151480279	0.158630791304429	0.564511805943071
Slc4a3	451.264005450726	-0.300258347440136	0.212636744252235	-1.4120717870095	0.157928824934028	0.564511805943071
Tnfrsf10b	83.6149037091281	0.430233679897177	0.304759818735502	1.41171392502557	0.158034211516455	0.564511805943071
Trem3	40.7961442458034	-0.520644163128851	0.369181389092328	-1.41026654785852	0.158460992241711	0.564511805943071
Ube3b	1188.2197904166	0.14232126487225	0.100915463794078	1.41030184593575	0.158450573707223	0.564511805943071
Vkorc1l1	94.3044396650755	0.40462376689442	0.286810973379711	1.41076808228929	0.158313008654515	0.564511805943071
Gtf2e1	307.409825402952	0.222239907876953	0.157749796982036	1.40881263956404	0.158890576708887	0.56471241240563
Ano1	120.240744678695	-0.664416595762308	0.471902754335587	-1.40795235810346	0.159145177721049	0.565302748248845
Minpp1	2135.19703487259	0.305642423512788	0.217095085939071	1.40787352321078	0.159168524404151	0.565302748248845
Wdr35l	891.863810943024	0.187276113739813	0.133032471574094	1.40774738320567	0.15920588572449	0.565302748248845
Hoxc4	58.891226780539	-0.465616277200479	0.330870219563186	-1.40724746341688	0.159354021880278	0.565652033944798
Arl5a	283.474770631805	0.266969091985099	0.18981578386215	1.40646413355688	0.159586347736279	0.565769940227868
Kat6a	792.40736573199	-0.182202604173709	0.129514183092261	-1.40681583918817	0.159482004566368	0.565769940227868
Pctp	195.87339666591	0.302798641496208	0.215277653921338	1.40654933747489	0.159561064907322	0.565769940227868
Rnf185	1448.77336130345	0.154893400933763	0.110117315863983	1.40662165362882	0.159539608682542	0.565769940227868
Celf4	302.860291911512	-0.26194475973029	0.186276637949335	-1.40621369708844	0.159660678086237	0.565856958880003
Thrap3	1858.53924345778	-0.177667685555088	0.126379612901507	-1.40582552419711	0.159775940791573	0.566088947330743
Prkcd	1143.76937597217	-0.194519610825888	0.138403096134142	-1.40545707617232	0.159885404665553	0.566300252237044
Lrp5	1068.02289857136	-0.321563721432586	0.228845890086102	-1.40515401570725	0.159975484754575	0.566442791190187
Clip2	283.317156360761	-0.424309726104534	0.302110436867211	-1.40448549379654	0.160174328350531	0.56670426233426
Foxo3	310.820240319651	0.48902831918768	0.348194203152951	1.40447001920036	0.160178933289822	0.56670426233426
Lphn1	958.779631338436	0.254258114613027	0.181043570241783	1.40440289745427	0.160198908576454	0.56670426233426
Bckdha	6555.81420666961	0.193169500027806	0.137608479376683	1.40376160613644	0.160389850440841	0.567026762087277
Rcor2	24.5059040298619	0.757298095642427	0.539476793928327	1.40376398793354	0.160389140952391	0.567026762087277
Tmem27	14814.5288652274	0.331453251407351	0.236175469119036	1.40341946876919	0.160491790783749	0.567210725761224
Kank2	450.309773721371	-0.46719893798189	0.333140804751348	-1.40240682413732	0.160793796720574	0.567927478702262
Osmr	111.386292593257	-1.40029825915254	0.998498215993403	-1.40240436760259	0.160794529866609	0.567927478702262
Galnt7	105.292709865613	0.365522077082099	0.260737458338607	1.40187788671091	0.160951714924845	0.568306055245664
RGD1305928	31.5343551594466	-0.700920781505364	0.500094698909749	-1.4015761075521	0.161041865817495	0.568447778534672
Kcne1	68.5054111707992	0.776378700528331	0.554083935184186	1.40119330525302	0.161156275735504	0.568675017078465
Pspc1	377.199026937377	-0.185953287421962	0.132794568658793	-1.40030792900693	0.161421127482616	0.569324529864807
Rad54l2	234.273156558281	0.348277435685445	0.248726399918497	1.40024314186017	0.161440520830043	0.569324529864807
Hsd3b7	161.770054502132	-0.340475736222389	0.243286451689051	-1.39948498512181	0.161667597818305	0.569477413921713
Naprt1	6442.39738482806	0.187940529721683	0.13427069285512	1.39971371060454	0.161599066420576	0.569477413921713
Nfkbil1	233.839849856907	0.231306530470949	0.165242693066285	1.39979884241031	0.161573564582206	0.569477413921713
Pragmin	180.289771864006	-0.439272862401577	0.313915865733768	-1.39933310275602	0.161713117378322	0.569477413921713
Vegfa	1322.51708629826	-0.246329995243416	0.176042779920536	-1.39926213023112	0.161734391354856	0.569477413921713
Abca17	151.517553424663	0.316126041310513	0.22596730258917	1.39899019764492	0.1618159225598	0.569588038344592
Ethe1	5141.85899717733	0.326753707326934	0.233624387092318	1.39862841971979	0.161924439410685	0.569617201591409
Rwdd4	1011.77910810044	0.141327992219688	0.101047009054076	1.39863607584917	0.161922142352815	0.569617201591409
Uba1	3333.35065775209	0.138871404889587	0.0993190613041336	1.39823517324975	0.162042457684385	0.569855994443292
Csnk2a1	408.256988694085	0.277961973791246	0.198826999058849	1.39800919948993	0.162110304653229	0.569918256321867
Ranbp10	790.537311032088	-0.163718677237168	0.117139918804058	-1.39763352159245	0.162223146640298	0.569962375491073
Rpl39	2630.76001212055	-0.190717811631089	0.136444863708276	-1.39776468272818	0.162183743170867	0.569962375491073
Dfna5	201.00627136638	0.308511343670017	0.220909578890678	1.39655032262177	0.162548837851106	0.570305084312281
Pcgf1	121.45545811105	-0.288082957273328	0.206251467848473	-1.39675591295658	0.162486984009653	0.570305084312281
Rcsd1	274.815374114058	-0.560100334331882	0.401081597247875	-1.39647477763915	0.162571570752401	0.570305084312281
Slc25a35	365.654210586982	0.226257651000899	0.162006541604229	1.39659577175366	0.162535162510853	0.570305084312281
Snrpc	1215.0886826505	-0.215461341927868	0.154223709360634	-1.39707015750761	0.162392474813366	0.570305084312281
Zfp57	18.8980662928718	-0.770790121373268	0.552028475475354	-1.39628688666746	0.162628121068447	0.570327437230474
Bucs1	768.045717448079	-0.291656182640859	0.209085567284468	-1.39491303215612	0.163042066715861	0.570861527290264
LOC498662	2215.84321153292	-0.186931899571039	0.133955268629808	-1.39548000973098	0.162871138830172	0.570861527290264
Phrf1	875.412962265344	-0.156736376940089	0.112373434664127	-1.39478140370594	0.163081768353993	0.570861527290264
Rangrf	540.260565256835	0.207991880696417	0.149053865896747	1.39541419771358	0.162890972377129	0.570861527290264
RT1-T18	17.3934502255669	-1.26114722390029	0.904061638866263	-1.39497924663835	0.163022097927243	0.570861527290264
Sestd1	1200.85355511376	-0.171442592157515	0.12289095570465	-1.39507900458966	0.162992016670386	0.570861527290264
Det1	61.941125059782	-0.430535141586178	0.308720661238226	-1.3945783215784	0.16314303607657	0.570900168733465
Lama1	286.475049785242	-1.04877886356318	0.752431883424712	-1.39385223655016	0.163362230184022	0.571491261394766
Dvl1	2330.49629241185	-0.221690467892408	0.159105730049306	-1.39335313582803	0.163513029867558	0.571737801813076
Slc14a1	101.391087135241	-0.807861142031907	0.5798243266541	-1.39328604353961	0.163533309316761	0.571737801813076
Rfc2	304.45707753968	0.194211717709404	0.139439255809221	1.39280517944761	0.163678711749413	0.572070183808066

RGD1304884	136.295574832079	-0.55597411461357	0.399321503358157	-1.39229695856101	0.163832492145735	0.572431634100344
Abl2	48.0174908822135	-0.522596937976443	0.37545775149737	-1.39189279191138	0.163954864915354	0.572603656134934
Lyrm7	380.765859168733	-0.233259409645324	0.167612202458097	-1.39166126466025	0.164024997294256	0.572603656134934
Strn4	476.872029977816	-0.186657285087198	0.134128017685901	-1.39163530713105	0.164032861549828	0.572603656134934
RGD1312005	11.5464657034947	0.947469421685276	0.681047077873612	1.39119519408775	0.164166244199902	0.572893316418817
Chchd10	8614.07764098303	0.223942371933643	0.161012335037976	1.39083985013213	0.164273995951863	0.573093381825927
Lox	14.8464904947597	-1.13916814096012	0.819168082830316	-1.39064029084747	0.164334532121731	0.573128656672473
Ccdc86	311.873170173649	0.235282526417832	0.169444557373214	1.38855168950399	0.164969116470629	0.574988953635439
Zranb2	1076.94489856142	-0.20807538521357	0.149845031798841	-1.38860383100923	0.164953251781555	0.574988953635439
Alkbh6	78.3289469138994	-0.405127887249735	0.29187917362319	-1.38799860990681	0.165137468406014	0.575146855950131
Alpl	7787.02762188052	0.184135852095871	0.132719527374791	1.38740587566955	0.165318034347095	0.575146855950131
Desi1	337.743863094442	0.204890316014027	0.147631461521976	1.38784994676441	0.165182742034222	0.575146855950131
Gapdh	42287.7421814689	0.225199420281346	0.162254502453032	1.38793942156726	0.16515549240611	0.575146855950131
Nrn1	9.23305860780565	1.12826628649973	0.813123199716938	1.38757114160867	0.165267674085871	0.575146855950131
RGD1309410	543.830573211141	0.148401302112776	0.106959612963363	1.38745174932156	0.165304054452256	0.575146855950131
LOC360231	247.408842217881	-0.272806863945335	0.196677296014254	-1.38707857731359	0.165417803626314	0.575317856798253
Adat2	43.8122658106494	0.442715524781713	0.319326227317699	1.38640514592387	0.165623226014355	0.575462588684416
Bcap29	362.878639931794	0.225627317686807	0.162731929771112	1.38649690939055	0.165595223349652	0.575462588684416
Glyat	13064.6954883217	0.202851129512243	0.14635269802994	1.3860429786593	0.165733780251992	0.575462588684416
LOC500124	936.991482078383	-0.246255049985827	0.17769764019006	-1.38580934289527	0.165805128752419	0.575462588684416
Ptma	14378.7216463346	-0.188226050406584	0.135842953616822	-1.38561511948217	0.165864458959189	0.575462588684416
Rbm10	979.610806550518	-0.17546044862766	0.126618509842613	-1.38574090664752	0.165826032426178	0.575462588684416
Rpl26	9268.85192576039	-0.243665900214983	0.175841240321401	-1.38571531780379	0.16583384898111	0.575462588684416
Thap6	44.7685120580434	0.54087690599835	0.390308315620999	1.38576833839112	0.165817653233431	0.575462588684416
Nthl1	153.673335961884	-0.280792642778725	0.202709173559258	-1.38519948480102	0.165991478210703	0.575727537791532
Adamts2	16.9977743282237	-1.17383671010602	0.848058221268807	-1.38414637187268	0.166313640325606	0.5759011786743
Adrb3	18.1991206184505	2.96883907345646	2.14532913747633	1.38386181476511	0.166400770995819	0.5759011786743
Ap1s3	618.45666997254	-0.198412882026725	0.143387817527023	-1.38374992693736	0.16643504016647	0.5759011786743
Il12rb2	77.718477533075	-0.446872020858529	0.322681647454112	-1.38486965213006	0.166092328157997	0.5759011786743
Ppap2b	1468.2389180876	-0.396657666623676	0.286655980549591	-1.3837411166625	0.166437738815938	0.5759011786743
Sgk2	855.170600316676	0.261153913449457	0.188646671770157	1.38435473575511	0.166249861589998	0.5759011786743
Slc16a10	182.060088988639	0.285242490132943	0.206151142738315	1.38365708937653	0.166463478620847	0.5759011786743
Tcirg1	282.76179137241	-0.293697248892823	0.212278653494406	-1.38354584438026	0.166497560542297	0.5759011786743
Zer1	1106.77726701451	0.188256275726829	0.13600120017314	1.38422510600763	0.166289538192094	0.5759011786743
Tnfrsf21	7384.99088592101	0.271635170426009	0.196385734237093	1.38317160093751	0.166612255318161	0.576122571933745
Clmp	184.75646474215	-0.5013904465696	0.362596463224846	-1.38277809471817	0.166732917627409	0.576320594861448
Msl1	738.913080833247	-0.14727312222389	0.106514793484834	-1.38265415916016	0.166770934057655	0.576320594861448
Mdm1	163.150226589376	-0.40325884543003	0.291762840723705	-1.38214600745511	0.166926874428651	0.576464813598447
Tmem169	151.596894885356	-0.522918231267877	0.378371746759694	-1.38202240454276	0.166964821957248	0.576464813598447
Vgll4	162.285522934545	0.495848967551962	0.35877968966343	1.38204302483542	0.166958490837559	0.576464813598447
Gja7	38.4416137414571	-0.756739349357692	0.548089670678701	-1.3806852962958	0.167375744206228	0.577532698435942
Khdrbs3	410.178792507103	-0.179394567537736	0.129919864216672	-1.38080938291741	0.167337577753725	0.577532698435942
Iqsec3	29.8322447391591	0.562245953622486	0.407276285816763	1.38050255613322	0.167431963169308	0.577551348522717
Car14	16.9715644914119	0.936188994894754	0.679014651976599	1.37874638223126	0.16797296341217	0.578912519355983
Epb41l4b	1068.96755499449	0.174308212270202	0.126442192227803	1.37856050420386	0.168030301016663	0.578912519355983
Tpp1	2286.13484598641	0.157502382727976	0.114221168094682	1.37892463678375	0.167917991214181	0.578912519355983
Wls	2273.06765960331	0.16305120454182	0.118273637445696	1.37859296512025	0.168020286772119	0.578912519355983
Cnrip1	25.2073071578221	-0.776203409276422	0.56317064141264	-1.37827392303231	0.168118731209597	0.578952628819187
Rps24	6645.28677718088	-0.192981284852463	0.140028058845427	-1.37816153736366	0.168153419529154	0.578952628819187
Wiz	771.585895758647	-0.137667988342738	0.0999021967005554	-1.37802763992649	0.168194754562815	0.578952628819187
Etv5	1238.61674015221	0.246419242807131	0.178895509962952	1.37744789043707	0.168373815124236	0.579393515804439
Pxn	2815.73812810355	-0.188878062543816	0.137161500914486	-1.37704867097928	0.168497200614939	0.579543247701772
Slc41a2	274.337564435327	0.33843832924769	0.245783544165829	1.37697717069027	0.168519306145905	0.579543247701772
Cbll1	336.55307776182	-0.198525690899032	0.144228565779123	-1.37646581886602	0.168677462713226	0.579736329361516
Rab3b	15.4572996918794	0.753300017835067	0.547261811928774	1.37648928066099	0.168670203751707	0.579736329361516
Lphn2	315.838804585256	0.306399729721658	0.222678898900537	1.37597110114379	0.168830580484692	0.580087175873341
Slc26a7	30.6465795982612	-0.684487194051726	0.497795764539455	-1.37503619518537	0.169120223586538	0.580906757716709
Praf2	159.687658555131	-0.312994206196865	0.227672665825806	-1.37475530960901	0.169207317488558	0.581030323436541
Klk1c10	10.1945492689068	1.06839206269092	0.777473567175549	1.37418441963531	0.169384436423475	0.581111833501123
Lactb	2193.0484717109	0.185437758442542	0.134940440666693	1.37421930391186	0.169373609570141	0.581111833501123
Pls3	1158.93896677462	-0.264677362670767	0.192591210721656	-1.37429616688632	0.169349755836065	0.581111833501123
St3gal4	137.327904289227	-0.32548899867392	0.236980729753444	-1.3734829790277	0.169602249093148	0.581683513335159
Myl6l	429.017730722895	-1.30506247519088	0.950538782362925	-1.37297130785832	0.169761266621076	0.581877731126401
Spire1	1124.71036221604	-0.192660495100491	0.140314564131684	-1.37306128050743	0.169733296761101	0.581877731126401
Atp6v0d2	1046.85271868984	0.25720459671019	0.187474903800798	1.37194147854309	0.170081656544562	0.582098195810145
Cxcl11	213.683565257382	-0.387197147621435	0.282187903421472	-1.37212525032699	0.170024450157452	0.582098195810145
Erap1	567.425402410629	-0.185931098686135	0.13551711340724	-1.37201194750509	0.17005971853005	0.582098195810145
LOC687813	26.1129771498491	-0.630334201660635	0.459338086432327	-1.37226635517301	0.169980535366353	0.582098195810145
Taf11	467.108205920647	0.174622690837561	0.127280530765962	1.37195130933772	0.170078595947664	0.582098195810145
Akap1	794.197622760393	0.15525091833602	0.113241661839666	1.37096997530673	0.170384315882764	0.582450019405231
Pcdhga8	21.0674726706484	0.687239349576997	0.501187345188086	1.37122247035006	0.170305615513952	0.582450019405231
Ppfibp1	1045.49911886696	-0.211929421521991	0.154542937431961	-1.37133035675147	0.170271996628324	0.582450019405231
Syt11	33.1727857503897	-0.682332356383182	0.497706398267001	-1.37095355566865	0.170389434675558	0.582450019405231
Atp6v1g2	32.7018715284876	0.504664336557275	0.368801498049981	1.36839014815737	0.171189986369749	0.583456001368012
Avpr1a	42.7293821605902	-0.74013001336329	0.540409376871513	-1.36957285539341	0.170820277861476	0.583456001368012
Chrne	94.107460445922	-0.381997670139516	0.279080382557605	-1.36877291996927	0.171070268251462	0.583456001368012
Dnah1	79.4841277463476	-0.344588361311038	0.251669312650904	-1.3692108810621	0.170933365706814	0.583456001368012
Hoxb3	486.967080804755	-0.179959455672117	0.131492934282863	-1.36858650735555	0.171128563998406	0.583456001368012
Lrrc41	1003.60524021894	0.184613963607643	0.134917419374223	1.36834787134177	0.171203212978503	0.583456001368012
Neurl3	661.275682845882	-0.188049108430299	0.137330878313758	-1.36931410283902	0.170901111495784	0.583456001368012
Pdcd6	1514.18004648782	-0.150052767306941	0.10968674961111	-1.36801179576336	0.171308383883481	0.583456001368012
Plekhn1	60.5735064120203	-0.510051746691441	0.372837373761515	-1.36802740976739	0.171303496593097	0.583456001368012
Sult1c3	17.4842638629607	1.05754331086787	0.773046712142853	1.36801993237433	0.171305837055398	0.583456001368012
Tmem178b	12.5177829919447	1.63840361836605	1.19777249618927	1.36787547182679	0.171351058645647	0.583456001368012
Usp8	1921.89679661086	0.216042895789608	0.15791163950605	1.36812521524945	0.171272885181161	0.583456001368012
Zbtb43	609.895641434787	-0.151326948707339	0.110604077785284	-1.36818598136237	0.171253868509366	0.583456001368012
Terf1	180.950266438366	-0.289179891107267	0.211440382727308	-1.3676663245555	0.171416545451506	0.583504179575267
Klhdc9	136.82698886499	0.295733449052654	0.216262380944161	1.36747522967951	0.171476396190007	0.583533149429826
Mcrs1	1381.52514392945	0.169876160425193	0.124282941679265	1.36685017372368	0.171672272406283	0.584024857279201
Tex261	1154.44392503766	0.114154691277975	0.0835353998820571	1.36654270451987	0.171768686655527	0.584178004945159
Zfp865	214.126127922437	-0.240098270209459	0.175744239028641	-1.36618003262303	0.171882463100878	0.584390091416268
Cdk9	1450.0398377055	-0.164923412764135	0.120777121492258	-1.36551865722936	0.172090093182592	0.58492105236643
Rnf187	1986.56580323065	0.162109912036748	0.118732897144279	1.36533274211072	0.172148492558081	0.584944623741449
Tmem140	1334.26418846309	-0.232379115649373	0.170230692313739	-1.36508353746863	0.172226795598436	0.585035791623378
Rgl3	1114.45081464533	-0.183554319456433	0.13452401047567	-1.36447254885871	0.172418888174827	0.585513320285354
Glul	71347.2375464986	0.214060477405791	0.156930989744771	1.36404210381859	0.172554314704694	0.585798190242996
Mapkap1	1063.63361525047	0.121862702621709	0.0893625585223752	1.36368860333376	0.172665592435687	0.585890949127784
Ndufv3	1815.10754136627	0.201558376005283	0.147810402393244	1.36362781469903	0.172684733378328	0.585890949127784
Mpv17l2	602.541942290508	0.177168534803787	0.130050244411591	1.36230835709216	0.173100591328178	0.586821102585703
RGD1562018	710.761622511739	-0.14374282214006	0.105517396848942	-1.36226656866678	0.173113774148325	0.586821102585703
Sox12	29.1765891346695	0.548346536144112	0.40244895940592	1.3625244228574	0.173032441933954	0.586821102585703
Sh2b2	38.1939454431783	0.756837395462366	0.556189619712002	1.36075426192646	0.17359136042607	0.588135918486012
Trib3	17.3540693316344	0.723406719036573	0.531638893237073	1.36071067831748	0.173605138704959	0.588135918486012
Rbm7	518.861861937025	-0.216911176098092	0.159453992456493	-1.36033706498303	0.173723284267743	0.588360801247668
Cox19	1427.22136428741	0.224845496991707	0.165337650048306	1.35991709647509	0.173856160158864	0.58863542340353

Dysf	405.315019663552	-0.44576406990517	0.327998765826334	-1.35904191219179	0.174133307944687	0.58887211487632
Imp4	1296.1675869414	0.133389520198603	0.0981483016969631	1.35906090978984	0.174127288404767	0.58887211487632
Rasgrp1	50.4972254631541	-0.599288900256237	0.44086182006673	-1.35935767847968	0.174033275062509	0.58887211487632
RGD1307439	62.244910160347	-0.416032112383738	0.306069357983221	-1.35927397347155	0.174059788129363	0.58887211487632
Kctd8	40.4302132924795	-0.590389642008685	0.434506691140575	-1.35875845883735	0.174223140852791	0.589000660003814
Pcbp4	1860.68181587233	0.211064208847654	0.15539233879509	1.35826650454098	0.174379134673099	0.589177540039964
Pip5k1c	316.099687324623	-0.235913216779642	0.173667363523135	-1.35841998170378	0.174330457400249	0.589177540039964
Cyb5r4	1020.17519021528	0.160518841899507	0.118245680461271	1.35750279649396	0.174621505497247	0.58929573381286
Pcgf6	156.904077785473	-0.304729117505114	0.224465980154331	-1.35757372807941	0.174598984019782	0.58929573381286
Sms	693.49880165251	0.350128000204301	0.257865650277883	1.35779232257958	0.174529591750581	0.58929573381286
Trim29	78.6597444940881	-0.702775924812086	0.517694096580742	-1.35751195436411	0.174618597661296	0.58929573381286
Scel	25.6347436634061	-0.637905276046544	0.470046748024456	-1.35711028472716	0.174746171059038	0.589541401085493
Bnip3l	2807.63223453454	-0.172442747360362	0.127100307187078	-1.35674532325517	0.174862145973285	0.589582661267385
Corin	33.4258743234546	-0.983723928999792	0.7250242838025	-1.35681514533624	0.174839953955767	0.589582661267385
Pi4k2b	337.330044578812	0.296740527446557	0.218808487582213	1.35616552504648	0.175046508325617	0.590029244848446
Asic1	201.54076838296	0.38048200380804	0.280664438448065	1.35564735565331	0.175211396864199	0.590060070150661
Chchd4	492.858907697784	0.250214255825227	0.184536725105741	1.35590493264607	0.175129417886112	0.590060070150661
Samd4a	35.9575751882534	0.601089934618884	0.443365950890334	1.35574221117301	0.175181203886722	0.590060070150661
Nploc4	793.778518186842	0.167469987688041	0.123558240583881	1.35539310770899	0.175292344384727	0.590076384681938
Nt5c3	772.624881541888	0.248509237513167	0.183360238313801	1.35530603471332	0.175320073119031	0.590076384681938
Trmt11	143.701284714713	0.276998113533943	0.204484439391226	1.35461707677415	0.17553958985557	0.590640313291417
Lix1l	191.636931573675	-0.570823794164307	0.421503402089653	-1.35425667108351	0.175654504467146	0.590780935729466
Nbl1	760.168227444412	-0.260245238866904	0.192182046304659	-1.35415999502028	0.175685338972866	0.590780935729466
Gkap1	822.893239831978	-0.211927625282491	0.15652357648655	-1.35396615666204	0.175747175228228	0.590814076795043
Uap1l1	1167.94731442311	0.200523289840713	0.148133339122587	1.35366752027894	0.175842474803858	0.590897432942488
Yaf2	737.228984311331	-0.173069345001857	0.127862086022352	-1.35356265790628	0.175875947179697	0.590897432942488
Ccdc117	357.157451103037	-0.252637723651876	0.186723328008924	-1.35300568143152	0.176053815346436	0.591320232041251
Pgc	52.0933546560454	0.516297880254604	0.381701039718956	1.35262371995306	0.176175870693583	0.591555375569648
Erich2	29.3523480954282	-0.628028011093997	0.464519038234851	-1.35199627873267	0.176376505723846	0.591585489417195
Hoxd4	248.534922434219	0.298663344386366	0.220913890444556	1.3519446141904	0.176393033928812	0.591585489417195
Pax8	2978.00118883525	-0.208832859755705	0.154439481116916	-1.35219866219061	0.176311771561838	0.591585489417195
Tmem67	310.765468627527	-0.252123236009656	0.186427769729229	-1.35239098968917	0.176250270303148	0.591585489417195
Dmrtc1c	1112.43278263455	0.563904970379127	0.417225155290509	1.35156033433911	0.176516006624341	0.591660576436371
Vps41	972.624083298474	0.177080982742867	0.131020733410088	1.3515493169208	0.176519533230313	0.591660576436371
Clcn3	574.072984919499	0.193180751719731	0.14297303219178	1.35116915937408	0.176641251428317	0.591894004638636
Metap1d	83.9617530079064	0.332712415990475	0.246343328432645	1.35060453273629	0.176822148050064	0.59232553337372
Asah2	82.8214009740934	0.416318620049074	0.308363071271919	1.35009233865737	0.176986365558668	0.592566126630137
Dnajc9	618.639522456202	-0.151626138232255	0.112351623642541	-1.34956784171335	0.177154645283231	0.592566126630137
Fbxo44	340.095227970232	0.238944785242191	0.177045605415675	1.34962279736448	0.177137007711202	0.592566126630137
Mgat3	743.516051950527	0.364938595358176	0.270353295237261	1.34985813669446	0.177061492250328	0.592566126630137
Patz1	728.27819445714	-0.233039257035819	0.172637651641044	-1.34987504070297	0.177056069031454	0.592566126630137
Cd14	72.9854105371795	-0.480809524862638	0.356389222070648	-1.34911353959892	0.177300499984838	0.592879518337059
Nxt1	208.109090811163	-0.255333923074651	0.189302826536026	-1.34881199476469	0.177397360996924	0.593028942673836
Il6st	1796.53257652299	-0.196009119704741	0.145362117783694	-1.34841953800104	0.177523483386935	0.593276069443076
Galnt10	149.965295106762	-0.402067577533751	0.2983668414023	-1.34756119562102	0.177799558429504	0.593675023827775
Gtdc1	206.381335857894	-0.274111798995094	0.203380917857534	-1.3477754052969	0.177730630665753	0.593675023827775
Hpse	29.748628659038	-0.76787170077742	0.569798403539553	-1.34761995822987	0.17778064798829	0.593675023827775
RT1-CE14	454.594273712731	-0.229541771855763	0.170385673682392	-1.34718939036883	0.177919244084437	0.593900184512104
Blmh	1360.1022112188	0.201711562028911	0.149847580547321	1.34611157078516	0.178266537605244	0.594559739539442
Prpf40b	782.608697011663	-0.220265134555645	0.163614818616767	-1.34624196278681	0.17822449607207	0.594559739539442
Ublcp1	916.270628639408	-0.144518778880793	0.107361965953849	-1.34608916292495	0.178273763184094	0.594559739539442
Polr1c	764.76677924928	0.1765368622979	0.131169200195016	1.34587130237459	0.178344025277145	0.594619592637145
Ccdc173	51.8953057342842	-0.403013768725219	0.299537140507334	-1.34545508461029	0.178478316739923	0.594692384929495
Guca2a	153.982212814406	0.596703829292953	0.443594809494388	1.34515512021676	0.17857514602889	0.594692384929495
Mettl13	398.301010922291	0.200885165049494	0.149336186159733	1.34518746069103	0.178564704560047	0.594692384929495
Ppp1r3b	56.4814694118599	0.517338800482235	0.384477958752198	1.34556166018263	0.178443923274402	0.594692384929495
Axin1	1022.02156495579	0.14055988059769	0.104537399803824	1.34458940878065	0.178757865507363	0.594747085840825
Ccdc71	161.24997058455	0.327150714584625	0.24333309680206	1.34445629831747	0.178800879141131	0.594747085840825
Hnrnpu	6574.12391255149	-0.151962145757669	0.112995904209188	-1.34484649528839	0.178674811748226	0.594747085840825
Mpdu1	1845.40508280323	0.171976191207508	0.127914612025904	1.34446087498339	0.178799400098558	0.594747085840825
Chid1	175.169179810157	0.262265676267548	0.195134042821923	1.34402830215991	0.178939235018957	0.594905146684307
LOC100360582	151.200816460846	-0.382475063803841	0.284582732968182	-1.34398548996507	0.178953079052758	0.594905146684307
Gga3	529.449185666271	-0.181452678161021	0.135078661759372	-1.34331119214269	0.179171229536422	0.595433827550114
Matr3	3279.71503336105	-0.123351730066222	0.0918362653768554	-1.34317014700065	0.17921688580406	0.595433827550114
Smarca5	1328.84376704925	-0.198707004527785	0.147986400693696	-1.3427382759249	0.179356736130591	0.595724331636557
Klhl15	29.7401612699861	-0.513200474441211	0.382300835956211	-1.34239956121883	0.179466476925882	0.595914687916908
Samm50	4751.64487753258	0.139733156002613	0.104110525400021	1.34216166392131	0.179543583541713	0.595996603544134
Zbtb2	138.000418347877	-0.3112633929327	0.231978948362957	-1.34177430809667	0.179669184932704	0.596239403195652
Sec13	1513.91017759101	0.187724456494795	0.140051057491601	1.34040013590081	0.180115291574724	0.597022573356171
Snx4	622.475955161808	-0.152540258144633	0.113800838727825	-1.34041418191531	0.18011072756455	0.597022573356171
Zfp423	20.4783004328979	0.80169049327289	0.598020426322773	1.34057376301088	0.180058880473377	0.597022573356171
Zmpste24	843.550232496409	0.308099610992285	0.229806456423508	1.34069170982947	0.180020567276078	0.597022573356171
Fto	233.159474103199	0.29676990883822	0.221462004214424	1.34004887154765	0.180229456814902	0.59722682395282
Bfar	577.068939542175	0.140222119910548	0.104722266135723	1.33899050397568	0.180573764317533	0.597933019791314
Pde10a	12.6159423127413	1.0611062903919	0.792515784223523	1.33890871515138	0.180600392129472	0.597933019791314
RGD1563667	18.1097145626715	-0.615727984134997	0.459777167500313	-1.33918782327219	0.180509535500997	0.597933019791314
Angpt1	25.0824648524902	-0.659447161887982	0.492679602128624	-1.33849089558171	0.180736466252088	0.597944698161168
Prkar1b	190.580716223954	-0.309697590802822	0.231414031717567	-1.33828354531585	0.180804023674706	0.597944698161168
Smarca2	2940.6061209088	-0.202519645013559	0.151331488980462	-1.3382518494859	0.180814352241768	0.597944698161168
Tex101	38.3462421672674	-3.44961096771621	2.57689705464637	-1.33866852053568	0.180678608632473	0.597944698161168
Cish	288.272235340868	0.460923726113944	0.344528981960081	1.33783730904632	0.18094947687168	0.598217496836391
En2	167.039633881623	-0.275815975853359	0.206212008708809	-1.33753595428498	0.181047754269287	0.598368355633822
Wdr70	270.381891928748	0.277629432465677	0.207708359928392	1.33663099820051	0.181343115459229	0.599170305322558
Lamtor1	2525.60760907482	0.158201836215138	0.118394316374	1.33622830098861	0.181474663418292	0.59925096581914
Mfsd7	523.389940445877	-0.323776922167816	0.242298274522774	-1.33627415550325	0.18145968068185	0.59925096581914
Prss8	5857.55382650144	0.170508613169915	0.127619317028321	1.33607213343789	0.181525697282712	0.59925096581914
Pusl1	78.9759585218001	0.335640549810705	0.251265587966424	1.33579991007585	0.181614682391251	0.599370638809222
Arfgap1	747.977119710888	0.141672841239104	0.106190200503974	1.33414232732145	0.18215721622025	0.599382241489133
Arl6ip1	2430.86826901456	0.191451650895125	0.14347595944623	1.3343813948627	0.182078894308795	0.599382241489133
Calu	800.004886920224	-0.189952328144165	0.142320086644132	-1.33468389897159	0.181979825483066	0.599382241489133
Cers4	80.8545356825257	-0.353810416608938	0.265133021360232	-1.33446379026557	0.182051906197011	0.599382241489133
Ift27	239.68704551835	0.218630563627932	0.163888886538913	1.33401701753598	0.182198279460228	0.599382241489133
Ikbip	77.9980298635197	-0.435020752507928	0.325708152437764	-1.33561517957722	0.181675086028629	0.599382241489133
Rhoc	1192.10622635563	0.27425528173222	0.205558052884005	1.33419867470228	0.182138753770177	0.599382241489133
Sncaip	55.2898037246471	-0.75253934229556	0.563859757881035	-1.33462147595632	0.182000265483596	0.599382241489133
Snx29	107.808233226073	0.451795221255396	0.338647203612958	1.33411767891565	0.182165292807712	0.599382241489133
Taf5l	366.760870894447	-0.19843845669291	0.148595330453886	-1.33542861735141	0.181736103736299	0.599382241489133
Wdr41	239.96566725874	-0.207709012888795	0.155595330967285	-1.33493088512063	0.181898968211519	0.599382241489133
Fhl3	32.4169418229876	-0.77437318326718	0.580744628140899	-1.33341428528772	0.182395887012604	0.599858695539715
Plekhh3	508.184561155915	0.15797958010179	0.118523491574138	1.33289677855105	0.182565679896229	0.600243424269754
Chst12	47.2044932275025	-0.70359906728015	0.528013389931716	-1.33254019821569	0.182682741336315	0.600440735062671
Crem	269.86325254542	-0.304397784164666	0.228487178929205	-1.33223135578641	0.182784175903294	0.600440735062671
Fitm1	18.7987968099333	0.934409196642163	0.701376867800908	1.33224980682911	0.182778114768467	0.600440735062671
Nr2f6	2335.75101680184	0.16959959578824	0.127437100467162	1.33084945566494	0.183238551205566	0.601411889402963
RGD1303130	1461.87248506483	0.128658091811291	0.0966591913519863	1.33104870847492	0.183172984348576	0.601411889402963

Tmem125	253.195653414254	-0.349547513189072	0.262648294373428	-1.33085773133593	0.183235827636959	0.601411889402963
Tmc3	473.409214812002	0.166327520674779	0.125009988132816	1.3305138506058	0.183349025942451	0.601600758909324
Ptcd1	566.742594153669	0.174873646759178	0.131584042204434	1.32898825594282	0.183851844480662	0.603076497652872
Xpnpep1	4844.71024655466	0.154767446479122	0.116482396448529	1.3286767030717	0.183954654192912	0.603239642110974
Isy1	525.561409297935	-0.18537076260086	0.139552065250743	-1.32832690270683	0.18407013596924	0.6034442357734
Csnk1g1	19.1064185305093	0.646800735281259	0.487367076009847	1.32713260111192	0.184464822714283	0.604389499847373
Gstm5	34.7808380914307	0.762411911165126	0.574429920107016	1.32724965131184	0.184426112890708	0.604389499847373
Prpf3	585.198477224962	-0.198174456108896	0.149350691078315	-1.32690685712983	0.184539495783111	0.604459916158745
Fstl3	15.4043117609299	-0.838436183953758	0.63234913798452	-1.32590705607048	0.184870485494102	0.605257792915708
Lin54	292.214312986137	0.29191777992285	0.220190458575331	1.32575126920398	0.184922099135027	0.605257792915708
Safb	1924.80586087714	-0.17303809796835	0.130526948747081	-1.32568867677771	0.184942839591435	0.605257792915708
Bex2	147.543981275117	0.279371337602671	0.210803905517483	1.32526642197102	0.185082801768905	0.605541486731541
Cep76	61.2281327437583	0.47848421610364	0.361176532470747	1.32479320522408	0.185239749083506	0.605773216360707
Dcun1d4	1004.50551739919	0.141705377044328	0.10696913123523	1.3247314941047	0.18526022347966	0.605773216360707
Abcg4	19.8176388061226	0.89200405277445	0.67394608597145	1.32355402211244	0.185651203597349	0.606326189361709
Ets1	560.564111676101	-0.758345764435699	0.572966122995908	-1.32354380826302	0.185654597777919	0.606326189361709
Omd	22.4789202533845	-0.563424164933909	0.425733761447884	-1.323419037799	0.18569606415345	0.606326189361709
Prickle2	119.215334108977	-0.343738285392273	0.259659801768577	-1.32380246403574	0.18556865759742	0.606326189361709
Zswim8	1123.47630339328	-0.120632650021536	0.0911098863095988	-1.32403468940369	0.18549152416861	0.606326189361709
Tbk1	562.281396910174	-0.175455712527747	0.132638360273724	-1.32281273807714	0.185897660071198	0.606810110387489
Col3a1	953.615914073727	-1.01861976121352	0.770139967605745	-1.32264238198189	0.185954332933289	0.606820829204641
Katnal1	95.959219089554	0.431909671497922	0.3266315186912	1.32231473933859	0.186063366675001	0.60682818525913
Sgms2	453.351982782051	0.361734481473454	0.273542608908225	1.32240634436158	0.186032877367373	0.60682818525913
Cyp2j4	600.732732233804	-0.353921325017144	0.267777719749702	-1.32169818067001	0.186268674738742	0.60715024877173
Tra2b	2376.18934828663	-0.206721601544856	0.156406156199953	-1.32169734598284	0.186268952794917	0.60715024877173
Abca1	70.394605098193	-0.557821249601377	0.422372936638986	-1.32068416608369	0.186606695794935	0.607317028504825
Cebpd	291.195829913701	0.319138026648395	0.241544378017849	1.32123972111167	0.186421445826614	0.607317028504825
Cnot11	337.314160924499	0.257764266161732	0.195120657591477	1.32105062243799	0.18648448556703	0.607317028504825
Ns5atp4	68.3596171742056	0.364518789707757	0.276095812272679	1.32026192902828	0.186747581789932	0.607317028504825
Pigs	1041.82476134173	0.127843093628141	0.0968255067390234	1.32034520586317	0.186719788953356	0.607317028504825
Rrm1	906.452947157998	0.147256705000944	0.111503673673065	1.3206444249784	0.186619952687441	0.607317028504825
Tm9sf1	1003.68702000234	0.190935839383938	0.144586523533421	1.32056456381845	0.186646594986916	0.607317028504825
Zfp438	62.0706634458579	0.358685944368527	0.271617618858435	1.32055477798541	0.186649859809611	0.607317028504825
Palb2	50.2588821898691	-0.428564147412155	0.3246827310615	-1.31994746382424	0.186852559062677	0.607484607067045
Dapk3	1008.83478841737	0.177002650685265	0.134172980318599	1.31921233518824	0.187098135587154	0.60758783116674
Eaf2	315.590030775592	-0.417874456379201	0.316695990727973	-1.31948135945344	0.187008237903208	0.60758783116674
Slc45a3	1056.21420129953	-0.216232598335456	0.16388055580617	-1.31945243455975	0.187017901972381	0.60758783116674
Vps37c	223.361801173661	-0.234548889072484	0.177783034100683	-1.31929849357646	0.187069341255526	0.60758783116674
Mecp2	109.180078112045	0.291142332944907	0.220723355306006	1.31903727424438	0.187156651438869	0.607604255999481
Ahdc1	619.500095058697	-0.227075107433678	0.172412546523768	-1.31704514556529	0.187823492339552	0.607735019624387
Bsg	9800.03095353369	0.147355905959553	0.111848875942883	1.31745540326039	0.187686020375749	0.607735019624387
Chit1	68.8135763224504	-0.35565799600171	0.269913758459205	-1.31767271898985	0.187613230826418	0.607735019624387
Cinp	286.499927327545	-0.176847253759668	0.134121908852469	-1.31855604556148	0.187317576521071	0.607735019624387
Emp1	376.976039289045	-0.449639609404486	0.341162422832458	-1.3179634664082	0.187515878045333	0.607735019624387
Extl2	31.2810688989806	-0.53727421374733	0.407794656541598	-1.31751165722429	0.187667176202983	0.607735019624387
Lipg	20.2548810047958	0.788134734866196	0.597794367969462	1.31840441646057	0.187368303150172	0.607735019624387
Plekhh2	117.153942688997	-0.454941640780359	0.345381083025157	-1.31721644044767	0.187766084641025	0.607735019624387
Prpsap2	903.170396541432	-0.119090160343003	0.0903986812331775	-1.3173882485721	0.187708517944606	0.607735019624387
RGD1561507	92.1142572961873	0.330988715497698	0.251243985029362	1.31739956066617	0.187704728127647	0.607735019624387
Urb1	307.905327599312	-0.189223979515018	0.143678021080874	-1.31700017923066	0.187838564485348	0.607735019624387
Yipf6	350.97995246855	0.265798691541	0.201580806973162	1.31857142320295	0.187312432587421	0.607735019624387
Eci3	16244.3093864147	0.311697344725576	0.236843729964324	1.31604642762773	0.188158460226617	0.608007923102878
Otud7b	552.945828007838	-0.16682819218533	0.126731859184707	-1.31638715993414	0.188044129830231	0.608007923102878
RT1-T24-1	43.4507827449932	-0.65221445874834	0.495622024624101	-1.31595132246797	0.188190381266578	0.608007923102878
Skap1	165.254104324564	-0.563523367342457	0.428133669602558	-1.31623230629253	0.188096083555023	0.608007923102878
Whsc1	458.952841922388	0.178077078380939	0.135292541756468	1.31623721506752	0.188094436488084	0.608007923102878
Arhgap29	454.228267573053	-0.410827585314762	0.312541422412008	-1.31447403721478	0.188686729573074	0.608573600546982
Casp6	246.219531584457	0.207106071256955	0.157542588659974	1.31460370823254	0.18864312321207	0.608573600546982
Cst6	14.9406773951033	-0.902282321127271	0.686179722515529	-1.31493585648307	0.188531460785951	0.608573600546982
Lman2l	80.1999539500691	0.411607243570579	0.313119743217747	1.31453621972455	0.188665817627634	0.608573600546982
Mrpl49	328.567072764138	0.238483659345579	0.181364206812685	1.31494335920366	0.188528939065819	0.608573600546982
Slc7a9	1355.45335088623	0.263219995089765	0.200237963645548	1.31453591665417	0.188665919545897	0.608573600546982
Hs6st1	1620.63357133675	0.136325438889849	0.10380354372927	1.3133023593626	0.18908108383699	0.609286689718271
Klra17	15.3365729470681	-0.780673679805473	0.594490915651353	-1.31318016684936	0.189122245409648	0.609286689718271
Mrpl3	1219.61127562646	0.123570818242516	0.0940827722580154	1.31342662717923	0.189039229955102	0.609286689718271
Zfp496	513.796638847718	0.142001925322731	0.108097655433176	1.31364482193153	0.188965757643516	0.609286689718271
Flnc	18.7225423009693	-1.41701880318913	1.07928755050539	-1.3129205488617	0.189209721832209	0.609395777371745
RGD735029	2732.14521241134	0.151289952230355	0.115299379792481	1.31214888148272	0.189469905703138	0.610014676987783
Tmem39b	338.133332684738	-0.260634075744708	0.198649124393444	-1.31203234114688	0.189509222632752	0.610014676987783
Xpnpep2	5633.11322238417	0.141290316385311	0.10773421419006	1.31147117419962	0.189698626342969	0.610451468577064
Park2	35.5636795484909	0.466287816633691	0.355633932772952	1.31114546072122	0.189808624321994	0.610632557046074
Bbx	59.0890064276145	0.447554562311499	0.341468320205219	1.31067667431791	0.18996702249973	0.610969207054876
Phb2	4632.15661515278	0.175648359234108	0.134034768744177	1.31046862601267	0.19003735110384	0.610977660038607
RGD1566099	233.304038404884	-0.238684599085226	0.182153193978589	-1.31035088582242	0.190077160469515	0.610977660038607
Paxip1	336.787733363136	-0.183747362625951	0.140268392162793	-1.30996983563266	0.190206040202156	0.611124157177161
Sqstm1	4266.0645640894	0.288342276845019	0.220125712010062	1.30989821321663	0.190230271695653	0.611124157177161
Elovl2	716.351539022255	0.198197306755531	0.151382096202277	1.30925196392247	0.190449015354592	0.611308531070403
Grap	223.382559069167	-0.514372282355673	0.39287016316483	-1.3092678716349	0.190443628660093	0.611308531070403
Tinagl1	1126.15697778708	-0.755358067508895	0.576825916747446	-1.30950785250451	0.190362379574036	0.611308531070403
Foxo4	873.268583876815	0.134624192915144	0.102863188069268	1.30876939984096	0.190612475286419	0.61166047264411
Slc30a9	865.458640381244	0.173809005673551	0.132848619987904	1.30832375744194	0.190763520378229	0.611972388546132
Gmppa	704.259323726237	0.154566815519049	0.118178073062494	1.30791450151089	0.190902310313435	0.612244824780615
Fam151b	136.522682596975	-0.295755545926113	0.226168061669204	-1.30768042022966	0.190981727115625	0.612326744822622
Cytip	508.678694626887	-0.271488471083136	0.207797202061416	-1.30650686529887	0.191380245892536	0.612702683177298
Il4ra	4001.95955110353	0.162092645022147	0.124092514529923	1.30622419600548	0.191476326820848	0.612702683177298
Incenp	311.831850754964	-0.277221949089393	0.212219565862138	-1.30629778627236	0.191451309647261	0.612702683177298
Ptpla	74.576745798175	0.467631362399739	0.357773457713034	1.30705996299709	0.191192347403321	0.612702683177298
Ska1	15.5973028258008	0.670195030227916	0.513047275074627	1.30630267967105	0.191449646210852	0.612702683177298
Ssh3	528.016930025317	-0.185157625107716	0.141680737742814	-1.30686519605667	0.191258498186026	0.612702683177298
Zdhhc9	1381.52302103339	0.133420207315997	0.102110236288004	1.30662911149949	0.191338704691004	0.612702683177298
Capn5	182.713598558684	-0.511360694371018	0.391667082088589	-1.30560038807488	0.191688488308522	0.612863954462633
Cdk12	189.089135549845	0.218814276429487	0.167578652846189	1.30574075344981	0.191640733981928	0.612863954462633
Lrrc33	125.69142370669	-0.554646173080838	0.42478531347888	-1.30570939126504	0.191651403090596	0.612863954462633
Gng10	600.650807948899	-0.181190128615035	0.138827078561529	-1.30514976251359	0.191841856784136	0.613009430477506
Thy1	179.78080387967	-1.02905254674631	0.788430805763886	-1.30519068918077	0.191827923843756	0.613009430477506
Agpat3	3073.14474586776	0.138671341157903	0.106321427558855	1.30426522989584	0.19214316606093	0.613731775097743
Pdxp	144.859406550507	0.329424022987297	0.252593015461933	1.30416916867182	0.192175909517233	0.613731775097743
Atrip	241.093837169828	0.228282708707258	0.175150174456352	1.30335416116955	0.192453878280687	0.614446848465812
Slc25a44	832.191209228067	0.155372474238009	0.119230608145149	1.30312573805597	0.19253183791128	0.61452313105858
Dctn3	1190.94627577423	0.165490324856167	0.127041793927095	1.30264474186453	0.192696075326031	0.614610029834263
Nub1	1064.15867896233	0.176251885714462	0.135310777572075	1.30257093246381	0.192721286849315	0.614610029834263
Rps21	4690.69629884521	-0.210645585874616	0.161686307035755	-1.30280411332566	0.192641646119641	0.614610029834263
Acta1	9.62824906737632	1.2831056825843	0.985321543128789	1.30222026660448	0.192841099023861	0.614819616130493
Prima1	86.0035355113952	1.27633261933418	0.980339105016229	1.30192972289221	0.192940410689342	0.614963743100129
Cnr2	72.0485559538	-0.368110043233418	0.282805814614582	-1.30163534202821	0.19304107226492	0.615112090654074

Chkb	1285.53019785115	-0.169396890504526	0.130199805545931	-1.30105332949033	0.193240201115849	0.615574026312428
Spetex-2A	48.5094105047819	0.547611848613822	0.420964459943635	1.3008505484932	0.193309615717204	0.615622609201945
Neurl4	1954.13417673932	0.13789831010452	0.106037231873941	1.30047067117383	0.193439702006926	0.615837796601867
Sap30bp	682.983001437336	0.154621651985854	0.118908923163738	1.30033682815326	0.193485550911954	0.615837796601867
Asb1	44.0947065604168	0.438317301139522	0.337141223419466	1.30009998983178	0.193566701165137	0.61592360734685
Dnajc15	676.657502854895	0.255554070117215	0.196653333484041	1.29951557692743	0.193767051254137	0.616216089690687
Stc2	30.0883040269901	1.06092438154934	0.816312787869773	1.29965424689462	0.193719498250762	0.616216089690687
Sdhb	6545.57390765847	0.175195137440496	0.134866214917017	1.29902909745257	0.193933943615586	0.616229578734905
St5	404.77248059198	-0.34169062813648	0.263020795349431	-1.2991011896323	0.193909204908683	0.616229578734905
Tmem69	678.980643408827	0.19134318302218	0.147268729982565	1.29927910049087	0.193848164031222	0.616229578734905
Epha7	47.3915539799168	-0.763235517599046	0.587849105362437	-1.29835277563019	0.194166138866729	0.616622613679588
Mki67ip	750.623112976753	0.152253970684671	0.117255415377467	1.29848135537738	0.194121979082212	0.616622613679588
Bnip2	151.614921889077	-0.303276960443524	0.233680230773237	-1.29782891535153	0.194346131106687	0.61702182294933
Ccp110	147.90513573006	-0.342350348861758	0.263924300604425	-1.29715357046595	0.194578352314563	0.617586582632596
Armcx2	51.3707844626486	-0.545089284466655	0.420526455469152	-1.29620687920463	0.19490422015198	0.618258450507064
ST7	747.182188103757	0.153678721729408	0.118546862651717	1.2963541867903	0.194853488000654	0.618258450507064
Tada2b	950.468215058246	0.138543549219767	0.106895555877861	1.29606463133104	0.194953218952782	0.618258450507064
Mybph	21.9602305458901	-0.712286869383974	0.549719594772112	-1.29572763306583	0.195069337664009	0.618454139996966
Zfp563	81.2238567559523	-0.364040712371403	0.281091099156356	-1.29509868318138	0.195286189125949	0.6189689976591
Ift52	938.228689462771	0.118850752764599	0.0917865395012618	1.29486037288687	0.195368400712104	0.619056939641421
Lims2	376.617910815453	0.267098153579018	0.206303824272591	1.29468348209629	0.195429440384255	0.619077764606309
Prpf4b	1362.59111987538	-0.181725125597272	0.140390765650176	-1.29442363787724	0.195519130133411	0.619189309862455
Col4a4	193.426933941627	-0.261582072160352	0.20218257716841	-1.29379136335009	0.195737496716703	0.6195752680791
Zfp192	159.059873259984	-0.413967576769924	0.319973676519611	-1.29375510283438	0.195750025309876	0.6195752680791
Osgin1	19888.1928895741	0.189735965525775	0.146779718815408	1.2926579166185	0.196129398859473	0.620603214765248
LOC680227	284.594651083109	-0.252570255145405	0.195500407039578	-1.29191677383196	0.196385968125848	0.621242113475755
Golga5	589.945298208626	0.18317684681965	0.141805169035926	1.29175013904637	0.196443687697546	0.621251795873763
Rangap1	1300.69407378128	0.19997444743123	0.154952175446992	1.2905559205888	0.19685770917641	0.622387961752176
Tnik	20.9211943836573	-1.05954335639669	0.821171183842062	-1.29028316780351	0.19695235892164	0.622514046580467
Fbxo8	548.037052566867	0.21353359727479	0.165593555205001	1.28950427455006	0.197222831241746	0.623022429097743
Neu1	2598.96112748185	0.2685912655967	0.208276541333226	1.28958961905833	0.197193181924825	0.623022429097743
Baiap2l2	2498.97163016679	-0.163796439596962	0.127143872855015	-1.28827631185769	0.197649795934213	0.623206187361726
Bmp5	49.9444925350677	-0.556704577076483	0.43195995960218	-1.28878745518263	0.19747198843903	0.623206187361726
Camk1d	17.8266016585612	-0.846548685728916	0.65714308994835	-1.28822580451307	0.197667371893466	0.623206187361726
Capn13	21.4613605475789	-1.07671461699138	0.836618673513282	-1.28698372517774	0.19809996077511	0.623206187361726
Ftsjd1	180.86194544006	0.327963219832232	0.254747796913245	1.28740355679668	0.197953665261613	0.623206187361726
Lsm5	304.310091093534	-0.199524370935089	0.154905640746185	-1.28803812420242	0.197732692444869	0.623206187361726
Poglut1	318.996088409918	-0.274467195036928	0.213264461667895	-1.28698046027163	0.198101098781784	0.623206187361726
Rbp7	40.0506423352196	1.90230092287895	1.47663370672552	1.28826865742985	0.197652459511152	0.623206187361726
Senp7	281.866349970859	-0.178607371950027	0.138716193068002	-1.28757406038723	0.197894273751577	0.623206187361726
Smc5	669.730034242685	-0.183226141003248	0.14235383062361	-1.28711774176071	0.198053252450853	0.623206187361726
Tal1	57.8809895706495	-0.595731189434801	0.462893063960689	-1.28697367883955	0.198103462514334	0.623206187361726
Timm23	3156.32113680455	0.178955678426915	0.139038013535019	1.28709892983218	0.198059808419499	0.623206187361726
Ttc5	505.544171312179	-0.155399879047316	0.120624361804237	-1.28829596876555	0.197642955871642	0.623206187361726
Zufsp	285.470774034195	-0.226804683957579	0.176161944015451	-1.28747832129785	0.197927620940348	0.623206187361726
Zzz3	587.797763014843	-0.160476494480835	0.124537274447886	-1.2885820345136	0.1975434324717	0.623206187361726
Gps1	1116.16540730562	0.13060983091979	0.101564057230553	1.28598477139706	0.198448376481707	0.624118496704781
Slc1a4	238.489049077269	0.334974399488991	0.260542205520834	1.28568190638966	0.19855409839087	0.624278252368085
Plekhf1	339.02557046404	-0.306859095101626	0.238707552149927	-1.2855022488308	0.198616831398172	0.624302794710073
Ddx56	398.928524714785	0.193800138374119	0.150780752191211	1.28531086068833	0.198683676442418	0.624340245077282
Rps7	7288.13834591572	-0.179111698487663	0.1393974511793	-1.28489937923815	0.198827447909944	0.624543593179253
Snx6	1353.15358035973	0.173081024897527	0.134713202596221	1.2848111511112	0.198858284683769	0.624543593179253
Fech	1058.46217782927	0.186604685630617	0.145290092538228	1.28435932809058	0.199016256966179	0.624694497839711
Prdx3	4489.96313648698	0.163862309842207	0.127576030137684	1.28442866316942	0.198992009172138	0.624694497839711
Fbxw17	175.519472963671	0.265885428037166	0.207075213015686	1.28400412664081	0.19914051152922	0.624911942032334
Cldn10	4164.87620713146	-0.182932489255848	0.142609053198988	-1.28275509269804	0.199577891696042	0.625593578879541
LOC652955	770.785161956799	-0.156875732265118	0.122276939880378	-1.28295435278792	0.199508068834578	0.625593578879541
Mrpl13	864.274336236763	-0.177948480068742	0.13870742028118	-1.28290526713001	0.199525267316013	0.625593578879541
Polq	13.2378703740039	-0.983610482826377	0.766585599858624	-1.28310586972646	0.199454987628081	0.625593578879541
Pde4d	19.5816331896333	0.642908178723483	0.501330517898257	1.28240383493661	0.199701019629859	0.625634451243929
Pet100	921.81807404385	0.248552372018435	0.19381585580949	1.28241505825379	0.199697084613032	0.625634451243929
Osr2	16.3789914321796	0.734229311887838	0.573098672252577	1.28115688874646	0.200138565275968	0.626487175138158
Scp2	9598.94739013185	0.313842911574339	0.244925733400098	1.28137989919443	0.200060260944351	0.626487175138158
Sox4	267.687609433246	-0.351491394138969	0.274337838755095	-1.28123555880584	0.200110939771838	0.626487175138158
Atp7a	49.2444320861724	-0.592479659641813	0.462643173349027	-1.28064066168515	0.200319910862427	0.626882188012761
Cdadc1	588.616202248201	0.175814178639903	0.13736698392642	1.27988672106302	0.2005849784172	0.627538911282657
Spred2	104.578876890157	0.33540688094111	0.262116819626934	1.27960838765894	0.200682898477869	0.627672488745036
Tmx4	752.503591458901	0.176867969306589	0.138260252285908	1.27923945155867	0.200812747100021	0.627905827658552
Rps6ka4	782.221066709896	0.157784670304799	0.12344386123011	1.27818968665176	0.201182551686621	0.628889131592445
Ankrd17	1370.98413871822	-0.106863806532243	0.0836773703253516	-1.27709327045937	0.201569320424621	0.629405740644572
Calhm2	35.4169254716448	-0.58922015131596	0.461286871864877	-1.27733995319199	0.201482254068685	0.629405740644572
Hist1h2ail	79.6397536647721	0.39714125907088	0.310934361073107	1.27725111403015	0.201513606576936	0.629405740644572
Pcdhga2	42.5954510709613	-0.604801322167041	0.473538814033614	-1.27719482383151	0.201533473969431	0.629405740644572
Dab2ip	1272.32989781788	-0.201828399368553	0.158310766157392	-1.27488738932573	0.202349103232032	0.630224651576613
Echdc3	1912.14546437446	0.26279317957562	0.205952383880081	1.2759899867371	0.20195905877928	0.630224651576613
Gcgr	670.324740666831	-0.292368788551868	0.229176622113292	-1.27573565687401	0.202048979443873	0.630224651576613
Gstk1	1547.47927595526	0.213360751057367	0.167383419872203	1.27468270883859	0.202421569439674	0.630224651576613
Iqgap3	48.8390369641436	0.519702648059848	0.407636474790459	1.27491694242277	0.202338641652792	0.630224651576613
Serinc3	3287.14887761942	-0.19047125779959	0.149400846967367	-1.27490078982747	0.202344359504018	0.630224651576613
Smim5	66.5517953797226	-0.62820362946612	0.492832649930792	-1.27467940598972	0.202422738953512	0.630224651576613
Snrnp48	214.571913657005	-0.223225105788433	0.175129759455	-1.27462691939454	0.20244132472124	0.630224651576613
Stk17b	102.154439209592	-0.414526854671705	0.325214396888021	-1.27462639611996	0.202441510021662	0.630224651576613
Trim28	2015.57043833102	-0.106081763420888	0.0831900247226086	-1.27517408216442	0.202247632707752	0.630224651576613
Yeats2	1503.35601010701	-0.164936112117898	0.129330171593571	-1.27531039420732	0.202199400164933	0.630224651576613
Tiam1	100.717936397539	-0.427745814297142	0.335698166036454	-1.27419765007203	0.202593377825466	0.630524735039497
Tmem183a	3460.69968220778	0.181617619515533	0.142625693669501	1.27338640635387	0.20288095870001	0.63124691392946
Grwd1	435.942992497136	0.155797530848036	0.1223870544706	1.27299028089169	0.203021490719145	0.631453309206335
Lrrc28	212.616127184367	0.221503891103454	0.174038434979302	1.27272973426702	0.203113962560123	0.631453309206335
Pcgf2	325.876594974474	-0.219110120823706	0.172154477400796	-1.2727529607818	0.203105717882273	0.631453309206335
Adamts15	84.7837819025156	-0.419039686252667	0.329401068840335	-1.27212606725262	0.203328330743697	0.631815591069937
Eif6	2993.43032791295	0.168440283433062	0.13241239213813	1.27208851613638	0.203341670960217	0.631815591069937
Trappc6a	1335.39731127771	0.276947902679784	0.217808399359178	1.2715207654737	0.203543444803331	0.632269689432811
Abcb11	100.192859669763	-0.566523687508625	0.445652842523299	-1.27122197695622	0.203649690264835	0.632426879658502
Eif4g2	26315.3897906293	0.20520945595381	0.161447184338486	1.27106246414042	0.203706427553578	0.632430280134927
Ap5m1	368.138727418182	0.196881354937149	0.15502518585008	1.26999592909726	0.204086080240199	0.633435933372502
Senp3	916.172314268345	0.11528615057238	0.0907988279564576	1.26968765089859	0.204195913366655	0.633603808715642
Alas1	2304.09083478572	0.256961055014533	0.202433190144686	1.2693622761706	0.204311884273996	0.63379063227572
Tmem104	110.572963946712	0.433887291749846	0.341925292167466	1.26895348688432	0.204457653921408	0.633943247761456
Upf2	425.490307104704	-0.208345251731642	0.164200778694546	-1.2688444804468	0.204496537159975	0.633943247761456
Uqcrc1	7459.14178891534	0.153752982691531	0.121184125670055	1.26875514298094	0.20452840837069	0.633943247761456
Lsg1	455.437213629153	0.160006790775584	0.126129821029476	1.26858810604505	0.204588008620386	0.633955099776255
Fah	9837.72343901881	0.231455718258313	0.182530560936246	1.26803816890233	0.20478432028726	0.634217597925066
Prkrir	871.534952742626	0.209554678889282	0.165255350235367	1.26806592700824	0.204774408164277	0.634217597925066
Lztfl1	545.294016747447	0.287219359471645	0.226629882265701	1.26734990372942	0.205030204012828	0.634806128795914
Apex1	815.304244067473	0.191220539469286	0.151050371584876	1.2659388882194	0.20553496227635	0.6350404056994

Ensa	443.258612640496	0.179800344454269	0.142051144102365	1.26574372625046	0.205604847998306	0.6350404056994
Irf2bp1	607.799642291051	0.155235500499206	0.122659869153489	1.26557692887275	0.205664590302612	0.6350404056994
Paqr5	3939.32357390744	-0.122471823174442	0.0966677248861993	-1.26693602563441	0.205178165864145	0.6350404056994
Prrc1	178.397179985165	0.252382558541331	0.199288692505502	1.2664168516955	0.205363880823824	0.6350404056994
Raf1	1390.32134094971	0.113627970462918	0.0897433069092558	1.26614423265919	0.205461448943042	0.6350404056994
Reep3	668.489096493	0.303965093325672	0.239994463241082	1.26655044129218	0.205316082497571	0.6350404056994
Rnasek	2282.15389078382	0.12151515804224	0.0959456483506868	1.26649994169715	0.205334150290086	0.6350404056994
Trim65	362.503528993774	-0.182557580861172	0.14419178097786	-1.26607480414714	0.205486302219935	0.6350404056994
Zmynd10	279.249028945823	-0.223519403029663	0.176613511376488	-1.2655849560297	0.205661714903469	0.6350404056994
L3mbtl2	920.965795566178	-0.125768424139888	0.0994091678514744	-1.26515920873411	0.205814261693041	0.635329956111651
Ccdc80	431.227405823374	0.3725202180456	0.294996455840725	1.26279557150589	0.206662656647121	0.636056327920313
Ier5l	221.920996552597	0.454890588640689	0.360227015987143	1.26278865396626	0.206665143323044	0.636056327920313
Jam3	166.276257874791	-0.478526287451514	0.378899466601418	-1.26293734785037	0.20661169651635	0.636056327920313
Mettl20	243.513843552438	-0.244381943236685	0.193297891220429	-1.2642763027249	0.206130872067767	0.636056327920313
Npc2	1786.2361264801	0.164603917886915	0.130309646063215	1.26317523575395	0.206526210516338	0.636056327920313
Nr1h3	1525.25937004782	0.177392921332909	0.140404228437932	1.263444294424	0.206429554125404	0.636056327920313
Pias2	526.971396593928	0.180083266841202	0.142518058172569	1.26358209724656	0.206380062687092	0.636056327920313
Sh3glb2	517.273845546083	0.19511009132564	0.154506819475388	1.26279274913635	0.206663671213079	0.636056327920313
Slc12a7	1638.33636128529	-0.216854966545788	0.171677875997164	-1.26315033481292	0.206535157568684	0.636056327920313
Wnt9b	242.841924177084	-1.02587387546159	0.81168864345429	-1.26387609797742	0.206274502077477	0.636056327920313
Zfp574	534.044326247244	-0.217306690407834	0.171925833682326	-1.26395600796888	0.206245817269395	0.636056327920313
Casc1	192.72481911014	-0.292644458356916	0.231798147859763	-1.26249696582548	0.206770017407754	0.636206826707237
LOC681383	24.6608588214933	0.572321794230658	0.453593765749425	1.26174969200706	0.207038869850166	0.636689338397452
Rhpn2	419.297616692955	-0.283306139185982	0.224531325071671	-1.26176665592452	0.207032763798299	0.636689338397452
Elmod3	189.805339352789	0.206838791221209	0.163975420579258	1.26140119348701	0.207164338551416	0.636902859609249
Cnnm4	455.136240242696	-0.187684261025384	0.148815490761597	-1.26118766309117	0.207241242407766	0.636960200664085
Napg	1067.32245803877	-0.151972697252884	0.120513948663926	-1.26103823613551	0.207295071463703	0.636960200664085
Exd2	531.812007532205	0.208027708611124	0.165074689664481	1.26020354200845	0.207595945440197	0.637539993481025
Phkb	342.880162701198	0.223184779421905	0.177085023966272	1.26032554545331	0.207551948302978	0.637539993481025
Gcc2	749.141937934905	0.198509757265429	0.157546638022025	1.26000630516582	0.207667087711448	0.637586201763457
Mgst1	4866.89306712566	0.278432692034933	0.221008100486618	1.25983025699908	0.207730602276018	0.63760897258148
Siah2	239.521056028317	0.360420854785549	0.286172218436663	1.25945438293941	0.207866257073251	0.637853098783632
Apc	553.951791381472	-0.168882306181817	0.134160479029497	-1.25880816320495	0.208099631108582	0.638224610680016
Lars	1051.7971219883	0.112260252308016	0.0891730285129915	1.25890366380974	0.208065130322028	0.638224610680016
LOC689574	1955.25307462812	0.175025570863188	0.139081335077375	1.25844039939518	0.208232529122408	0.638287736247383
Slx1b	453.577992908216	-0.200545160127708	0.159348496900369	-1.25853185959512	0.208199472600457	0.638287736247383
Adck2	320.53026118011	0.217083704741926	0.172573178606523	1.25792261865263	0.20841974271563	0.638345134924777
Chsy1	90.5462283510041	-0.388007671938872	0.308417427500246	-1.2580601397389	0.208370007425068	0.638345134924777
Slc10a1	72.6347534519875	0.631902176216593	0.502278084099607	1.25807236313994	0.208365587178262	0.638345134924777
Akap9	936.15954768689	-0.170142720711116	0.135439575104049	-1.25622603718601	0.209034029188462	0.638486321703252
Amigo3	43.6770168971756	-0.444090855699214	0.3536223983465	-1.2558335042569	0.209176341452835	0.638486321703252
Atp8b1	153.00595513028	-0.479653427938816	0.381730848833819	-1.25652257187007	0.208926567473652	0.638486321703252
Card6	87.2298607621439	-0.378779740282152	0.301583100861303	-1.25597136974976	0.209126350517153	0.638486321703252
Entpd8	217.580381601927	0.255584141186618	0.203336836025416	1.25694953350544	0.208771910427354	0.638486321703252
Mad2l1	72.6212751100132	0.34792616916093	0.276976810483694	1.25615631342326	0.209059302316581	0.638486321703252
Mcm2	305.83586261783	0.281275230366911	0.223857613173589	1.25649168853059	0.208937757472579	0.638486321703252
Mctp2	14.1170908522983	-0.87649985972333	0.698145227453834	-1.25546924229499	0.209308466889523	0.638486321703252
Pon3	144.803924947316	0.30712957230266	0.244325317194284	1.25705176945881	0.208734890115211	0.638486321703252
Ppif	560.30599473228	0.202444259857529	0.160998258848911	1.25743136171126	0.208597478882206	0.638486321703252
Ppp2r1b	4125.11579197209	-0.160846696701835	0.128038803969826	-1.25623398309578	0.209031149134501	0.638486321703252
Rab14	434.582166451753	0.196213772638453	0.156286054867004	1.25547844179333	0.209305129295091	0.638486321703252
Rrp1	962.681707334034	0.142808324047445	0.113731193798391	1.25566539203482	0.209237311764686	0.638486321703252
Smim11	105.84188027773	-0.282949190699599	0.225179434568797	-1.25654987650815	0.208916674511735	0.638486321703252
Zbtb24	190.208867054825	-0.218901503757735	0.174296511598941	-1.25591442851955	0.209146996733019	0.638486321703252
Adm2	638.830559904482	0.273103106309783	0.21761670073324	1.25497310357885	0.20948852400595	0.638521470595769
RGD1310587	3000.98779049357	0.133581712403861	0.10643604380444	1.25504206685187	0.209463489359688	0.638521470595769
Zfp951	51.0828239849678	-0.422483203597825	0.336621904365811	-1.25506747516557	0.209454266327634	0.638521470595769
Rmnd1	444.198425406932	0.147489475457966	0.117566175387532	1.25452303753013	0.209651957580978	0.638620890664389
Speg	32.6192877355509	0.666419655688777	0.531257567642534	1.25441912977546	0.209689702970218	0.638620890664389
Zfp444	621.852129983566	-0.260627048893277	0.207766534913826	-1.25442265763048	0.209688421365729	0.638620890664389
Kif20b	141.066755331629	0.398966881702299	0.318261554298964	1.25358176730176	0.209994061731483	0.639205277354053
Tmem9	526.287584670894	0.149311482034934	0.119103304170394	1.25363005732673	0.209976500920462	0.639205277354053
Smcr8	117.877113866808	0.396402260542042	0.316279092511169	1.25333058658642	0.21008542149127	0.639312155467141
Ddx50	747.813222656631	-0.145423610063549	0.116062276646726	-1.25297912694056	0.210213303112504	0.639530086503406
Thbd	259.801077165731	0.368213417277329	0.293998337192776	1.25243367290166	0.210411882814107	0.63996292749937
Sult2a2	148.995784709792	-0.382831109411427	0.305743524917663	-1.25213153578485	0.210521938198378	0.639999922538285
Zfp655	645.85962600162	-0.150697886935528	0.120356964816261	-1.25209112048967	0.210536662886737	0.639999922538285
Atp1b2	208.996964311704	-0.484884414477162	0.387562773800134	-1.25111194174499	0.210893639288586	0.640748835132247
Tmc4	713.602769963505	-0.217417739390559	0.173780418938124	-1.25110608386767	0.210895776194768	0.640748835132247
Tbc1d25	248.241884851524	-0.183852574565171	0.14697465384103	-1.25091347222379	0.21096604808537	0.640791048246478
Aif1l	10923.9184287182	0.185969465826137	0.148763570307636	1.25010085091102	0.211262709002491	0.64093546130353
Fam129b	1485.73615941179	0.281046688920825	0.224860650442822	1.24987047919392	0.211346864877394	0.64093546130353
Fbxo28	215.81593403967	0.215187818522723	0.17209109595923	1.25042970598376	0.211142618678213	0.64093546130353
Magt1	208.425454930466	0.323977911050078	0.259122772888916	1.2502872960107	0.211194617470308	0.64093546130353
Ncbp1	2621.54717399213	0.119904426255879	0.0959345467419011	1.24985659835832	0.211351936386207	0.64093546130353
RGD1305014	43.7097108397626	0.426601291933578	0.341220582037638	1.25022145319043	0.211218662084698	0.64093546130353
Bak1	318.240434699549	0.254693310157571	0.203922045644443	1.24897388780443	0.211674624669188	0.641400635561181
Pmf1	112.996935619509	0.280961201354652	0.224941999845896	1.24903842567032	0.211651019810267	0.641400635561181
Rsu1	417.902137001443	-0.233487108011413	0.186908526752581	-1.2492052239034	0.211590021822374	0.641400635561181
Rundc3a	53.0533898415005	0.376220869700169	0.301267352117823	1.24879402648659	0.211740419361846	0.641428999591349
Rcn1	489.329706344662	-0.20134565526086	0.16127091131387	-1.24849331860595	0.211850453694954	0.641591328722848
Abcc10	208.667504594491	-0.212218031150336	0.17019022478074	-1.24694606534389	0.212417274400039	0.642465441323851
Arse	437.085870391144	-0.236847401462331	0.189972239852247	-1.24674742818499	0.21249012240026	0.642465441323851
Aurkb	44.4602821361205	0.532311352581257	0.426800519600046	1.24721345953394	0.2123192390019	0.642465441323851
B4galt5	89.9225978209292	-0.406878779465441	0.326558005931074	-1.2459617344409	0.212778443811284	0.642465441323851
Brpf1	572.395360718089	-0.176781035902057	0.14191280956986	-1.24570175474563	0.21287390920514	0.642465441323851
Cep44	148.286366733499	-0.231428733362806	0.185740699322618	-1.24597750631288	0.212772653323121	0.642465441323851
Fbxo7	800.243872118733	0.135827226921122	0.108903994545357	1.24721987919875	0.212316885746037	0.642465441323851
Gsk3b	156.424068782553	0.244005232607222	0.195823711954434	1.24604538526978	0.212747733528325	0.642465441323851
Retn	44.1411305540078	2.23512364185632	1.79360842205712	1.24616031814393	0.212705544054944	0.642465441323851
Ror2	109.316462971156	-0.268437611208806	0.215378547008214	-1.24635259610405	0.21263497631464	0.642465441323851
Sf3b3	902.977885627047	-0.119291460357723	0.0956435161836768	-1.24725088660095	0.212305519631115	0.642465441323851
Ttc36	3779.06713175022	0.26000004173768	0.208698202617112	1.24581830833823	0.212831106521216	0.642465441323851
Ugt1a8	10.4885226826484	-1.32815714221634	1.06573304518248	-1.24623811583971	0.212676989564223	0.642465441323851
Lysmd1	211.646216867213	0.237453830283133	0.190652906042935	1.24547710922202	0.212956424679292	0.642543860606538
Zfp110	886.989284761869	-0.139208818287598	0.111828989408164	-1.24483659402036	0.213191821742571	0.643083398600335
Adck5	365.074838278169	-0.219398313666902	0.176387955663283	-1.2438395401879	0.213558624706332	0.643347173236257
Evl	395.40136132117	-0.203231796273812	0.163326800964554	-1.24432606941171	0.213379580164943	0.643347173236257
Hif1a	1869.96794854075	0.140885065083781	0.113267174354169	1.24382960806681	0.213562280892169	0.643347173236257
LOC100362172	37.9096976860336	-0.478503794301471	0.384643355142061	-1.24401939590181	0.213492424516706	0.643347173236257
Troap	40.7617882470779	0.575223248116703	0.462338974993053	1.24415911101881	0.213441009253219	0.643347173236257
Epb49	2492.6795875543	-0.148213456289812	0.119242478203018	-1.24295853728794	0.213883112849871	0.643675422661252
Olr1331	66.3974813642476	-1.37865345182878	1.10920640607683	-1.2429187609049	0.213897771545975	0.643675422661252
Phf1	1987.85026451478	-0.146187776128377	0.11761077080193	-1.24297949185771	0.21387539080343	0.643675422661252
Tnfsf12	433.157110466871	-0.333791708066688	0.268515042097205	-1.24310245511628	0.213830081208588	0.643675422661252

Angptl6	20.3954955105652	0.599789894126795	0.48308573835514	1.24158062742448	0.214391333076696	0.644102105765941
Camta1	42.4086457297542	0.410457682081624	0.330452854851814	1.24210663050766	0.214197222509933	0.644102105765941
Cog2	812.960729635808	0.155898980036058	0.125623812696521	1.24099863465118	0.214606253258457	0.644102105765941
Dsc2	60.7047875716745	0.702421356366296	0.56585039101847	1.24135525487932	0.214474540961554	0.644102105765941
Fundc1	527.731661999854	-0.156113581167547	0.125793848993109	-1.24102714414994	0.214595721561443	0.644102105765941
Grrp1	42.50896938835	-0.703571432595548	0.566832080417249	-1.24123432124315	0.214519199441334	0.644102105765941
Neto2	109.658768573879	-0.473425987415028	0.381361856259161	-1.24140886049522	0.214454747576817	0.644102105765941
Plekha3	485.865742186696	-0.208787739324592	0.168225751972936	-1.24111639791143	0.214562752729043	0.644102105765941
Rpa2	251.001327538266	-0.192997084558485	0.155430646201056	-1.24169260873325	0.214349998065344	0.644102105765941
Wdr7	92.7639730584378	0.312075826590582	0.251373800781892	1.24148111545387	0.214428070176556	0.644102105765941
Cope	920.546384291406	0.126063821561191	0.101597095950023	1.24082111188693	0.21467184034606	0.644128864142904
Wfdc2	1489.82072756954	0.259261312200758	0.208991112306766	1.24053750104073	0.214776652434931	0.644273273047092
B3gnt2	766.237362647034	0.167595530705257	0.135142508629563	1.24013926043545	0.214923889703881	0.644470818991625
Gimap8	68.8879625963784	-0.553984261323486	0.446742522065555	-1.24005267902883	0.214955910152846	0.644470818991625
Mif	7989.64876935546	0.163703736989267	0.132034339148424	1.23985728292427	0.215028186236007	0.644517501254868
Fbxo34	263.804630155753	-0.196165766273844	0.158292239285035	-1.2392633218146	0.21524799713549	0.644925918059403
RGD1306502	579.854362576651	0.142073938302784	0.114651345473337	1.23918247724205	0.215277928305242	0.644925918059403
Wdr54	428.405792776651	-0.176801703023165	0.142720741609384	-1.23879473319343	0.215421524868306	0.645186047866447
LOC100188936	694.136590139243	-0.181105974827376	0.146317932746082	-1.23775651711581	0.215806355999851	0.646094106098357
Pdk4	117.210166191784	-0.58907130620757	0.475951790918054	-1.23767011165421	0.215838405846864	0.646094106098357
Nsbp1	998.48160530027	0.177523380214814	0.143470012786061	1.23735529653526	0.215955207280629	0.646273535005695
Gda	32.1092405250479	0.561669717310282	0.454241899888802	1.23649913723894	0.216273086229462	0.646895592919238
Naa40	350.750735173326	-0.221728080944534	0.179320725390801	-1.23648886909928	0.216276900676852	0.646895592919238
Mtmr12	170.124942332648	0.263468165035832	0.213206101658697	1.23574401945398	0.216553729433308	0.647553193564584
Pfkp	699.492394536882	-0.329120567092448	0.266373221342642	-1.23556176342926	0.216621505019322	0.647585488177174
Rbm20	204.247778482833	-0.453679507812789	0.367241012827722	-1.23537266254523	0.216691842139275	0.647625421444912
Zfp410	665.401486477921	-0.146026013786402	0.118227952724935	-1.23512257821246	0.216784887627935	0.647733184221638
Mtfp1	450.67512899119	0.22045070575658	0.178559533493161	1.23460619236566	0.216977103270342	0.648137123724235
Cbr4	781.244638879256	0.193739892713922	0.156990688861624	1.23408524491978	0.217171141100671	0.648500306830469
Tac4	26.3740447992572	-0.739442394840125	0.599236876714268	-1.2339734478536	0.217212798531022	0.648500306830469
Alcam	289.899146566188	-0.328540303366746	0.266353741632295	-1.23347358048493	0.217399127655906	0.648886156758674
Coq5	533.011739198018	0.146722114295609	0.119039234419072	1.232552569845	0.217742741895894	0.648977634667242
Cryab	11200.4286550536	-0.304554801759053	0.247139322432472	-1.2323202910871	0.217829462982186	0.648977634667242
Ppt1	407.615256387155	-0.264791545112909	0.21482835631356	-1.23257259728983	0.217735265828082	0.648977634667242
RGD1305089	352.99137057656	0.151538066767556	0.122962006005311	1.23239748350406	0.217800640501552	0.648977634667242
Rpgrip1l	304.144981931904	-0.180156117154869	0.146175598571994	-1.23246368692747	0.217775923328964	0.648977634667242
Trpm7	1669.1354160361	-0.162103764521459	0.131449564399149	-1.23320123016328	0.217500696542723	0.648977634667242
Wnt10a	33.7697808902748	0.782410537020258	0.634833548876974	1.23246564143365	0.217775193641056	0.648977634667242
Emd	593.723088213793	-0.30681030435428	0.24901861603125	-1.2320777829549	0.217920029687986	0.64907732113041
Dcaf8	1963.63670545589	0.119737911507405	0.0972081990699104	1.2317676148006	0.218035904043861	0.64908226436944
Krt19	1224.49537500753	-0.558329758964548	0.453262528113584	-1.231802155118	0.218022998087887	0.64908226436944
Fut8	246.430941985805	0.25002473319379	0.203167983183191	1.23063058104165	0.218461062207886	0.649447512952327
Itgam	11.6849457531227	-0.935750847726599	0.760221798710273	-1.23089189143762	0.218363300692555	0.649447512952327
Pdk1	164.659356335441	0.297922725380569	0.242110711353458	1.23052269647678	0.218501433180512	0.649447512952327
Ppp3ca	415.554824339934	0.176011147286068	0.143015157951878	1.23071672825962	0.21842882931353	0.649447512952327
Utrn	508.495350484407	-0.714570720284707	0.580678327484145	-1.23057928368132	0.218480257282063	0.649447512952327
Wdr6	1091.93409563411	0.15211875281358	0.123593522812297	1.2307987453728	0.218398144917063	0.649447512952327
Ece1	490.92526369332	0.296265066022635	0.240838884842733	1.23013800788895	0.218645429308031	0.649689673143914
Sdhc	11249.3774147655	0.154273073638223	0.125425296559161	1.22999967207935	0.218697227648127	0.649689673143914
Bin3	666.796659586766	0.137499366373486	0.111859300576032	1.22921711172355	0.218990414848217	0.650050941542134
Cst3	4369.03253748793	0.217859494665484	0.177231539051501	1.22923660106668	0.218983109716141	0.650050941542134
St7l	254.366622786337	-0.249983473660676	0.203342319986125	-1.22937258548901	0.218932143953135	0.650050941542134
Foxa1	46.9184797569646	-0.437207211415946	0.355847574506328	-1.22863619914366	0.219208236926075	0.650357823258096
Lap3	5208.91721924832	0.17024149932547	0.138555441527968	1.2286886566711	0.219188560784631	0.650357823258096
Cyp4b1	275.562141840345	-0.76724541614021	0.62469229077767	-1.22819735006667	0.21937289323597	0.650486292761323
Rps27a	7348.06863943095	-0.177835661517582	0.144815192022313	-1.22801799337586	0.21944021347571	0.650486292761323
Sema3b	1490.16941539454	0.16277135312725	0.132533501048511	1.22815251871805	0.219389718969183	0.650486292761323
Slbp	556.961781140885	-0.180162190201989	0.146722541300535	-1.22791078047754	0.219480462144965	0.650486292761323
Lias	1299.58095035312	0.15005863322291	0.122222060165543	1.22775408154358	0.219539297841514	0.650491047775457
Bag2	332.880606481574	-0.180442660839269	0.147012521135512	-1.22739654721615	0.219673583518992	0.650719299003613
Kiaa0415	569.589686746759	-0.12711915549969	0.103602603644081	-1.22698803918479	0.219827086465657	0.651004342044985
Timm22	604.01134533856	0.144348478276497	0.117709682669693	1.22630929760941	0.220082303842036	0.651420694132443
Ugt1a2	66.7829222080517	0.718306611433024	0.585729908498972	1.22634443112833	0.220069087878097	0.651420694132443
Arhgef6	50.1661206087972	-0.671323287847631	0.548717368735165	-1.22344092988177	0.221163202113959	0.65153867803485
Arid5b	156.642706488001	0.305091144177858	0.249054366898812	1.22499817199276	0.220575909256129	0.65153867803485
Dscr3	593.159125261582	-0.166671791061802	0.136047766237468	-1.22509759381775	0.220538451674105	0.65153867803485
Dtwd1	90.8624930758721	-0.319692290835172	0.261168692420684	-1.22408351426832	0.220920724397104	0.65153867803485
Dync2h1	612.465494285294	-0.201274929287257	0.164429104232203	-1.22408335329141	0.220920785117488	0.65153867803485
E2f1	89.347460955188	0.32483460641835	0.265477426997349	1.22358654026579	0.221108239666978	0.65153867803485
Exosc3	153.044918539021	0.262879845615774	0.214900852404212	1.22326106516003	0.221231107804513	0.65153867803485
Faim2	21.6437065013576	-0.713167818941512	0.581703957844776	-1.22599787971843	0.220199472925459	0.65153867803485
H2afz	2517.08877398221	-0.138852888752967	0.113535577704427	-1.22299011076907	0.221333431477592	0.65153867803485
Jak3	591.434814938612	-0.196358460809008	0.160293132697468	-1.22499609000474	0.22057669370246	0.65153867803485
Mri1	358.610390873539	0.164136969895927	0.134195496552675	1.22311831702564	0.221285011223988	0.65153867803485
Mss51	70.3745721950558	-0.409836367119795	0.334739066611935	-1.22434579049275	0.220821809868347	0.65153867803485
Mxi1	3729.24271685994	0.285827625675673	0.233570916600016	1.22372952008039	0.221054279688959	0.65153867803485
Myh10	1657.24605344854	-0.241149935356282	0.197190461400875	-1.22292900804182	0.221356511100578	0.65153867803485
Nucb2	403.114637414253	-0.289029501206271	0.235841973446477	-1.22552189070732	0.22037864735034	0.65153867803485
Pdk3	921.979345924318	0.170073091627937	0.139078349317481	1.22285814048384	0.221383281239714	0.65153867803485
Pkn1	1509.54762657317	-0.225740414343809	0.184293943571328	-1.22489328715482	0.220615429995998	0.65153867803485
Rft1	367.176863186563	0.168987057349326	0.138052256151747	1.2240803740547	0.220921908885768	0.65153867803485
Sf3a3	647.214338857197	-0.138994456526495	0.113493436447526	-1.22469158461652	0.220691446039911	0.65153867803485
Srp19	377.506975618793	-0.165643335432973	0.1351556339054	-1.22557477366361	0.220358735692329	0.65153867803485
Tmtc3	276.057071744673	-0.189052120440441	0.154401972888216	-1.22441518656832	0.220795643232064	0.65153867803485
Wdfy2	950.138467133842	-0.13299144470076	0.108659368801996	-1.22392984762412	0.220978692799651	0.65153867803485
Sec31b	20.9212494551771	0.599873361042471	0.490699933960255	1.22248510653164	0.22152423263842	0.651784734188009
Isoc1	3039.39216172805	0.121586716546253	0.0994943130874496	1.22204689668429	0.221689892964102	0.65193462443208
Slc25a29	312.486478157668	0.309602246389667	0.253322175237887	1.22216796101221	0.221644117080028	0.65193462443208
Fhod3	756.325523397194	-0.199355267916968	0.163170309868759	-1.22176190066264	0.221797680140099	0.652082884757466
Hspa13	110.021522014373	0.331520496917233	0.271421986878245	1.22142093472312	0.221926684789043	0.652124792996967
Tnfrsf12a	904.031548987479	-0.991737993132076	0.81192710041318	-1.22146186846995	0.221911194645405	0.652124792996967
Tmem80	203.665067731575	-0.228077307820569	0.186761582155132	-1.22122175871865	0.222002068002015	0.652177695763995
LOC684112	1086.65263413144	0.108989407782691	0.0892669905540072	1.2209374048154	0.22210972073133	0.652248473905629
Pla2g15	433.466405511323	0.16781691069183	0.137458513491397	1.22085497965416	0.222140932824977	0.652248473905629
Esrrb	47.4923350353611	-0.471647382051008	0.386426863991036	-1.22053466257447	0.2226225772916	0.652436162538645
Nyap1	257.532217949167	-0.221475265038329	0.181505120081852	-1.22021497210906	0.222383392596874	0.652512216730458
S1pr2	47.5371910436206	-0.54242860196606	0.444554111913079	-1.22016327693336	0.222402985009132	0.652512216730458
Grb7	934.610763357686	-0.239462921757003	0.196519781674756	-1.21851815484569	0.2230271299943	0.65383708009162
Lrp2	38097.4498971197	0.182534184713374	0.149796542308208	1.21854738367598	0.223016029905002	0.65383708009162
Sipa1	458.143609942975	-0.366649807317268	0.300853424825297	-1.2186991307484	0.22295840801882	0.65383708009162
Car7	358.676103269754	-0.203519176984626	0.167156576970117	-1.21753616084762	0.223400286823373	0.654099352149363
Hipk4	37.4396577588698	-0.426740112701541	0.350556232251716	-1.21732285277166	0.223481402710137	0.654099352149363
Impa1	1615.70684214618	-0.140406003840722	0.115323864137331	-1.21749305654138	0.223416676647152	0.654099352149363
Lyplal1	684.632405012417	0.229999314129356	0.188954152243626	1.21722286278636	0.22351943372762	0.654099352149363
Mrps5	808.756238022399	0.132118568295553	0.108478537453412	1.21792357637835	0.223253015963752	0.654099352149363

Ric8b	170.017237370912	0.279073970316111	0.22923223303424	1.21742900909763	0.223441031401517	0.654099352149363
Zfp280b	70.3336885708701	-0.355287998306863	0.291759894279807	-1.21774104416877	0.223322394498935	0.654099352149363
Chtf8	1551.11020507187	0.146649146794869	0.120581172392674	1.21618610836943	0.223914034332206	0.654916859037534
Mllt4	2024.67701078908	-0.127326254850688	0.104688550276778	-1.2162385906965	0.223894046995794	0.654916859037534
Ciapin1	1522.08107786817	0.184016376119406	0.151344218062195	1.21587979029225	0.224030717770501	0.65508956474904
Dgkq	1324.29814628155	-0.122460939862496	0.100745155729786	-1.21555164588712	0.224155763570092	0.655286627761746
Ska2	329.041941144143	-0.220449694590541	0.181400760684511	-1.21526334155755	0.22426566870494	0.655439339290395
Rasip1	304.450756658987	-0.398311178130258	0.327856541047209	-1.21489471235806	0.224406250766815	0.655513093347626
Slc7a11	58.6905178146296	-0.396788027247988	0.326566419402566	-1.21503009395114	0.22435461372737	0.655513093347626
Fam83h	554.192089496208	-0.153991882717952	0.126780578016378	-1.21463307020149	0.224506069986671	0.655516115052749
Sec11a	978.945574843717	-0.14288348810159	0.117639308197662	-1.21458966641926	0.224522632051764	0.655516115052749
Gfm1	2215.33509511804	0.197026183166694	0.162291018662957	1.21403010955199	0.224736226595733	0.655971225343376
Baz1b	1884.72398432821	-0.117070525613395	0.0965477256412515	-1.21256637415159	0.225295651510648	0.656087721001799
Clasrp	767.279120287382	-0.191600441356934	0.15800423316053	-1.21262853231452	0.22527187508651	0.656087721001799
Col17a1	386.031376309926	-1.06362240094509	0.876305487727969	-1.21375754898304	0.224840321226354	0.656087721001799
Dhrs4	4750.62008847756	0.196020122431133	0.161630287538227	1.21276850655093	0.225218339415568	0.656087721001799
G2e3	121.18648855796	-0.282856231139401	0.233242663980003	-1.21271222988455	0.225239862349151	0.656087721001799
Hp	34.0657071762193	-0.439244637525471	0.362020903902852	-1.21331291312212	0.225010207703055	0.656087721001799
Lyl1	31.909970541678	-0.569517984249343	0.469507278523536	-1.21301204539429	0.225125215252586	0.656087721001799
Ptbp1	1740.84180564102	-0.107155962923785	0.0883252494159091	-1.21319739975152	0.225054358054901	0.656087721001799
Sorbs3	470.87118408173	0.270068060695421	0.222675800558098	1.21283076121672	0.225194531915583	0.656087721001799
Dusp10	30.5481518836598	-0.551282766913943	0.455265683209442	-1.21090340705589	0.225932426699568	0.656763787644195
Foxk1	63.6760876984141	0.355736827267329	0.293944961178218	1.21021576910668	0.226196108707361	0.656763787644195
Galnt2	1505.47927906999	0.152109981396984	0.125604427561989	1.21102404070839	0.225886191085658	0.656763787644195
Hspa5	9869.54060797094	-0.209703582474556	0.173255141362784	-1.2103743694131	0.226135272275866	0.656763787644195
Hspb6	626.005606766609	-0.204249754283488	0.168562036740175	-1.21171859472915	0.225620118708887	0.656763787644195
Krt18	762.947850053179	-0.250728765001485	0.207239687414905	-1.20984917574939	0.226336772497124	0.656763787644195
LOC302022	117.223854794363	-0.362209499326575	0.298970822301095	-1.21152123320513	0.225695701938976	0.656763787644195
Ormdl3	197.40353758482	0.283508038332011	0.234175945913342	1.21066250944888	0.226024776443934	0.656763787644195
Pycrl	584.540392772141	0.138979342685987	0.114854471376959	1.21004729741735	0.226260744427664	0.656763787644195
Rbpms	481.071316700394	-0.160852945978592	0.132866986713215	-1.21063139879722	0.2260367048889	0.656763787644195
Sh3bp5	1886.06124544601	0.237148128946403	0.195748531513504	1.2114937829306	0.225706215958881	0.656763787644195
Stk4	409.119143371694	-0.232531858018853	0.192065050738579	-1.21069323713325	0.226012995277279	0.656763787644195
Tbc1d8	175.163717627868	-0.223370823853474	0.184560450957691	-1.21028542515146	0.226169388372576	0.656763787644195
Uqcrq	3539.16636200155	0.185189321325668	0.1530524096232	1.20997324891249	0.226289157989665	0.656763787644195
Aox2p	673.225401776786	0.856813913448378	0.708389575501846	1.20952360548415	0.226461747829218	0.656923336606015
Ppap2c	1185.19581529142	0.138811446644725	0.114776659100908	1.20940483659388	0.226507351412896	0.656923336606015
Slc5a2	25.5074460914262	0.563400455760995	0.465970246068994	1.20909105358966	0.226627865907218	0.657105184668734
Zcchc2	106.197157098386	-0.271508246161795	0.224623911243211	-1.20872370469865	0.226769011534847	0.657346744479743
Slc19a2	756.000678844083	-0.158987876068716	0.131556657252386	-1.20851258605412	0.226850157535499	0.657414301516696
Hopx	176.943491979651	0.57068901242872	0.472489707967092	1.20783374284307	0.227111219462629	0.657893495046097
Pkd2	1234.98786010657	-0.18825586025628	0.155963052507253	-1.20705421720009	0.227411264852012	0.657893495046097
Plod1	427.096524248002	-0.193544649378655	0.160347870350019	-1.20702974698804	0.227420688196033	0.657893495046097
Sccpdh	884.679383212584	0.164417821970187	0.13619510964444	1.20722265578717	0.227346407630582	0.657893495046097
Taf1d	280.296401390705	-0.252641249965695	0.209208877831951	-1.2076029114244	0.227200038811975	0.657893495046097
Ttc13	572.839264303195	0.140252611492234	0.116193101280566	1.20706487688604	0.227407159952637	0.657893495046097
Xrcc4	99.4479084400326	0.294644829099729	0.244085609650084	1.20713723976651	0.227379295392589	0.657893495046097
Atp9b	616.196560354144	0.185939446045021	0.154079914677006	1.20677277395176	0.22751966390782	0.658012340960887
Cdkl1	2060.55012884917	-0.130782327256237	0.108425628973239	-1.20619385374758	0.227742753246223	0.658489985600757
Amdhd2	1253.30022987243	0.204338278076434	0.169430479125953	1.20603022036271	0.227805838307344	0.658504872380791
Bcl2l13	52.7156333566683	0.468439440190028	0.388572392414754	1.20553968664358	0.227995026830688	0.658549294779567
Rfx2	30.0189905855181	-0.530218377018766	0.439803425995371	-1.20558036995452	0.227979331878965	0.658549294779567
Rps15a	4803.83243582734	-0.192198523394176	0.159390306484494	-1.20583571004598	0.227880843451038	0.658549294779567
Aadat	95286.0892987946	0.238410879865286	0.197865809155438	1.20491195969081	0.228237291134745	0.658579601685076
Ovol2	52.7308460019169	-0.544325001102127	0.451717734182308	-1.20501135977638	0.228198916552438	0.658579601685076
Plbd1	80.1682085491367	0.466279132142458	0.386888330551997	1.20520340191494	0.22812478942423	0.658579601685076
S100a13	399.902803925108	-0.173889534087998	0.144303821494999	-1.20502376365705	0.228194128209707	0.658579601685076
Gfpt1	106.700113878587	0.368073122732516	0.305518785634286	1.2047479239889	0.228300629114932	0.65859516510668
Cnot8	831.234569649779	-0.12886008833507	0.107016494803946	-1.20411426828305	0.22854541582946	0.658632656267152
LOC680200	60.5105024742931	-0.483384067055942	0.401362629668984	-1.20435743470837	0.228451456432454	0.658632656267152
Myt1	68.8938964751701	-0.345697180060211	0.287079890663006	-1.20418458869352	0.228518241226706	0.658632656267152
Vsig10	271.445978659887	-0.308135655774887	0.255890140198202	-1.20417166341859	0.228523235894282	0.658632656267152
Atg4c	161.771548712953	-0.231660008017359	0.192469285415971	-1.20362065831277	0.228736231118841	0.658863926217046
Hiatl1	1011.92307826714	-0.139334499728998	0.115792976238296	-1.20330700751879	0.228857538334058	0.658863926217046
Pten	395.135451642922	0.154604686796349	0.128479744750217	1.20333899399413	0.228845165185408	0.658863926217046
Tnfrsf11b	1227.40002694061	0.22811866635345	0.189552182862479	1.20346103594571	0.228797960757457	0.658863926217046
Gc	4277.41014007324	-0.32464510040843	0.26989719386302	-1.20284726106933	0.229035432090195	0.659042207882824
Hspa12a	958.741595179703	0.219707631621981	0.18263481294065	1.20298878447329	0.228980660706323	0.659042207882824
Cpt1c	32.5534231144861	-0.499518211463608	0.415534811789187	-1.20210917904281	0.229321230496176	0.65969757170831
Cfd	186.714475207634	1.89428376497377	1.57611700354692	1.20186747602547	0.22941487731412	0.659799973570922
Itgb6	382.28080619054	-0.228319236837417	0.19006067356806	-1.20129657835636	0.229636177248263	0.660102374963013
Ubtd1	475.790943605823	0.201340892254953	0.167590411913824	1.20138670199392	0.229601232078408	0.660102374963013
Faf1	1369.13497091064	0.105578080267673	0.0879370433229762	1.20060984856978	0.22990257892606	0.660144653917004
Fam38a	422.222528571361	-0.384567627415272	0.320279613298993	-1.20072465260617	0.229858027936423	0.660144653917004
Fam46c	55.8904354165525	0.41070021039355	0.342190040310275	1.20021088288004	0.230057449776989	0.660144653917004
LOC679203	961.146895544842	-0.113762150595377	0.0947212488005608	-1.20102038387297	0.229743294395326	0.660144653917004
Ntf3	18.2701384142305	0.647423575632999	0.539404196606503	1.20025683838959	0.230039606947251	0.660144653917004
Spats2	1115.17674558197	0.161726752930291	0.134716430363517	1.20049761186434	0.229946139570862	0.660144653917004
Tmem189	512.947020192429	0.194050018838105	0.161622923687696	1.20063425664213	0.229893106586727	0.660144653917004
Vwa1	153.595255094551	-0.448271216940109	0.373592159078581	-1.1998946071184	0.230180274687595	0.660330389222414
Eepd1	176.377762793439	0.264255739166205	0.220315057732083	1.19944474919892	0.230355056215342	0.660665043891895
Rps23	6311.18938872783	-0.180657056590686	0.150672274588871	-1.19900663266438	0.230525366581796	0.660986709527926
Ehmt1	521.301617020704	-0.165624198270323	0.138156427274554	-1.19881645420074	0.230599323084359	0.661032006601974
B4galt7	323.93360167043	0.175616755504043	0.146548425319473	1.19835307081056	0.230779593981343	0.661219567898083
Cnpy3	1598.83651084193	0.112499624693949	0.0938827840695415	1.19829877020496	0.230800725200363	0.661219567898083
RGD1562658	21.3799033716785	0.563813454015969	0.470667891159802	1.19790082265149	0.230955629469945	0.661219567898083
Slc17a3	7168.63289979091	0.282101861060325	0.235475820824308	1.19800776178547	0.230913995299472	0.661219567898083
Trim35	1269.1493322934	-0.13337891507505	0.111336898383888	-1.19797584638258	0.23092642023283	0.661219567898083
LOC500956	179.418895021169	-0.347839706174724	0.290527373588396	-1.19726999173415	0.231201336801265	0.661756331927267
Psmd2	3666.0525748066	0.104643314871856	0.0874344065377698	1.19682078275047	0.231376415931308	0.662090720915218
Atp10a	87.9939012340353	-0.462663863862507	0.386716425853902	-1.19639051493845	0.231544201023056	0.662095994201327
Noc4l	244.887518243193	0.182169813988267	0.152275094601649	1.19632047817684	0.231571520385026	0.662095994201327
Pik3r1	252.093951043102	0.288457610148348	0.241171320445801	1.19606929055718	0.231669520406359	0.662095994201327
Tmem14a	215.598265660947	-0.237259323221713	0.198316335151334	-1.19636803010031	0.231552971473679	0.662095994201327
Trpc4ap	2446.36795836052	0.0907489005252084	0.0758689677420162	1.19612673305098	0.231647106809888	0.662095994201327
Ap5b1	616.895726955086	-0.154388842110746	0.129201667532722	-1.19494465558384	0.232108654141482	0.662454148562319
Ctse	74.5469523593279	-0.702773520507332	0.58815791745629	-1.19487215873373	0.232136982084713	0.662454148562319
Gramd1b	2826.24616197251	0.212379527126271	0.177685090305374	1.19525800820581	0.231986240697981	0.662454148562319
LOC292543	20.9419592411176	-0.552379139189445	0.462020708064321	-1.19557225368467	0.231863524489466	0.662454148562319
RGD1309594	597.772019950519	0.139502457585601	0.116762387855454	1.19475509320945	0.232182730294554	0.662454148562319
Serbp1	2168.22784815016	0.109745960711127	0.0918305604736138	1.19509191869368	0.232051119044481	0.662454148562319
Tmem194b	70.7808849780829	-0.318102476050194	0.266260565762864	-1.19470367359432	0.232202826664814	0.662454148562319
Shank2	235.606452906451	-0.319192950615537	0.267216022661987	-1.19451276699563	0.232277449647924	0.662462001753037
Stip1	1718.84729184742	-0.144602628569555	0.121067329156493	-1.19439843578808	0.232322148424037	0.662462001753037
Kcnj10	26.3339413008205	0.709615108689709	0.59427654100261	1.19408231644566	0.232445769793148	0.66252976323979

Nat8	6920.24038517012	0.254376050170846	0.213064756109558	1.19389079083566	0.232520690344461	0.66252976323979
Pnmal2	59.8885267809252	-0.410668834926336	0.343975278147182	-1.19389055265366	0.232520783526616	0.66252976323979
Foxj3	271.749569486547	0.254672540356969	0.213353691100466	1.19366362514462	0.232609574713859	0.662616648169855
Ace2	878.397920653065	0.248685780810927	0.20842369471595	1.19317422690279	0.232801146163385	0.662664119031564
Agtrap	2103.38517485381	0.215337737280871	0.180471572803841	1.1931947726467	0.232793101428446	0.662664119031564
Ccdc102a	123.389422383661	-0.321874344555406	0.269702730297674	-1.1934411794799	0.232696635611614	0.662664119031564
Eif3d	1882.2433133932	0.148710794265044	0.124738906949422	1.19217650612685	0.233192043458629	0.663281585741381
Gnpat	614.52759259234	0.142867656879464	0.119855450005378	1.19199967021152	0.233261374581822	0.663281585741381
Lypd2	52.2739630512659	-1.43320246314431	1.20258062868679	-1.19177245080802	0.233350480748565	0.663281585741381
Rbbp8	632.908402861862	-0.223324307656152	0.187395486098805	-1.19172725184215	0.233368208813988	0.663281585741381
Snx13	403.592248961824	0.245372288420096	0.205815218021595	1.19219701428662	0.23318400387852	0.663281585741381
Usp43	15.0697271086525	-0.808953423949337	0.678719655700761	-1.19188153334695	0.233307700021814	0.663281585741381
Apeh	1761.00102013212	0.0964572090801487	0.0809588711099275	1.19143470947337	0.233482973696866	0.663441869759645
Hspa9	12305.0117426499	0.183955778802836	0.154454932339997	1.19099970467695	0.23365370081658	0.663522972030485
Phax	541.389577998446	-0.153709728313601	0.129068512654829	-1.19091577916197	0.233686649396272	0.663522972030485
RGD1359634	359.312107706269	0.186179147123418	0.156323566232903	1.1909857970233	0.233659160638056	0.663522972030485
Arl1	924.717247191339	0.141744542315897	0.119175365362111	1.18937787088137	0.234291004816302	0.664574884138279
Blk	20.1587275404696	-0.569308032368987	0.478542072333792	-1.18967184973422	0.234175393737635	0.664574884138279
Hemk1	150.189885470896	0.261904186604681	0.220184779115913	1.18947452978484	0.234252987960819	0.664574884138279
Rfx1	471.209154235023	-0.164611968119332	0.138366102627332	-1.18968421451235	0.23417053201078	0.664574884138279
Ing3	137.119732899467	-0.260000553888744	0.21873431257989	-1.18865920404592	0.234573800308288	0.66483167924256
Papolg	174.049723876477	-0.251652392226853	0.211730008095178	-1.18855326408776	0.234615508133196	0.66483167924256
RGD1565002	240.602507403565	-0.215240027384878	0.181086073945724	-1.18860618431316	0.234594673151937	0.66483167924256
Tpr	1642.73969512268	-0.117732853744115	0.0990535727079707	-1.18857755985455	0.234605942594632	0.66483167924256
Tmem185b	1482.119642699	0.1658134449335	0.139545833198828	1.18823644628102	0.234740268439849	0.665019414528246
C1galt1c1	715.047543147688	-0.164439262666358	0.138481437134127	-1.18744624600544	0.235051647668158	0.665238303634533
Ncl	6255.12732925285	0.109689267399501	0.0923721895860813	1.18747068669712	0.235042012408243	0.665238303634533
Necab2	88.1283014479462	-0.521605679671307	0.439172022729428	-1.18770243247637	0.234950665108606	0.665238303634533
Rcbtb2	228.581741556731	0.222724740509344	0.187537748618384	1.18762618272954	0.234980717717895	0.665238303634533
Ezr	8149.9145595151	0.207979970544599	0.175314693027744	1.18632367289195	0.235494501368222	0.665260918781865
Fam111a	1596.09922920011	-2.1138284013699	1.7832946380279	-1.18535005730042	0.235879069390367	0.665260918781865
Fancc	200.004387216469	0.203161269750633	0.171303007437752	1.1859760829036	0.235631744834366	0.665260918781865
Gngt2	45.8269974383308	-0.53332627064457	0.449407898869747	-1.18673096753723	0.235333755931216	0.665260918781865
Map4k1	53.4389876885701	-0.565958856366081	0.477110944482694	-1.18622065352058	0.235535171943593	0.665260918781865
Myh11	531.251798807332	1.28978273955147	1.08723075126255	1.18630082717371	0.235503520102356	0.665260918781865
P4ha1	662.36168470347	-0.252046709053528	0.212553574537989	-1.18580320091714	0.235700027144391	0.665260918781865
Pced1b	47.1525409454697	-0.519276550314209	0.437547967349022	-1.18678771029461	0.235311367646727	0.665260918781865
Pecam1	858.975127325647	-0.723167679469207	0.609696437104345	-1.18611104716927	0.23557844842176	0.665260918781865
Pla2g16	669.230967676582	0.349068933434049	0.294342895562336	1.1859261381769	0.235651469808532	0.665260918781865
Slc25a27	25.3918071390489	-0.563168585556302	0.474901997016852	-1.1858627445113	0.235676507936437	0.665260918781865
Slc26a6	175.421401105705	-0.247660592906908	0.20892754347262	-1.18538986669972	0.235863336385578	0.665260918781865
Tnfrsf9	43.298299295575	-0.436683842094936	0.368335522450254	-1.18555994596995	0.23579612800875	0.665260918781865
Ttpa	63.9692106879129	0.378482555880007	0.3192413692026	1.18556863988329	0.235792692893537	0.665260918781865
Lypd3	29.603775978995	-0.774602866014397	0.653597423108802	-1.18513757647642	0.235963056131243	0.665320705720696
MGC116121	226.348585734532	-0.322723140874588	0.272351780147121	-1.18494962911664	0.236037363223398	0.665320705720696
Pik3c3	542.360482663934	0.148096600384722	0.125003542864314	1.18473922411523	0.236120568830784	0.665320705720696
Taf5	82.1322184333511	-0.335834702289171	0.283475567687635	-1.18470422346673	0.236134412007504	0.665320705720696
Sema3e	31.1127008201897	0.650517413280226	0.549289162395726	1.18428954695373	0.236298465236834	0.665532744024494
Tgoln2	4165.56562116937	0.147766769958112	0.124780040212222	1.1842180023888	0.236326777659938	0.665532744024494
Plau	18192.5205982086	0.245606891594888	0.207433188852403	1.18402890566199	0.236401620701165	0.665578603143286
Fam167a	62.3249918412445	-0.595628908176804	0.50314490243855	-1.18381187067586	0.236487542148205	0.66560966961367
Tmed3	256.947054119749	-0.260018379784134	0.219664810723419	-1.18370520488839	0.236529777896324	0.66560966961367
Gpr132	16.7439581422054	-0.787922308135824	0.665742089189185	-1.1835248528382	0.23660120283966	0.665645859276132
Atp5c1	11380.506344144	0.154822620411388	0.130926016616217	1.18251990255855	0.236999473186211	0.666014202594805
Cacna1h	31.2066203963575	0.565386452207821	0.478306936831179	1.18205781407552	0.237182761715336	0.666014202594805
Ern1	71.4701118746063	-0.412233464832124	0.348807491727938	-1.18183661362886	0.237270536856375	0.666014202594805
Exog	218.12988699356	-0.186794580470633	0.157985284706695	-1.18235429848687	0.237065148934449	0.666014202594805
Gjb1	637.278313110636	-0.138933368770671	0.117576264095889	-1.18164469537291	0.23734671104641	0.666014202594805
Mphosph8	713.763139178516	-0.12640003508414	0.106981115626287	-1.18151726446458	0.237397299135939	0.666014202594805
Ppm1a	854.967372810594	0.141586290070344	0.119789861885017	1.18195553315062	0.237223345230452	0.666014202594805
Slc9a5	34.6751727334527	-0.512160380327867	0.433071398383401	-1.18262342477405	0.236958424574744	0.666014202594805
Slpi	13.6035610246277	1.05446842747367	0.891425372403014	1.18290151942966	0.236848179409639	0.666014202594805
Sod2	4163.13350698839	0.170037549172771	0.143926227214142	1.18142156898047	0.237435293763343	0.666014202594805
Zbtb42	53.5341126783936	-0.423152755242614	0.357862507389748	-1.18244506341023	0.237029151522373	0.666014202594805
Zfp37	122.431819274853	-0.304791749078921	0.257966518412508	-1.18151669819234	0.237397523954215	0.666014202594805
Pyroxd2	12239.883483484	0.186833063779175	0.158172359661861	1.18119919421184	0.237523601316606	0.666097520988043
Chd6	1399.20584310147	-0.284731720493324	0.241117153973959	-1.18088537377177	0.237648262457839	0.666282721039911
Myo7a	1295.88326661895	0.146204322169258	0.123869986296233	1.18030466088543	0.237879065062194	0.666711212324608
Nedd9	1210.9467236036	0.224027888933572	0.189821053176404	1.18020569997248	0.237918412562785	0.666711212324608
Ralgapb	1477.98244517373	-0.12668608458486	0.107381339357146	-1.17977746732611	0.23808873361455	0.667024043939606
Pon2	1089.30287609708	0.119564639108948	0.101357956170302	1.17962756577347	0.238148374324686	0.66702671822927
Myl3	53.8283862417047	0.372219953705161	0.315598014931419	1.17941158085562	0.238234325896282	0.667103066799
Cd44	224.226770856034	-0.337258879995623	0.286056663236652	-1.17899326720669	0.238400856813177	0.667114831903953
Ddx17	1680.36190191627	-0.18798338038421	0.15944865188181	-1.17895873163952	0.238414609114363	0.667114831903953
Dnajb2	2654.87736800182	0.16430951407191	0.139347162082748	1.17913785696144	0.23834328625451	0.667114831903953
Lrrc58	46.4766548750907	0.589035110957869	0.499805512894666	1.17852863916291	0.238585921817509	0.667429876292839
Cerk	215.015209891469	-0.375417417573238	0.318919050561324	-1.17715582343693	0.239133317385951	0.667810635235558
Cul4a	1207.75219083588	0.160601711276434	0.136372379221611	1.17767037719162	0.238928040880677	0.667810635235558
Magix	177.708216542904	0.241678199863916	0.205264666771549	1.17739795974186	0.239036703834099	0.667810635235558
Mrpl16	1386.17219073419	0.129610266731657	0.110050765857428	1.17773161978326	0.238903616991556	0.667810635235558
Paqr9	1137.73506503179	0.131849053116418	0.111997207801229	1.17725303786521	0.239094525051893	0.667810635235558
Serinc5	469.005923478409	-0.320705859960589	0.272241320499099	-1.17802051272981	0.238788428617064	0.667810635235558
Ubap2	1124.47802884005	0.104082253007862	0.0883857486376057	1.17759089686069	0.238959740709161	0.667810635235558
Mdh1	16746.9174256076	0.161985905259255	0.137716740097401	1.17622523699508	0.239504882205416	0.668203579545785
Pou3f1	402.91394895317	-0.856367962481806	0.727927016278363	-1.17644756044379	0.239416075844057	0.668203579545785
Slc44a4	2122.38153949221	0.161133211979105	0.13699305368647	1.17621446958824	0.239509183799888	0.668203579545785
Tnfsf13	467.338092678868	0.635404653239118	0.540155447318656	1.17633665714802	0.239460372893156	0.668203579545785
Agpat2	499.970507812993	0.585409306267486	0.497803038774194	1.17598580295736	0.239600549310849	0.668294440114627
Bcl6b	235.899199096906	-0.4422122006396	0.37623689996836	-1.17535574175948	0.239852422243631	0.668832833960036
Abhd2	276.283047442743	0.266387613835628	0.226828567395594	1.17440063610261	0.240234590606587	0.669734205747969
Usp5	1883.47620875232	0.112004866380579	0.0954218846965633	1.17378593743719	0.240480778072702	0.670256136236962
Scyl1	1389.83747016672	0.103697843051014	0.0883773942858174	1.17335257380015	0.240654447462887	0.670575741569789
Efhc2	308.564169097853	0.252431490835349	0.215326881135838	1.17231759222901	0.241069571142094	0.671567829804177
Smarcb1	1131.37950145542	0.151399196364741	0.129219046271	1.17164768456211	0.241338534967976	0.672152361785352
Polb	188.230396050074	0.237721870550799	0.202964631208206	1.17124776437988	0.241499201140053	0.672435061283155
Clpx	2075.70029514232	0.179038772378786	0.152900170726105	1.1709520763028	0.241618040925125	0.672601188624777
Cul5	192.595838289638	0.256353775818821	0.219015987194741	1.17047974032544	0.241807962520414	0.672635658836766
Gins2	1048.09866086856	-0.12875980499078	0.109978722224015	-1.17077014887034	0.241691179699857	0.672635658836766
Rgs7	24.8997803700805	0.792597380463986	0.677100902644024	1.1705749872271	0.241769656235573	0.672635658836766
Anpep	28204.4596560561	0.716393835945042	0.612226106048194	1.17014584786204	0.241942280719672	0.672844620176117
Add3	847.40577715664	0.15743543113202	0.134692253957454	1.16885289618622	0.242462904300374	0.673405662233382
Bzw1	1987.73422582518	0.167916954538305	0.143749387743739	1.16812291985305	0.242757186383326	0.673405662233382
Clmn	609.469020446544	0.153123964708595	0.131079342711276	1.16817769712102	0.242735094807191	0.673405662233382
Dctn1	1993.85499336507	0.157769051237838	0.134994635398957	1.16870608059035	0.242522071258772	0.673405662233382
Dtx2	218.092475563449	-0.211668284342485	0.181069726556596	-1.1689877063813	0.242408584482031	0.673405662233382

Iba57	533.195836782357	0.169953172249364	0.145367933280799	1.16912422439875	0.242353585244371	0.673405662233382
LOC363060	853.725916769371	-0.333204273070608	0.285215742788218	-1.16825344145896	0.242704549577244	0.673405662233382
Nr2f2	659.880291798308	-0.231788975032529	0.198444821955532	-1.16802732743749	0.24279574202291	0.673405662233382
Ppp1r42	31.413039716317	-0.552884525338358	0.473299858075941	-1.16814851283908	0.242746864600991	0.673405662233382
St3gal2	777.762678431007	-0.166877963564418	0.142706653830339	-1.16937759442398	0.242251532908923	0.673405662233382
Supv3l1	855.161187913968	0.111469731522358	0.0954038168564066	1.16839907663372	0.242645827238318	0.673405662233382
Pam	2715.20312429645	0.182504015686251	0.156381408134181	1.16704420214489	0.243192519396817	0.674203940592654
Rnf14	1876.85798935568	-0.11342269412103	0.0971899973835974	-1.16702024050236	0.243202195709122	0.674203940592654
Itga7	495.628307373213	0.203384014902973	0.174324503152588	1.16669780337735	0.243332430227975	0.674400488166586
Prkaca	1315.37848414707	0.117604371660895	0.100826868380352	1.16639913100596	0.24345310972064	0.674570464428277
Kirrel3	51.5753131852398	-0.439604417367469	0.376994014518245	-1.16607797587777	0.243582920356195	0.674601236054705
Podxl2	99.8263671098323	-0.355774376722876	0.305080451126934	-1.16616576187915	0.243547432498851	0.674601236054705
Eln	314.605709917955	0.876937569520738	0.752816539692922	1.16487553511835	0.244069377774598	0.675672439400999
Heatr2	398.9962713357	0.163222520913516	0.140126300733222	1.16482430535482	0.24409011834409	0.675672439400999
Nudt6	241.112361963113	0.213683198405453	0.183469254949657	1.16468123481554	0.244148047564658	0.675672439400999
Fbxw5	1762.95779596783	0.201048686495972	0.172676909981316	1.16430556070135	0.244300203861729	0.675928947685593
Abca7	1486.49179541159	-0.183890207322405	0.158075774890494	-1.16330416504233	0.244706116413923	0.676031194422676
Bmp4	1415.00562344263	-0.191740923506762	0.164924098538717	-1.1626010098321	0.244991420889231	0.676031194422676
Ccar1	1842.18774674896	-0.169788681272019	0.145931255179471	-1.16348400528185	0.244633183892636	0.676031194422676
Doc2g	99.4101720556798	-0.478643003406649	0.411387127119587	-1.16348561209965	0.244632532331536	0.676031194422676
L2hgdh	830.906883187504	0.190157658133485	0.163437253175241	1.1634902963622	0.244630632880276	0.676031194422676
Mbrl	2605.84197750855	0.128976348631743	0.1109106343495	1.16288532103526	0.244876033826683	0.676031194422676
Otud5	1800.75685802567	-0.121219183417969	0.104145925124785	-1.16393592233904	0.244449980518172	0.676031194422676
Rfx6	25.0765174071101	0.69759966721504	0.59982819929512	1.16299911880571	0.244829859946522	0.676031194422676
Scaf4	575.049188028847	-0.205493564816825	0.176655677460982	-1.16324347889817	0.24473073055355	0.676031194422676
Syk	179.212042154245	-0.338920397882669	0.291477662980003	-1.16276627998737	0.244924341726295	0.676031194422676
Zfp772	139.763872004072	-0.258517961144588	0.222337791330513	-1.16272613664805	0.244940633752636	0.676031194422676
Rab2a	944.835537178843	0.112944401710461	0.0971740661386032	1.16228955109652	0.245117869500823	0.67609438333568
Spop	1344.40106559283	-0.118576342412402	0.102024193650388	-1.1622374867155	0.245139011498068	0.67609438333568
Tril	301.956915087499	-0.78799656666243	0.678076845271751	-1.16210510970424	0.245192772137047	0.67609438333568
Add1	6320.67069198647	0.180845633926696	0.155835101524067	1.1604935740281	0.245847908527624	0.67626024402831
Ap3d1	2845.15803836816	0.122689659406318	0.105681384929384	1.16093917096468	0.24566663769689	0.67626024402831
Chac2	218.32124955867	-0.23612729935648	0.203409881563371	-1.16084478070411	0.245705028251424	0.67626024402831
Cyb5d2	99.51256387348	0.279872738680027	0.241145542223449	1.16059677528972	0.245805917449322	0.67626024402831
Mmadhc	2717.50919551082	0.224232242816675	0.193015090050717	1.16173426004027	0.245343424768746	0.67626024402831
Parp1	843.863641617547	0.1137780115608	0.0980113465615919	1.16086571149495	0.245696514884421	0.67626024402831
Syvn1	1427.87299836827	-0.132285440491087	0.113989047034014	-1.16051010104166	0.245841183590216	0.67626024402831
Thnsl2	7763.43181711637	0.186428552103282	0.160508185148104	1.16148937782369	0.245442940425446	0.67626024402831
Zc3h13	937.203668512296	-0.117671257995395	0.101306245288148	-1.16154001819631	0.245422358781941	0.67626024402831
Zdhhc3	669.259493854442	0.162134931428666	0.13961639164932	1.16128865323999	0.245524532339399	0.67626024402831
Becn1	1145.86914970369	-0.16563253042586	0.142807043609441	-1.15983446081865	0.246116210655797	0.676661203172993
Cdh3	92.3824175190732	-0.299948993806598	0.2588229918035	-1.15889624687718	0.246498478931187	0.676661203172993
Cyb561d1	137.949606864052	-0.277713540270658	0.239555503269232	-1.1592868311547	0.246339287759578	0.676661203172993
Elovl6	116.989893257367	-0.328423856524192	0.283456571848654	-1.15863906199906	0.246603339610583	0.676661203172993
Fam35a	336.49855172437	0.236990923370466	0.204488746344649	1.15894359766398	0.246479176242613	0.676661203172993
Kat7	689.763195233942	0.159730200950869	0.137771257622274	1.15938697016761	0.246298485519958	0.676661203172993
LOC500594	58.3121001172515	-0.417198555719125	0.3597906194503	-1.15955929133599	0.246228283318454	0.676661203172993
Sp2	253.17087470511	0.241292928245917	0.208202230127626	1.15893536826193	0.246482530906698	0.676661203172993
Tmem2	379.791800096896	-0.232603981952829	0.200729139489448	-1.15879529272359	0.246539636727801	0.676661203172993
Traf6	86.0598551506369	0.276105376044801	0.238324257431639	1.15852821286561	0.246648545200221	0.676661203172993
Unc119b	2544.05035635003	0.134129121348214	0.115769587196443	1.1585868499351	0.246624631585451	0.676661203172993
Sec16b	819.875001729551	-0.272257136141956	0.235094510599834	-1.1580752585303	0.24683332567731	0.677004724818608
LOC685909	1169.02710027102	-0.116782418276724	0.100856316215807	-1.15790882176223	0.246901247089324	0.677027641596444
Pfkl	1930.65110622688	0.187322462624231	0.161816462165827	1.1576230262176	0.247017908330132	0.677184164515264
Rab3ip	2013.29541802201	0.119583106807941	0.103319075670932	1.15741556950055	0.247102615979672	0.677253034295866
Gdi2	5224.50231145936	0.13924107355942	0.120353213059974	1.15693690279821	0.247298140298622	0.677375619645283
LOC499843	107.544757769042	-0.313319420399446	0.270801941006774	-1.15700581478332	0.247269984666863	0.677375619645283
Smurf2	784.089370639919	-0.125094397922248	0.108131917438198	-1.15686839636174	0.247326132458906	0.677375619645283
Cyc1	6626.49559672331	0.146374073126395	0.126587320785071	1.1563091170475	0.247554740492136	0.677838395671795
Klhl31	28.4131289043319	-0.450800951091296	0.390036939451749	-1.15579040212181	0.247766899829552	0.678255920872516
Bcat2	1125.94563029946	0.301144857930075	0.260766460293083	1.15484505787903	0.248153881864422	0.678447377726795
Dgcr2	385.173822983059	0.313468790236934	0.271339890061507	1.15526246496922	0.247982961791921	0.678447377726795
Gpha2	95.571312180928	-1.39892253900997	1.21115319931009	-1.15503351665738	0.248076701462036	0.678447377726795
Pfn2	337.296199173026	-0.254623669063691	0.22045930948053	-1.1549690038659	0.248103119795368	0.678447377726795
Slc25a38	2097.39310861459	0.125161464030509	0.108398218635139	1.15464502651832	0.248235820018936	0.678447377726795
Tcerg1l	13.9037426513198	2.39330885595306	2.072284166988	1.1549134496509	0.248125871121142	0.678447377726795
Tnip2	190.051452323072	-0.218918177242014	0.189605375481192	-1.15459900167087	0.24825467569644	0.678447377726795
Arhgef39	180.260583550204	-0.264065276974722	0.228740678823668	-1.15443076558449	0.248323607978859	0.678472626992239
Med27	195.143096457151	-0.190586821475969	0.165189356759329	-1.15374758528567	0.248603668729669	0.678917635482243
Plek2	83.7130300626858	0.27615445793158	0.239355463396913	1.15374202874845	0.248605947464112	0.678917635482243
Dis3l2	261.451551610982	0.185673671831937	0.161011565938096	1.15316977851966	0.248840705359264	0.67939549774523
Dars	1045.13405470934	0.152050782204013	0.132013531121729	1.15178179775987	0.249410749462381	0.679643206824525
Fan1	110.059134816689	-0.27212500992092	0.236098322563631	-1.1525918819164	0.24907793687244	0.679643206824525
Fbxo32	45.2867316689289	-0.460443571831549	0.399555579267128	-1.15238929381515	0.249161138443465	0.679643206824525
Klhl8	682.389996939175	0.268335628800999	0.233016087583882	1.15157554820932	0.249495534035974	0.679643206824525
LOC684903	198.096504420605	0.244472682650665	0.212111865439286	1.15256486073686	0.249089033166479	0.679643206824525
RGD1311517	49.3030405085725	-0.393321922196316	0.341544557848624	-1.15159768515662	0.249486433067665	0.679643206824525
Rhbdf2	108.908899959232	-0.25969573160815	0.225499967517829	-1.15164420849691	0.249467307092388	0.679643206824525
Tcp11	31.9521309051077	0.561806829337898	0.487853900355632	1.15158827043989	0.249490303628937	0.679643206824525
Ttk	26.0709614503294	0.637781026936585	0.553873033659938	1.15149319099757	0.249529394868797	0.679643206824525
Zfp395	425.756057183666	-0.167507992723489	0.14540040602	-1.1520462515108	0.249302067909877	0.679643206824525
Ccny	970.631640478908	0.126456105870788	0.109872076032	1.15093944191933	0.249757149940507	0.679774848233668
Cyb561	1108.67972956655	0.10787246707558	0.093703390680499	1.15121199235354	0.249645032749587	0.679774848233668
Slc4a5	53.670736081071	-0.59414154534899	0.51619544054103	-1.15100114934425	0.249731762702691	0.679774848233668
Xrn2	1391.78166090859	0.145741191451383	0.126644695696945	1.15078796351752	0.249819477772595	0.679781705617265
Kti12	390.137562658141	0.169226536732861	0.147140508632283	1.1501016158356	0.250102020947644	0.680062098107875
Ppp2r1a	3101.66162076812	0.0985813269392038	0.0856976411368852	1.15033886150658	0.250004330719013	0.680062098107875
Ttl	59.0460385468038	0.372874246263015	0.32417576671004	1.15022245508109	0.250052259855012	0.680062098107875
Rhot1	1019.09132806114	0.132054512267802	0.114845893375308	1.14984095980043	0.250209381555184	0.680180899846129
Skiv2l2	1143.8145568296	0.152652725657233	0.132775561664969	1.14970498895286	0.250265398834815	0.680180899846129
Gmip	173.828117647241	-0.21451374651355	0.186668320009303	-1.14917060646852	0.250485638616848	0.680616726875233
Gbf1	1558.56012591539	0.10879148762172	0.0946895755764429	1.14892781976716	0.250585745143623	0.680725998877252
Zbed4	520.594967005861	-0.136863638048619	0.119148020319829	-1.14868579168362	0.250685566672154	0.680834444634578
Rrp7a	834.350949079549	0.220005082221738	0.191554726748271	1.1485233800096	0.250752566946221	0.680853720953355
Kbtbd3	157.826375208834	-0.242444518718029	0.211186796048863	-1.14800983420353	0.250964504098538	0.68110376160076
Pacs1	430.264701470968	0.177515749716699	0.154625417377008	1.14803731965929	0.250953157856805	0.68110376160076
Klf13	12.9650687783809	0.771717148827523	0.672394696863637	1.14771450820057	0.251086439902278	0.681109421462839
Mtx2	867.922391490197	0.114265913074987	0.0995557741672519	1.14775776724936	0.251068576287352	0.681109421462839
Dact3	23.17125768252	-0.727289036155801	0.634596655716278	-1.14606503139368	0.251768245139375	0.681532363352598
Dio1	8859.74604663083	0.206675961248734	0.180162251211602	1.14716573454664	0.251313130323572	0.681532363352598
Eif4e2	337.305014474393	0.187101651167874	0.163193286921073	1.14650335622178	0.251586939327203	0.681532363352598
Gstp1	10042.3151908127	0.329951723527872	0.287959467971745	1.14582696603762	0.251866755110562	0.681532363352598
Haus7	918.923863740515	-0.22079870324788	0.192722799120078	-1.1456802425867	0.251927481752619	0.681532363352598
Klhl42	66.2245935007419	0.398659882527347	0.347763590578361	1.14635313565845	0.251649065317356	0.681532363352598
Nr3c1	1207.2624852589	-0.137646754952666	0.120168042948169	-1.14545224816581	0.252021865491023	0.681532363352598

Ogfod1	35.9853277761833	0.406584306720519	0.354698531254218	1.14628133723258	0.251678762423241	0.681532363352598
Pih1d2	126.859635003293	-0.346681317204959	0.302262476736605	-1.14695453086974	0.251400413534979	0.681532363352598
Plk1	53.3050880339749	0.442059232206556	0.385704324031762	1.14610909098897	0.251750016498579	0.681532363352598
Vangl1	290.746406046739	0.161600317884488	0.140990278825987	1.14618056812228	0.251720446442007	0.681532363352598
Zbtb17	519.22292520828	-0.158378545574717	0.138258470666875	-1.14552507930108	0.251991712622691	0.681532363352598
Zdhhc24	72.4888487615472	0.339342343464217	0.296202202876225	1.14564422603575	0.251942390024986	0.681532363352598
Mrpl38	897.241286768649	0.150496185994181	0.131460945724324	1.14479768242178	0.25229297561216	0.682103225691677
Il17rc	305.548765153691	0.251591113151933	0.219832025841023	1.14446979319509	0.25242885821244	0.682146077613551
Stx1a	80.9462430231539	-0.375515844064502	0.328088241964131	-1.14455745751948	0.25239252370335	0.682146077613551
Hspa1a	964.829036128087	-0.480589044776485	0.420052636152915	-1.14411624499729	0.252575431449145	0.682379927228663
Atp5l	7687.14321764702	-0.19297451706643	0.168846239231717	-1.14290088985399	0.253079743061991	0.683488665645619
Ripk2	126.52574127746	-0.303179884913937	0.265286971141789	-1.14283744734639	0.253106087779554	0.683488665645619
Selo	1756.90736374149	0.169240907686131	0.148115777006217	1.14262579656877	0.253193990302702	0.683563632726962
RGD1560220	885.513582440027	0.200906249536318	0.17587846160536	1.14230160818166	0.253328673021557	0.683764829627882
Chic2	118.142294579547	-0.243251489528988	0.213044737358027	-1.14178595794271	0.253543000550414	0.68393760139253
Nr2c1	178.987133942201	-0.23111164723304	0.202392068994945	-1.14190070974971	0.253495293597812	0.68393760139253
Tmeff1	39.145540597696	-0.574247931171704	0.502970338373287	-1.1417133126159	0.253573205372877	0.68393760139253
Wdr83	312.494013239703	-0.169138417474795	0.148175450617204	-1.14147395381808	0.253672744848824	0.684043752776213
Shroom3	674.384159870455	-0.289883254172939	0.254225091236748	-1.14026216988544	0.254177093388413	0.684591669064623
Tob2	477.777376928323	0.309535115288524	0.271439256223181	1.14034764018813	0.254141497505316	0.684591669064623
Tpra1	841.304274339067	-0.112603229135042	0.0987418859814581	-1.14037956654166	0.254128202002129	0.684591669064623
Wipi2	523.02249776572	0.125489738444149	0.110042119986207	1.14037914263991	0.254128378529831	0.684591669064623
Zbtb49	80.6862411371215	-0.27316725102638	0.239447126169963	-1.14082493031252	0.25394278389453	0.684591669064623
Ccnd1	5644.32582520146	0.184517908022867	0.16203343373265	1.13876441282677	0.254801428572705	0.684909404164715
Fam188a	2537.1798064922	0.233985663505114	0.205468120363644	1.13879303071931	0.254789489310739	0.684909404164715
Itpka	22.4556566189652	0.601851022765952	0.528630348729548	1.13851015972196	0.254907518961987	0.684909404164715
Krt76	58.1268253865092	0.840704274819105	0.738212771614122	1.13883734764014	0.254771001249338	0.684909404164715
Map3k6	93.781679677539	-0.502428245943323	0.441107753605383	-1.13901476869708	0.254696994364659	0.684909404164715
Muc20	613.210666776222	-0.196315193064648	0.17232171152431	-1.13923655544097	0.254604502442002	0.684909404164715
Rabggtb	1941.23817264729	-0.151563495820773	0.133130466324015	-1.13845838601583	0.254929125972964	0.684909404164715
RGD1305464	105.970455582856	-0.327237341068496	0.287456419093937	-1.13838940212207	0.254957917387024	0.684909404164715
RGD1566320	371.26002149775	-0.230909888280237	0.20283371283074	-1.13841966928311	0.254945284676354	0.684909404164715
Rsbn1	380.970374320342	-0.177731661060358	0.155983701270793	-1.1394245655949	0.25452611470053	0.684909404164715
Triobp	704.944172377663	0.112967003974553	0.0991832366299638	1.13897275197838	0.25471451926695	0.684909404164715
Btbd10	246.214251422101	-0.179732531527139	0.157972153622403	-1.13774818792905	0.25522564548799	0.685181207449646
Gtf2ird2	112.079139368902	-0.263488522486536	0.231629886664713	-1.13754112770404	0.255312141703115	0.685181207449646
Pced1a	356.258703586061	-0.208811010946359	0.183582166733718	-1.1374253537885	0.255360513351812	0.685181207449646
Sar1b	1519.82787509247	0.128389349559152	0.11283623627718	1.13783793039469	0.255188163286244	0.685181207449646
Tpm4	1298.09806174127	-0.161932673666262	0.142365486349305	-1.13744333559152	0.255352999933682	0.685181207449646
Bcl2l15	20.5915298464697	0.743901439057269	0.655390852864514	1.13505007859951	0.25635433644954	0.685411542714164
Clic1	4027.8016634771	0.133761997750054	0.117792022116045	1.13557773563201	0.256133331227616	0.685411542714164
Cnep1r1	207.265108310616	-0.195889501242063	0.172527445087473	-1.13541066549004	0.256203292990201	0.685411542714164
Dcaf15	455.158363729017	0.16897396775168	0.148679765074622	1.13649606364977	0.255749012055973	0.685411542714164
Ehhadh	3650.53082623256	0.232739706269306	0.204810787638262	1.13636449013795	0.255804050805888	0.685411542714164
G4	384.150979183477	-0.148915233813149	0.131097528858556	-1.13591182922919	0.255993467058913	0.685411542714164
Galnt14	885.350058277381	0.218802591111318	0.192815971561659	1.13477420640618	0.256469936171783	0.685411542714164
LOC362855	2672.16798302834	0.126477103010946	0.111470247891366	1.13462655195856	0.256531823218903	0.685411542714164
LOC500625	28.0218804635142	0.557884428461581	0.490988488202344	1.13624747191968	0.255853007819539	0.685411542714164
Mansc1	67.2817865873375	-0.492448690790877	0.433804827571964	-1.13518490226849	0.256297853966356	0.685411542714164
Mien1	1387.243438872	0.145875519404531	0.128553896442575	1.1347421077174	0.256483388951988	0.685411542714164
Peli1	294.166448365107	0.195725370064592	0.172501123509124	1.134632436491	0.256529356610993	0.685411542714164
Rc3h2	85.5375512770575	-0.292383722855384	0.257358634921732	-1.13609447355168	0.255917027699702	0.685411542714164
RGD1309139	100.032043580487	-0.277152883062451	0.243759543153638	-1.13699295410873	0.255541231145729	0.685411542714164
Tmprss3	13.7047394920398	-1.39564882059305	1.22840867131207	-1.13614373879529	0.255896412180111	0.685411542714164
Ubac2	1427.59420075421	-0.126005562678789	0.110914327999858	-1.13606208459335	0.255930581802512	0.685411542714164
Wrnip1	515.032020805596	-0.144704854570953	0.127460946521551	-1.13528777653072	0.256254762052676	0.685411542714164
Zfp418	85.4879765639988	-0.322906293489964	0.284487095898612	-1.13504724166837	0.256355525034871	0.685411542714164
Rnf43	110.004131597773	-0.334040701533152	0.29449006776805	-1.1343020974013	0.256667849686798	0.685613814228002
Phtf1	719.978511521756	-0.146595362718304	0.129265156056183	-1.13406711592556	0.256766396132934	0.685715897191479
Hook1	1224.7675724687	0.22587093246466	0.199247142820544	1.13362193940264	0.256953166116902	0.685731483763207
Stxbp4	134.68090657953	0.245861034236242	0.21688100562299	1.13362179195918	0.256953227991158	0.685731483763207
Vom2r34	51.1316251205571	-0.458976058433809	0.404849080940751	-1.13369667869143	0.256921803305488	0.685731483763207
Solh	373.402129766754	-0.14908309911095	0.131547397062738	-1.13330329934122	0.25708690670654	0.685927178785571
Mast3	1464.53529593135	-0.153088147609932	0.135109984666096	-1.13306317063292	0.257187726186465	0.686035131620597
Bop1	485.147667286402	0.168834231081115	0.149043998931907	1.13278114040841	0.257306173303043	0.686190043585731
Dqx1	773.664032657082	-0.162356233596225	0.143357692934014	-1.13252543531763	0.25741359709715	0.686253544381841
Tpgs1	743.225678984656	0.173429565980739	0.153147203736919	1.132437039325	0.257450740211523	0.686253544381841
Cpz	33.0617268188871	-0.767476326208101	0.677932292662833	-1.13208403628857	0.257599105640435	0.686488026895471
Bcl2l1	260.598417838233	0.17051415920264	0.150720493833656	1.13132696732555	0.257917497960204	0.687059909817205
Galc	293.647484106882	0.280336396884937	0.247834692952003	1.1311426723426	0.257995046199176	0.687059909817205
Vps26a	786.609586264381	0.182508423719038	0.161331446560967	1.13126379022498	0.257944080003748	0.687059909817205
Gpr162	135.718537433175	0.390453694902182	0.345359100394376	1.13057305991448	0.258234831771019	0.687215429152282
Rassf1	270.937162316704	-0.233964343725308	0.206918457865516	-1.13070794234012	0.258178037346903	0.687215429152282
Tox3	129.336508770428	-0.387309493182395	0.342509080102985	-1.13080065809041	0.258139002905556	0.687215429152282
Ubqln1	1556.30786663919	0.134797714253205	0.119249934008783	1.1303797806989	0.258316230205113	0.687271131205833
Cd2bp2	1466.01084378421	-0.119955684826442	0.106138639174188	-1.13017922369985	0.258400712438978	0.687335009965229
Fem1b	123.195863663004	0.32473009171686	0.287414710390059	1.12983114634654	0.258547381318292	0.687540261995112
Spp2	1169.18078277434	0.719386355980906	0.636789073533589	1.12970901336133	0.258598858000097	0.687540261995112
Ache	19.4755876922326	-0.598666860721103	0.530614319569376	-1.1282523645554	0.259213354793998	0.68796476016828
Akt1s1	1126.34376139715	0.12985906997156	0.11507659009366	1.12845775031975	0.259126650269495	0.68796476016828
Ctdsp1	4462.50368299947	0.105903999747934	0.0939189611268212	1.12761042581092	0.259484481678953	0.68796476016828
Dnajc10	2035.65777021431	-0.143300957785514	0.127045108058816	-1.12795337006736	0.259339612581234	0.68796476016828
Dohh	1124.99347473124	0.182826072358061	0.162007491147982	1.12850381832691	0.259107205211924	0.68796476016828
Dvl3	266.979871229618	-0.228511745975905	0.202651485427393	-1.12760952871366	0.259484860711017	0.68796476016828
Enpp4	483.935630624008	-0.315974740835139	0.279846136735531	-1.129101671801	0.258854946163331	0.68796476016828
Itgb4	278.358614072605	-0.289180423382603	0.256365764734314	-1.12799937886518	0.259320181460703	0.68796476016828
Rpl30	5949.54188176836	-0.178253457582731	0.158073472323544	-1.12766206095532	0.259462665989024	0.68796476016828
Ss18l1	83.4604068104407	0.337381723635055	0.299003202286066	1.12835488401315	0.259170073231955	0.68796476016828
Synj2bp	4458.30334962722	0.154405481122195	0.136874708923798	1.12807897336357	0.259286568308306	0.68796476016828
Tcf3	466.491341553065	0.172254449598955	0.152736848340163	1.12778580591976	0.259410389291294	0.68796476016828
Csnk1g3	818.761968397275	0.173242432867211	0.153692930976584	1.12719844540935	0.259658587633449	0.688104338317039
Zfp148	212.713951457657	0.187138433677617	0.166007208780612	1.12729100773527	0.259619463068781	0.688104338317039
Gtf2a1	59.1244099467392	0.429126399213421	0.380879130415042	1.12667343770136	0.259880576956561	0.688116571234235
RGD1565712	2172.15292930164	-0.148649647506697	0.131929996323973	-1.12673123359805	0.259856132649412	0.688116571234235
Rpap1	735.573046063986	0.133559404728083	0.118554277265215	1.12656757570459	0.259925354560085	0.688116571234235
Rrad	135.67261052624	-0.480716138902944	0.426715697084279	-1.12654899312973	0.259933215185475	0.688116571234235
Shroom4	128.700544440957	0.355483693695784	0.315572679898935	1.12647170157325	0.259965912095707	0.688116571234235
Nsun4	247.909841765604	0.171376401219404	0.152179332685076	1.1261476719316	0.260103018444256	0.688319186877162
Hmox1	96.1279707329372	0.550057412263398	0.488668627112425	1.12562456794848	0.260324464526246	0.688520635688147
Ist1	1669.94123658815	-0.126278626105989	0.112180144129959	-1.12567716047589	0.260302194586879	0.688520635688147
Slc1a5	475.594585463031	-0.346982202453358	0.30828103570469	-1.12553859065707	0.260360873850753	0.688520635688147
Bre	972.454520764926	0.160869608842595	0.143020891924449	1.12479796956919	0.260674655062843	0.688795916073645
Eral1	513.20755205049	0.17297752225792	0.15375583420069	1.12501436551761	0.260582946887434	0.688795916073645
Fchsd2	963.84338733415	-0.160404984173895	0.142633728429805	-1.12459364232939	0.260761269035113	0.688795916073645
Ncoa2	164.289234056357	0.345763703998449	0.307460895922236	1.12457781976249	0.26076797702489	0.688795916073645

Rrp8	91.1676447903913	-0.251109192967445	0.223256801385814	-1.12475495218396	0.260692888405745	0.688795916073645
Orai1	443.555441852385	0.231798735026449	0.206157962385561	1.12437439885506	0.260854228111626	0.688863651653518
Gmnn	53.1985593180175	0.354570522358833	0.315505126705887	1.1238185764549	0.261089999115883	0.689006020420507
Grem1	2427.53570611401	-0.312056887163998	0.277661435728497	-1.1238755081175	0.261065842840249	0.689006020420507
Iars	745.641677125527	0.119551319862748	0.106353467765947	1.12409423382269	0.260973051241043	0.689006020420507
Dpf2	934.598703861817	-0.10198210043548	0.0907974292852881	-1.12318268521733	0.261359914726438	0.689078355551077
Dynlt3	1187.35330759061	0.148018486702917	0.131770083202391	1.1233087443344	0.261306391314757	0.689078355551077
Pcyox1l	251.174292747472	0.224949254469838	0.200219946439845	1.12351071144364	0.261220653943773	0.689078355551077
Xrcc5	474.262241847323	-0.149990100218026	0.133530880172216	-1.12326152590758	0.261326438888079	0.689078355551077
Gal3st3	36.3348850860597	-0.444733792443929	0.396071654726796	-1.12286195474074	0.26149612777813	0.689136120828339
Trpm5	16.3796105926583	-0.643677966616162	0.573256005304469	-1.12284557101899	0.261503087201533	0.689136120828339
Trappc6b	777.956883891316	-0.158542138318957	0.141254293205503	-1.12238810390211	0.261697460275535	0.689488487132993
Eif3m	2232.0451660722	-0.116045346658201	0.103422339356118	-1.12205300499553	0.261839903699193	0.689703903927005
Abl1	949.637092795508	-0.143346739740768	0.127932204717455	-1.12048987240825	0.26250506595824	0.690048307144722
Cenpc1	190.798604631847	-0.204139594047885	0.18229870437731	-1.11980825505687	0.262795480722224	0.690048307144722
Dak	10085.9726941216	0.197881720203486	0.176670056969467	1.12006371423588	0.262686611978453	0.690048307144722
Fbxo21	1473.60306206015	-0.164676997471812	0.146926606884194	-1.12081127417316	0.262368204147047	0.690048307144722
Grip2	10.7663202286114	0.962481983315383	0.859418012338651	1.11992298217753	0.262746583744448	0.690048307144722
Limk1	106.117812562138	-0.260134732632228	0.232335854978898	-1.11964953776013	0.262863136777443	0.690048307144722
March9	176.835763939898	-0.231435994465573	0.20671046695437	-1.119614298567	0.262878159738019	0.690048307144722
Pex6	800.869020357964	0.147664651228533	0.131730292906412	1.12096198961192	0.262304042298529	0.690048307144722
Ppp2ca	6263.2103638649	-0.163644688773781	0.145963934165169	-1.12113098149715	0.262232112779757	0.690048307144722
Psme2	1455.10657595539	-0.148338086881904	0.132358605499908	-1.12072869249146	0.262403365018379	0.690048307144722
Pxdn	316.895805955919	-0.413009271033328	0.368615775403805	-1.1204329781624	0.262529298246107	0.690048307144722
Rtfdc1	1417.92459405667	0.0999495120597965	0.0892747091110651	1.1195725312916	0.262895966478916	0.690048307144722
Slc38a9	74.187379112141	-0.291843976686902	0.260699632575444	-1.11946447259547	0.262942039261287	0.690048307144722
Tubg1	836.319650019521	0.124429702857333	0.111088100206691	1.12009929619661	0.262671450526567	0.690048307144722
Ubl7	882.047369689958	0.114951358579658	0.102575786965238	1.12064807866026	0.262437691175315	0.690048307144722
Usp16	1012.06023807976	-0.137638987806197	0.122798112415859	-1.12085589182412	0.262349208606772	0.690048307144722
Kpna5	53.3638076271655	-0.399954896366557	0.357414274160982	-1.1190232883268	0.263130203999911	0.690382709755999
Atp6v0c	11855.0236026297	0.158180983811807	0.141380360141242	1.11883279724129	0.263211477016606	0.69043656768307
Ddost	2604.27580854799	0.123180214967273	0.110165152708087	1.11814137174277	0.263506619247751	0.691051276965838
Plod3	800.311260713478	-0.149501756675783	0.133741656927484	-1.11783987211139	0.263635388997838	0.691100610502321
Vars2	610.251023602288	0.111088209138855	0.0993799937299887	1.117812599593	0.263647039164003	0.691100610502321
Rrnad1	172.910955534308	-0.223839948516935	0.200335066437437	-1.11732784727849	0.263854172996138	0.691415685016511
Tbxa2r	71.9081377543031	-0.367972116566371	0.329398174067147	-1.11710429970799	0.263949732296907	0.691415685016511
Wipf1	164.082637897766	-0.342295405498158	0.306411470797444	-1.11711028509254	0.263947173429131	0.691415685016511
Fahd2a	3692.86145810828	0.179317172043127	0.160581495471648	1.11667394500499	0.264133762106435	0.691738327212383
Dpy19l3	2041.92632395918	0.174431562778024	0.156307406143795	1.11595200177243	0.264442680713909	0.691768489338867
Gnb4	15.4216191489544	-0.751016597915961	0.672870200153318	-1.11613888940963	0.264362687844758	0.691768489338867
Snapc1	194.110491875356	-0.240167954656238	0.215176892493733	-1.11614194197564	0.26436138140397	0.691768489338867
Tecpr1	921.33418946214	-0.143899309339097	0.128949445519789	-1.11593585190728	0.264449594068043	0.691768489338867
Tjap1	569.766256177455	-0.132901690416461	0.119059988592929	-1.11625821560304	0.264311621794019	0.691768489338867
Zfp867	69.5805207964275	-0.335496854548826	0.300680052430927	-1.11579352150704	0.264510527547301	0.691768673746578
Itsn1	173.979108189897	0.198809062999533	0.178279032359357	1.11515673138047	0.264783264122214	0.692120128596805
Tcta	951.915781097283	0.148870192701768	0.13352650538238	1.11491117269525	0.264888488421265	0.692120128596805
Tmem176b	127.04863848688	-0.548394515967582	0.491774084317834	-1.11513504565473	0.264792555508034	0.692120128596805
Top1mt	404.322134679026	0.145108543827205	0.130150204177712	1.11493135753417	0.264879837933506	0.692120128596805
Itch	331.93221032176	0.271120634055017	0.243239451499171	1.11462442619404	0.265011398568636	0.692282131953831
Atg16l2	162.908630673333	-0.266432010296433	0.239126208084757	-1.11418991849692	0.265197719502145	0.692609669085794
Gstm2	974.21769325749	0.216079106946463	0.19398487400849	1.11389667906275	0.265323514284691	0.692779017541878
Pnpo	2545.0054336808	0.190780398652861	0.171361817539154	1.11331918272441	0.265571370658532	0.69326692671219
Pold2	298.707596773926	0.20500844306876	0.184214663077219	1.11287798508648	0.265760836235128	0.693374645947207
Prmt10	349.590508127381	0.139404097853	0.125245021041767	1.1130510154692	0.26568651989415	0.693374645947207
Tmed9	2137.84293355133	-0.116437838995622	0.104635297134754	-1.11279694504684	0.265795647755761	0.693374645947207
Zkscan3	538.690941175308	-0.131805074604116	0.11846134852116	-1.11264202416683	0.265862204232194	0.693389126503698
Pde3a	18.1631140669472	-0.898821989209059	0.807993981481727	-1.11241173796959	0.265961160052256	0.693488081017194
Ngfr	13.891114933994	-0.8552680322701	0.769586624925081	-1.11133432490899	0.266424469864985	0.694059253835759
Rab3gap2	476.948034321707	-0.135704481108025	0.12210647357234	-1.11136188883244	0.266412609891752	0.694059253835759
Ralgds	147.263231470734	-0.319340000783898	0.287268233347301	-1.11164397491115	0.266291257149188	0.694059253835759
Tysnd1	765.973219275876	0.164578430035303	0.148087919697104	1.11135621576648	0.266415050821185	0.694059253835759
Tns4	119.813704092697	0.388955993665287	0.350098453618123	1.11099032185257	0.266572515169174	0.694285794549229
Cnr1	61.3470337009935	-0.654986110869052	0.589673533928167	-1.11076057035458	0.266671422642557	0.694368494094197
Evi5	1184.81270538592	0.122093544187533	0.109952946445058	1.11041630201825	0.266819676597045	0.694368494094197
Flot2	126.539483606186	-0.345666692443259	0.311396238425357	-1.11005416825585	0.266975685201569	0.694368494094197
Insr	305.542182585799	0.287392682527452	0.258930126801956	1.1099236928396	0.26703190987909	0.694368494094197
Psmd9	646.574037924382	0.198542711722009	0.178867429274904	1.10999924651942	0.266999351172711	0.694368494094197
Rpe	404.956052302033	-0.164731637198363	0.148382976523268	-1.11017881604856	0.266921979378302	0.694368494094197
Ssb	2201.74296499844	-0.175576228871313	0.158133087895091	-1.11030671195009	0.26686688179727	0.694368494094197
F11r	3211.05042909991	0.14319363181967	0.1290644316449	1.10947400453166	0.267225752689627	0.694477708481557
G6pd	1573.61483526414	0.141572765682091	0.12761189206214	1.10940103931029	0.267257214226494	0.694477708481557
Vps37a	296.310075813285	0.180912366039899	0.163061689118901	1.10947192450571	0.267226649531143	0.694477708481557
Adck3	1319.18815122832	0.18483415858571	0.16667617712813	1.10894167223202	0.267455345087464	0.69451621024997
Ier3	1249.65014690343	-0.248134359770415	0.223753426963131	-1.10896339393855	0.267445973965321	0.69451621024997
Pcmtd2	1107.37689212026	-0.149825154873561	0.135105439133716	-1.10894983824653	0.26745182210024	0.69451621024997
Brf2	194.616296059179	-0.25211629714643	0.227418956602781	-1.10859842518224	0.267603457804828	0.694742097169866
Lrrc61	173.23891408868	-0.198850837512387	0.179418983331655	-1.10830433781253	0.267730402727758	0.694754282512259
Pianp	27.4866573493455	0.571743927444574	0.515821776238672	1.10841370756714	0.267683187655384	0.694754282512259
Aldob	91473.7972101049	0.216596300711489	0.195456122962382	1.10815817600749	0.267793509914016	0.694759423346886
Acaa2	2100.85835605641	0.676024157175461	0.610153853461366	1.10795687569687	0.267880440573476	0.694826354988164
Sec61a1	3116.40296372677	0.0924058607183459	0.0834187651283668	1.10773469945461	0.267976408923924	0.694916692637308
Lrp11	698.226623228089	0.170068113013559	0.153565674301992	1.10746176700344	0.26809433359712	0.695063913244722
Oas1f	12.6320574679254	0.765852161748132	0.691770186461332	1.1070904423704	0.268254827373331	0.695162874583967
Tmem39a	178.32275815425	-0.216377615334593	0.195436417373644	-1.10715095089424	0.268228669904259	0.695162874583967
Brox	1430.76116185345	-0.102906783886326	0.0930144436062743	-1.10635273293603	0.268573875622569	0.695513937189821
Pde6d	380.285098540051	0.147153354050016	0.133003989636389	1.10638300739931	0.26856077724626	0.695513937189821
Suv420h2	744.326112346873	-0.215283630361226	0.194572673443882	-1.10644329725632	0.268534693888002	0.695513937189821
Itpa	1437.03826361167	0.129706894120129	0.117258960434243	1.10615763298419	0.268658296976948	0.695574078232345
Cotl1	4560.8303651924	0.173955593429761	0.157293516650144	1.10592983826967	0.268756888681304	0.695670871498907
Arhgef19	188.142020435434	0.305387300791497	0.276259099091877	1.10543798121173	0.268969853788667	0.695679709859318
Hccs	697.680519581714	0.18899037942436	0.171000572773975	1.10520319527914	0.269071552641221	0.695679709859318
Msrb2	1153.81452804495	0.156240318260369	0.141320997002676	1.10557045006844	0.26891248580131	0.695679709859318
Plin5	37.9735970195126	0.509758288398538	0.461288845652795	1.10507395355973	0.269127545684623	0.695679709859318
Snx27	609.473277600022	0.137039624161518	0.123997918259376	1.10517681333054	0.269082981780664	0.695679709859318
Trim11	508.223817087151	-0.131660782651231	0.119085627732024	-1.10559758686837	0.268900734765696	0.695679709859318
Plekhb1	198.414880066975	-0.386411244825775	0.349764603032913	-1.10477515870699	0.269257027019362	0.695697969789059
Zfp521	26.7856594811852	-0.584041275198469	0.528650118339358	-1.10477848190616	0.269255586691657	0.695697969789059
Brd1	1052.69144863194	-0.156663091106628	0.141869282999638	-1.10427773929772	0.269472676187026	0.696027588256515
Cul1	2059.02891184969	0.130484905215433	0.11817969366454	1.10412289259966	0.26953983197147	0.696027588256515
Vps35	2700.48997195042	0.134532581206282	0.121852905410885	1.10405722992522	0.26956831281939	0.696027588256515
Il1rap	17.0885012104021	-0.642858647565279	0.582530916073418	-1.10356142451375	0.26978343253035	0.696266688042678
Rnf181	3600.13217184126	0.145547141724401	0.131882507022538	1.10361218489369	0.269761403241978	0.696266688042678
Asna1	1532.4675574352	0.115992552183673	0.105124555426398	1.10338209482259	0.269861268966085	0.696297616672805
Ctsf	785.79470285957	0.174298948886834	0.157986588368786	1.10325155246704	0.269917939385921	0.696297616672805
Abcg2	3681.57337247246	0.21104799324399	0.191447272116507	1.1023818250884	0.270295709608608	0.696481758198014

Dda1	806.70191192853	0.10956733296387	0.0993708736187286	1.10261014091779	0.270196504467505	0.696481758198014
Exoc6	1495.00369002495	0.186210442454441	0.168897851804636	1.10250332058592	0.270242915688271	0.696481758198014
Fggy	822.206361614374	0.179714932509509	0.163010942914152	1.10247158440249	0.270256705455598	0.696481758198014
Lypd1	81.878581950818	0.321950842648682	0.291926573572881	1.10284870167294	0.270092874497261	0.696481758198014
Map3k3	664.837840205771	-0.15031390933067	0.136392466352409	-1.10206900242049	0.27043167422668	0.696674163476982
Oplah	10332.27592362	-0.134904955627189	0.122447345337274	-1.10173850854505	0.270575370352579	0.696886394613056
Eif4h	4646.4286245674	0.105342630946627	0.095641879473894	1.10142786325504	0.270710484182151	0.696918542064401
Hadha	7451.48675948318	0.153380345979143	0.139248818497392	1.10148400276743	0.2706860631204	0.696918542064401
Erbb2	1112.81342053337	-0.258274718217709	0.234568379677005	-1.10106365816803	0.27086895249757	0.697168594675585
Cby1	172.140238111358	0.190348407597168	0.172907706662336	1.10086711154461	0.270954497786505	0.697230885633102
Glud1	21117.0436214411	0.190109715858055	0.172733056984015	1.10059834045343	0.271071508141614	0.697374097224443
Gimap4	52.2011313715296	-0.561751092292417	0.510510316382036	-1.10037167568625	0.271170214281368	0.6974701641824
Hap1	15.9083865621462	-0.564655809474277	0.513645928656701	-1.09930942303189	0.271633123571849	0.697871175975957
Lbr	159.684827932413	0.254322027458519	0.231319626975368	1.09943989960524	0.271576235253388	0.697871175975957
Mmp11	299.481607472894	-0.232952362222471	0.21187584190994	-1.09947580678636	0.271560581007722	0.697871175975957
Ppp2r3c	405.978189529136	0.161254122997674	0.146667951786923	1.09945029594428	0.27157170275814	0.697871175975957
Rnf180	32.9466086893062	-0.430405063618221	0.391416521681416	-1.09960883043291	0.271502592845022	0.697871175975957
Elac2	725.083587722669	0.12443658364688	0.113275188861239	1.09853344671368	0.27197162099985	0.698582925262335
Rpl23a	6145.96059024488	-0.180614161646195	0.164435717119366	-1.09838765452085	0.272035250877222	0.698588491068799
LOC690344	977.760474600673	0.195239908123827	0.177805273952802	1.09805465149281	0.272180625698436	0.698803928549451
Dnajc8	1361.50553922291	-0.133866757171695	0.121982149913219	-1.09742906865416	0.272453872060136	0.69911066084437
Lmo7	1285.27090479407	-0.196674447782607	0.179188920073873	-1.09758152290624	0.272387264753875	0.69911066084437
Tspan4	429.255723850529	-0.311465655195675	0.283832130141687	-1.09735869240806	0.272484623221939	0.69911066084437
Hrasls	88.9113165556934	0.714155049932291	0.650978313475397	1.09704891107602	0.272620011559688	0.69916483985856
Rps2	13862.1023239589	0.171880604602824	0.156678285919521	1.09702888051195	0.272628767398657	0.69916483985856
Mrpl27	1674.87376075449	0.148989897960338	0.1358381076789	1.09681959286805	0.272720263544511	0.699241713387527
Cacnb1	92.3020746717492	-0.332476131505373	0.303280989875282	-1.09626433111451	0.272963114072594	0.699493533639135
Hsd17b12	2027.2282105626	-0.143115387517046	0.130538333533026	-1.09634759111459	0.272926689868184	0.699493533639135
Tango2	572.078563458679	0.129391496621877	0.118039311144781	1.09617292211382	0.273003107093366	0.699493533639135
Casp8	101.228712780924	-0.383619646708077	0.350225867559137	-1.09534926526608	0.273363651980334	0.69978632171362
Kcnb1	140.484124177498	0.268139111189493	0.244774100953676	1.09545540212295	0.273317173711101	0.69978632171362
Mettl14	651.796565348206	-0.14750679359157	0.134665176339641	-1.09535959927414	0.273359126389269	0.69978632171362
RGD1559482	20.6205295534612	-0.945935642756095	0.863460200462774	-1.09551736403035	0.273290042541437	0.69978632171362
Dcaf11	2081.38438645265	0.116754189272948	0.106636797679978	1.09487711383957	0.273570476758654	0.699792906297886
Npdc1	968.162014062089	0.121934545932388	0.111363779248315	1.09492104843625	0.273551226803378	0.699792906297886
Rnf144b	260.122702063905	-0.201570012036393	0.18408863202258	-1.09496175739776	0.273533390987527	0.699792906297886
Svip	303.032086573583	0.197138681134265	0.180071304882188	1.0947812104946	0.273612500051716	0.699792906297886
Kpna1	156.272550143126	0.215782677298685	0.197154617629505	1.09448452130189	0.273742532392427	0.699810531527739
RGD1564541	468.289825955251	-0.138782028479919	0.126797460121255	-1.09451741657288	0.273728113037871	0.699810531527739
Camta2	1546.95106214965	-0.133861273224111	0.12235738886522	-1.0940187140767	0.273946770644849	0.700175173184024
Lin7c	100.398940826917	0.311112945135306	0.28444603564123	1.09375032924597	0.274064494169124	0.700318579299969
Ceacam1	82.358535770976	-0.388135017618547	0.35491525640635	-1.09359913560369	0.27413082851841	0.70033063541026
Ccdc23	73.874819272648	-0.274632841650292	0.251302548039547	-1.09283747336725	0.274465165268747	0.700397635483739
Fam126b	203.38765807576	-0.192497065299353	0.176122700638751	-1.09297134668737	0.274406380510763	0.700397635483739
Hmgcl	2334.99433064603	0.214772595987853	0.196506226504997	1.09295567783137	0.274413260376082	0.700397635483739
Pqlc2	48.0235064651063	0.383743591834304	0.35103084900323	1.09319050711345	0.27431016412319	0.700397635483739
Tom1	1213.90750153287	0.0984732767723624	0.0900681443201552	1.09331970271676	0.274253455127859	0.700397635483739
Fgf13	205.171277809441	0.312954973485323	0.286427781873524	1.09261389184486	0.274563360734434	0.700430659625845
H2afx	594.584172285324	0.224849738847751	0.2058069574916	1.09252739357429	0.274601356615587	0.700430659625845
Gas2l1	298.487422547578	0.178873765647788	0.163818696803405	1.09190079727255	0.274876707247813	0.70081845100463
Ube2i	1518.7553197439	-0.111059435298168	0.101711847720755	-1.09190264248346	0.274875896114169	0.70081845100463
RGD1563941	386.471358546097	0.189645554116111	0.173719297222118	1.09167811031166	0.274974609892611	0.700910835622205
Cand2	132.752545132285	-0.272299270119426	0.249479139379967	-1.09147109772855	0.275065642764334	0.700985671672515
Mafb	67.3063676590653	0.412499189776988	0.378112256943978	1.09094371367629	0.275297650539203	0.701279009505016
Med24	878.667540184053	-0.109915974484187	0.100793329553477	-1.09050841926866	0.275489246651407	0.701279009505016
Ppp1r9a	43.4500526687536	-0.382341146797605	0.350538830290787	-1.09072409033954	0.275394307026043	0.701279009505016
Ube2h	1806.64346747866	0.114134272769632	0.104655855219907	1.09056748454072	0.275463243564099	0.701279009505016
Wipi1	411.691159637393	0.136194463922195	0.124872787038241	1.09066568587508	0.275420014796819	0.701279009505016
RGD1306001	101.098474724597	0.246330462653652	0.225939878834579	1.09024783019381	0.275603989228731	0.701414003935011
Pdlim3	23.989956704087	0.922526073906897	0.846426338562071	1.08990709749664	0.275754069831308	0.701533485048333
Pole4	1118.12751771472	-0.108248588930035	0.0993232985198886	-1.08986099478321	0.275774380714055	0.701533485048333
Alkbh7	470.621723379085	0.204430201277962	0.187712998597338	1.08905724593151	0.276128642062007	0.702009276671049
E4f1	532.930818842394	-0.16028364195062	0.147182044165487	-1.08901627817047	0.276146707372537	0.702009276671049
Hmgn2	2532.70410120443	0.216064667894514	0.198360690418741	1.08925144109148	0.276043019939668	0.702009276671049
Asun	618.554494295443	-0.124082539852959	0.114094377294191	-1.0875429867417	0.276796911677487	0.702153532297063
Dnaja2	2887.27773341083	0.142442182003368	0.130923668427537	1.08797884839444	0.276604445271395	0.702153532297063
Fam160a1	181.983589197296	0.192588706812091	0.177092090176299	1.08750597850172	0.276813257861673	0.702153532297063
Mmp19	20.4465300819987	-1.07433819637181	0.987908134745811	-1.0874879541793	0.276821219270028	0.702153532297063
Npm1	4247.84508287823	-0.137420412901372	0.126301968172647	-1.0880306529628	0.276581575641697	0.702153532297063
Papd4	373.840192433594	0.203675673687372	0.18717316464588	1.08816706749984	0.276521360285933	0.702153532297063
Ppat	1371.10714147359	-0.246325710499015	0.226366589883199	-1.08817167156211	0.27651932814197	0.702153532297063
Ppp1r1b	2089.63754325801	0.172819781986641	0.158805885831045	1.08824544557817	0.276486767107963	0.702153532297063
Siah1a	358.515930693828	-0.156850447517024	0.144093537087213	-1.08853214854522	0.276360252183266	0.702153532297063
Zfp346	358.418867405218	-0.165826604958129	0.152447331087943	-1.08776325419871	0.276699635267448	0.702153532297063
Usp46	513.112744451916	0.174002012849594	0.160127431681472	1.08664712237265	0.277192791169681	0.702939148691343
P4hb	11468.4881626578	0.115050719418989	0.105905489694552	1.08635274479929	0.277322959804618	0.70311237143415
Mbd3	2874.18984511668	0.122893915386046	0.113146789906624	1.08614584194095	0.27741447355915	0.70318753489592
Glt8d1	294.314063066957	-0.187801240116767	0.172988721598925	-1.08562707661477	0.277644015454572	0.703612459232254
Decr1	4098.85895714521	0.252451340024491	0.232577716699024	1.0854493870158	0.277722668804642	0.703654893810423
Cyb5r1	771.114288424606	-0.145107792887849	0.13381770945115	-1.08436912784567	0.278201166249608	0.703925747015372
Kif2c	21.6912871067355	0.517335858353404	0.476946858605581	1.0846823897025	0.278062350150185	0.703925747015372
Klhdc1	29.563936891568	-0.409437798097978	0.377417254377112	-1.08484122903632	0.277991981506046	0.703925747015372
Oraov1	384.504613172678	-0.224675182295995	0.207186890661752	-1.08440829233155	0.278183808664831	0.703925747015372
Prepl	663.464758042246	0.137228799346403	0.126488636447041	1.08491010102602	0.277961473760521	0.703925747015372
Ralbp1	1161.11133520997	-0.140086135560161	0.129175816193888	-1.08446100584258	0.278160447355832	0.703925747015372
Slc41a3	90.9570945545657	-0.322417860471602	0.297431403795095	-1.08400745972917	0.278361491329954	0.704174651781941
Lrp10	3403.31119914521	0.158550351683368	0.146283693174584	1.08385526946017	0.278428975061433	0.704188636081052
Mst1	11.978264936299	0.988448077962205	0.912203784615955	1.08358252249342	0.278549943518813	0.704337854957693
Slc30a3	66.2969321744663	-0.339924627601448	0.313774555338674	-1.0833403213162	0.278657394312866	0.704452834466201
Espn	2940.91872454316	0.277506151181219	0.256209498737258	1.08312202533053	0.278754263902254	0.704541019237942
Traf3ip3	83.5492872921023	-0.404727283651281	0.373718776815383	-1.08297283615272	0.278820480286549	0.704551707189176
RT1-N1	74.9910236418035	-1.95224407395229	1.80441102312002	-1.08192870079936	0.279284210631848	0.705566645486017
Akr7a3	32184.0202679415	0.284624166490912	0.263154788261213	1.08158460034703	0.27943715030492	0.705719929089881
Pds5a	525.035321856984	0.225851126020516	0.208828895959627	1.08151280972237	0.279469065707685	0.705719929089881
Galnt11	3077.81493227174	0.0934775739400589	0.0864440171132904	1.08136545548954	0.279534581602614	0.705728577186874
Lrp4	405.550223518467	-0.155709008760406	0.144032667717796	-1.08106731082345	0.279667173111448	0.705906526667715
Nusap1	65.7381355225624	0.45497694321773	0.421026129240156	1.08063825881507	0.279858057017334	0.706231500013103
Chpf	1122.85477630806	0.115917030717679	0.107294657675341	1.08036162497884	0.279981177489745	0.706369575638634
Snx33	351.16593913742	-0.276329720971781	0.255804936685086	-1.08023607578755	0.280037067378822	0.706369575638634
Chfr	471.768482992538	0.150156777116174	0.139116260612876	1.079361797497	0.28042647416345	0.706567791031207
Dgat2	1739.93232116226	0.277970714953256	0.257432913434643	1.07977923741219	0.280240499022179	0.706567791031207
Pbld1	5104.89285606444	0.119622086514352	0.110821019940628	1.07941694254789	0.280401901491135	0.706567791031207
Prmt7	213.653019626206	0.172284359503242	0.159605779482037	1.0794368478532	0.280393032032769	0.706567791031207
Sft2d3	180.155608038726	0.246079772511418	0.227933968775079	1.07960991437062	0.280315924629566	0.706567791031207
Adipor2	267.955835223841	0.310844973495047	0.288326073476095	1.07810219779107	0.280988150939337	0.706704736856827

Aoah	248.473583240388	0.256296186774215	0.237614492315903	1.07862186466925	0.280756330222333	0.706704736856827
Apba3	877.998403344894	0.101704855149267	0.0944416666640051	1.07690661062878	0.28152198950368	0.706704736856827
Cd300le-ps1	16.4225445863737	-0.700145563904048	0.648887343727065	-1.07899402056845	0.28059039322036	0.706704736856827
Cox17	1271.04456072065	-0.192502839070219	0.178618262588519	-1.07773324116183	0.281152819452911	0.706704736856827
Gimap6	34.8891178527936	-0.687447588427819	0.637518606588678	-1.0783176856693	0.280892007141306	0.706704736856827
Hoxc9	54.9411532054029	0.400940120095358	0.371647773476168	1.07881749524612	0.280669094086875	0.706704736856827
Lmtk2	1943.60749701193	0.116655923533945	0.108173130607701	1.07841866902242	0.280846959286397	0.706704736856827
Luzp1	606.822318294047	-0.184738166576138	0.171347243888951	-1.07815079124276	0.280966468144938	0.706704736856827
Mtmr11	1099.90220908891	-0.152303682817738	0.141343607829551	-1.07754206331994	0.28123816952527	0.706704736856827
Nudcd3	123.908793667433	0.371700810576887	0.344947753651323	1.0775568376439	0.281231572999942	0.706704736856827
Pnp	2322.09605694559	0.10523013297208	0.0975706208944861	1.07850223773688	0.280809683670118	0.706704736856827
Ppm1l	217.430215801778	0.185526052975136	0.172220726929552	1.07725740265297	0.281365286965356	0.706704736856827
Rmnd5b	108.710582971305	-0.236816296179715	0.219911451463016	-1.07687114338173	0.281537836396948	0.706704736856827
Specc1	110.511091683427	-0.382788001953084	0.355379286626772	-1.07712524718723	0.281424315270233	0.706704736856827
Tollip	1904.76183318012	0.103872688341555	0.0964100364379967	1.07740534263108	0.281299218349084	0.706704736856827
Tsn	167.379937490103	0.351240448840758	0.325944555818652	1.07760796298184	0.281208747074815	0.706704736856827
Ankrd33b	203.986694953781	0.218317032831096	0.203066928321835	1.07509890771131	0.282330448459473	0.706835301987685
Ash1l	997.782630279071	-0.137703391294737	0.128102452374481	-1.07494734677045	0.282398302509773	0.706835301987685
Dmap1	394.281883408625	0.137980338245816	0.128341112283729	1.07510629906945	0.282327139620576	0.706835301987685
Hspbp1	1052.88755022501	0.116045544168645	0.107829385298698	1.07619591679195	0.281839645513393	0.706835301987685
Lims1	60.1426582598709	-0.396267531975618	0.368299760865801	-1.07593752177322	0.281955199559248	0.706835301987685
Lypla1	5559.02473499075	0.232628949845122	0.216252869904366	1.07572653231376	0.282049577705944	0.706835301987685
Map9	26.7516572218291	-0.430771095951507	0.400189106683878	-1.07641884488322	0.281739978066062	0.706835301987685
Ninj1	1534.44786638193	-0.168182924846638	0.15637806555672	-1.07548922700822	0.282155752847926	0.706835301987685
Pcmtd1	2434.78017210298	-0.118587213205095	0.110248551664186	-1.07563511189071	0.282090477823297	0.706835301987685
Top1	730.939085030884	0.159076927869082	0.147984832539291	1.07495427159298	0.282395202015786	0.706835301987685
Tsen2	119.617106987448	0.253876142929365	0.235978549604317	1.07584415344131	0.281996961705162	0.706835301987685
Upk3a	97.943406772695	0.4950690741435	0.459938286740871	1.07638152425093	0.281756661834333	0.706835301987685
Wdr75	400.265401046607	-0.172602059886885	0.160450953468601	-1.07573097046545	0.282047592246234	0.706835301987685
Ager	64.7151002448433	-0.305504648265247	0.284379602414085	-1.07428467327415	0.28269511223286	0.706905620330623
Cuta	369.849755256048	0.149564967933808	0.13926164531163	1.07398535755571	0.282829244256111	0.706905620330623
Ddx47	950.248560729868	-0.117691194864434	0.109645656429605	-1.07337763023922	0.283101717102617	0.706905620330623
Dennd1a	684.140943157788	0.146665319474729	0.136483241019767	1.07460314086099	0.282552445054295	0.706905620330623
Fam96b	449.781374881377	-0.184102010360081	0.171492068990935	-1.0735307553483	0.283033047132387	0.706905620330623
Helz	437.7554103653	0.149292211652401	0.139017090042439	1.07391265064479	0.282861832834036	0.706905620330623
Mtfr1l	2235.09969345977	0.0963480222262105	0.0897367810710955	1.07367370520987	0.28296895050655	0.706905620330623
Nsmce4a	452.128755750197	0.161030788449153	0.150015568773976	1.07342717669373	0.283079496431392	0.706905620330623
Taok1	359.164085013033	-0.27277486007864	0.254020655381496	-1.07382944772337	0.282899129039552	0.706905620330623
Tardbp	2748.03572788444	-0.157583539509475	0.146710996538516	-1.07410857554978	0.282774021489853	0.706905620330623
Tnxa-ps1	611.393680317801	-0.247425280911355	0.230515158903434	-1.07335796087495	0.283110538777494	0.706905620330623
Slc7a1	265.826475496058	0.208989541155816	0.194764705165605	1.0730360050509	0.283254961866471	0.707110893163697
Mprip	1475.79600401835	-0.144368865351938	0.13457737916559	-1.0727572958179	0.283380025678008	0.707267758422539
Elavl1	1070.69549112711	-0.169390012348705	0.157932337848503	-1.07254799527626	0.283473968659271	0.707346899622674
Alg1	472.612091779069	0.138541100091356	0.129201523382546	1.07228689309767	0.283591191984732	0.707348823519361
Mib1	172.386590430353	0.297208446174998	0.277177120957379	1.07226904279989	0.283599207177347	0.707348823519361
Mrps7	1670.91196881469	0.174526713525129	0.162818855567973	1.07190726108665	0.283761688518782	0.707460438881185
Ptprz1	13.9274106796245	0.718126773160948	0.669961735918053	1.07189222109363	0.283768444559152	0.707460438881185
Ssr1	1068.17252989861	-0.141445241571943	0.132058809433556	-1.07107766743203	0.284134508846809	0.707907239709081
Tpk1	1003.12110982803	0.158109097467763	0.147609686627884	1.07112955172344	0.284111182272237	0.707907239709081
Ube2m	2490.60831072183	0.132207251794037	0.123429397227617	1.07111640146985	0.284117094350815	0.707907239709081
Cobl	558.308535920977	-0.238230364882563	0.222642983282892	-1.07001065728564	0.284614511683196	0.707960012707887
Cyp2e1	1874.79993564277	0.744941770431247	0.696172825309695	1.07005292845186	0.284595485238596	0.707960012707887
Erp29	2526.03766068894	0.100615972596991	0.0940532487217435	1.06977668463812	0.284719839403125	0.707960012707887
Gng12	929.411197727762	0.116459286694275	0.108876449540121	1.06964625670825	0.284778565731168	0.707960012707887
Kdelr2	1098.80412369163	-0.129534257528873	0.121066800077061	-1.06994037544912	0.284646147763122	0.707960012707887
Lrg1	40.7986119725372	-0.573342039840164	0.535668948252182	-1.07032905624062	0.284471220033927	0.707960012707887
Psme4	2922.93194291847	-0.201761997530855	0.188602157537657	-1.06977565985994	0.284720300786624	0.707960012707887
Rtkn	949.505297058085	0.125306826486956	0.117081468565753	1.07025328621142	0.284505315004167	0.707960012707887
Serpina10	65.9843717559468	-0.352674647038952	0.329388694412802	-1.07069445011664	0.284306839046865	0.707960012707887
Sfi1	713.222973274259	-0.12840528888575	0.120020896434758	-1.06985777227177	0.284683333109897	0.707960012707887
Mkrn2	417.202364319557	0.13046684360464	0.122019885545434	1.06922607754832	0.284967810827745	0.708275560434757
Ccnj	82.390179339718	-0.266776705330645	0.24973997778445	-1.06821786282411	0.28542224895677	0.708854043142731
Ddit4	1849.31745134362	-0.538035205568704	0.503788594481616	-1.06797813896983	0.285530373055702	0.708854043142731
Eng	1107.41283024847	-0.598482420032874	0.560662817890364	-1.06745516366649	0.285766349849071	0.708854043142731
Galnt1	498.625321108923	0.217268334335917	0.203468385255475	1.06782355432327	0.285600110985683	0.708854043142731
Hdac2	1426.12834572069	-0.129742599606231	0.121652881683897	-1.06649836658497	0.286198416679746	0.708854043142731
Kdm4c	310.817900477445	-0.176141516251661	0.165012537063306	-1.06744323423187	0.285771734182903	0.708854043142731
Lrig2	124.062589957049	-0.261982135095702	0.245534775406512	-1.066985866511	0.285978218178966	0.708854043142731
Map4k5	368.770922169309	-0.231309795746497	0.216877759135857	-1.06654456717067	0.286177543457544	0.708854043142731
Oxsr1	1205.75454626091	-0.102945203465393	0.0965181935483593	-1.06658858481239	0.28615765743662	0.708854043142731
Pp3111	993.014438528071	0.130325139575445	0.122122662866477	1.06716588482791	0.285896934780756	0.708854043142731
Ppargc1b	126.744848254231	0.398702359360757	0.37342411101187	1.0676931339018	0.285658956637809	0.708854043142731
Riok3	2155.00186929526	0.128898543190971	0.120697989760975	1.06794275071387	0.285546336779795	0.708854043142731
Rpl35a	3202.99875072857	-0.187765677700109	0.175971321573658	-1.06702430839854	0.285960859260528	0.708854043142731
Sympk	842.629193947359	0.142362588429675	0.133431846110607	1.06693111561737	0.286002942863713	0.708854043142731
Tmx1	639.847866736406	-0.166103113946839	0.155735770499183	-1.06657008479443	0.286166015147836	0.708854043142731
Ubiad1	306.361973897842	0.168959505031422	0.158144291260471	1.06838826545523	0.285345408061861	0.708854043142731
Arg2	2337.13731286136	0.214298728827868	0.201041733301574	1.06594151029532	0.286450082820131	0.708900452789748
Elmo2	764.109703993468	-0.121714887364624	0.114263173984731	-1.06521535434407	0.286778486884327	0.708900452789748
Ints12	148.52734583499	0.211149470956646	0.198068269188934	1.06604390406034	0.286403795676653	0.708900452789748
Lgals12	9.70460328711282	0.912632467225887	0.85626550461319	1.06582883732793	0.286501022519832	0.708900452789748
LOC100359977	53.6562944733411	0.466855062558296	0.438101289250104	1.06563270643967	0.28658970832478	0.708900452789748
Mlst8	448.913416432787	0.14319224450377	0.13442479962613	1.06522193004583	0.28677551188274	0.708900452789748
Ptgr1	446.486191339105	-0.200650852735414	0.188341677422034	-1.06535555742024	0.286715060241195	0.708900452789748
RGD1305627	10.7863314379094	0.84198406236779	0.790112508662833	1.06565084483062	0.286581505790299	0.708900452789748
RGD1308114	2437.49884219862	0.185944563283207	0.174500267827776	1.06558325438633	0.286612072314069	0.708900452789748
Sh2b3	399.580586813886	-0.289271064138689	0.271617102955838	-1.06499576422374	0.286877846568612	0.708991868688593
Ccl20	40.6071905870319	0.826111140989929	0.775893609384663	1.06472218742089	0.287001666547279	0.709049401453207
Efemp1	366.529507864002	-0.222496855798453	0.208988382623796	-1.06463743584721	0.287040032154439	0.709049401453207
Lsmd1	976.267538548971	-0.137155883471274	0.128869852001151	-1.06429767196481	0.287193872288724	0.709049401453207
Map3k1	222.012107874114	-0.308395816473509	0.289813658543364	-1.06411760585592	0.287275426156257	0.709049401453207
Mrps16	1145.45064905889	0.141659244711547	0.133107452043116	1.06424728696378	0.287216690620181	0.709049401453207
Synpo2	41.6080437854695	-0.554402999512466	0.520936023040353	-1.06424392822134	0.287218211769108	0.709049401453207
Ikzf5	236.094847349164	0.175983603823373	0.165407526076634	1.06393951954665	0.287356098722716	0.709094532801214
Adrm1	2386.92813124936	0.166029899025749	0.156093472947112	1.06365689667244	0.287484157421489	0.709102633084341
Cnih	996.168376011206	0.101543070120969	0.0954634045439602	1.06368582396628	0.287471048463152	0.709102633084341
Nbn	700.103876878647	0.175742789328134	0.165297106781313	1.06319337797388	0.287694264828825	0.709466915609552
Sh3bgrl	1763.86319069068	-0.16881276158352	0.158836534865368	-1.06280813621758	0.287868969056946	0.709743753210682
Dlg3	251.36693163089	-0.218762636493947	0.20595523936571	-1.06218534263891	0.288151552567156	0.709893148552343
Dolk	854.960096642595	0.10852597340625	0.10216540926253	1.0622575115162	0.288118797412041	0.709893148552343
Mcat	474.04800319542	0.171792439046916	0.161744239072018	1.06212400535901	0.28817939357914	0.709893148552343
Ubxn4	1596.2998698738	0.112991191232718	0.106361902484286	1.06232766238279	0.288086960574356	0.709893148552343
Trub2	408.34423393358	0.158348552497946	0.149118018489563	1.06190086283254	0.288280693349261	0.709988810532546
Gnai3	2442.07720587625	0.127347599275773	0.119967167958367	1.0615204263217	0.288453455052653	0.710143963021557
Tpx2	86.0949158390898	0.426943652253244	0.402212804487134	1.06148697279204	0.288468650120582	0.710143963021557

Ccnk	539.763489395897	0.14074932315272	0.132674898933238	1.06085871769569	0.288754112723948	0.710429768923424
Dyrk1a	582.323783561838	-0.112837640389163	0.106387460927635	-1.06062913246811	0.288858477699181	0.710429768923424
Enpep	8658.83338047886	0.158060495626772	0.149095181145079	1.06013148388055	0.28908478631628	0.710429768923424
Il17rb	37.5286945521417	-0.454522233463493	0.428440357959507	-1.06087632740343	0.288746108743104	0.710429768923424
Keg1	1129.81254769868	0.673348785986646	0.635091596370653	1.06023885347345	0.289035949260064	0.710429768923424
Lyrm2	912.693470279811	0.128241821883463	0.12092762152122	1.06048411661648	0.288924412179634	0.710429768923424
Napsa	11107.0190169699	0.134711620408142	0.127045251171586	1.06034361116105	0.288988305586864	0.710429768923424
Polr2d	886.037931282755	0.191150940690755	0.180272311941295	1.06034553300122	0.288987431585646	0.710429768923424
Atp7b	213.63171746959	0.183653846435722	0.173298039213935	1.05975720942233	0.289255068508121	0.710694576018872
LOC499602	164.693794618569	0.254643345421337	0.240391008733881	1.05928814377261	0.289468572863873	0.711049319851185
Tmem116	226.477952738058	-0.28456722150297	0.268671244461718	-1.05916515953577	0.289524569089502	0.711049319851185
Arhgap39	170.180806202791	-0.209036607957873	0.197519064047633	-1.05831104944616	0.289913657097675	0.711191964959275
Hmg1l1	2300.60562791438	0.127330522705369	0.120286245945866	1.05856261207681	0.289799021730325	0.711191964959275
Kif2a	380.539850308721	0.133945128999758	0.126530798717754	1.05859704006566	0.289783335506147	0.711191964959275
Otud4	209.429159878129	-0.247230214750394	0.233623929498882	-1.05824011812787	0.289945985530059	0.711191964959275
Pdcd6ip	6103.73069842955	-0.124181968759439	0.117350572107851	-1.0582135777345	0.289958082493514	0.711191964959275
Rnf4	263.144147525053	0.19118391653711	0.180582874323495	1.05870458233279	0.289734340330835	0.711191964959275
Clrn3	520.01065122855	0.7897665125707	0.746596315113448	1.05782267683801	0.290136292298427	0.711214101246422
Exoc8	139.669698508298	0.336367426156407	0.317982561853665	1.05781720920659	0.290138785487493	0.711214101246422
Fxn	229.882326534492	0.211399941920929	0.19985208364777	1.05778202589827	0.290154829093759	0.711214101246422
Crabp2	37.1670071946099	-0.446238756843823	0.42198183306957	-1.05748333665884	0.290291055588559	0.711394596338844
Fabp4	1152.23315313088	1.30824224557504	1.2372941445053	1.05734133745384	0.290355833824458	0.711399958449836
Snap29	834.360489670603	0.119207793147108	0.112788842375706	1.05691122132471	0.290552106697999	0.711586131443209
Znhit1	526.985093565706	0.223748419429107	0.211702458818417	1.05690042845002	0.290557032907613	0.711586131443209
Mdh1b	19.0596294744391	0.49867765827745	0.471979750982658	1.05656578961112	0.290709800547502	0.711806892077317
Peli2	363.825065168085	-0.275303517559837	0.260666605574458	-1.0561518494213	0.290898845185062	0.712116363207715
LOC689986	44.2633738407013	-0.489011022743452	0.463185143839591	-1.05575713998462	0.291079184217263	0.712404394447332
Mst4	22.6184783679934	-0.537378435352535	0.509089655197399	-1.05556738359605	0.29116590888192	0.712463233660258
Sart3	543.832179009588	-0.143235193326168	0.135762242464162	-1.05504439766439	0.291405019899229	0.712894846357002
Cdk14	299.440241550624	0.231838370156264	0.219846518915382	1.0545464686002	0.291632797466615	0.713145089502053
Fbxl8	71.1706343715108	-0.322066529435876	0.30540156982566	-1.05456736721992	0.291623234991323	0.713145089502053
Cyp4f5	26.386695696923	-0.537310485961842	0.50980444228272	-1.05395410749259	0.291903928776556	0.713501086983728
Fgr	41.4301492156057	-0.390035728773067	0.370062852122091	-1.05397157952073	0.291895929180721	0.713501086983728
Banf1	2102.14578091313	0.107835117221325	0.102340577800935	1.05368876684552	0.292025433519441	0.71362108683617
Errfi1	12887.6669369552	0.616309566225108	0.585042224046208	1.05344459065305	0.292137276657808	0.71362108683617
Gys1	587.933019283935	0.228302848646195	0.216722162293287	1.05343563496398	0.292141379293393	0.71362108683617
Padi2	83.9189585252146	-0.609909772937882	0.579049795359944	-1.0532941688698	0.292206190589472	0.713626034000846
Rab20	1017.84136040552	-0.116907408822431	0.11103098944995	-1.05292593897968	0.292374936770691	0.713884754314859
Arcn1	3091.75106743311	0.12270965539961	0.116578936401656	1.05258856520042	0.292529600177232	0.713955644323474
Dlc1	141.332687534088	0.393951007035739	0.374220708705833	1.05272369452279	0.29246764578861	0.713955644323474
Grik5	525.776650766976	0.166205352086725	0.158008281786403	1.05187747254541	0.292855768711606	0.714009758491629
Kif27	69.1764114201566	0.281124337226638	0.2672132854377	1.05205973111012	0.292772145925674	0.714009758491629
LOC100363112	59.0745481864031	-0.331262563499986	0.315013627498118	-1.0515816922935	0.292991510962956	0.714009758491629
Polr3d	545.171399490097	0.163029877389473	0.154997612975536	1.05182185879989	0.292881288270668	0.714009758491629
Tfpi	210.824922422329	-0.170173541344166	0.161793880612144	-1.05179219819883	0.292894899283554	0.714009758491629
Tmem229b	76.6897103726959	-0.302575148706	0.287655066085917	-1.05186796402754	0.292860131793024	0.714009758491629
Wfikkn1	52.3813493732061	-0.412682856614793	0.392401846815949	-1.05168428732793	0.2929444223029	0.714009758491629
Pigb	47.6218885580778	-0.324102409024043	0.308283132696317	-1.05131411566169	0.293114346123658	0.71415598243119
Setd2	1206.39489593522	-0.105343090131555	0.100242587994896	-1.0508815887407	0.293312977329864	0.71433368337931
Tsks	73.1404579729308	-0.338440314625232	0.322042267682652	-1.05091892769411	0.293295826439646	0.71433368337931
Uaca	724.175671805997	0.230677872015681	0.219561639038303	1.05062921294479	0.29342891867545	0.714462958368716
Mcmdc2	30.0591292452197	-0.435474345934328	0.414873354721404	-1.04965609620015	0.293876255983037	0.715245722805824
Rufy2	55.1017743695237	0.347788784524473	0.331334752212512	1.04965984461964	0.293874531974708	0.715245722805824
Rap1gap2	510.189670475936	-0.238733087106034	0.227514480244757	-1.04930941911569	0.294035732291177	0.715327511391592
Rnf168	299.948595425007	-0.246817467175156	0.235203648234839	-1.04937771598135	0.294004310188232	0.715327511391592
Gtf3c2	1476.64522294916	-0.0968539107110048	0.0923291078741919	-1.04900732760224	0.294174745922819	0.715512553425799
Abi2	325.906846817134	0.161417083044373	0.15407236538825	1.04767057114762	0.294790410797416	0.715723276203542
Agps	83.2790298639759	0.363154556902574	0.346348073122332	1.04852483696165	0.294396865311352	0.715723276203542
Ambp	166.670634193944	-0.253466287644921	0.242223203151831	-1.04641621589837	0.295368909146963	0.715723276203542
Arhgef15	722.301411444592	-0.147762305221307	0.140992514307726	-1.0480152506452	0.294631580509972	0.715723276203542
Atp5s	537.581031353983	-0.143291078434049	0.136726203777109	-1.04801475120045	0.29463181061547	0.715723276203542
Ell	664.685263769187	-0.231977288719274	0.221699308226427	-1.04636000254159	0.2953948520738	0.715723276203542
Gfra1	157.85219195326	0.967968990955555	0.924352342201038	1.04718617215883	0.295013721586797	0.715723276203542
Glod4	1373.00576799495	0.107648515711856	0.102793920268861	1.04722648411791	0.294995133213813	0.715723276203542
Ikbkg	46.6337443287216	0.349765251377538	0.334212619839563	1.0465351414481	0.295314029056093	0.715723276203542
Rab38	22.4323920655153	0.51823501749446	0.495130120119322	1.04666429376094	0.295254437398529	0.715723276203542
Rps6ka1	1361.14860028578	0.123980823498844	0.118465867090543	1.04655312575467	0.295305730505638	0.715723276203542
Sipa1l3	497.293901213483	-0.139938505383549	0.133627052164574	-1.04723185250844	0.294992657837747	0.715723276203542
Slc35a3	575.975773815524	0.247654426331939	0.236509936121837	1.04712060048234	0.295043959223049	0.715723276203542
Spire2	74.8574115463015	0.283828223768343	0.271218676986506	1.0464921771684	0.295333854819887	0.715723276203542
Tnk2	630.061348669987	0.147215950558445	0.140647504896959	1.04670147306415	0.295237284136536	0.715723276203542
Tnrc18	1133.64715235377	-0.12125139535948	0.115839416078341	-1.04671966990476	0.295228888978435	0.715723276203542
Xpo6	1152.41757880023	0.100997131562028	0.0964798540902246	1.04682094012683	0.295182170628399	0.715723276203542
Zc3h7b	2077.04286860217	0.109500020857644	0.104522054802872	1.04762598730153	0.294810959479092	0.715723276203542
Bloc1s6	656.386660480851	-0.148761952503024	0.14228944053929	-1.0454883506408	0.295797321891359	0.716393002475428
Zfp91	1837.85739552794	0.0923433772859997	0.0883185015570736	1.04557228279428	0.295758551742095	0.716393002475428
Psmg1	707.641251087106	-0.107189113155087	0.102575964137249	-1.04497300178106	0.29603544769816	0.716620644371679
RGD1559747	287.706793477268	0.162672365441816	0.155685867071368	1.04487561075306	0.29608046330894	0.716620644371679
Sox7	35.5843744802588	-0.486060102700081	0.465171697398335	-1.0449047210279	0.296067007618239	0.716620644371679
Abcb9	125.09775398957	0.258297831254062	0.247401613863797	1.04404262858311	0.296465667461824	0.716966749858687
Ehd1	3060.6246377418	0.14572450463146	0.139580082099101	1.04402076886583	0.296475780779151	0.716966749858687
Plk3	233.938052160067	0.38886445640669	0.372439406070727	1.04410126873858	0.296438538950878	0.716966749858687
Zwint	2572.82067535196	0.128413122422415	0.122953613079074	1.04440300050255	0.296298975992876	0.716966749858687
Cib1	2422.09955171124	0.116699308744001	0.111823024622123	1.04360715638266	0.296667180564629	0.717276999425138
Cdc27	213.141649132611	0.230621109009639	0.221019984158306	1.04344007573748	0.296744520811634	0.717311404411959
Egf	15809.1789326657	-0.217122190331681	0.208207145361785	-1.04281815090642	0.297032523181929	0.7173959198625
Lamb2	4247.37360748066	-0.260347210421903	0.249670024342786	-1.04276519020344	0.297057056978003	0.7173959198625
Shprh	316.923606476011	-0.18150887383052	0.174069145127992	-1.0427400772093	0.297068690927758	0.7173959198625
Sprtn	63.5389071238141	-0.343672481327236	0.329574083259031	-1.04277762962667	0.297051294352064	0.7173959198625
Zfp956	27.8394314268458	-0.465511595580746	0.446455477351343	-1.04268313235276	0.297095072566671	0.7173959198625
Rbm41	46.2640652798249	-0.416494175042809	0.399525335681767	-1.04247249885188	0.297192669303358	0.717479158730239
Acss2	25380.8649056557	0.165724627158333	0.159034452052719	1.04206745783232	0.297380404724812	0.717649540152976
Casp9	416.980541137794	0.127224858998314	0.122139323676408	1.04163716621995	0.297579930549523	0.717649540152976
Khdrbs1	1903.26615123525	-0.107252163142976	0.102966372028926	-1.04162321182732	0.297586402685985	0.717649540152976
Mcm3	88.337797619458	-0.391842165938577	0.376102209649304	-1.04185020955859	0.297481131369562	0.717649540152976
Tmem88	114.468294249043	-0.278945660861559	0.267799882386974	-1.04161980347131	0.297587983517575	0.717649540152976
Trex1	209.491995868532	0.187223775416307	0.179781088502609	1.04139860858385	0.297690588048313	0.717649540152976
Zdhhc17	173.740559188442	-0.209420265700918	0.201101296418751	-1.04136705943877	0.297705224513574	0.717649540152976
Bpifc	19.5816847169041	-0.479216949054299	0.460278627643447	-1.04114534169838	0.297808098678655	0.71774530313435
Anapc15	744.923572266192	0.125112069383402	0.120237601525468	1.04054029518296	0.298088953279961	0.718145893034226
Harbi1	259.564115382679	-0.247992715840287	0.238336502529272	-1.04051504158424	0.298100679512183	0.718145893034226
Socs2	19.5536494826365	-0.533284213246105	0.512805095123239	-1.03993548098024	0.298369876817704	0.718642089406659
Birc2	444.676772497375	0.124465113526027	0.119730796960254	1.03954134346357	0.29855304056385	0.71893090234083
Akt2	663.749787801566	0.142151095504881	0.136817179842333	1.03898571560015	0.298811379664685	0.718943721691111
Esyt1	620.118031822294	-0.227476619094078	0.218912555801363	-1.03912093238035	0.298748496906451	0.718943721691111

Sertad3	173.877423997967	-0.227952885365726	0.219330472151068	-1.03931242717938	0.298659457090242	0.718943721691111
Tceb3	836.34282557347	-0.109488298350013	0.105363756730615	-1.03914573423899	0.298736963725275	0.718943721691111
F5	165.87015202777	-0.338735229450996	0.326147627833549	-1.03859479739608	0.298993226431291	0.719228997167843
Casr	178.933929067032	0.275155512897863	0.265220992836527	1.03745751780463	0.299522684444055	0.72004543811149
Itm2c	4537.15400516435	-0.175311919303297	0.168966871119711	-1.03755202508953	0.299478662973046	0.72004543811149
Rfwd2	1644.4942183715	-0.107913699334577	0.104002209612763	-1.03760967902873	0.299451809901551	0.72004543811149
MGC95152	613.419220915859	-0.175458075098346	0.169250481272193	-1.03667696410369	0.299886431221425	0.720494072126655
Mybl2	18.5620748841126	0.557606632640954	0.537893387903399	1.03664898134255	0.299899476969136	0.720494072126655
Zfand2a	377.268782695837	-0.161414329076426	0.155686032823969	-1.03679389954611	0.299831919242304	0.720494072126655
RGD1564379	1452.90933576328	0.11011894436303	0.106258280455753	1.03633282875197	0.300046895682521	0.720695903704042
Pip5k1b	64.545260902168	0.39551167358333	0.38170179279262	1.03617976402383	0.300118285552568	0.720715071538239
Cnpy2	1560.39757848046	-0.156330214854942	0.150971011549866	-1.03549822744154	0.300436293815023	0.720896431521744
Ikbkb	709.393917775827	-0.120376076243816	0.116242740059711	-1.03555780070206	0.300408487689529	0.720896431521744
Spcs3	148.059886151267	0.312472517938265	0.301767555666794	1.03547419883432	0.300447509776395	0.720896431521744
Zfp955a	213.54470032002	0.221518131130629	0.213903619976008	1.03559786017401	0.30038979068969	0.720896431521744
Armc10	456.724494122392	0.128174622348978	0.123964332089439	1.03396372318209	0.301153122736218	0.722323149280457
Kif13b	209.788759758438	-0.25854339858952	0.250117204345743	-1.03368898299427	0.301281585111609	0.722323149280457
Rpl22	2316.3375054071	-0.153476490637004	0.148443949615062	-1.03390196121157	0.301181998074888	0.722323149280457
Zfp282	243.260867903803	-0.169717611483915	0.164191347742436	-1.03365746013698	0.301296326829021	0.722323149280457
Fam214b	215.0884727587	0.202259986053062	0.195787437400524	1.03305906006266	0.301576260742597	0.722774697528434
Ttc1	955.844825589916	0.104037752679609	0.10071584304998	1.03298298985572	0.301611859086518	0.722774697528434
Slc39a11	83.8981896397232	0.268596593548905	0.260133649638515	1.0325330610713	0.301822468098713	0.723126933475964
Ankrd29	37.3431570505539	-0.431323929198573	0.417981069805751	-1.03192216192715	0.302108583035253	0.723202644224659
Apip	238.373492264951	0.167451482230387	0.162253706229953	1.0320348676231	0.302055783690505	0.723202644224659
Mutyh	144.297530780251	-0.2602003551791	0.25209009339599	-1.03217207655348	0.301991513581062	0.723202644224659
Ppp1r16b	83.7215849355353	0.358079571713099	0.346967655637204	1.03202579806895	0.302060032285972	0.723202644224659
Dennd2c	30.0140781030302	-0.492311122920761	0.4773836989274	-1.03126923694064	0.302414580266116	0.72332578268196
Fam60a	170.982418244354	-0.217430890690314	0.210819801327084	-1.03135895832182	0.302372519584867	0.72332578268196
Plp2	692.475774962728	-0.143110596518948	0.138766528600732	-1.03130486841474	0.302397876044046	0.72332578268196
Spns3	146.272789128035	0.365773454014488	0.354642195927534	1.03138729179657	0.302359237882252	0.72332578268196
Lgals8	1566.6270414827	0.107605222225563	0.104358739658414	1.03110887097501	0.302489768270607	0.723353399150794
Dcun1d1	739.97325002271	-0.191024895906779	0.18528713828824	-1.03096684244544	0.302556369102363	0.723360473541745
Eif3b	3171.09247372964	0.0942896384505004	0.0915509403211676	1.02991447296691	0.303050156088802	0.723497331179788
Engase	197.532122879501	-0.207874949268613	0.201840871448562	-1.02989522278984	0.303059193537478	0.723497331179788
LOC687399	35.337960784441	0.42163859900001	0.409247746169402	1.03027714372672	0.302879925250995	0.723497331179788
Nptxr	54.4296750936767	-0.323004931192277	0.313544987746173	-1.03017092862528	0.302929774037932	0.723497331179788
Rpl12	6375.33316260546	-0.128721821706605	0.124961966111117	-1.03008799967299	0.302968697979883	0.723497331179788
Tmem9b	460.281425616315	0.131053693629688	0.12716856965902	1.03055097640152	0.302751435476674	0.723497331179788
Tssc1	280.399004930349	0.16261075615861	0.157875386720772	1.02999434893682	0.303012658349026	0.723497331179788
Amn1	191.063261782631	-0.173076690467099	0.168111229031842	-1.02953676243909	0.303227513918169	0.723515667199567
Lgmn	7757.18173059991	-0.215940711408817	0.209751278196945	-1.02950844097389	0.303240815331013	0.723515667199567
Matn1	219.502489206175	-1.21972934284166	1.18493820762741	-1.02936113882588	0.303310003270321	0.723515667199567
Txndc12	1103.74582742461	-0.10491911205154	0.101928859050498	-1.02933666705286	0.303321498698393	0.723515667199567
Slc6a4	92.4617555421019	0.270220553187845	0.262630778157245	1.02889903111834	0.303527123722586	0.72385423588563
Tanc2	138.160413948778	-0.295945882190238	0.287890591955614	-1.02798038720163	0.303959053538011	0.724732243027697
Abcg3l3	98.5244562992998	-0.401096100097229	0.390627962546447	-1.02679822888905	0.304515483540235	0.724807053707223
Aldh1a2	164.897819378222	-0.459179077891146	0.447138010316937	-1.02692919701833	0.30445380487366	0.724807053707223
Arhgap18	1466.78554742709	0.155578842768272	0.151414868707524	1.02750043041539	0.304184882869846	0.724807053707223
Cirh1a	410.837319473515	0.161880231483597	0.157692073957957	1.02655908708992	0.304628127358737	0.724807053707223
Dnajb12	1626.06631446599	0.110836565826682	0.107911114907461	1.02710982016755	0.304368755077325	0.724807053707223
Hoxa6	49.991900680734	-0.319227767004465	0.310844661015186	-1.0269687951593	0.304435157996288	0.724807053707223
Prkar2b	320.506992431251	-0.648967154553197	0.632076610573261	-1.02672230501397	0.304551243238734	0.724807053707223
Rem2	29.0122149197973	0.593013648651154	0.577442605116191	1.02696552591894	0.304436697462165	0.724807053707223
Rpl27a	8145.4318553058	-0.150328218195183	0.146424385740021	-1.02666108131806	0.304580081269641	0.724807053707223
Swt1	427.418963011604	-0.179377508634362	0.174722664244441	-1.02664133133529	0.304589384436984	0.724807053707223
Thoc6	389.070871837936	-0.173240833947646	0.168804419617945	-1.02628138729864	0.30475896799147	0.724966603221233
LOC288913	1459.83692107387	0.145139857524749	0.141443483067656	1.02613322563137	0.304828790885694	0.724980966144967
Fmo3	17305.0579060435	0.157067717650381	0.153192827699242	1.02529419953489	0.305224391894195	0.725769966165151
Memo1	605.726864509756	0.130230385548574	0.127038982053909	1.025121450464	0.305305885388246	0.725811899879273
Tmem216	491.372575842241	-0.170271291580648	0.166128849009139	-1.02493511871187	0.305393802622662	0.725869084192634
Dpys	3196.09066095107	0.191942600231341	0.187423466686654	1.02411188750575	0.305782430214129	0.726187211944181
Ick	286.635671753683	-0.207037328656587	0.202137978354029	-1.02423765361884	0.305723037853823	0.726187211944181
Kcng4	428.322250146796	-0.16681789065992	0.162933492206212	-1.02384039279531	0.305910668191525	0.726187211944181
Prdm4	206.357739041972	-0.174534756270533	0.170442301777194	-1.02401079104581	0.305830178011813	0.726187211944181
Rlim	100.132048746199	0.266684251669945	0.260508039414955	1.02370833648305	0.305973056638413	0.726187211944181
RT1-A3	435.96836507685	0.138148488215089	0.134949580751505	1.0237044638877	0.305974886327704	0.726187211944181
Zbtb41	61.7931817135846	0.338436024890595	0.330460087230764	1.02413585775719	0.305771109803665	0.726187211944181
Uso1	2821.74791870657	0.119773620826344	0.11701934315685	1.02353694351029	0.306054041804171	0.726223431972069
Tnfaip1	1495.79513697354	-0.137570420209333	0.134487943425895	-1.02292009755608	0.306345626253285	0.726763595907919
C1qbp	2190.61084017987	0.142999300715702	0.139856559065286	1.02247117812293	0.306557947172372	0.726963827991067
LOC687696	43.0620907495108	-0.365927677919366	0.357884566953546	-1.02247403690605	0.306556594774007	0.726963827991067
Fndc1	237.785578182528	-0.669997669776625	0.655550489451078	-1.0220382419936	0.306762800963646	0.727297871454485
Asnsd1	703.086392763597	0.133931317248626	0.131077076096017	1.02177528853724	0.30688726775865	0.727441227392036
Heca	794.175293269695	-0.114191861493787	0.111783401865109	-1.02154577145169	0.306995935067438	0.727547080478836
Cesl1	2365.89769440215	0.484894624056755	0.475006120305441	1.02081763440218	0.307340847959511	0.728212649136502
Cox20	869.514074783393	-0.184703731075636	0.181040260946918	-1.0202356653131	0.307616706627255	0.728430561922338
Ino80	543.834634394143	-0.192450177714735	0.188589526341504	-1.02047118654002	0.307505047677345	0.728430561922338
Myo18a	2944.34416386001	0.159234062822593	0.156078468552595	1.02021799867248	0.307625083338661	0.728430561922338
Tti1	694.426677383827	0.13160891264873	0.129054908347352	1.01979005939475	0.307828038657198	0.728759318345702
Mtch2	3655.97801906972	0.117107995481662	0.114871484076528	1.01946968321263	0.307980038879718	0.728967330676149
Pign	688.682141233942	0.275369334343434	0.270182948674229	1.01919582895462	0.308110006464749	0.729123117526199
Adat1	40.6183176065264	-0.330170916058029	0.324227422876244	-1.0183312476442	0.30852056332364	0.729207284449225
Capn10	386.385363358154	-0.150724705713068	0.147951954025652	-1.01874089264772	0.308325993376443	0.729207284449225
Cyp4a1	354.414099076577	0.379762641329984	0.372983565866481	1.01817526583981	0.308594671669961	0.729207284449225
Gsdma	1802.15903985505	-0.167075752990794	0.16406352680958	-1.01836012086166	0.308506846691587	0.729207284449225
mrpl9	1096.38224149028	0.123553783331841	0.121309336061023	1.01850185108332	0.308439521585532	0.729207284449225
RGD1359529	2998.54666815661	-0.287016569716994	0.281888946515584	-1.01819022442984	0.308587564200163	0.729207284449225
Scn7a	12.9101273733763	0.825138158652141	0.810056406717778	1.01861815030323	0.308384284047347	0.729207284449225
Dph2	172.751076046196	-0.17472673945151	0.171645131641697	-1.01795336564655	0.308700118713806	0.729304832529853
Nap1l4	1576.95486491435	0.090481377893152	0.0889089318383552	1.0176860302141	0.308827188235287	0.729383592029034
RGD1305645	552.688122993215	0.175542849132749	0.172504489095634	1.01761322301259	0.308861800847769	0.729383592029034
Kif3a	221.798754453926	0.169138839048302	0.166274453854088	1.01722685071471	0.309045525519705	0.729665858549432
Sp110	450.50472792512	-0.182604525959262	0.179567650622482	-1.01691215163896	0.309195222082345	0.72986768311276
Zeb2	149.064012958337	-0.310759282800291	0.305656460527914	-1.01669463247583	0.309298719952201	0.729960392644667
Srp68	1482.39034036023	0.0897133055121498	0.0882600685520882	1.01646539577752	0.309407817920649	0.730066277451962
Bet1	683.02153869711	-0.14592932050103	0.143651958922281	-1.01585332769448	0.309699236965139	0.730450617834772
Hnrnpa3	1990.0110691325	-0.114987500090676	0.113190949089431	-1.01587186091906	0.309690410229231	0.730450617834772
Ccdc124	1171.13042124896	0.134796446682838	0.132726100214171	1.01559863858974	0.309820553438714	0.730542009519506
Mboat1	291.803780707343	-0.191604834706442	0.188704962410057	-1.0153672286057	0.309930808543292	0.730542009519506
Spata9	37.3393458166782	-0.414602415891805	0.408326184563572	-1.01537063153307	0.309929187033526	0.730542009519506
RGD1311558	853.454472455418	0.145695687894712	0.143571170796795	1.01479765809616	0.310202290328902	0.730878779410926
Slc19a3	140.341648687968	0.365049973925421	0.359711241136919	1.01484171796141	0.310181283902068	0.730878779410926
Cdk17	324.462687951727	-0.283634035078997	0.279696064010955	-1.01407946544392	0.31054483555676	0.731273597794891
Fth1	94426.6408696304	0.218841252020843	0.215757082948717	1.01429463649571	0.310442182567723	0.731273597794891
Gramd1a	693.81672253831	-0.181994365589919	0.179474250224032	-1.01404165423587	0.310562876698569	0.731273597794891

Ahcyl2	2790.42900404065	0.433498695833532	0.427875523624208	1.01314207497006	0.310992303622975	0.731687785255777
Asb7	61.4803288250492	0.387456661396666	0.382503343737167	1.01294973688623	0.311084169744875	0.731687785255777
Hsh2d	139.601411663701	-0.306403241198014	0.302501939167633	-1.01289678354167	0.311109464905968	0.731687785255777
Ndufs8	2877.82426341211	0.112931580119671	0.111435044589632	1.01342966690192	0.310854974954673	0.731687785255777
Pex3	422.32366381211	0.193884459051101	0.19136799134441	1.01314988828075	0.310988572142611	0.731687785255777
Phtf2	249.551834199373	-0.211155373160755	0.208473526914995	-1.01286420528038	0.311125027814638	0.731687785255777
Vps16	819.798864648358	-0.109249309180875	0.107883359861043	-1.01266135316504	0.311221943525601	0.731764296671902
Necap2	1001.35268122138	0.092216862748943	0.0910830168448259	1.01244848867982	0.311323664208806	0.731852071850524
Hoxa2	41.3977350523163	-0.349234673339145	0.345026378198126	-1.01219702436375	0.311443858684386	0.731896934355282
Trpv4	1567.12239307288	0.081182255033886	0.0802085925265857	1.01213912969458	0.311471535410567	0.731896934355282
Pkp3	667.860658984079	0.132133838134195	0.13064369563265	1.01140615698545	0.311822075534583	0.732569183655658
Sept11	159.239354403799	-0.357671568561758	0.353738319408662	-1.01111909266621	0.311959433293181	0.732740425461934
Map2k5	284.408840884582	0.153264104191766	0.151646577749121	1.01066642232653	0.312176113095303	0.733097872198598
Adar	401.143585473886	-0.145935724396637	0.144499675028357	-1.0099380802621	0.312524956852514	0.733462452939434
Dlgap3	33.5791160524784	0.889237934119408	0.880402324060699	1.01003587770868	0.312478101264949	0.733462452939434
Klhl5	310.30966495896	0.173318081429719	0.171608068182819	1.00996464364996	0.312512229649886	0.733462452939434
Rnmt	341.83666493446	0.21015951763898	0.20812992004367	1.00975158975165	0.312614318867848	0.733520715989256
Bet1l	256.53864644464	-0.158161499048551	0.156673034957229	-1.00950044844493	0.312734686667688	0.733651692190906
Arid5a	71.0269180923517	-0.479363500402955	0.475835655514274	-1.00741399861023	0.313735867006293	0.73387978275232
Arl2	730.563889700127	0.136199156933018	0.135048705951428	1.00851878567429	0.313205473912785	0.73387978275232
Eps15	402.036005554157	0.173070702162474	0.171690732646549	1.00803753059151	0.313436445261801	0.73387978275232
LOC690137	91.5428795949389	-0.373704632509041	0.370836450308381	-1.00773435890209	0.313582005627544	0.73387978275232
Mib2	796.761091119162	-0.0968835266336373	0.0961510730484681	-1.00761773698355	0.313638010594106	0.73387978275232
Mylpf	68.27602600117	-0.414247054835616	0.410675512818591	-1.00869674939349	0.313120091209912	0.73387978275232
Ncapd3	229.247744841696	0.16603925158513	0.164687499894457	1.00820797991067	0.313354627782286	0.73387978275232
Nmnat1	523.652985237212	-0.159678781060469	0.158430109852719	-1.00788152712202	0.313511340902616	0.73387978275232
Pgrmc1	10024.0652078943	0.175777815666978	0.174310522887789	1.00841769478331	0.31325398171107	0.73387978275232
Pold4	1420.58290257709	0.17393138207784	0.172619719518603	1.00759856731835	0.313647217010225	0.73387978275232
Rfng	264.339019614638	0.145220044651432	0.144083870418839	1.00788550605485	0.313509430512052	0.73387978275232
RGD1308923	298.208456625746	0.171941229584611	0.17047070069695	1.00862628523053	0.313153896384085	0.73387978275232
Ssr4	982.850207468321	-0.114057673256993	0.11320592965942	-1.00752384261262	0.313683105966611	0.73387978275232
Tdrd3	444.77251271283	0.144019267205031	0.142804412151556	1.00850712548143	0.313211068727233	0.73387978275232
Gltscr1l	197.367423219894	-0.194841928571403	0.193437530298076	-1.00726021610782	0.313809742702925	0.733901550527047
Aar2	454.825198060192	0.153525972161563	0.152559210248828	1.00633696196485	0.314253506081615	0.733927319441496
Ctsh	10053.4821119127	0.282261078089887	0.280446867465399	1.0064689994254	0.31419001681627	0.733927319441496
Flii	1505.86288183012	-0.0892138618775448	0.0886556055128538	-1.00629690995241	0.314272766472089	0.733927319441496
Fuom	604.468768666807	0.129912184515767	0.12899634158031	1.00709975898725	0.313886837024602	0.733927319441496
Mtf2	171.649932663726	-0.238143628694599	0.236646582845722	-1.00632608267939	0.314258737684163	0.733927319441496
Nmt1	1305.1479096425	0.126399745954379	0.125531117868129	1.00691962360411	0.313973400943247	0.733927319441496
RGD1565310	625.410284119548	0.119960358079977	0.119192194458049	1.00644474770702	0.314201677448526	0.733927319441496
Alg8	241.194315997333	-0.147162773535152	0.146267016593177	-1.00612412123279	0.314355866781133	0.733970579670164
Trpm1	16.1958914893287	-0.681661662277643	0.67809805100679	-1.00525530380977	0.314773931291601	0.734795749242212
Colq	7.12537492109158	0.838031438267293	0.833806693200893	1.00506681596688	0.31486467759878	0.734856658231567
Lysmd3	36.5108957350288	0.42044998703672	0.418563822943541	1.00450627596029	0.315134647712398	0.735335743358473
Ror1	18.6490501197596	0.549749633236594	0.547358896544599	1.00436776803499	0.315201380028055	0.735340493718979
Cd74	3553.77184333933	-0.276222212142941	0.275147568916839	-1.00390569769645	0.31542406998706	0.735709004611722
Cox4i2	24.2215292513149	-0.573965039421011	0.572211143954985	-1.00306511937867	0.315829442850274	0.736050327544847
Pid1	24.2297025518689	-0.527051127039883	0.525305393006064	-1.00332327453147	0.31570490992754	0.736050327544847
Rgs5	66.2162109755652	-0.671646181309271	0.669416469366517	-1.00333082922932	0.315701266059874	0.736050327544847
Tmem252	10531.6122058327	0.94167644474273	0.938784894534265	1.00308009878014	0.315822215970766	0.736050327544847
Trappc11	1041.22441698873	0.138419364060635	0.138031313797398	1.00281132050809	0.315951905761187	0.736184780828547
Slc1a3	56.3248438493093	-0.373162992493104	0.372254364153001	-1.0024408802894	0.316130706353857	0.736450421893409
B4galt6	636.811375798429	-0.135385546659381	0.135137440943542	-1.00183595097041	0.316422830704058	0.736913687371158
Cks2	73.3647723897845	0.359461375831469	0.358867153823075	1.00165582723875	0.316509847855916	0.736913687371158
Faah	266.443105520747	0.191362960073399	0.191077833495353	1.00149220122937	0.316588908625142	0.736913687371158
Slc9a4	193.59577641229	-0.299859289477953	0.299379306904619	-1.00160325901712	0.316535246341822	0.736913687371158
Sema4b	578.922084542487	-0.263465841753179	0.263109812959194	-1.00135315665342	0.316656102327513	0.736919176710589
Baz1a	246.941097014636	-0.158173010600221	0.158031301766667	-1.00089671370146	0.316876745502391	0.737199863505942
Depdc7	759.308606408114	0.207405102054251	0.207280179052338	1.00060267702626	0.31701893535803	0.737199863505942
Golga1	383.384502306648	-0.15457161700111	0.154444393850401	-1.00082374728883	0.316912026597855	0.737199863505942
Mcu	120.981995616258	0.238715086144962	0.238579793503897	1.00056707501955	0.31703615456775	0.737199863505942
Crebzf	440.147529110708	-0.141077170238386	0.141157798119608	-0.999428810293898	0.317587009181764	0.737575387486703
Gpr19	19.6607078924441	-0.590038117231406	0.590321972403979	-0.999519151944461	0.317543266114902	0.737575387486703
LOC498675	131.128131424021	-0.266659460702187	0.266678405545003	-0.999928959966677	0.31734488830073	0.737575387486703
Plcd4	78.9800201173714	0.303373495688246	0.303407096397369	0.999889255361783	0.317364104751226	0.737575387486703
Ptges2	1106.19293400903	0.137108525621364	0.137186404938062	0.999432310244349	0.317585314446171	0.737575387486703
Rac1	1042.08268990909	0.119985015446902	0.12003749474478	0.999562809120688	0.317522128899837	0.737575387486703
Ppp2r5a	2825.98556264017	-0.144030434891511	0.144135959958847	-0.999267878277104	0.317664941607198	0.73760566420989
Dhx40	802.591282617999	0.107910165784453	0.108023391944636	0.998951836651811	0.317818023392683	0.737659721029454
Hsd17b4	9392.78493886666	0.129544940734802	0.129680426525549	0.998955233304073	0.317816377891707	0.737659721029454
Ramp2	145.812893202202	-0.361941196350748	0.36255871634795	-0.998296772441656	0.31813547222843	0.738245769160541
Cenpf	96.2420906895296	0.36373785807866	0.364416006914281	0.99813908054873	0.318211921867971	0.738272444162352
Cd164	17869.417212059	0.188392978797613	0.188886648084122	0.997386425713428	0.318576977634944	0.738516403793345
Pus7	68.9355601153287	0.289435265100908	0.290167818199068	0.997475415769031	0.318533801018822	0.738516403793345
Rps9	3651.08625024099	-0.136538851527135	0.136857305383698	-0.99767309566946	0.318437903461786	0.738516403793345
Snx8	871.770510379595	0.169623548691139	0.170037216647609	0.997567191673529	0.318489276765614	0.738516403793345
Hic2	57.2483462283933	-0.301456391402887	0.302400052957063	-0.996879426623943	0.318823038966166	0.738785455838826
Tns1	3642.8059264074	0.218783308293557	0.21945016218512	0.996961251315911	0.318783318657028	0.738785455838826
Bmyc	183.362610954878	0.281864241108053	0.282801310943605	0.996686472801612	0.318916717720359	0.73884871315628
Fam76b	307.606563048836	-0.163262905040465	0.163827219011979	-0.996555432150304	0.318980348007907	0.73884871315628
Scn1a	10.1893117689464	-1.07949545140706	1.08346651164102	-0.996334856507978	0.319087473167818	0.738946255098903
Zfp112	43.5756567193351	0.336655989542549	0.337950230989002	0.996170319390918	0.319167397889941	0.73898077906235
Pdlim5	143.575971910673	0.26532427921861	0.266416218405985	0.995901378700186	0.319298065375193	0.738982246193125
Timp2	370.615015143424	-0.251250077925968	0.252251739166474	-0.996029120576868	0.319235996361035	0.738982246193125
Upk2	99.1410891118361	0.523696114532985	0.525974648752164	0.995667977107671	0.31941149420929	0.739094267748939
Rsl1d1	732.04619625475	0.134026114152354	0.134631180210075	0.995505750920576	0.319490348649467	0.739126257429236
Baalc	29.4992248229004	-0.566971783199526	0.57033880720479	-0.99409644940388	0.320175913785655	0.739403373277302
Eef2	24419.2966425867	0.149650053435551	0.150434669454846	0.994784340457304	0.319841164024719	0.739403373277302
Fam118a	384.992626633808	-0.189237082266694	0.190419828388544	-0.993788744943952	0.320325726730374	0.739403373277302
Mapre3	483.654805242998	0.132169942297856	0.132993112679661	0.993810428485964	0.32031516810203	0.739403373277302
Mcf2l	607.696449771759	0.111692023261114	0.112354858443458	0.994100520515734	0.320173931978975	0.739403373277302
Mdfi	284.125936313704	-0.907855566722059	0.913364707892277	-0.993968300808413	0.320238300288951	0.739403373277302
Pdhb	5820.89316686057	0.120893332030685	0.121492245984822	0.995070352438691	0.319702048856575	0.739403373277302
Pex1	1718.64850378159	0.140159305212766	0.141030928419019	0.993819630799966	0.320310687177178	0.739403373277302
Slc46a3	58.3280263286183	0.373926562273287	0.375875794205356	0.994814159458739	0.319826658324426	0.739403373277302
Xiap	121.481885071859	0.426763722839724	0.429182990762964	0.99436308526827	0.320046133078769	0.739403373277302
Zswim5	109.457494724721	0.213959089797991	0.215138479912558	0.994517995502031	0.319970748821378	0.739403373277302
Fmc1	829.74417390617	-0.136350944765663	0.137397428110096	-0.99238353032639	0.321010469966798	0.74008215043461
Krt8	4057.76634136829	0.179086418062716	0.180436592513159	0.992517180514007	0.320945302808188	0.74008215043461
Mgat4b	2891.98667945206	0.140594547771387	0.141646075172209	0.992576374604493	0.320916442829682	0.74008215043461
Mif4gd	648.750374670194	-0.135870820865625	0.136904512010566	-0.992449546550658	0.320978279707886	0.74008215043461
RGD1310852	56.2224377457145	0.304012377018039	0.306223457440123	0.992779519764529	0.320817412645788	0.74008215043461
Usp45	254.237351529594	-0.217621758175556	0.219212552338467	-0.992743142917954	0.320835144338834	0.74008215043461
Ing4	773.42434501019	-0.138329174514925	0.139434263888716	-0.992074477657279	0.321161195596424	0.740172760491225
RGD1563888	6.53648478954778	1.08533518235463	1.09404829717365	0.992035895635016	0.321180015374162	0.740172760491225

Atp6v1a	4847.50227496164	0.12940196352902	0.130630572980807	0.990594778666643	0.321883488279269	0.740806075766069
Calr3	59.1457305444403	-0.273504919462569	0.276113607287734	-0.990552121459025	0.321904326462199	0.740806075766069
Ddt	4672.03838874705	0.166016193557711	0.167611432766655	0.990482515526464	0.321938331074451	0.740806075766069
Fgfr3	619.729399503219	-0.128389363993104	0.129632604201202	-0.990409509893301	0.321973999062745	0.740806075766069
Nono	2916.47408043328	-0.0760446338752924	0.0767145002252979	-0.991268060822423	0.32155470441076	0.740806075766069
Pdzd2	201.783925934408	0.262706002736966	0.265145637774811	0.990798886761557	0.321783793005563	0.740806075766069
Spag7	1383.78704559979	-0.1217205400024	0.122899774323148	-0.990404910609128	0.321976246197816	0.740806075766069
Usp33	1108.64717977765	-0.122940256160687	0.124117735102595	-0.990513209567236	0.321923335814264	0.740806075766069
Slc26a10	72.2438485916102	0.478692915157967	0.483502240715294	0.990053147323139	0.32214814231917	0.741051565593945
Fam210a	1747.79252158769	0.0873041557125373	0.0882197021250078	0.989621973432044	0.322358925687899	0.741373993372515
Snap47	337.527046483274	0.138492734314793	0.140000133082069	0.989232876183107	0.322549216718906	0.741373993372515
Thop1	722.998830658837	0.133466945273703	0.134892400692058	0.989432648458759	0.322451507385467	0.741373993372515
Tm4sf20	216.237140973602	0.304932754131476	0.308237633464804	0.989278144604928	0.322527074079949	0.741373993372515
Akr1c12l1	23494.0261050511	0.226491126207971	0.229006359148795	0.989016755036091	0.322654944090323	0.741427922615294
Ap5s1	77.1532740175269	0.243760712066196	0.246591988954983	0.988518374417654	0.322898840132474	0.741427922615294
Cars2	533.703489667393	0.120389360126519	0.121784955695694	0.98854049286135	0.322888013324876	0.741427922615294
Plcg1	2382.10430112838	-0.112768152652547	0.114045097368173	-0.988803159933274	0.32275945791699	0.741427922615294
Zfp36l1	2511.10997196874	-0.152056081169602	0.153806732599944	-0.988617849162069	0.322850149874013	0.741427922615294
Alad	1812.97565130501	0.155117892703629	0.156949246943372	0.988331551279097	0.322990298061999	0.741488129220902
Gtf3c1	1100.34858227616	-0.119682021369809	0.121272390518634	-0.986885975100984	0.323698540435735	0.741595143664528
Kcnj14	102.56457419708	-0.362480366981717	0.367212694628267	-0.987112843004132	0.323587322389582	0.741595143664528
Kptn	89.9391624604685	0.227450205249685	0.230199782736638	0.988055690347466	0.323125374685512	0.741595143664528
Mep1a	16481.5912640737	0.272950227145559	0.276626090595976	0.986711797710413	0.323783944773428	0.741595143664528
Mobp	59.9284709520574	-0.321866020356143	0.326107356047027	-0.986994050847869	0.323645555073346	0.741595143664528
Pabpn1	2903.21436342631	-0.115738688859992	0.117191156803145	-0.987605993636596	0.323345649768093	0.741595143664528
Rab11fip1	219.684139491762	-0.285498699202259	0.28921056869721	-0.987165512271313	0.323561505758771	0.741595143664528
RGD1563482	161.132388519967	0.202331636008945	0.204858568450471	0.987664990238683	0.323316745867189	0.741595143664528
Sp140	294.769685974248	-0.227769707251677	0.230730129764193	-0.987169328446434	0.323559635255482	0.741595143664528
Tubgcp5	269.286333222644	-0.161595122834794	0.163778567119721	-0.986668314888047	0.323805267979907	0.741595143664528
Znhit6	391.967735947501	-0.140124784455989	0.141832935643072	-0.98795659710956	0.323173905152269	0.741595143664528
Zpbp2	44.3053444809127	-0.363064503612697	0.367981274675621	-0.986638529182611	0.323819874890643	0.741595143664528
Cox16	1312.62166142681	0.175918619152714	0.178463371154729	0.985740760215672	0.324260342274358	0.741823230162628
Dhrs7	35824.6169271382	-0.192397632665889	0.1952012268008	-0.985637415395077	0.324311070796947	0.741823230162628
Sh3yl1	517.551865037685	-0.170421122923116	0.17286749489789	-0.985848282372465	0.324207568721929	0.741823230162628
Tmx2	1301.91708790008	0.134700638142717	0.136657140587228	0.985683130525752	0.324288630128002	0.741823230162628
Ugcg	279.169309363228	0.244615304099733	0.248107140850754	0.98592609330369	0.324169381391392	0.741823230162628
Zdhhc13	859.39076992449	0.172901094677506	0.17539785542713	0.985765158054273	0.324248366943816	0.741823230162628
Ankrd27	722.975094874299	-0.111685819157747	0.113345998912141	-0.985352991986242	0.324450711435223	0.741844052740443
Reep6	1651.87172916333	-0.2113412786697	0.214461780695555	-0.985449612440339	0.324403270226038	0.741844052740443
Milr1	30.2041098117611	-0.441193250059713	0.447865456146697	-0.985102208720473	0.324573868565025	0.741944403963644
Smurf1	762.263860027344	0.134774893634042	0.136827609975119	0.984997791443922	0.324625155807778	0.741944403963644
Rfxap	300.729194030856	-0.182624036320978	0.185458475141473	-0.984716585109776	0.324763303796374	0.742110918968555
Bag1	768.706718581588	0.109519541473063	0.111331239530408	0.983726956917154	0.325249781810361	0.742625355575846
Flna	1587.60652107232	-0.526912140582265	0.535586576055651	-0.983803859429659	0.325211961358663	0.742625355575846
Pik3r6	24.2906210056246	-0.667208967541693	0.677992663848434	-0.984094671105245	0.325068966816381	0.742625355575846
RGD1307325	114.390120344054	0.225950847286758	0.229666975312196	0.983819493332089	0.325204272996877	0.742625355575846
Vps53	683.139420435462	0.100520969596906	0.102197932827601	0.983591025921004	0.325316639312275	0.742628825551981
Rdx	1563.70126134821	-0.101199964697316	0.102930755410528	-0.983184902254831	0.325516444016954	0.742935723433072
Ank3	1251.58645066105	0.102840086423957	0.104621310183557	0.98297456076134	0.325619959183378	0.743022777771186
Mfsd9	194.541161860151	0.202849527757231	0.206537731191853	0.982142713520971	0.326029545115318	0.743808071011784
B4galnt1	409.060553201054	0.281477380652003	0.286930649268168	0.98099447155585	0.32659546782871	0.744195793357948
Ddx59	107.410728491632	0.215069609212759	0.219108262236428	0.98156777392853	0.326312830202477	0.744195793357948
Hmgb2	560.66553183596	0.166314442927298	0.169526424774738	0.981053208361427	0.32656650331829	0.744195793357948
Hnrnph2	1511.55901552965	0.130642240797432	0.13314096947256	0.981232458460936	0.326478121165995	0.744195793357948
Nxph4	203.473905731158	0.172712759716379	0.176065576101162	0.980957002163463	0.326613945745756	0.744195793357948
Rab15	285.806140424933	-0.196569298738653	0.200306180924684	-0.981344149397788	0.326423058008522	0.744195793357948
Secp43	612.066163431491	0.164784526869547	0.167998671702417	0.980868034251112	0.3266578227224	0.744195793357948
Art3	31.067468076284	-0.5638695321006	0.575013676958708	-0.980619339496323	0.326780493703104	0.744232809218122
Bsdc1	1663.29132990324	0.119001798085926	0.121360534204042	0.980564224328894	0.326807683817752	0.744232809218122
Galk2	1292.80501919488	0.141103036660772	0.143935431784924	0.980321765884684	0.326927313949269	0.744232809218122
Sh3gl1	762.894326744191	-0.127487173797139	0.130048585737939	-0.980304192265788	0.326935985960415	0.744232809218122
Chchd6	261.2072065796	0.195300238439591	0.199275072417961	0.980053531381824	0.327059695242345	0.744311547690907
Nme2	7768.09819747004	0.159673158427008	0.162936990781635	0.979968745347698	0.327101546781284	0.744311547690907
Tubb2a	1962.40656226377	0.203421265648641	0.207613474926435	0.979807624339991	0.327181087883993	0.744343523796929
Narfl	856.279194147265	0.140163983671405	0.14310650433895	0.979438246492454	0.327363487176079	0.744561087182843
Ppp1r12a	281.854069256642	-0.219315731603124	0.224091710155734	-0.978687392990614	0.327734463114995	0.744561087182843
Ppp2r5b	301.649792979613	0.134601004125628	0.137532403710732	0.97868575327696	0.327735273550569	0.744561087182843
Pus1	472.488439673117	0.146035434235335	0.149160793304099	0.979047047152719	0.327556733915846	0.744561087182843
Rpl7a	4968.02050942054	-0.139050547754238	0.142011565791965	-0.979149458558439	0.327506137029318	0.744561087182843
Tbc1d5	254.133057162969	0.263619058323845	0.269201403178491	0.979263314422826	0.327449891904095	0.744561087182843
Zfyve21	601.434150593004	0.131668005119969	0.134503370497931	0.978919744780627	0.327619635386346	0.744561087182843
Nudt3	1793.98796283337	0.113571493960911	0.116087587686369	0.978325902229483	0.327913162950499	0.744816348939923
C1d	781.344427921827	-0.146223145199125	0.149555478627675	-0.977718412865065	0.328213612437785	0.745200942662378
RGD1309148	365.681534785576	-0.183718586996968	0.187904739072866	-0.977721945191198	0.328211864919099	0.745200942662378
Pck2	699.497506169609	0.161025228194993	0.164723989233171	0.977545705058525	0.328299062113367	0.745246083479235
Cidea	105.853113213738	0.909186878890934	0.93043883809855	0.977159208819091	0.328490338966755	0.74546110722726
Pofut1	61.2593578976139	0.319403756672957	0.326893116472103	0.97708927040137	0.328524959185408	0.74546110722726
RGD1564854	112.005815781618	0.234183292612762	0.239729342308586	0.976865369743995	0.32863580816879	0.745563791546202
Golt1b	111.473602199803	-0.263018009208412	0.269430305981557	-0.976200536351006	0.328965097507985	0.746013028387023
LOC499584	18.9709909904097	-0.461032958563716	0.472266193481757	-0.976214187945097	0.328958333777813	0.746013028387023
Capza1	1098.16127302915	0.106638999905966	0.10935755661522	0.975140659745905	0.329490492243479	0.746653250950979
Hao2	118221.896574465	0.208501501521441	0.213809046190557	0.975176238967993	0.329472846343424	0.746653250950979
Lrrc48	228.921454452267	0.174068244922145	0.178486131315929	0.975248013046101	0.329437251076276	0.746653250950979
Tmem204	310.406725809983	-0.380147867716609	0.389854878027441	-0.975100964851467	0.329510180078314	0.746653250950979
Ino80b	471.875233959163	0.148290813800452	0.152103471506841	0.974933789027834	0.329593104142468	0.746692290548793
Ap4m1	169.718317752272	-0.180382368254105	0.185154722558066	-0.974225046825562	0.32994481097916	0.747191217690603
Maea	2300.10908594124	0.111922206156752	0.114879189377956	0.974260061920568	0.329927429353975	0.747191217690603
Abhd1	815.25688994497	-0.151669909122529	0.155883853104078	-0.972967411969629	0.330569498758895	0.748131134096992
Aplp1	1419.36704495344	0.213056093825393	0.219204169564237	0.971952742728088	0.33107405903621	0.748131134096992
Cntf	116.247495076143	-0.305568426256528	0.314376576086342	-0.97198216883883	0.331059419428968	0.748131134096992
Cyp4v3	577.962512622184	-0.25616154581923	0.263247568372489	-0.973082286772605	0.330512406825558	0.748131134096992
Eef2k	243.611450362403	-0.268285992980286	0.276026892641463	-0.971955994623931	0.331072441184503	0.748131134096992
Impdh1	123.707013169606	-0.301723568580376	0.310371657799527	-0.972136343632455	0.33098272369932	0.748131134096992
Mat2a	15088.4760542622	0.158037225426669	0.16258663252767	0.97201856616209	0.331041312194998	0.748131134096992
Mllt3	83.3608158285036	0.266833607463935	0.274505007403401	0.972053697628209	0.33102383531742	0.748131134096992
Pkdcc	653.251490961719	-0.122302135624788	0.12575508048649	-0.972542303274394	0.330780830161578	0.748131134096992
Ptp4a1	2022.8902439759	0.110338721592615	0.113525032886632	0.971932963039097	0.331083899745546	0.748131134096992
Rtdr1	36.1437773908469	-0.32474752934066	0.33407863602881	-0.972069130791875	0.331016157958252	0.748131134096992
Bloc1s5	1153.59201705898	0.12941815006588	0.133180066276022	0.971753158596981	0.331173363986895	0.748177567409977
Ilk	1661.16134373392	-0.0999137125449509	0.102831336821997	-0.971627089881206	0.331236100581296	0.748177567409977
LOC686120	777.428857868841	-0.229815158654907	0.23658165363135	-0.97139890235535	0.331349674929093	0.748182468690668
Ppp6r1	2647.09202467791	0.0999312983564114	0.102877904047251	0.97135822586854	0.331369923226185	0.748182468690668
Hmgcs1	808.565280013608	-0.140709710304586	0.144893059400544	-0.971128022879313	0.33148453075808	0.748292587208805
Cbx2	47.2289050526418	-0.381730399876201	0.393157272786786	-0.970935618640374	0.331580339680706	0.748360234473968
Ppox	789.291976826632	0.170872882876002	0.176020752120625	0.970754191295039	0.331670698986139	0.748415557807515

Cct6a	3195.46677056611	0.147334393278285	0.151833321675883	0.970369294777065	0.331862448156228	0.748699600187314
Clec11a	43.7719448397055	-0.316027702970076	0.326348824813952	-0.968373957375947	0.33285764087043	0.748905001453313
Dcaf13	645.088992939157	-0.134550923574482	0.138947964886315	-0.968354762767263	0.332867223703946	0.748905001453313
Eif3a	4830.48300289521	0.109833516642356	0.113425854502613	0.968328756472589	0.332880207529273	0.748905001453313
Fzr1	364.817940253655	0.147150872154541	0.151816911317455	0.96926535309919	0.332412811244472	0.748905001453313
Hr	66.5501905958218	-0.361172226239135	0.372916374030391	-0.968507288472405	0.332791080756448	0.748905001453313
Hspa14	727.429117892768	0.161025209707936	0.166268182854718	0.968466768224906	0.332811307928928	0.748905001453313
LOC100125368	282.127106679227	0.18924742971826	0.195462085408533	0.968205313694044	0.33294184166317	0.748905001453313
LOC304396	258.076185753296	-0.184682277579395	0.190448381837023	-0.969723532423801	0.332184317335022	0.748905001453313
LOC502684	10.9696051810167	0.590458218978012	0.609445703624535	0.96884466567965	0.332622697328769	0.748905001453313
Lta4h	755.541866525276	0.113221737426227	0.116876941713244	0.96872604438962	0.3326818944017	0.748905001453313
Rpl21	11017.6413537295	-0.143705929316151	0.148216516192012	-0.969567582670626	0.332262078042163	0.748905001453313
Sh3bgr	47.0902993588245	-0.352580367947725	0.364087971520954	-0.968393343166059	0.332847962771122	0.748905001453313
Sh3bp2	121.574601074506	-0.206985114797441	0.213440702108741	-0.969754656691434	0.332168799352468	0.748905001453313
Tead1	76.5789221011481	-0.352106247391761	0.363126513217893	-0.969651717996369	0.332220124463646	0.748905001453313
Vps13b	491.163283021815	-0.169235937896756	0.174647959511229	-0.969011824531935	0.33253928949402	0.748905001453313
Cdca7l	103.49306490088	0.30511190379444	0.315307681016839	0.967664037902538	0.333212183929209	0.749216554409376
Nbr1	4390.66354908795	0.122016925543302	0.126081361055594	0.96776339120816	0.333162550927397	0.749216554409376
Gata2	501.011835672666	0.186070574801132	0.192314671149436	0.967531877256249	0.333278213583168	0.749216806880198
Mrpl48	1151.24227677478	-0.119321067536778	0.123406585252692	-0.966893843569632	0.333597104588995	0.749785384765377
Nup35	366.235104363767	0.188073620483492	0.194541525015778	0.966753090211656	0.333667480002607	0.749795290175886
Cbwd1	137.12740231636	0.282212325722694	0.292025489377121	0.966396208511257	0.333845960569446	0.750048070336495
Ero1l	189.922583941561	0.186167557556402	0.192788089731789	0.965659018746449	0.334214832426371	0.750077264310931
Exd1	64.0971880566878	-0.261240825080423	0.270477365674568	-0.965850966600812	0.334118761086648	0.750077264310931
Rnf150	69.9175049864296	-0.383751578442675	0.397459634848689	-0.965510821215259	0.334289018584343	0.750077264310931
Spns1	1265.21399977834	0.0832702005427285	0.0862503996096093	0.965447127429323	0.334320906299417	0.750077264310931
Tcf12	221.839104901958	0.206568851800566	0.213877098427938	0.965829690597593	0.334129409008112	0.750077264310931
Trpm4	255.004838038391	-0.214547683426359	0.222118852833586	-0.965913882092214	0.33408727528704	0.750077264310931
Wsb1	773.5168540011	-0.181054012193756	0.187451392719547	-0.965871789838544	0.334108339970901	0.750077264310931
Adcy4	163.2029386925	-0.359953858104332	0.373045198402601	-0.964906825354334	0.334591482491504	0.750536173277764
Acot9	577.788736958323	-0.118632560215674	0.12306541987116	-0.963979648709386	0.335056129208779	0.75068952584013
Adamts5	50.1288042104785	-0.624321080161719	0.6473226917849	-0.964466545178947	0.334812073364614	0.75068952584013
Cdc45	33.6774993263141	0.389538623244564	0.403864756106408	0.964527400211991	0.334781577961018	0.75068952584013
Dhrs7b	724.804925258223	0.10045503176435	0.104207014906372	0.963994908160514	0.335048478701413	0.75068952584013
Rhbdl2	22.7710256895304	-0.425063207886321	0.440789265448015	-0.964322957035466	0.334884034710793	0.75068952584013
Srsf1	3894.83781874013	-0.115164826587222	0.119442275438984	-0.964188149999304	0.334951604343055	0.75068952584013
Plk5	30.5051400699035	-0.439145664981952	0.455710621173782	-0.963650274050749	0.335221292543232	0.750911551250764
Hpgd	1007.12499473304	0.145690848381128	0.151217440476736	0.963452680602286	0.335320399986951	0.750985550000332
Ncdn	319.257087718293	0.174151890894098	0.180835296743197	0.963041474925165	0.335526709947486	0.751299563684618
Oas1a	90.2804340662563	-0.260036889226127	0.27006594259955	-0.962864427565775	0.335615563249521	0.751350500668516
Hgfac	52.5789736399923	0.482323845617186	0.50123597195094	0.96222901620779	0.335914488583734	0.751871618204552
Clca5	34.81941271224	-0.555535444808128	0.577967746374357	-0.961187623864915	0.336457838029288	0.75207223824952
Il17ra	930.163269011191	-0.134667176254014	0.140036990416161	-0.961654316147551	0.336223277512323	0.75207223824952
Iscu	3727.68850158024	0.183788055297969	0.191224599247316	0.961110945042539	0.336496387032176	0.75207223824952
Lrrc8b	156.862954424299	0.23095701319501	0.240320578090831	0.961037190530216	0.336533468567399	0.75207223824952
Med31	505.898387074016	-0.185487394642203	0.192908565382265	-0.961530112852396	0.336285692070607	0.75207223824952
Slc25a20	1953.03736734131	0.17209653079794	0.179008898360468	0.961385341031432	0.336358452119189	0.75207223824952
Usp43_predicted	63.4720984085584	-0.455939103046409	0.474103342163916	-0.961687173444927	0.33620676733168	0.75207223824952
Wasl	1903.09895020253	-0.112190749946075	0.116631379147516	-0.961925947940433	0.336086803260615	0.75207223824952
Lgals5	20.7871081971951	0.657132357232655	0.684218156179812	0.960413504519691	0.336847144095892	0.752477327003519
Trmt44	179.057835518039	-0.157647640623419	0.164144670890676	-0.960418877859369	0.336844440835367	0.752477327003519
Aatf	269.725467433782	0.140982322723446	0.14690744895977	0.959667625581423	0.337222521904404	0.75264310088442
Dnajc18	159.336239235241	0.184767523815258	0.192557117527612	0.959546581230696	0.337283465133741	0.75264310088442
Fam134c	1206.93522653698	-0.101120051426984	0.105345955742368	-0.959885462278981	0.337112863641582	0.75264310088442
Mocos	230.283015417092	0.213532094006409	0.222550584186001	0.95947667262893	0.337318665837166	0.75264310088442
Polr2e	2938.04724508015	0.125673171693852	0.130908520541736	0.960007577610546	0.337051401221921	0.75264310088442
Vasn	863.983633150033	-0.175620774769874	0.183031305815077	-0.959512221080418	0.337300766020921	0.75264310088442
Dpf3	17.7875540408199	0.547566667444308	0.571001911865921	0.958957677838536	0.337580066332775	0.752798004517862
Icmt	325.895565428057	0.150791718941545	0.15724762451987	0.958944336373681	0.337586787702582	0.752798004517862
Sgsm2	424.519065191994	0.13488359007992	0.140638043099982	0.959083240258321	0.337516812765186	0.752798004517862
Copz1	3350.12345651383	0.0745515397888254	0.0777606290472342	0.958731181862488	0.337694185645716	0.752889783061829
Nppc	4.67441146877405	1.27126236271019	1.32753635253582	0.957610208023202	0.338259349778012	0.753706286919602
Slc6a13	1569.09001831576	-0.186086729071671	0.194276715073243	-0.957843707628657	0.338141575655772	0.753706286919602
Uqcrfs1	8039.35812781525	0.145195703855603	0.151619575206513	0.957631649197275	0.338248534034325	0.753706286919602
Tmem126b	332.253603741468	0.209731448474779	0.219053869006361	0.95744233793327	0.338344037511794	0.753747222728156
Dusp2	146.532966884553	0.424030428126172	0.443134408564539	0.95688897077469	0.338623298649673	0.754073748521195
Pdha1	14743.2147632846	0.147093584300273	0.153713912805658	0.956930843899894	0.338602161871175	0.754073748521195
Nmrk1	831.376702437909	-0.176718450700385	0.184851397479051	-0.956002784455081	0.339070827724673	0.754922434460066
Dnajb4	272.973832138947	-0.206732251707951	0.216378715707546	-0.955418609598215	0.339366046746689	0.755041582433812
Ndst2	531.769568720338	-0.129826401506434	0.135891070085893	-0.955371102931005	0.339390061996687	0.755041582433812
Thrb	866.710431543106	-0.0966530301817664	0.101142216040913	-0.955615112780895	0.339266723397324	0.755041582433812
Zbtb39	303.704444170885	-0.357387990026848	0.374078620151198	-0.955382026062854	0.339384540112249	0.755041582433812
LOC498368	922.890410742137	0.182400901333178	0.191055008426636	0.954703584246625	0.33972761679529	0.755411765189557
Nos1ap	26.6169638920205	0.465708256381582	0.487966839094031	0.95438505054201	0.339888770647492	0.755411765189557
Phf17	1234.88537751213	-0.129042367825141	0.135192909800307	-0.95450543978785	0.339827857076352	0.755411765189557
Taf7	162.166857830664	-0.166248020801506	0.174144876589358	-0.954653527898655	0.339752938259022	0.755411765189557
Ung	905.896209879987	0.177450273180223	0.185887902003567	0.954609048074674	0.339775439802188	0.755411765189557
Stx6	600.695271955522	0.137729145401824	0.144343764116116	0.954174544672603	0.339995297513575	0.755500792089795
Amhr2	23.0320582156808	0.467904504306266	0.490588028479349	0.953762581114354	0.340203834315107	0.755816415329458
Plxnd1	566.658018489686	-0.640304301821993	0.67190439491902	-0.952969360915052	0.340605594826998	0.756413284643153
Urgcp	835.636740724667	0.100971999807413	0.105951557007979	0.95300156655375	0.340589276977786	0.756413284643153
Dfnb31	600.346917436617	0.137425189545852	0.144307427000053	0.952308501389891	0.340940547577198	0.757009233006921
Snx30	113.949568848178	0.221419129076037	0.232549430238386	0.952137912567926	0.341027043756263	0.75705339440111
Atp6v1d	2591.39108737191	0.124507692492884	0.130826790732195	0.951698744546553	0.341249786273011	0.757184698394808
Epor	26.1139498417619	-0.422398330507825	0.443869511016361	-0.951627268880507	0.341286046971371	0.757184698394808
Ranbp1	916.752442791121	0.125210507835494	0.1315590606427	0.951743705251532	0.341226978283586	0.757184698394808
Slc35e4	91.6251538368483	-0.276103967681915	0.290306814669168	-0.951076425803373	0.341565580836787	0.757656985127034
Golim4	410.396895066388	0.278495026864983	0.293386741466919	0.949242032794057	0.342497528142236	0.759163902385826
Mcm4	222.112745138225	-0.155766564642495	0.164100683334745	-0.949213382157283	0.342512096730118	0.759163902385826
Skp2	30.5104652898789	-0.401156143272512	0.422560391186581	-0.949346298516138	0.342444513316704	0.759163902385826
Spns2	1026.66062234614	-0.25798503604448	0.271760790285018	-0.949309264864552	0.342463342802421	0.759163902385826
Rragc	1715.1211175438	0.0903803436079135	0.0952369852791599	0.949004668123309	0.342618237871715	0.759251099951241
Nudt15	19.8594614046007	0.444683042422884	0.468675289972268	0.948808379569567	0.34271807924319	0.759324305785204
Map2k2	3414.39805952427	0.129751836086529	0.136805807854504	0.948438067954856	0.34290648736857	0.759376979390284
Pgam1	7732.69804789745	0.111278602689836	0.117336978519257	0.948367719146382	0.342942287102792	0.759376979390284
Trmt10a	155.885083461462	-0.185084688730785	0.195118037557747	-0.948578055865329	0.342835256167434	0.759376979390284
Fam175a	144.134016345863	-0.232081148646335	0.244759715280696	-0.948199945322617	0.343027674995504	0.759418105570491
Cdkn2c	228.221098454515	0.194684335928937	0.205363305616594	0.947999621180651	0.343129647051215	0.759495923735951
Micu1	677.455897931574	0.109019591935336	0.115031395380312	0.947737716080903	0.343262995199449	0.759643147086631
Rps17	6977.39845941262	-0.140466290437401	0.148322527952995	-0.947032742604793	0.34362209467415	0.760289804957445
Alkbh3	769.575280768737	0.152962168607383	0.161604070018391	0.946524234135781	0.343881267666653	0.760482412490397
Fam212a	20.1905998042997	-0.419474542247012	0.443154048946787	-0.946565970104409	0.343859991273696	0.760482412490397
Wdr60	261.716265252904	-0.138907195280933	0.146763732775428	-0.946468127064354	0.343909871564371	0.760482412490397
Pts	852.461437083171	-0.14435156395513	0.152619799266749	-0.945824622025822	0.344238044929643	0.761060030863707
Gpr160	189.247477560052	-0.220233960006481	0.232886916724838	-0.945669095987445	0.344317389756714	0.761087408007548

Klhdc8b	64.7180694185046	-0.284644240462402	0.301361338577153	-0.944528059924078	0.344899869807168	0.762226700413818
Slc4a11	521.910750759523	-0.830004644503567	0.878979314894698	-0.944282340253936	0.345025387834205	0.762355862776743
Tbce	784.491582816821	-0.120933495446204	0.128152192519938	-0.943670904634659	0.345337846559096	0.762897952184779
Ankhd1	877.617830825319	0.109847496206797	0.116552826755197	0.942469601681278	0.345952267294762	0.763958319423406
Capn1	2130.47460525681	-0.157881793166294	0.167499259211773	-0.942582038328182	0.345894730703741	0.763958319423406
Anapc7	460.998297924306	0.145974104710404	0.155006091972051	0.941731404574238	0.346330172293316	0.7646442770563
Npy1r	19.9452171123279	-0.715136038747579	0.759534459591003	-0.941545218544355	0.346425527947243	0.764706263478027
Aqp6	1313.84054219316	-0.214781915456764	0.228212532516605	-0.941148643714978	0.346628690492113	0.765006154589
Depdc1b	184.094029887318	0.734253686698681	0.780788724995703	0.940399961209381	0.347012442105507	0.765129516217821
Gemin2	215.259363934854	-0.165773041167538	0.176374695782712	-0.939891294677359	0.347273323115695	0.765129516217821
Lmbrd1	1620.96861546291	-0.114053945719322	0.1212561430037	-0.940603444032043	0.34690811632451	0.765129516217821
LOC100188932	967.980799817514	-0.105957000223715	0.112618320839343	-0.940850471166851	0.346781492176917	0.765129516217821
Ncoa4	1963.41570051315	-0.15152620175976	0.16108860568599	-0.940638855954402	0.346889962647565	0.765129516217821
Pm20d1	7966.64382778101	0.112044485614107	0.119214280029347	0.939857922947863	0.347290442914635	0.765129516217821
Pnpla2	1103.75450495026	0.19301236013968	0.205315461552798	0.940077082748324	0.347178023085228	0.765129516217821
Tfrc	1327.57508205364	0.157214582263113	0.16726209907065	0.939929506664313	0.347253720909366	0.765129516217821
Wbp1l	2009.87379354585	0.124464825374515	0.132396864817789	0.940088917859272	0.347171952826421	0.765129516217821
Cc2d1b	779.062474278054	-0.10365684878338	0.110366344450065	-0.939207049937936	0.347624450115656	0.765214939870471
Fndc5	11.2414486578153	0.678656563374851	0.722390650206363	0.939459229131746	0.347495015585471	0.765214939870471
Marveld1	227.689673995731	-0.323930472332725	0.344927440490637	-0.939126419956485	0.347665841060277	0.765214939870471
Pmfbp1	163.130692286655	-0.235770888261458	0.251025876912517	-0.939229417944126	0.347612968181644	0.765214939870471
RGD1564937	134.116945614075	0.305036423449618	0.324661378634527	0.939552541582098	0.347447129425047	0.765214939870471
Cops8	1075.69883402415	0.0878292263266626	0.0935727943080009	0.938619253343734	0.347926264030761	0.765343510155532
Kif5b	633.278653193247	0.194830133162486	0.207562059018127	0.938659666820279	0.34790550772824	0.765343510155532
Slc2a2	521.624916819331	0.259802630532971	0.276764347667532	0.938714226461941	0.347877487224988	0.765343510155532
Hcfc1r1	761.852922365674	0.131253339378243	0.139896973795621	0.938214285964434	0.348134298159908	0.765504823541404
Vamp4	419.94838424201	-0.126240814629455	0.134547373110604	-0.938262945688869	0.348109297193724	0.765504823541404
Atp5sl	1462.6794700148	-0.0991175974488649	0.10566928246081	-0.937998206674917	0.348245331709459	0.765600858841338
Tmem35	53.7079865016865	-0.337875227667481	0.360321908392052	-0.937703813723845	0.348396643391883	0.765785389439595
Abcc4	385.668426942701	-0.239755418592379	0.255782424906949	-0.937341252744787	0.348583049388937	0.765955377764838
Sri	823.708373702264	0.175360153511195	0.187092488759224	0.937291254577689	0.348608760265485	0.765955377764838
Acot8	1217.64258468905	0.12634460579527	0.135217772392896	0.934378695635927	0.350108583277771	0.766049795047126
Acp2	2221.94555612119	0.0835393012894621	0.0893134223483013	0.935349907023812	0.349608003412362	0.766049795047126
Ak7	19.7096349946598	0.468037942753111	0.499729447612674	0.936582674863447	0.348973267271062	0.766049795047126
Arf2	536.240698103579	0.114225534023869	0.121965365457103	0.936540743314905	0.348994845248294	0.766049795047126
Cmtm7	134.451591054737	-0.294848943174822	0.315262520363131	-0.935248956441775	0.349660013996769	0.766049795047126
Ctr9	1593.22753320458	0.0940550388914546	0.100617505991473	0.934778078274225	0.349902679241601	0.766049795047126
Eif2b1	727.600713729238	-0.142325607044847	0.152194804910568	-0.935154173813484	0.349708851269666	0.766049795047126
Hgs	1200.61583278482	0.101402273968434	0.108354326837379	0.935839637678901	0.349355759780502	0.766049795047126
Hoxa9	58.6579816966828	0.272938941915277	0.291382096092014	0.936704573053408	0.34891054326815	0.766049795047126
Inpp5f	1127.04331833854	-0.18816591703831	0.201081464354336	-0.935769577979267	0.349391838068568	0.766049795047126
Kcne2	16.6664784513867	-0.691568138334667	0.739829805230074	-0.934766527984909	0.349908632981066	0.766049795047126
Lad1	2336.31485482618	0.121179501492109	0.129657367512703	0.9346133105798	0.349987616869347	0.766049795047126
Lmf2	770.25239101427	-0.170212198435649	0.181845791859643	-0.936024951113669	0.349260341264204	0.766049795047126
Nat10	361.518495249754	0.142975221075288	0.152849735555893	0.935397241973022	0.349583617741696	0.766049795047126
Nfkbib	509.133616930193	0.145544253017242	0.155683377887751	0.934873427028159	0.349853533002886	0.766049795047126
Pbk	56.4676622541767	0.504943684102364	0.540068038100653	0.934963094424517	0.349807319140769	0.766049795047126
Srd5a3	116.157637613922	-0.227365411769947	0.243173102566451	-0.934994081871432	0.349791349358131	0.766049795047126
Thoc4	838.055026739735	-0.13108057404501	0.140293892270845	-0.934328443835255	0.350134496328508	0.766049795047126
Tie1	829.491725853354	-0.43593045390015	0.466537386171439	-0.934395542182676	0.350099896390325	0.766049795047126
Tstd2	259.701798764272	0.163245811304175	0.174680883490729	0.934537357734621	0.350026774911068	0.766049795047126
Ubl4a	729.277240366612	0.12332492477021	0.131680925744037	0.936543573591899	0.348993388760926	0.766049795047126
Xrcc1	1187.39757726737	-0.112582806962829	0.120338430795478	-0.935551562527604	0.349504123512452	0.766049795047126
RGD1564664	392.767969231523	-0.532367106324615	0.570284056888745	-0.933512168004503	0.350555590881809	0.766823488445467
Brpf3	143.481944261788	0.226840987244664	0.24306118860493	0.933267003862841	0.350682127095274	0.766840466043729
Hace1	202.953428466335	-0.165663251842472	0.177545875624067	-0.93307293824862	0.350782310431454	0.766840466043729
Tab2	758.13597630494	-0.109172644278247	0.116987437621793	-0.933199722103406	0.350716858195953	0.766840466043729
Ykt6	278.179430507495	0.145640131037288	0.156103042537036	0.932974326895227	0.350833223951029	0.766840466043729
Lars2	279.019289909918	0.210578587870478	0.225827118839405	0.932476971555526	0.351090082314351	0.767090411112268
Mon1b	178.293981638172	-0.193237399147422	0.207256009222602	-0.932360899315959	0.351150044784192	0.767090411112268
Tcp11l1	53.9931624288223	0.299198306148049	0.320894778125993	0.932387581671944	0.351136260207893	0.767090411112268
Arhgap8	458.802318615509	0.117810682404746	0.12650341930792	0.931284569612976	0.351706381628539	0.767420758992124
Copb1	2156.4111257554	0.110653966092449	0.118783165544737	0.931562697331668	0.351562568594335	0.767420758992124
Cyp20a1	594.250672995071	0.148074961738489	0.158952844343297	0.931565347888247	0.351561198235634	0.767420758992124
Glg1	844.83555014244	0.237583197083594	0.255058514003777	0.931485067305283	0.351602705432334	0.767420758992124
Lamp2	4286.78042078353	0.124808342188371	0.134010297555975	0.931333968094803	0.351680836162111	0.767420758992124
Rps25	6420.19760325609	-0.159716871882849	0.171381584580038	-0.931937187266808	0.351368987871794	0.767420758992124
Nedd4	5075.81556853436	-0.13043609730243	0.140164799742717	-0.930590972497057	0.35206518601132	0.76776149351586
Rab35	698.33812072029	-0.105761216897531	0.113635747753691	-0.930703752896241	0.3520068278657	0.76776149351586
Rpusd3	571.663170598459	0.137071848899405	0.147281927623959	0.930676635692722	0.352020859086041	0.76776149351586
Aebp1	633.56437666184	-0.260664913175129	0.280483417154183	-0.929341619621812	0.352712072592199	0.767799541975299
Agbl5	225.498977157613	0.1613010111799	0.173630150615733	0.928991944128879	0.35289326124221	0.767799541975299
Anapc4	902.844284532878	-0.0942095850831044	0.101272271493934	-0.930260412779893	0.352236269212074	0.767799541975299
Anxa13	1668.03727360641	-0.591931486951087	0.637004878863125	-0.929241684942067	0.352763848965421	0.767799541975299
Atad1	1188.84695803511	0.0885360547686724	0.0952956845631468	0.929066779619016	0.352854479362908	0.767799541975299
Cacng5	640.608338314435	0.626681409532758	0.674197915821937	0.92952142809393	0.352618925543893	0.767799541975299
Cidec	704.583032756035	0.161345593461616	0.173579570868257	0.929519485815953	0.352619931628335	0.767799541975299
Cln3	379.8018725659	-0.168480200245601	0.181265819362154	-0.929464809407843	0.352648254315257	0.767799541975299
Fscn2	18.696844315125	-0.497553832977572	0.535458367678686	-0.929211051709889	0.352779721071979	0.767799541975299
LOC497899	214.548327473097	0.202448488558269	0.217755762812198	0.929704389650847	0.352524161066663	0.767799541975299
Nr0b2	81.496750764809	0.416916630213664	0.448323640211258	0.929945674997654	0.35239921257366	0.767799541975299
RGD1305807	139.11458582439	0.207653996354228	0.223232259610948	0.930214999911435	0.352259777030464	0.767799541975299
Brd8	796.829129141959	-0.101943606398432	0.109827769366589	-0.928213392536083	0.353296888752005	0.76782539673226
Cybasc3	338.882838535924	-0.171164640013044	0.184349983462916	-0.928476568306696	0.353160416872304	0.76782539673226
Hmgb3	333.372603676249	0.133702536058079	0.144012508573879	0.928409187383117	0.353195354608224	0.76782539673226
Prr5l	22.7032036624536	-0.53565717593955	0.577025213463062	-0.928308093722215	0.353247776861779	0.76782539673226
Rundc3b	42.0574444097982	0.327974570706851	0.353349593796646	0.928187201753517	0.353310472013876	0.76782539673226
Unc93b1	242.052140021972	-0.286984867564867	0.309174912011308	-0.9282281854563	0.353289216881694	0.76782539673226
Cops6	2095.16759040546	0.0926739464162929	0.0998961916950744	0.927702496399194	0.353561913175911	0.767847352352057
Gimap1	31.6194411444271	-0.431132557385088	0.464824607717124	-0.927516637947577	0.353658357343218	0.767847352352057
Mfi2	25.9886321707496	-0.430961492903372	0.464603817987447	-0.927589219499303	0.353620691931563	0.767847352352057
Morn2	106.555890049938	0.293996576438419	0.316971518330538	0.927517330222204	0.353657998082807	0.767847352352057
RSA-14-44	117.228462734557	0.219327901080749	0.236413475525394	0.9277301160322	0.353547582437986	0.767847352352057
Pdp2	327.699600949241	0.199031107186645	0.214657635590887	0.927202550418358	0.353821378899772	0.767907922966356
Pik3ca	1094.25663884822	-0.117470630707171	0.126688315704238	-0.927241238106076	0.353801296178054	0.767907922966356
Gramd3	527.039904217888	0.12752955329437	0.13764236929986	0.926528320771214	0.354171486190512	0.768521021771929
Dynlt1	344.351304924839	0.161354287614373	0.174294408068337	0.925757110641836	0.354572220788058	0.769098813844423
Mcm5	90.9565206256948	0.292300508199417	0.315742687960056	0.925755431069221	0.354573093836422	0.769098813844423
RGD1564149	239.599149788293	0.212244641627549	0.229344474655852	0.925440396791932	0.354736873882851	0.769307252156552
Pld1	3059.23867391862	0.160593460677255	0.173598884779428	0.925083481275253	0.354922484852904	0.769416166858308
Slc35c2	566.449329112748	0.222250316627142	0.240244411649692	0.925100879978895	0.354913435379432	0.769416166858308
Ube4b	1746.6146926293	0.104584873056285	0.113174606194937	0.924101939229574	0.35543324374968	0.770376477585595
Arfgap2	1804.7462797983	0.0897326112708881	0.0971313948517804	0.923827063410522	0.355576362237831	0.770539739408044
Edf1	3649.8752344091	0.129605988177014	0.140315991897708	0.923672251638278	0.355656983461731	0.770567532219984
Gtlf3b	310.269213820442	0.133483001993908	0.144624096345672	0.92296515841223	0.356025362516221	0.771168420449709

Pigh	571.594249319086	-0.142651382682778	0.154572062519922	-0.92287946707312	0.356070022041182	0.771168420449709
Adra1d	26.3136350811751	0.412147684186945	0.446748925413594	0.922548798086944	0.35624238901879	0.771394740633943
Ifi30	1924.40921227794	0.114651601127342	0.124328346205626	0.92216782919094	0.356441040899782	0.771408101319989
Rp9	49.510095181108	0.287491118285367	0.311762957981168	0.92214649279384	0.356452168584601	0.771408101319989
Srebf2	993.719951542582	-0.1754614742804	0.190240339499143	-0.922314766375773	0.35636441391297	0.771408101319989
RGD1563982	19.5079732051109	0.420489494721715	0.456059269953678	0.922006244417363	0.356525318516821	0.771419526035063
Fkbp1b	75.625995025042	0.228599196892142	0.248002364835117	0.921762165631466	0.356652646266647	0.771548149498803
Gstt2	2509.15012882101	-0.25804011024793	0.280158455833314	-0.921050587177195	0.357024016488053	0.771827126500931
Kcnj2	14.4182394197617	-0.570693752118146	0.61954740371626	-0.921146224961847	0.356974089310772	0.771827126500931
R3hdm1	585.14648756863	0.101318757710757	0.109975167604599	0.92128759535093	0.356900295735745	0.771827126500931
Rfc1	615.083550610064	-0.111502408440247	0.121081811903904	-0.920884868560942	0.357110539389591	0.771827126500931
Rnf217	388.414944707822	0.173471687226542	0.188379152461938	0.920864569987871	0.357121138330846	0.771827126500931
Anxa4	3655.85425722668	-0.0983937079479805	0.106926927727666	-0.920195782661788	0.357470457787849	0.772288390651338
Lpp	23.6715025175369	0.388411135862444	0.422080951313824	0.920229009751386	0.357453097614144	0.772288390651338
Spata6	360.823054878408	0.160881173525887	0.174912086328276	0.919783057323689	0.357686138840994	0.772607497922223
Cd86	33.904291971244	-0.374501732232258	0.408262092792006	-0.917307138831163	0.35898171757492	0.772943170459241
Chmp5	2609.42130176489	-0.120002198634282	0.130879889621845	-0.91688798776502	0.359201339176039	0.772943170459241
Cstf2	619.20383654988	-0.156443464020562	0.170489992451025	-0.917610833172521	0.358822644308562	0.772943170459241
Hivep1	286.48776539538	-0.189209261605558	0.205999763342192	-0.918492616378673	0.35836102276511	0.772943170459241
Hsd17b13	21.8030557162909	-0.470187092709472	0.511701709073922	-0.918869498326314	0.358163835629255	0.772943170459241
Map1lc3b	4286.73309107337	0.0999376923456935	0.108893245221607	0.917758416900073	0.358745356844427	0.772943170459241
Mbnl1	124.526300085316	0.313708952388475	0.341559790194938	0.918459846252488	0.358378171538815	0.772943170459241
Morn4	295.73597188699	0.139937730855284	0.152616599281514	0.916923398333342	0.359182781918805	0.772943170459241
Nlrp6	1391.1003441673	0.190414171977387	0.207595947752798	0.91723453197713	0.359019755172482	0.772943170459241
Osbpl2	943.722687984761	0.0842563209015382	0.0917247902658873	0.918577416828102	0.358316648654651	0.772943170459241
Parp6	531.313927502712	-0.110909651259305	0.120698958159576	-0.918894851707601	0.358150573022851	0.772943170459241
Psma2	2182.15880348406	-0.152179156147044	0.165965006428007	-0.916935198704418	0.35917659795192	0.772943170459241
Rbbp5	403.903064416887	-0.110205552450763	0.120212306037511	-0.916757660537468	0.359269643633307	0.772943170459241
Rbm4b	371.009751417222	-0.16648520823824	0.181590914571635	-0.916814635968828	0.359239781824317	0.772943170459241
RT1-CE16	1549.1917558844	0.393242801503077	0.428546582329482	0.917619735445091	0.358817982020485	0.772943170459241
Sema4c	129.926541756464	-0.433053354103681	0.471353497947377	-0.918744330931067	0.358229316469039	0.772943170459241
Tmub1	268.230514021867	0.138510608090285	0.15108214155591	0.91679007633756	0.359252653760564	0.772943170459241
Trove2	69.3807598809593	-0.293337429185261	0.319868285934284	-0.917056932757398	0.359112807308377	0.772943170459241
Ubxn6	1169.46814626821	0.098861451702842	0.107538443945415	0.919312648349485	0.357932063934918	0.772943170459241
Wsb2	1970.94369323135	0.198374283344058	0.216106257290912	0.917947892073371	0.358646146782006	0.772943170459241
Zfp800	128.436342112091	0.200871248561143	0.218819544935795	0.917976721960928	0.358631052834135	0.772943170459241
Ube2cbp	101.688791932031	0.224096573243634	0.244481654133127	0.916619179619945	0.359342230380092	0.772953026211227
RGD1561849	259.446679035418	0.805398981327464	0.879025095540832	0.916241169237531	0.359540416814589	0.77323299479936
Wnk4	1990.47100258156	-0.167858792613874	0.183240446176659	-0.916057541423163	0.359636715511541	0.773293777620919
Cenpm	24.6333656438442	0.365805482717667	0.39961922748738	0.915385090496475	0.359989502702761	0.773905936773138
Celf1	455.392527257559	0.181644412513428	0.19877418415751	0.913822955849698	0.360809882088941	0.773981751216706
Col4a1	3306.78169942959	-0.404114134191879	0.441746622035441	-0.914809789217715	0.360291494800177	0.773981751216706
Ctbs	1122.97727471347	0.178936014803785	0.195861050548175	0.913586516068304	0.360934154252287	0.773981751216706
Gars	1890.28778840525	0.0818683549319522	0.0896009293078221	0.913699841780605	0.360874587188497	0.773981751216706
Magoh	582.288495768607	-0.190496393854835	0.208477993430457	-0.913748212558365	0.360849164075659	0.773981751216706
Mlph	129.28560550155	0.354357581518651	0.387665922665246	0.91407978055539	0.360674926079636	0.773981751216706
Naa25	174.666561753903	0.194971641360938	0.213386331276759	0.913702579703021	0.360873148138623	0.773981751216706
Ncs1	169.176696602184	-0.235556152518062	0.257868179959667	-0.913475065263599	0.360992741829119	0.773981751216706
Nsmaf	437.469199787268	-0.129216108058165	0.141322999390993	-0.914331769174156	0.360542542121985	0.773981751216706
Pafah2	121.606957974648	0.400808987528058	0.438215465454471	0.914639073982433	0.360381138698073	0.773981751216706
Shkbp1	273.424809346647	0.160811347321969	0.17603814394688	0.913502856349673	0.360978132023353	0.773981751216706
Slc35b1	959.667950961757	0.124385026813259	0.136182017517272	0.913373359279857	0.361046211943601	0.773981751216706
Spry2	295.153252381027	-0.234474068444525	0.256250019482989	-0.915020685335365	0.360180770937275	0.773981751216706
Ube2z	1779.78321491861	0.0787952416510736	0.0862360361481316	0.913715949510056	0.360866121030242	0.773981751216706
Vcl	1004.8535769744	-0.147838196060106	0.161558966083512	-0.915072679925952	0.360153476221622	0.773981751216706
Irak2	942.396487366646	-0.120561367530788	0.132101950308856	-0.912638816072847	0.361432532470128	0.774663806157926
Aco1	7347.67958914064	0.185940558130991	0.203771424548688	0.912495746362922	0.361507807699337	0.774679061521618
Tert	35.9141496133527	-0.367401016716585	0.402740692573999	-0.91225203584085	0.361636057088912	0.774807808647045
Zc3h18	720.785274226672	0.0916762515961581	0.10051235668853	0.912089365094154	0.361721676254299	0.774845188900558
Bax	678.296503482657	-0.156065830461412	0.171722064227016	-0.908828059829824	0.363440892543323	0.775945265095556
Ccdc114	120.226089211021	-0.300723165977479	0.330316803086304	-0.910408320641525	0.362607211250448	0.775945265095556
Cdkn2aip	450.607808176069	-0.177431540961124	0.195080525108445	-0.909529748612732	0.363070562180265	0.775945265095556
Cyb5a	8380.4455617377	0.170879211141091	0.187731570749574	0.910231616657787	0.362700373570509	0.775945265095556
Dhx16	675.382542669071	0.109977183842948	0.121016727542357	0.908776712743745	0.363468001276873	0.775945265095556
Dvl2	252.876902224491	-0.144988085233108	0.159556801838807	-0.90869260076786	0.363512410992525	0.775945265095556
Gfod2	77.2391244602828	0.274788936491108	0.302236113022988	0.909186310473121	0.363251789044599	0.775945265095556
Mt1a	5626.57305305124	0.856597980270178	0.94270994878685	0.908654863961617	0.363532336497786	0.775945265095556
Otog	181.926062687279	-0.193661909939644	0.213054305739217	-0.90897909463839	0.36336116093317	0.775945265095556
PCOLCE2	96.6415673420956	0.314723751860148	0.3459699728273	0.909685165126313	0.362988569950676	0.775945265095556
Pde4b	63.8138143115456	-0.338927576026921	0.372492439211156	-0.909891155763275	0.362879914473706	0.775945265095556
Pitpna	1520.99316572676	0.0900714935279691	0.0990537373726034	0.909319485736854	0.36318150784978	0.775945265095556
Plce1	177.411306564867	-0.279063909916908	0.306547411920542	-0.910345020264735	0.362640582912152	0.775945265095556
Polr3gl	180.707766894861	-0.175892277944821	0.193260629981143	-0.91012990054924	0.362754007389687	0.775945265095556
Pomt1	490.736502151054	0.124886544608013	0.137379596280693	0.90906181113566	0.363317499349604	0.775945265095556
Pphln1	433.542144776561	0.10949623920422	0.12027408836274	0.910389267503616	0.362617255773067	0.775945265095556
Recql5	376.528875797173	-0.116058045374487	0.127518468708547	-0.910127345080865	0.362755354924847	0.775945265095556
RGD1561149	1578.99178715595	-0.15667878865443	0.172262437405858	-0.909535421731484	0.363067569040586	0.775945265095556
Smlr1	46.3617969702087	0.564939050942823	0.621461179061221	0.909049623656653	0.363323932257098	0.775945265095556
Amn	3615.50871022766	0.149414282048742	0.164572120558231	0.90789546578076	0.363933453438645	0.775959588730739
Ankrd49	276.226724785824	0.133048107948959	0.146550272481217	0.907866670572116	0.363948668619001	0.775959588730739
Blnk	458.413927289132	-0.16976019086359	0.186876368817877	-0.908409083167878	0.363662128544596	0.775959588730739
Cnot10	716.499599667135	-0.104008901031457	0.114547893839833	-0.907994879215222	0.363880927154787	0.775959588730739
Met	501.088413734679	0.25154073998505	0.277032554267566	0.907982603885983	0.363887412716071	0.775959588730739
Sbno2	1085.98777168628	-0.105708592495513	0.116379150139884	-0.908312119210826	0.363713341292781	0.775959588730739
Nit1	5952.51272987272	0.186560906804245	0.205739917648401	0.906780312428571	0.364522982976725	0.776234495135847
Nras	251.5156363312	0.153368420628254	0.169209999778152	0.906379178708898	0.364735189521006	0.776234495135847
Phyhd1	6125.5994508728	0.119317821652133	0.131560228423232	0.906944470089278	0.364436163041775	0.776234495135847
Plekhd1	121.726183159845	0.229896753520002	0.25383712398121	0.905686094745619	0.365102024546563	0.776234495135847
Ppil2	904.267981650624	0.0930423661001776	0.102622908508623	0.906643238359973	0.364595488805232	0.776234495135847
Prps1	415.076758909944	0.134281213640797	0.148042147166206	0.907047190352089	0.364381842778623	0.776234495135847
Rbmx	785.904394629512	-0.131436984345001	0.144882307343015	-0.907198309824112	0.36430193737065	0.776234495135847
Rcor3	114.392744690802	-0.208357301767057	0.22994202567475	-0.906129713155508	0.364867199950344	0.776234495135847
RGD1310212	228.399076767203	-0.15048882946848	0.166117727529947	-0.905916735715939	0.36497992547155	0.776234495135847
Scara3	12.6168061510923	-0.700080058356189	0.772457914228069	-0.906301878019843	0.364776091765544	0.776234495135847
Shfm1	2979.75976176909	-0.155904775788187	0.172135704953231	-0.905708527063259	0.365090147977073	0.776234495135847
Smarcd1	387.816731904859	0.129686534962737	0.142989684681236	0.906964269848171	0.36442569219086	0.776234495135847
Sqrdl	373.910260292457	-0.264452905719187	0.291621823984908	-0.906835099326699	0.364494005767862	0.776234495135847
Tma16	70.813538116593	0.246317270694935	0.271700413901082	0.906576722347621	0.364630675939956	0.776234495135847
Zfp644	392.279084950059	-0.132014116788096	0.145749754342351	-0.905758760169212	0.36506355342937	0.776234495135847
Ttc8	114.409706524965	-0.193177906217601	0.213478611639964	-0.904905202135188	0.365515610836682	0.77667928688105
Vrk3	1281.343321903	0.0905766975661968	0.100067845531881	0.90515286988306	0.365384406229647	0.77667928688105
Wdr55	183.686059734309	-0.185919907076299	0.205458149591742	-0.904904027636447	0.365516233109866	0.77667928688105
Pigq	1941.25681849883	0.109058551579384	0.120556949850283	0.90462268425687	0.36566531357525	0.776803369596003
Stard3	1035.86345520611	0.0857396285547715	0.0947885322630963	0.904535881163256	0.365711317148356	0.776803369596003
Adam15	841.972392974834	-0.135183522787596	0.149602566270644	-0.903617672861556	0.366198166874356	0.77701511860624

Ap1m2	437.645361335054	-0.154835232361919	0.17157874313513	-0.902415005103374	0.366836453144561	0.77701511860624
Cd320	826.806156861681	-0.126114702924511	0.139613559231031	-0.90331271274173	0.36635995133876	0.77701511860624
Cldn16	506.217155014211	-0.170957293732681	0.189251851075456	-0.903332214513026	0.366349604115415	0.77701511860624
Ggta1p	160.60779349885	-0.422110714271115	0.467722229593111	-0.902481617429911	0.366801082160894	0.77701511860624
Golga3	526.771110363667	0.107625726486234	0.119256568401721	0.902472106389077	0.36680613237036	0.77701511860624
Lrfn4	171.585312615995	-0.250322454221282	0.277374849472026	-0.90246990560883	0.366807300955395	0.77701511860624
Mpzl3	36.0892676837829	0.380130459484816	0.42092136671916	0.903091383665586	0.366477396592967	0.77701511860624
Numb	363.32180839423	0.115854923693889	0.128286146423979	0.903097699349348	0.366474044936144	0.77701511860624
Oaz1	8162.45970674114	0.107373665527133	0.118785461140762	0.903929357144924	0.366032861233931	0.77701511860624
RGD1309492	720.424988006518	0.127440566062309	0.141157570899532	0.902824873297192	0.366618847838696	0.77701511860624
Scarb1	211.986776363742	0.260507160844858	0.288488325291516	0.903007636727124	0.366521841885551	0.77701511860624
Tceal1	214.296074873015	-0.20376658862297	0.225558252700417	-0.903387866253822	0.366320077492607	0.77701511860624
Uba5	669.830844069337	-0.107125730691412	0.118704622055968	-0.902456272013599	0.366814540264818	0.77701511860624
Zrsr1	159.395495805878	0.161517131945056	0.178697881990433	0.903855883158721	0.366071824882648	0.77701511860624
Ppp2r3a	384.676044938603	-0.164637863105838	0.182502686387044	-0.902111998267693	0.366997375872434	0.777211137351608
Scrn3	112.015611524043	0.324798760951405	0.360126992389718	0.901900628986781	0.367109657214284	0.777304091635162
Krt14	53.1866696783719	-0.53751653724808	0.596071847115432	-0.901764677948273	0.367181886991647	0.777312223420946
Srrm1	2283.57717133439	-0.10078317182746	0.111786770284354	-0.901566183288923	0.367287361634022	0.777390717380316
Mef2bnb	309.682163578999	0.170919535612024	0.189665130333389	0.90116478085131	0.367500713613802	0.777697469918911
Zbtb48	224.013438257512	-0.189173238843209	0.209992604977433	-0.900856669993394	0.367664531953556	0.777899305693246
Ruvbl1	632.464147868792	0.141661995094919	0.157356070053724	0.900263936729947	0.367979808034908	0.778384317098671
Zfp93	217.2618240997	0.232287834276433	0.25804936028429	0.90016822370931	0.368030733775141	0.778384317098671
Sept8	1233.82946070933	0.115404450391059	0.128337607504188	0.899225508682584	0.368532555764105	0.778575664813203
Akr1e2	1723.1065009519	0.12795273173966	0.142571070686943	0.8974663031087	0.369470146508148	0.778575664813203
Baiap2	758.98917803996	-0.155466105646678	0.173084892651558	-0.898207251164609	0.369075068305544	0.778575664813203
Dll1	89.1234315386665	-0.309350798788431	0.344098552818593	-0.899018017525693	0.368643063706576	0.778575664813203
Ercc5	336.553929752238	-0.120759101848081	0.134261280693274	-0.899433561370239	0.368421769455915	0.778575664813203
Fer1l4	15.4718708887678	-1.0197980691506	1.13464295858068	-0.898783235235749	0.368768131525033	0.778575664813203
Fos	247.420227290513	0.590205662715418	0.657033520141736	0.898288511350376	0.369031755852369	0.778575664813203
Kmo	3580.10679893284	0.1911162338621	0.212944100917161	0.89749484977021	0.369454920379392	0.778575664813203
Mrpl43	913.471007290313	0.14534823084433	0.161826356940455	0.898174027966354	0.369092777497235	0.778575664813203
Ntng2	38.7489396465166	0.436500117243013	0.485633475144995	0.898826253920587	0.368745213626163	0.778575664813203
Prcc	851.321636693862	0.0823305793734785	0.0916718725807727	0.898100770232838	0.369131828442122	0.778575664813203
Samd10	141.330048479813	0.197625263990161	0.22020514832456	0.897459780090521	0.369473625789873	0.778575664813203
Scnm1	492.288895826003	0.122116202130438	0.135830375288389	0.899034563301222	0.368634250817425	0.778575664813203
Scyl2	1431.89769104075	0.111372771280503	0.12386508302568	0.899145817045257	0.368574996381985	0.778575664813203
Sema6c	36.5438285099998	-0.362321614006271	0.403733888304655	-0.897426806374166	0.369491213795743	0.778575664813203
Slc38a1	125.859606543051	-0.319349983014502	0.355756525980489	-0.897664440966617	0.369364472401883	0.778575664813203
Tpmt	5419.84128619306	-0.140156498574093	0.155978029386656	-0.898565644951551	0.368884064779908	0.778575664813203
Tuba1a	593.306067055961	0.24032856346595	0.26767253662721	0.897845428949097	0.369267961370237	0.778575664813203
Ube4a	2290.541649598	0.0703950762423625	0.0783100941512225	0.898927232885514	0.368691421280966	0.778575664813203
Wdsub1	480.230954794735	0.117983570078882	0.131176486026561	0.899426213132373	0.368425681977703	0.778575664813203
Sar1a	3057.83100580253	0.076494007919502	0.0852598853549089	0.89718638021952	0.369619471567256	0.778701559561341
Mms22l	37.1414619025679	0.374310561596337	0.417427061740788	0.896708900557039	0.36987426997587	0.779093949693429
Prim2	447.173228084948	0.215070630165282	0.23989768235973	0.896509828897724	0.369980533206916	0.779109718391904
Ril	189.535934353227	0.269144391370463	0.300237579406284	0.896438053832877	0.370018850948399	0.779109718391904
Rnft2	29.3276301271335	0.348581553561711	0.388910065761918	0.896303758244982	0.370090552436145	0.779116358397707
Osbpl9	2492.8161017378	0.114865383619584	0.128175635066423	0.896156149802253	0.370169371720522	0.779137977588047
Ssr2	1521.45036224443	0.0937100385586917	0.104652020014595	0.89544414475347	0.370549711379776	0.779649762966038
Unc5c	21.6370514003032	-0.492330317028911	0.549810414685779	-0.895454694706505	0.370544074023423	0.779649762966038
Lancl2	242.948698629853	0.142516141291391	0.159226072365532	0.89505530830541	0.370757522880623	0.779942625404619
Dpy19l1	607.184827932889	-0.127192926017974	0.142243006588602	-0.894194583399402	0.371217789851442	0.779942903309423
Fbxl12	142.518054039484	-0.192845668290895	0.215669601270274	-0.89417176623433	0.371229996002125	0.779942903309423
Gtf2h2	235.906934896577	0.136469205486827	0.152623348361179	0.894156804657939	0.37123799990357	0.779942903309423
Mnf1	589.177602103427	0.121842019109035	0.136230415478826	0.894381909361296	0.37111758835487	0.779942903309423
Slc9a8	202.146795755528	0.231436075555149	0.258642519106566	0.894810630342617	0.370888326753194	0.779942903309423
Ybey	427.344598372523	-0.148021159483726	0.165444763081355	-0.894686279135583	0.370954815394494	0.779942903309423
Zfp746	334.542538278495	-0.124042318010932	0.138652848024804	-0.894625099866261	0.370987529705955	0.779942903309423
Hsp90aa1	8251.51025046791	-0.187459012685697	0.209695514507183	-0.893958142720669	0.371344286994322	0.780022022912118
Pln	18.5213568457536	0.56376078073541	0.630978233412846	0.893471043662047	0.371604972120658	0.780337210556396
Slc27a2	11963.1945042948	-0.197226023492559	0.220753684208318	-0.893421209253495	0.371631648842317	0.780337210556396
Pex12	257.68194613382	0.169008791991204	0.18921632416853	0.893204075990149	0.371747895720536	0.780437122785299
Car3	128.774066385742	1.41253316862944	1.58207200535038	0.892837471273383	0.371944216469647	0.780560924001847
Wtap	205.127646337231	-0.147614232413227	0.165331335675675	-0.89283880644863	0.371943501352401	0.780560924001847
Arhgap21	699.099589151332	0.0894089524178683	0.100278029141518	0.891610586918188	0.372601693590155	0.780636729405779
Atf6	268.731596271301	0.22661263911291	0.25408229109471	0.891886790443177	0.372453615645506	0.780636729405779
Atf7	84.7434592558621	0.242030124016672	0.271469995969589	0.89155386455253	0.372632108039674	0.780636729405779
Cspg4	200.818625615421	0.337792466806703	0.378810013322478	0.89172000455844	0.372543028357079	0.780636729405779
Ehd2	204.399300449606	-0.355603681814548	0.398600800974378	-0.892129872657748	0.372323324838025	0.780636729405779
Elof1	971.189204040568	0.127524948564587	0.143052898271318	0.891453092566637	0.372686145624387	0.780636729405779
Epb41l4a	582.450918790411	-0.113266326885117	0.127107817586635	-0.891104332020463	0.372873201121236	0.780636729405779
Ip6k1	285.943303374082	0.201281076060686	0.225870365971468	0.89113538730491	0.372856542459083	0.780636729405779
Papss1	1507.3443197721	0.10236323413167	0.114734133979988	0.892177685757617	0.372297700589216	0.780636729405779
Pbx4	34.8183559150144	0.366279728312641	0.41098053475727	0.891233762516199	0.372803775114344	0.780636729405779
Rhbdl1	50.2928282246692	-0.340461874215379	0.3816997054615	-0.891962632781543	0.372412961512976	0.780636729405779
Serpinf2	14609.8538468587	0.103695197375216	0.116271449553649	0.891837142938258	0.37248022992512	0.780636729405779
Sox5l1	27.5813954987603	0.454368109948238	0.509342666878132	0.892067638340914	0.372356679418808	0.780636729405779
Spata24	326.720645076986	0.148109245104297	0.166274842974742	0.890749571339521	0.373063534403679	0.780891368698381
Rps27	5640.43667428808	-0.138374091770117	0.155396928352503	-0.890455771791245	0.373221206858979	0.781077561620172
Cmss1	155.012750236871	0.165188407768213	0.185812323050803	0.889006741081694	0.373999456937719	0.78113464022971
Etv6	344.118179511703	0.211847834358728	0.238240639950432	0.889217869809301	0.37388600079755	0.78113464022971
Kif1b	390.19055997767	0.243107840873742	0.273257919953863	0.88966439075138	0.373646119985034	0.78113464022971
Mis12	44.9607616713678	-0.286972179107163	0.322804284762325	-0.888997428638395	0.374004461739406	0.78113464022971
Pak2	138.186668906719	0.207635391216359	0.233319523129225	0.889918633604267	0.373509577733307	0.78113464022971
Sdhaf2	2742.39631444429	0.0875963605167682	0.0985045736409294	0.889261861444891	0.373862363298503	0.78113464022971
Slc15a2	7836.6491867458	0.148222282332095	0.166592577133495	0.889729211724241	0.373611304660344	0.78113464022971
Tesk1	732.576524124871	0.0961961631665249	0.108065541561596	0.890165003353028	0.37337729323695	0.78113464022971
Tmem37	1048.90313264005	0.132045937941207	0.148466262910988	0.889400294398018	0.373787986809819	0.78113464022971
Traf4	1677.55373290795	0.124182161655072	0.139597764725746	0.88957127572358	0.373696135552322	0.78113464022971
Tspo	817.622862244809	0.198949123371021	0.223779386143708	0.889041331283564	0.373980867431535	0.78113464022971
Psmf1	780.115139453684	0.0974291851357945	0.109643624187486	0.888598729363365	0.374218774204939	0.781438652877702
Map2k3	384.889332986013	-0.190599897770908	0.214554752287739	-0.88835085561421	0.374352051843306	0.781573369076233
Anxa8	82.5617563180787	-0.413079466633218	0.465405156229561	-0.887569596305605	0.374772314158593	0.782221118177633
H6pd	1816.94159965306	0.112739523279293	0.12703252703724	0.887485480362391	0.374817579977815	0.782221118177633
Rps28	3350.86585315831	-0.156563010426126	0.1764308208083	-0.887390364726798	0.374868769198801	0.782221118177633
Cpne4	154.991982405515	-0.29676802604654	0.334594956712543	-0.88694709855265	0.375107382654057	0.782575350816082
Med14	304.344310399444	0.172198316703748	0.194266516873695	0.886402450998311	0.375400698928787	0.782996906290556
Vps13d	1471.99074936194	-0.0877791282314544	0.0990381756791725	-0.886316085988993	0.375447223234518	0.782996906290556
Taf10	1054.82870574254	0.139892098054756	0.157945146900832	0.885700515651734	0.375778930385768	0.783544913367206
Gabarap	7056.30449645457	0.128883135074887	0.145587004342945	0.88526538241895	0.376013516063728	0.783602791268855
Gpsm2	217.8074018588	0.270266880821787	0.305293233781778	0.885269802654623	0.37601113260589	0.783602791268855
Neu3	116.306004736905	0.241776825696885	0.273061304546158	0.885430566951734	0.375924452406978	0.783602791268855
Hdac5	1020.54579485378	-0.131154403591418	0.148246214731543	-0.884706593209977	0.376314898756376	0.783705012321216
Mn1	22.5441880048621	-0.615426273172834	0.695726243457172	-0.884581081942065	0.37638261369578	0.783705012321216
Nradd	28.6112034585263	-0.444151625369858	0.502094287749222	-0.884598045042281	0.376373461446163	0.783705012321216

Slc2a1	1421.56308690568	-0.138545111192901	0.156577978945557	-0.884831392804437	0.376247575228796	0.783705012321216
Trappc1	803.746038985557	0.0929524920023638	0.105086245804256	0.884535281386931	0.376407325555298	0.783705012321216
Klhl21	292.290800631822	0.138512417755304	0.156678013349651	0.884057787011837	0.376665019042624	0.783820848666294
N5	51.6065338052863	-0.260243971913723	0.294300227607207	-0.884280566242246	0.376544776323013	0.783820848666294
Tmem161a	374.367327010188	0.115302387815937	0.130425358979681	0.884048843859425	0.376669846508472	0.783820848666294
Fdxr	188.21800086262	0.157189068017983	0.178020856911252	0.882981189649851	0.377246434776282	0.784733341447148
Map1b	130.493066957499	-0.430063163432294	0.487024795059059	-0.883041618815613	0.37721378539379	0.784733341447148
Prpf18	785.730929922046	-0.0937244656279429	0.106191839398496	-0.882595745198762	0.377454728245239	0.785022953565486
Prss53	168.415710577365	-0.34099808826626	0.386445503319995	-0.88239631548746	0.377562527513999	0.785103492082714
Nsg1	168.410168433001	-0.788106707211118	0.893533111112794	-0.882011754695493	0.377770450665858	0.785392160649011
Egfl7	349.922626036014	-0.303158312424439	0.343880001326486	-0.881581689121303	0.378003060754253	0.785588368720577
Scoc	2340.26095573104	0.159914628070267	0.181385188369668	0.881630024521939	0.37797691312757	0.785588368720577
Cfp	106.969492302517	-0.232496597022443	0.264033386682296	-0.880557568661573	0.378557333134622	0.786129821605643
Col5a3	44.3736835739633	0.551748647703894	0.626749461591049	0.880333660444223	0.378678582904355	0.786129821605643
Cyth1	163.803725600408	-0.199223768808724	0.226278115136899	-0.880437636172606	0.378622275463356	0.786129821605643
Gipc2	1233.7258237414	-0.171779424212734	0.195019492447239	-0.880832075076843	0.378408716273337	0.786129821605643
Mgst3	3488.79088347656	0.234988687466057	0.266895013750969	0.880453644163308	0.378613606888497	0.786129821605643
Prss30	1933.57067502493	-0.146699241015943	0.166523474859309	-0.880952317022478	0.37834362899953	0.786129821605643
Acot11	21.6859697182961	0.597942543613957	0.679969749047884	0.879366390124291	0.379202649415722	0.786253407009417
Bnip1	371.703087581842	0.135270510946192	0.153778266836286	0.879646478849986	0.379050851627742	0.786253407009417
Knstrn	48.4646957675956	0.306495351106035	0.348301824197746	0.879970559476667	0.378875258514185	0.786253407009417
Lpin1	68.6170784960549	0.302280333175044	0.343759100410711	0.879337689719021	0.379218206091949	0.786253407009417
Pigp	873.385631911779	-0.112713290881635	0.128109580862185	-0.879819371221638	0.378957168986883	0.786253407009417
Pmpcb	2695.5104242439	0.133255784919873	0.151444955575111	0.879895830229772	0.37891574381534	0.786253407009417
Prdm1	31.3567529684242	-0.34742895440367	0.395106425713234	-0.879330053355884	0.379222345348022	0.786253407009417
Prmt5	990.640974041381	0.11965277968012	0.136094324039363	0.879190080296899	0.379298222035008	0.78626729740104
Akap10	365.634020797936	0.112517377921976	0.12805027147875	0.87869691038217	0.379565634313139	0.786392949024684
Glo1	2041.43305050097	0.137460875496833	0.156437441779112	0.878695495998496	0.379566401403257	0.786392949024684
Ppm1f	138.182688185977	0.218614419422537	0.248734409738945	0.878907022361642	0.379451690800427	0.786392949024684
Il18bp	71.789639215367	-0.241615249511614	0.27505636726133	-0.878420855758835	0.379715370433589	0.786558210378745
Dcbld2	44.460888342153	-0.340838437389844	0.388213224159083	-0.877967096891512	0.379961574872446	0.78658784708966
Prr13	576.938218844067	-0.159612235194595	0.181791040435519	-0.877998359062196	0.379944609224777	0.78658784708966
Rnf111	600.049342773835	-0.114681585108064	0.130634051034172	-0.877884320360431	0.380006499064695	0.78658784708966
Tufm	3868.46069126212	0.102223176070431	0.116395306209294	0.878241394774295	0.379812732209796	0.78658784708966
Chchd3	2751.92311171428	0.0898545712390654	0.102392657393219	0.877548972032208	0.380188531551147	0.7868213491643
Pigw	152.430876535301	-0.175261305371007	0.199930265610857	-0.876612176928398	0.380697322924817	0.787730888833693
Cops2	1720.55599666481	0.0937274088402478	0.106949194024521	0.8763732134228	0.380827175499817	0.787856147930638
Col14a1	15.9772453574397	0.462596432741042	0.528105711272352	0.875954232016389	0.381054915300909	0.788040423455264
Rps8	9392.13635194114	-0.13592487618991	0.155157001030824	-0.876047328105466	0.381004305151389	0.788040423455264
Clec12b	29.9487652272941	-0.453563241511052	0.518024373744767	-0.875563514960252	0.381267367177595	0.78833634625078
Clock	68.7461862275129	-0.333743484487616	0.381300585537786	-0.875276611539697	0.381423416815504	0.788474884611232
Cpm	588.650543695936	-0.209255199210416	0.239133015900998	-0.875057751527914	0.381542483315307	0.788474884611232
Mcm3ap	1088.49609760892	-0.0849801347035107	0.0971120110848185	-0.875073368929496	0.381533986218452	0.788474884611232
Myadml2	32.354771287076	-0.338913610912716	0.387390039169013	-0.874864030163803	0.381647892653085	0.788549345190869
RT1-M6-2	14.2210982595929	0.50284806347648	0.575222682907991	0.874179823602179	0.382020332002864	0.789175407768912
Abhd11	886.242932324312	0.156475456195211	0.179024029808127	0.874047223509139	0.382092536996004	0.789181133108592
Capn7	815.333847171218	-0.108702181313294	0.124387914325232	-0.873896647459458	0.382174540620313	0.789207090968474
Ube2a	596.202975635049	-0.128738287506541	0.147340574899827	-0.873746336296481	0.382256410750422	0.78923276377644
Ppp2r4	2129.23719328189	0.112876570936854	0.129227302458996	0.87347308803161	0.382405268695002	0.789396709768869
Exoc3	747.52778735002	0.0832813967718593	0.0953626374412424	0.873312641160676	0.382492692180966	0.789415221726362
Idi2	21.5351903598773	-0.624923583715617	0.715669219019541	-0.8732017070285	0.382553144577582	0.789415221726362
Dok1	71.5180177988953	-0.233134882910681	0.267163299239169	-0.872630647901882	0.382864429944887	0.789615905099675
Myo1e	929.951851690171	0.116159270658162	0.133131768132899	0.872513542689567	0.382928283381085	0.789615905099675
Ptplad1	1122.97892637907	-0.128457717636752	0.147195213586566	-0.872703089365101	0.382824933378258	0.789615905099675
Zfp949	91.9411836209176	-0.217293360775474	0.248941889386554	-0.872867805859956	0.382735135871424	0.789615905099675
Atp5i	5478.23790050455	-0.128336190782277	0.147142909414786	-0.872187394504385	0.383106155135557	0.789696147854685
Socs4	43.5491188635271	0.347896416371387	0.398832734996978	0.872286514731605	0.383052092487438	0.789696147854685
Dpep2	91.3767469444236	-0.330778005655974	0.379596729923733	-0.871393190669563	0.383539502498874	0.790050506771685
Fam227b	53.436616016278	-0.290220637740247	0.333113904770428	-0.871235435039128	0.383625615596774	0.790050506771685
Hmg20a	534.696996727906	-0.0992578530437244	0.113901083115819	-0.871439062109665	0.383514465158685	0.790050506771685
Synrg	782.594359181735	0.157622813359584	0.18083435541663	0.87164196756989	0.383403728230744	0.790050506771685
Ube2j1	252.419970329871	0.137285167132539	0.157554257263708	0.871351682377943	0.383562159227811	0.790050506771685
LOC100360880	46.8068810527712	-0.483418488441016	0.554948918072809	-0.871104479525434	0.383697108470413	0.790054589651217
Sin3a	713.932861851379	-0.0949637058992982	0.109039642563607	-0.870909915574072	0.38380334231929	0.790130191777042
Car12	1908.46649024592	-0.11726467738142	0.134705240148553	-0.870527956092139	0.384011948349054	0.790416480430161
Ddx58	1162.2818208689	-0.405568368354891	0.466135880661577	-0.870064685385891	0.38426505535917	0.790651089647416
Gnb5	73.4753903780115	0.228219333422111	0.262289671872771	0.870104155427107	0.384243487005513	0.790651089647416
Sars2	284.740839339876	0.129314249617688	0.148649539532403	0.8699270110386	0.38434029312826	0.790662764107838
Rbmxrtl	556.653809285194	-0.13670834658238	0.157262408030299	-0.869300860228727	0.384682592495601	0.79122373259229
Srprb	734.691909734195	0.103986595305527	0.119640776575447	0.869156806583846	0.384761369019218	0.791242576492207
Ndufa10	2445.20455187664	0.136402941717261	0.156971791003802	0.868964677315541	0.384866451311401	0.79131550029041
Tut1	393.602489259341	0.132926797480362	0.153023395852417	0.868669766083107	0.385027782829763	0.791504029597231
Dctn2	3375.07113413686	0.0749754633736711	0.0863537008551434	0.868236828661704	0.385264696609133	0.791561558506762
Siva1	92.1244430920333	-0.243983841625775	0.280971170099663	-0.868358990494404	0.385197837692285	0.791561558506762
Ugt1a1	10926.0418053291	0.142875299240965	0.164549802729268	0.868279978895124	0.385241079766072	0.791561558506762
Cyp2d3	138.819360473949	-0.255004620262422	0.294005822070259	-0.867345477945955	0.385752745636487	0.792421056733113
Fads6	85.6433580165894	0.347419595463455	0.400694435256551	0.867043724330769	0.385918052950199	0.792617381610402
Brix1	541.212067662999	-0.130826677635154	0.151055621438353	-0.866082813664405	0.38644474926716	0.792839534326813
Cdr2	1171.38592475988	0.181001519870967	0.208889757082171	0.866493036323301	0.386219843535531	0.792839534326813
Hddc2	278.251172758076	0.15398335653612	0.177759200675401	0.866246900025738	0.386354778865107	0.792839534326813
Rassf7	633.116575502634	-0.115374115076929	0.133134082515371	-0.866600895105946	0.386160722923511	0.792839534326813
Slc22a1	2025.18520321014	0.16472871658796	0.190173935680729	0.866200281328314	0.386380339122145	0.792839534326813
Syf2	1808.60902907015	-0.0904645557047292	0.10442485784227	-0.866312462128246	0.386318834000429	0.792839534326813
Cblc	118.807779466541	-0.289508253617715	0.334556871360477	-0.865348400827725	0.386847592789175	0.793487191956145
Cnot7	938.991607642381	-0.127769509716306	0.14768897466859	-0.8651255789609	0.386969866708861	0.793487191956145
Mtmr4	1297.71499072734	-0.0753944548371513	0.0871415190829375	-0.865195553515588	0.386931465500911	0.793487191956145
B3gat1	92.5573598075077	0.244203290060717	0.2823862715328	0.864784568793572	0.387157042324884	0.793645375503826
Jph2	7.65136571960021	0.805924096507851	0.931994408210104	0.86473061362635	0.387186662605025	0.793645375503826
Rbm42	1232.19955870118	0.101918061884875	0.117881293591029	0.864582146837179	0.387268174960565	0.793669325027368
Grasp	68.9404806153931	-0.41532777356224	0.480453394600546	-0.864449659904158	0.387340922770051	0.793675307049648
Utp14a	307.485179273203	0.141051111008414	0.163195494518862	0.864307629473875	0.387418920112805	0.793692041456766
Auh	1416.23216513467	0.138113364310235	0.15992208558752	0.863629084143291	0.387791681785693	0.794037789367246
Mitf	64.0610205791922	-0.263246081362172	0.30474261812056	-0.863830871394658	0.3876808062978	0.794037789367246
Ube3a	607.336067267585	0.121427043759526	0.140602575543373	0.863618915160399	0.387797269820305	0.794037789367246
Pdrg1	792.793770214132	0.138008109824489	0.159846990651481	0.863376340474196	0.387930583416296	0.794167689320896
Kif18b	44.5591773455413	0.456739277474909	0.529313196284341	0.862890403415437	0.388197727497479	0.794556369737091
Vstm5	193.387503514136	0.224793329178309	0.260546363062075	0.862776691781156	0.388260256688352	0.794556369737091
Aplnr	127.265853002702	-0.445417620878728	0.51674596397358	-0.861966327619932	0.388706047610802	0.794738310835305
Crcp	710.733947982286	-0.133637637914911	0.154965883098025	-0.862368124152705	0.388484975640486	0.794738310835305
Crlf3	93.4334350831541	-0.242321578553359	0.281115499363836	-0.862000064392509	0.388687482398019	0.794738310835305
Eif1ad	546.293484409304	-0.133133003365813	0.154566394728089	-0.861332138852164	0.389055139644236	0.794738310835305
H2afy	1635.96147536597	-0.0778584384577303	0.0903503642672719	-0.861739065350214	0.388831123208408	0.794738310835305
Kars	1666.48971078407	0.101128716732736	0.117359185430693	0.861702612893966	0.388851187387451	0.794738310835305
Mboat7	293.226068438675	0.134306741119983	0.155949866331584	0.861217417361661	0.389118308973999	0.794738310835305
Mtf1	219.351290323552	0.158094222572886	0.183395992422815	0.862037498662478	0.388666883098904	0.794738310835305

Polm	252.608994385182	-0.192504237421623	0.22342905225925	-0.861590001278151	0.388913175109771	0.794738310835305
Ptprq	95.125449944277	-0.406788461179484	0.472024673269543	-0.861794910765593	0.388800385969585	0.794738310835305
Tnpo1	1815.23452428567	-0.0967174383926026	0.112282972136385	-0.861372268228918	0.389033044596666	0.794738310835305
Nckap1	4025.58396113775	0.0928794038864693	0.107883768634982	0.860921017699342	0.389281545022223	0.794928861071252
Fkbp8	4187.06888642906	0.0880915378988871	0.102380984620936	0.860428703875475	0.389552768847434	0.795178198496994
Mcm7	470.03787606475	-0.110110143981682	0.127987659905681	-0.860318440565488	0.389613530479479	0.795178198496994
Usp48	799.882818921013	-0.0940387985804007	0.109289403967757	-0.860456688080619	0.38953734880562	0.795178198496994
Scarb2	321.641258370241	-0.26345241170309	0.306447202151367	-0.859699190769444	0.389954880976768	0.795731988722072
Chm	175.298409738279	0.219151162899825	0.255022694411278	0.859339845835041	0.390153046863566	0.795786161474426
Cx3cl1	2270.17102571727	-0.119122417286723	0.138632085898033	-0.859270179158524	0.390191472628627	0.795786161474426
Fam117a	386.226068114448	-0.168021769786017	0.195534469596204	-0.859294886129267	0.390177844842146	0.795786161474426
Edn3	34.8741507906428	-0.764272922039342	0.889792545509249	-0.858933833393638	0.390377021853591	0.79588516548545
Gsk3a	950.598522884767	0.0905128929557739	0.105394438475557	0.858801415567722	0.39045008646374	0.79588516548545
Scaf11	1445.56315282461	0.0918849233042226	0.106991263783038	0.858807719951337	0.390446607685305	0.79588516548545
Psmc1	3081.34513845111	0.108548061402195	0.126446204328085	0.858452509341831	0.390642643366359	0.79613489053291
Bcs1l	191.119952104875	-0.152646598517679	0.177888301901212	-0.858103635181416	0.39083524024767	0.796167877481405
Best3	28.6206802798466	-0.387429007714047	0.451526900779875	-0.858041917424813	0.390869317697883	0.796167877481405
LOC691931	40.7232308067726	0.325311738052586	0.379065362983428	0.858194311113589	0.390785176818608	0.796167877481405
Ndrg2	501.556745487767	0.274098768574073	0.319520534137605	0.857843985876756	0.390978617747246	0.796167877481405
Reep5	4081.35566034691	-0.0939696111205012	0.109566639532856	-0.857648016961606	0.391086852293395	0.796167877481405
Shisa7	125.651213854291	-0.204873986089	0.238870561740291	-0.857677834373527	0.391070382824187	0.796167877481405
Spats2l	141.035227063098	-0.224456579717937	0.261807610897764	-0.857334051322087	0.391260294902444	0.796167877481405
Wdhd1	27.7933169525799	-0.39409981374886	0.459708693006089	-0.857281621480322	0.391289263031069	0.796167877481405
Zmynd11	833.423187461267	-0.103154430919832	0.120314130043601	-0.857375861691803	0.391237195095162	0.796167877481405
Cndp2	29743.1677799284	-0.109712984135681	0.128060690393433	-0.856726477099388	0.391596066967321	0.796649525174615
RGD1565983	116.833102010618	-0.200398199375427	0.234067886769872	-0.85615417877657	0.391912503921512	0.797150594053669
Rpl31	6750.66479499655	-0.132923551453547	0.155299689028225	-0.855916404503484	0.392044020409496	0.797275422432337
Naa20	997.082831373562	-0.138464606585604	0.161801740475488	-0.855767102249318	0.392126615388876	0.797300735332731
Cdc42	2439.3996634563	0.0927592144217391	0.108441486622728	0.855384939017406	0.392338078656569	0.797546419832526
Slc24a6	573.154242247087	-0.190967793536216	0.223277075038421	-0.855295123797887	0.392387786354345	0.797546419832526
Cenpk	15.8904785458397	0.421181060898797	0.492528254233691	0.855140912787021	0.392473142425798	0.797577281747116
Cryzl1	441.984297671281	-0.126765219804591	0.148275117451434	-0.854932519922721	0.392588506134574	0.797669102739644
Ppfibp2	628.493569896789	0.18190200612198	0.212875440509397	0.854499728511189	0.392828159771654	0.798013380511996
Acsl3	911.614762286402	0.110862984390643	0.130112276439643	0.852056296486904	0.394182846884652	0.798019710349485
Amph	137.134083588198	-0.26933342038226	0.315253297607001	-0.854339740223797	0.392916774024728	0.798019710349485
Cldn19	706.933458561743	-0.220560020652981	0.258443358632235	-0.853417250960731	0.393427958294816	0.798019710349485
Ldhb	7041.95770670888	0.185214261951794	0.217551209415702	0.851359376255559	0.394569750838532	0.798019710349485
Lpin2	3049.67210731138	-0.240915270262638	0.282189755133577	-0.853734998808155	0.393251837396522	0.798019710349485
Lrch4	748.322199677265	-0.117243566709179	0.137718029319537	-0.851330557723476	0.394585754750274	0.798019710349485
Mmp14	212.668661407283	-0.257282265682767	0.302112356159811	-0.851611198406826	0.394429922133391	0.798019710349485
Mpped1	293.050417944915	0.171791461060067	0.201413739308867	0.852928214577388	0.393699114126138	0.798019710349485
Obfc1	288.893144425757	-0.156125643149801	0.1829598575856	-0.853332775889136	0.393474789074422	0.798019710349485
Pdxdc1	497.24927004597	0.095899187682835	0.11251860460724	0.85229627595883	0.394049672448176	0.798019710349485
Pigv	175.306949628772	-0.271782941295135	0.319187530580807	-0.851483580203112	0.394500780651915	0.798019710349485
Pik3ip1	112.655552924263	-0.342408802355855	0.401612968895586	-0.85258402709818	0.393890023465401	0.798019710349485
Ppp1r8	433.478082051327	-0.104019511692496	0.122023929848565	-0.852451743044067	0.393963411933329	0.798019710349485
Rpl37	4655.46306970658	-0.13722281668527	0.160845657139939	-0.853133489117981	0.393585281846326	0.798019710349485
Rps12	7837.65667914143	-0.134826213489032	0.15818957157684	-0.852307849026197	0.394043250767543	0.798019710349485
Sema5b	16.445258680203	-0.589820829801946	0.691133152837823	-0.853411281719182	0.393431267376017	0.798019710349485
Sidt2	2680.37264062425	0.10622898321031	0.124412828136579	0.853842684885301	0.393192160123866	0.798019710349485
Snrpd1	479.769762411732	-0.134761992072774	0.158275139973738	-0.851441307176441	0.394524253954523	0.798019710349485
Snx7	2824.86274567718	0.169492328561994	0.19848022089723	0.853950725144322	0.39313229208534	0.798019710349485
Stub1	1338.14935413072	-0.0907501169448289	0.10643933419137	-0.852599442060269	0.393881472100425	0.798019710349485
Thpo	217.83859827155	0.182987113722088	0.214868761428983	0.851622695198381	0.394423539052133	0.798019710349485
Vars	803.53111346277	0.108215210184716	0.12690547864936	0.852722918950681	0.393812978034054	0.798019710349485
Zbtb33	56.8433771266992	0.320490799974154	0.376169734880607	0.851984543828	0.394222670662249	0.798019710349485
Zfp238	194.833979253055	0.211300300519922	0.248200485452686	0.851329118613678	0.39458655394722	0.798019710349485
Zfp503	546.670619013968	0.201538555268119	0.23622517925796	0.85316288424968	0.393568982796629	0.798019710349485
Hsd3b1	66.7233454141938	-0.395860521442407	0.465108007106794	-0.85111525794806	0.394705330467618	0.798117912487981
Ube2l3	3294.45012441749	0.0784752515878957	0.0922226977425613	0.850932075387325	0.394807085803247	0.798181667954413
Aamdc	265.092427290889	0.135060230071667	0.159183953977697	0.848453796358076	0.396185293539243	0.798436730078274
Arhgap5	338.114212974527	0.235255628045763	0.277282997380834	0.848431495143755	0.396197708749029	0.798436730078274
Calm3	2211.29364081128	0.114064530930976	0.134334963316721	0.849105311936146	0.395822694886052	0.798436730078274
Dcdc2	377.653656115907	-0.185581959955954	0.21860098130535	-0.848953004912299	0.395907442853961	0.798436730078274
Dync1h1	4594.9681793324	-0.0772024109736045	0.0909831751163047	-0.84853502721702	0.39614007385541	0.798436730078274
Fstl1	612.329871713077	-0.58282198694129	0.686354662190349	-0.849155719408014	0.395794649146118	0.798436730078274
Kif16b	999.58519616974	0.138865735719099	0.16364008105039	0.848604662303598	0.396101311800014	0.798436730078274
Kremen1	59.6242493464416	-0.438103812625386	0.515170717718268	-0.850405113407421	0.395099894216938	0.798436730078274
Lifr	267.621516519565	0.309388301742942	0.36428301973347	0.849307502637119	0.395710207150086	0.798436730078274
Mthfd2l	21.4271513229218	-0.384008285285252	0.452469389204514	-0.848694507180643	0.396051303440423	0.798436730078274
Ndufb8	4993.68876443704	0.150930955115368	0.177660539463239	0.849546869391323	0.395577061680516	0.798436730078274
Ndufb9	5278.14122984861	-0.130517809286331	0.153597119435649	-0.849741256645197	0.395468955547965	0.798436730078274
Nr1d1	845.315728426754	0.367470483152503	0.432331526303019	0.849973829794092	0.395339636250631	0.798436730078274
Plaa	888.298755419364	0.132024521165079	0.155387956996031	0.849644488011714	0.395522770027436	0.798436730078274
Rnf41	335.014170578176	-0.112275010117197	0.132260640892386	-0.848892076733163	0.395941348097749	0.798436730078274
Skiv2l	1264.64807149153	0.0747609318030529	0.0879101704972632	0.850424147515223	0.395089315558223	0.798436730078274
Xrcc6	615.995460742784	0.115881401456479	0.136400987938299	0.84956423855887	0.395567401300544	0.798436730078274
Yars	753.576746432637	0.103612028287043	0.121901843101572	0.849962770462055	0.395345785070619	0.798436730078274
Pdpk1	101.298495338374	0.250311023928446	0.295135933182216	0.848121139400212	0.396370509895386	0.798643363848779
Nudt16l1	764.264948619281	0.0905467331802712	0.106809013193168	0.847744309897464	0.396580383723308	0.798852747351389
Tmem185a	525.60401030125	-0.0957891703063581	0.113001279599835	-0.847682173560962	0.396614996771414	0.798852747351389
Ubald1	302.566391314804	0.206050695977841	0.2431493780402	0.847424318493523	0.396758654425533	0.799000507831433
Blvrb	759.589946693403	0.134825933639252	0.15914336544802	0.847197954245156	0.396884793616334	0.79911294317861
Pcsk4	80.2537698613608	-0.250776441916698	0.296138650632212	-0.846821046092184	0.397094875553309	0.799293945667612
RT1-DMb	190.949735411016	0.300979362272441	0.355438033756872	0.846784344070276	0.397115336194352	0.799293945667612
Rbm6	800.306182047284	-0.087581167637763	0.103452367923176	-0.846584465836498	0.39722677546182	0.799376687305071
Rragd	212.646529019643	0.171227547233093	0.20230051690941	0.846401926445733	0.397328564179628	0.799439982380183
Uggt1	403.51835564849	0.214461939026251	0.2534414561517	0.846199127335676	0.397441668687462	0.799526018814459
Acta2	405.914168806434	0.836716560435185	0.98961875353025	0.845493840380834	0.397835170334112	0.800175994694304
Ebpl	248.606560701505	0.140329233604884	0.166076647712739	0.844966679768303	0.398129443013232	0.800369594912708
Fam83b	13.9720649443709	-0.52248855473737	0.618214007237274	-0.845158066010685	0.398022591831927	0.800369594912708
Nlk	672.281815350177	-0.12109904006867	0.143322145698004	-0.844942974296795	0.398142679010773	0.800369594912708
Agk	230.782666228165	0.151613865912191	0.179507605488557	0.844609706087668	0.398328788045871	0.800497318297427
Dcps	291.715242088221	-0.130996583354442	0.155103191692573	-0.844576967920089	0.398347073050347	0.800497318297427
Hbb-b1	756.156485132476	0.178195040975924	0.211032355595885	0.844396777322415	0.398447722609754	0.800558036977631
Plekho1	171.528121291469	0.311263195293419	0.368697533166084	0.844223699086175	0.398544413818213	0.800610782512868
Grem2	426.716985271047	-0.522826903277319	0.619821659439722	-0.843511831693523	0.398942251420012	0.800702389129411
Rpl5	11796.8698312989	-0.122651304966211	0.145381173955645	-0.843653284871883	0.39886317919716	0.800702389129411
Slc39a12	14.715090648796	0.722663962826148	0.856564973419754	0.843676761543239	0.398850056668064	0.800702389129411
Tbx2	142.164419604081	0.282205472775049	0.334531475713236	0.843584216323365	0.398901787286286	0.800702389129411
Zfp524	267.819720848004	0.151786492744253	0.179930899294271	0.843582138140773	0.398902948988223	0.800702389129411
Id4	2650.58434100327	-0.169895518765995	0.20146327322169	-0.843307646347244	0.399056407518238	0.800790100256407
Diaph1	1723.33980639243	0.0772081149342368	0.0916072537727442	0.842816608450808	0.399331017838656	0.801199708517946
Ttll1	125.981157550117	-0.169748869706933	0.201493438447212	-0.842453585660577	0.399534109479046	0.801465705672226
Cd1d1	247.607258627133	-0.220748748487405	0.262113944702585	-0.842186205460696	0.399683733984459	0.801482942919436

Cyp2t1	292.199487543889	-0.147529021411525	0.175151151423045	-0.842295470014902	0.399622586062602	0.801482942919436
Acp6	366.523320356742	-0.127488398724211	0.151774722815155	-0.839984395026553	0.400917136093693	0.80166158931745
Ccne1	22.5561931870052	0.458923129313705	0.546251421881094	0.840131688322822	0.400834554592239	0.80166158931745
Cd3eap	205.839842380583	-0.172817829431304	0.205599363327455	-0.840556248007732	0.400596578016136	0.80166158931745
Crk	216.084001352968	0.165240406914288	0.19634633231258	0.841576234035418	0.400025196790992	0.80166158931745
Eif2b5	1161.16075417522	0.0902045974023867	0.107393637386751	0.839943590676027	0.400940015279921	0.80166158931745
Galnt6	68.1493680071988	0.28777138384315	0.342708841504096	0.839696409874244	0.401078627449993	0.80166158931745
Gmeb1	186.194670537795	-0.173196906887201	0.205898921808401	-0.841174423673619	0.400250226551838	0.80166158931745
Insig1	809.810002483689	0.206529269712044	0.245974430793042	0.839637148650681	0.401111863784372	0.80166158931745
Ldha	6927.28600452899	-0.100572635969021	0.119492982178186	-0.841661444343643	0.399977485411792	0.80166158931745
LOC500300	812.425360489559	-0.219380759523137	0.261112124845818	-0.840178370317647	0.40080838398418	0.80166158931745
Mid2	35.5172474305964	0.386461712129053	0.460154372211661	0.839852309283914	0.400991200013767	0.80166158931745
Mvb12b	51.3187876446466	0.362900813631666	0.432058568582107	0.839934305255426	0.400945221767306	0.80166158931745
Myd88	453.917604171671	-0.113946108082338	0.135361529660871	-0.841790931055621	0.399904989119098	0.80166158931745
Ndufaf1	243.914610382742	0.144193765986146	0.171566745471534	0.840452883744134	0.400654508513678	0.80166158931745
RGD1308139	401.285859464756	-0.106360692371406	0.126491823640817	-0.840850335697788	0.400431784112845	0.80166158931745
Sec24c	2722.67441875417	0.0773827639165991	0.0919905204901434	0.841203675164448	0.400233841987712	0.80166158931745
Slc35a4	1375.74827406446	0.135654012232965	0.16146226744493	0.840159217256329	0.400819121345902	0.80166158931745
Tgds	490.96093240256	0.127775056149545	0.15193122825633	0.841005878883374	0.400344640975798	0.80166158931745
U2af1l4	422.351125127257	0.111310427461314	0.132569976804524	0.839635263913806	0.401112920855916	0.80166158931745
Pgm5	101.301958276241	-0.263545169604729	0.31406297613855	-0.839147526540872	0.401386528952477	0.801926399731738
Wisp1	15.1828989192763	-0.535520704355423	0.638124523697324	-0.839210349184812	0.401351280783528	0.801926399731738
Rab12	90.3624998458634	-0.258655170306076	0.308307342927475	-0.838952351410329	0.40149604855018	0.802004233360518
Cryz	19145.7279689096	0.436077277087446	0.519896565074014	0.838776992160672	0.401594464059145	0.802059862677252
Chd1	564.021581732467	-0.124783619199523	0.148804373431901	-0.838574944550465	0.40170787561852	0.802145417125808
Cyp21a1	85.0797993079469	-0.226129750266923	0.269792843962697	-0.838160667812928	0.401940473830902	0.80246889611137
Bard1	17.441561412075	0.506893683937502	0.605183229069715	0.837587129961774	0.402262623498162	0.802574242667376
Diablo	1302.09951876143	0.104840500124146	0.125153835869266	0.837693063068888	0.402203110424638	0.802574242667376
Gpr4	49.1679936073177	-0.330228634490288	0.39427277513054	-0.837563877903953	0.402275687178958	0.802574242667376
Tex264	6273.88947821175	0.122173264862171	0.145839913363278	0.837721732306885	0.402187004994248	0.802574242667376
Phka1	119.476744504984	0.249525029912986	0.297995744632483	0.837344272216785	0.402399080574584	0.80267952787131
Impad1	625.502461186846	-0.111899597269297	0.133732184059425	-0.836743959999734	0.402736503494343	0.802789038708647
Mrps18c	750.826726379855	-0.144321843749839	0.172474101469864	-0.836773999805739	0.402719614718988	0.802789038708647
Pacsin1	26.1134408474747	0.465253548717647	0.556020436104999	0.836756202661926	0.402729620456849	0.802789038708647
Plekhg5	427.998887182083	-0.138108580905339	0.164980572511038	-0.837120266970213	0.402524969400553	0.802789038708647
Ankzf1	887.181433760993	-0.0870924807452228	0.10425032292475	-0.835416891783522	0.403483022312622	0.803572460417954
Csnk1a1	540.344222551637	0.14971727962512	0.179202236985008	0.83546546150339	0.40345568567182	0.803572460417954
Fnta	1049.62714407247	0.0909113432987509	0.108787779678274	0.835676061848211	0.403337165682222	0.803572460417954
Tarbp2	598.894321560995	0.125094970502463	0.149675054000576	0.835777019342056	0.403280357021186	0.803572460417954
UST4r	117.212061492709	0.259621894830762	0.310686400734698	0.835639713282652	0.403357620150086	0.803572460417954
Psmd10	186.980399479154	0.187039498781934	0.22400097010496	0.834994146205232	0.40372100431343	0.80371006270466
Tmpo	851.297682923348	-0.0925639647453158	0.110854627136333	-0.835003167089067	0.403715925185475	0.80371006270466
Wdr46	659.909924391493	0.114872916593673	0.137585974931429	0.834917342780931	0.403764249343974	0.80371006270466
Acot13	446.308107385258	-0.139861593860975	0.167546083194387	-0.834764926725905	0.403850077127601	0.803740146494889
Lyn	162.565898176717	-0.206518297870343	0.247762492530751	-0.833533339775839	0.404544002942582	0.804135560938332
Mrpl20	1374.74727941796	0.156953640185649	0.188255015967447	0.833728861773275	0.404433790353054	0.804135560938332
Mvb12a	1261.98054244095	0.092711721932924	0.111212780555023	0.8336426934947	0.404482359804943	0.804135560938332
Rab36	17.4142377700572	0.592486180570844	0.710203453648812	0.834248520655353	0.404140954367592	0.804135560938332
Rnf135	129.03603543705	-0.193212529843762	0.231782109777239	-0.833595526546266	0.40450894731714	0.804135560938332
Sema4d	306.322439914111	-0.147190332110427	0.176488219136182	-0.833995225465169	0.404283674352801	0.804135560938332
Tm2d3	163.372313085497	0.186407017900255	0.223481858153623	0.834103579772984	0.404222618080864	0.804135560938332
Ccdc91	1109.34891814863	0.0873874093841855	0.104886420010814	0.833162285214575	0.404753209792386	0.804398337873906
Rab5a	327.66243411798	0.148121581804053	0.177806811942894	0.833047846623696	0.404817745261195	0.804398337873906
Slc39a8	3323.7648528164	-0.190664370874537	0.228967336277704	-0.832714281321285	0.405005888177678	0.804517003031337
Zcchc17	428.198242737481	0.14201410568193 4	0.170548388433821	0.832690985743561	0.405019029680214	0.804517003031337
Slc7a5	252.498265212821	0.590451410400912	0.709698818853561	0.831974627426772	0.405423266209249	0.805179249298326
Abt1	385.368814940282	-0.137059541177145	0.164910857965438	-0.831112898617445	0.405909853707373	0.805723261829899
Nfyc	1202.74991052717	-0.0877652755591229	0.105581065184758	-0.831259614643415	0.405826983777155	0.805723261829899
Tmprss2	3017.31776623574	-0.10306308311757	0.123989792944113	-0.83122231814698	0.405848049079917	0.805723261829899
Exo5	131.792775085175	-0.246367988061458	0.296560896710122	-0.830750077958776	0.406114829878806	0.805989376008819
Birc6	2670.5771500343	-0.0946313708378394	0.114036286292165	-0.829835606847024	0.406631736071549	0.806069862214206
Dtwd2	62.7964387691848	-0.26237122960214	0.315984433538142	-0.830329604102062	0.406352454625989	0.806069862214206
Gyg1	617.880995193012	0.093505853983582	0.112684796240864	0.829800089301426	0.406651820335761	0.806069862214206
Prr3	526.464028873005	-0.0986264559645044	0.11884461941547	-0.829877334368123	0.406608140981162	0.806069862214206
Sctr	64.6021418182901	-0.246985749161999	0.297487481680777	-0.830239133984906	0.406403593358226	0.806069862214206
Shroom2	639.474717425048	-0.107425253889395	0.129408119587144	-0.830127616660512	0.406466634425999	0.806069862214206
Zfp583	23.0390345167843	-0.37501974025928	0.451923203836248	-0.829830681575639	0.406634521151277	0.806069862214206
Snx24	516.188786026144	-0.126904544274828	0.15296941364767	-0.829607313309858	0.406760840556921	0.806145372932862
Sult4a1	652.997173710404	-0.470527560707642	0.567444031512931	-0.829205233603589	0.406988283999923	0.806455515331785
LOC688298	289.398362436824	-0.138489617825268	0.167082403748619	-0.828870154595273	0.407177885262035	0.806690577634355
Cltc	10294.8136018928	0.100410972611913	0.121168186070303	0.828690895427393	0.407279338977739	0.806750952739802
Abhd8	312.765651157373	0.171997837202705	0.207636956840271	0.828358495617029	0.407467504225243	0.806757676905263
Ccdc93	289.707911064257	0.180379998154696	0.217854803481978	0.827982652994925	0.407680324045401	0.806757676905263
Cenpt	20.3669640555053	0.462694193611174	0.558700497563075	0.828161413188893	0.407579093331274	0.806757676905263
Eif5b	3624.30567910175	0.109155961849668	0.131759530055127	0.82844832403393	0.407416648947154	0.806757676905263
Snd1	1561.65691028771	0.0814567303477874	0.0983474412895341	0.828254698645178	0.407526272345488	0.806757676905263
Thsd1	54.5185869147913	-0.35205926383334	0.425226908603866	-0.827932703010835	0.407708613069139	0.806757676905263
Ankh	113.247592426116	0.180881879427303	0.218770760217586	0.826810124202159	0.408344690770666	0.806973167131658
Atp6ap1	2926.69091075601	0.0675222517117763	0.0817019346571344	0.826446178969156	0.408551036898844	0.806973167131658
C1galt1	86.9439354584163	0.232836790867978	0.28159908797632	0.826837872740404	0.408328960721416	0.806973167131658
Cab39	2827.22405487862	-0.081963354012614	0.0992228393165814	-0.826053301610336	0.40877385635452	0.806973167131658
Cbx5	423.760623926811	-0.129107382008606	0.156099480766971	-0.827083993964985	0.408189455692343	0.806973167131658
Cnot1	2057.46548580706	0.0739025417015148	0.0894718038457657	0.82598694253343	0.408811498886511	0.806973167131658
Grpr	29.9449268300978	0.330555167849401	0.400055175133311	0.82627394518581	0.408648709973625	0.806973167131658
Hoxa11	82.5887369529887	-0.220260905984782	0.266410261044564	-0.826773357456895	0.408365533603316	0.806973167131658
Krtcap3	158.675554917008	-0.166294192757318	0.201286706778941	-0.826155862045806	0.408715682472218	0.806973167131658
Lrrc14b	34.1259418388637	0.338413104507949	0.409603255472255	0.826197301869032	0.408692178554769	0.806973167131658
Plekha5	1259.25216884216	-0.0889765070813842	0.107713122150693	-0.826050766190806	0.408775294546542	0.806973167131658
Rabgef1	322.296036061053	-0.124046501374163	0.149947121188191	-0.827268308929243	0.408085001929711	0.806973167131658
Rad23a	1045.95329963337	0.0753447422367848	0.0910413714789781	0.827587952738412	0.407903893253949	0.806973167131658
Strn3	912.496990174834	-0.122203329180895	0.147696172156325	-0.827396725295982	0.408012236065273	0.806973167131658
Ccdc27	29.9314178405295	0.351464594930611	0.425863688907036	0.825298338613071	0.409202234831846	0.807353524226225
Ccnb2	115.380434120986	0.205522712740304	0.249036386143424	0.825271824423041	0.409217284274792	0.807353524226225
Larp7	520.152667901218	-0.127398280098477	0.154327281124105	-0.825507189464625	0.409083702691112	0.807353524226225
LOC687575	1357.51161397191	-0.112858490475831	0.136798748545107	-0.824996512585911	0.40937357061779	0.807468026748606
Zfp455	95.7969358488976	-0.227180539719656	0.275397288776588	-0.824919303776998	0.409417406136918	0.807468026748606
Cnot4	397.595983167431	0.1308267145146	0.158619552471967	0.82478302627743	0.409494784884957	0.807480521339768
Ssx2ip	283.807468641608	0.118368736224654	0.143548462910424	0.824590760672356	0.409603968628826	0.807555716814438
Timm13	2515.76286409779	0.129001058298388	0.156477687996406	0.824405446873369	0.409709220966628	0.807623136577643
Mnat1	305.208767147162	-0.126411559078544	0.153391771082716	-0.824109130406848	0.409877552699705	0.807814854167652
Creb3l1	88.7460665966364	0.214938389392712	0.260968688113015	0.823617541808811	0.410156905480305	0.807861288825098
Jrk	247.596206641544	-0.152701515896896	0.185384709883571	-0.823700703217645	0.41010963978019	0.807861288825098
Kdm8	274.960657782274	-0.137954891135274	0.16753436846952	-0.823442332433256	0.410256498249029	0.807861288825098
Scnn1a	799.316847332185	0.234966822717813	0.285214752067679	0.823824227233724	0.41003943952495	0.807861288825098
Wnt7a	27.0191496925708	0.35560431672134	0.431848400596223	0.823447108361133	0.410253783318627	0.807861288825098

Pdcl	385.730305164441	-0.181547093028062	0.220586480876104	-0.823020034169867	0.410496600698258	0.807914175504138
RGD1306739	355.152774740117	0.117398665417765	0.14262660722262	0.823118965695668	0.410440344574689	0.807914175504138
Rpp30	258.834089095086	-0.140908203678128	0.171180669936023	-0.823154879174097	0.410419923976235	0.807914175504138
Shoc2	312.47119271598	0.165823966647139	0.201523877252445	0.822850219576783	0.410593174234178	0.807964338356245
Ogfrl1	607.797630187634	-0.103043845547465	0.125283600755172	-0.82248470610956	0.410801087477418	0.808093658751875
Plec	138.20999805881	-0.183581093407185	0.223189944928171	-0.822532992990642	0.410773617102341	0.808093658751875
Slc24a2	52.3136194618308	-0.274146035924517	0.333446316894787	-0.822159436269974	0.410986161703197	0.808317825561263
Ogdhl	64.7394630710869	0.304933723523087	0.371022374278543	0.821874217467434	0.411148488152759	0.808497182758522
Cbr3	74.7808847918443	0.280882992314186	0.341886288882118	0.82156846135188	0.411322545259051	0.808559676481213
Tmem181	176.685907458981	0.207327020121648	0.252346361745897	0.821597025165032	0.411306282948609	0.808559676481213
Pipox	61.0005131041303	0.601127749303015	0.73206224725113	0.821142944551819	0.411564851038467	0.808756240820058
Scn8a	457.855439128694	-0.149630984618451	0.182201351037293	-0.82123970962117	0.411509741813482	0.808756240820058
Mknk1	214.1429564888	-0.149243342899367	0.181870882621622	-0.820600531256365	0.411873844985219	0.809141543292158
Prkra	813.044996757158	-0.097779410061345	0.119176353746773	-0.820459822668412	0.411954024235845	0.809141543292158
Rps27l	1148.39863821598	-0.139110585840317	0.169559401864323	-0.820423900478432	0.411974495075815	0.809141543292158
Fbxl3	1489.25225978521	-0.100234788415624	0.122197698300547	-0.820267401183733	0.412063685765362	0.809176892261421
Capns1	3508.472039611	0.0681278850412725	0.0831252309591499	0.819581302273344	0.412454836294624	0.809245929453598
Cttnbp2	136.646778119023	0.27953810063365	0.341045009158506	0.819651638718836	0.41241472679677	0.809245929453598
Kalrn	283.786956559738	0.153194620788505	0.186814118807301	0.820037702538562	0.412194614315275	0.809245929453598
LOC500227	46.2430582877677	0.257073172951174	0.313589061046591	0.819777233597381	0.412343111818486	0.809245929453598
Map3k11	972.070907802191	0.0858640242429971	0.10474966910872	0.819706878060672	0.412383228073376	0.809245929453598
Cdh6	89.2683902483626	0.269825957689217	0.329602617902697	0.818640214104347	0.412991716103814	0.809740598842702
Cpne3	118.772393485042	0.319952068416476	0.390797945977143	0.81871481595553	0.412949141516551	0.809740598842702
Kif1c	6067.73413852068	0.112930346591227	0.137948754310097	0.818639843150515	0.412991927810236	0.809740598842702
Krtcap2	550.326156648025	-0.110030700133107	0.134373758754988	-0.818840681041997	0.412877317368927	0.809740598842702
Mob3a	221.08199831869	-0.188889863341247	0.230809091323037	-0.818381382893103	0.413139448844924	0.80975047000714
Stk32b	56.6288402489375	-0.387201665527348	0.47310361963138	-0.818428879975674	0.413112336654514	0.80975047000714
Acadm	18449.0825602554	0.102176075096987	0.124883485331574	0.81817123237474	0.413259419193495	0.809845958371252
Nubp1	1181.9216812223	-0.111859365623759	0.136780832981924	-0.817800003005843	0.413471396352147	0.810121684354164
Eif2ak1	899.734997663878	0.0982234433888385	0.120175126981982	0.817335881854864	0.413736506553453	0.810222111214084
Pno1	452.933868506302	0.126777492685023	0.155097940880948	0.817402809895045	0.41369827044416	0.810222111214084
Stam2	180.608147203157	0.173683458549551	0.212444330763203	0.81754809801512	0.413615274279814	0.810222111214084
Ap3s2	1441.31146691793	0.0971820624543274	0.119284318860204	0.814709455382987	0.415238639736346	0.810292427849045
Cald1	634.877076847357	-0.38383087017729	0.47113928543841	-0.814686616124833	0.415251716319782	0.810292427849045
Dnajc12	487.199133115441	0.169088511643153	0.207532246187839	0.814757777401541	0.415210973838171	0.810292427849045
Dyrk1b	362.686734630908	0.118734080073878	0.145769870313505	0.814531012605819	0.415340813359892	0.810292427849045
Edem3	132.890862242392	0.273897213809856	0.335541898867572	0.816283196626823	0.414338182652462	0.810292427849045
Gnpda2	112.908739570314	-0.186662086009771	0.228867634068972	-0.815589704368237	0.414734839908114	0.810292427849045
Hint2	2480.6816402865	0.155283455938347	0.190411118507668	0.815516746896762	0.414776582492007	0.810292427849045
Ift88	200.111481601878	-0.152344530243967	0.186976310349654	-0.81477985076867	0.415198336493266	0.810292427849045
Itpripl2	30.528137788445	-0.399692333747171	0.489314045715058	-0.816842143092543	0.414018645021798	0.810292427849045
LOC498265	70.3380256958934	-0.244223308203401	0.299827602045374	-0.814545780766515	0.415332356771689	0.810292427849045
LOC499219	156.920150886018	-0.201020118318605	0.246581731140249	-0.815227135396619	0.414942308076522	0.810292427849045
Maff	938.51345705049	0.470499135034126	0.577191540483483	0.815152513565972	0.414985015575407	0.810292427849045
Pim2	255.804049572054	0.203956534784472	0.25004975802459	0.815663795861038	0.414692451036399	0.810292427849045
Polr3e	488.223762523781	0.116999627847665	0.143286402621439	0.816543829052479	0.414189166572652	0.810292427849045
Rbp4	2817.33634561187	0.493407653980051	0.60564574388924	0.814680296127508	0.415255334867873	0.810292427849045
Rdh11	470.775232734857	0.10557154677632	0.129238628120961	0.816873007020084	0.414001005029625	0.810292427849045
RGD1311805	354.870501136519	0.142606073663615	0.174765421849088	0.815985634656934	0.41450835189044	0.810292427849045
Slc24a3	27.3057226680974	0.654244084515194	0.802160101372977	0.81560287453264	0.41472730486923	0.810292427849045
Slc39a14	344.212236975961	0.229427010957759	0.281108429794001	0.816151301922482	0.414413605274571	0.810292427849045
Tekt4	12.1034231730087	-0.676409934847859	0.828961509285928	-0.815972668538642	0.414515767871908	0.810292427849045
Ticam2	48.0690468524793	-0.302964294345195	0.370919293422074	-0.816793032117765	0.414046714861798	0.810292427849045
Ubr4	3325.53521233452	-0.101115785898615	0.124106049248057	-0.814753080218597	0.415213663077559	0.810292427849045
Arl6ip4	963.362985101246	0.12747332863107	0.156567575011015	0.814174509773832	0.41554498594047	0.810315740818011
Ulk2	668.90864358275	-0.0882946168280287	0.108435160494268	-0.814261872491961	0.415494946993506	0.810315740818011
Wdr48	925.890962340977	-0.0859246147260661	0.105540770322272	-0.814136702467614	0.41556664202254	0.810315740818011
Csnk1d	2441.00263238273	0.0981985357798012	0.120659076107416	0.813851215737639	0.415730190772624	0.810356602353223
Rars	1160.77036879074	0.0924201144466235	0.113556913134134	0.813866033303109	0.415721701196691	0.810356602353223
Cdc16	779.421762904389	0.0921547171054143	0.113381534773291	0.812784174157457	0.41634181067476	0.810378313533481
Chd5	124.76907221352	-0.210240443221344	0.25851636260037	-0.813257780306719	0.416070277816473	0.810378313533481
Coq10a	354.751404054199	0.15713017796261	0.193150583006697	0.813511279731224	0.415924981822068	0.810378313533481
Ly6g6e	33.3149671260479	0.484985946584334	0.596460507594866	0.813106551748017	0.416156970401503	0.810378313533481
Msx1	12.1113410492569	0.512372792892826	0.630328126930102	0.812866776845647	0.416294444505503	0.810378313533481
Mtmr9	95.8023563023433	0.237572339317078	0.292320336581679	0.812712321336208	0.41638301522396	0.810378313533481
Pfkm	1012.00686335929	0.138874510622737	0.170865786663917	0.812769562205541	0.416350189815287	0.810378313533481
Serpina3m	20.2885414634603	-0.518645134986454	0.638042492124859	-0.812869270288287	0.416293014760974	0.810378313533481
Slc35f2	311.955951705962	-0.204094563839481	0.250852183111205	-0.813604893958621	0.415871333368676	0.810378313533481
Cdc23	498.402362111282	-0.0949305094314135	0.116830572434384	-0.812548526069493	0.416476953865399	0.810422369052238
Cml5	835.065990203505	0.205149200841089	0.252555636039621	0.812293101267022	0.416623468146896	0.810429974149858
Mid1ip1	864.903438927934	0.127713038561593	0.1572143246488	0.812349885081338	0.416590893741513	0.810429974149858
Coro1c	784.663009740641	-0.0937885363839862	0.115503488520001	-0.811997434759259	0.416793103446157	0.810621220699696
Eif5	7607.77111604264	-0.127729886038522	0.157447843952991	-0.811252049133541	0.417220940745446	0.811032808364985
Ifngr2	278.074187398632	0.153032789027868	0.188800352516773	0.810553513210592	0.417622122102166	0.811032808364985
Numa1	1523.1622387985	-0.131043099364594	0.161704519338986	-0.810386128354795	0.417718287890958	0.811032808364985
Paqr3	102.355903425149	0.201445612262458	0.248577024938643	0.8103951373309	0.417713111730361	0.811032808364985
Rap1a	1999.93348568481	0.129379423433472	0.159563570570663	0.810833092859226	0.417461527361857	0.811032808364985
Scrn1	19.4175704944917	0.683221465218997	0.842185583813273	0.811248112471228	0.417223201002474	0.811032808364985
Sf3a1	1248.4250299196	0.0762481412509102	0.093976213290433	0.811355752495216	0.417161401471002	0.811032808364985
Smad6	107.342594903053	0.367207932991203	0.452883057564779	0.810822853400028	0.417467408418528	0.811032808364985
Smim7	1482.28245587553	0.0880845583421486	0.10865291858058	0.810696661377046	0.417539891102662	0.811032808364985
Tmem63a	590.503159010017	0.0975560042493935	0.120358889501624	0.810542575237679	0.417628405777602	0.811032808364985
Wars2	255.812446097371	0.149320900297941	0.18429796063964	0.810214609970153	0.41781684200345	0.811085606526254
Arnt	256.576538458118	0.144510413641203	0.178481925103134	0.809664135781252	0.41813323585955	0.811195361103146
Casp8ap2	158.141751711747	-0.171340137811077	0.211662885304708	-0.8094954274313	0.41823023192543	0.811195361103146
Ikbkap	392.215818370822	0.0974219765137577	0.120300898196498	0.809819194821224	0.418044098921931	0.811195361103146
Il11ra1	1127.63925232548	0.0855718589851022	0.105709121259999	0.809503077550257	0.418225833329755	0.811195361103146
Lipe	212.047299917072	0.632900275084515	0.781454435361463	0.809900419583345	0.417997410680359	0.811195361103146
Dstn	11135.9049131441	-0.108854952097192	0.134536237421939	-0.809112505174322	0.418450435878101	0.811207172810932
Eci1	2732.19274683427	0.198670415082237	0.245507158861596	0.809224529351656	0.418386007994178	0.811207172810932
Pvrl2	481.133211593028	-0.166649273344108	0.205929257501366	-0.809254961466571	0.418368506738576	0.811207172810932
Repin1	311.360209690321	0.128352028543923	0.158697612917639	0.808783611701428	0.41863962441601	0.811340554503861
Rplp2	2625.35759095475	-0.123347186641361	0.152516838967304	-0.808744709610749	0.418662005293431	0.811340554503861
Commd6	387.747643425903	-0.11399218792945	0.141145374112365	-0.807622556859008	0.419307897389468	0.811523554975194
Cyp8b1	266.55825590329	-0.536772926188779	0.664431048984825	-0.807868516994964	0.419166276783349	0.811523554975194
Dpp3	1816.56365599326	0.0927671471701592	0.114861615134398	0.807642719124342	0.41929628716403	0.811523554975194
Hsd17b11	1980.20241282485	0.0905151163896515	0.112040362324899	0.807879540117622	0.419159930473291	0.811523554975194
Pik3cd	178.593907865693	-0.227217499324071	0.281396337366464	-0.807464309772319	0.419399028851336	0.811523554975194
Stx8	456.305884663783	-0.132485065312719	0.164024324124917	-0.807715965418773	0.419254110657617	0.811523554975194
Tada1	129.09533275684	-0.176315335592247	0.218355225478327	-0.807470190859927	0.419395641837431	0.811523554975194
Trim2	600.762654081744	-0.145855252728884	0.180417686228372	-0.808431012380131	0.418842505111257	0.811523554975194
Zfp385a	121.870042798424	0.209879904076707	0.25984861046689	0.807700698108794	0.419262901637765	0.811523554975194
Aqp5	11.2595668955173	0.607163760444204	0.752387981924841	0.806982268497819	0.419676698937031	0.811784438413596
Tacc2	660.241684821549	-0.0917621709280211	0.113696307828734	-0.807081361571096	0.419619609562471	0.811784438413596
Casc3	258.548074436443	0.176855070588044	0.21921673913604	0.806758969616333	0.419805362337883	0.811895141795538

Dhx35	369.327210816331	-0.107891995377019	0.133848890931389	-0.806073136850456	0.42020067975567	0.812219420644512
Rab5b	2606.46746087991	0.0777917235936914	0.0964846372499858	0.806260206919137	0.420092829953242	0.812219420644512
Rbm8a	1183.31687970406	-0.118818865651748	0.147423032199003	-0.805972200404597	0.420258878480589	0.812219420644512
Taldo1	7024.11060339797	0.122269600931515	0.151680446354083	0.806099954677667	0.420185217719371	0.812219420644512
Nphp1	1201.61167843861	0.0915632542980607	0.113727868039908	0.80510833339398	0.420757167320993	0.812629730462261
Raver2	78.4850153119939	-0.271647503562689	0.337373716771579	-0.805182769310421	0.420714218141647	0.812629730462261
Tmem56	94.0889055592704	0.242174611841228	0.300741233708847	0.805259088867349	0.420670184783049	0.812629730462261
Vamp7	256.432271980148	0.140401420310648	0.174364142454983	0.805219572865426	0.420692983611689	0.812629730462261
Mpp2	19.9631557157131	0.368062687052828	0.457333776835082	0.804801013386673	0.420934516793452	0.812834134875021
Fam175b	623.469393116836	0.0932207413583387	0.115892504276307	0.804372482417712	0.421181888412271	0.813173661235582
Crot	2107.20224844485	0.178664998186449	0.222255237570548	0.803873061167962	0.42147028940717	0.813454119443352
Sirt6	415.902433567301	0.123795507741787	0.153989813754093	0.803920108244802	0.421443116171038	0.813454119443352
Cbx6	807.291798916313	0.126245157998806	0.157132562390811	0.803430912587146	0.421725713779661	0.813808907100106
Cacybp	1860.56946423438	-0.154296105679836	0.192112991524608	-0.803152896924578	0.421886366843414	0.813882896261126
Mrps30	851.971192584313	0.134020907165244	0.166875999917668	0.803116728776856	0.421907269467759	0.813882896261126
Atg2a	963.907193980423	-0.0831926384235512	0.103615283491531	-0.802899298445206	0.422032941575644	0.813948852998172
Hbp1	1215.03086648434	-0.113339612126442	0.141185755555735	-0.802769455603471	0.422107999634943	0.813948852998172
Maml1	266.905548790885	-0.173949666601318	0.216709505287649	-0.802685910663802	0.422156298471249	0.813948852998172
Tmbim1	841.491234445339	-0.16701314333379	0.208136717159988	-0.802420378358388	0.422309828992435	0.814106770069203
Herpud2	516.964034430749	-0.106371262710379	0.132644930509282	-0.801924825185346	0.422596444855238	0.814521145027071
Pou2af1	47.776781405376	0.333481757545874	0.415919893470885	0.801793236584433	0.422672571633866	0.814529747234745
Dnajb3	205.609674676352	0.252891154523259	0.315629172990033	0.801228708130998	0.422999254381812	0.814882970390454
Gpa33	31.178512587469	0.431938855779458	0.539086948571349	0.801241537982236	0.422991828329725	0.814882970390454
Krt15	187.103593858648	-0.790759134308397	0.987371006261544	-0.800873358943794	0.423204964543973	0.815141099306354
Acsbg1	14.8721853324134	-0.927673438612914	1.15900984227121	-0.800401691839848	0.423478101098265	0.815488506116007
Cc2d1a	1082.16505382156	-0.0836243692588946	0.104496294461367	-0.800261575684972	0.42355926049171	0.815488506116007
Fndc3a	792.863729595433	-0.124753319559617	0.155927775131073	-0.800071183307456	0.423669555935379	0.815488506116007
Vhl	1460.55018086162	0.083669344876441	0.104578000292192	0.800066406344237	0.423672323474839	0.815488506116007
Zfp879	45.2091225350679	-0.306424412410503	0.383060261171836	-0.7999378778501	0.423746790585289	0.815493738874432
Tmem19	450.073242466392	-0.105986040389117	0.132671434368513	-0.798861042647106	0.424370990399366	0.816556742319146
Elk4	1118.70612356864	-0.0784114903074215	0.0981781906188383	-0.798665058025381	0.424484652911414	0.816637197865798
Ubxn2a	1052.19795524016	0.126893669916371	0.15895357953869	0.798306463337521	0.424692668212086	0.816899114384594
Actr1b	2323.73691520396	0.0743458288672949	0.0933418742696709	0.796489565363824	0.425747538107557	0.816899785826486
Cited4	15.1243261396878	0.423348264903858	0.530981437735298	0.797293906750283	0.425280358301566	0.816899785826486
Clec9a	26.4187277864138	-0.382028981385856	0.479438116204185	-0.796826469306323	0.425551820175613	0.816899785826486
Def6	26.3844689510666	-0.427767787334631	0.536350928419157	-0.797552059051031	0.425130481045945	0.816899785826486
Elfn1	694.518746784023	0.135044329884397	0.169634299193385	0.796090946975559	0.425979175711341	0.816899785826486
Enoph1	265.433389785357	-0.130940483955719	0.164378012017739	-0.796581503501745	0.425694123185008	0.816899785826486
Fubp3	305.018589439124	0.139255384527141	0.174783560002578	0.796730450650435	0.425607595033638	0.816899785826486
Maf	164.039793270704	0.199863483609457	0.250800947602687	0.796900831196522	0.425508628128192	0.816899785826486
Nlrx1	464.877678891714	0.154202011944293	0.193210072591672	0.798105450072376	0.424809298986863	0.816899785826486
Prickle3	357.440204502917	-0.142916561551588	0.179553724222888	-0.795954314900089	0.426058589686733	0.816899785826486
Ptcd3	1104.29899627127	-0.0838999562932385	0.105292387124201	-0.796828323345649	0.425550743252101	0.816899785826486
Rgn	25263.3637813277	-0.148288207442046	0.185897133088659	-0.79768958766741	0.425050647709057	0.816899785826486
Rtp4	183.778324912895	-0.66142279277392	0.830892465405622	-0.796038982554776	0.42600937771299	0.816899785826486
Smoc1	30.0950018687013	0.363170102272079	0.455549276513556	0.797213651729446	0.425326958797247	0.816899785826486
St14	1497.98584715111	-0.208863019337051	0.261715428407017	-0.79805390384639	0.424839209859838	0.816899785826486
Tbkbp1	108.874674411997	-0.319002279129114	0.399805884630465	-0.797892906013535	0.424932640446678	0.816899785826486
Ttbk2	50.9822208619511	-0.267356259395422	0.335574896825509	-0.796711142354731	0.425618811259486	0.816899785826486
Uckl1	518.385674935673	-0.106422192036404	0.133641821856769	-0.796324014128321	0.425843731193802	0.816899785826486
Zfp105	218.068093313639	-0.143072399563794	0.1796552793113	-0.796371807787977	0.425815959518225	0.816899785826486
Ercc8	73.1074577771235	-0.232143465860578	0.291717605693654	-0.795781472662856	0.426159062286346	0.816954613247868
Ino80e	701.596297448254	-0.1213206600009	0.152516857764565	-0.795457379460166	0.426347493805494	0.817178012578962
Apool	569.994627206559	0.133298830450355	0.167927086901695	0.793789929366122	0.427317736341451	0.817276864423888
C1qtnf6	48.7388210831734	-0.423905400207934	0.534431864535454	-0.793188857809604	0.427667798215825	0.817276864423888
Cdon	61.4034110020819	-0.284600835157394	0.358108313217154	-0.794733952419628	0.426768277864848	0.817276864423888
Hacl1	1580.4931273717	0.151892585851291	0.191566496819044	0.792897444874039	0.427837576132409	0.817276864423888
Hist1h2an	222.422385758095	0.325806143225917	0.409854655139731	0.794930932563986	0.426653679644543	0.817276864423888
Homez	74.1302306942494	0.251391407688497	0.316892329690021	0.793302280097484	0.427601728710759	0.817276864423888
Lrtm2	27.1650138330094	0.330175482559711	0.416480835970603	0.792774730655352	0.427909081487467	0.817276864423888
Mlh3	613.56058894372	0.121704633083915	0.153224463819308	0.794289828466535	0.42702672412194	0.817276864423888
Nsfl1c	2343.40829487569	0.109875043289743	0.138411817795446	0.793827037602551	0.427296130112634	0.817276864423888
Nup54	347.332447369143	-0.106313538116696	0.134009228868546	-0.793329974467526	0.427585597393509	0.817276864423888
Pald1	99.3185574783859	-0.366166132555923	0.460568008657261	-0.795031625456235	0.426595105917971	0.817276864423888
Ppp1r36	1501.4917648881	-0.117544344626722	0.14823432907063	-0.792963042796349	0.427799355194248	0.817276864423888
Prpf38a	519.574349374531	-0.0857566624768747	0.108068217654863	-0.793541934324808	0.427462147513465	0.817276864423888
Rab8a	2238.51990508835	0.0772679809957062	0.0972153611978965	0.794812466297539	0.426722598264428	0.817276864423888
RGD1562136	556.501051552549	0.0971365088512306	0.122294229438746	0.794285301089238	0.427029359179663	0.817276864423888
RGD1563216	1549.47508509211	0.11837723390918	0.149072085235154	0.794093902439514	0.427140767109596	0.817276864423888
Sh3gl2	114.960401972084	0.259530286396346	0.326903427013201	0.793905064769682	0.427250700958693	0.817276864423888
Spink8	12.5624892253578	-0.640053781362134	0.807183072431511	-0.792947477743896	0.427808424065418	0.817276864423888
Tmem222	626.627482990938	-0.108409572226821	0.136692367174949	-0.793091629528005	0.427724439298047	0.817276864423888
Trappc2	326.519527467609	-0.107112300336142	0.134784037103584	-0.794695741705853	0.426790509999405	0.817276864423888
Wfdc3	174.648511491278	-0.187583306533918	0.236135011093155	-0.794390063826312	0.426968386821495	0.817276864423888
Adk	4498.74097435173	0.130658219777426	0.165473101893641	0.789603979632939	0.429759083391409	0.817317997305695
Atf3	572.97617783553	-0.723516247177678	0.9163526658066	-0.789560912709102	0.429784243096283	0.817317997305695
Bora	66.2749544317749	-0.227494512304521	0.287597355933788	-0.79101739849408	0.428933838734528	0.817317997305695
Cyp4a8	29860.2037901753	0.20546816326036	0.260480359319064	0.788804821206043	0.430226091234466	0.817317997305695
Epb4.1l5	699.20015095968	-0.096440187628113	0.122300784933377	-0.788549212342739	0.430375524711824	0.817317997305695
Eps15l1	549.571266073654	0.124063859096306	0.157036237820658	0.790033312170864	0.429508314023671	0.817317997305695
Fam65b	25.2527095674429	-0.439308695354762	0.55580792423219	-0.790396603217985	0.429296185337381	0.817317997305695
Fkbp11	63.7169991706862	0.204729033423218	0.259589628489846	0.788664148926991	0.43030832701767	0.817317997305695
Fmo1	5407.97792573564	0.14842160511282	0.187839526203735	0.790151083280725	0.429439539815016	0.817317997305695
Hint3	227.279583022955	0.159238723802806	0.201670390155224	0.789598927637523	0.429762034723772	0.817317997305695
Ift80	353.671831057914	0.166745400594447	0.210996386972937	0.790276094233948	0.429366544792914	0.817317997305695
Irak4	109.496295899006	-0.192243478983115	0.243453239478672	-0.789652581312055	0.429730691309035	0.817317997305695
Lcn12	33.2245046413218	-0.290882163420889	0.36833965973451	-0.789711766662729	0.429696117938923	0.817317997305695
Mecom	1140.1482055762	-0.139046447397134	0.176046010056202	-0.789830154927933	0.429626965787643	0.817317997305695
Nkd2	1470.85864461013	-0.441980384745366	0.558072648876957	-0.791976431087942	0.428374419026037	0.817317997305695
Nprl3	276.328815297054	-0.130683571632039	0.16519172735736	-0.791102397938673	0.428884239991649	0.817317997305695
Poc5	119.445834391165	0.205707502870064	0.260490858658322	0.789691829991948	0.429707763848056	0.817317997305695
Psmb7	3485.1658174556	0.13125328692508	0.166383195768648	0.788861437110415	0.430192996647402	0.817317997305695
Ptcd2	283.909348411353	0.135988403064808	0.172417288273441	0.788716748921024	0.430277576453487	0.817317997305695
Rabl6	1695.17844031579	0.0763306586201243	0.0967740941782124	0.788750948983919	0.430257583385992	0.817317997305695
RGD1306772	150.289259268331	-0.173562334505845	0.220068998605847	-0.788672350968901	0.430303531925263	0.817317997305695
Rnf32	41.7208052925093	0.265687540302112	0.335474512473318	0.79197533768901	0.428375056582063	0.817317997305695
Rpl32	8718.13709737368	-0.120755220634696	0.153112011649416	-0.788672419190672	0.430303492041459	0.817317997305695
Rpsa	15869.7959909127	0.132615073952922	0.168036699785024	0.789203037923156	0.42999334670386	0.817317997305695
Runx1t1	15.2720640806595	0.425761204307488	0.537665345695961	0.791870273425148	0.428436321659534	0.817317997305695
S100a10	1802.85196077809	-0.113426914825029	0.143164256763302	-0.79228515126203	0.428194427759254	0.817317997305695
Setd7	34.7071950511148	-0.275003233512656	0.347388632188911	-0.791629915405835	0.428576498405757	0.817317997305695
Siat7F	147.45680993627	0.223285819487173	0.282279109713205	0.791010782604601	0.428937699367892	0.817317997305695
Snx20	26.820075141167	-0.418841349152504	0.530606226078511	-0.789363804205589	0.429899404838628	0.817317997305695
Tbcel	84.8893495688024	0.267544361188902	0.33765042378642	0.792370873368535	0.428144457523051	0.817317997305695
Tbp	205.264100294469	0.137396412091566	0.173496413958661	0.79192652433902	0.428403519990549	0.817317997305695

Tmem246	196.904108645457	-0.254563440129798	0.321245506276425	-0.792426462491125	0.428112054608692	0.817317997305695
Wdr91	970.472941749036	0.158748866619011	0.201273366997341	0.788722666030166	0.430274117322949	0.817317997305695
Zfp689	116.8700962679	0.171683143664519	0.217152897981827	0.79060949800857	0.429171902587236	0.817317997305695
Fam69b	64.8786368210179	-0.339958567531676	0.431445998235592	-0.787951606740923	0.430725013085413	0.81783698798778
Me1	5712.89328111343	-0.170035899529562	0.215835837155689	-0.787801978440259	0.430812543641957	0.81783698798778
Pkmyt1	79.0797983834272	0.223527852308228	0.283768184990193	0.787712873153675	0.430864673946052	0.81783698798778
Cetn3	226.197150666611	-0.145561693020065	0.184857908746173	-0.787424752380677	0.43103326164308	0.818020379334655
Fam122a	282.482837606756	0.169717416575904	0.215636928419047	0.787051725417328	0.431251587199654	0.818161498933612
Ppfia1	881.4257511717	0.0870028100021638	0.110531328016835	0.78713258551379	0.43120425588718	0.818161498933612
RGD1562079	585.820440827685	0.141162107803268	0.179437786349022	0.786691090407765	0.43146272093131	0.818425448281921
Commd5	248.185898601462	0.133942058095279	0.170336473204668	0.786338096447144	0.431669439230013	0.818680935472773
Atp6v1e1	3072.61612204047	0.0808456053074742	0.102893507212294	0.785721154792307	0.432030866854219	0.819044647764345
Fam173b	398.296376513116	-0.122645285785441	0.156093392438742	-0.785717344400545	0.432033099669588	0.819044647764345
Fbxw4	636.545512159846	-0.096330869427177	0.122614244331284	-0.785641749476565	0.432077398204735	0.819044647764345
Pidd	67.0920317739259	-0.23278896225129	0.296448838669147	-0.785258472579464	0.432302038492861	0.819068813570171
Sae1	834.463454535211	0.0699777592979132	0.0891053706429691	0.78533716646893	0.432255910138471	0.819068813570171
Tubd1	50.8345757196135	0.236597264478942	0.301307531427904	0.78523514947569	0.432315710450095	0.819068813570171
Zswim3	191.398095255497	-0.178804217224074	0.227738876240657	-0.785128214276105	0.432378398858088	0.819068813570171
Osgep	232.65721633814	-0.132692550701016	0.169140431522279	-0.784511127864407	0.43274025500502	0.81961768678338
Arl4d	912.274626895642	-0.170560686948176	0.217468902784277	-0.784299202159342	0.432864567520437	0.819654045902978
Papss2	2554.24247886791	0.105019024004387	0.133913118928672	0.784232529602454	0.432903680939255	0.819654045902978
Prr15	2252.73185645889	0.15121075541044	0.192891150878477	0.783917534432176	0.433088500337786	0.819756888011491
Txndc15	1592.9230757666	0.0750706918490816	0.0957663677329949	0.783894112580164	0.433102244633908	0.819756888011491
Ric8a	1214.8108999702	0.0984522981033375	0.125767436286284	0.78281231621221	0.433737334011617	0.820822267461877
Dusp23	34.5997563123242	0.5139077396146	0.656731592503281	0.782523249194887	0.433907127474132	0.8210068937691
Arhgap27	641.954367338324	0.100173253230123	0.12815656537255	0.78164745550819	0.434421789503454	0.821160495509106
Dclk2	20.8064608530856	-0.423124924998062	0.54115272287147	-0.781895585321779	0.434275939700922	0.821160495509106
Fgfr1op2	939.913907086048	-0.111935733713859	0.14316361825671	-0.781872762625657	0.434289353617269	0.821160495509106
Sf3b2	2594.6112806569	0.0781877575545722	0.0999564040074567	0.782218591504547	0.434086120230342	0.821160495509106
Sf3b5	1174.23987511037	0.0949969003593103	0.121506667395092	0.78182458951341	0.434317667889577	0.821160495509106
Srp54	1616.11289696059	0.0880644310130008	0.112664355792332	0.781652993918725	0.434418533737178	0.821160495509106
RGD1307155	526.470574258576	0.110614008060393	0.141542445458004	0.781490016669325	0.434514346150772	0.821198879007263
Cdipt	1723.6685636316	0.0641426868549008	0.0821349556254038	0.780942612880184	0.434836247746629	0.821426907547729
Ptbp3	241.572332307144	-0.291681830965938	0.373629918575107	-0.780670434740103	0.434996353733044	0.821426907547729
Thap4	945.103588338787	0.128156601201391	0.164147780959325	0.7807391635294	0.434955921519782	0.821426907547729
Tmem138	70.9360748199747	-0.261541234916791	0.334954639444983	-0.780825831671303	0.434904938921869	0.821426907547729
Txnl4b	426.403740453247	-0.127105117150773	0.162719538661723	-0.781130024065584	0.434726024829258	0.821426907547729
Hdhd3	298.558208934946	0.148047680925744	0.189715038492772	0.78036871563761	0.43517387664865	0.821625628237303
Mknk2	4162.79641551955	0.152939598704181	0.196092786343589	0.779934854085884	0.435429221686086	0.821971189783101
Megf9	182.0444468532	0.236755948984606	0.303606665189364	0.779811434103192	0.435501875117376	0.821971822083045
RGD1311021	279.439377400757	0.121242707365451	0.155501583423126	0.779687927907107	0.435574586299903	0.821972563155354
Wipf2	168.913107202387	0.184433670115026	0.236587524113789	0.779557885843215	0.435651152878832	0.821980578290306
Arid3a	59.7459700393784	-0.321979981688692	0.413114407742163	-0.77939664086867	0.435746101986041	0.822023268908439
Cux2	23.8186319151392	-0.360391461714718	0.463305246758424	-0.777870452010298	0.436645389207969	0.822082407443727
Hcn3	171.325111477051	-0.262583678550759	0.337091977961267	-0.778967450186342	0.435998889081729	0.822082407443727
Klhl11	52.2939003082419	-0.281615717635096	0.361525201696031	-0.778965660800259	0.435999943181168	0.822082407443727
Med26	155.445120669281	-0.16902849399825	0.217139026984491	-0.778434426761627	0.436312949831849	0.822082407443727
Mttp	18.535467591657	-0.366136920850619	0.470606167318054	-0.77801131025801	0.436562345531736	0.822082407443727
N6amt1	746.121074950381	-0.108085710568848	0.138795610567918	-0.778740121006615	0.436132816920043	0.822082407443727
Polr3h	513.56494196457	0.121287401116923	0.155896101719297	0.77800150086697	0.436568128411918	0.822082407443727
Rbm33	284.560319196921	0.202684315181499	0.26049457234102	0.778075003098952	0.436524798090252	0.822082407443727
Rnf144a	23.8199761266339	-0.349634392922508	0.449141582602923	-0.778450284866216	0.436303604255656	0.822082407443727
Stau1	803.028532655818	-0.0958798811695795	0.12319702581804	-0.77826457686724	0.436413053841923	0.822082407443727
Trpv6	15.4780374769865	0.612364308618778	0.787213389179661	0.777888584005044	0.436634698890542	0.822082407443727
Zfp472	74.7013136181389	-0.204058781911491	0.262247195869748	-0.778116163395863	0.436500534754273	0.822082407443727
Ecm1	326.461571983709	-0.223762944553594	0.287848146131643	-0.777364549887564	0.436943721330635	0.822507839788671
Ercc1	311.739054011292	-0.126497242268623	0.162836357516749	-0.77683659962494	0.437255180443516	0.822617715801932
Fam185a	113.072712032776	0.240758514754883	0.309946314068392	0.776774892382677	0.437291592369811	0.822617715801932
Trmt2a	611.602363712382	-0.0930926305630545	0.119840319354101	-0.776805594851489	0.437273475379762	0.822617715801932
Wrb	359.410927087627	0.123649540417542	0.159148962269626	0.776942172001462	0.437192888827686	0.822617715801932
Azgp1	1045.88350214811	0.173315699023966	0.223295636406067	0.776171455087412	0.437647757858927	0.823015290507043
Fbxw11	1402.81260552057	-0.0887915505802422	0.114390346133239	-0.776215420109139	0.437621802843126	0.823015290507043
Chdh	6048.3963995878	0.133398619637434	0.171996681544949	0.775588333677079	0.43799209087261	0.823144271404618
Mapk1ip1l	100.596705155327	0.248035796692174	0.319914291675053	0.775319525093649	0.438150874713878	0.823144271404618
Mterfd3	167.395294860725	-0.151545387060004	0.195415056587179	-0.775505171948691	0.438041210539929	0.823144271404618
Mum1l1	34.7170966635316	0.312582646157227	0.402919991388096	0.775793340708539	0.437871016526501	0.823144271404618
Tmem184c	498.074559529262	-0.110080251699346	0.141876641241057	-0.775887071588568	0.437815666766	0.823144271404618
Wipf3	107.698142132031	-0.233482682306027	0.301110635474353	-0.775404966809663	0.438100401145518	0.823144271404618
Brd2	1449.37129821768	-0.082746510967168	0.106771676242034	-0.774985594303076	0.43834817199998	0.823378833738518
RGD1309350	82.8899637452797	-0.460391412410454	0.594276736309724	-0.774708791848967	0.438511754696155	0.823480403908823
Slc47a1	3395.02604511173	0.17093758772427	0.22066459475531	0.774648909644155	0.438547148060893	0.823480403908823
Mrps34	1820.41839560554	0.123602800687829	0.159613989708856	0.774385759752552	0.438702702209756	0.823636424399751
Agtr1b	39.5783311526617	-0.679870351525413	0.878211759065177	-0.774153095204634	0.438840262154101	0.823758615960942
Miip	121.635079855921	-0.188555957634818	0.243690145606902	-0.773752903159978	0.439076928556735	0.823812529105305
Mrps23	1753.56031370103	0.0947852331034532	0.122521357663131	0.773622125246625	0.439154284161088	0.823812529105305
Thtpa	973.072750530917	0.0780906778058964	0.100942648582062	0.773614313700238	0.439158904966483	0.823812529105305
Tmem175	376.244350589845	0.117996383066911	0.152505457340887	0.773719086020382	0.439096930765692	0.823812529105305
Cecr5	462.77961470482	0.117475233002539	0.151901684542939	0.773363596039195	0.43930722814241	0.8239547780638
LOC100366030	271.912044581579	-0.176516120644777	0.228309584362868	-0.773143716841199	0.439437331127972	0.824062812341285
Abhd15	12.0135991268224	0.44081930957278	0.570286436520294	0.772978772321009	0.439534943668975	0.824109892732031
Slc10a7	45.6577902189786	-0.26674233941928	0.345152642781768	-0.772824270645771	0.439626387526522	0.824145393472943
Pik3c2a	903.527620209065	-0.138620203176333	0.179469603276797	-0.77238819635957	0.439884542753298	0.824493356354548
Cant1	596.787602460896	0.088454132331205	0.114574638923576	0.77202191656235	0.440101446965013	0.824763898797088
Dennd2d	275.584536890786	-0.112757174930051	0.146118469237426	-0.771683247973489	0.440302054904999	0.825003817350844
Arpc1b	2067.25592767963	-0.118484659318484	0.153662084292267	-0.771072837285771	0.440663759769524	0.825223620465391
Car11	163.192181296492	-0.18595489641446	0.241277142795122	-0.770710786194784	0.440878377165583	0.825223620465391
Fanca	105.616638303617	-0.176106715333041	0.228523707340845	-0.770627771543968	0.440927595203794	0.825223620465391
Ltv1	550.648806333019	0.120755257266592	0.1566002109712	0.771105329409803	0.44064450195009	0.825223620465391
Magohb	71.2899406176926	0.217164174893934	0.281777956678518	0.770692560389661	0.440889182679659	0.825223620465391
Pgrmc2	1659.62370885715	0.0947739488459472	0.122967472664319	0.770723727116557	0.440870704986931	0.825223620465391
rnf141	138.733407468945	0.215923473478565	0.280162666872581	0.770707517489359	0.44088031506839	0.825223620465391
Alg12	310.069730922668	0.134391170426957	0.174467348522462	0.770294106978157	0.441125451266512	0.825457997875399
Sds	1046.56894607793	-0.671003718036398	0.871368573032895	-0.77005728551914	0.441265912265731	0.825475153482284
Usp13	27.3417998879854	0.315053100701836	0.409141937739554	0.770033750249255	0.441279872651624	0.825475153482284
Tor3a	153.511144700211	-0.296953312559161	0.38573113680791	-0.769845325468349	0.441391649458551	0.825548377117967
Hprt1	476.428678026652	0.152758844898499	0.198467013302402	0.769693876864775	0.441481503118337	0.825580579868874
Yae1d1	1012.26448275724	0.116552879861009	0.151461163199494	0.769523205810153	0.441582773910553	0.825634118813513
Dennd6b	220.926083228051	-0.13229104140249	0.17208731785268	-0.76874369972889	0.442045477194336	0.825916977553077
Mark4	347.726515903921	0.124355455241821	0.161782917347356	0.768656278924822	0.44209738618977	0.825916977553077
Utp18	609.675134385277	0.153799050460998	0.20007042723489	0.768724556580428	0.442056843773943	0.825916977553077
Wdr62	55.5167452771073	0.253077976756778	0.329062864734111	0.769087016127662	0.441841655472561	0.825916977553077
Zfp365	13.81200922343	-0.638131321454863	0.830083529341736	-0.768755551577933	0.442038440035272	0.825916977553077
Chrna2	96.0524002796126	-0.464552597275894	0.604667547607889	-0.768277707500095	0.442322215758946	0.826201200380637
Ndufa9	4104.75386953001	0.0882666181199259	0.114931859831963	0.767990862141942	0.442492613738112	0.826335752506864
Ptms	11884.5558609865	0.124954062230504	0.16271931379435	0.767911683725666	0.442539655596453	0.826335752506864

Tom1l2	157.114624916964	0.238990305974153	0.311422605510747	0.767414766125272	0.442834951952268	0.826732481377156
Zfyve26	328.500347291908	-0.198569866842481	0.258787229155878	-0.767309374153367	0.442897596261262	0.826732481377156
Arfgef2	882.199613868505	0.114601336536005	0.149497351960646	0.76657770209985	0.44333263707096	0.826865564973503
Ftsj2	161.914300501388	0.180248552584101	0.235096929731413	0.766698879436774	0.443260570046611	0.826865564973503
Plcl1	549.043322234847	-0.109004471026501	0.142160883748584	-0.766768383483599	0.443219237369724	0.826865564973503
Rab7l1	7290.78714810646	-0.111464451190888	0.145335996217808	-0.766943180572013	0.443115298747081	0.826865564973503
Rhbdd1	67.4755131426775	0.280482779234794	0.365789749623385	0.766786875585162	0.443208240855002	0.826865564973503
Actr3	2553.69170369646	0.0903840069709593	0.117950848274254	0.766285349307554	0.443506533740548	0.826928021751751
Atg4a11	294.451528426829	-0.150965404012202	0.197123594123738	-0.765841373191677	0.443770693184487	0.826928021751751
Cd34	58.4977146534448	-0.447544843324952	0.584187822988217	-0.766097521573945	0.443618277593076	0.826928021751751
Dlg4	66.4660029153456	-0.258664408730886	0.33766384951823	-0.76604116520007	0.443651808676573	0.826928021751751
Il18	29.534061561943	0.387822115875137	0.506435570070972	0.76578767131382	0.443802651124906	0.826928021751751
Slc7a12	7204.48541484945	-0.399224089938162	0.521164543886134	-0.766023119994492	0.443662545572808	0.826928021751751
Zbtb10	135.659334885283	0.157135726933673	0.205255253825994	0.765562508167927	0.443936659822769	0.82704213662442
Myo19	599.265034769592	-0.187389911766856	0.244814531263391	-0.765436229621708	0.44401182621028	0.827046610407415
Xpo4	686.241038412231	0.0845145163426502	0.110441432645712	0.765242846982677	0.444126949904715	0.827125497725215
Map3k10	277.408494665983	0.106237979203174	0.13891129631689	0.764790064019121	0.444396565293303	0.827356488308548
Syne3	66.1741075616293	0.310208834007438	0.405598573389496	0.764817369585629	0.444380303199476	0.827356488308548
Stmn1	3188.71375378825	-0.0985231214954675	0.128847143899997	-0.764651186773179	0.444479280234223	0.827374958916178
Cpsf4	1225.72846127759	0.103373325296998	0.135297157568834	0.764046541365107	0.44483950789833	0.827638868353645
Hoxd3	128.472956297766	-0.209386637107676	0.274009704275814	-0.764157742737864	0.444773245318433	0.827638868353645
Tsga10	39.2016619435929	-0.290619477509539	0.380299303472661	-0.764186194546715	0.444756292383231	0.827638868353645
Def8	2485.34918777938	0.0825497473768812	0.108171110253877	0.763140428004645	0.445379651778217	0.828376285245352
Kirrel	23.9538364874199	-0.514213207018748	0.673814950420175	-0.763137129412307	0.445381618787115	0.828376285245352
Crbn	718.984983128105	0.107490897008841	0.140891390771195	0.762934459092709	0.445502484177775	0.828465516224573
Dkk2	235.74859337088	-0.588463192391498	0.771816082325603	-0.762439661296467	0.445797642546039	0.828878783768736
Ppp1r32	48.9912437627212	0.326551926048398	0.42838376026287	0.762288294607654	0.44588795853641	0.828911111665821
Arhgef2	664.85504313159	-0.168992319100483	0.221850690857616	-0.761738980605395	0.446215805354491	0.82938492946184
Irgq	387.900803575131	0.0998920679054476	0.131175979393783	0.761511889349627	0.446351380211678	0.829487916093741
Ttc26	278.198885763942	0.108199305579116	0.142105416381638	0.761401699767279	0.446417172509714	0.829487916093741
Fam70b	16.6155474914975	0.45536300969483	0.598259538500842	0.761146259090007	0.446569712960225	0.829635723685178
Pbdc1	292.310965321391	-0.119168730824183	0.156601271902141	-0.760969112043043	0.446675516545688	0.829696669563374
Adipor1	6852.30743935773	0.163549835282832	0.215063895285885	0.760470905939023	0.446973153667323	0.829830364975813
Atp5d	7196.75908948656	0.111144726951899	0.146323777287452	0.759580766791959	0.447505219268893	0.829830364975813
Atp5g3	9904.08625155652	-0.106560179725068	0.140324724934432	-0.759382780011574	0.44762361144349	0.829830364975813
C1qtnf1	43.3217520440124	-0.425807274924803	0.5598144875717	-0.760622106747937	0.446882811712682	0.829830364975813
Cdk5rap2	352.530426208138	0.098192761259779	0.129256936866127	0.759671114297554	0.447451199165041	0.829830364975813
Fxyd3	27.9811630515591	-0.511418538289436	0.673406063363463	-0.759450450646453	0.4475831437412	0.829830364975813
Hsd17b10	6183.79600813033	0.0990564889966184	0.130347375527263	0.759942335593097	0.447289054230521	0.829830364975813
Lrrc32	289.243679892174	-0.262728484726911	0.345698374276231	-0.759993405456334	0.447258526739338	0.829830364975813
Nat14	86.6683155277686	0.189859476554422	0.249978092872951	0.759504460460524	0.44755084683583	0.829830364975813
Nat8b	3942.87086915264	0.174253267988603	0.229408830473395	0.759575242282625	0.447508522574645	0.829830364975813
Sos1	553.383476405942	0.0851297280256584	0.111924128115712	0.760602110187071	0.446894758991753	0.829830364975813
Zfp523	582.660061120586	-0.0912062819860419	0.120082158853955	-0.759532330668438	0.447534181466853	0.829830364975813
Foxn3	87.7432429292471	0.273923632424893	0.360859282335128	0.759087117427957	0.447800444954834	0.830022807788102
Eef1b2	5326.43045063161	-0.124423168210119	0.163995726328068	-0.758697625822359	0.448033457464612	0.83031930173533
Akirin2	998.254167750254	-0.142760439710797	0.18842128918002	-0.757666186937089	0.448650846164573	0.831192423391449
Pacsin2	3172.87777711061	0.067868247426649	0.0895628146283111	0.757772605833176	0.44858712463679	0.831192423391449
Kif26b	54.9176175470884	0.274005266381202	0.361750999467248	0.757441629144717	0.44878532378385	0.831294263485054
LOC500034	46.7968025614862	0.323773661164768	0.4275198368145	0.757330147712543	0.448852093525231	0.831294263485054
Cand1	1261.70120416448	0.0688315438348808	0.0909994280167763	0.756395345937683	0.44941219754964	0.831442101433752
Ccl27	45.9291140143865	0.27266841657786	0.360415777969691	0.756538512586397	0.449326390868964	0.831442101433752
Cmtm6	94.1432160278375	0.292509890470309	0.386507688221869	0.756802256162103	0.449168340948125	0.831442101433752
Egfl8	178.923772327059	0.24596098739776	0.325402200931416	0.755867620728233	0.449728568982552	0.831442101433752
Lppr2	66.223634467611	-0.224633962261192	0.297157446540508	-0.755942564712307	0.449683632349269	0.831442101433752
Slc7a4	57.1119267027459	0.249376530632079	0.329895154976767	0.755926623565118	0.449693190494856	0.831442101433752
Syt3	60.4669819612637	-0.327757899044053	0.433625793557827	-0.755854250170069	0.449736586276149	0.831442101433752
Taf1c	243.528721296437	-0.151589772360432	0.200430296832236	-0.756321647756255	0.449456372048908	0.831442101433752
Tspyl4	69.6688417046439	0.362348300181401	0.479063728658206	0.756367636506923	0.449428806221881	0.831442101433752
Usp12	355.41588536701	0.158277013172746	0.209352799729806	0.756030076392677	0.449631163310222	0.831442101433752
Xpc	887.705051809657	0.0939872175235019	0.124226164634976	0.756581496335108	0.449300630448218	0.831442101433752
C4b	112.637710068275	0.334171689995249	0.443121700776744	0.754130726185341	0.450770728991041	0.831493329643972
Cox18	201.442280288401	0.129556360763578	0.171557840955161	0.755175980545474	0.450143398608668	0.831493329643972
Cyp4f1	5682.31847949596	0.0805926034151633	0.106816008783269	0.754499295875087	0.45054946801584	0.831493329643972
Fam110b	59.9612957986737	-0.210435030055533	0.278785771729142	-0.754827008388306	0.450352786224814	0.831493329643972
Farsa	693.260758982778	0.105478917084985	0.139842734324465	0.754268125509129	0.450688237783011	0.831493329643972
Fbxo33	431.964202729049	-0.200977986352204	0.266404044617796	-0.75441041685588	0.450602818534114	0.831493329643972
Fbxw2	971.935477951269	0.0714403619897086	0.0946396562928044	0.754867090479282	0.450328733669552	0.831493329643972
Klhl20	131.860917276904	-0.187479763658084	0.248353888979984	-0.754889582877414	0.450315236697241	0.831493329643972
Malsu1	413.366186106692	0.164765224350488	0.218266871587202	0.75487967162557	0.45032118409581	0.831493329643972
Mrap	1025.97790667316	0.153027574822728	0.20287058990986	0.754311282333835	0.450662329240629	0.831493329643972
Phf15	168.358268808635	-0.202926212400086	0.269129503437181	-0.754009537447283	0.450843494852866	0.831493329643972
Serpinb6b	39.264756263616	-0.323838304723252	0.429468079092591	-0.754045109493303	0.450822135490747	0.831493329643972
Slain2	934.920562855773	-0.0732557822239338	0.0969525701745352	-0.75558370543512	0.449898828515448	0.831493329643972
Slc35a5	111.280754083706	-0.236448668642934	0.312994241676092	-0.755440954366271	0.449984447885461	0.831493329643972
Taf6l	312.711096674299	-0.129383161885962	0.171600411789697	-0.753979320542225	0.450861639151487	0.831493329643972
Fam212b	43.2504698985714	0.283284679214162	0.376192724234812	0.753030723255937	0.451431452059174	0.831977760449843
Gab1	890.086991003913	-0.121148073959796	0.160846915253165	-0.753188668673658	0.451336547565543	0.831977760449843
Klf10	331.965906365571	0.193267641496177	0.256804265607952	0.752587349118365	0.451697922118426	0.831977760449843
Nars	856.365308473444	0.0772082215761253	0.102593096350813	0.752567417520134	0.451709903196901	0.831977760449843
Plekhh1	771.953891710168	-0.134261016393547	0.178301552101931	-0.752999706456807	0.451450090416061	0.831977760449843
Rpl37a-ps1	4867.27028611201	-0.093706455238323	0.124428369320937	-0.753095582219088	0.451392478957556	0.831977760449843
RT1-CE12	182.578470698485	-0.621109417259072	0.825163134065596	-0.752711059931692	0.451623562359128	0.831977760449843
Zfp385b	62.3096722838395	-0.208352237985024	0.276831550323066	-0.752631836009567	0.451671181261602	0.831977760449843
Flot1	1065.32040264222	-0.0777012886748354	0.103293697574177	-0.752236491670139	0.451908852220059	0.832071073113558
Kcns3	46.8128178774793	0.239093784931394	0.317837542473564	0.752251553012434	0.451899796428893	0.832071073113558
Npepo	85.7856671392631	0.188527160618229	0.250661727034084	0.752117855601441	0.451980186996578	0.832071073113558
Cldn1	148.916360672434	-0.321493167463086	0.427625388708223	-0.751810289922817	0.452165153050465	0.832103478338255
Fzd9	46.4295474412734	-0.386591718966722	0.514357173571166	-0.751601686203047	0.452290628996458	0.832103478338255
Golt1a	190.588323119119	0.244063342339725	0.324706755924334	0.751642329230127	0.452266180514856	0.832103478338255
Rpp25l	228.019913189898	0.140718473302173	0.18717994483028	0.751781786396856	0.4521822968712	0.832103478338255
Ankrd42	195.971318189333	0.131083814992526	0.174559001109771	0.750942742334407	0.452687115508843	0.83246984723735
Cct6b	17.9896645175685	0.385352971757009	0.513729278273434	0.750109032236047	0.453189040063571	0.83246984723735
Gtpbp3	273.186250397237	0.132152068167972	0.176060250724399	0.750607065616648	0.452889167634993	0.83246984723735
Hoxc5	26.3265471866129	-0.396487549735477	0.528637324145358	-0.750018077850394	0.453243816992655	0.83246984723735
Mki67	224.321135627194	0.232989574836695	0.310731161308132	0.749810781306396	0.453368674502833	0.83246984723735
Mrps26	809.277675397576	0.10714248622183	0.142828087523545	0.750149974557128	0.453164383927811	0.83246984723735
Nudt18	984.400604522204	0.0735516588811794	0.0980644254199198	0.750034057368156	0.453234193118921	0.83246984723735
Rars2	346.39318145447	0.11685379338466	0.155831148075885	0.749874430288837	0.453330335795597	0.83246984723735
Slc19a1	1492.44685928194	0.0721737162874304	0.0962243086271429	0.750057000327168	0.453220375623239	0.83246984723735
Tmem184b	815.850710509866	-0.0915438342362196	0.12204757107908	-0.750066825802737	0.453214458261063	0.83246984723735
Zfp286a	47.526320315861	-0.332916307133543	0.443672691648741	-0.750364657099779	0.453035110983344	0.83246984723735
Zfp422	518.700686344723	-0.124464289066233	0.165736298575972	-0.750977849364603	0.452665986659685	0.83246984723735
Tspyl3	60.3955703917996	0.211919663899873	0.282788248213718	0.749393460437274	0.453620091365912	0.832796956624933
Lnpep	39.4222220075794	-0.364761668019135	0.486893626571039	-0.749160901094513	0.45376023192742	0.832821500489002

LOC100365008	31.3533147173526	0.297985322779661	0.397776197521151	0.749128089203518	0.453780006381171	0.832821500489002
Pp2d1	190.950224198811	-0.168710355971202	0.225316899861124	-0.748769204951725	0.45399632372743	0.833083987001286
Myof	1237.71830705102	-0.162103398197168	0.216552942275642	-0.748562436943631	0.454120979462933	0.833178216719241
Mzf1	142.728112498473	-0.173677320264844	0.232182390731473	-0.748021069632744	0.454447448909104	0.83350810138791
Parpbp	28.4084152315709	-0.360355214358759	0.481717630483191	-0.748063163055299	0.454422059893142	0.83350810138791
Cnn1	42.533981878318	0.958808761121558	1.28284883132783	0.747405881119389	0.454818596449437	0.833584381461247
Ddx52	306.186028933521	0.118529535913213	0.158626764155876	0.747222806592331	0.454929079553415	0.833584381461247
Ndufs7	3234.53138706225	0.113103048339012	0.151329016259307	0.747398292375114	0.454823175857934	0.833584381461247
Phf20	466.244863490161	-0.154823861393598	0.207102194200902	-0.747572289086465	0.454718184513203	0.833584381461247
Slc22a4	272.802353561077	0.142923726324714	0.191126747262926	0.747795525071634	0.45458350170091	0.833584381461247
Umod	58400.7798806183	-0.13121030570169	0.175584390152082	-0.747277736865118	0.454895928258558	0.833584381461247
Vps4a	777.505283927963	0.0784364896128485	0.105027355668879	0.746819617739743	0.455172452096309	0.833895888221574
Man1b1	1500.13689868284	0.0819534928914245	0.109800760443124	0.746383654909893	0.455435690112976	0.834243683130392
N4bp2l2	730.408647282767	0.101697625569265	0.136301361527999	0.746123328697448	0.455592918118595	0.834397213557195
Ccdc77	226.768581123601	-0.287060870498665	0.38494033393097	-0.745728221221273	0.455831607687567	0.834488938087604
Farsb	567.84701168161	0.0979677913875363	0.13138120724631	0.74567583477802	0.455863260301419	0.834488938087604
Rap1gap	2045.37109554156	-0.181769057100168	0.243688427881385	-0.745907627540869	0.455723217275678	0.834488938087604
Atg12	1260.67259457149	0.130580670280896	0.175429027872509	0.74435041831158	0.456664506560004	0.834628281953762
Cul9	547.85474731212	0.102774004024817	0.138161481960543	0.743868714828694	0.456955903928605	0.834628281953762
Cyp26b1	56.9574552018753	-0.426622113589629	0.573234778271843	-0.744236270652987	0.456733548573939	0.834628281953762
Fam81a	1393.16110577291	0.147251187478968	0.197959015887939	0.743846835257679	0.456969142039037	0.834628281953762
Gpsm1	232.51964591781	0.19961386686386	0.268183572447329	0.744318024561566	0.456684099267572	0.834628281953762
Hp1bp3	3373.598802062	-0.0726168158806675	0.0976042097100397	-0.743992662779565	0.456880913991048	0.834628281953762
Laptm4a	7500.83200127362	-0.0758234803681765	0.101776725295888	-0.744998231646192	0.45627278890775	0.834628281953762
Nars2	153.759705279477	0.193250042882942	0.259628394680773	0.744333234893482	0.456674899545684	0.834628281953762
Prmt1	1113.54427657224	-0.0959724671949719	0.129011274611108	-0.743907596326535	0.456932379430355	0.834628281953762
RGD1562114	472.895394953271	0.118904836092211	0.159653366440407	0.744768737066342	0.456411537326443	0.834628281953762
Sdccag8	296.016162414071	0.10986590653213	0.147513118289365	0.744787364040494	0.45640027489846	0.834628281953762
Slc16a11	1329.82627350074	0.103794625301314	0.13955986940929	0.743728306286339	0.457040861066357	0.834628281953762
Slc44a2	652.694864653875	-0.202884576345885	0.272238121289479	-0.745246754513676	0.456122563070316	0.834628281953762
Tmem55b	580.3912568536	-0.0891609286755724	0.119807024383172	-0.744204516676871	0.456752755954313	0.834628281953762
Wibg	485.520683838817	0.0983702044682761	0.132187459116936	0.744171989729037	0.456772431361291	0.834628281953762
Gja4	155.472689864966	0.256793907172038	0.345457798478192	0.743343784112748	0.457273569700841	0.83491909931241
Stxbp3	860.420965220989	0.102795144581094	0.138325101180845	0.743141654721803	0.457395922885444	0.835008361631216
RGD1310039	24.5251893996791	-0.336440500636133	0.452935188541234	-0.742800535590324	0.4576024511746	0.835251238164526
Ankrd13c	3511.53244737254	0.116216245262468	0.156491061266904	0.742638233274262	0.457700734398421	0.835296491196605
Eif4g3	1049.66396816789	-0.107077012768434	0.144289147256608	-0.742100253583211	0.458026596211624	0.835408648163672
Gpatch2l	156.838107605608	0.148260183271749	0.199735826147166	0.742281373009722	0.457916875113578	0.835408648163672
Kpna3	188.878114334808	0.182744013527108	0.246337887182051	0.741842903735125	0.458182522622398	0.835408648163672
RGD1309922	1118.79037427356	-0.0642817238688839	0.0865970798876595	-0.742308215846022	0.457900615134014	0.835408648163672
RGD1559896	904.815078726246	-0.114941654720234	0.154916000789822	-0.741961154007439	0.4581108719408	0.835408648163672
Zfp319	43.3064402389352	0.32260305811311	0.434887080044425	0.741808788801347	0.458203194848877	0.835408648163672
Wdr3	494.15345200102	0.100885077485489	0.136041690354438	0.741574713035741	0.458345049105288	0.835533252306449
Gmps	891.463899016713	0.126212596034762	0.170275652285003	0.741225150754439	0.458556936179195	0.835719592598603
Sult1a1	1589.46460943395	-0.201676861275152	0.27214492117357	-0.741064210955919	0.458654508250783	0.835719592598603
Ywhaz	8328.6196216293	0.0806298148505691	0.108805969164621	0.741042200805896	0.458667853125997	0.835719592598603
Mrps15	1112.58595731172	0.111243282865662	0.150170224724537	0.740781223905871	0.458826101448564	0.835873933172685
Bcl6	2008.75334024824	-0.169670961499372	0.22915926021464	-0.740406306690166	0.459053493204162	0.836020189674492
Zcchc8	600.013246887135	-0.0857930818962701	0.115855765070931	-0.740516294927095	0.45898677748457	0.836020189674492
Clcn2	880.323974938431	0.101017789764968	0.136497587285788	0.740070149031023	0.459257430505819	0.836257602551929
Abhd14a	2706.08494157981	0.113001638620929	0.153076649674548	0.738202977796936	0.460391113623164	0.836674130154103
Acaa1a	2733.17419244301	0.137681858437666	0.186848960182986	0.736861785598541	0.461206405193144	0.836674130154103
Adck4	558.668741429252	0.108855262932182	0.147672065766093	0.737141871534487	0.461036078338002	0.836674130154103
Ankfy1	1332.23378157137	-0.0763994561024064	0.103632175540207	-0.737217526353726	0.46099007689178	0.836674130154103
Atp1a4	97.6517691267659	-0.324530703287292	0.440296463329927	-0.737073154830479	0.461077863329424	0.836674130154103
Dag1	3272.81545381556	-0.153310939829573	0.207737268300568	-0.738004023465603	0.460512004170091	0.836674130154103
Dgkz	1660.09318496603	-0.104002942933144	0.141062525160594	-0.737282583129294	0.460950521572328	0.836674130154103
Fbxw9	248.680324325256	-0.126706084697916	0.171552754435734	-0.738583796655867	0.460159766316455	0.836674130154103
Gnl1	1537.28558390587	0.0754519767152421	0.102059048439819	0.739297278082437	0.459726501778339	0.836674130154103
Invs	199.844577462939	0.132318392462685	0.179119778118656	0.738714584466669	0.460080327781392	0.836674130154103
Lpar2	117.055955444181	0.171151998760922	0.231524218309397	0.739240153840854	0.459761182296839	0.836674130154103
Mapk3	1850.80799684356	0.066654338665194	0.0901590776954683	0.73929703329856	0.459726650385184	0.836674130154103
Mios	241.838592291568	-0.117769438619046	0.159501780572657	-0.738358143691063	0.460296842550594	0.836674130154103
Mrpl36	477.280230816468	0.0859339440047423	0.116644016330545	0.736719693886598	0.461292827952342	0.836674130154103
Nrxn2	49.2047230430311	0.304011282497879	0.411520722477552	0.738750847509173	0.460058303517621	0.836674130154103
Ppp2r2b	48.9630520567792	0.246734027878275	0.334188156391861	0.738308713696485	0.460326872582335	0.836674130154103
Ptpn21	661.567623185891	-0.0753270710305705	0.102038016882093	-0.738225548989379	0.460377399819112	0.836674130154103
RGD1306215	440.314046930738	0.126643206540642	0.171764710097208	0.737306321356523	0.46093608891073	0.836674130154103
Rps6	5608.20158544706	-0.116432470045015	0.157864656960953	-0.737546150521927	0.460790288770904	0.836674130154103
Scnn1g	1137.72153207162	0.12284041581961	0.166439137353848	0.7380500630597	0.46048402756981	0.836674130154103
Sdhd	9442.52437326559	0.117592478481224	0.159167422463731	0.738797403771613	0.460030028558702	0.836674130154103
Slc22a2	30694.2605597211	0.0775963903602115	0.105252788572862	0.737238332706929	0.460977426166855	0.836674130154103
Stap1	21.0566302816265	-0.381526519910471	0.517665998295933	-0.73701290246296	0.461114503100826	0.836674130154103
Stt3a	2670.34496803864	0.0789145122715484	0.107124106476845	0.736664368711496	0.46132648017559	0.836674130154103
Vdac2	5116.06292033293	0.0863057853131105	0.117060803061277	0.737273135465615	0.460956265750256	0.836674130154103
Camk1	803.499530416407	-0.08360705668597	0.113525392791705	-0.73646128526832	0.461450019953461	0.836735393881738
Vti1a	123.601917649048	0.148755462568973	0.202012719222209	0.736366814632821	0.461507494654638	0.836735393881738
Arhgef12	1059.38412475575	-0.114761423822777	0.155908533558702	-0.736081734612477	0.461680957853838	0.836738472743256
Cftr	412.691378313263	-0.472515484753567	0.642179056267168	-0.735800210458724	0.461852293133386	0.836738472743256
Crocc	956.896250150067	-0.176540404320595	0.239895733724435	-0.735904726523333	0.461788680632403	0.836738472743256
Emid1	128.272005091876	-0.298495147776377	0.405763514295049	-0.735638216006102	0.461950898861863	0.836738472743256
Fam3a	1164.0704772952	-0.0819459990982842	0.111330915890215	-0.736057890506281	0.461695467973736	0.836738472743256
Mapk1ip1	487.646474625734	-0.134825815552713	0.183253240605308	-0.735734959487574	0.461892009867272	0.836738472743256
Ncor2	2189.56011326451	-0.120282463242267	0.163546297452051	-0.735464300422522	0.462056774041504	0.836796891930486
Stard3nl	259.708067931119	-0.108298318312797	0.147306815763042	-0.735188780993038	0.462224530661027	0.836967343554761
Dusp11	348.734892927206	0.182618526240868	0.248517520383294	0.734831596417072	0.462442061651181	0.837227854846658
Tmem109	609.763496278013	0.125440509557021	0.170750921490511	0.734640308011409	0.462558582760607	0.837305439028039
Cadm4	78.0091473352545	-0.231010908573648	0.314696109267571	-0.734076150834235	0.462902327908029	0.837401736114937
Tfap2a	107.547334412261	-0.184756435757552	0.251687992245648	-0.734069329685104	0.462906484956022	0.837401736114937
Tuba4a	3043.4255717035	-0.0837017977699768	0.113984844215085	-0.734323921275315	0.462751342004795	0.837401736114937
Uvssa	45.3412623445644	-0.253949833911751	0.345924329117795	-0.734119610954786	0.462875842272074	0.837401736114937
Cyp2j10	18.2466163275328	0.357678842454116	0.487458980032455	0.733761930963508	0.463093846144227	0.837607360801286
Stk35	137.515967687525	0.161960865130457	0.220777762921595	0.733592292027955	0.463197260094097	0.837661107116867
Cadps	20.1914997440777	0.506539120439087	0.690934840745243	0.733121403883372	0.463484386653105	0.837780460989058
Hspb11	149.227680123926	-0.156280183060464	0.21311730251357	-0.733305936295399	0.463371855215646	0.837780460989058
Syt17	389.304916181911	-0.125820374551451	0.171606716950589	-0.73319026660057	0.463442391054202	0.837780460989058
Atoh8	58.5167640591594	-0.333611694932857	0.455410724245764	-0.732551249172654	0.463832174217647	0.838009297751992
Dis3	224.313973928989	0.149126448093677	0.203530861652624	0.73269698208323	0.463743265058363	0.838009297751992
Tada2a	155.706448472808	-0.141636892477147	0.193346181754515	-0.732555932534417	0.463829316831012	0.838009297751992
Akt1	989.949454620958	0.0875628493110841	0.12019506371256	0.728506201556543	0.466303773792459	0.838894352618659
Alg9	472.684631006986	0.0926653284414169	0.126751211398706	0.731080416659141	0.464730035205188	0.838894352618659
Arsk	84.3290915646011	0.184814227358867	0.252853230214939	0.730915033997253	0.464831052502365	0.838894352618659
Astn1	22.6858521501723	-0.535542971196278	0.734600009779797	-0.729026632271366	0.465985371656196	0.838894352618659
Atat1	155.678513321659	-0.149638546461773	0.205132867887448	-0.729471332423805	0.465713397555244	0.838894352618659
Casc5	27.5746133478931	0.307526146317985	0.422630569538903	0.727647663190811	0.466829295759597	0.838894352618659

Ccdc61	177.400529715385	0.152288738070711	0.208784028972181	0.729407985948016	0.465752134229771	0.838894352618659
Ddx28	285.327382455142	0.134439263715878	0.183861234535364	0.731199613967674	0.464657235932759	0.838894352618659
Dusp6	939.769485723881	-0.139220898157594	0.191235250350468	-0.728008554398053	0.466608349762471	0.838894352618659
Fam21c	2441.80719253186	0.0719469933210871	0.0986880196353609	0.729034725662969	0.465980421032049	0.838894352618659
Fance	406.486748344117	0.102907160536664	0.141356307305358	0.727998364546714	0.466614587430424	0.838894352618659
Filip1	53.1442105558341	0.285426098279549	0.391073481595046	0.729852858126304	0.465480130611624	0.838894352618659
Hist2h2ab	813.137422112907	0.447946475422	0.613773982938253	0.729823172493552	0.465498278243301	0.838894352618659
Lnp	37.3571983793558	0.336199929471335	0.461949304759997	0.727785334899477	0.466745003088421	0.838894352618659
LOC690806	31.8080021802683	0.288984169244141	0.397150449029132	0.727644070277617	0.466831495716436	0.838894352618659
Mdm4	278.095682737856	-0.123153548533944	0.168398634429051	-0.731321539224418	0.464582777141196	0.838894352618659
Naa35	905.506792504781	-0.0797243570638163	0.109050736408237	-0.731075824791901	0.464732839796379	0.838894352618659
Nicn1	621.200985502729	0.0761666502219965	0.10459616938892	0.728197319911271	0.466492806239979	0.838894352618659
Paox	494.32029877409	-0.151926492066484	0.207896854681758	-0.730778213547519	0.464914632960101	0.838894352618659
Pex26	357.496043446103	0.102208935072443	0.140271528581848	0.728650611466063	0.466215410976314	0.838894352618659
Phldb3	201.735686004543	-0.197510721989967	0.27133601204757	-0.727919307501799	0.466662983388198	0.838894352618659
Pmm2	1447.23726505996	0.0806689443742751	0.110700662851279	0.728712387952456	0.466177613471272	0.838894352618659
Poc1a	40.7462188144863	0.24286014924919	0.333669763734222	0.727845839345023	0.466707960520886	0.838894352618659
Prelp	222.379032554763	-0.572268779001871	0.786122671792302	-0.727963712962432	0.466635799575487	0.838894352618659
Prlr	3563.75963400305	-0.0729315940210603	0.100022161468888	-0.72915434889643	0.465907252436432	0.838894352618659
Rnf146	566.440646551815	-0.107326832919434	0.147147488232585	-0.729382704445425	0.465767594489886	0.838894352618659
Rps4x	10836.8452519004	-0.0943208599727095	0.12944818766956	-0.728637933606922	0.466223168044926	0.838894352618659
Sema4a	1371.65832753171	0.120383993735794	0.165370374612314	0.727965900893769	0.466634460206269	0.838894352618659
Smpd4	590.648307618435	0.0875933302021577	0.120138935564237	0.729100268707826	0.465940330265798	0.838894352618659
Snrpb2	523.268583592084	-0.112978948300895	0.155013970098823	-0.728830751375957	0.466105198403343	0.838894352618659
Stxbp5	247.84200875574	0.144070942839619	0.196935331397682	0.731564731514272	0.464434281357866	0.838894352618659
Tap1	261.794796154986	-0.14401706668685	0.197438274192488	-0.729428310067396	0.465739705738035	0.838894352618659
Tmem129	486.064466259084	0.114891510566715	0.157703495783109	0.728528622629433	0.466290053977873	0.838894352618659
Vamp5	128.553623712403	-0.178826958540523	0.245194468218283	-0.729327051462364	0.4658016286612	0.838894352618659
Aspg	8546.65828026184	-0.103869618197424	0.142789523056374	-0.727431648864148	0.466961572520814	0.838953518477644
Echdc1	484.362681780635	0.105327734895873	0.144906430107337	0.726867226097925	0.46730729592486	0.838953518477644
Kdsr	294.143113133885	-0.115346937597432	0.158661335309743	-0.727000925412914	0.467225388844995	0.838953518477644
Mex3c	571.497350602834	-0.137279465448456	0.188822997110486	-0.727027255944515	0.46720925913252	0.838953518477644
Rbm3	1458.32949624549	-0.139738659924835	0.192246970845717	-0.72687054214747	0.46730526434522	0.838953518477644
Shq1	63.3015293957516	0.256161481201507	0.352297967813853	0.727115977395754	0.467154911895268	0.838953518477644
LOC288978	43.5055220029477	-0.269984636714803	0.371560724353458	-0.726623184365343	0.467456821671355	0.838956929593656
Morn1	147.183763954662	-0.193918898535605	0.266859490738739	-0.726670421197257	0.46742787732819	0.838956929593656
Bmp6	34.3740005794611	0.40602693212647	0.559064372604018	0.726261504082743	0.467678473991641	0.839089745128492
Ptges3	5017.3598601876	-0.124782392849665	0.17180553534315	-0.726300189341599	0.467654763317498	0.839089745128492
Aldh18a1	57.105433086225	0.21366777796155	0.294755148710291	0.724899221935424	0.468513858243632	0.839660755723293
Egr1	685.727181627727	0.575000398439155	0.793195157239414	0.724916678059849	0.468503148505468	0.839660755723293
Hhip	12.8523756324196	0.499004377092769	0.688101194103733	0.725190395495147	0.46833523421952	0.839660755723293
Kctd15	744.085901268615	-0.0988405819035763	0.136318459120361	-0.72507114987491	0.468408382361708	0.839660755723293
Nip30	1096.47329899606	0.0843585166097476	0.116313459414705	0.725268743911875	0.468287176852489	0.839660755723293
Snx17	1179.06816403597	0.076157175752122	0.104958227467883	0.725595101874467	0.46808702472165	0.839660755723293
Spdl1	88.0789548129769	-0.1998640377062	0.275692350991215	-0.724953147911488	0.468480773840958	0.839660755723293
Arhgef11	855.211054158406	-0.0750056230145228	0.103734814792363	-0.723051592318884	0.469648187954975	0.840448879128388
Asns	151.470116924851	0.16942971029795	0.234319237877815	0.723072129426686	0.469635571110219	0.840448879128388
B3gnt7	258.787460528455	-0.198835130464129	0.274867958045963	-0.723384172813917	0.469443892242855	0.840448879128388
Ccdc112	95.9222621285807	-0.188268996995385	0.260224439187963	-0.7234869929315	0.469380742412901	0.840448879128388
Impact	1207.94399268777	-0.128025008849968	0.176938682293447	-0.723555794530236	0.469338488624089	0.840448879128388
Nipa1	70.0447836011283	-0.282402961954466	0.390610424194091	-0.722978559871055	0.469693056503917	0.840448879128388
Pdzd11	681.726915081552	-0.106101819393589	0.146669864567313	-0.723405722822461	0.469430656317704	0.840448879128388
RT1-CE3	38.0437343045683	-0.366714664101945	0.50716980499309	-0.723060916662696	0.469642459578985	0.840448879128388
Timmdc1	807.687233597011	0.091827785973259	0.12696585439428	0.723247887483957	0.469527602901234	0.840448879128388
Trpc1	266.34393268356	-0.125652516483967	0.173787066776542	-0.723025705045896	0.469664091907441	0.840448879128388
Ttc39b	139.976908841579	0.210953675206852	0.291848933653514	0.722818043451541	0.46979168043187	0.840493033179386
Cyr61	1267.74748387772	0.408303299570286	0.565221066961683	0.72237806309149	0.470062070269268	0.840844427239612
Smarcd2	713.865400842657	-0.101359880901895	0.140339076168047	-0.722249879859002	0.470140861417182	0.840853033968165
Snx16	165.849262614621	-0.17053302161247	0.236178260180727	-0.72205215451234	0.470262412733535	0.840938103072586
Atp13a2	431.568366538827	-0.147005601526007	0.203748620050007	-0.721504771369383	0.470599006097943	0.84104223902494
Elp5	572.354722519707	-0.108498565668885	0.15035265509818	-0.721627201049664	0.470523710871611	0.84104223902494
Orc6	62.8904744943778	0.218408275250549	0.302724194554978	0.721476113171668	0.470616632078006	0.84104223902494
Stag1	579.109369317178	-0.0937585503829318	0.129946136965754	-0.721518565862725	0.470590522042406	0.84104223902494
Mypn	190.740008964639	-0.225980786204896	0.313350705971927	-0.72117528985284	0.470801672883961	0.841240656185993
Cpvl	34.827596620865	-0.471640776799355	0.654377703047391	-0.720747015986268	0.471065179642773	0.84155839578481
Hars	1088.56544358755	-0.094832791572759	0.131594211233961	-0.720645617185668	0.471127579832725	0.84155839578481
Itpr1	605.70012098246	-0.0896910438493096	0.124505913859813	-0.72037577227293	0.471293662923836	0.841722780137071
Ostc	1220.28864588119	-0.0895047092584268	0.124352815409986	-0.719764236646619	0.47167016796931	0.842262863965309
Pkn2	1044.08264150837	-0.0979586031098612	0.136141120588548	-0.719537217604634	0.471809979311934	0.842380179839687
Pdcd2	412.544608944537	0.114233039571786	0.158923753553281	0.718791477156298	0.472269409762813	0.843068024401466
Agap1	1099.95269939614	0.0778093064796334	0.108383499748153	0.717907307481642	0.472814441862122	0.843414157767693
Aspdh	1854.96251973812	0.0995991921936862	0.138753893923399	0.717811870913486	0.472873292888332	0.843414157767693
Ccdc53	1309.53117968486	0.102167027177425	0.142365056876412	0.717641178383523	0.472978560609689	0.843414157767693
Cdk11b	1946.78322247199	-0.0938559570717388	0.130785198108997	-0.71763439921939	0.472982741651529	0.843414157767693
Mov10	354.417497222011	0.106409954401409	0.14819877545589	0.718021819506064	0.472743833263956	0.843414157767693
Rpl17	8917.75168408704	-0.12439978639796	0.173276552154288	-0.717926256330359	0.472802757521109	0.843414157767693
Tagln2	2854.63819106263	0.0966250434562762	0.134579622344905	0.717976776667145	0.472771606176151	0.843414157767693
Derl1	2372.34386865999	0.0623504409725518	0.0869411023178448	0.717157239904862	0.473277080217478	0.843525288375363
Eif4b	5952.95155908177	0.0853092210149509	0.118949885821152	0.717186237094991	0.47325919025269	0.843525288375363
Ocrl	221.109351013475	-0.117215785968315	0.163468984547654	-0.717052144739692	0.473341922334864	0.843525288375363
Rfesd	123.172647294231	-0.158893344734416	0.221522044249294	-0.717280057941332	0.473201309550877	0.843525288375363
Aig1	160.587096395642	-0.130999534301403	0.182998142081408	-0.715851717462394	0.474082914291251	0.844690350233212
Enthd2	396.558578688963	-0.101987208868161	0.142489535731652	-0.715752271522818	0.474144328214666	0.844690350233212
Ctns	152.644756079515	0.1887535829576	0.263818881979697	0.715466541064814	0.474320808495429	0.844739941924011
Scamp2	2242.08740801667	0.0644326786330163	0.0900448199301928	0.715562302006575	0.47426165811043	0.844739941924011
Zfp40	78.1427623999537	-0.245690605614552	0.343471314478242	-0.715316229501652	0.474413662318114	0.844772941549066
Farp2	718.579645935658	-0.0957665374996547	0.133966852239067	-0.714852487007436	0.474700198624527	0.845150760896988
Adrbk1	684.368014616958	0.092922632220697	0.130187620057287	0.713759358837712	0.475375994379427	0.845622852171906
Arhgef7	189.681514780715	0.128928117998753	0.180587935947512	0.71393538733521	0.47526713406839	0.845622852171906
Cd302	1026.36368165305	0.101453522249811	0.142151189498699	0.713701535721164	0.475411756587936	0.845622852171906
Esam	711.561215062958	-0.344880941573842	0.482857800463124	-0.714249497974468	0.475072914383411	0.845622852171906
Fastkd1	438.65457846566	-0.151956232902841	0.212856803431764	-0.713889480876065	0.475295522422508	0.845622852171906
Srr	710.491442639851	0.0867063764478143	0.121447775896699	0.71393959920324	0.475264529514166	0.845622852171906
Caln1	39.3087421980175	0.375555505897005	0.527036112819439	0.71258021369338	0.476105559074256	0.845692297935956
Cep55	26.4455033824125	0.303487979757546	0.42580922158837	0.712732285659442	0.476011434133989	0.845692297935956
Cldn2	9438.08352756417	0.105867194724986	0.148389236794512	0.713442544836254	0.475571954337253	0.845692297935956
E2f6	643.141202380047	0.086806255150567	0.121801310483282	0.712687366056557	0.476039236059706	0.845692297935956
Eif2d	729.828036151461	0.0834397634589575	0.117137185713964	0.712325150637544	0.476263453248639	0.845692297935956
Exoc7	252.677549652104	0.114247786749047	0.160389241861851	0.712315772696603	0.476269259113853	0.845692297935956
Kras	92.2556378062326	0.188435635352537	0.26425553871757	0.713081119385477	0.475795562047392	0.845692297935956
LOC685322	2116.5200146583	0.098278546533985	0.137923029604107	0.712560816102159	0.476117565946496	0.845692297935956
Ppp1r2	1982.48022462312	-0.0853046210722003	0.119672008966867	-0.712820164118896	0.475957046414098	0.845692297935956
Rp2	301.65784670479	0.123695207783344	0.173397436396392	0.713362379248634	0.475621546432527	0.845692297935956
Serpinb12	387.094325247328	-1.10752771193229	1.55480097545734	-0.712327641553298	0.476261911134002	0.845692297935956
Adcy9	20.5772919392868	0.341346110873485	0.479394157938778	0.7120364427075	0.476442209554139	0.845735148179344

Tbrg4	961.929979423312	0.117952502314546	0.165629684861818	0.712145907981119	0.476374428745993	0.845735148179344
Pwp2	274.226893159594	-0.115506521781391	0.162340258748159	-0.71150879438093	0.476769003071153	0.846183086962324
Coro6	270.188961458264	-0.177833891883024	0.250330879292282	-0.710395347093351	0.477459007726566	0.846524259083701
Ctnnbl1	659.433074075459	-0.0914798686186541	0.128845436001964	-0.709996965800635	0.477706017751439	0.846524259083701
Efna4	111.132175014345	-0.148135135201255	0.208434964051909	-0.710701949047102	0.477268951626229	0.846524259083701
Klhl24	1214.64294732594	-0.12019730147397	0.16926959648273	-0.710093861931278	0.477645932405193	0.846524259083701
Nmd3	552.430242865725	-0.106042086385189	0.149350167704017	-0.71002321601234	0.47768973957183	0.846524259083701
Nsmf	76.7269350728544	-0.237280715549361	0.334037913700679	-0.710340670376659	0.477492905022917	0.846524259083701
Nsun6	63.1146623854722	-0.20595414948119	0.289825857953418	-0.710613438481711	0.477323813212291	0.846524259083701
Slco1a2	306.677818305536	-0.227747151715383	0.320766179976935	-0.710009863670041	0.477698019536707	0.846524259083701
Tfeb	664.073253103236	0.106012561872805	0.149306379131629	0.710033707128779	0.477683233946195	0.846524259083701
Ubfd1	354.26994892141	-0.129573385101153	0.182405202782107	-0.710360138443724	0.477480835477941	0.846524259083701
Zmiz2	1354.36630687702	-0.0967889483274731	0.136348535115503	-0.709864233198264	0.477788332024089	0.846538142133406
Ccrl2	20.5792970241643	-0.433821052670994	0.611679951099583	-0.709228824471258	0.478182488886147	0.846736695393569
Nfyb	360.373954583681	-0.0996731373846399	0.140506624456351	-0.709383901081503	0.478086275341628	0.846736695393569
Prkacb	122.055580450411	0.246680678866645	0.347886818449992	0.709083143666464	0.478272882669955	0.846736695393569
Scamp4	764.915591276621	-0.0824747368803918	0.116311349129652	-0.709085893144079	0.478271176554444	0.846736695393569
Trim16	227.172409256657	-0.228649118213449	0.322292276895569	-0.709446470191208	0.478047458842341	0.846736695393569
Fbxo22	2180.56102158994	0.153824840898665	0.21704964129471	0.708708109265205	0.478505631502048	0.846803025297577
Frmd4a	51.9109654067856	0.269912977612193	0.38086640718135	0.708681502287687	0.478522146319041	0.846803025297577
Srf	1279.44305007633	0.16828383650368	0.237466783903379	0.70866263372713	0.478533858128307	0.846803025297577
Abcf2	1447.31619246197	0.0717432487468914	0.101535671375157	0.706581714339709	0.479826455675049	0.847112068220348
Cad	478.160557338583	-0.0846866966706874	0.119824787584951	-0.706754406809591	0.479719112477561	0.847112068220348
Calm1	6566.132848554	-0.0900542222323488	0.127227572817245	-0.707820012896939	0.479057036686447	0.847112068220348
Dazap1	1021.51081217743	0.103754011017742	0.146788864993539	0.706824805970867	0.479675357113045	0.847112068220348
Diexf	277.244294149335	0.114315613744058	0.161690498293511	0.707002668372911	0.479564819554457	0.847112068220348
Fubp1	635.149341458781	0.0909855768104161	0.12869925131835	0.706962751363289	0.479589625885651	0.847112068220348
Gpm6a	2030.95721983262	-0.162191169535476	0.229072015327956	-0.70803572100796	0.478923074995594	0.847112068220348
Hsf2	502.720885397778	0.106167996177356	0.150185465082541	0.706912590502723	0.479620799226345	0.847112068220348
LOC498122	198.972628388114	0.147626752175399	0.208830401321256	0.706921747223462	0.479615108540237	0.847112068220348
Nedd4l	2125.16342903531	-0.0753583378574953	0.106628075985732	-0.706740107245103	0.479728000387204	0.847112068220348
Nfe2l2	2291.2586799641	0.109178506624889	0.154274496695216	0.707689922596745	0.479137836844855	0.847112068220348
Nrf1	136.1135845690.151959231319599	0.215052841625511	0.706613454493279	0.479806725461813	0.847112068220348	
P2ry14	188.559421353194	-0.121836825982162	0.172120561671572	-0.707857473848141	0.479033770760299	0.847112068220348
Sh3bp5l	312.887728166292	-0.117553654875559	0.166234428817801	-0.707155886488481	0.479469609019905	0.847112068220348
Vps33b	352.418905873776	0.113948510940258	0.160904764986886	0.708173626489839	0.47883744196595	0.847112068220348
Eif2s2	1795.77984216391	0.0867182086170711	0.122887868967233	0.705669398825639	0.480393755450091	0.847184890648258
Glrx2	313.445458752779	0.117612955501238	0.166695309766368	0.705556477060324	0.480463998338789	0.847184890648258
Hnrnpk	8231.30126634396	-0.0846881261662971	0.119961450471506	-0.705961171971766	0.480212284127218	0.847184890648258
Ing5	158.287488950636	-0.147862326953555	0.209331165042816	-0.706356012127062	0.479966768726234	0.847184890648258
Lipa	1969.63200725427	0.106980100114101	0.151619563696281	0.705582429510218	0.480447854143922	0.847184890648258
Syt8	28.0257955381688	-0.462228876843332	0.654892753940234	-0.705808506907859	0.480307231079685	0.847184890648258
Tmem48	197.7527434156	0.150132790595414	0.212718826679924	0.705780456477027	0.480324677591164	0.847184890648258
Tpd52l2	539.719139792141	0.0895348652858305	0.126777321542911	0.706237237040261	0.480040617016996	0.847184890648258
Gtf3c6	350.880584064095	0.11277057761243	0.159951931211145	0.705027921567056	0.480792860236732	0.847402594361336
RGD1309708	501.456835738403	0.0849315377508129	0.12047051241193	0.704998559816885	0.480811132415648	0.847402594361336
Ythdf2	759.970311912887	-0.123691550204946	0.175398829287961	-0.705201686391396	0.480684731988735	0.847402594361336
Enc1	157.754451928996	-0.152410807685956	0.216303932463966	-0.704614132298986	0.481050401288998	0.847561441799837
Gcm2	45.3322441788366	-0.246090739601388	0.349200074650015	-0.704727053245999	0.48098011221867	0.847561441799837
Mapk8	597.694272780035	-0.0882690381485901	0.125307320430981	-0.70442044283605	0.481170978766408	0.847642489872751
Appbp2	1207.25430430768	-0.0948306856165745	0.134660665067238	-0.704219644015748	0.481295999400639	0.847731338786249
LOC100362431	110.257733310375	-0.169196427520473	0.240335032897554	-0.70400234822443	0.481431311237575	0.847838283781574
Fgfbp3	99.5613756049807	-0.182884082837126	0.259834542886284	-0.703848228975334	0.481527295081843	0.847875946692522
Atrn	1338.77400491361	-0.115557746252963	0.16424747385925	-0.703558743022065	0.48170761202948	0.847930729181828
Tmem53	506.023359766134	-0.137533305563755	0.195478655421876	-0.70357198471073	0.481699363156175	0.847930729181828
Ttc19	345.915250766459	0.0964770713804915	0.13726818060612	0.70283638170542	0.482157721437789	0.848591616887877
Mus81	246.55816735749	-0.126224014339319	0.179638425660625	-0.702655981731784	0.482270165852737	0.848658105756651
Taf13	692.987410832068	0.0936627847534143	0.133327477824024	0.70250173694156	0.482366318884909	0.84869591028574
Spopl	135.041087380089	0.184230103114656	0.262295229914673	0.702376871949169	0.482444164786469	0.84870149775004
Ampd2	1164.26257365818	-0.0835458408467448	0.119177832916836	-0.7010182917577	0.483291599936062	0.848870566061552
Chaf1b	41.6906705893341	0.262310362320869	0.373857811527138	0.70163135350678	0.48290909328197	0.848870566061552
Cyp51	718.406136900753	-0.132537052994328	0.18899784991794	-0.701262226273331	0.483139382556879	0.848870566061552
Eif2c4	61.1057196858139	-0.240789309601602	0.343318908601571	-0.701357552901473	0.483079904940167	0.848870566061552
Ipo9	567.320417936314	0.0878089938393422	0.125199600509188	0.701352028937971	0.483083351425626	0.848870566061552
Ndufaf4	713.176287279925	0.0919144270676244	0.130965948802108	0.701819273699217	0.482791877467579	0.848870566061552
Pfdn6	649.557889358077	-0.0978660162390016	0.139666195607709	-0.700713696776603	0.48348170654942	0.848870566061552
Pla2g12a	676.080769253747	0.108969632541982	0.155488108154243	0.700822936464603	0.483413522205936	0.848870566061552
RGD1562084	30.4239903745075	0.298879224327868	0.426400370523082	0.700935658102785	0.483343169999055	0.848870566061552
RGD1565059	162.582673128809	-0.123338489401869	0.175852660384019	-0.701374031717963	0.483069623633424	0.848870566061552
Rin1	17.0353096512035	-0.361977215475285	0.516704715150511	-0.700549472187118	0.483584220744468	0.848870566061552
Usp1	456.730593473652	-0.088313327583543	0.125797840727942	-0.702025782577102	0.482663084729288	0.848870566061552
Zfand5	1374.06453059623	-0.327664393777935	0.467726601749211	-0.7005468420067	0.483585862682434	0.848870566061552
Zfp513	966.648347419963	-0.0792264251370869	0.112861599637093	-0.701978577229454	0.482692523493039	0.848870566061552
Ccdc127	154.903330444477	0.193966696599052	0.277133327215087	0.699903900220963	0.483987321532701	0.848931224798971
Creg1	1780.39028181305	0.0889107210978931	0.127034658929367	0.699893413712623	0.483993870904217	0.848931224798971
Ctnnd1	2059.67851414131	0.116701383394032	0.166681058096171	0.700147843594193	0.483834979736311	0.848931224798971
Wdr18	746.089584705176	0.113628691728919	0.162260586856471	0.700285225946025	0.483749196382428	0.848931224798971
Zadh2	1134.02442168105	0.0823265222040356	0.117592255028469	0.700101568628855	0.483863876293357	0.848931224798971
Wdr37	520.041085321798	-0.0862437217339439	0.123281139000744	-0.69956947537144	0.484196210972972	0.849155089325536
Gnas	3026.47366914252	-0.103063705856039	0.147456617173794	-0.698942562439001	0.48458792686675	0.849588976159122
Mrpl18	1164.39651750798	-0.106656593595652	0.152625007541763	-0.698814665522411	0.484667862169254	0.849588976159122
Ythdf1	879.978499910835	-0.0775124115393902	0.110909547225069	-0.698879523708576	0.484627325047066	0.849588976159122
Chtop	1830.0794454146	-0.0959911832791375	0.13749481399813	-0.698144028039071	0.485087126116178	0.85008319391839
Whamm	380.085186461544	0.111280360747659	0.15939903634919	0.698124425946221	0.485099383754591	0.85008319391839
Ccdc47	2873.51579339213	-0.0885701204554571	0.126923522043438	-0.697822744196658	0.485288053429662	0.850218370825175
Mad2l2	301.141167997689	0.106221456585422	0.15228387010924	0.697522702235142	0.48547573699238	0.850218370825175
Mms19l	549.337216303826	0.103504491327523	0.148387865709293	0.697526653091024	0.485473265380443	0.850218370825175
RGD1560212	1220.63055482552	0.0736813250259317	0.105608051844173	0.697686622745868	0.485373195847259	0.850218370825175
Ckb	4369.19653134487	-0.183620555836788	0.263292537213971	-0.697401292796856	0.485551692720742	0.850220388147274
Pitpnm2	633.928962383334	-0.103539140789541	0.148533910074825	-0.697074093972092	0.485756425672886	0.850447864170983
Itfg1	3052.95109807149	0.0896171365998895	0.12865644938401	0.696561556213965	0.486077221697474	0.850672383240168
LOC687346	869.177702567744	0.0747486225899238	0.107337156670891	0.696390932164457	0.486184040254103	0.850672383240168
Ormdl2	974.723264603484	-0.0816031602473368	0.117176123691501	-0.69641457386131	0.486169238699455	0.850672383240168
Spg21	1036.89382520595	0.0667094630156941	0.0957860196219388	0.696442583990774	0.486151702479112	0.850672383240168
Sertad4	28.5722360565366	-0.302065208093857	0.434042839225719	-0.695934089438513	0.486470107442951	0.851041902600674
Brsk1	73.5125177257004	-0.183930610662554	0.264737724773071	-0.694765397792159	0.487202336318125	0.851086478994257
Cisd2	1214.47181385784	-0.0937515928876367	0.13496553576657	-0.69463357704729	0.487284964265093	0.851086478994257
Dffb	84.9637515311661	-0.193105635756564	0.278037326846545	-0.694531334863333	0.487349056973532	0.851086478994257
Fbxl15	273.799319511815	0.145388760152811	0.209099493258941	0.695309002842808	0.486861673474626	0.851086478994257
Fzd8	109.235114236192	0.261248471354531	0.376213322769924	0.694415788975925	0.487421494872411	0.851086478994257
Hist3h2ba	41.053934523471	0.335312712476949	0.482584740941323	0.694826595268828	0.487163979056293	0.851086478994257
Ispd	258.536176059136	-0.10875136276727	0.156495148470934	-0.694918429292197	0.487106422526199	0.851086478994257
Pex11g	268.431098556861	0.129290622114139	0.185848222568111	0.69567855063427	0.486630161193903	0.851086478994257
Ptk2	1536.32446382388	0.0874189124330578	0.125836664561915	0.694701442837795	0.487242423650442	0.851086478994257
Ptrh2	338.081298209087	-0.109861843753089	0.158013035708248	-0.695270762065068	0.486885633745139	0.851086478994257

Slit1	80.8455507167634	-0.417512403677281	0.601308321487984	-0.694339972951172	0.487469028528296	0.851086478994257
Stk38l	291.139210292283	-0.1164460215774	0.167677373628533	-0.694464727455541	0.487390813705753	0.851086478994257
Unc13b	129.614845558088	-0.155487508584899	0.223614568478562	-0.695337113511036	0.486844060765934	0.851086478994257
Ctnna1	5181.74240572476	-0.0636402760215295	0.0917930014542569	-0.693302049320648	0.488120017247517	0.851580142407799
Gins1	24.1982810616473	0.301734526170923	0.435220048428771	0.693291881337366	0.488126396954673	0.851580142407799
Las1l	794.883653939605	-0.080782576823089	0.116502979042021	-0.693394945668745	0.488061733288343	0.851580142407799
Pak6	175.504098325626	-0.170878705385843	0.246386528889047	-0.693539156366755	0.487971261707073	0.851580142407799
Zfp458	59.8844630708075	-0.224605450023742	0.323827647077829	-0.69359565821679	0.48793581734666	0.851580142407799
Apaf1	113.248780000371	-0.177451349734415	0.256160296083905	-0.692735573963779	0.488475509936154	0.851763615060365
Nadkd1	529.241625709456	-0.0966753651656548	0.13957385640377	-0.692646657881152	0.48853132206392	0.851763615060365
Pccb	8869.68396319856	0.0990303979116905	0.142909689557581	0.69295789682469	0.488335974163847	0.851763615060365
Sema4g	170.679381549704	0.235637922008001	0.340097673430455	0.692853672391221	0.488401385528973	0.851763615060365
Acap3	294.066643076276	-0.112871817853205	0.163452024911903	-0.690550134903745	0.489848293360957	0.851898806166296
Anapc11	673.601358268717	-0.105223082271545	0.152470039647859	-0.690123007212204	0.490116835908342	0.851898806166296
Atp2a2	3084.32825972978	-0.0980558237593075	0.142021850571852	-0.690427729004268	0.489925243966666	0.851898806166296
Cabp1	104.681469893851	0.254174991336148	0.368310781455434	0.690110103026956	0.490124950224383	0.851898806166296
Cav1	247.858451226812	0.191452852190584	0.277428078492328	0.690099045601394	0.4901319033319	0.851898806166296
Ccnc	812.681275725499	0.115130305739612	0.167024211703731	0.689303093037981	0.490632551923682	0.851898806166296
Dck	14.5106833350213	0.373376787691193	0.541600376963091	0.68939536154835	0.490574501594094	0.851898806166296
Dgcr14	238.519307453003	0.127983808810191	0.185217996007813	0.690990139018632	0.48957173799735	0.851898806166296
Erc1	48.0567142360529	0.28222868409737	0.408686597639477	0.690574845682457	0.489832759691294	0.851898806166296
Gpt2	24.0634960867326	0.328121839783338	0.474099910660078	0.692094287312777	0.488878118977667	0.851898806166296
Gtf3c4	113.65202216004	0.185271410122955	0.268621821837627	0.689710943271563	0.490375982647138	0.851898806166296
Klhl4	17.3224141051974	-0.426019190515506	0.617571524988628	-0.689829717332499	0.490301278161908	0.851898806166296
Krt5	117.876581537854	-0.240003286372521	0.348009805363247	-0.689645184341889	0.490417345210527	0.851898806166296
LOC100134871	177.87639017789	0.88071480837367	1.27287664254368	0.691909002755894	0.488994476588038	0.851898806166296
LOC683722	65.7171956309072	-0.188096143876675	0.272618919085302	-0.689959979695394	0.490219354997484	0.851898806166296
Manea	38.1347894152437	-0.288402091348937	0.418142678848817	-0.689721728819771	0.490369198684138	0.851898806166296
Mbtps2	47.1572447701375	0.26373073898591	0.38205076516142	0.690302868192088	0.490003744556503	0.851898806166296
Mettl2	131.53388934568	0.138971065538334	0.201213640669207	0.690664236659783	0.489776569021558	0.851898806166296
Mfn2	2460.10460405305	0.0983781834297086	0.14239612612624	0.690876824433357	0.489642951468367	0.851898806166296
RGD1359127	543.751737269532	0.115019595145293	0.166467087260546	0.690944961181848	0.489600129707306	0.851898806166296
Rogdi	3746.57885739737	0.0843255325697394	0.122295061242322	0.689525249123937	0.490492789651792	0.851898806166296
Rpl36a	2994.72680617848	-0.130263686511563	0.18816866870052	-0.692270862153383	0.488767244902331	0.851898806166296
Rps19	3405.79556898335	-0.108708770015404	0.157565017642019	-0.689929602663365	0.490238458726213	0.851898806166296
Srgap2	188.26671399929	-0.189294810641873	0.273570032949472	-0.691942785549307	0.488973260080672	0.851898806166296
Tecr	2360.34724811715	0.078124115675604	0.112860320362757	0.692219510138699	0.488799488229696	0.851898806166296
Tlr1	57.7150409950613	-0.23381591795993	0.338234414184182	-0.691283642806993	0.489387309084836	0.851898806166296
Vps9d1	733.595094957323	-0.0804898296891645	0.116375402722065	-0.691639537277438	0.489163725945062	0.851898806166296
Cab39l	538.692806621928	0.0930421189417142	0.13507085813216	0.68883932647172	0.490924384541795	0.852173828876061
Pdzk1ip1	5786.62411710646	0.131092193015208	0.190316059320843	0.688813090618942	0.490940896663773	0.852173828876061
Zdhhc18	479.140549820506	-0.0960875253403889	0.139590393687726	-0.688353423197186	0.491230247067612	0.852545883061609
Hk2	53.6518815971092	-0.277193230242643	0.402922776914146	-0.687956219217924	0.49148035187644	0.852700191512778
Iqcg	51.7654972166895	-0.224083847544251	0.325743149481264	-0.68791576400332	0.491505828880228	0.852700191512778
Siae	336.50982577401	-0.147768487275839	0.214825116912657	-0.687854797425378	0.491544224422992	0.852700191512778
Ltn1	988.209608371578	-0.0722341049259524	0.105076891840351	-0.687440441573982	0.491805220184653	0.85302275791527
Fsip1	24.955016773396	0.310695414296676	0.45231365722457	0.686902571554275	0.492144126337295	0.853350135766545
Zcchc10	93.3889753488164	0.181503980494072	0.264207068106968	0.686976248571016	0.492097695832581	0.853350135766545
Aplp2	2567.71879042293	-0.0786460538696267	0.114572979333161	-0.686427588139574	0.492443512442903	0.853420165175473
Gnai2	3209.04138202859	-0.10019746698565	0.145987406189352	-0.686343223714031	0.492496698272446	0.853420165175473
Lrrk2	640.319137866299	0.117827956291635	0.171784431478571	0.685905907057318	0.492772444983592	0.853420165175473
Myh14	2302.63462378358	0.12968014475851	0.189031733479318	0.686023147392331	0.492698511859649	0.853420165175473
Ptch1	113.230802170832	0.179375561315458	0.261446775561959	0.686088252302615	0.492657458510946	0.853420165175473
Tmem106c	493.722138806054	0.0882366713331824	0.128538451870456	0.686461288814255	0.492422267401527	0.853420165175473
Tuba1c	5770.58138331554	0.116697992833583	0.170051935800548	0.686249129033478	0.492556021975572	0.853420165175473
Xkr6	16.7384498652855	0.345819067787964	0.504193445519449	0.685885687053472	0.49278519655522	0.853420165175473
Mill1	13.1390014372214	0.466682057162459	0.680535015960511	0.685757596916275	0.492865979607751	0.853430031112385
Brf1	741.497391319552	-0.0876633340712225	0.127976647576173	-0.68499476843261	0.493347222248037	0.853743076746527
Cwf19l2	269.744947995616	0.104005965300193	0.151827336422139	0.685027925478561	0.493326299361359	0.853743076746527
Klhdc8a	1288.45413742858	-0.184869255621089	0.269796158842968	-0.685218264092079	0.493206200351899	0.853743076746527
Zfp652	190.195542085489	0.13626064532252	0.198829970122739	0.685312406567307	0.493146804541852	0.853743076746527
Adpgk	459.997692367907	0.0948532702218476	0.138552932125289	0.684599515628254	0.493596672786334	0.853847746221799
Ikbke	125.579594271544	-0.20360841709705	0.297437503420725	-0.684541844103117	0.493633075877481	0.853847746221799
Rbp1	288.247742582071	0.177418523131426	0.259121804169529	0.684691601696905	0.493538549722952	0.853847746221799
Bcas1	9.00958855683263	0.533125383641849	0.780187870045409	0.683329495510894	0.494398660195876	0.854147636807568
Card10	584.924963927777	-0.0903182377011754	0.132196334111622	-0.683212876575788	0.494472337186309	0.854147636807568
Chmp4bl1	2114.92118581363	-0.0619471078391509	0.0907616707489189	-0.682524983597096	0.494907050567713	0.854147636807568
Fancb	26.2319659742051	-0.276540133708115	0.405286473617202	-0.682332502340828	0.495028725491685	0.854147636807568
Fra10ac1	314.080609044629	-0.0998602260298772	0.146352537500827	-0.682326577558063	0.495032471031947	0.854147636807568
Fxyd6	1362.52777383866	-0.123767018088202	0.181420763975518	-0.682209772332913	0.495106316269667	0.854147636807568
Gbe1	1532.61630623916	0.0715653273116872	0.104900457747907	0.682221306256549	0.49509902416507	0.854147636807568
Map6	31.258468567504	-0.294250034627209	0.430783272633854	-0.683058171753358	0.494570084967961	0.854147636807568
Mcee	997.957195173646	0.104932565099786	0.153698670907919	0.682716151544676	0.494786221658048	0.854147636807568
Mrps9	1711.36973152491	0.104920518823441	0.153767785498353	0.682330947821089	0.495029708229419	0.854147636807568
Nphp3	143.245185317961	0.164923489241147	0.24175987458237	0.682178916274034	0.495125824710102	0.854147636807568
Nup62	448.691886774898	0.0892260055707178	0.130780035304045	0.682260142867218	0.495074470875994	0.854147636807568
Per2	933.363529972622	-0.346762681268834	0.508355592786395	-0.682126224614076	0.495159139444401	0.854147636807568
Pycr2	222.40255954454	0.115777188546092	0.169643518270814	0.682473399079527	0.494939657589407	0.854147636807568
Rab11a	3004.11567656533	-0.0985155838453921	0.144125634429736	-0.683539637033969	0.494265912743828	0.854147636807568
RGD1305455	278.37183808375	-0.105737897161492	0.154696893420174	-0.683516616421613	0.494280454050003	0.854147636807568
Smap2	645.572409347255	-0.0872814548222693	0.127894058038726	-0.682451211266127	0.494953683052088	0.854147636807568
Ten1	272.062785965516	-0.17186063578861	0.251716257143441	-0.682755407771198	0.494761411463379	0.854147636807568
Eif1b	1310.50042663129	-0.122892480136701	0.18022550094007	-0.681881750893657	0.495313725625387	0.85415503041392
Hpdl	363.201492384327	0.568852673328611	0.834185776254253	0.681925644768162	0.495285968652102	0.85415503041392
Aldoc	24.7678971705238	-0.385926088148713	0.56645505446179	-0.681300458189743	0.495681393437572	0.854264505303168
Bbs2	1938.61241800141	-0.0946952138969483	0.13934661728612	-0.679565932357803	0.496779346938992	0.854264505303168
Caap1	153.966992012458	-0.158071832342186	0.232716703151863	-0.679245753318504	0.496982161654864	0.854264505303168
Ccdc82	173.275182634172	-0.15578420663856	0.228585725873433	-0.68151327491384	0.495546769891913	0.854264505303168
Cdca8	85.3052985771288	0.186048827389955	0.274196322199592	0.678524153414892	0.497439414761251	0.854264505303168
Cyth2	1148.49144992325	0.0878750068084778	0.129279116099966	0.679730875793798	0.496674882128484	0.854264505303168
Dd25	329.491112922664	0.170294882693192	0.250791678764339	0.679029238658323	0.497119336044949	0.854264505303168
Dusp12	107.115077323659	-0.149792972351511	0.220703017908799	-0.678708310247978	0.497322699605542	0.854264505303168
Emc10	1767.51122884025	0.073334967112779	0.107995064632173	0.679058504780336	0.497100793107997	0.854264505303168
Emc6	682.931949572989	0.128503969577918	0.189082488582395	0.679618565110647	0.496746011387541	0.854264505303168
Fam13a	130.180403981891	0.21449436292852	0.315738732539355	0.679341306033096	0.496921629974045	0.854264505303168
Gemin5	357.482179715727	0.172382274290289	0.253629551189956	0.67966163044299	0.496718736357435	0.854264505303168
Hebp1	108.925110065272	0.207591136235843	0.305916540224485	0.678587486912314	0.497399273529828	0.854264505303168
Kntc1	38.3582106854812	0.271234923370035	0.398838820503067	0.680061492078225	0.496465525809506	0.854264505303168
Lepre1	422.132242921349	0.0850294770046857	0.124899474484877	0.680783304776682	0.496008615551162	0.854264505303168
Lgals3	639.828242404421	-0.0993217993812208	0.145964647103938	-0.680451063677741	0.496218897590928	0.854264505303168
LOC500251	325.489017285704	0.110144310245252	0.161891366283645	0.680359384034546	0.496276931842115	0.854264505303168
LOC679383	1378.64700095777	0.0928328079497103	0.136379034059637	0.680697063077274	0.496063195078798	0.854264505303168
LOC690276	60.1538728642211	0.284750042488385	0.417923040861788	0.681345641774643	0.495652809585285	0.854264505303168
Mastl	15.9232251641051	0.390157197905369	0.573248798288872	0.680607092539878	0.496120137879989	0.854264505303168
Mbd2	1725.00117904969	0.105422897599229	0.154805722121915	0.681001297330629	0.495870669539402	0.854264505303168

Mpp6	2229.25766291877	0.0884248618946245	0.129973944895348	0.680327599241694	0.496297052820614	0.854264505303168
Mpzl2	1065.80304968468	0.116253505304837	0.170975176328144	0.679943766115601	0.496540068085858	0.854264505303168
Myo10	784.847621146157	0.13776205219001	0.203051860344784	0.678457473652733	0.497481678743768	0.854264505303168
Park7	5618.62467081345	0.114226619607113	0.167945869901714	0.680139497767709	0.496416137088829	0.854264505303168
Poli	42.4547210531374	-0.240413965177056	0.35389179892903	-0.679343137943892	0.49692046951542	0.854264505303168
Rpain	122.977259484705	-0.137577069316124	0.202761235223313	-0.678517612918478	0.497443560273734	0.854264505303168
Usp31	28.3193506889478	-0.317046312647741	0.465838314525262	-0.68059303574212	0.49612903481226	0.854264505303168
Fbxo3	1603.04642949659	-0.0663182497797161	0.09778491417424	-0.678205327884684	0.497641514899871	0.854325018463447
St3gal1	14.4867553595217	-0.427148854522847	0.629860020162678	-0.678164736368764	0.497667248569988	0.854325018463447
Zfp629	684.862148384149	-0.105415811912198	0.155500112168645	-0.677914700137779	0.497825778839113	0.854468106657408
Dio2	8.99205708176919	0.753697202229078	1.11227100886231	0.677620108969663	0.498012592721971	0.854533713149105
Mospd2	420.546607359638	-0.0782029052712146	0.115428852543411	-0.677498766972527	0.498089552139083	0.854533713149105
RGD1565785	20.3361237030132	-0.43669529049972	0.644553633168975	-0.677515831153863	0.498078729045076	0.854533713149105
Acsm5	1124.31385346986	0.121559590402221	0.179565740039581	0.676964271555508	0.498428623480421	0.854857361472531
Sbf1	1315.63382561309	-0.0725096653483648	0.107100584088253	-0.677023995393112	0.498390729989666	0.854857361472531
Ifnar1	322.875828619974	-0.123697311406588	0.182837523657538	-0.676542259663716	0.498696424608413	0.855187622525532
Tmem126a	614.85925830545	0.119122531649424	0.176277200071465	0.675768230951765	0.499187807991651	0.855901135259181
Acvr2b	26.6618958694028	0.289746824707354	0.431968128288938	0.670759729091741	0.502373605723645	0.855926385319032
Apoc2	118.077179608642	-0.341114493026328	0.507586051669541	-0.672032834441257	0.501562793509564	0.855926385319032
Aqp8	38.7196395056369	-0.383156832881582	0.571335373031977	-0.670633836039655	0.50245382182931	0.855926385319032
Ccdc32	440.329306853388	-0.0797438949589903	0.118369458209902	-0.673686406653838	0.500510705809866	0.855926385319032
Ccng2	724.701489009922	0.0975918700350797	0.145045021764961	0.672838466619166	0.501050062651739	0.855926385319032
Chst11	26.9817113470575	-0.305631122142904	0.452786290044895	-0.675000831214654	0.499675236844464	0.855926385319032
Clec1a	78.6264596376134	-0.305251886350605	0.454021013852669	-0.672329863678205	0.501373721993155	0.855926385319032
Dnmt1	401.371133420619	-0.109295403646028	0.162554557867463	-0.672361360271056	0.50135367530997	0.855926385319032
Dyt1	707.630428593412	0.123558816878929	0.182952603944236	0.67535970636739	0.499447258872341	0.855926385319032
Ehmt2	2720.82068703344	0.069864137219611	0.104357675415907	0.669468124325065	0.503196907655742	0.855926385319032
Fam120b	838.398887961321	0.0668614274348793	0.0989690626655113	0.675579071217971	0.499307933009471	0.855926385319032
Fam134a	1177.92031049752	0.0678284320681716	0.100964932970933	0.671801882815084	0.501709829950828	0.855926385319032
Fam134b	516.44156848127	0.0762766574790135	0.113682787424991	0.670960478773815	0.502245706738214	0.855926385319032
Fcho2	440.243810600777	0.143953229499813	0.213894428570666	0.673010655124447	0.500940512247943	0.855926385319032
Gabarapl2	3569.5071163954	-0.0881801338380808	0.130962688026358	-0.673322571237491	0.500742096159243	0.855926385319032
Gpbp1l1	1934.12644491325	-0.0849051364071391	0.126066107900757	-0.673496928087756	0.5006312024283	0.855926385319032
Ier5	289.142887411454	0.151919685116437	0.226326543539116	0.671241131247076	0.502066930017971	0.855926385319032
Kctd13	149.794596963814	0.131448340750828	0.194704504717428	0.6751171008683	0.499601369677657	0.855926385319032
Lbp	70.4116617126493	-0.290495733913949	0.433701793924409	-0.669805239414297	0.502981953154196	0.855926385319032
Mbip	111.084948337092	-0.179479718397474	0.267878944379608	-0.670003082224841	0.502855825353597	0.855926385319032
MGC114529	27.484353161772	-0.345959259828521	0.516813647523309	-0.669408134801467	0.50323516382683	0.855926385319032
Mocs3	208.923785192111	0.111480596797883	0.16540636982534	0.673980070511191	0.500323984198273	0.855926385319032
Mrpl17	1267.46966687945	0.0820021130741033	0.121643449031234	0.674118612446181	0.500235907284838	0.855926385319032
Mrpl44	882.161349594853	0.0818691394935473	0.121919687719134	0.671500567505958	0.501901698062236	0.855926385319032
Mrps11	956.434979647972	0.0899536972320903	0.133942453906415	0.671584658997963	0.501848147338972	0.855926385319032
Mto1	450.935503376026	0.125879407911887	0.187212717929926	0.672387054169066	0.501337322190993	0.855926385319032
Mtor	1280.54947722033	0.0599466564705111	0.0894499982150998	0.670169454071511	0.502749773711324	0.855926385319032
Myo1d	2739.84031556454	0.101683963610351	0.151853898248561	0.669617077883047	0.503101924500231	0.855926385319032
Nup153	752.948466662033	-0.0613609186408174	0.0910177198529359	-0.674164533455274	0.500206715190117	0.855926385319032
Pqlc1	816.339376646976	0.141320342068649	0.20961804854321	0.674180219932339	0.50019674346641	0.855926385319032
Prr22	68.3169349686735	0.173915354754855	0.259116614141399	0.671185656431704	0.502102265021639	0.855926385319032
Ptpn4	547.915910369085	-0.0892049225431727	0.132734298382709	-0.672056308204309	0.501547850105153	0.855926385319032
Qrich1	706.212790949876	-0.0679806572582902	0.101414841551375	-0.670322570329635	0.502652182125562	0.855926385319032
RGD1566386	162.87174678432	0.153293546889615	0.227509998276848	0.673788176566544	0.500445992804894	0.855926385319032
Ripk4	1127.16228280649	0.112800816211159	0.168521661123257	0.669354998397838	0.503269050949067	0.855926385319032
RT1-Cl	27.396292306589	-2.50886902105585	3.73799796732457	-0.671179878369903	0.502105945467091	0.855926385319032
Sall2	28.8077876118453	-0.336021552408799	0.499250576161979	-0.673051907104418	0.500914268646479	0.855926385319032
Slc25a5	22310.2566548128	0.0843442209530482	0.125105055939971	0.674187148707394	0.50019233895267	0.855926385319032
Smek2	1505.84342465638	0.0924907530169272	0.137906191001802	0.670678758836278	0.50242519729771	0.855926385319032
Smo	118.214061985334	0.213480405457109	0.318152558801418	0.671000121015392	0.50222045243357	0.855926385319032
Smpd1	1727.36957073086	0.106697351248714	0.158487264986118	0.673223500058884	0.500805112805923	0.855926385319032
Sntg2	56.051033469863	-0.245405663023791	0.364231610453966	-0.673762671828305	0.500462210229132	0.855926385319032
Stard10	5208.84077678444	0.0879552261427466	0.130403775410618	0.674483739951482	0.500003819837602	0.855926385319032
Tgfa	278.085689471401	0.164663019766632	0.245213867301195	0.671507780448555	0.501897104633646	0.855926385319032
Tgfbi	3082.96291053965	0.087925386691113	0.130676277365897	0.672848878644744	0.501043437913983	0.855926385319032
Tmem17	190.212276292122	-0.132083727598418	0.196251955302661	-0.673031396781338	0.500927316771397	0.855926385319032
Ttc17	550.413739535607	-0.0972787649724293	0.144943926375572	-0.671147576893739	0.502126520764546	0.855926385319032
Ube2d4	25.7249092670393	-0.306084823750262	0.457226371435238	-0.669438253942918	0.503215956230576	0.855926385319032
Ufm1	1471.22561727693	-0.101980666186655	0.152177633779169	-0.670142278165815	0.502767095842898	0.855926385319032
Vtn	21.6049648974649	-0.471341109307615	0.698431896527876	-0.674856219555264	0.499767118034351	0.855926385319032
Vwce	200.599453181444	0.11991595367916	0.178425669992829	0.672077922890688	0.501534090397969	0.855926385319032
Wdr93	26.9442537251092	0.292914046090283	0.433873996636078	0.675113162718464	0.499603871525147	0.855926385319032
Ydjc	16.9398355207484	0.362502290519476	0.537477424249143	0.674451193975063	0.500024504846168	0.855926385319032
Zfp668	69.7102119572455	-0.187472976327978	0.279693105201927	-0.670281007437168	0.502678672042414	0.855926385319032
RGD1565222	234.701782994721	-0.137873563538142	0.206117096745823	-0.668908914956059	0.50355358299274	0.856026028162651
Tfr2	59.2408967781924	0.265205276259108	0.396436328727659	0.668973192013635	0.503512578963113	0.856026028162651
Tsc1	174.171818630959	0.160756797948757	0.240244011953377	0.669139666132257	0.503406388930695	0.856026028162651
Abo	86.4491433903334	-0.235506057158856	0.352276255835247	-0.668526627207592	0.503797490847268	0.856141842641383
Asah1	1355.12251002069	-0.0654373954560602	0.0979074539768916	-0.668359688645411	0.50390402081925	0.856141842641383
Dlg1	497.713682468822	0.123112596242028	0.184209310350088	0.668329934073656	0.503923009613835	0.856141842641383
Glt25d1	949.408412661184	-0.114362610696248	0.171055603111666	-0.668569801958443	0.503769941298879	0.856141842641383
Ftsj3	664.556339594701	-0.0999433486404127	0.149616842020975	-0.667995309153777	0.504136586793097	0.856299189787416
Hey2	49.9791782670819	-0.315320782014859	0.47219437445859	-0.667777506617693	0.504275626753102	0.856299189787416
Pcsk6	580.14499264699	-0.409542054364346	0.613350670150371	-0.667712736441524	0.504316978394946	0.856299189787416
Rpl24	5199.40358900627	-0.108658127433138	0.162683506882081	-0.667911145485063	0.504190312480131	0.856299189787416
Pip4k2a	1000.81810288408	-0.110373584296686	0.165373831245725	-0.6674186808473	0.504504736696026	0.856383877896557
Rheb	1917.7680586951	-0.112715483640089	0.168887798671107	-0.667398619243013	0.504517547632883	0.856383877896557
LOC686999	391.746810010648	0.138434775408399	0.207476638477587	0.667230664735076	0.504624806731269	0.856438039914529
Cep97	68.1062114076437	0.17956084708063	0.269479744030102	0.666324096925714	0.505203966524806	0.856640848156874
Cfl1	7422.70721163842	0.0707176709994358	0.106148593809287	0.666213922027942	0.505274375485203	0.856640848156874
Dnajc6	183.780793978585	0.168676316519587	0.253247187266981	0.666054057065451	0.505376548834656	0.856640848156874
Fam207a	472.979882718588	-0.0819302617983772	0.122944255154993	-0.666401709417732	0.505154370172853	0.856640848156874
Fam86a	267.013743583239	-0.114121438640319	0.171316480228212	-0.666143960512712	0.505319088148861	0.856640848156874
Khsrp	262.505871223065	0.129916855168868	0.195016658882902	0.666183370759506	0.505293900657769	0.856640848156874
Mpg	154.857317620316	-0.137130769727067	0.205873070701317	-0.666093769621855	0.505351166616958	0.856640848156874
Slc22a7	1026.14476356421	0.106470743317391	0.1598421346839	0.666099358144493	0.505347594775431	0.856640848156874
Tsen15	332.457686627572	0.136876036114274	0.205525136602622	0.665981973674199	0.505422622535632	0.856640848156874
Skap2	774.699610330932	-0.0797933869824852	0.119845341155006	-0.66580299420469	0.505537030803775	0.856707006428465
Pacrg	47.566385417683	0.239478082026243	0.359832348066245	0.665526830239717	0.505713588642918	0.856878450881843
Cep120	425.376952139792	-0.0920417321879069	0.13847684789796	-0.664672351985729	0.506260081343443	0.857171566937763
Creb3l2	164.249431981189	0.221421750439739	0.333200854789744	0.66452935896416	0.506351564724427	0.857171566937763
Extl1	41.7165931805122	-0.233683081812237	0.351564818051711	-0.664694161114451	0.506246129169108	0.857171566937763
Guf1	786.634388052518	0.0731527354576979	0.110098315494877	0.664431014488152	0.506414488121334	0.857171566937763
Klhdc3	1073.51846391769	0.0648433733749319	0.0975888309978937	0.664454863449809	0.506399228547205	0.857171566937763
Nomo1	2809.60974958965	0.0747552237498793	0.112500552568137	0.664487614001745	0.506378273753247	0.857171566937763
Nupl1	188.662256118494	-0.136570478889473	0.205413049182372	-0.664857853155285	0.506141415261745	0.857171566937763
Nagk	614.227786195501	0.0955715267293758	0.143924681366802	0.664038480556404	0.50666568266861	0.857341394850592
Nhlh2	33.1291162977805	-0.972401056938523	1.46426335887696	-0.664088909309539	0.506633408090564	0.857341394850592

Ctu1	110.989918370085	-0.161373530077211	0.243294591694256	-0.663284493721943	0.507148365732706	0.857395462919273
Eml2	684.536658086025	-0.0769974574632006	0.116085630045878	-0.663281557181286	0.507150246102962	0.857395462919273
Tradd	387.814258742942	-0.0950170847049097	0.14323797191406	-0.663351228973826	0.50710563379134	0.857395462919273
Tspan9	593.204247708503	-0.315791376494504	0.475968903642708	-0.663470605070362	0.507029199563134	0.857395462919273
Txndc17	2090.77643390352	-0.0650299233034274	0.0980090169821025	-0.663509596421139	0.507004235459534	0.857395462919273
Vps29	2147.44501048063	0.123762020751778	0.186562047973672	0.663382623079072	0.507085532160414	0.857395462919273
Slc35f1	112.032774129127	-0.36492118895679	0.550391150108421	-0.66302154183421	0.507316757592531	0.857549415050076
Tomm20	4176.70589271311	0.0944540499500095	0.142492328224299	0.662871125253339	0.507413096125827	0.857584721273777
Ccdc134	166.07889624669	-0.119878247264586	0.181111881054712	-0.661901618858302	0.508034274230181	0.857996665809842
Lmbr1l	68.6753496659302	-0.195137888306506	0.294685647970622	-0.662189996867304	0.507849464193213	0.857996665809842
Lrrc8c	54.5557918299414	-0.398706087006386	0.602228794466229	-0.662050852881868	0.507938631653837	0.857996665809842
Mynn	150.901127975428	-0.12972729880017	0.195985344098477	-0.661923468802779	0.508020270231345	0.857996665809842
Serpinb8	431.895983786444	0.14541571967043	0.219675752679183	0.661956169021514	0.507999312486667	0.857996665809842
Polr2m	2471.37956230158	0.064754336065434	0.0978592444866725	0.661708931078585	0.508157779854096	0.858067295836048
Wdr20a	328.794804355699	-0.0959668856256141	0.145052550085955	-0.661600816867725	0.508227083896557	0.858067295836048
Catsperd	22.4155366106301	-0.303300554044911	0.458643762653327	-0.661298765495619	0.508420733012615	0.858068980485696
Egfr	112.92106163883	-0.200131079564465	0.302628719653485	-0.661308945805336	0.508414205651873	0.858068980485696
Ptgds	139.174906397717	-0.2038972433097	0.308353083284544	-0.66124600129763	0.508454564804783	0.858068980485696
Usp6nl	376.703926310806	-0.0956969527066046	0.144772154148312	-0.661017674770298	0.50860097873289	0.858188646715859
Cfdp1	1023.80139764661	-0.0733261681294058	0.110997726786957	-0.660609638160824	0.508862686424133	0.858371993039263
Cnnm1	135.820863034463	-0.19515296973628	0.295464701791187	-0.66049503901214	0.508936201046243	0.858371993039263
Cox6a1	9139.50921732278	0.100836620962968	0.152643463057021	0.660602288126153	0.508867401257424	0.858371993039263
Spo11	388.599416533306	0.0946538577288462	0.143357743463583	0.660263306619996	0.509084872898575	0.858495350944392
Tmem86a	492.579752942453	-0.203931737747093	0.308928292624001	-0.660126452047887	0.509172684958865	0.858516056555771
Nqo2	1291.55951600443	0.11433029428728	0.173307854214679	0.659694823442089	0.509449689286071	0.858855705788414
Mrpl40	721.690385062911	0.0846986581175706	0.1284719531439	0.65927742238573	0.509717637892628	0.859052590789978
Rpl10a	5163.91198969023	-0.0970652693225506	0.147214061520434	-0.659347811751511	0.509672446606602	0.859052590789978
Acpp	84.0661133583596	0.186562879796556	0.283090808392517	0.659021325545401	0.509882074536551	0.859202321598583
Lrrc39	18.9041896093841	-0.42980590665215	0.653259410079705	-0.657940628210328	0.510576282665881	0.859252696406181
Ofd1	454.669289462385	0.0797725971688811	0.121227925122072	0.658038130146604	0.510513630022542	0.859252696406181
Psen1	567.157406863536	0.176156945306493	0.267716693323198	0.657997613521354	0.510539664644796	0.859252696406181
RGD1561648	27.4690017609141	-0.277043060706368	0.420897794876136	-0.658219320887384	0.510397211432622	0.859252696406181
Rph3al	251.633579076468	-0.106764327847254	0.162251479086609	-0.658017593727229	0.510526825948978	0.859252696406181
Ybx1	9779.81564801823	-0.0938843745070084	0.142699720413547	-0.657915616337083	0.510592355404483	0.859252696406181
Zfp157	147.388343862042	0.153245386505609	0.232670730944413	0.65863628778568	0.510129354797299	0.859252696406181
Zfp287	340.964953240737	0.179852280942778	0.273110049828854	0.658534100284789	0.510194992534189	0.859252696406181
Zfp978	24.4630730389185	-0.286919572848113	0.435761486149499	-0.658432610425047	0.510260186529742	0.859252696406181
Mafk	1009.42003492895	-0.0897872904338216	0.13649804147986	-0.657791785584478	0.510671933481136	0.859259392442131
LOC304131	653.744084908773	-0.118388397785231	0.18003718035586	-0.657577493444548	0.510809660599957	0.859363913910467
Swsap1	247.063212658776	0.101420405130432	0.15434212779642	0.65711420840464	0.51110748360285	0.859737702919934
RGD1305045	1424.46083451544	-0.0843872600962749	0.128482444889484	-0.656799924447747	0.511309572816174	0.859950370616844
Ace	798.771602026294	0.335900394927344	0.511893243620412	0.656192280545954	0.511700415093125	0.86015341732045
Cdc5l	1326.77653762344	-0.0624347159174464	0.0952387757443456	-0.655559832951268	0.51210737688349	0.86015341732045
Erf	1064.3334741362	0.120677943273311	0.184055857246282	0.655659347541622	0.51204333092354	0.86015341732045
Eri1	363.127160739401	0.107482505480728	0.163891137399589	0.655816459548211	0.511942224716778	0.86015341732045
Hdlbp	7264.61246243524	0.0618317885531012	0.0943414680916503	0.655404137796893	0.512207588116375	0.86015341732045
Itga1	118.306646399018	0.248588141632078	0.378970901830604	0.65595574866377	0.511852596777188	0.86015341732045
LOC681996	1949.49866978895	-0.0897913409143147	0.13689506727214	-0.655913633000482	0.511879695950141	0.86015341732045
Mllt11	78.5720102226412	-0.24193742491258	0.369219185655344	-0.655267749651617	0.51229538103795	0.86015341732045
Olr59	43.3911219004734	-0.251569758425843	0.383864901518409	-0.65536014736106	0.512235903861472	0.86015341732045
Parm1	475.27808078315	0.183117451665709	0.279126367836645	0.656037812138466	0.511799795478397	0.86015341732045
Rpl18	7098.55219698484	-0.103631824631533	0.158168020203328	-0.655200871189462	0.512338433508662	0.86015341732045
Slc25a3	5607.79730384762	0.0658618799844102	0.10040420313358	0.655967359222862	0.511845126136095	0.86015341732045
Ankrd52	86.4258970865322	0.175457965268909	0.268100990395079	0.654447284996412	0.512823678911879	0.860643407784043
Olr1668	612.156010431774	0.423406726221774	0.647020261326061	0.654394849017566	0.512857452131033	0.860643407784043
Ppp1r37	1293.8752912935	0.0843421076582017	0.128871330752633	0.654467577587877	0.512810609072351	0.860643407784043
Dis3l	934.797169311591	-0.0761649352958419	0.116425522277041	-0.654194491089362	0.512986510309277	0.860732901708776
Taco1	162.101079499234	0.132919559112441	0.203255280336937	0.653953781137197	0.513141583134837	0.860738966043471
Tmub2	866.11819477171	-0.0619565187411369	0.0947314670943531	-0.654022582374111	0.51309725674977	0.860738966043471
Hdgfrp2	952.77929791627	-0.0753102310853729	0.115258440161279	-0.653403177936407	0.513496390592226	0.861207020137412
Creld1	702.517862034252	0.079552977370815	0.121887770591276	0.652673988414952	0.513966475160903	0.861386730512197
Dctn6	968.420066428205	-0.0816650662364182	0.125056201416421	-0.65302692158771	0.513738922767445	0.861386730512197
Fbxl5	1431.12255071069	0.0877160164076913	0.134369696418122	0.652796119556174	0.513887725614548	0.861386730512197
Hnf1b	5019.2454841907	0.123514919440747	0.189251628456115	0.652649176381532	0.51398247460265	0.861386730512197
Ilvbl	498.862752645193	0.120642048751307	0.184791786805783	0.652853954370287	0.513850436207332	0.861386730512197
Syn3	16.9860475935691	0.330235262761148	0.506350226096569	0.6521874499927	0.514280255001501	0.861723641357096
Tmem174	8178.33887610599	0.124383899069408	0.190742904353605	0.652102365175385	0.51433513839227	0.861723641357096
Mettl5	388.409967338265	-0.162323736298612	0.249061442689251	-0.651741733067613	0.514567795519273	0.861885914743354
Pex10	401.95459306051	0.0831688354835111	0.127614924671517	0.651717153754459	0.514583654535316	0.861885914743354
Socs1	25.9962548168092	0.449667733223293	0.690408121564278	0.651307131504288	0.514848245746133	0.862095579145364
Srm	285.687690715753	0.12437005933480 4	0.190960129498697	0.651288096951422	0.514860530638636	0.862095579145364
RGD1311251	50.0697250028875	0.245297265047258	0.37672606921881	0.651128990239282	0.514963224001229	0.862140522020617
Sugp1	708.527899098962	0.0812519329790625	0.124821189347319	0.650946633371489	0.515080936960163	0.862210593444656
Crlf2	347.407718117169	0.106543996589015	0.163710019085306	0.650809261304262	0.515169621063909	0.862232059050565
Zfp84	424.561260467256	-0.08516848018152	0.130927488441183	-0.65050113765667	0.515368567118676	0.862438035022213
Vipr2	28.9293154202902	-0.304872482754433	0.468908528400606	-0.650174744729679	0.51557935258712	0.862663759974268
N4bp3	58.9665023731262	-0.235459466181283	0.362267211056509	-0.649960744431144	0.515717578862638	0.862768030814357
RGD1308430	469.65211911318	-0.0846183732913558	0.130224665590125	-0.649787602893046	0.515829427871338	0.862828149696192
Ap1ar	484.211364283079	0.0939483246121528	0.144698639115937	0.649268888678895	0.516164591298195	0.862886199390351
Cdc40	418.045532764542	0.0925141425387864	0.142413876799646	0.649614662684448	0.515941159384041	0.862886199390351
Chp2	10.916341975266	-0.540222288116229	0.832097679595775	-0.649229413040383	0.516190102819155	0.862886199390351
Mapk14	2598.31902006155	0.0617941797154668	0.0951970806940241	0.649118431625876	0.516261829156473	0.862886199390351
Psip1	354.182934617143	-0.121846694001127	0.187736906568175	-0.649028985448206	0.516319641215359	0.862886199390351
Wdr1	3272.7195785427	0.0656634938665368	0.101143061074403	0.649214025846351	0.51620004712225	0.862886199390351
Atg4d	954.894832541947	0.0677524416746451	0.104412977096669	0.648889089829493	0.516410067166335	0.862910441548451
Pnrc1	1373.87193470278	0.147800598761336	0.22786809523395	0.648623488117503	0.516581769807073	0.863070468267998
Inpp5k	512.662226066022	0.0816448906487606	0.125958583986025	0.648188381173126	0.516863115845378	0.863160458747745
Pcdhga11	22.8746801232998	-0.426798781750205	0.658286314660968	-0.648348252492559	0.516759731662562	0.863160458747745
Snx18	863.903158414429	0.0904496698190755	0.139542371674225	0.648187849567577	0.516863459637265	0.863160458747745
Fastk	1136.59642474013	0.092516064129827	0.143007647964311	0.646930884094504	0.517676676471557	0.863503537536795
Nans	1380.26373621492	0.0701562991615781	0.108348222445995	0.64750761551761	0.517303467336035	0.863503537536795
Pou3f3	1610.4675945087	-0.113018934725606	0.174570355688823	-0.647411951929949	0.51736536263946	0.863503537536795
Rbm48	164.120764200759	-0.156135904294592	0.241138537357047	-0.647494614531091	0.517311878879976	0.863503537536795
Smad7	645.229366560906	0.0909649827454365	0.140557583569791	0.64717235765703	0.517520399367262	0.863503537536795
Tex26	13.5593986399911	0.396721673514109	0.613145839549161	0.647026609209016	0.517614722073981	0.863503537536795
Tor1aip2	719.235968866741	0.0710029389845333	0.10974471108063	0.646982786554213	0.517643084125128	0.863503537536795
Trip4	241.409234539838	0.122565312628861	0.189437983517799	0.646994390210797	0.517635574152644	0.863503537536795
Rpl36al	3620.29191355826	-0.102902598251795	0.159124818249997	-0.646678496688851	0.517840042902955	0.86352258327978
Tmed10	2393.91993744963	0.0551107843898788	0.0852067062131357	0.646789282665438	0.517768329584258	0.86352258327978
Abracl	413.222288818818	0.0768853185171716	0.119304464854705	0.644446279615826	0.519286082377497	0.863864617913875
Dcakd	391.458655658234	-0.17692590376352	0.274429425515237	-0.644704566324637	0.519118656597234	0.863864617913875
Dimt1	98.7720687570621	0.171661675730665	0.266048728329451	0.645226447081885	0.518780449806367	0.863864617913875
Dnpep	2878.64949904639	0.0698148837359277	0.108355346498257	0.644314157004246	0.519371737247381	0.863864617913875
Efcab7	183.142597954251	-0.144569209577137	0.224248136648072	-0.64468410635679	0.519131918075249	0.863864617913875
Eif4enif1	622.110550375042	-0.0677068671785576	0.10484762165599	-0.64576445425445	0.518431911429636	0.863864617913875

Fam222a	79.883028005913	0.20663310510116	0.320656459066129	0.644406495671262	0.519311873475423	0.863864617913875
Fdps	967.880260321608	0.0717427019434045	0.11112933410579	0.645578438138651	0.518552404970938	0.863864617913875
Gtf2h1	547.814354707349	0.0937079407459876	0.145266058751313	0.645078014448027	0.518876630549053	0.863864617913875
Mark3	1635.71393418898	0.0701807967658231	0.108784159070447	0.645138017938577	0.51883774863503	0.863864617913875
Mettl6	249.067581405354	-0.106774642210333	0.165384282961281	-0.645615413378369	0.518528452788514	0.863864617913875
Nr1d2	363.649975834003	-0.175876813690908	0.273003054220722	-0.644230205383389	0.51942616673268	0.863864617913875
Rdh7	97.3698865656539	-0.400309340738435	0.621470073195188	-0.64413293254864	0.51948923662162	0.863864617913875
RGD1309748	1001.17498931231	-0.101764255756461	0.157704809389856	-0.645283147357244	0.518743711835903	0.863864617913875
RGD1563159	29.84146727535	0.265057697007992	0.411019256801452	0.644879023602614	0.519005586234555	0.863864617913875
Rras2	975.855183957215	-0.093898012554416	0.145417553489562	-0.645713053900029	0.518465205004982	0.863864617913875
Slc17a9	19.7407623112058	-0.286574861789703	0.443630504985818	-0.645976456913991	0.518294602528135	0.863864617913875
Spryd4	349.140383796551	0.114057929646456	0.177049413846528	0.644215234427862	0.519435873363259	0.863864617913875
Tsx	86.2238893029807	-0.261836477232024	0.405231751848174	-0.646140081663997	0.518188639663465	0.863864617913875
Pllp	3444.03391482711	0.115259103809203	0.179022480487237	0.643824750363795	0.519689082285542	0.864070525052292
Mapre2	594.963970356346	0.134699805264174	0.209421413118149	0.643199772452019	0.52009448094629	0.864491645281593
Rab4b	381.37738615577	0.0885848092516662	0.13770369022983	0.643300183922569	0.520029336970722	0.864491645281593
Clcn7	382.953815119654	0.102432423356875	0.159292381630592	0.643046593367042	0.520193867085516	0.864530412822632
Arl8a	977.806656099471	-0.0713848487226807	0.111088026423769	-0.642597145891923	0.520485535520266	0.864721208847786
Nek7	103.64852867128	0.209661639186497	0.326259484602278	0.642622357606192	0.520469172175808	0.864721208847786
Tfb2m	1278.43658505357	0.0998069531360347	0.155337210708786	0.642517994758801	0.520536909285285	0.864721208847786
Arfip2	1361.05504960305	-0.0796645203577964	0.124087757520632	-0.642001450824435	0.520872240992159	0.865151818079333
Cct4	3302.74161737791	-0.0933774985969016	0.145503156789191	-0.641755826179012	0.521031735436778	0.865239457868337
Mark2	431.476537190118	0.100144283539895	0.15607586194859	0.641638510206539	0.521107922519733	0.865239457868337
Swi5	1585.55692853931	-0.0948247560544637	0.147801448718342	-0.641568515577725	0.521153381010614	0.865239457868337
Rarres1	1740.33613871005	-0.268312248056992	0.41831847697442	-0.641406638304909	0.521258521133475	0.865287622492052
Cpne1	119.025047060048	-0.135172192125472	0.210861157767017	-0.641048325622994	0.521491285891364	0.86538942396111
Fcgbp	67.6458890812917	-0.238953221612031	0.372879478029455	-0.640832321678899	0.521631630805366	0.86538942396111
LOC498972	60.1180855632478	-0.283566782152997	0.442570865233986	-0.640726275560585	0.52170053959014	0.86538942396111
Nek9	1983.4644923765	0.060884717313037	0.0950146791912885	0.640792747302348	0.521657345693967	0.86538942396111
Polr2b	1222.71193919537	-0.0775131912544184	0.120896009022565	-0.641155914749431	0.521421388923506	0.86538942396111
Brcc3	1954.84422864979	0.0890480227891066	0.139016639691951	0.640556576438831	0.521810819848094	0.865446049670718
Fam160b2	846.474076605592	-0.0628801539498092	0.098187658774345	-0.640407916175295	0.521907437695985	0.865480002458793
Eps8l3	10.4580296596769	1.70928918722621	2.67097626165226	0.639949224471523	0.522205610338567	0.865721844677385
Phf3	1302.30836702518	0.0983780772278742	0.153705365502396	0.640043221043841	0.522144500713625	0.865721844677385
Car4	483.606163628549	-0.118824735560776	0.185775085732389	-0.639616098640612	0.522422213733204	0.865828358310236
Necap1	1096.22566636959	0.0920386207465999	0.143883069351822	0.639676517614091	0.522382924986535	0.865828358310236
Prpf19	2267.58613315801	0.0524068665428093	0.0819744384212739	0.639307417679225	0.522622963909503	0.865908543410409
Rnf40	967.326336850381	-0.0777554698864354	0.121602651969508	-0.639422484847885	0.522548125525406	0.865908543410409
Dtd2	126.286976561132	0.161686620750561	0.253023178943283	0.639019007767679	0.522810566643729	0.865967400263442
Larp1b	2436.12381561662	0.0980716505502488	0.153495131711027	0.638923524525066	0.522872683492551	0.865967400263442
Optc	11.2701974256112	-0.47785000699486	0.748180788562458	-0.638682540770652	0.523029472893683	0.865967400263442
Prkci	1251.23341782715	-0.0775368011243732	0.121470872789968	-0.638315995789707	0.523268001496509	0.865967400263442
Prpf8	3115.53811762569	0.0897594952938788	0.140615204779144	0.638334207419878	0.523256148987332	0.865967400263442
Raver1	1176.21637797407	-0.0656450588885179	0.102775337539754	-0.638723846206063	0.52300259694295	0.865967400263442
Taf6	606.55100990822	0.074184953427785	0.116199287528474	0.638428642771207	0.523194690698604	0.865967400263442
Zmynd19	249.601391445937	0.11164392314308	0.174876044930201	0.638417475576148	0.523201958087851	0.865967400263442
Abcg3	120.520125139883	-0.209813738425703	0.328771452062961	-0.638175051724147	0.523359735496626	0.865967524829615
Rpusd4	319.088632386999	-0.0952676540904453	0.149303207132893	-0.638081766091258	0.523420455356278	0.865967524829615
Afg3l2	1855.61296682825	0.049302247272421	0.0773273883579218	0.637578073168823	0.523748372819237	0.866093061567651
Brk1	1908.8241837102	0.0498024224617352	0.0781218093570488	0.637497043035928	0.523801135422843	0.866093061567651
Sema7a	301.775221757705	-0.235630036433576	0.369478901734739	-0.637736107061241	0.523645477219084	0.866093061567651
Wdr78	79.0450296471267	-0.173126204868815	0.271489004147619	-0.637691406369737	0.523674580712073	0.866093061567651
LOC497978	56.6289096730567	0.206471532328358	0.323998250950051	0.637261255957176	0.523954683196093	0.866220935157351
Rab3il1	47.4205672508417	-0.239784280151591	0.376400776542379	-0.63704512608675	0.524095450194615	0.866327644984293
Scaf1	1721.6072847086	-0.0703327200782072	0.110451808221825	-0.63677291671817	0.524272769767718	0.86641980924575
Wif1	15.6309012836697	0.335157348662114	0.526376518040975	0.636725494346661	0.524303664261153	0.86641980924575
Arhgap31	73.4055103723921	-0.337667550260167	0.530770250055635	-0.636184017896205	0.524656488804077	0.866732772123724
Gabpb1l	104.112574154679	-0.160334551080925	0.252065148165426	-0.636083775356759	0.524721819899995	0.866732772123724
Tmprss9	180.912265266678	-0.144245578027905	0.226757118113149	-0.63612370464123	0.524695796277979	0.866732772123724
Cnp	245.12258560411	-0.182172623398036	0.286722440469244	-0.635362279631466	0.525192163364817	0.866821745698535
Med19	938.8001230586	-0.0907731352081074	0.142795928238027	-0.635684338679444	0.524982186236064	0.866821745698535
Nkap	120.118499726508	0.176993209746633	0.278508745536083	0.635503238528296	0.525100255225988	0.866821745698535
Paqr7	606.62459150492	0.153068958194965	0.240979320212945	0.635195410376721	0.525300976237364	0.866821745698535
Rpl29	8167.1002332294	0.0896285559183551	0.141107810387877	0.635177852111687	0.52531242638244	0.866821745698535
Sfxn4	143.959827055668	-0.151592530963222	0.238747793328208	-0.634948406642766	0.525462064742469	0.866821745698535
Slc16a7	722.239830480746	-0.281467472002755	0.442809066299427	-0.635640716110421	0.525010624907167	0.866821745698535
Stxbp6	86.7649914536771	-0.207891666079459	0.327194381680981	-0.635376637616464	0.525182801282775	0.866821745698535
Wdpcp	185.712533521067	0.123552896373288	0.194558138055595	0.635043579302669	0.525399992965639	0.866821745698535
Dek	2621.05982760012	-0.0941682867649325	0.148442991872502	-0.634373408788568	0.525837159137433	0.866937213628672
LOC100910318	199.202700878145	-0.116033094063864	0.182858172545982	-0.634552409926805	0.525720374770163	0.866937213628672
Pdzd3	1102.80944319217	-0.0994727540204923	0.15678238990696	-0.63446382007267	0.525778171125143	0.866937213628672
Ywhag	1016.90219921801	0.0872502737959663	0.137481086457175	0.634634741726052	0.525666664106561	0.866937213628672
Adss	2807.07830183471	-0.0728982859490945	0.114988148797847	-0.633963471116072	0.526104661652361	0.867082464435237
Esd	586.153416192764	0.112844985933939	0.17800211491013	0.633953062810023	0.526111454438935	0.867082464435237
Med12	1065.45746483113	-0.0659879218752864	0.104100337264846	-0.633887685756518	0.526154122576969	0.867082464435237
Cd81	2746.98341611985	0.0950725273605393	0.150254800593121	0.632742028775433	0.526902119073686	0.867210976800979
Cr1l	376.747061858601	-0.0990773845474576	0.156560710686541	-0.632836834433041	0.526840200109453	0.867210976800979
Emcn	848.56941921725	-0.425455385738157	0.672319823398994	-0.632816958433884	0.526853181108003	0.867210976800979
Gtse1	31.6781371213649	0.314060207016292	0.496169721557386	0.632969311449546	0.526753683650989	0.867210976800979
LOC690435	19.5697361322667	-0.29472315943927	0.465742854197045	-0.632802321674654	0.526862740467696	0.867210976800979
Mip	401.720124210933	-0.103595787939281	0.163554979134611	-0.633400392255974	0.526472208921441	0.867210976800979
Nudt14	673.240352366819	-0.102110920953267	0.161401455281019	-0.632651798433167	0.526961053282341	0.867210976800979
Rtp3	46.7732730092724	-0.349673603471282	0.552756523787342	-0.632599686160935	0.526995092096108	0.867210976800979
Trappc13	865.174185666625	0.0934372689763374	0.14758166063209	0.633122493514078	0.526653654438557	0.867210976800979
Txlna	375.88796576181	-0.0881089773533836	0.139221088955904	-0.632870910679997	0.52681794532078	0.867210976800979
March8	1700.61706941574	0.0728228214267096	0.115154868304853	0.632390297507213	0.527131872370421	0.867283719502733
Znrd1as	45.4546699363013	-0.20413670260149	0.322848676837816	-0.632298402461902	0.527191907271193	0.867283719502733
Trim17	18.4085724815079	-0.319545218416285	0.505773040296853	-0.631795673072519	0.527520401393128	0.867698535779203
Adora1	49.6294921796714	-0.325823010541511	0.515810933980998	-0.631671391738109	0.527601625561309	0.86770656578351
App	13130.3944547264	0.151936923469878	0.241264243279299	0.629753176039388	0.528856085300093	0.867760685075914
Ccng1	2160.401517897	0.0928347293394517	0.147379850762934	0.629901094748562	0.528759296586043	0.867760685075914
Cyp2r1	25.1615404123448	0.278046942677897	0.441132872656405	0.630302024429873	0.528496998731033	0.867760685075914
Dirc2	817.929811381965	0.102906234508123	0.163143660084789	0.630770662216609	0.52819048861432	0.867760685075914
Dnm2	1597.05538962126	0.0655502034486539	0.104076484551349	0.629827224960823	0.528807631209006	0.867760685075914
Fam204a	180.117275580761	-0.124093397252142	0.19697510610286	-0.629995331427012	0.528697638724207	0.867760685075914
Irf6	1420.81465121747	-0.0842733680310928	0.133611026363255	-0.630736626496487	0.528212746450453	0.867760685075914
l7Rn6	599.040327848085	0.0928698770396548	0.147278980903598	0.630571154620111	0.528320964452593	0.867760685075914
LOC100233176	480.702008960171	-0.124473075314016	0.197448292299687	-0.630408467271475	0.528427372392595	0.867760685075914
Nr2c2	81.8126170498044	-0.23248970296362	0.3688628682021	-0.630287629917357	0.52850641482097	0.867760685075914
Nupl2	200.661374427374	0.120731599082044	0.19171052105342	0.629759902683706	0.528851683610781	0.867760685075914
Orc3	159.636416175585	0.139719985420562	0.221624265992331	0.630436314340223	0.528409157852866	0.867760685075914
Rit1	129.067226283344	-0.16603940791663	0.263168458901251	-0.630924422363749	0.528089942322345	0.867760685075914
Rps26	4583.99831441496	-0.0948168614254537	0.150369051197254	-0.630561014188172	0.528327596625232	0.867760685075914
Suclg1	6604.064481546	0.107427176424163	0.170222295577797	0.631099328437064	0.527975580219061	0.867760685075914
Tpt1	34562.9849467706	-0.11305468585482	0.179295000632691	-0.630551244908538	0.528333986092287	0.867760685075914

B4galt2	24.9043499197202	-0.340304752577014	0.540586447712759	-0.629510329045162	0.52901500867474	0.867896158862168
Ano6	2158.79254863181	0.0758422444395311	0.120541064398672	0.62918180470594	0.529230039488114	0.867897683044903
Map2k6	99.44185066078	-0.261995309144223	0.416498724815713	-0.629042284007345	0.529321374174491	0.867897683044903
Ncstn	1530.95500718873	-0.0753713960738907	0.119784700497508	-0.629223897215978	0.529202485966616	0.867897683044903
Tor1b	629.348837970502	0.0695933653243318	0.110617577127486	0.629134782477889	0.529260820838365	0.867897683044903
Smtnl2	363.443257580057	0.130741660549992	0.207901627420805	0.628863093434874	0.529438689780974	0.86796482735476
Map1lc3a	1747.4459620688	0.0738166783071165	0.117481027101347	0.628328506554824	0.529788760980864	0.868413478123521
Erlec1	1802.88171745031	0.0758233043358471	0.120754971795185	0.627910414027941	0.530062628471599	0.868437517650987
Myo9a	458.393212440818	-0.192023710786277	0.305846467428272	-0.627843481080292	0.530106478933548	0.868437517650987
Nat1	70.5802025963252	-0.174778920374886	0.278263733329257	-0.628105280856266	0.529934973880191	0.868437517650987
Ppid	1019.7337283694	-0.109850667009979	0.174966146464117	-0.627839551993035	0.530109053093661	0.868437517650987
Zfp384	500.588948325652	-0.0897218947568497	0.142948945096611	-0.627649925616534	0.5302332952599	0.868515871728494
Lct	52.4702689301436	0.249534355152674	0.397962343287487	0.627030067948943	0.53063952578129	0.868831581273963
Ldlrad4	193.942589542192	0.141600107433587	0.225752659661228	0.627235611071324	0.530504803336128	0.868831581273963
Taf3	218.402145054945	-0.109732631861141	0.175010522492972	-0.627005909690646	0.530655361358777	0.868831581273963
RGD1564450	111.209302519055	0.150310695788884	0.239784524446668	0.626857367612629	0.530752734973587	0.868865848438685
Slco2a1	640.515833077966	0.325596790104632	0.519576144406212	0.626658466925449	0.530883134307791	0.868954162520501
Cwc27	309.943208904205	0.102781550806885	0.164174053157049	0.626052344023962	0.531280608840868	0.869199826358273
Kpna2	678.00645256431	0.137517065015677	0.219621828661893	0.626153902157805	0.531213999989745	0.869199826358273
Szrd1	1297.04290554883	0.0565469461993328	0.0903359035922587	0.625963143675009	0.531339116095133	0.869199826358273
Trappc8	808.337289408634	0.0810093196789804	0.129405476816485	0.626011523406097	0.531307383019786	0.869199826358273
Ppapdc2	733.237767710101	-0.0710052671296185	0.113491175046536	-0.625645712986084	0.531547348033227	0.869415334256102
Afap1	20.7790051887866	-0.393898981736925	0.629947743221574	-0.625288344907011	0.531781828063712	0.869673706154266
Dph1	359.457041075909	0.0987844181634209	0.158026129375005	0.625114457679335	0.531895939698617	0.869735182076605
Commd10	656.428958924702	0.0936994609013647	0.150209162849275	0.623793243527934	0.532763377405571	0.870406227633689
Hdac10	250.367006402123	-0.102212917984641	0.163758769880148	-0.624167597615989	0.532517524116979	0.870406227633689
LOC498063	14.1409149644195	0.380374783526083	0.609449730976005	0.624128232720574	0.532543373914733	0.870406227633689
Mdh2	8699.15517106437	0.0731057439839095	0.117218075006104	0.623672961530057	0.532842383589232	0.870406227633689
RGD1562608	37.7421052616634	-0.223059092642958	0.357531979746177	-0.623885708912848	0.532702646351986	0.870406227633689
Tctn2	210.909878326377	-0.120340610117785	0.192938953235196	-0.623723763915563	0.532809013761322	0.870406227633689
Zfand6	1721.80890578505	0.0705945060858104	0.113177942316122	0.623747919790148	0.532793147212399	0.870406227633689
Arpc2	6361.59811109413	-0.0688893822953433	0.110491893535915	-0.623479063402523	0.532969756365097	0.870444967040874
Cgref1	1721.95960702568	0.145559816854939	0.233511009679187	0.623353121786073	0.53305249637434	0.870444967040874
Lefty2	37.0355035371174	0.252914746804027	0.405927469202204	0.623054033029834	0.533249015089355	0.870444967040874
Ppp3cc	150.567021310535	0.138709798091693	0.222601870603245	0.623129525892091	0.533199408430465	0.870444967040874
Rrp15	326.510557160457	0.082525008602909	0.132445216855177	0.623087874084167	0.533226777709917	0.870444967040874
Cxxc5	1413.16138906952	0.0818169856612786	0.131445057689755	0.622442464549625	0.533650965161896	0.870476014642667
Fam73b	1334.97388806663	0.0728839223754853	0.1170821811192	0.622502260196906	0.533611658044894	0.870476014642667
Hist1h2aa	29.2623557115469	-0.409180652583118	0.657094769134312	-0.622711778884181	0.533473940910519	0.870476014642667
Ubb	19876.523946003	-0.0713578037853388	0.114560410884629	-0.622883623010061	0.533361000766493	0.870476014642667
Uhmk1	287.765185852866	0.171717620594274	0.275786710479836	0.62264646579781	0.533516869431193	0.870476014642667
Col13a1	22.4789996607324	0.39851466294191	0.640436694399607	0.622254574771839	0.533774485436449	0.870552561554123
Hif3a	20.2050801892381	-0.475517612715493	0.764398333227202	-0.622080912589006	0.533888665217171	0.870613854929465
Igflr1	23.6195612151506	-0.308379520013578	0.495824548540829	-0.621952908384858	0.533972833590479	0.870626198047537
Top2b	848.52667024867	-0.0881426744066215	0.141746491852692	-0.62183319851205	0.534051554141366	0.870629656392824
Lrrc1	292.92504487068	0.108849284568846	0.175099356944573	0.621642971557582	0.53417665839012	0.870708719240227
Bhmt	1312.22897588913	0.440134324259912	0.709264943568551	0.62054994857838	0.534895780173602	0.871755870010491
Afmid	4415.71111060119	0.109871194374703	0.177330925549202	0.619582816896869	0.535532482426456	0.872036874479586
Apobec2	21.8904960286074	0.279559332156333	0.450783087018718	0.620163755488733	0.535149981050462	0.872036874479586
Col1a1	435.360993196913	-0.550133887953329	0.88766171383941	-0.619756242019081	0.535418281499096	0.872036874479586
Cxcr7	590.661386953209	0.173885146525255	0.280804431818318	0.619239323963945	0.535758709703585	0.872036874479586
Gcs1	1284.00975957935	0.0735535390244028	0.118763430766724	0.619328176607472	0.535700186005229	0.872036874479586
Gtf2e2	507.933269654115	0.0917644166763714	0.148178915477014	0.619281200574087	0.535731126846721	0.872036874479586
Mfsd12	88.3253394941947	0.193228745641518	0.311775663271379	0.619768533611701	0.535410187916783	0.872036874479586
Rbmx2	96.5694910991679	-0.150463321086168	0.242833374610636	-0.619615492835053	0.53551096429157	0.872036874479586
Usp49	61.5138827246638	0.192014065487926	0.30981602576228	0.619768022055957	0.535410524757056	0.872036874479586
Rnf13	1927.14382626782	0.078868507147047	0.127442757997761	0.618854365568834	0.536012303664624	0.872199834424068
Wdr45b	302.950420054876	0.0875445335550378	0.141460348972041	0.618862700334076	0.536006812441314	0.872199834424068
Rps20	5961.42418822373	-0.0978102568980883	0.158096611287056	-0.618673962090778	0.536131166476706	0.872268370766424
Aak1	95.4530390395891	-0.155799558518009	0.253618597440352	-0.614306522039069	0.539012793526007	0.872504864180495
Actg1	35085.037263439	0.154046987865535	0.252851579477342	0.609238780251872	0.542366178920025	0.872504864180495
Acyp2	371.800402310608	-0.125211662181732	0.204254305036676	-0.613018473021899	0.539864125147009	0.872504864180495
Akap13	901.942833119111	-0.0893965712721049	0.145284156196106	-0.615322232050112	0.538341938251987	0.872504864180495
Ankrd50	111.692460926601	-0.196803053797552	0.32127405492-0.612570641119956	0.540160275715011	0.872504864180495	
Ap3m1	910.275266281038	0.0798052456176772	0.130054540717932	0.613629060370621	0.539460475626912	0.872504864180495
Apol3	172.74019805692	-0.206119744597966	0.337990993019205	-0.609837980464333	0.54196913936674	0.872504864180495
Atf4	3363.04435217563	-0.111154739030514	0.181861779280155	-0.611204506359095	0.541064200438235	0.872504864180495
Atl2	1271.27746907016	0.0912860353732842	0.149802484093677	0.609375978813539	0.542275256179813	0.872504864180495
Atp1b1	65383.3143570203	0.127929855147967	0.209293632265256	0.611245806971473	0.541036862159307	0.872504864180495
Atp6v1h	1573.52400986223	0.0755988083012105	0.124293416237654	0.608228581927962	0.543035880509422	0.872504864180495
Bmp1	288.541582974467	-0.107718152216287	0.175506319901555	-0.6137565432214	0.539376217833252	0.872504864180495
Ccdc103	35.6550052627954	0.264683042637551	0.429479460721925	0.616288011055609	0.537704450104535	0.872504864180495
Ccdc43	396.412982161561	-0.0884143987563127	0.144610321810125	-0.611397565883311	0.540936413705355	0.872504864180495
Ccser1	65.6245272719004	0.262288890036453	0.431581883369835	0.607738415682501	0.543360980007578	0.872504864180495
Cdk5rap1	562.066091426894	0.0707172998830806	0.115978529250351	0.609744754828114	0.5420309026238	0.872504864180495
Chmp7	1317.66911806416	0.0517635994248485	0.0842020899407872	0.61475433045961	0.538716973772498	0.872504864180495
Clu	1242.15136486365	-0.271630930430188	0.440661612258522	-0.616416140806998	0.537619903142865	0.872504864180495
Cluap1	659.195035129771	0.0874543991266512	0.141787039240616	0.616801081361457	0.537365938648252	0.872504864180495
Coprs	648.15220492679	0.119261658310573	0.194733353258848	0.612435704078104	0.5402495253108	0.872504864180495
Cstf1	480.170606016065	-0.0708819412570781	0.115141911863627	-0.615605039987786	0.538155224089715	0.872504864180495
Cycs	48466.6528170055	-0.0951353356640685	0.155700781433806	-0.611013861253573	0.541190403857345	0.872504864180495
Cyfip1	1585.31115188719	-0.0703451576412501	0.115785725149863	-0.607546029963549	0.543488605025966	0.872504864180495
Dhrs1	700.403966875506	0.0875994975629593	0.142055924437528	0.61665501041094	0.537462301846867	0.872504864180495
Dnph1	133.088050623619	0.21467515357802	0.350854485630628	0.611863785045134	0.540627883907546	0.872504864180495
Eif2b4	779.491924089745	0.0733388867384596	0.120280500016397	0.609732140525371	0.542039260041305	0.872504864180495
Elp2	1106.69729740082	0.0842774555236243	0.137615763079576	0.612411351996727	0.540265632966719	0.872504864180495
Eya3	381.319990660348	0.08740161735706	0.143505513498654	0.609047103670201	0.542493217467746	0.872504864180495
Fam195a	976.526111775157	0.102810807937687	0.167798954035682	0.612702317058691	0.540073190178524	0.872504864180495
Fam89b	424.892037152577	-0.0893050782517515	0.144783655201581	-0.616817403369277	0.537355171537395	0.872504864180495
Fbxw8	928.813321823339	0.0541897918150594	0.0891396369839281	0.607920265872631	0.543240357775886	0.872504864180495
Frs3	318.855184653739	-0.129531292666944	0.213135531816718	-0.607741428952975	0.543358981180064	0.872504864180495
Gbas	310.689546772896	-0.146633619831741	0.239565128283007	-0.612082488309892	0.540483182994768	0.872504864180495
Gnaq	70.8470566656909	0.171401396676476	0.280283163618793	0.611529406416986	0.540849156689961	0.872504864180495
Gorasp1	759.517832129146	0.0994013827237223	0.161880976590557	0.614039924994623	0.539188944704113	0.872504864180495
Hcfc2	190.160095361389	-0.115528156544807	0.187643778798292	-0.615678053835155	0.538107024500273	0.872504864180495
Hip1r	266.74923573479	0.124616217007353	0.203285056667759	0.613012186188487	0.539868282058058	0.872504864180495
Hipk3	248.002430018008	0.168696158817396	0.275144020557249	0.613119480029899	0.539797340597256	0.872504864180495
Hist2h3c2	83.5714670800382	0.276452672146428	0.450067915384445	0.614246567454789	0.539052405369731	0.872504864180495
Hn1l	611.703729543088	-0.077623176512856	0.12722359706018	-0.610131911897903	0.54177442876768	0.872504864180495
Kdm2b	292.735034759818	-0.0988850601543557	0.160538504356026	-0.615958523788528	0.537921894336798	0.872504864180495
LOC378467	423.845136216024	0.0757539447037084	0.123097084609578	0.615399990535715	0.538290597661173	0.872504864180495
LOC680254	154.412375379652	0.170720870498511	0.277729689329819	0.614701549951221	0.538751836050681	0.872504864180495
LOC684274	412.419572399272	0.0879853135929978	0.143740642326798	0.612111593274788	0.540463927700915	0.872504864180495
LOC688276	42.0283462238316	-0.267652449323388	0.439757287333009	-0.608636757213546	0.542765234916458	0.872504864180495
Map2k1	881.322696487835	-0.0675341096289357	0.110924875980371	-0.608827452201855	0.542638815321827	0.872504864180495

Med20	470.979843098619	0.0946875297909636	0.154463503601211	0.613009077117823	0.539870337809589	0.872504864180495
Mnt	398.179471734662	-0.115961546832112	0.189740178047836	-0.611159681756368	0.541093872147088	0.872504864180495
Mthfd2	72.5405290475176	-0.251762161398457	0.413714401643931	-0.608540965453601	0.542828744772951	0.872504864180495
Mtmr2	1449.21965545868	0.0757039197042094	0.123830149062561	0.611352891661001	0.54096598237862	0.872504864180495
Ndfip2	1826.96541798232	0.116241802525188	0.188598954569058	0.616343832821327	0.537667615054098	0.872504864180495
Ndufaf6	314.148373100563	-0.17669178297278	0.288005714603175	-0.61350096200776	0.539545146872963	0.872504864180495
Ndufs2	8346.24870136734	0.0807891548802566	0.132462073169056	0.60990404987206	0.541925369607905	0.872504864180495
Nuf2	48.1835288276727	0.217558986121552	0.356355779802231	0.610510614538909	0.5415236141904	0.872504864180495
Nup210	351.197841071282	0.111072108084992	0.179920459819046	0.617340063474174	0.537010446565416	0.872504864180495
Pigu	554.242231508866	0.069676333958905	0.11407911853823	0.610772022537633	0.541350517622296	0.872504864180495
Pnpt1	880.966162822318	0.088800382372616	0.143817967848343	0.617449847895614	0.536938051436341	0.872504864180495
Polr2i	197.124312317123	-0.134470906679469	0.219068111833119	-0.613831495393112	0.539326682446515	0.872504864180495
Pros1	798.557911581518	-0.159921841376725	0.260657522137669	-0.613532423945396	0.539524350350838	0.872504864180495
Psph	348.475541881153	0.139150427332161	0.226166892129277	0.615255513404777	0.538385991668219	0.872504864180495
Ptprk	1506.00310938972	0.0793829024550338	0.13036015831997	0.6089506447222	0.542557153708032	0.872504864180495
Rabgap1l	114.105166835027	0.156953878428252	0.257055577411824	0.610583438836647	0.54147538933931	0.872504864180495
Rai14	442.720880101198	-0.11586684896998	0.188902615096129	-0.613368157508129	0.539632935835962	0.872504864180495
Rbm18	1118.28347082299	0.0598854010608915	0.0968687135563635	0.618211999130627	0.536435601445379	0.872504864180495
Reps1	437.128152499975	0.0974835237074996	0.159754081013029	0.610209911943026	0.541722764635033	0.872504864180495
RGD1306746	346.188173949459	-0.0895161684626524	0.144953993361974	-0.617548826261833	0.536872786349549	0.872504864180495
RGD1311267	73.5424757731942	-0.199005032658376	0.327037502740239	-0.608508293363658	0.542850407187924	0.872504864180495
RGD1562161	209.777624482845	0.100055328660268	0.162359000799807	0.61625982032027	0.537723052777304	0.872504864180495
Rnf8	229.741961119786	0.0986874264882849	0.160388179170413	0.61530361525851	0.53835423048497	0.872504864180495
Rps6kb2	669.500661484209	-0.0903434169667758	0.148575776796836	-0.608062895005503	0.543145760417576	0.872504864180495
Sema5a	1996.48380516848	0.101402949334296	0.16427193049023	0.617287135006473	0.53704535093217	0.872504864180495
Ska3	32.1323460740523	0.261253429685218	0.427770937047031	0.61073206957137	0.541376971494251	0.872504864180495
Slc16a5	371.761504008652	-0.457760484531096	0.748470336720293	-0.611594691296581	0.540805951309551	0.872504864180495
Smad5	154.175592264883	0.121136443570587	0.196162543701357	0.617530958178276	0.536884568043514	0.872504864180495
Smcr7l	287.695626731546	0.130180206503833	0.211941469866986	0.614227157080366	0.539065230068116	0.872504864180495
Smim14	1129.2644510902	-0.0817283669352565	0.134172644086945	-0.609128391941771	0.542439339779235	0.872504864180495
Smug1	18.2519802438356	0.329530984943448	0.5417858375976	0.608231079654394	0.543034224157122	0.872504864180495
Snrpe	1089.78606044596	-0.0898602865319627	0.145978184371051	-0.615573394881756	0.538176115061922	0.872504864180495
Spock1	62.3381319581564	0.296441571710132	0.483743731155855	0.612807055921564	0.540003924832324	0.872504864180495
Tbl1x	883.075514480513	-0.0936403997325942	0.153388464940254	-0.610478759071405	0.541544709818026	0.872504864180495
Tmem81	26.8086170433179	-0.264429875263513	0.430454134488792	-0.614304414981518	0.539014185628921	0.872504864180495
Tmem98	1354.80427938426	0.0751169626876843	0.121521125883065	0.618139127183254	0.536483632217388	0.872504864180495
Tpgs2	365.946633301635	0.078859945998805	0.128995048657917	0.611340875632634	0.540973935604339	0.872504864180495
Traf2	438.7323498502	-0.0784015615602422	0.128640682428902	-0.609461642148655	0.542218490159678	0.872504864180495
Tubb2b	331.702956884803	0.133262840145304	0.216502726809214	0.615524996425277	0.538208066786714	0.872504864180495
Ubd	194.692069063336	-0.525009106956298	0.862627275644565	-0.608616399897632	0.542778731490019	0.872504864180495
Ube2b	2800.91642390841	-0.0943656767592126	0.15499558198221	-0.608828171438097	0.542638338538155	0.872504864180495
Ubqln2	732.487615465618	0.0889220703490069	0.14570558511592	0.61028594256193	0.541672407338731	0.872504864180495
Uqcr11	5386.03907600133	0.0969228556979987	0.156999945824562	0.617343242948021	0.537008349856326	0.872504864180495
Vps28	1377.87115654606	-0.0626318154804223	0.102370485721809	-0.611815163704739	0.540660055936641	0.872504864180495
Vstm2b	13.543901509752	-0.55529715009859	0.913954460567152	-0.607576388164901	0.543468464982556	0.872504864180495
Wdr33	1260.58776288536	0.0882483465191897	0.144626817213057	0.610179690182814	0.541742782037157	0.872504864180495
Yap1	208.400388112744	0.157912356669927	0.25992077884623	0.607540333523504	0.54349238416311	0.872504864180495
Yipf2	792.261821056225	-0.061988182445579	0.101176843635293	-0.612671637287129	0.540093480003181	0.872504864180495
Agap3	802.830175476795	0.0854583285466318	0.140838036372496	0.606784436560923	0.54399397796142	0.872719507547978
Commd7	396.433094068261	-0.0960098997321364	0.158195489616621	-0.606906682136206	0.543912843336245	0.872719507547978
Foxi1	589.79894668256	0.12096260586278	0.199358144829214	0.606760290463209	0.54401000448508	0.872719507547978
Igf1	32.2152053781576	-0.402143853546361	0.662394304067856	-0.607106448646584	0.543780270871784	0.872719507547978
Wisp2	19.3158680413994	0.568433847258111	0.936361204507481	0.607066850401072	0.543806548458489	0.872719507547978
Steap3	225.060569447548	0.107880028683314	0.177894641627262	0.606426521319017	0.544231561604856	0.872951725825683
Usf2	1198.48981865236	-0.0578634576668084	0.095446117801857	-0.606242129061038	0.544353981061238	0.873024883412168
Asb6	355.116700860118	-0.0943469395502663	0.155745519046281	-0.605776269699487	0.544663329679721	0.87302826190096
Fhl1	399.600302018824	-0.18011242926016	0.297267137128366	-0.605894183259092	0.544585022264871	0.87302826190096
Klhl29	145.9281383287	-0.141438349194007	0.233478389568615	-0.605787753870218	0.544655702696727	0.87302826190096
Tle1	111.098291839615	-0.154202502893928	0.25441121649469	-0.60611519027561	0.544438264663445	0.87302826190096
Flt1	169.577145001259	-0.257417054182825	0.425089139520885	-0.605560176091437	0.544806853832404	0.87313518057799
Pdzd8	650.598493569673	0.0753158026228235	0.124516576939856	0.604865669084389	0.545268255770072	0.873258981975854
Proca1	111.475715882206	0.138332321624551	0.228681325862579	0.604913064513536	0.545236762027839	0.873258981975854
Tap2	232.120487821218	0.135060788164204	0.223290363561332	0.60486617519034	0.545267919463455	0.873258981975854
Tfb1m	257.169237710451	0.130064744423487	0.214957556212931	0.605071748650922	0.5451313247335	0.873258981975854
Upk1a	204.432002922568	0.256485662785339	0.423774971566424	0.605240233601517	0.545019386392643	0.873258981975854
Pip4k2b	69.8233761862912	-0.290491376491751	0.480401194216643	-0.604684959131785	0.545388343832877	0.873328249648419
Arl4a	259.200879713894	0.180329422337341	0.298388949061421	0.60434350167647	0.545615290037723	0.873568585232957
Rpp14	407.412947925145	-0.0935427306225493	0.154815434803315	-0.604220959889372	0.545696747604428	0.873575948348158
Ccdc97	753.519020263017	-0.0607603548555353	0.10061406124598	-0.603895261786414	0.545913279190667	0.873799511516846
Mta1	783.629666580321	-0.0733002496828677	0.121438274584225	-0.603600882290446	0.54610902609403	0.873837973754942
Phf19	235.99325484813	0.127098986154132	0.2106289636429	0.603425967425904	0.546225351767441	0.873837973754942
Tlk2	579.972072638937	0.0810947965442048	0.134358904634892	0.603568455433395	0.546130590377905	0.873837973754942
Tom1l1	717.470065591406	0.0684317837678892	0.113410940044743	0.603396671792787	0.546244835784697	0.873837973754942
Fgb	21.6954630348041	0.416204927917107	0.690243942018114	0.602982369827427	0.546520417705252	0.874155793363058
Lpcat1	147.159683549067	-0.157582196858665	0.26153069366599	-0.602538060255057	0.546816036349086	0.87432376033276
Med4	318.87774379337	0.125320524632898	0.208008570186326	0.602477698494064	0.546856203783705	0.87432376033276
Ttc25	155.821154832069	-0.140385673435497	0.232997789994664	-0.602519334791598	0.546828496959619	0.87432376033276
Hsd17b1	145.785797907911	-0.307254056384406	0.510116342136087	-0.602321531393788	0.546960131184724	0.874350356577548
Ptgis	21.8303317552975	-0.426021810429275	0.707454496308478	-0.602189699340765	0.547047871502777	0.874350356577548
Slk	459.533865953495	-0.124876865790236	0.207400155006727	-0.602105942428953	0.547103619213401	0.874350356577548
Rnf215	755.054454516199	-0.0953885756293665	0.158494324032059	-0.601842218716121	0.547279169276404	0.874507948544301
Heatr5b	570.482555404941	0.0694098961915732	0.115453708348292	0.601192436211598	0.547711821742484	0.875076267349602
Sec24b	588.887479689324	0.0658185685475895	0.109545447045796	0.600833446962646	0.547950924356415	0.87533523629445
Ankrd32	198.101471157796	0.11841074424256	0.197277615865766	0.600223921618811	0.548357013140838	0.875496460672808
Arpc1a	2360.26401051877	0.061240502336563	0.101993649144836	0.600434466753888	0.548216723222865	0.875496460672808
Rad51	30.1507597138653	0.24638187874627	0.410266835939509	0.600540568145258	0.548146032724014	0.875496460672808
Rbfox2	52.0293687324377	0.206141772997361	0.343443978669449	0.600219499541041	0.548359959838969	0.875496460672808
Dctpp1	145.048983615931	-0.131991343907941	0.21999841511504	-0.599964976288219	0.548529577270296	0.875644266187386
Far1	174.418391070854	-0.161288150956756	0.269106515921107	-0.599346880935577	0.548941591447175	0.875865965860597
LOC498330	14.1078466998854	0.498765584184959	0.832076275402422	0.599422912212872	0.548890901768216	0.875865965860597
Tmem87b	316.828347845194	0.166944580162162	0.278445970758831	0.599558254361515	0.548800675539056	0.875865965860597
Zfp36l2	898.446815577902	-0.0803353892029768	0.134049996891445	-0.599294226526788	0.548976697236573	0.875865965860597
Coil	148.339969703775	0.142542708912455	0.237966071765874	0.599004336436235	0.549169992825749	0.876051387853678
Mrpl12	2969.60900064475	0.0900022564010162	0.150353836108467	0.598602993648182	0.549437659277836	0.876063532183891
Pank4	606.287378453649	-0.0671394762800637	0.112144826565681	-0.598685452875007	0.549382659720045	0.876063532183891
Pias1	235.514717460984	0.102829249428953	0.171725288931174	0.598800852623202	0.549305693691439	0.876063532183891
Pwwp2b	233.309711100881	0.165618893368307	0.276709127855928	0.598530647151395	0.549485915972106	0.876063532183891
Ift46	1741.87226579187	-0.0583963192488414	0.0976326591500391	-0.598122797813994	0.549757999483027	0.876074540513822
Nvl	239.600895053787	-0.120158299883683	0.200926285777392	-0.598021803960523	0.549825384525182	0.876074540513822
RGD1309188	954.582490311508	-0.0737860664039591	0.12340547332021	-0.597915671150991	0.549896202763542	0.876074540513822
Rnaseh1	207.612020848884	-0.10592580108912	0.177106076282844	-0.598092416207978	0.549778270247061	0.876074540513822
Srsf3	2784.41380847393	-0.0830601183172046	0.138907972814009	-0.597950690911153	0.549872834961796	0.876074540513822
Vamp2	2718.48435111907	-0.0770727283388569	0.128921431545329	-0.597827121643137	0.549955291797125	0.876074540513822
RGD1564865	2504.63737569321	0.14191526648315	0.237442851535526	0.597681781386107	0.550052284044205	0.876106258470632
Atg14	206.402358169903	-0.106345751433466	0.178047300850972	-0.597289321012951	0.550314233016692	0.876266386704287

Manba	1567.9474870275	-0.139814268407846	0.234211632314861	-0.596956978720373	0.550536103999826	0.876266386704287
Mrvi1	86.8029662529913	-0.302255053648752	0.506442966093364	-0.596819531289591	0.550627876471038	0.876266386704287
Mst1r	16.7912695981441	0.355838506906876	0.596317652574869	0.59672643493008	0.550690040388789	0.876266386704287
RGD1308117	59.0757021595566	0.175134218508895	0.293521211182625	0.596666311791446	0.550730188690301	0.876266386704287
Rnf125	53.5756242356209	0.296813145674318	0.497118400011261	0.597067309654187	0.550462442429053	0.876266386704287
Tgs1	137.996374853345	-0.154518775515712	0.258995774774121	-0.59660732168497	0.550769581789146	0.876266386704287
Tmem43	386.005480073152	-0.0849656297010925	0.142336960369154	-0.596933006583338	0.550552109448146	0.876266386704287
Ywhab	3194.20992822517	0.0486016216286572	0.0814929670077299	0.596390380829393	0.55091446499888	0.876374221018512
Sept7	817.254251607569	-0.068915366945547	0.115633916697275	-0.595978834877354	0.551189366067636	0.876476304568475
Clp1	180.325239261598	-0.138399311848863	0.232221741945357	-0.595979130504623	0.551189168572766	0.876476304568475
Gja1	526.81553441786	0.17768809570776	0.298333747001536	0.59560172958524	0.551441320954528	0.876476304568475
Mtrf1l	190.737421760124	-0.109291075671127	0.183489219614069	-0.595626685322426	0.551424645560049	0.876476304568475
Ndufb3	3089.34905567535	-0.0912723348305233	0.153191766844104	-0.595804439826108	0.551305877378819	0.876476304568475
Opn3	81.8502200175219	-0.152798457855458	0.256412926757239	-0.595907779642176	0.551236835784562	0.876476304568475
Agpat4	426.667392464447	-0.0922439739983524	0.155109220464365	-0.594703356268526	0.552041777967189	0.876572624807917
Nr2c2ap	644.152584927562	0.0910080105850867	0.152986817356169	0.594874853649713	0.551927127325833	0.876572624807917
Pnoc	6.64382663638526	0.824398427457895	1.38559818749785	0.594976548682281	0.551859146967868	0.876572624807917
Slc25a2	38.7172244387831	-0.207760028753366	0.349323914397893	-0.594748942715442	0.552011301052352	0.876572624807917
Spint1	1323.46719208887	-0.0521220408648546	0.0875481917313103	-0.595352569072125	0.551607823308578	0.876572624807917
Taf8	422.011468443923	-0.0782650640214023	0.131580495945966	-0.594807486160733	0.551972162920997	0.876572624807917
Usp25	725.479686187897	-0.0649757919987498	0.109187085074769	-0.595086790294437	0.551785458100502	0.876572624807917
Hsp90ab1	18893.4435111294	0.0842622991743775	0.141760745179477	0.594397970098821	0.552245965837964	0.87665190610535
Ubap1	1681.27957884423	0.0614603558136123	0.103382703120459	0.5944936044282	0.552182018647124	0.87665190610535
Hist1h2ak	33.401079818196	0.342353184119281	0.576147161551462	0.594211352525603	0.552370760675733	0.876727561212174
Rab11fip2	64.5574001324707	0.16561489290038	0.278833751026251	0.593955689692629	0.552541749901663	0.876876505079908
Zc3h3	305.697147492933	0.0958025471120111	0.161575682932449	0.59292676579349	0.553230164417429	0.877846439867164
Zfp385d	23.1314368653187	-0.294345930077282	0.496621210260505	-0.592697057628451	0.553383910944795	0.877967829675954
Abcb10	883.043120571325	-0.091823253853506	0.155043060988639	-0.592243556519014	0.553687506323987	0.878146931506512
Katnbl1	197.444667335577	-0.122942875045169	0.207609876116406	-0.592182208982367	0.553728581568465	0.878146931506512
Odz4	27.8985089458355	-0.287337680912726	0.485070506505318	-0.592362712346387	0.553607729801586	0.878146931506512
Hnrpdl	1325.27395372642	0.0769155589380555	0.130006482098243	0.591628645715775	0.554099287336544	0.878489677760797
Ints6	733.772485100778	-0.136443921063545	0.230581842122175	-0.591737492457229	0.55402638613864	0.878489677760797
Amz2	812.972807366787	-0.0686306407318752	0.116095094516365	-0.591158834210701	0.554414002165777	0.878605221512395
N6amt2	251.532489366502	0.103708466125569	0.175490991459437	0.59096176540515	0.554546039595621	0.878605221512395
Oma1	498.240001420944	-0.0662962245149532	0.112213994456947	-0.590801751918642	0.554653261024257	0.878605221512395
Pitpnb	1570.47470766593	0.0560890754367347	0.0948451661201955	0.591375161551766	0.554269079096762	0.878605221512395
Tob1	3122.82608937701	0.0999301538370778	0.169149919879908	0.59077860579553	0.554668771558598	0.878605221512395
Uhrf1	15.1991953631816	0.400733857083161	0.678391031528995	0.590712197624377	0.554713273761477	0.878605221512395
Zfp579	284.537368947897	-0.0889991025067652	0.150590948337159	-0.590999017467533	0.55452107928358	0.878605221512395
Camkmt	144.823495460351	0.127216114155007	0.215988534414392	0.588994756133364	0.555864791313937	0.878890435766939
Cd48	568.240826562351	0.0951080597999381	0.161684896518906	0.588230947031078	0.556377287863675	0.878890435766939
Cnot6	342.385543212455	-0.111606972984734	0.189787847333058	-0.588061746592636	0.556490848237376	0.878890435766939
Dstyk	695.936636545176	-0.131693065626385	0.223910430533623	-0.58815065163572	0.556431177420881	0.878890435766939
Fam220a	149.873223589024	-0.12408622268763	0.211097889731339	-0.587813657661631	0.556657375752128	0.878890435766939
Fkbp4	2570.50789653446	0.0697340699948474	0.118376900336543	0.589085115394935	0.555804177783441	0.878890435766939
Fut4	102.789808577539	-0.227528517958833	0.386388040147501	-0.588860146582112	0.555955094184612	0.878890435766939
Hist1h2bh	3210.10943616035	0.101847640037282	0.173119657453119	0.588307772413783	0.556325729557968	0.878890435766939
Id1	1032.38081344961	-0.148309962575237	0.252357343088829	-0.587698224905755	0.556734867254531	0.878890435766939
Ldlrap1	343.719405250064	0.110065773517198	0.18698961559992	0.588619711121781	0.5561164082193	0.878890435766939
Lsm12	1460.93283119926	0.0588566438308797	0.100151363360646	0.587676910786889	0.556749176273268	0.878890435766939
Mad1l1	232.633811719783	0.0928791034436925	0.157645319728438	0.589164991410386	0.555750599163408	0.878890435766939
Mpnd	1857.85594721857	0.0718748274792635	0.122281400426066	0.587782174793793	0.556678510087186	0.878890435766939
Nebl	80.4547075950721	0.603646672354384	1.02569984438112	0.588521754840091	0.556182136032164	0.878890435766939
Pa2g4	1695.50996039401	0.0802242038384144	0.136471381417897	0.587846352875664	0.556635427987454	0.878890435766939
Pdss2	472.213791541248	0.0899255771514076	0.152787208126203	0.58856744785289	0.556151475946518	0.878890435766939
Pkia	19.850294650504	-0.270302179838444	0.458645455867226	-0.589348867149126	0.555627269991068	0.878890435766939
Rpo1-3	643.65730761252	0.069695824798952	0.118465239779757	0.588322996083289	0.556315513073167	0.878890435766939
Slc35a1	990.253029837308	-0.125841400035736	0.213224998279287	-0.590181268853413	0.555069128581319	0.878890435766939
Slc35g2	42.6228252046303	-0.20772241836405	0.353260038581918	-0.588015613647964	0.556521812732588	0.878890435766939
Slc50a1	575.90239405531	0.0710537857199111	0.120408276277651	0.590107158050059	0.555118810192426	0.878890435766939
Spata5l1	69.0310569136345	0.184509650119851	0.312957387423129	0.589567965271858	0.555480333903717	0.878890435766939
Tor2a	1188.72734988946	-0.0659284342690425	0.112170090289311	-0.587754133913943	0.556697334157482	0.878890435766939
Vnn1	321.860234528763	0.353290576610314	0.599406121865152	0.589401014976276	0.555592295822367	0.878890435766939
Dusp7	404.394862838494	-0.109335386362644	0.186089827175776	-0.587540909796041	0.556840483387173	0.8789125030105
Hecw2	21.3585801106093	-0.398451425643998	0.678577959262484	-0.587185923452417	0.557078845278928	0.879125073582345
Pcdhb20	59.9863980791621	-0.228367530896897	0.388968920500763	-0.587109969102144	0.557129852631852	0.879125073582345
Gchfr	2009.64592469124	0.100709172897208	0.17159748594735	0.586891890293241	0.557276316696595	0.879234122095155
Secisbp2	692.057205948395	-0.0592830636167093	0.10106710077541	-0.586571328967352	0.557491643148211	0.87932972745248
Taok2	860.229636481582	-0.0920260708782694	0.156877838335883	-0.586609758615088	0.557465827168923	0.87932972745248
Irx3	820.797968107795	-0.110983079833771	0.189476646415653	-0.58573487515875	0.558053693926	0.879378839048716
Krt72	19.4872258461233	-0.367069748894578	0.62611844043708	-0.586262478770525	0.557699141337145	0.879378839048716
Nedd8	2046.95580963304	0.0681806403315277	0.116270452920403	0.586396961730276	0.557608785567471	0.879378839048716
Piwil1	25.8574574895724	0.238942071984725	0.407738026288473	0.586018611410245	0.557863007948013	0.879378839048716
Prrc2a	3183.10493453891	-0.068229202536565	0.116487944972603	-0.585718999099966	0.558064364425338	0.879378839048716
Sorcs2	469.264838715353	0.343768922710961	0.586761357980841	0.585875191055417	0.55795939015635	0.879378839048716
Wbp1	1805.6752366773	-0.0554468545635741	0.094625308405953	-0.585962207126459	0.557900912127271	0.879378839048716
Rptor	662.160497232849	0.074400279540281	0.127110827455509	0.58531819066572	0.558333785697889	0.87968142614946
Trak2	2467.46337988772	0.0672992921581581	0.115042724354706	0.584993901488783	0.558551817402124	0.8799029738867
Acy1	36936.2984209717	0.0987140358161993	0.168826885699347	0.584705661111303	0.558745646879081	0.88008633902822
Peli3	103.209230879542	0.178466145839343	0.305407404954342	0.584354350759842	0.558981932422891	0.880302251393296
Zfp397	95.6189998823594	0.168405631416765	0.288231781083869	0.584271556673908	0.559037625422911	0.880302251393296
Eif3k	756.677626231147	-0.0995963205144584	0.170660811885717	-0.583592210853614	0.559494701897974	0.880477492021191
Hsd17b8	1539.18887282515	0.109617979758118	0.18787398359043	0.583465457341276	0.559580004081268	0.880477492021191
Msantd3	71.9548392422157	0.167576470277414	0.28723355000148	0.583415378449178	0.559613707756562	0.880477492021191
Pard3b	77.5978751592144	0.246915133200766	0.422981437628929	0.583749335632497	0.559388969313288	0.880477492021191
Sgcb	346.317285094045	0.0947920206854203	0.162445386892136	0.58353162560635	0.559535473581532	0.880477492021191
Slc16a13	1342.23297864367	0.0743427979212927	0.127396341496418	0.583555202983462	0.559519606686829	0.880477492021191
H3f3b	13014.0300781237	0.130050868650186	0.222957477013133	0.583298978766814	0.559692049897332	0.880478870468246
Adam9	1393.2534186076	0.12360386159695	0.212092094426558	0.582783917199472	0.560038772975871	0.88053682302445
Akr1c1	195.794668906363	0.594223148348744	1.01947013293538	0.582874504266043	0.559977785096081	0.88053682302445
Mmp15	412.731433937668	0.177470135200816	0.30440412397399	0.583008314355098	0.559887703142407	0.88053682302445
Slc6a17	1203.26651868432	-0.0753534933579042	0.129256903583099	-0.58297461310807	0.559910390412183	0.88053682302445
Bsnd	1461.29217900641	-0.279098423329558	0.47929588427545	-0.58230924254956	0.56035840103485	0.880677462729828
Fadd	54.7044691085862	0.183473412553199	0.315143087944046	0.582190819256603	0.560438156600814	0.880677462729828
Lyrm1	108.974620870781	0.158933879777406	0.272970605701364	0.582238074202332	0.560406330741786	0.880677462729828
Ppp1r15a	1002.09648739695	0.140446086082256	0.241210679508719	0.582254841984221	0.560395037974261	0.880677462729828
Ccno	131.182683920194	-0.616096283842716	1.0588363524873	-0.581861665776259	0.560659863341005	0.880782309154647
Socs6	317.182515140341	0.0913227315951061	0.156920259401143	0.581969032829937	0.560587539828605	0.880782309154647
Scg5	76.276404516225	0.153535221876482	0.264063854039981	0.581432178344391	0.560949215517515	0.881115088940308
Fmr1	818.103064533271	-0.0805845390718595	0.138700938451148	-0.580994908698776	0.561243884930296	0.881290131547529
Rab23	547.313569264623	0.162020435077366	0.278902391599626	0.580921641252729	0.561293266080641	0.881290131547529
Smyd2	666.70355656621	0.0803917780937841	0.138383990928297	0.580932646576429	0.561285848524792	0.881290131547529
Ndufs5	3176.62568623055	-0.100778785251733	0.17353718527599	-0.580733086637634	0.561420358703335	0.881367927765485
Wdr45	798.244126320242	-0.0574349958174184	0.0989580236458973	-0.580397563546123	0.561646547803792	0.881601251531267
Rin3	280.526213125061	-0.122015341795207	0.210279153461873	-0.580254104063296	0.561743272838149	0.881631322711737

Dnajb5	344.179435524937	-0.0951129963855883	0.164155235540777	-0.579408850849363	0.56231333339792	0.882404162280927
Fyn	135.219176840039	0.177237355310952	0.305985006552862	0.579235424989139	0.562430330804228	0.882465922131537
B4galnt4	22.7952555027851	-0.313625063587186	0.54234050319234	-0.578280732752058	0.563074600350647	0.882769422415298
Gfra4	21.9865629549026	-0.266297269949451	0.460219902991233	-0.578630494288996	0.562838524112748	0.882769422415298
Megf6	16.7938866982115	0.461200933952791	0.797381227214824	0.578394522233389	0.562997791373171	0.882769422415298
Mlf2	5304.92815866132	0.0823202351042364	0.142358943217876	0.578258262132839	0.563089768829044	0.882769422415298
St13	6226.21585307887	0.0926594232490601	0.160098102141299	0.578766531331402	0.562746716990249	0.882769422415298
Steap1	28.7371806003684	-0.314913621344655	0.544454910895234	-0.578401654650978	0.562992977092105	0.882769422415298
Gper	17.8336529318585	-0.558021501853862	0.965654271588082	-0.577868827666613	0.563352682442509	0.882850301600446
Tdg	807.998825960236	-0.0714862766550785	0.123669325478541	-0.578043717619232	0.563234604037398	0.882850301600446
Tubgcp6	373.453895622549	-0.091072202841265	0.157608896622772	-0.577836688110579	0.563374383029037	0.882850301600446
Col11a2	233.429593245006	-0.182362358673011	0.315836885511603	-0.577394113982647	0.563673249484784	0.883196878087132
Gata3	195.656516018499	-0.142722700351595	0.247297211188231	-0.577130246094693	0.563851473520526	0.883354355345871
Eif5a	9914.71605016367	0.103720326370416	0.179993800822408	0.576243881158732	0.564450348821778	0.883855383846332
Nat9	805.546998536193	0.0790863617244558	0.137255874844381	0.576196551252344	0.564482336032072	0.883855383846332
Pcsk7	762.622893840383	-0.0617797382612156	0.107214023172243	-0.576228150322875	0.564460980175441	0.883855383846332
Sumo1	2369.88403735919	-0.0791879091685272	0.13735997959131	-0.576499133183745	0.564277855593583	0.883855383846332
Eif3s6ip	2652.78862261238	0.0717470248606967	0.124550178888622	0.576049151441653	0.564581959581059	0.883889607795912
Fam108b1	201.847580951826	0.12302226816521	0.213854890481505	0.575260485688304	0.565115141135042	0.884060690806623
Hmgcs2	128.631797245337	-0.271470257329672	0.471642090455791	-0.575585306789149	0.56489551483596	0.884060690806623
Mavs	1080.90472532883	-0.0899776834286676	0.156510005500931	-0.574900519239535	0.565358578743314	0.884060690806623
Pcdhga7	62.2269970848074	-0.187190316902253	0.325487042184099	-0.575108353457513	0.565218018759575	0.884060690806623
Pqlc3	145.601889638594	-0.144598265768855	0.251460331492553	-0.575034101444895	0.565268234075193	0.884060690806623
Snap91	19.7423832553419	0.493987622621457	0.858390247797236	0.575481401249731	0.564965765624846	0.884060690806623
Snx14	453.268955483624	-0.0940346218147129	0.163573514101056	-0.574876821174228	0.565374607005778	0.884060690806623
Snx15	421.674312745941	0.0860443649759243	0.149680851813533	0.574852186725363	0.565391268825738	0.884060690806623
Zfp280d	490.908144726836	-0.079666851307402	0.138486129884955	-0.575269533299715	0.565109023078289	0.884060690806623
Ddx27	726.019579617337	0.0613468057016532	0.106742470777904	0.574717872413649	0.565482118153451	0.884081121723814
Fkbp1a	1650.51981511347	0.0518428570506004	0.0902620808564947	0.574359205534205	0.565724752465867	0.88423854097758
Nip7	560.05881171565	0.108861731627404	0.189542631841704	0.574339031644976	0.565738401371543	0.88423854097758
Atpaf1	583.907044327649	0.0666422583267878	0.116184849172337	0.573588198474463	0.566246499705618	0.884731396385001
Clcc1	563.493302751099	0.058465590696788	0.101997860726986	0.573204087615925	0.566506517034887	0.884731396385001
Efr3a	1201.43681630768	-0.0471859216139715	0.0823462834082559	-0.573018230586479	0.566632350353785	0.884731396385001
Mpped2	154.510882331761	-0.140198748098949	0.244694983398923	-0.572953095120815	0.566676453077847	0.884731396385001
Ndufs6	2333.57845731947	-0.0798035679616091	0.139155953714312	-0.573482958015912	0.566317734752631	0.884731396385001
Papd5	927.802846505922	0.0715277241799005	0.124772003339308	0.573267417894912	0.566463642734454	0.884731396385001
Parn	216.453106026177	0.11546861703878	0.201438674736139	0.573219701678588	0.566495946243478	0.884731396385001
Prkag2	329.225127339554	-0.126883995707084	0.221386223985271	-0.573134106643986	0.566553895633275	0.884731396385001
Acsl1	2905.25531474182	-0.0969881910664223	0.16948086482842	-0.572266321419893	0.56714156242413	0.884825919357343
Caskin1	105.310378580748	-0.143273699217525	0.250402315179325	-0.572174019696742	0.567204086612493	0.884825919357343
Eif4a2	9007.33290497885	-0.0540680998281707	0.094467793786043	-0.57234426317426	0.567088768094993	0.884825919357343
Hnrnpa2b1	7893.75198676544	-0.0780734029486514	0.136323228724246	-0.572707994663024	0.566842423465929	0.884825919357343
Mrps25	1891.52260119076	0.0857573336712165	0.149832365922215	0.572355199381537	0.56708136057579	0.884825919357343
Plekha4	45.9998971565952	-0.179866795014878	0.31415726739964	-0.572537431661796	0.566957934377487	0.884825919357343
Aars2	259.958415141663	0.0957711863369825	0.167489955672495	0.571802565428166	0.567455739094791	0.884967538412409
Brca2	62.2186501280072	-0.177862311744177	0.311401710860705	-0.571166777640916	0.567886596035514	0.884967538412409
Cactin	293.642338307478	-0.0897298511436962	0.157320076534898	-0.57036490904447	0.568430224777853	0.884967538412409
Cldn3	1291.9146245207	-0.263583982723055	0.462641095267907	-0.569737503691536	0.568855749171184	0.884967538412409
Ctps	242.872636889219	-0.10714494218509	0.18798389826442	-0.569968721652843	0.568698912777754	0.884967538412409
Dusp18	37.2674940991204	-0.21281789303226	0.373225524947564	-0.570212589458236	0.568533518321437	0.884967538412409
Ebp	574.889952314513	0.0869675635474897	0.152107467127858	0.571750783769129	0.567490824329499	0.884967538412409
Edn1	90.8319237322521	0.192468408439421	0.337228155262711	0.570736474507836	0.568178290145157	0.884967538412409
Efcab14	594.824430811575	0.0780894738199502	0.137043460594393	0.569815396380505	0.568802911830923	0.884967538412409
Ets2	875.646204252467	-0.065145353814021	0.114178482807498	-0.570557185663911	0.568299847706028	0.884967538412409
Fdx1l	603.005251166237	0.091092284214358	0.159546475962482	0.570945134731641	0.568036834495263	0.884967538412409
LOC497940	268.536396790697	0.117357611200577	0.205523882830411	0.571016903653067	0.567988184558042	0.884967538412409
Lppr3	14.5289913489665	-0.338601040586716	0.593953602811018	-0.570079950663168	0.568623472900813	0.884967538412409
Mmp24	1148.08065259893	0.100786111091647	0.176465986687278	0.57113618881271	0.567907329239862	0.884967538412409
Pef1	1417.52880672132	0.0615622931535248	0.107991031885479	0.570068570312481	0.568631191281299	0.884967538412409
Ppapdc1b	884.379281409493	-0.0925706313879232	0.162025076751962	-0.571335210842924	0.567772437968441	0.884967538412409
Rn45s	821031.8873888	-0.130662483773693	0.229428305127522	-0.569513354950113	0.569007810198652	0.884967538412409
Sesn3	159.682135248008	-0.134828732250545	0.23672549868149	-0.569557284709557	0.568978007010013	0.884967538412409
Trappc10	978.263320192446	0.0591530278281067	0.103484824583722	0.571610649832529	0.567585778834399	0.884967538412409
Trim24	170.608955209735	0.157980392467836	0.276400998794391	0.571562306782235	0.567618537763536	0.884967538412409
Ufc1	2794.88623893639	0.0819668083799636	0.143619786599405	0.570720861802918	0.568188875040758	0.884967538412409
Usp10	2288.94839484393	0.056185887473467	0.0986437372621005	0.569583929329226	0.568959930911332	0.884967538412409
Cct7	3726.62791016268	0.065958606908518	0.115841749957472	0.569385449829039	0.569094588888548	0.884981406116738
Arhgap11a	94.5834263632933	0.195912990765695	0.344322734960209	0.56898070000616	0.569369237666775	0.885166291248881
Mybbp1a	1265.28760424353	0.0824354393711014	0.144862623880123	0.569059410654597	0.569315822475521	0.885166291248881
Rab24	1260.5615037742	-0.0734429005127187	0.129143577456583	-0.568691854129636	0.569565276846652	0.885349963982364
Gpn3	400.199384229737	0.0821429593786985	0.144481199530781	0.568537357424131	0.5696701467047	0.885391889436021
Htt	521.61673199626	-0.0632281977336076	0.111237528539462	-0.568407070561419	0.569758590473872	0.885408277184308
Ctu2	224.663779668705	-0.109540221712053	0.192803157153904	-0.5681453733904	0.569936260441111	0.885515135039395
Dnajc27	190.780993217462	0.130114339773156	0.229191403827632	0.567710383549163	0.570231639737747	0.885515135039395
Eva1a	892.699901762137	0.0841840684318583	0.148311296434036	0.567617372755558	0.570294808066899	0.885515135039395
Fastkd2	307.883843236077	0.104112670939101	0.183335137705209	0.567881706923567	0.570115294108779	0.885515135039395
Nup85	282.782086255328	-0.0803222969483168	0.141431677226434	-0.567922961273519	0.570087279988539	0.885515135039395
Slco1a1	12981.6170245006	0.133542100211212	0.235252058901592	0.567655394111021	0.570268985440413	0.885515135039395
Gtpbp5	502.516788796451	0.0745282282467965	0.131355986343293	0.567375955382956	0.570458782363247	0.885527795730765
Hirip3	660.147715121238	-0.0739518184568434	0.130333423008061	-0.567404866304092	0.570439144448453	0.885527795730765
Mcoln3	536.275488010015	-0.119059825425678	0.209898033144179	-0.567226970363728	0.570559986428915	0.885563949986488
Inpp5e	344.721364198455	0.0800921890972084	0.141272782472525	0.566932905938801	0.570759766614994	0.885632151173518
Sufu	145.479126805437	0.111226543176966	0.196181759707586	0.566956598527569	0.570743669215122	0.885632151173518
Hoxb9	102.283094165691	-0.147699677964453	0.260713238796472	-0.566521587650393	0.571039262087964	0.885633315989801
Mfsd6	506.405420916208	-0.0837365748798112	0.147808477367371	-0.566520786704864	0.571039806403147	0.885633315989801
Pqbp1	585.744112446441	-0.0767135691576701	0.135413984218768	-0.566511424948074	0.571046168584906	0.885633315989801
Slc25a42	1266.52225862395	-0.0802173045387408	0.141608313972338	-0.566473127802442	0.571072195397611	0.885633315989801
LOC500797	454.763333428378	0.0885711167079421	0.156422160228225	0.566231258913149	0.571236582995608	0.885767394587733
Atp5j	4271.9932697961	-0.0831901465579568	0.147032785381457	-0.565793175597748	0.571534386187067	0.885769428956355
Clptm1l	2412.99242284392	0.0512514995554087	0.0905754842270177	0.565842954004555	0.571500543772054	0.885769428956355
Ddah2	647.636684878413	0.069062438017677	0.122119774447349	0.565530343715569	0.571713091227655	0.885769428956355
Kcnn2	17.2041821962213	-0.302888696287737	0.535275564909305	-0.565855638000321	0.571491920565916	0.885769428956355
Pcbp2	4108.6950055448	-0.0713762392334542	0.126233014194741	-0.565432424225736	0.571779675583849	0.885769428956355
Rbm17	808.62855105676	0.0846560748593762	0.149720629129306	0.565426924477207	0.571783415471826	0.885769428956355
Ripk1	576.917874583668	-0.0601139214197232	0.106255684097424	-0.565747817920084	0.57156522394913	0.885769428956355
Fhit	250.027884709962	0.120683445605658	0.213773641975198	0.564538473923084	0.572387724011544	0.885981052855129
LOC688869	5064.68665112016	-0.0835525867500247	0.147942040378259	-0.564765678074989	0.572233154853149	0.885981052855129
Nop58	734.326025242396	0.0664077962384781	0.117569007221376	0.564841005363223	0.572181913355614	0.885981052855129
Slc25a26	179.039148359197	0.102309341947652	0.181092678099232	0.564955706776781	0.572103891739446	0.885981052855129
Tex30	147.484102244771	-0.152717549982121	0.270292588992015	-0.565008276962535	0.572068134402763	0.885981052855129
Vil1	2928.63592714917	0.0912187191435721	0.161570971997849	0.564573685580022	0.572363767886012	0.885981052855129
Acsm3	5185.86429745461	0.357953188807103	0.634263543874303	0.564360339269384	0.572508924587522	0.885990298195433
Spock2	43.5113237078097	-0.33090552767348	0.586399429291379	-0.564300562286282	0.572549598825044	0.885990298195433
Sept10	82.8498674759291	-0.176886511963922	0.315124998782057	-0.561321737874114	0.574578225257022	0.886096519105748
Ambra1	920.90680629746	0.0574848440176724	0.102519120193105	0.560723150075753	0.574986283026968	0.886096519105748

Arhgef9	87.4152335512431	0.144011671006867	0.255520076092652	0.563602176428001	0.573024905304358	0.886096519105748
Aste1	34.718827303705	0.195932517383048	0.34810202045603	0.5628594661024	0.573530583378658	0.886096519105748
Aven	291.700952617574	-0.10263193797931	0.183092899825769	-0.560545701537167	0.575107276162177	0.886096519105748
Cbx3	2469.05984208698	-0.0711516533037198	0.126652112407163	-0.561788129320579	0.574260380853857	0.886096519105748
Ccnd3	1181.23268985466	0.0712991297234886	0.126566378630506	0.563333884519499	0.573207548798474	0.886096519105748
Ddb2	91.4098159357013	-0.131759628580166	0.234384491781565	-0.562151649107229	0.574012700927124	0.886096519105748
Dync1i2	202.062646034374	-0.105849442225194	0.188813936330578	-0.560601851125394	0.575068989311395	0.886096519105748
Fam110c	458.229592000473	0.123694956400003	0.219649920202837	0.56314592004302	0.573335524708421	0.886096519105748
Gadd45g	3071.32870976749	0.270091977274891	0.480426070205998	0.562192591170335	0.573984808704991	0.886096519105748
Golph3l	224.010379801024	0.143378454612603	0.255439502680418	0.561301024736117	0.574592343130239	0.886096519105748
Gpx4	5323.97037688011	0.0932848205163016	0.166488258551433	0.560308704817662	0.575268890945039	0.886096519105748
Hdgfrp3	144.660555053884	-0.150090102760396	0.267662597269729	-0.560743653731893	0.574972303402256	0.886096519105748
Hspbap1	324.078552219516	0.211990566750864	0.378096524138445	0.560678433196177	0.575016772058832	0.886096519105748
Hspe1	7127.17083647898	0.11685047646392	0.207953095892791	0.561907847354974	0.574178806792946	0.886096519105748
Kcnj11	63.0546947964424	-0.26203021981517	0.465781428000134	-0.562560471636269	0.573734214973904	0.886096519105748
Kctd6	614.406320026698	-0.117379845724415	0.209412900309563	-0.560518695605182	0.57512569118924	0.886096519105748
Lrrc8a	441.607839533065	0.109451408019674	0.195290759083985	0.56045359510638	0.575170083598799	0.886096519105748
Med8	139.352955595536	0.108415084504612	0.192941876534305	0.561905411370538	0.574180466581269	0.886096519105748
Mlc1	916.375956055561	0.13662825170437	0.242852551791946	0.562597554344088	0.573708957759376	0.886096519105748
Nmnat3	227.774262657425	0.13069604334692	0.2324326870484	0.562296314716292	0.573914148793983	0.886096519105748
Nrarp	78.9002901334755	0.17003583360994	0.301661127614306	0.563665046785023	0.572982109417168	0.886096519105748
Nrip1	802.195228404525	0.125353744108079	0.223247453099898	0.561501340183203	0.574455816954237	0.886096519105748
Osr1	14.79355417107	-0.36740871590987	0.652245530947917	-0.56329817296856	0.573231862021636	0.886096519105748
Pkn3	197.231118186417	0.101220180885995	0.180140722830224	0.561895052355214	0.5741875248507	0.886096519105748
Polr3f	311.335606207039	-0.0886455073591153	0.157361689443149	-0.563323307425096	0.573214749870097	0.886096519105748
Rbfa	680.086321371557	0.0690904499015127	0.123273282622979	0.560465726485275	0.575161811018028	0.886096519105748
RGD1563348	856.475918397861	0.0834511126401487	0.148402173292031	0.562330798727122	0.573890658057724	0.886096519105748
RGD1564300	364.980041565241	-0.0879396933848722	0.156934822716001	-0.560358063704021	0.575235229951196	0.886096519105748
Rps13	4399.61320774126	-0.063484188608546	0.113115216397859	-0.561234735963846	0.574637526015297	0.886096519105748
Slc12a5	18.8073413789186	0.256484972953953	0.455200235441621	0.563455273051691	0.573124908425966	0.886096519105748
Ssbp1	363.473963349627	-0.108942062918795	0.193771761235679	-0.562218468904206	0.573967179550774	0.886096519105748
Upb1	5629.97703414929	0.178647678377613	0.31671455443018	0.564065262801163	0.57270971771058	0.886096519105748
Aldh3b1	75.2566201975683	0.247733564544473	0.442718187687887	0.559573948922837	0.575770078210856	0.886509587154366
Aldh9a1	15156.5293825278	0.0681112173468558	0.121688976685848	0.559715589709426	0.575673447008624	0.886509587154366
Anks3	507.160692252953	-0.0754327290376609	0.13482385910504	-0.559490950180351	0.575826705772584	0.886509587154366
Apba2	8.01001984319524	-0.565422571251512	1.01086751487262	-0.559343893173541	0.575927044830885	0.886509587154366
Jdp2	150.391826342671	-0.158892897393707	0.284022239413234	-0.559438224703694	0.575862680155581	0.886509587154366
Nsun2	979.660820048515	0.0641249234100761	0.114687966628436	0.559125122671563	0.576076330285024	0.886619305351332
Asb9	238.004738146253	0.102767033753354	0.183902354048797	0.558813041219067	0.57628932122867	0.886827027495947
Ddit4l	39.620473614562	0.264076671272556	0.472694210875477	0.558662799748404	0.576391872042353	0.886838009448092
Gcn1l1	1832.07164612772	-0.0541508916459374	0.0969647303208929	-0.558459673602264	0.576530534208336	0.886838009448092
Plxdc2	1469.47143042634	0.0703125233202891	0.125893923568209	0.558506092489794	0.576498845401648	0.886838009448092
Hyal3	79.0946952374799	-0.154948046441037	0.277774220585822	-0.55782011057129	0.576967228655566	0.886909591682332
Plcb3	485.121582124564	-0.102368135252869	0.183405999583255	-0.558150417573445	0.576741675151741	0.886909591682332
Rell2	30.0231805820313	-0.249770838294418	0.447741639431144	-0.557845901068642	0.576949615855388	0.886909591682332
Zfp608	119.877395487742	-0.213432230548695	0.382454232795348	-0.558059533002746	0.576803732461739	0.886909591682332
Zfp870	77.1368686838893	-0.168189163152709	0.30140267006403	-0.558021477105624	0.576829718518102	0.886909591682332
Cox6c	6349.72243829986	-0.0793600355282077	0.142403666956051	-0.557289269472955	0.577329805976145	0.887107012941039
Hira	650.589156158869	0.0643814578260172	0.115486432514809	0.557480705084224	0.577199038447954	0.887107012941039
Mfsd10	113.604673018041	-0.154384499032403	0.277023480478679	-0.557297521371245	0.577324168908818	0.887107012941039
Fbxo25	307.462430486164	0.0802912846248638	0.144293330541687	0.556444877413562	0.577906767205179	0.887444162039006
Fzd3	119.795898852643	0.131905622232816	0.236965019314057	0.556645966626818	0.577769341162711	0.887444162039006
Prkab1	1578.48956978983	0.0791185779003016	0.142227316073712	0.556282576965013	0.578017695897832	0.887444162039006
Scrib	1210.33765284587	0.0522306584712563	0.0938363374615795	0.556614419149103	0.577790899954428	0.887444162039006
Tgfbr1	1022.30228807156	0.0614350805497667	0.110389278864088	0.556531224607473	0.577847754929828	0.887444162039006
Ube2q1	1505.31441297956	0.0539386640780869	0.0969545176667428	0.556329559221652	0.577985583555945	0.887444162039006
Lyve1	184.428754244485	-0.26717843888861	0.480787967630494	-0.555709495404734	0.578409464312936	0.887925711963915
Sept9	735.28184419648	-0.0855034872107489	0.154295130245518	-0.55415544920111	0.579472464778012	0.887998386769164
Ascc1	861.403453177142	-0.0802579661976392	0.144702096343603	-0.554642733074596	0.579139053659019	0.887998386769164
Cox11	118.098935436705	-0.137998131019349	0.248950755414307	-0.554318988868667	0.579360557038772	0.887998386769164
Gipc1	1362.25794957886	-0.058815460115597	0.105997268151167	-0.554877131660767	0.578978704708611	0.887998386769164
Kif5a	23.5106937770026	-0.244946231252203	0.441878776035666	-0.554329025371503	0.579353689540926	0.887998386769164
LOC311352	94.1642449678421	-0.122991720155479	0.221458658610166	-0.555371015643967	0.578640913523334	0.887998386769164
LOC688765	68.7123508775351	-0.154591569506085	0.278926781369947	-0.554237096727712	0.579416593333448	0.887998386769164
Phkg2	520.97182074783	0.0709465265832595	0.128009741484968	0.554227559248612	0.579423119704904	0.887998386769164
Scyl3	338.535577652581	-0.0818526879080333	0.147382761846031	-0.555374908726056	0.578638251223793	0.887998386769164
Selrc1	399.606086376911	0.097799535911008	0.176199307991116	0.555050624352843	0.578860034098674	0.887998386769164
Socs3	22.6539853488411	-0.277610790492285	0.500246658419391	-0.554947815882351	0.578930354666434	0.887998386769164
Sppl2b	799.166774274498	-0.0843129743582899	0.151964759548794	-0.554819252888811	0.579018296866815	0.887998386769164
Uba6	520.949707757347	-0.0684969134418121	0.123412505024485	-0.555024091182839	0.578878182292856	0.887998386769164
Ptpn23	672.328655242327	-0.071250751132353	0.128665059893571	-0.553769229900412	0.57973678910247	0.888215156751666
Ube2f	588.384361320522	0.0889972409476684	0.160759102507677	0.553606231680837	0.579848360293627	0.888215156751666
Vezt	290.133448216987	-0.0753576503982749	0.136115826370293	-0.5536288645324	0.579832867656107	0.888215156751666
Slc12a6	848.63734908291	0.104135938882571	0.188158904897081	0.553446773829446	0.579957517869085	0.888236388039514
Zkscan5	327.302969424509	0.0789061466065674	0.14259519785905	0.553357671164799	0.580018517686897	0.888236388039514
Chtf18	50.0841270593385	-0.175361695330244	0.317188900169095	-0.552862017670723	0.580357897703406	0.888468239262557
Cmpk1	4269.52142794325	0.0889571901593163	0.160901078191955	0.552868825733972	0.580353235508992	0.888468239262557
Sdad1	162.391628843617	0.101052754070554	0.182803611534798	0.55279407896883	0.580404423414084	0.888468239262557
Cbfa2t2	136.779159518577	0.114197446027521	0.206643208778453	0.552631014116486	0.580516100445317	0.888519525674854
Bzw2	269.58147962916	-0.096719491850541	0.175121295044694	-0.552300003411103	0.580742828271022	0.888746867662372
Ap2a1	1097.91686472313	0.0526793702539588	0.0955406358721257	0.551381825890989	0.581371956548296	0.888752341375646
Bckdhb	5302.78724655738	0.119523802508201	0.216596918126589	0.55182596106167	0.58106759869182	0.888752341375646
Chmp2a	2592.41304196196	-0.0789326046105274	0.14302336685588	-0.551886075301705	0.581026409212504	0.888752341375646
Hax1	709.825182915827	0.0769098129373314	0.139464499025697	0.551465164788357	0.581314840215916	0.888752341375646
Rnf216	631.193142394606	0.0818306955343903	0.148251831724294	0.551970890225303	0.580968297476781	0.888752341375646
Rps3	7287.15235848463	-0.0828847283248682	0.15023475162375	-0.551701436778394	0.581152925424418	0.888752341375646
Ushbp1	211.330173944375	-0.185894112158641	0.336667222668217	-0.552159817297802	0.580838862111116	0.888752341375646
Zfp592	674.055892763783	-0.0623872461428859	0.113124203670319	-0.551493350836776	0.581295523495184	0.888752341375646
Adam4l1	27.4456669319037	-0.242561744087253	0.440217814301023	-0.551003926254988	0.581630983080407	0.888909204476523
Adamtsl3	35.9143886055079	0.202132270512249	0.366832724148334	0.551020280378568	0.58161977223611	0.888909204476523
Brd3	369.45815614476	0.0699993391208793	0.127088189855527	0.550793423058853	0.581775293231109	0.888931368566578
Rhod	412.334135543883	-0.0916742567781134	0.166452092269144	-0.550754607697458	0.581801904871263	0.888931368566578
Asb3	140.597921458211	0.121242756373444	0.221232647959459	0.548032840051987	0.583669353703968	0.889105204275836
Camk2d	129.339001088185	-0.162866289097796	0.297555503044087	-0.547347595428828	0.584139950766809	0.889105204275836
Chmp4c	849.65607799399	-0.0660551445520599	0.120067557436033	-0.550149815342512	0.582216621308244	0.889105204275836
Cnih4	448.933102134286	0.073514670338495	0.134224177870961	0.547700656502958	0.583897461277602	0.889105204275836
Dbp	2151.20550910336	0.413722598498663	0.753991784198643	0.548709690435653	0.58320469490936	0.889105204275836
Fam96a	2212.87114054194	-0.0846469560468018	0.154532012452791	-0.547763241436211	0.583854481570796	0.889105204275836
Gins3	122.945754513326	-0.147828679480058	0.268937565989458	-0.549676572464603	0.582541228276185	0.889105204275836
Ints7	422.770511467413	0.105276818140837	0.191772247035513	0.548967954269947	0.583027441873131	0.889105204275836
Itfg3	2392.44612148582	-0.0939476288707613	0.170907843942565	-0.549697583817939	0.582526814367345	0.889105204275836
Kctd21	25.947876350352	0.225745874893443	0.412462158339499	0.547312936057594	0.584163758050721	0.889105204275836
Lcmt2	212.896644617076	0.103639002590203	0.189007291722973	0.548333356059651	0.583463027730648	0.889105204275836
Lzts1	27.9838657762743	-0.25156148821538	0.457286981099464	-0.550117319348445	0.582238908273695	0.889105204275836
Mef2a	371.044835184795	0.0851160078261122	0.155453715968045	0.547532796473057	0.58401274509713	0.889105204275836

Mycbp	939.310662009148	0.0903648783163743	0.16460748317311	0.54897187281177	0.583024752671751	0.889105204275836
Nfe2l1	515.282409094546	0.0861126885170623	0.157275160729212	0.547528854002105	0.584015452856025	0.889105204275836
Pim1	39.9493810957798	-0.228324839892039	0.417087384489035	-0.547426866366996	0.584085501801927	0.889105204275836
Qk	119.069040898221	0.189618218499269	0.344696962799156	0.550101216324767	0.582249952475917	0.889105204275836
Rbpj	373.998737861594	-0.116233994759169	0.212383645342146	-0.547283170377448	0.584184204252327	0.889105204275836
RGD1304694	1203.90437416897	0.123819099996785	0.225749274018118	0.548480612109733	0.58336193821221	0.889105204275836
RGD1305110	104.762098509728	-0.145492437212102	0.26492847045981	-0.549176300152208	0.582884467109046	0.889105204275836
RGD1306151	790.205528460443	0.0971622911078834	0.177250443015527	0.548163883005764	0.583579379059655	0.889105204275836
RGD1309586	95.4968866284617	0.135841907654229	0.247308749623957	0.549280637505881	0.582812873047854	0.889105204275836
Rrm2b	107.040618212616	0.197808892972544	0.360104277084396	0.54931003478802	0.582792702002254	0.889105204275836
RT1-DOa	13.3565450617364	-0.435503099980456	0.792905520590964	-0.549249675618186	0.582834118005078	0.889105204275836
Slc25a17	1221.15348969594	0.084109077636808	0.153424128639718	0.548212842285838	0.583545765069951	0.889105204275836
Tcea2	33.5571789731294	-0.193928478158916	0.353233367898758	-0.549009509810802	0.58299892359557	0.889105204275836
Tfip11	876.580669201627	-0.0599323182607567	0.109079468020137	-0.54943720709834	0.582705446039458	0.889105204275836
Tnk1	313.919251026626	-0.0748817862488296	0.13637720566935	-0.549078461325732	0.582951605746949	0.889105204275836
Ubxn2b	50.4634914111133	-0.17731869901384	0.323585922740576	-0.547980262899134	0.58370545521517	0.889105204275836
Cyb5b	2123.87204623762	0.0576763941134405	0.105430599987476	0.547055542890694	0.584340573777329	0.889224121241547
Cadm1	107.196622055499	-0.167922609935473	0.307109837404064	-0.546783559116465	0.584527439548615	0.889247244829766
Lsm7	238.547656370859	-0.0892439391179668	0.163220745314436	-0.546768359291848	0.584537883370141	0.889247244829766
Rpl9	9180.91877448823	-0.0823143609966954	0.150599490005268	-0.546577953177771	0.584668719040369	0.889247244829766
Timeless	99.8996742232716	-0.131412902750999	0.240400067734606	-0.546642536291108	0.584624339869243	0.889247244829766
Zfp133	185.757637053927	0.125654214373158	0.229989012562782	0.546348771069475	0.584826217308625	0.889367779760481
Tefm	320.593243581356	0.0974339835108617	0.178630860674377	0.545448771522588	0.585444904716192	0.890189536722975
Erbb4	36.4697850098847	0.22691490678651	0.416132266623301	0.545295150092079	0.585550539181217	0.890231063180004
Emilin3	19.9165942131205	0.29299965290261	0.537606710129073	0.545007432723942	0.585748405633027	0.890336450334049
Man2b1	2655.78026636335	-0.0833139693683088	0.152908584148851	-0.544861296257939	0.585848917198452	0.890336450334049
Ppp2r2d	769.922269857437	0.0857131656214924	0.157345320435815	0.544745565893439	0.585928521354181	0.890336450334049
Racgap1	108.714768379875	0.232309702819582	0.426460741342844	0.544738777332898	0.585933190964164	0.890336450334049
Rilpl1	309.315407499916	0.0898816855497914	0.165052589248083	0.544563923288076	0.58605347280903	0.890400183390915
Smc2	247.739091542138	0.133086760586227	0.244481067577893	0.544364281065752	0.586190820409624	0.890489824214887
Zfyve1	471.492569301077	0.0730754192957035	0.134278100028324	0.544209512052147	0.586297306918976	0.890532565874793
Angptl2	77.7527912501749	-0.245148439537048	0.452008692816112	-0.542353373802879	0.587575093442364	0.891019644219253
Ccs	924.92979203189	0.0977898662776412	0.179997172226069	0.543285569813405	0.586933198122885	0.891019644219253
Cisd3	1825.12989552993	0.0949217311826717	0.174810837602611	0.54299683294495	0.587131983005108	0.891019644219253
Cog7	857.759083952287	0.0573682705014693	0.105697341669935	0.542759823426925	0.587295178772464	0.891019644219253
Dnajc2	715.016390209609	0.0862256293689552	0.159069907213481	0.5420612287982	0.587776326662865	0.891019644219253
Gtf2h3	215.839205471801	-0.116144077704394	0.213771552402981	-0.543309324364409	0.586916845362358	0.891019644219253
Itgb1	4783.47788970726	-0.0773677155597329	0.142684742720261	-0.542228370635361	0.587661193323335	0.891019644219253
Kcmf1	1354.09680103789	0.0676291426746233	0.124688909479856	0.542382983031453	0.587554700005725	0.891019644219253
Lrba	1454.55360510795	-0.0640818896871115	0.118128310884822	-0.542476982927428	0.58748995949227	0.891019644219253
Mapre1	2069.30392530066	0.0630771604851842	0.116324531365028	0.54225157621523	0.587645209305874	0.891019644219253
Ndufa7	1668.27626385351	-0.0833546037299065	0.153780641105166	-0.542035740850521	0.587793884599327	0.891019644219253
Sh2d4a	531.304620199592	-0.102387331321939	0.188347081934953	-0.543609862547801	0.58670997149069	0.891019644219253
Trim3	555.352818907506	0.0610804666967433	0.112487369931597	0.542998442704155	0.587130874657754	0.891019644219253
Uchl1	161.972704768787	0.22633121658686	0.416707775753752	0.543141332502054	0.587032496393843	0.891019644219253
Zfp295	367.180919494664	-0.0964049700475244	0.177773887183877	-0.542289824308166	0.587618864435264	0.891019644219253
Alkbh1	384.325369812016	-0.0940529494992943	0.173696036133666	-0.541480114301034	0.588176700504092	0.891295402125141
Atf5	294.423589660714	0.11455938783442	0.211764609299251	0.540975133727527	0.588524722237303	0.891295402125141
Cmbl	3808.67162989669	0.246670084025524	0.455915107152415	0.541043892066206	0.58847732987799	0.891295402125141
Fam173a	478.786673064765	-0.0726699941434469	0.134249498803187	-0.541305515411887	0.588297019538815	0.891295402125141
Pcid2	465.409266944002	0.0650170792203498	0.12006745558278	0.541504597601174	0.588159829558955	0.891295402125141
RGD1559909	600.390409470867	0.0955556620121509	0.17657885491584	0.541150083104198	0.588404140127594	0.891295402125141
Srsf7	1033.9534249285	-0.0893989189359164	0.165225803023036	-0.541071172300203	0.588458527197644	0.891295402125141
RGD1561909	18.2806529529542	0.32426196358616	0.599598563470263	0.54079843305402	0.588646522995612	0.891361095172945
Armcx6	51.3411284037676	0.210123509907559	0.38939312652802	0.539617922332378	0.589460554124692	0.891578892754779
B4galt3	1267.9753858479	0.0533570391895483	0.0989293405697783	0.539344939350058	0.589648865716758	0.891578892754779
Cd47	1081.11235716887	-0.0791062897880622	0.146511162633918	-0.539933533840844	0.589242870710292	0.891578892754779
Dnal4	495.594623359584	0.0755355992547228	0.139999645374677	0.539541361355374	0.589513365312606	0.891578892754779
LOC688452	71.7195200463822	-0.147655787868073	0.273467460921775	-0.539939148044784	0.589238998820038	0.891578892754779
Mpc1	8854.74099133184	-0.0720851492187019	0.13344884287076	-0.540170657669275	0.589079346181848	0.891578892754779
Plat	1248.98523553736	0.243634916685628	0.451729036967436	0.53933862281957	0.589653223371254	0.891578892754779
Prosapip1	1926.1674612332	-0.0868950440582295	0.161022071848464	-0.539646789168168	0.589440642564179	0.891578892754779
Pxdc1	244.536546671535	0.126152591414331	0.233505672361227	0.540254933161437	0.589021233447711	0.891578892754779
Snx5	2316.08450438172	0.075374519172191	0.139706907105864	0.539518916663694	0.589528847906175	0.891578892754779
Zswim4	219.521463861034	-0.0897220563013995	0.166257950523242	-0.539655733870342	0.58943447281413	0.891578892754779
Actg2	79.0021654659753	0.981156141212749	1.8197804048409	0.539161834363488	0.589775192393482	0.891644697624943
Cdk5	374.475482549248	0.0724932581057163	0.13458644628465	0.538637136999614	0.590137257467446	0.89166153014843
Fat1	3888.59783141108	0.0855647991396321	0.158984198714062	0.538196876367086	0.590441136317761	0.89166153014843
Itgb1bp1	477.357462158924	-0.0695230084638998	0.129277363896012	-0.53778176139036	0.590727724970762	0.89166153014843
Lipt1	71.8923385502285	0.14380004562291	0.267195350329686	0.538183188612669	0.590450585101106	0.89166153014843
Pax9	38.0723841331047	-0.201026935054766	0.373597355023692	-0.538084470758681	0.590518733009012	0.89166153014843
Pdia6	3566.42287675292	-0.061004333843095	0.113293720407758	-0.538461740187656	0.590258312081729	0.89166153014843
Porcn	144.647032375769	-0.155960931244264	0.289988430168881	-0.537817771396729	0.590702861710871	0.89166153014843
Rabep2	697.072304369506	0.0711619333509513	0.132159549751889	0.538454719954388	0.590263157513998	0.89166153014843
RGD1308134	462.932395675789	0.0702478029951485	0.130566407925347	0.53802355530309	0.59056078658885	0.89166153014843
RGD1310352	3170.01997224592	0.0528400880803579	0.0982424124123619	0.537854138379332	0.590677752463942	0.89166153014843
Snapc3	182.395058738173	-0.105686331928979	0.196077230177409	-0.539003594825137	0.589884374098249	0.89166153014843
Sync	54.3897927616325	-0.156459345589985	0.290379038680949	-0.538810743022993	0.590017450073458	0.89166153014843
Stk24	758.241943669357	0.0893445297320544	0.16619825127365	0.537578037358203	0.590868396149824	0.891755436281888
Rbks	495.598973979244	0.087295056825069	0.162446777253312	0.53737635366533	0.591007673653407	0.891782552873958
Rbms2	551.898211121597	-0.0888929820650365	0.165444956556614	-0.537296415165235	0.591062881276452	0.891782552873958
Sfr1	1532.04472607119	-0.0866903785550402	0.161371134328812	-0.537211186595484	0.591121744972057	0.891782552873958
Smarcd3	510.962082968851	-0.129471995728253	0.241112954785562	-0.536976521412552	0.59128383201393	0.891908697368326
Klhl23	60.3524792154022	-0.16693432173592	0.311088654508215	-0.536613339370469	0.591534727997888	0.892168752444797
Lig1	545.820472278335	0.0705286597219067	0.131512881768827	0.536287082856886	0.591760156524408	0.892271947340995
Ppt2	1211.34633504496	0.058565920324206	0.109204748890983	0.53629462929924	0.591754941827607	0.892271947340995
Piga	35.8549607301414	-0.199587061784229	0.372349851532487	-0.536020253433122	0.591944552937516	0.892431594201859
Armc9	503.771922240257	0.059340362308606	0.110758020088	0.535765827715758	0.592120402151059	0.892578314436198
Atf6b	1138.87274812892	-0.0896193359982529	0.167436785843055	-0.535242811470693	0.5924819660281	0.893004909942367
Rps16	10583.2298163753	-0.0704101598873636	0.131666187043339	-0.534762655989934	0.592813989162245	0.893386873616823
Abhd13	595.564116953572	0.0620739908586387	0.116277271124223	0.53384457906931	0.593449068303516	0.893419924031892
Aga	978.846730625184	-0.0610050366183936	0.114158348820123	-0.534389619759813	0.59307199916802	0.893419924031892
Dnase1l1	357.493935385647	-0.0752864794392671	0.140944137597575	-0.534158289394241	0.593232024345053	0.893419924031892
Nf1	252.962710310886	-0.103680278038982	0.194222599377756	-0.53382190523219	0.593464756857363	0.893419924031892
Pes1	1253.49008076105	0.0623283904744492	0.116677640223321	0.534193101224475	0.593207941636661	0.893419924031892
Rabepk	227.395742986145	0.107506210634111	0.201384859710701	0.533834622863647	0.593455957209774	0.893419924031892
Rps6kb1	464.791785149935	-0.0606453896960593	0.113446370437753	-0.534573203726555	0.592945017135038	0.893419924031892
Rrs1	354.593945743156	0.0988433732870131	0.185107117957687	0.533979321689872	0.593355840676422	0.893419924031892
Lsm3	499.553908838271	0.0716296848059737	0.134231631730039	0.533627460850898	0.593599305231364	0.893504132334749
Ppcdc	269.341620477338	0.102028822874093	0.191246372451024	0.533494160262943	0.593691552411561	0.893507707647902
Tceal8	1246.21234095931	-0.0742793744855598	0.139257245349248	-0.53339683906049	0.593758905143815	0.893507707647902
Gne	368.235743870177	-0.088358277982665	0.16586829006215	-0.532701446126667	0.594240264934914	0.894053561836969
Ufsp2	1296.25890372864	0.0615245186051255	0.115507428585064	0.532645556729857	0.594278960028005	0.894053561836969
Mylk	1337.70764709043	-0.233008932095317	0.437897913142129	-0.532107884286009	0.594651277044696	0.894495290483061
Snrpf	394.645551138911	-0.105765642266063	0.198874640139457	-0.531820659446057	0.594850212609062	0.894676130278496

Dbr1	421.850833400247	0.0920996694367263	0.173314370182345	0.53140238365594	0.595139970071644	0.894875102504869
Mgme1	121.507627192154	0.118014868264526	0.222079050673122	0.531409279294121	0.595135192646959	0.894875102504869
Ccdc51	303.8655762496	0.08522870854177	0.160422992504232	0.531274895271149	0.595228299517992	0.894889530730357
Kctd5	391.654963816982	0.0985825328598258	0.18564097370965	0.531038654289829	0.595391993263618	0.894898888578985
Ttll9	13.5244643254225	0.308404068285058	0.58069073250339	0.531098657206929	0.595350414694543	0.894898888578985
Hcfc1	1019.53418945645	-0.0581607449369907	0.109751298835297	-0.529932178973775	0.596158954356086	0.894946420641827
Hras	680.636586776234	0.0813626643749567	0.153310584318176	0.530704809043706	0.595623352999394	0.894946420641827
Mplkip	423.449693775684	0.0886592188450613	0.167272154339297	0.53002975417668	0.596091301327157	0.894946420641827
Osbpl7	253.032188769563	-0.101366144153765	0.191308453770312	-0.529857108538796	0.596211006255491	0.894946420641827
Pparg	20.1688032931468	0.338674702747407	0.638965975459027	0.530035582104519	0.596087260688192	0.894946420641827
Rad51c	147.098201125092	0.129397033334323	0.244198030458763	0.529885655061307	0.596191212590592	0.894946420641827
Slc8a1	26.4119229225008	0.414903909439609	0.782757599058106	0.530054144397785	0.596074391098397	0.894946420641827
Smu1	1296.11881562022	0.0736067103783784	0.138787375209867	0.530355951087585	0.59586516056792	0.894946420641827
Spcs2	1623.42314195656	-0.0693554427817487	0.130835324316989	-0.530097228281514	0.596044520713124	0.894946420641827
Wnt9a	25.252225679591	-0.255079518794348	0.481116045157871	-0.530182938942824	0.595985098886316	0.894946420641827
Actb	22248.2545472875	0.0470823239732182	0.0890861104082822	0.528503531666605	0.597149896502338	0.895161361414894
Aimp2	970.388004943324	-0.0967559284410703	0.182777328806883	-0.529365042550214	0.596552243349169	0.895161361414894
Cbfa2t3	29.1816328590223	-0.33673066743719	0.639075759358065	-0.526902581589744	0.598261241024307	0.895161361414894
Dip2a	195.662300961473	-0.150525216509944	0.285014769002903	-0.528131286096303	0.597408217444221	0.895161361414894
Fbxo2	41.6783201232903	-0.193078939171797	0.366517009000515	-0.526793939791008	0.598336691789179	0.895161361414894
Gadd45b	647.586309841778	-0.43459456111448	0.825119768460232	-0.526704822410791	0.59839858624268	0.895161361414894
Hars2	644.267770612565	0.0674627645902404	0.128123931092933	0.526543043245428	0.598510953713689	0.895161361414894
Hmgb1	5579.36919727453	-0.0759342934090075	0.144243757622732	-0.526430361080948	0.598589225379863	0.895161361414894
Ino80c	1002.89316224655	-0.075322351050055	0.142404205656625	-0.528933472875636	0.59685160073843	0.895161361414894
KIFC2	932.204314903677	0.0882965444465451	0.167491319477908	0.527170869044298	0.598074936271737	0.895161361414894
Lrrc47	1620.29598287044	-0.0757645491919885	0.143784929473829	-0.526929696104061	0.598242410908236	0.895161361414894
Mtfmt	572.48861650288	0.0765136457381595	0.144814770018155	0.528355261887768	0.597252782677064	0.895161361414894
Nup155	226.439492002129	0.0923978106952892	0.174600989863547	0.529194082848552	0.596670821006809	0.895161361414894
Pla2g6	844.795383312156	0.141520082228273	0.268516305723535	0.527044649474593	0.598162582656042	0.895161361414894
Pnrc2	1593.04909374674	-0.0831679807114762	0.158005009831598	-0.526362934948183	0.598636063368701	0.895161361414894
Podxl	1315.46704888873	0.316610274767574	0.600617698797436	0.527141100572785	0.598095606858525	0.895161361414894
Pus3	290.80913454713	-0.130078304395054	0.245930736210033	-0.528922518590613	0.596859200044534	0.895161361414894
Ramp3	309.206062993533	-0.361521693360894	0.684932615080081	-0.527820818283892	0.597623706370249	0.895161361414894
RGD1304704	2539.45675967704	0.0693743239478828	0.131141421439837	0.529003904229522	0.596802741511567	0.895161361414894
RGD1311747	416.018188105656	-0.0757988402195375	0.143791998486757	-0.527142268118059	0.59809479613407	0.895161361414894
Selm	436.886503165892	-0.12511472227828	0.23703816454834	-0.527825223911421	0.59762064827338	0.895161361414894
Slc25a45	651.687631236092	0.247818073148034	0.470814885548408	0.526359893781552	0.598638175973483	0.895161361414894
Slc3a1	114755.581716822	0.0839559217470993	0.159016108700302	0.527971174953924	0.597519342679325	0.895161361414894
Spsb3	1906.27737757081	0.0660333939278132	0.125424894639984	0.526477571437103	0.598556431397416	0.895161361414894
Tmed1	681.840168497951	-0.0663224793220599	0.125618279002356	-0.527968380468069	0.597521282277339	0.895161361414894
Tmem135	1055.37088202076	0.0570882008105446	0.108451938907001	0.526391702959765	0.598616079284491	0.895161361414894
Tmem164	655.513489838634	0.0707874017863189	0.134397091237088	0.526703376797373	0.598399590284612	0.895161361414894
Ube2g2	1038.80387901198	-0.0523624505296485	0.0990981780963227	-0.528389638795907	0.597228927411099	0.895161361414894
Zyx	2209.48613943451	0.11529866205198	0.218628229253816	0.527373168805773	0.597934472258804	0.895161361414894
Homer1	446.581032062795	0.103765891853473	0.197199327838736	0.526197999712906	0.598750643677584	0.895211762173036
Fkrp	235.464494025676	-0.0781320976309507	0.148553596313409	-0.525952245990143	0.598921386939432	0.895235930737669
Ubr2	739.413718628812	0.085707209383833	0.162957572637394	0.525947999817993	0.598924337262822	0.895235930737669
Kdelr1	2803.99724456226	0.0422878235773051	0.080464463195085	0.525546581660264	0.599203280192277	0.895535106201896
Fxr2	1439.07666054468	-0.0579563032445177	0.110341379531215	-0.525245411021187	0.599412600401175	0.895698039889013
Plb1	16.3872831310505	0.25348183240806	0.482766214594424	0.525061250653202	0.599540612218055	0.895698039889013
Zfp819	54.5638056565165	-0.16812774343699	0.320213071162965	-0.525049595340925	0.599548714365266	0.895698039889013
Rps5	5112.3366737892	-0.0826718388756378	0.15754638683515	-0.524746016309103	0.599759763668181	0.895895580740248
Farp1	1014.76227902236	-0.126679157552668	0.241468760982799	-0.524619238683598	0.599847909911277	0.895909506445674
Sqle	520.08063966842	0.0768470378620609	0.146519741758272	0.524482482291314	0.599943000787739	0.895933799363216
Rpap2	326.773080656306	0.089906275090575	0.171474510391848	0.524312767449368	0.600061018160489	0.895992319024187
Ankrd9	180.825956693008	0.144252652388082	0.275929882386619	0.52278735141112	0.601122242659793	0.896122865953509
Fpgs	674.614513257622	-0.0758517862958747	0.145034282071891	-0.522992117534502	0.60097973863048	0.896122865953509
Gmpr2	296.725748232266	-0.0793217018103711	0.151708969487989	-0.522854397324549	0.601075581343634	0.896122865953509
Lrrc24	73.108825312207	0.158029028197692	0.301893814477115	0.523458980010573	0.600654889492468	0.896122865953509
Midn	446.091804097556	-0.0997854948949849	0.190940270575621	-0.522600573436735	0.601252241400797	0.896122865953509
Neurl1B	26.6615425045195	0.285312173696707	0.545940049967446	0.522607150205814	0.601247663709202	0.896122865953509
Pcgf3	174.345295073426	0.109628720846391	0.209466728185514	0.523370569617616	0.600716400532044	0.896122865953509
Ppp1r14c	295.833945225132	-0.123427086978998	0.235997483002599	-0.523001709207374	0.600973063818244	0.896122865953509
Rilpl2	722.569819179002	-0.0505747500448777	0.0966300274214373	-0.523385446475179	0.600706049841885	0.896122865953509
Rps10	5157.59645129306	-0.0976563994281191	0.186449151801326	-0.523769609486765	0.600438793334019	0.896122865953509
Srsf5	1064.80649620748	0.072938770396992	0.13931103475254	0.523567788628907	0.600579190408533	0.896122865953509
Supt7l	222.104383655159	-0.0960578811067068	0.18352444877142	-0.52340645483342	0.600691433249374	0.896122865953509
Tbc1d22b	735.781679987048	0.0535762065928066	0.102440726217116	0.52299713767409	0.600976245128393	0.896122865953509
Tgfb1i1	137.413187813415	-0.192426532318278	0.367929061727125	-0.522999002620215	0.600974947319569	0.896122865953509
Fpgt	163.907235457915	0.118177612774735	0.226183796963988	0.522484874518004	0.601332775000621	0.896125386214378
Strn	109.675392841202	-0.222069096485508	0.425258338154605	-0.522198100686677	0.601532408302084	0.896187882128915
Trmt10b	171.412215995923	-0.110095753336073	0.210798579638342	-0.522279388812579	0.601475817755662	0.896187882128915
RGD1562963	28.330291676655	0.244693759297809	0.468759376791664	0.522002911115229	0.601668303695629	0.896272862359701
Zfp709l1	195.570639049815	-0.101902516579599	0.19527475664119	-0.521841728712747	0.601780532967696	0.896322570791618
Ddx55	410.938050433767	0.0959523124012579	0.184085663049077	0.52123729144337	0.602201479068088	0.8965347092408
Irx1	881.180811041178	-0.0810664543952303	0.155560140866513	-0.52112613130631	0.602278908370781	0.8965347092408
Prpf6	1528.05660482562	0.0470818601451652	0.0903210717248263	0.521272159929696	0.602177192125752	0.8965347092408
Tmem150a	4020.03126102513	-0.104957315836094	0.20127928503643	-0.521451155875765	0.602052523052199	0.8965347092408
Ubl5	1878.26377328137	-0.0796320623811604	0.152823840631932	-0.521070940580205	0.602317353489596	0.8965347092408
Agmat	6608.73680786264	0.147264812615871	0.282690096359079	0.520940827119787	0.602407993154852	0.896552212936762
Dpep3	21.8027869604583	-0.410761159234651	0.789010861693035	-0.520602667437622	0.602643590752236	0.896785421902319
Parp3	791.779139324205	0.0785674907317395	0.150970536478917	0.520416053119818	0.602773623674246	0.896861501071015
Prrt1	268.718162973602	-0.145751941533402	0.2801449400512	-0.520273332464132	0.602873079999946	0.896892071633427
Adamts3	34.4654634882437	0.256290797755217	0.49305313711539	0.51980360424162	0.603200466985696	0.896895323241165
Iffo1	279.411694466792	0.101092514376562	0.19460413483402	0.519477730844646	0.603427638291852	0.896895323241165
Med11	217.567917308662	-0.105294006533898	0.20252811360662	-0.519898223801243	0.603134513457439	0.896895323241165
Mtmr14	776.463924558723	-0.0856602659212014	0.164777933976189	-0.519852773087807	0.603166193974811	0.896895323241165
Nudcd1	190.033946694018	0.112221989950773	0.21580764628085	0.52000933185068	0.603057070949529	0.896895323241165
Tox	312.565227172866	0.139024786503537	0.267576108728575	0.519571000430988	0.603362614713921	0.896895323241165
Ubxn7	17.5637218035145	0.257695420673784	0.495943362829727	0.519606551851889	0.603337830608836	0.896895323241165
Mtap	74.2735289732467	0.139882235418545	0.270152304024675	0.517790273614576	0.604604605238047	0.898527189217527
Entpd7	28.4173794527839	0.222453121829716	0.429966931344438	0.517372629411616	0.604896062459987	0.89867894172562
Slc4a2	1410.80054280208	0.0533505801535497	0.103094481255459	0.517492105337352	0.604812678546049	0.89867894172562
Znrf2	1722.14719792075	0.060333107702567	0.116629874268109	0.51730406194105	0.604943918981411	0.89867894172562
Abhd3	216.374609401462	-0.120673519871245	0.233975445502635	-0.515752922756529	0.606026986544162	0.898985865326315
Ankmy2	714.695599802903	0.0707594855736303	0.137428334776161	0.51488279828815	0.60663492195492	0.898985865326315
Apoc3	101.756343999703	0.204225779740156	0.396654097003344	0.514871222264054	0.606643011686493	0.898985865326315
Atg10	164.193527538682	0.107572843328734	0.208804757523466	0.515183871309276	0.606424538507242	0.898985865326315
Cdc42bpb	2512.19090428356	0.0617256340780035	0.119712977081375	0.515613558219721	0.606124338917569	0.898985865326315
Cib2	578.205940887181	-0.0808293325793391	0.156798423151366	-0.515498376544961	0.606204803758592	0.898985865326315
Cnbd2	262.01459564653	0.0945316654625275	0.183665218968863	0.514695520432498	0.606765804207029	0.898985865326315
Dedd	497.282644252508	0.0718291411509516	0.139475517571629	0.514994619855511	0.60655677960501	0.898985865326315
Edc4	994.739557623476	-0.0722378252962169	0.140368432948087	-0.514630132851401	0.606811504380036	0.898985865326315
Fancd2	115.284463114489	-0.12472081460591	0.242073765224967	-0.515218220735333	0.606400537929606	0.898985865326315
Impdh2	1051.57293068484	-0.0640373720228033	0.123979047077529	-0.516517698210393	0.605492881214273	0.898985865326315

Itgb7	16.1514333811759	-0.35020940538871	0.678893105185434	-0.515853530863379	0.6059567116225	0.898985865326315
Meis2	265.753584053948	-0.167150236281489	0.323761557299719	-0.51627573599404	0.605661840444413	0.898985865326315
Mlycd	2463.02747973204	0.0866064712964677	0.168028978998506	0.515425802219733	0.606255505957301	0.898985865326315
Mocs2	679.960834897269	0.0762494834006325	0.147752423733744	0.516062488003834	0.605810766383849	0.898985865326315
Mtbp	35.8918703703161	-0.177898898042205	0.345494882316849	-0.514910371028465	0.606615653351301	0.898985865326315
Paics	3146.42862603521	0.0653350512266046	0.12693018159448	0.514732196912306	0.606740171243998	0.898985865326315
Taf1b	59.1410076399016	-0.156322992365965	0.302838823183256	-0.516192048043224	0.605720283621003	0.898985865326315
Tbc1d7	133.245407541252	-0.119541230125855	0.231872928587376	-0.515546298803091	0.606171325136703	0.898985865326315
Trmt12	138.908740214633	0.0960284925621376	0.186020556133432	0.51622516649857	0.605697155166075	0.898985865326315
Ubtf	1101.9826159751	-0.059872952853483	0.116193827077458	-0.515285143448887	0.606353779015025	0.898985865326315
RGD1305713	342.131942835083	0.120019895899129	0.233435100327684	0.514146740274499	0.607149401010101	0.899369228708563
Cdt1	30.9796711079495	0.269982250098931	0.526979886551219	0.512319837984349	0.608427183486639	0.900365424465227
Itpkc	210.410053549256	-0.151425428286206	0.295523820452934	-0.512396692943817	0.608373405019211	0.900365424465227
Mospd3	402.705356767482	0.0680182401835766	0.132728056346258	0.51246316759233	0.608326891775635	0.900365424465227
Ncoa1	558.432843122827	-0.0602094049678034	0.117510023257269	-0.512376759861454	0.608387352785187	0.900365424465227
Ran	3133.51630236389	0.0780691364741734	0.152384931425739	0.512315330287225	0.608430337767083	0.900365424465227
Rps4y2	770.82002108286	0.0630897757103907	0.122994499484131	0.512947944623577	0.607987734410929	0.900365424465227
Stk3	345.020482607567	0.0915332302096817	0.178678415402316	0.512279169275056	0.608455641854426	0.900365424465227
Wdr92	146.047290422901	-0.135246550052698	0.263900251594694	-0.512491175114201	0.608307294994321	0.900365424465227
Flvcr2	407.252468266673	0.13123720401397	0.256455563506334	0.511734673327643	0.608836715859037	0.900812042756289
Acad9	766.39550731568	-0.0739675150023592	0.144667992443171	-0.511291500996085	0.609146955342593	0.900833381423583
Cops7a	945.152016516815	0.0585144154640509	0.114451115698088	0.511261206211453	0.609168165548272	0.900833381423583
Fbxo16	44.3502162296388	0.219774955627104	0.429758463736338	0.511391803005742	0.609076733512285	0.900833381423583
MGC94282	112.000364637634	-0.111654952256965	0.218305056690119	-0.511462968150376	0.609026912699769	0.900833381423583
Ccnh	290.884963827332	0.0969276349679308	0.189676886311934	0.51101447758125	0.60934091922886	0.900854433917172
LOC100125364	750.629141972322	0.0904867247606464	0.177071573853111	0.511017792362931	0.609338598151016	0.900854433917172
Dctn4	158.040614098114	0.118470032847937	0.231936926860751	0.51078555903719	0.60950122217989	0.900857072990459
Sema3c	980.120288902497	-0.0926375571054879	0.181338342993288	-0.510854767813316	0.609452755928663	0.900857072990459
Tf	2918.78801294235	-0.240812520865476	0.471685632800386	-0.510536052234149	0.609675963655205	0.900998180068269
Mrfap1	8250.08302935756	0.0750722079230918	0.14709615457613	0.510361458050475	0.609798253500871	0.901061745877652
Pgap1	43.6350739329987	-0.212789192862614	0.417102194946404	-0.510160808168262	0.60993880682442	0.901110422391488
RGD1559502	22.8676169738446	-0.217716190651365	0.426820779732928	-0.51008807675108	0.609989758039777	0.901110422391488
Arhgap24	7030.71062216223	0.0715217294533321	0.140769056833778	0.508078487289901	0.611398301384757	0.901127403589059
Arl6	339.731302761444	0.10333201923777	0.203465010589466	0.507861371045583	0.611550566749544	0.901127403589059
Cabp7	185.65709597514	-0.123232213513506	0.241906434449316	-0.509420982513489	0.610457171918475	0.901127403589059
Camsap1	306.318805938659	-0.0826617806386806	0.162770026152195	-0.507843996789614	0.611562752180348	0.901127403589059
Ddx31	174.80792507147	0.0908416708670372	0.178186704667806	0.509811722689376	0.610183372152937	0.901127403589059
Dscr6	12.7375225866007	0.302451737178425	0.595323586359661	0.508045950317346	0.611421118754986	0.901127403589059
Emb	731.839174354871	-0.102086912533188	0.201372222957583	-0.506956277453876	0.612185496979741	0.901127403589059
Esf1	530.703648262796	-0.079033866086354	0.155048149618558	-0.509737563981183	0.610235332509582	0.901127403589059
Gsdmd	168.316439297091	0.134185626113399	0.263589662155001	0.509070139611518	0.610703061277173	0.901127403589059
Hebp2	44.9381649289195	-0.180864306312345	0.355217473741421	-0.509165003645075	0.61063657118565	0.901127403589059
Kctd10	792.688148904989	0.108100338470974	0.212476578022019	0.508763551622013	0.610917970401038	0.901127403589059
LOC100125385	108.902425221301	0.148748582489122	0.293439788069767	0.506913474370954	0.612215530896268	0.901127403589059
Lrrc42	707.935895680431	0.0548493288821277	0.108033097258709	0.507708565929372	0.611657740271211	0.901127403589059
Mapk12	131.352594086984	-0.193973479136462	0.382515369562749	-0.507099830676587	0.612084773818132	0.901127403589059
Mcm10	46.7620375709457	-0.203522734886438	0.40105047531903	-0.507474114634919	0.611822194457406	0.901127403589059
Mdc1	126.692822869985	0.166878029263454	0.327618008704508	0.509367692952337	0.610494517246328	0.901127403589059
Med29	578.777922154844	0.0571628560192264	0.112217628392221	0.509392836386025	0.610476896600079	0.901127403589059
Mettl18	122.217473716081	-0.11539530704677	0.227019107225522	-0.508306584661772	0.611238352958668	0.901127403589059
Ogfod3	353.851737211574	0.0730338498006314	0.143820660317274	0.507811948850156	0.611585229278077	0.901127403589059
Pdlim1	308.395622038952	-0.123224672728673	0.2427397997872	-0.5076409918633	0.611705137609748	0.901127403589059
Rab17	2288.35173270641	0.0736880741697405	0.145210746477861	0.507456066145732	0.611834855252497	0.901127403589059
Rpia	316.139656627482	0.101731650347724	0.199945408387224	0.508797132018683	0.610894429899827	0.901127403589059
RT1-CE7	189.23816326763	0.468660374685887	0.920405033591179	0.509189278178214	0.610619557709432	0.901127403589059
Slc38a7	85.7279931993282	0.145548893764449	0.28716970917623	0.506839297856196	0.612267580355164	0.901127403589059
Slc39a1	1161.4522381309	-0.0714780734186093	0.140885005721842	-0.507350466803634	0.611908934227264	0.901127403589059
Tbck	457.679139180992	-0.0690216843724778	0.135909669847316	-0.507849694948258	0.611558755766121	0.901127403589059
Trip11	269.810765827709	0.13246004954592	0.26028805963789	0.508897909993248	0.610823785024341	0.901127403589059
Ttc33	221.904417235441	0.104052132558925	0.205315078936193	0.50679245332615	0.612300452042786	0.901127403589059
Zfp777	374.869330189125	0.0815741655949462	0.160566987534022	0.50803821412955	0.611426544006727	0.901127403589059
Prdx4	406.723487099061	-0.0933714022766791	0.184365846951627	-0.506446306734768	0.612543373800161	0.901368201270408
LOC100361087	37.5169036315475	0.198606869121464	0.392481077841732	0.506029157414699	0.612836181055423	0.901682334482321
Cdk2ap2	1886.94600864151	-0.0669568477577127	0.132525015688164	-0.50523931206516	0.613390762017094	0.902090696600202
Efcab2	120.04622487725	-0.147046586696731	0.290987162441315	-0.505337024022104	0.613322142670416	0.902090696600202
Gpbp1	1491.85894556936	-0.0655900830227471	0.129834628774346	-0.505181734964892	0.613431197784882	0.902090696600202
Kdr	461.836218557992	0.350673954871272	0.693937031949419	0.505339733615532	0.613320239875515	0.902090696600202
Zfp511	225.483844622752	0.0782244566643694	0.154919505957859	0.504936135580293	0.613603692754084	0.902227628957687
Mars	1276.18179254851	0.0558237669409619	0.11067078673487	0.504412849930278	0.61397129002189	0.902352286250262
Mccc1	6009.68148953543	0.070054330257615	0.138896538739175	0.504363398062535	0.614006033948577	0.902352286250262
Pmepa1	628.047591192906	-0.100193242718799	0.198615553716348	-0.504458189925494	0.613939435778934	0.902352286250262
Rab3a	210.63212514105	0.137132516573342	0.271750163725249	0.504627171860653	0.61382072159307	0.902352286250262
Kcnip4	31.6740924000234	-0.19317197084496	0.383267350189203	-0.504013636302697	0.614251794538328	0.902596754585991
Alg14	743.415211733474	0.0653676765945483	0.12993559213069	0.503077528817517	0.614909764873986	0.902768459580522
Mblac2	51.6819618441191	0.199755306721065	0.39708334121158	0.503056376305215	0.61492463611406	0.902768459580522
Mtr	381.072783544827	0.0772088618557505	0.153460278549891	0.503119521125134	0.614880242717938	0.902768459580522
Rybp	640.468597927246	-0.0532785184008536	0.105841861638613	-0.503378508049754	0.614698179052453	0.902768459580522
Slc12a9	192.795398164591	0.0901197949791758	0.178933752626472	0.503648940774761	0.614508094502863	0.902768459580522
Tm2d2	1162.34476345265	0.0563509529278645	0.111967119186571	0.503281260938459	0.614766539433948	0.902768459580522
Wdr73	295.646008089228	0.0805862117223782	0.160156482204664	0.503171714394908	0.614843549775003	0.902768459580522
Cd97	235.830069881064	-0.194432051327768	0.386862849637036	-0.502586514859693	0.615255012243604	0.902997106817573
Gemin6	514.425667849057	-0.324571016771789	0.645836886184315	-0.502558809685515	0.615274495163833	0.902997106817573
Nacad	12.0531599024373	0.367066492133215	0.730486512834198	0.502495919752223	0.615318721828444	0.902997106817573
Manbal	920.906425908183	-0.0461820629152976	0.0919342164620433	-0.502338135816545	0.615429687824564	0.90304335551433
Glcci1	195.609563750155	0.134988513834527	0.268836579697387	0.50212108034731	0.615582352567689	0.903043650272603
Uba2	575.931564924674	-0.0586223465669936	0.116751551773138	-0.502111926365689	0.61558879133487	0.903043650272603
Alkbh8	38.0763756210014	0.1771275542457	0.352894709472223	0.501927485709847	0.615718530305916	0.90311741069261
Adsl	852.425911884407	-0.05948774963811	0.118641119682447	-0.501409206161693	0.616083162019116	0.903372376312375
Ccnd2	26.5602613660103	-0.302838450872763	0.604192333244498	-0.501228556222367	0.61621027921431	0.903372376312375
Cops3	1183.78403435694	0.0636803595387651	0.126987182916363	0.501470763239993	0.616039849034533	0.903372376312375
Ddx10	291.956259470739	-0.086366756076686	0.172280997944959	-0.50131330272581	0.616150644559427	0.903372376312375
Patl1	586.316737708268	0.0614392710319611	0.122660479380312	0.500888887295696	0.616449323808371	0.903489750406957
Samd14	12.4395728676627	0.300324728865308	0.599546869970808	0.500919517568211	0.616427765851966	0.903489750406957
Suv39h1	225.834878394543	0.0814948189795113	0.162859925088919	0.500398234464472	0.616794696280946	0.903762861664205
Tdp1	67.758587457234	0.140797664606371	0.281334580441433	0.500463414008509	0.616748811255528	0.903762861664205
Prpf31	426.144803283529	-0.0615533870144438	0.123076252627381	-0.500123993869063	0.616987772274495	0.903929236874441
Slc5a11	1048.93264794849	-0.0816195046374099	0.163304585060174	-0.499799222461113	0.617216458167093	0.904031219526699
Ugp2	2368.89337233741	-0.0905905225443907	0.181253232364813	-0.499800866238109	0.617215300617623	0.904031219526699
Sertad2	61.0245204832845	0.168335305620672	0.337023111462954	0.499477038503264	0.617443358802798	0.904247032103157
Cdyl	82.3204165794907	0.130627732937351	0.261719249687019	0.499113966945742	0.617699098619517	0.904272018921873
Haus8	214.564696232496	0.101267553165767	0.202875295825597	0.499161579795411	0.617665558508555	0.904272018921873
Tsen54	284.713684799275	-0.0948664446300961	0.190046134336885	-0.499175870959476	0.617655491481977	0.904272018921873
Sep15	2579.88973760574	0.0543280563681308	0.108903582991091	0.498863810317198	0.617875330552011	0.904357606148155
Cyp4a2	154550.853617949	0.14700726504574	0.294902180938312	0.49849500799891	0.618135186867164	0.904357606148155
Mk1	6493.33049123259	0.10149240302547	0.203563422990635	0.498578779696289	0.618076157542997	0.904357606148155

Nf2	1542.16519447994	-0.0566234925424421	0.113595421425893	-0.49846632753048	0.618155396987948	0.904357606148155
Sulf2	708.32435118951	0.0891604921783282	0.178754891175842	0.498786307842177	0.617929934451476	0.904357606148155
Bcam	4017.35694900875	-0.219496000457155	0.441785602861014	-0.49683828317558	0.619303096102252	0.90459213912338
Ccni	7619.83192634477	0.0687142215667667	0.138232776255192	0.497090656993774	0.619125122863129	0.90459213912338
Cep63	405.29036857265	-0.155981321821882	0.314061553314942	-0.496658442192264	0.61942993302809	0.90459213912338
Ctif	45.2978444369152	0.192818860650198	0.387650990165737	0.49740324555281	0.618904717326682	0.90459213912338
Dock1	1081.5561983497	-0.0962967137875213	0.193445993367953	-0.497796372573898	0.618627572938864	0.90459213912338
Efcab4a	510.379557495862	-0.0657500972063943	0.132061104025533	-0.497876325444636	0.618571214866893	0.90459213912338
Eps8l2	6740.00959410462	-0.0601433889532533	0.121069275314566	-0.496768389808121	0.619352388636193	0.90459213912338
Hoxb4	334.099931596517	-0.11002266215398	0.221139724032475	-0.497525546960629	0.618818492186212	0.90459213912338
Mob2	473.827518543755	0.0703907191324148	0.141580219560888	0.497179050510954	0.619062793305872	0.90459213912338
Ndn	21.4095523786726	-0.281308151074262	0.566322912839782	-0.496727475961841	0.619381244058931	0.90459213912338
Polr2c	941.772834365622	0.0939804379984325	0.1889161467085	0.497471707082009	0.618856449819953	0.90459213912338
Sfmbt1	469.281286406203	0.0649745586470649	0.130469771565104	0.49800469386614	0.618480733793611	0.90459213912338
Ube2t	20.9329253482324	0.26012303057337	0.522786792208168	0.497570012193024	0.618787144535084	0.90459213912338
Wdr19	456.11504655005	0.0748364809144674	0.150527274131378	0.497162267411759	0.619074627482801	0.90459213912338
Ptafr	82.7972462912419	0.221122969109278	0.445427379645714	0.496428776527289	0.61959192641106	0.90471246603136
Aqr	552.116714075164	0.0542070555158512	0.109286626293412	0.496008133422624	0.619888672545188	0.904797053056197
Cdkn1a	275.184074572984	-0.156344253122389	0.315197357569424	-0.496020189788403	0.619880166420769	0.904797053056197
Gpr89b	783.376781590845	-0.0494562179257843	0.0997007148454428	-0.496046773610921	0.619861410923547	0.904797053056197
LOC691909	83.2204764207675	-0.130358366781792	0.263059268860435	-0.495547514240803	0.620213691156803	0.904982668442283
Tmem256	1864.90441945788	0.0910270750494077	0.183688179664018	0.495552164629777	0.620210409413775	0.904982668442283
Trmt61a	217.635902863286	0.108420321198731	0.218814614868106	0.495489395276834	0.620254705891728	0.904982668442283
Ddr2	125.656704252663	-0.174021473612236	0.352257353908508	-0.494018000423164	0.62129346703987	0.905126381875908
Ebag9	947.398861102679	-0.0762702557522611	0.154345880293612	-0.494151548503741	0.621199154860982	0.905126381875908
Hsd17b14	37.1264296443154	0.2025121119596	0.409800699125647	0.494172197342955	0.621184573125161	0.905126381875908
Lyrm9	224.302869960348	0.0850060879870694	0.172000143100409	0.494221030603709	0.621150088790464	0.905126381875908
Pigm	329.448253983753	0.0741129118383219	0.149947384115968	0.494259451575386	0.621122957833827	0.905126381875908
Prdx1	19125.0876494458	0.111487336736846	0.225204579919254	0.495049153870758	0.620565423982553	0.905126381875908
Rev3l	510.245528824726	-0.0645459768152168	0.130493182866283	-0.494631025142189	0.620860597870385	0.905126381875908
Riok2	343.720408188727	0.0736523927448982	0.148888064496078	0.494682988822373	0.620823911295303	0.905126381875908
Slc2a6	59.8057095024788	-0.137645518840786	0.278510836173154	-0.494219617204445	0.621151086871719	0.905126381875908
Smn1	297.668115772471	0.0817858757553306	0.165559705295355	0.49399626321771	0.621308818528579	0.905126381875908
Trappc4	541.498497551379	0.0746965244832188	0.150947255147894	0.494851823638882	0.620704719720237	0.905126381875908
Yod1	19.516632022216	-0.245067353923032	0.495569804857745	-0.494516315402589	0.620941586763521	0.905126381875908
Pik3r4	700.48593297854	0.047787263335479	0.0968756915335574	0.493284358325593	0.621811678918717	0.905742860770234
Chn2	149.374841041577	0.0939769945547264	0.190602466443382	0.493052352932915	0.621975596172085	0.90584534006075
Fam25a	32.6677384740369	-0.220674262133121	0.447652199403063	-0.492959182211963	0.622041428750145	0.90584534006075
Arl6ip5	424.675406074218	0.0668526465787203	0.135828526677351	0.492184139915785	0.622589175372105	0.906052235583054
Deaf1	621.896779802359	-0.0661856948032308	0.134493197086322	-0.492111840874381	0.622640282025668	0.906052235583054
Kif18a	31.2044873753732	0.205136547533413	0.416683544932069	0.492307771757235	0.622501786868605	0.906052235583054
LOC690871	592.793750375332	0.10258761419129	0.208260537950987	0.492592668782183	0.622300429187604	0.906052235583054
Rnd2	276.213713904508	0.086253089917073	0.175282157848222	0.49208140164363	0.622661799413974	0.906052235583054
Srp9	1144.41065251276	-0.0924419667397343	0.18782427227383	-0.492172633603834	0.622597308817448	0.906052235583054
Atg16l1	473.206687580523	-0.0615972824174164	0.12549808328309	-0.490822495499547	0.623551998003086	0.906099670984467
Csrp2bp	591.522993189523	0.110103302154443	0.224122027038399	0.491264975644623	0.623239048370418	0.906099670984467
Cul2	844.764193062646	0.0762364819778377	0.155644434540223	0.489811808581798	0.624267073877752	0.906099670984467
Eva1b	306.825141342694	-0.130769561355661	0.266053626062693	-0.491515801873893	0.623061678578336	0.906099670984467
Fam104a	768.643013176067	-0.0655652158954009	0.133843885161303	-0.489863364444213	0.624230588770983	0.906099670984467
Fkbp2	892.15874247105	0.0674317451253871	0.137710712647987	0.489662306067319	0.624372879183386	0.906099670984467
Haus4	219.821226209527	0.124947353114961	0.254830176575464	0.490316157976526	0.623910194960515	0.906099670984467
Psmc3ip	248.638406291051	0.0969317863851523	0.197162061202963	0.491635083310315	0.622977337321362	0.906099670984467
Ptp4a3	128.587524514662	0.182282293911247	0.371332945203307	0.490886403336624	0.623506794182848	0.906099670984467
RGD1307315	237.306007585851	-0.0923237058098639	0.188508670307198	-0.489758405591696	0.624304867133242	0.906099670984467
Rhov	17.5212227292471	-0.276910954036028	0.564379166516346	-0.490647016163393	0.623676126791065	0.906099670984467
Rnf128	1039.60628153625	-0.0813019395042693	0.16534751832412	-0.491703415499097	0.622929023375045	0.906099670984467
Scpep1	3823.19951505456	0.0863674088072519	0.175646116675337	0.491712600551784	0.62292252925136	0.906099670984467
Sec23b	911.669686290134	0.0557637079625366	0.113736535315303	0.490288435531705	0.62392980914188	0.906099670984467
Slc25a46	972.55773651842	0.0612215537011568	0.124646925809574	0.491159756275791	0.623313459926279	0.906099670984467
Surf4	3582.31911259354	0.0458062178784824	0.0932367451200345	0.491289328252619	0.623221826657344	0.906099670984467
Timm17b	451.010786077569	0.0834933764568828	0.170551091568173	0.489550525236648	0.624451993314769	0.906099670984467
Twistnb	318.824673757463	0.0772001649450261	0.157730592552072	0.489443193586811	0.62452796256311	0.906099670984467
Ube2d2	693.200311044745	-0.0554114175509337	0.113049683296559	-0.490151019756288	0.624027037473161	0.906099670984467
Unc45a	1641.09824991981	-0.0577634754955402	0.117642986612787	-0.491006537309905	0.623421823865099	0.906099670984467
Vmp1	2460.92308941514	0.0946337338294736	0.192952879113805	0.490449970293824	0.623815523832719	0.906099670984467
Wwox	128.769111813578	0.111664780802369	0.227686671222002	0.490431785941006	0.62382838874991	0.906099670984467
Xrcc2	32.7069549389524	-0.196462183689821	0.400887487556697	-0.490068135793427	0.624085685066492	0.906099670984467
Aff3	35.4010361336082	0.251545949752067	0.514601946446586	0.488816553238935	0.624971577703926	0.90616777989171
Lyc2	18.4571330650929	-0.915900796622235	1.87339946790726	-0.488897756358057	0.624914084218324	0.90616777989171
Prpsap1	1706.35205931293	-0.0628862316168593	0.128650691730045	-0.488813785384201	0.62497353744247	0.90616777989171
Spcs1	1443.84234551215	-0.0686789479261937	0.140397805295281	-0.489173942439841	0.624718555852654	0.90616777989171
Timp3	3221.28602924044	0.130797748153034	0.267561258268141	0.488851596078057	0.624946766370072	0.90616777989171
Cog3	218.647004746252	-0.0750452355316355	0.153653674076418	-0.48840508359281	0.625262941786926	0.906170755329044
Commd8	907.181100648666	-0.0754242568187672	0.154515036254786	-0.488135385700567	0.625453948227727	0.906170755329044
Pank3	998.985274448724	-0.0755880231646791	0.154843560478955	-0.488157356566032	0.625438386998945	0.906170755329044
Pcp4l1	37.5392737709777	0.184083604554707	0.376911591613499	0.488399955455534	0.625266573420713	0.906170755329044
Rnf6	943.645583083973	0.0884707031668855	0.181131681128211	0.488433070437099	0.625243122280953	0.906170755329044
Srp14	1371.98478059906	0.054162793052405	0.110917190614985	0.488317390226866	0.625325045547875	0.906170755329044
Cdkn1b	1047.99035786767	-0.0636585924175408	0.130607531462655	-0.487403687250171	0.625972281047977	0.906324597843276
Crkl	228.774151751441	0.086188694352033	0.176740945563622	0.487655500977317	0.625793876140336	0.906324597843276
Fis1	2202.48795587809	0.070477706337089	0.144625983635742	0.487310126198315	0.626038572731617	0.906324597843276
LOC679462	87.0300193599284	0.158401566084576	0.324783653869191	0.487714095821995	0.625752366025277	0.906324597843276
Psmd5	952.364950875725	0.0673873256022384	0.13820079812941	0.487604460425313	0.625830035564196	0.906324597843276
Romo1	660.483767140604	0.0790799477209005	0.162271493821672	0.487331113176324	0.626023702369614	0.906324597843276
Ppa2	2055.09237794278	0.0964338455227406	0.197961181575644	0.487135127984129	0.626162573959946	0.906388667298617
Kif23	74.3794980349521	0.160741876305966	0.330335536205466	0.486601829619644	0.626540526726387	0.90682027591648
Bbs7	665.939501298541	0.0922733166419583	0.189828180016442	0.486088612522999	0.626904340362542	0.907124442449157
Entpd6	114.176129843301	-0.117129327571361	0.241070223738038	-0.485872231564529	0.627057757517064	0.907124442449157
Snrpd2	1550.49689781505	-0.066292015745963	0.136444012327527	-0.485855074291074	0.627069922956451	0.907124442449157
Tmco4	412.268023222099	0.0854044527402711	0.175767385994708	0.485894765157641	0.627041780128894	0.907124442449157
Exnef	683.173137566949	-0.114534676181697	0.235849687770992	-0.485625727403587	0.627232552101861	0.907128793382078
Nme7	1524.10027603097	-0.0913039543061434	0.187993252189605	-0.485676763621583	0.627196360915495	0.907128793382078
Dhodh	138.673335644873	-0.0943858698466553	0.194601890339958	-0.485020313429478	0.627661936219474	0.907172630604087
Erp44	2240.83125868681	0.0710211940077894	0.146410391631485	0.485083013687648	0.627617460800487	0.907172630604087
Fam210b	95.3350988896387	0.138251499055694	0.284845640653535	0.485355853572122	0.627423941971538	0.907172630604087
Ggn	31.7475971669345	-0.191394787164218	0.394336409633322	-0.485359156518639	0.627421599427235	0.907172630604087
LOC499781	90.9468737830386	-0.145960441208629	0.30081749088991	-0.485212614388989	0.62752553491668	0.907172630604087
Sapcd1	33.8922226737588	-0.221565529553368	0.457028215520671	-0.484796172378437	0.627820938125473	0.907287067099063
Dnajc7	1708.55074016225	0.0512227563676903	0.105748661444336	0.48438207792023	0.628114735239126	0.907596247232127
Glb1l2	908.43911259142	0.101055564509562	0.208700824611766	0.484212578927511	0.628235010586943	0.907654649845073
Hiat1	495.001185515942	-0.0802893728034076	0.165882617049509	-0.48401317890617	0.628376516141463	0.907735344561518
Ndufv2	4493.44121178485	-0.0593419393289502	0.122630418819356	-0.483908804196172	0.62845059179611	0.907735344561518
Alg10	80.2393764058914	0.134188927354139	0.277509759385051	0.483546696345007	0.628707611942855	0.907760507792491
Cybrd1	65.4819408058999	-0.136386176695442	0.281975075494891	-0.483681674545425	0.628611800646542	0.907760507792491
Mrto4	515.724463729406	0.0744947068431923	0.154054959428455	0.483559290266074	0.628698672161417	0.907760507792491

Il2rg	72.49379880699	-0.220688336159727	0.456700151692365	-0.483223697960109	0.62893691060206	0.907860925311533
Zfyve9	524.568154909096	0.0903005403634384	0.186841095450804	0.483301278798245	0.628881832192613	0.907860925311533
Aup1	2530.82277043136	-0.0549570790858938	0.113785328238376	-0.482989151033259	0.629103439427345	0.907870707533164
Nol10	253.165740787902	-0.097516534713926	0.201887249173143	-0.483024733425803	0.629078174637561	0.907870707533164
Mrps24	796.935957663382	0.0688371736972519	0.142633504122424	0.482615736890039	0.629368603201349	0.908138065759366
Tusc2	1162.25052545093	0.0777266257614432	0.16109073289006	0.482502154946984	0.629449267946798	0.908139169774474
Eif3j	1209.49067710231	0.0553550108693243	0.114751733404307	0.482389321948549	0.629529405176573	0.908139512531657
Map2k4	241.952244201812	0.077165221903696	0.160043447031834	0.482151711518354	0.629698177178753	0.90826770073778
Anxa3	283.768820166187	-0.143271107142665	0.297983041719151	-0.480802888366037	0.630656597227545	0.908390413679344
Cyp2c24	705.628670533384	0.696814653482632	1.44899011256942	0.480896762122831	0.630589874126422	0.908390413679344
Dyrk2	541.7114003692	-0.0743506609549775	0.154694315973738	-0.480629559573475	0.630779802879734	0.908390413679344
Eea1	1153.32874727366	0.0813938103268378	0.169719149936343	0.479579413150293	0.631526487825758	0.908390413679344
Fam172a	539.56647412119	-0.0760899782979201	0.15843345214169	-0.480264598601762	0.6310392581623	0.908390413679344
Fam98c	389.497596225216	-0.0891311203563175	0.185701068704081	-0.479970960739866	0.631248041979516	0.908390413679344
Gabbr2	23.0400711011704	-0.34641294067768	0.72037704132754	-0.480877263993999	0.630603732657306	0.908390413679344
Gtf2ird1	330.399072361385	0.0667098373243283	0.138512769823414	0.481615069927307	0.630079418773257	0.908390413679344
Iws1	363.646074325392	0.0785208205534003	0.163426710972798	0.480465035892876	0.630896759173642	0.908390413679344
Me3	411.931491871104	-0.0930213367337333	0.193618693610263	-0.48043572136158	0.630917599204674	0.908390413679344
Naa30	782.203176463275	-0.0722601442012703	0.150564219174527	-0.479929060154123	0.631277836736784	0.908390413679344
Pcyt2	2205.18909381525	0.0676813394536138	0.141120085069158	0.479601039217382	0.631511107261494	0.908390413679344
Ppie	234.01256570476	-0.0821500477585236	0.170537801047545	-0.481711663067711	0.630010789677849	0.908390413679344
Rabl3	257.421940587185	0.0853342407518316	0.177575902579292	0.480550792716527	0.630835795365543	0.908390413679344
RGD1306954	951.200080026515	0.0731973475364212	0.152132364413198	0.481142509148243	0.630415217597172	0.908390413679344
RGD1307704	190.451784596791	0.108971732403142	0.227043091947454	0.479960572543482	0.631255428784345	0.908390413679344
RT1-DMa	75.4635048595454	-0.201135077648266	0.418732695595337	-0.480342423135361	0.630983927919825	0.908390413679344
Senp8	60.682082967725	-0.158266875114204	0.329812383746955	-0.479869413380281	0.631320251524604	0.908390413679344
Smad1	546.577958477798	-0.0675392041957018	0.140339555023227	-0.481255653009046	0.630334811301091	0.908390413679344
Tinf2	300.797303659201	-0.0675871853177408	0.140936338393648	-0.479558260758583	0.631541531664102	0.908390413679344
Trim13	63.3462004081578	0.131756015103545	0.273635455351624	0.481501985677394	0.630159768802366	0.908390413679344
Vwa7	205.782337019507	0.0985226956670045	0.204501762976922	0.481769419651031	0.62996975534871	0.908390413679344
RGD1566036	160.851588658874	0.0849964291652687	0.177306988443025	0.479374388520401	0.631672310265369	0.908463555418978
Wfs1	177.348793027075	0.125369042029903	0.261666489733429	0.479117682044888	0.63185491128966	0.908611199610106
Atp5g2	7209.56972574752	0.0665637738028862	0.13904087918615	0.478735276938013	0.632126966191837	0.908806473833809
Osbpl5	261.574791305659	-0.162875847312904	0.340244740761837	-0.478702027688102	0.632150623097844	0.908806473833809
Naca	1312.0462463386	-0.0760440161700815	0.158926382941548	-0.478485791739499	0.632304484582409	0.908912706685679
Bid	126.948993992725	0.115649621413875	0.242021198434445	0.477849139505028	0.632757583351705	0.908989217944323
Cuedc2	312.378961192575	-0.0755674458491963	0.158062441077689	-0.478086035708218	0.632588970676461	0.908989217944323
Gtf2f2	224.591497205241	-0.0894449048745905	0.18715593908756	-0.477916465331855	0.632709661740285	0.908989217944323
Thyn1	341.317074237535	-0.0724323797860658	0.151553975790548	-0.477931241382738	0.632699144553781	0.908989217944323
Usb1	143.009582509717	0.110705531839906	0.231590087526727	0.478023619327531	0.632633394157866	0.908989217944323
MGC95208	275.682531294013	0.0813943951825148	0.170432219142406	0.477576338512056	0.632951775180813	0.909040369575043
Pabpc1	4387.94545417648	0.0426251552760226	0.0892744332174022	0.477462065451833	0.633033127325925	0.909040369575043
RGD1565192	1417.0061067724	0.0581707161769972	0.121819063680588	0.477517347609252	0.632993770845694	0.909040369575043
Trmt5	201.80679310483	0.0967420419888881	0.202737823319261	0.477178063791994	0.633235330196938	0.909215861927539
Polr2h	171.011322787448	-0.101753853454951	0.213313738880059	-0.477015001420816	0.633351439366874	0.909267709971439
Fuz	313.674768832181	-0.0715558207160437	0.150127123827247	-0.476634860455887	0.633622155225748	0.909541477178057
Adprhl2	787.676222226391	0.0555500572440257	0.11672448928787	0.475907477367722	0.634140294875334	0.909821064145458
Cntfr	13.6904325399205	0.374066046559011	0.786128169209738	0.475833408863905	0.634193066450684	0.909821064145458
Per3	348.484863078388	-0.324706346743244	0.682064300954033	-0.476064128102091	0.63402869207863	0.909821064145458
RGD1311899	1522.44011059039	-0.0601371152630991	0.126378188056715	-0.47585043105786	0.634180938486202	0.909821064145458
Src	219.539457974125	0.105196499720608	0.22109413857703	0.475799586536563	0.634217164457546	0.909821064145458
LOC683897	527.512131342234	0.0719107648769139	0.151288153110248	0.475323172360434	0.634556645097785	0.909855599230937
Notch3	253.94095199397	-0.159145372794142	0.334898975740579	-0.475204119218984	0.634641491351651	0.909855599230937
Pcdhgb8	48.8701521399663	-0.203892078795748	0.428835413220681	-0.475455320409428	0.634462472046925	0.909855599230937
Phka2	33.4932077792716	-0.179100375608383	0.376707315731129	-0.475436414768791	0.634475944466277	0.909855599230937
Ybx1-ps3	352.851660652462	-0.895271246009645	1.88376811851387	-0.475255546163471	0.634604840040697	0.909855599230937
Alkbh4	77.3984799644544	-0.131880027867888	0.277737652388292	-0.474836691150224	0.634903378588801	0.910116256910117
Bcap31	2436.12361272482	-0.0628642263899272	0.132615279248236	-0.474034566350797	0.635475258057253	0.910244605654645
Eif4e3	50.3795648994197	-0.201094283726593	0.42455680635397	-0.473656954068315	0.635744554206883	0.910244605654645
Erh	1130.67353054829	-0.0695741087816049	0.146672909901122	-0.474348731667678	0.635251246170028	0.910244605654645
LOC690617	59.5798267885243	-0.130583335019441	0.275595912360641	-0.473821741044408	0.635627029598164	0.910244605654645
Nit2	7384.97986116629	0.0868203841918097	0.183324729238915	0.473587958112636	0.635793764234755	0.910244605654645
Orai3	369.16923964752	0.0654148782170128	0.138027930316203	0.473924937272887	0.635553435636272	0.910244605654645
Ppp4c	927.839908630642	-0.0645291244392134	0.136213464885874	-0.473735283756853	0.63568868902422	0.910244605654645
Sgk196	496.360095065107	-0.0683711518916045	0.144342998821996	-0.473671410803373	0.635734243424629	0.910244605654645
Stk25	1933.40614947734	-0.052921579832922	0.111597585744224	-0.474217963408416	0.635344484886042	0.910244605654645
Tspan33	1737.31336981719	0.065451582097261	0.138183621012175	0.473656585475455	0.635744817094322	0.910244605654645
Rad54l	31.5785934973884	0.191932971844779	0.405416103636917	0.473422170759825	0.635912015423892	0.910299240215108
Msantd4	748.685652031721	-0.0679899497372307	0.143666706810941	-0.473247777765953	0.636036414777642	0.910362660919617
Ammecr1l	1472.37668807408	-0.0710149346563212	0.15013086367025	-0.473020223291992	0.636198751126466	0.91043398523743
Gas5	511.144235921146	-0.0957722007969664	0.202498190080955	-0.47295336693468	0.636246449475709	0.91043398523743
Bri3	3236.95248537255	0.0870199306551411	0.184207344486758	0.47240206897069	0.636639827859572	0.910570850997371
Iffo2	20.8220699877338	0.269157944615369	0.56961199860745	0.472528572560601	0.636549552225251	0.910570850997371
Msl3	902.666478907749	-0.0544607067156904	0.115258584817642	-0.472508896424992	0.63656359317695	0.910570850997371
Ppp1r3d	94.2096911636751	-0.153686806444018	0.325429776212697	-0.472257972926149	0.636742664413787	0.910570850997371
Ranbp17	17.9399890083647	-0.235926444177682	0.49949710566645	-0.472327950455087	0.636692722904735	0.910570850997371
Commd1	1106.23501689138	0.0608565742627803	0.128898891192053	0.472126437240697	0.636836543167407	0.910590533355233
Kctd11	69.2258731007432	-0.134952438952176	0.286390025311929	-0.471219061506031	0.637484307959112	0.911373064379042
Lrrc16a	506.068959678732	-0.0775896055668795	0.164686495633597	-0.471135203092213	0.637544187478248	0.911373064379042
Vprbp	554.947375924036	0.0521153597509718	0.11064960510225	0.470994539047947	0.637644634653795	0.911402026845452
Eef1d	2194.0655543009	-0.0656657079967098	0.139630855972541	-0.47028078098743	0.638154426644632	0.912015995629686
RGD1309534	24902.5139381525	0.087831604566334	0.186824345336458	0.470129331421745	0.638262619455253	0.912055938235907
Mrps18b	1446.49045492378	0.0808132881064288	0.171945778261409	0.469992860095515	0.638360118683074	0.912080591948689
LOC100360619	16.8579053849355	-0.251983787694814	0.536467986600432	-0.469708899671016	0.638563008609611	0.912255801389748
Fdx1	891.241594439014	0.101515561715208	0.216703886802169	0.468452888470256	0.639460753928019	0.913005123257033
Nos1	15.6671897769332	-0.298130165521143	0.636773827170648	-0.468188472578738	0.639649814930121	0.913005123257033
Rassf8	188.539632860104	-0.127832425865693	0.272971399426025	-0.468299705150375	0.639570279257525	0.913005123257033
Rrp9	282.630687571707	0.103512790550522	0.220842801114761	0.46871706946305	0.639271884267393	0.913005123257033
Upp1	192.46921174451	-0.198595853363897	0.424055746718817	-0.468324872143713	0.639552284440106	0.913005123257033
Ust5r	1906.02731780284	-0.115525075383864	0.246563961650648	-0.468539986989458	0.639398482414544	0.913005123257033
Zfp167	124.07349795985	0.126095616455468	0.269004604827564	0.468748914303149	0.639249119166492	0.913005123257033
Eid1	2598.82977014151	-0.0564493093085185	0.120631102291068	-0.467949875582776	0.639820435156615	0.913074095994455
Tmem101	410.039432781613	-0.0584075838207963	0.124830210134442	-0.467896222860566	0.639858804733049	0.913074095994455
Dolpp1	333.289432605001	0.0858596847353499	0.183576777913151	0.467704497874833	0.639995924127249	0.913155118457233
Map3k7	452.583786038448	-0.0665923690032103	0.142416186298957	-0.467589890824775	0.640077895577499	0.91315744460071
Atox1	1449.06092107844	0.0752849447859694	0.161107940950886	0.467295059086629	0.64028879096681	0.913229062382829
Rpl27	5555.6024689136	-0.0769319085199973	0.164607256595731	-0.467366446115673	0.640237724614244	0.913229062382829
Pafah1b2	1037.83098162033	-0.0517836393904257	0.110843268490422	-0.467178928370379	0.640371868130893	0.913232955229076
Adh5	1838.81988174831	0.0608077538886023	0.130269580996498	0.466783983056158	0.640654436380402	0.913292151498764
Enpp3	1021.64300324484	0.0661281426086717	0.141654067318801	0.466828407121177	0.640622650061851	0.913292151498764
Nxt2	231.044678233175	0.0987857848065358	0.211621332249494	0.466804474560583	0.640639774211927	0.913292151498764
RGD1309779	278.65048184673	0.0881115393928297	0.18882448932229	0.466631948583952	0.640763225113164	0.913332683300254
Mcts1	637.420194528318	-0.0843992013588315	0.180917141561469	-0.466507488623767	0.640852288564754	0.913333389318217
RGD1311739	180.592115837601	-0.128503261398245	0.275517628643793	-0.466406676156401	0.64092443367958	0.913333389318217
LOC679504	16.8831193894459	-0.304761783866781	0.653593045965849	-0.466286760160396	0.641010254400548	0.913341174817178

Anks6	1171.40645970965	-0.0446494863255065	0.0958891547380782	-0.465636457506241	0.641475742438604	0.913757121023016
Bik	16.3499225565454	0.283330425640042	0.608665905110647	0.465494162332842	0.641577616433333	0.913757121023016
Clip3	26.2102593492406	-0.187516637679344	0.402929835913996	-0.465382855687482	0.641657309381913	0.913757121023016
Gorasp2	1190.46684030968	0.0487920369337701	0.104829234230132	0.465443034971112	0.641614221913027	0.913757121023016
Usp36	353.266527470653	-0.0980543936935984	0.210725808404413	-0.465317439928468	0.641704147457832	0.913757121023016
Rnf208	157.065914451807	0.124467276020218	0.26765152192543	0.465034815138828	0.641906524887201	0.913930798179623
Stx11	14.2996100148484	0.255227957594924	0.548982493268782	0.464910922887963	0.641995248051241	0.913942633936674
Mrpl42	774.861027570769	0.0769486159211338	0.165590862395472	0.46469119616855	0.642152613881371	0.914052173998204
Naf1	113.074583757721	-0.115944361590784	0.249659983934697	-0.464409072545286	0.642354691239744	0.914080702444341
Nek8	401.116089614428	0.0606479284745387	0.130614865877523	0.464326384803763	0.642413923203009	0.914080702444341
Polr2l	791.890876449065	0.0747685496703517	0.160953213518438	0.46453592342713	0.642263828180101	0.914080702444341
Mast2	1262.01909143527	0.0468521479911884	0.1009955880557	0.463902917871513	0.642717302208084	0.914209086683774
Mok	80.6916051751081	-0.110192531439972	0.237553721663848	-0.463863629111652	0.642745452374629	0.914209086683774
Wbp11	525.358384876698	0.0526025022450418	0.113367947097998	0.463998013473515	0.642649168889566	0.914209086683774
Ccz1	917.615460342826	0.0584996283784231	0.126163586969962	0.463680763866924	0.642876481002819	0.914281041426181
Trpt1	317.671959117536	-0.0784696081999325	0.169280920248171	-0.463546677823428	0.642972564878519	0.914303286927217
Rab34	1020.7601774553	0.0700305753719121	0.151321250802864	0.462794055695095	0.643511992444738	0.91484143916534
Ripk3	112.434504799193	-0.110089538230821	0.237869154498913	-0.46281552756486	0.643496600286026	0.91484143916534
Anks4b	1664.1154036251	0.0856819682641773	0.185384582715987	0.462184972498192	0.643948678806848	0.915094040181319
Calml3	18.1993163191675	-0.285155690043665	0.61666216990903	-0.462418004473554	0.64378159041082	0.915094040181319
Dnajc17	290.711233406685	-0.082303732056766	0.178367960181075	-0.461426659660249	0.644492528189331	0.915094040181319
Eif1	7649.9716264652	-0.0825619260366232	0.178796523425975	-0.461764716978994	0.644250055564416	0.915094040181319
Fau	4708.76448804441	-0.0740515512872568	0.160258773462726	-0.462074866088252	0.644027633475804	0.915094040181319
Kcnj5	13.783958873782	-0.424288424653165	0.91952061319053	-0.461423505429616	0.644494790748852	0.915094040181319
Nfix	59.6894013313152	0.212034877666	0.459304433615376	0.461643437658515	0.644337039168173	0.915094040181319
Nov	36.2563443410272	-0.241730231159422	0.522974800512065	-0.462221565786218	0.643922439526115	0.915094040181319
Nudt19	470.853901349085	0.096277321969366	0.208415922089145	0.461948017235389	0.644118598729262	0.915094040181319
Trmu	227.439077078195	0.0725876972194606	0.157274865207608	0.461533997334173	0.644415535821604	0.915094040181319
Armcx1	78.1469467826883	-0.143724988059044	0.312971075276856	-0.459227703173221	0.646070658589487	0.915648421757242
Bysl	382.252786335233	0.0816542669168268	0.177452551032027	0.460147044615266	0.645410678301045	0.915648421757242
Carkd	564.389448240615	-0.0674885608414206	0.147276082520656	-0.458245220040771	0.646776275323631	0.915648421757242
Etnk2	147.341891180589	0.114716885864101	0.250464079595467	0.458017317490732	0.646939999742459	0.915648421757242
Fkbp5	1592.74800523238	-0.336835398758583	0.731142588456355	-0.46069727584839	0.645015809733818	0.915648421757242
Fmo2	578.653345591722	-0.145218562380469	0.315730146394429	-0.459945190659914	0.645555562076649	0.915648421757242
Git1	1391.38989541643	-0.050369735962255	0.109383123349519	-0.460489099413493	0.64516519394245	0.915648421757242
Grsf1	2681.21949114446	0.0601906569941203	0.13139091034387	0.458103660569763	0.646877969156055	0.915648421757242
Idnk	728.768812313045	-0.0697110018801645	0.15195304009124	-0.458766746873288	0.646401676557691	0.915648421757242
Mppe1	482.2564071966	-0.0732155963514423	0.159891816199099	-0.457907090505956	0.64701919256692	0.915648421757242
Nae1	430.222926919587	0.0738455544594473	0.161096647158428	0.458392870131091	0.646670213125908	0.915648421757242
Ppp4r1	1277.78984264089	0.0415132140894872	0.0906699436436263	0.4578497837459	0.647060366316573	0.915648421757242
Ppp5c	2501.81245663735	-0.0639215932469991	0.138975530728872	-0.459948545702654	0.645553153833263	0.915648421757242
RGD1311745	270.745393259938	-0.0682577929200997	0.149006885851997	-0.458084822924878	0.646891502285814	0.915648421757242
S1pr1	356.204515285817	-0.153718615610422	0.334888303890739	-0.459014584339066	0.646223692946299	0.915648421757242
Sft2d1	1026.61895158068	-0.0620784940972125	0.135470738995857	-0.458242824667184	0.646777996062993	0.915648421757242
Sparcl1	312.200708638354	-0.332170903156714	0.72284797800036	-0.459530791073954	0.645853045892618	0.915648421757242
Tagln	806.483714151706	-0.332190469092319	0.722954117931427	-0.459490389297192	0.645882052029358	0.915648421757242
Thoc5	644.235837628015	0.0542774618812763	0.118506270274026	0.458013417819739	0.646942801401615	0.915648421757242
Timm8a2	15.9702614351444	0.236825477848033	0.516228956769811	0.458760545572484	0.646406130259792	0.915648421757242
Tmod1	180.424032694448	-0.110947060512729	0.242224944815961	-0.458033174894622	0.646928607283998	0.915648421757242
Trim27	524.691725016745	0.0548057391037736	0.119488597548603	0.458669197129718	0.646471737116682	0.915648421757242
Vrk2	21.2997203869058	-0.276648393630394	0.603359513425389	-0.458513353108179	0.646583671329468	0.915648421757242
Wdr81	1097.34278788573	-0.0692458477190039	0.150836609000049	-0.459078523297891	0.646177778691183	0.915648421757242
Zfp51	93.2807278281168	-0.113963828364087	0.247938744783392	-0.459645096871206	0.645770983864897	0.915648421757242
Zfp516	27.9050552481897	-0.244940932992986	0.533752000308696	-0.458904009448816	0.646303099417012	0.915648421757242
Zmat3	203.666503054115	0.105403862139508	0.229034851864308	0.460208834077159	0.645366330655927	0.915648421757242
Stambp	373.552821838302	0.0627304842119602	0.137055142695509	0.457702520155166	0.647166177187007	0.915684149123307
Dtnbp1	885.313104019223	0.0712321008155518	0.155980223646489	0.45667392410586	0.647905436066202	0.916025494636319
Grb2	1857.06713719102	0.0529395264517396	0.115959324403253	0.456535312914039	0.648005083429878	0.916025494636319
Gskip	255.933770904172	-0.0915563838495902	0.200539112313933	-0.456551257224394	0.64799362076972	0.916025494636319
Hcr	433.511777186578	0.0742729933789014	0.162595012946382	0.456797487407526	0.647816611924129	0.916025494636319
Phlpp1	323.501568026373	0.0638224813567062	0.139817529065957	0.456469813070427	0.648052173356558	0.916025494636319
Prim1	317.231590717067	-0.0939055724452641	0.20543348250778	-0.45710938304182	0.647592426375839	0.916025494636319
RGD1307947	148.60060006078	0.101033201382987	0.221310661415887	0.456522070543746	0.648014603678686	0.916025494636319
Slc25a32	187.719451410937	0.111160060057419	0.243323868562259	0.456839934011557	0.647786100112037	0.916025494636319
Epc2	361.841084638731	-0.0712486122918493	0.156251301228213	-0.455987321269006	0.6483990954762	0.916174057581115
Pold1	118.388568606707	0.140498709091329	0.308117454072976	0.455990750391091	0.648396629593286	0.916174057581115
Usmg5	2092.22036749591	-0.0707447995593138	0.155071452066298	-0.456207758531003	0.648240586806363	0.916174057581115
Cks1b	163.980772496699	0.0933052703814279	0.204789978739905	0.455614434629787	0.648667261392532	0.91643904201212
Npff	21.113437781718	-0.222093188701821	0.487624976226645	-0.45545900954547	0.648779050668732	0.916483058650362
Aff4	1362.81099482655	-0.0512358871412773	0.112659519707372	-0.454785243842334	0.649263747199951	0.917053777889231
Cnot2	535.823739349363	0.0674210872197263	0.148369231930921	0.454414209349799	0.649530727057903	0.917062165255855
Hells	23.8954421227916	-0.201678943674731	0.443979833240559	-0.454252487557146	0.64964710892944	0.917062165255855
Meis3	140.543098212134	-0.11654207711957	0.256347934848056	-0.454624599135732	0.64937933443178	0.917062165255855
Rdh14	381.328287365947	-0.0658387729491672	0.144876393373389	-0.454447901525828	0.649506481813275	0.917062165255855
Tbcd	917.162657287291	0.0513840494903093	0.11312681352029	0.454216360306951	0.649673108801702	0.917062165255855
LOC317165	122.992137589419	-0.117452878343363	0.258884134215929	-0.453688978272414	0.650052700931754	0.917484043063494
Uspl1	266.52817278806	-0.0833004766933634	0.183704022654223	-0.453449388259481	0.650225179903579	0.917499614498333
Zp2	9.53523325386796	0.393378445809407	0.867387760526685	0.453520863114952	0.650173723753835	0.917499614498333
Champ1	541.26505820108	-0.054160334913596	0.119537511795565	-0.45308233457479	0.650489455316336	0.917758583555793
Arl8b	1751.75206509142	0.0421963163764981	0.0931890513165467	0.452803368854616	0.650690337571021	0.917814144555998
Sap18	1561.45537977995	0.0702745008459469	0.155166205375207	0.452898236932561	0.650622020546563	0.917814144555998
Abca5	39.9508207247633	0.21485320498296	0.476639114132326	0.450767044945689	0.652157455303904	0.91783328838338
Arl2bp	2851.67292425922	0.0560759054316057	0.123913937385911	0.452539130097738	0.650880638392595	0.91783328838338
Ash2l	600.799265479774	0.0527835874918885	0.116995543693626	0.451158957217308	0.651874987988576	0.91783328838338
Cenpn	192.49780417119	0.0769926461820545	0.170260496265525	0.452204991003803	0.65112131297926	0.91783328838338
Ctps2	938.238234725222	0.0406098908718446	0.0900228348101488	0.451106554881189	0.651912753618897	0.91783328838338
Gale	154.262306597617	0.0901172061938229	0.199848220037992	0.450928240325039	0.652041269111221	0.91783328838338
Hspa12b	124.69944320593	-0.14592138706776	0.322819120949316	-0.452022131274779	0.651253039065327	0.91783328838338
Pttg1ip	15714.1987737721	0.0712217222309872	0.15765030188195	0.451770287660589	0.651434476679209	0.91783328838338
RGD1303142	501.539726812943	-0.0770023570731646	0.170428067502554	-0.451817345590747	0.651400572807411	0.91783328838338
Rps19bp1	221.579248439214	0.078824774774062	0.174779700712239	0.450995020891133	0.651993137576849	0.91783328838338
Setd6	359.434847859232	0.0839088622985929	0.185961816337026	0.451215544951022	0.651834207003434	0.91783328838338
Stx18	880.645772020563	0.0473253725843551	0.104972844807991	0.450834429331884	0.652108885047901	0.91783328838338
Txn2	4696.99387527159	-0.108656728023851	0.240953828068318	-0.450944186672325	0.652029775783075	0.91783328838338
Ube2q2l	28.1211051483523	0.215689300561526	0.477451697248352	0.45175103953884	0.651448344599543	0.91783328838338
Usp24	2525.7360968677	0.0495587661319174	0.109912409832035	0.450893272266996	0.652066472624728	0.91783328838338
Zbed3	306.939427354989	0.0835148364665812	0.184544915284513	0.452544771216407	0.650876575505084	0.91783328838338
Zfp14	97.7844994963248	-0.147004785882913	0.325225749903712	-0.45200844621447	0.651262897767024	0.91783328838338
Zfp207	1150.70332358556	0.0553221159483736	0.122358415386255	0.452131680307689	0.65117412226629	0.91783328838338
Cdk10	329.142965289256	-0.081105209091075	0.179986751055426	-0.450617662775074	0.652265134324824	0.917859872483584
Dbndd2	433.660590424827	-0.0637088690124959	0.141649700838776	-0.44976352675117	0.652880959611016	0.917859872483584
Mlh1	393.497918948269	0.0806174040872692	0.179229550033591	0.4497997348772	0.652854849034592	0.917859872483584
Mlx	1679.64300485253	0.0711944840985623	0.158303972023064	0.449732771633739	0.65290313822187	0.917859872483584
Sdf2l1	410.621110596886	0.0879563732306365	0.195565295210791	0.44975450851764	0.652887462948707	0.917859872483584
Sephs2	3782.53254419199	0.0940127599315214	0.208980511637301	0.449863765740446	0.652808675834243	0.917859872483584

Slc5a9	2364.12781838334	0.24174632230794	0.537034103591877	0.450150783145901	0.652601721485556	0.917859872483584
Sphk2	1871.33338825402	0.0522698234501442	0.116068768926755	0.45033495171409	0.652468940533151	0.917859872483584
Tmem168	500.311128684185	0.0984079619708419	0.218538045291497	0.450301282047162	0.652493214702252	0.917859872483584
Camkk1	122.08101458294	-0.133606719598704	0.297476535157145	-0.449133641845482	0.653335253232573	0.918266029821588
Ddx1	1452.90710574752	0.0453777383622648	0.101039665068075	0.449108162934741	0.653353632163222	0.918266029821588
Gpn2	287.397451085658	0.0755856273310137	0.168447018067845	0.448720483140702	0.653633306637073	0.918318437977376
Nod1	273.633218651554	-0.110532854757918	0.246282899599459	-0.448804423440209	0.653572747485255	0.918318437977376
Vill	6352.12463333706	0.081765345984336	0.182135636537639	0.44892557842429	0.653485343634689	0.918318437977376
Ankrd40	1650.22420535769	0.0668666165512522	0.149235464080405	0.448061169396208	0.654109051153216	0.918438850928038
Arhgef18	938.030026914738	-0.0564828826974098	0.126066105618721	-0.44804178268375	0.654123042254309	0.918438850928038
Med21	253.39104726886	-0.0709549558946741	0.158318505521419	-0.448178535168617	0.654024352621702	0.918438850928038
Srpk1	480.136540545976	-0.0519104379124735	0.115854491073518	-0.448065823184467	0.654105692601761	0.918438850928038
Tmem199	707.386356098395	0.0795083287219579	0.177356188555388	0.448297459308152	0.653938534018568	0.918438850928038
Mt2A	9634.46209455539	0.277141796688771	0.62016571363191	0.446883454852301	0.654959210567624	0.919385746240783
Sgpl1	196.392621995978	-0.104448467474793	0.233721190941587	-0.446893441942529	0.654951999281713	0.919385746240783
Abhd10	136.351080854575	0.0938900745105302	0.210406223172886	0.446232402705042	0.65542937918167	0.919417833984513
Aen	658.593589887793	0.226354332036353	0.507417985813253	0.446090478392422	0.655531890430469	0.919417833984513
Ap2m1	4505.98045536563	0.0594767858849462	0.133573901510112	0.445272506174747	0.656122834278463	0.919417833984513
Cherp	1040.37337707159	-0.050873091485721	0.114090805615322	-0.445900011060039	0.655669474268619	0.919417833984513
Galns	18.67968285801	0.289530657831091	0.650330584758606	0.445205353425843	0.656171358333435	0.919417833984513
Igfbp1	2893.33805906491	-0.359506330762227	0.806468749563732	-0.445778377595791	0.655757342162826	0.919417833984513
LOC682033	237.906308160878	0.0837837978012669	0.188118057158129	0.445378817254315	0.656046017684531	0.919417833984513
LOC688318	1022.14007393804	-0.0749412564088151	0.168277797375274	-0.445342508505087	0.65607225268264	0.919417833984513
Lurap1	25.3437209450771	0.185049288451855	0.415101082451516	0.445793317037348	0.655746549668351	0.919417833984513
Ndufaf3	449.374388968348	0.0682653546272406	0.153073434348728	0.445964741809609	0.655622714782001	0.919417833984513
Pde4a	115.510157531859	-0.146034825921018	0.32804134324874	-0.445172015437963	0.656195448643531	0.919417833984513
Rgs11	126.096468843365	0.1179952887405	0.264554052674216	0.44601580489037	0.65558582934797	0.919417833984513
Rnd3	295.718509693781	0.106911034431002	0.239957119580709	0.445542247789162	0.65592793570321	0.919417833984513
Tmem223	1089.13471457696	0.0469476864716878	0.105117754490696	0.446619952063791	0.655149487215246	0.919417833984513
Vcp	9623.69561991301	0.060674467493392	0.136177698255501	0.445553627874901	0.655919713673288	0.919417833984513
Bmp3	230.830587426393	-0.126587247082049	0.284848245494791	-0.444402411052815	0.656751670586341	0.919609049008045
Ca2	8500.10944793317	-0.0732158112260159	0.165092997512598	-0.443482233220879	0.657416967330728	0.919609049008045
Cars	420.623102367133	0.056262047636058	0.126945636343462	0.443197964551032	0.657622551027495	0.919609049008045
Catsper2	158.013621481284	-0.101122538986006	0.228136930075064	-0.443253702733413	0.657582239015454	0.919609049008045
Ccdc59	287.713353727955	-0.0846076386302428	0.190996541180527	-0.442979951926318	0.657780235764764	0.919609049008045
Dnaaf2	412.211343975069	-0.0749376987767149	0.169342523534232	-0.44252144832108	0.658111913129635	0.919609049008045
Ints4	748.70136002876	0.0485690322190403	0.10924524706993	0.444587142431472	0.656618140928995	0.919609049008045
Klhl28	44.469028207908	-0.171716653545469	0.387433227960276	-0.443216123845411	0.657609417413181	0.919609049008045
Mgat5	68.7977401003129	0.224720051454391	0.506401126115359	0.443758988409515	0.65721684229667	0.919609049008045
Morf4l1	2827.63433076943	-0.0423443671354609	0.0956473100016578	-0.442713622941691	0.657972887549529	0.919609049008045
Ndufa4l2	48.0733226201546	0.214445625975061	0.484197017274613	0.442889192465715	0.657845884993084	0.919609049008045
Nus1	1393.17523762941	0.055769807041837	0.125844536894675	0.443164307470201	0.657646893614	0.919609049008045
Polr3c	608.216129575672	0.0542776107938151	0.122013211200454	0.444850276947823	0.656427957951135	0.919609049008045
Ppp1r10	1954.00008372537	-0.0632606643388982	0.142726829570255	-0.443228960731305	0.65760013326663	0.919609049008045
Prcp	299.618714209293	0.0994304258514558	0.224467793893741	0.442960765670126	0.657794113579145	0.919609049008045
Rps3a	14247.6536751259	-0.0640776039154058	0.1447711579235	-0.442613051069645	0.658045643147321	0.919609049008045
Slmo2	1348.47438633453	-0.0618051423078176	0.13958179031705	-0.442788003846574	0.657919081077391	0.919609049008045
Smarcal1	294.857084832606	0.0794056661795158	0.179370162499446	0.44269161087347	0.65798881121986	0.919609049008045
Snupn	197.019056284949	-0.0763651753071361	0.17183492648398	-0.444410090950022	0.656746119096387	0.919609049008045
Trim26	425.595456430133	0.0745924682489591	0.167962644036229	0.444101536249155	0.656969175834526	0.919609049008045
Zbtb8a	32.2798198558308	-0.208056225331211	0.469495386935901	-0.443148604055648	0.65765825128076	0.919609049008045
Zmym6nb	489.27052592684	0.533070246967923	1.20415803397761	0.442691268028224	0.657989059237521	0.919609049008045
Cenpi	16.9196183347411	0.26406535380692	0.597314495705413	0.44208763675669	0.658425790460409	0.919770475545312
Jmjd4	24.354465282414	0.207741369064795	0.469909991025465	0.442087576413198	0.658425834125155	0.919770475545312
Zfp622	375.518618625244	0.0558297618334123	0.126304175331495	0.442026256747908	0.65847020583426	0.919770475545312
Taok3	118.639767328799	-0.108069657316306	0.244655616547023	-0.441721546562307	0.658690715931319	0.919965430975101
Asap1	185.370184061392	-0.150547055682489	0.34099875593564	-0.441488577485903	0.658859329050599	0.919974832185395
Popdc2	11.8647990158994	0.290469743093707	0.657902764889262	0.441508621935339	0.658844821047721	0.919974832185395
Nde1	323.843608459186	-0.0951085800284484	0.215497062421316	-0.441345134638088	0.658963155516571	0.920006783638539
Dmrt2	392.035809751952	-0.0966940025893238	0.219386023374318	-0.440748235015608	0.659395272570196	0.920157970292553
Grb10	431.380195451467	0.0788440289794764	0.178861436469764	0.440810666265697	0.659350071022021	0.920157970292553
Ipo13	419.206406559411	0.0564400988654077	0.128052276767313	0.440758261315153	0.659388013232998	0.920157970292553
RGD1305592	360.136346071871	-0.063523600561136	0.144091820543408	-0.440855007047394	0.659317968111836	0.920157970292553
Fam229b	81.1158691702118	-0.116255487132127	0.263931138960528	-0.440476586392914	0.659591966408485	0.920319456199219
Mrps33	1021.7457016115	0.0682433601359627	0.155012173487186	0.440245166561736	0.659759550171963	0.920440290567637
Fam110a	71.7255802248142	0.15158347798955	0.344471056827143	0.440047066321787	0.659903018960361	0.920527456247356
RGD1561102	14.8100574204603	0.234946935245727	0.534494714388036	0.439568304271685	0.660249801171651	0.920898176477725
Wbp4	347.544742718176	-0.0833967198266007	0.189798501627399	-0.439396091705297	0.660374557930217	0.920959168765012
Nop16	700.185813033853	-0.0684809133608006	0.156021793553267	-0.438918895887585	0.660720304453005	0.921328300872635
Atp13a1	1232.48153923938	0.0476225854222213	0.108731008945364	0.437985317014314	0.661396927246858	0.921485475778777
Cdc14a	131.582957276995	-0.101209025764212	0.231354716978972	-0.437462555705797	0.661775925837608	0.921485475778777
Fmo5	574.46945566992	-0.117734151994921	0.268777284570811	-0.438036094392876	0.66136011859547	0.921485475778777
Kcnk6	31.0783132084211	-0.16895966828148	0.385925187920788	-0.437804200321229	0.66152822586714	0.921485475778777
LOC687565	653.279970700169	0.062155800324925	0.141843197282985	0.438200784496707	0.661240739952865	0.921485475778777
Lppr4	34.8768306885815	0.242877615670882	0.555138442600347	0.437508190809501	0.661742837226105	0.921485475778777
Paip1	1380.92121341808	-0.0633174794985159	0.144762560623174	-0.437388501736544	0.661829621501008	0.921485475778777
Ppan	561.930436607693	-0.0918959243358429	0.210139376886966	-0.437309397682633	0.661886980842683	0.921485475778777
Ppp1r16a	1263.75480078458	0.0549033311906592	0.125417577393306	0.437764245903777	0.66155719179897	0.921485475778777
Rpa3	471.503863593231	-0.0856547746912745	0.195763223717205	-0.437542726692168	0.661717796757894	0.921485475778777
Snrpb	1786.06956909096	0.0523647477449436	0.119648816155243	0.437653705465842	0.661637333431937	0.921485475778777
Thbs2	217.878585697731	0.184725949304001	0.42227425056851	0.437454921902776	0.661781460938115	0.921485475778777
Wrap53	320.403646476052	-0.0600170080601246	0.136883856317339	-0.438452054718465	0.66105861872347	0.921485475778777
Zfp566	65.9524587779593	-0.114505834383095	0.262050158371388	-0.436961515668356	0.662139258002176	0.921723797483494
Cd248	38.7199223667947	-0.236792069254885	0.542409334509043	-0.436556036538742	0.662433351851419	0.921947188309078
Dhx38	700.638426481002	-0.047111008719847	0.107924898333153	-0.436516591143038	0.662461964360394	0.921947188309078
Cdc25a	745.888040367773	-0.0612422788664005	0.140514223874498	-0.435843982037718	0.662949930661129	0.922227314544657
Ddx6	328.612448066576	0.108458308437952	0.24885050404901	0.435837206166927	0.662954847166679	0.922227314544657
Lamtor5	4216.10009585472	-0.0726654454266224	0.166743495189615	-0.435791785124749	0.662987804605348	0.922227314544657
Rtn1	44.702241953322	-0.187128436263829	0.429187577659	-0.43600618005889	0.662832245698022	0.922227314544657
Junb	2191.29292593988	0.123242280483922	0.283031580182875	0.435436499362691	0.663245621914966	0.922360178476141
Tmem186	554.775636813368	-0.0552859696266519	0.126954621040435	-0.435478198222051	0.663215360578577	0.922360178476141
Faim	112.181531052303	-0.114508551324232	0.263295408038771	-0.434905235063464	0.663631214538246	0.922670628066264
Mrps27	152.038686334121	0.0788571246377416	0.181286091738918	0.434987173485481	0.66357173766447	0.922670628066264
Acat3	707.462199480055	-0.0832410789748594	0.19155247294188	-0.434560189677719	0.663881696858196	0.922792545044764
Cables1	196.936313662018	-0.169045030491594	0.389424260465339	-0.434089623203226	0.664223360521156	0.922792545044764
Hscb	191.581138914255	-0.076854450310983	0.177174584020981	-0.43377807678037	0.664449603083437	0.922792545044764
Itpr2	462.297429026516	0.0822048944200526	0.189496580895549	0.433806742219609	0.66442878518935	0.922792545044764
Ldlr	241.043082688255	0.0983253596571275	0.22629210008922	0.434506373038922	0.663920767906949	0.922792545044764
Ndufa1	2876.50680827652	-0.0703703427172297	0.162205981594499	-0.433833216417072	0.664409558885415	0.922792545044764
Pdcd2l	207.09505922354	0.0953319366270778	0.219540912621223	0.434233125338033	0.664119160689992	0.922792545044764
RGD1562987	1895.14947705671	0.0420187400982824	0.096753736401707	0.434285451507805	0.664081167204659	0.922792545044764
Ryk	616.595756870457	-0.0552849932909188	0.12732877724058	-0.434190875692312	0.664149838346393	0.922792545044764
Parp16	395.562666750229	0.0784100032401584	0.180813067904825	0.433652302616929	0.664540947956674	0.922806648072762
Foxq1	299.269481325385	-0.0936820545686132	0.21670523294645	-0.432301764451452	0.665522104932514	0.92295243019776
Lrrc51	28.7557381326904	0.185352565378021	0.42826295166057	0.432800840369976	0.66515946277727	0.92295243019776

Nrp1	289.401150320379	0.143471686984866	0.331464285643547	0.432842068358321	0.665129508899347	0.92295243019776
Psmd12	1721.40473401489	0.0610822410536073	0.141231266829769	0.4324980043352	0.665379502349205	0.92295243019776
Pus10	52.5916076003681	0.141654541480416	0.327777658372141	0.432166555170117	0.665620365153179	0.92295243019776
Rad52	104.206521133973	0.10516063572499	0.243049606077705	0.432671492137163	0.665253443210031	0.92295243019776
RGD1309540	341.009082716089	-0.0539038351869365	0.124719240111225	-0.432201440121546	0.665595012771221	0.92295243019776
Slain1	37.183313352763	0.179803806790766	0.415312540678065	0.432936136474972	0.665061166433736	0.92295243019776
Srsf4	1193.53799860495	-0.0696622616102983	0.161158175128144	-0.432260178888889	0.665552325667609	0.92295243019776
Sspn	44.0275542989919	0.204444127279107	0.47292795163936	0.432294446903425	0.665527422644453	0.92295243019776
Tmed7	1912.7532777557	-0.0469863188372955	0.108480213410582	-0.433132618014486	0.664918427440482	0.92295243019776
Zfp9	21.4038391720594	0.203332440236809	0.470135816681716	0.432497233824807	0.665380062236047	0.92295243019776
Hnrnpc	245.30163754567	0.114652909680164	0.265376779761035	0.432038212926566	0.665713640224629	0.92296916745464
Aasdhppt	771.28299005677	0.0615551917959385	0.142532467083176	0.431867861797533	0.665837454009869	0.923028235428244
Hsbp1	7095.70230171806	0.0699740014561624	0.162141629501267	0.431560985734486	0.66606051917185	0.923112286417174
Urod	1537.29669255428	0.0556271439816374	0.128868414815048	0.431658479399111	0.665989648792604	0.923112286417174
Exoc2	960.537668284757	-0.055062043178832	0.127712971403419	-0.431139003139332	0.666367302369624	0.923199751186391
Ift43	145.761957693242	-0.0847061500802824	0.196450025351437	-0.43118421557212	0.666334430061951	0.923199751186391
Lsr	988.954992496922	0.0599011513433978	0.138871399646922	0.431342605429884	0.666219275639682	0.923199751186391
Ahctf1	940.527691552152	-0.0441278962235592	0.102394475982225	-0.430959734890578	0.666497648069068	0.923267796216091
Hm13	1110.8539451492	0.0368018833885313	0.0854364395501178	0.430751604143604	0.666648992284564	0.923364909371966
Rbm22	402.767989943699	-0.0533122332377606	0.123844420055726	-0.430477474994608	0.666848348558641	0.923459067123032
Zc4h2	100.563026935958	-0.105247083175337	0.244513479469839	-0.430434687705302	0.66687946709008	0.923459067123032
Atpaf2	486.015577782784	0.076165761668127	0.178125521931997	0.427596005569629	0.668945274602834	0.923619046542251
Espnl	14.5267239540139	-0.392438924780457	0.913467421820684	-0.429614582201809	0.667476027807577	0.923619046542251
Gng5	1293.32080500774	-0.0548463475099741	0.127981316546689	-0.428549642947027	0.668250999046726	0.923619046542251
Jup	2386.50084235975	-0.0441908602138571	0.103155534294888	-0.428390590150305	0.66836677437966	0.923619046542251
Kap	201732.790076902	-0.0659232061089563	0.153951815485567	-0.428206747033369	0.668500604540551	0.923619046542251
Kif9	18.0502159204099	0.193536715219943	0.452597373678526	0.42761342967361	0.668932586810674	0.923619046542251
Klf12	19.7118176972169	0.203514353997759	0.475773615214514	0.427754603218171	0.668829791289497	0.923619046542251
Ntn4	266.314729908349	-0.136876653072845	0.318852602117812	-0.429278770703809	0.66772036431507	0.923619046542251
Oaf	901.4272241458	-0.109578681090704	0.256045636749036	-0.427965430233471	0.668676289148711	0.923619046542251
Phf14	1340.59399039731	0.0602776396950582	0.140675407905231	0.42848739941572	0.668296305487414	0.923619046542251
Ppargc1a	242.344569041823	-0.150324718181145	0.350540078276522	-0.428837463950589	0.668041512748563	0.923619046542251
Psmb3	1405.51401148637	-0.0801497377247263	0.186446714692968	-0.429880128790218	0.66728284101661	0.923619046542251
Rbbp9	545.237431493078	-0.0564279116636145	0.131719253337028	-0.428395319849203	0.66836333149416	0.923619046542251
RGD1306063	667.573306509653	-0.0627803598007004	0.146664742563076	-0.428053523318329	0.668612153097393	0.923619046542251
RGD1562407	1130.85830661211	0.0554111739555777	0.129338249778073	0.428420626153947	0.668344910416837	0.923619046542251
RGD1566052	93.6256274984009	0.101034885501673	0.234926521814746	0.430070154366589	0.66714460976617	0.923619046542251
Sdpr	204.375899298427	0.138773463575519	0.324148250018252	0.428117269081985	0.668565744588517	0.923619046542251
Sfta2	39.7095949202969	-0.167311807330623	0.391262690673437	-0.427620141963059	0.668927699116359	0.923619046542251
Slc25a22	147.812665586396	0.0943175277558435	0.21935971523985	0.429967406060479	0.667219351075249	0.923619046542251
Slc35f6	348.1782198616	0.0816768962218791	0.19095217286792	0.427734835352591	0.668844184887774	0.923619046542251
Sox13	1890.17957497557	-0.0619098829500587	0.144589074849739	-0.42817815256372	0.668521421074879	0.923619046542251
Tlcd1	504.763245691135	0.0588641777661302	0.13748556237858	0.42814806695151	0.668543323423649	0.923619046542251
Zc3h6	154.529131845421	-0.0799415299105899	0.186770557332157	-0.428019978376035	0.668636575162176	0.923619046542251
Zhx1	1004.36319596598	0.0543100493344439	0.12647863636918	0.429400971527854	0.66763144689501	0.923619046542251
Avpi1	672.36557676878	-0.0592507375898213	0.138625533508392	-0.427415758773144	0.669076531310998	0.923658712506063
Rabl5	194.517226512129	0.0838878748145513	0.196330007347086	0.427279945374058	0.669175438055497	0.923658712506063
RGD1560888	630.908897429712	-0.0505540044966513	0.118331992364298	-0.42722178074223	0.66921779847681	0.923658712506063
Eefsec	630.002248334425	0.0908318133090638	0.212706236031439	0.427029385709398	0.669357924367139	0.923707080182277
Tceb2	1879.62083703749	-0.0565565076816345	0.132466193333835	-0.426950501544976	0.669415380913374	0.923707080182277
Fam49b	372.870561118956	-0.0614474985643815	0.14414141580423	-0.426300090238036	0.669889192148415	0.923855422681734
Lekr1	25.3006399421012	0.176458791143719	0.413713281109157	0.426524356845969	0.669725803695629	0.923855422681734
Rab8b	24.9724890605918	-0.181888978712653	0.426555050844434	-0.426413843541588	0.669806315725736	0.923855422681734
Tbc1d20	206.452915311805	0.0865164820737158	0.202973578338405	0.42624504520225	0.669929297355521	0.923855422681734
Xylt2	392.431896462705	-0.0865724487172166	0.203099937865078	-0.426255417048565	0.669921740470524	0.923855422681734
Cast	656.428098806776	0.0979218085096129	0.229811163298855	0.426096831433171	0.670037288953714	0.92389225115224
Api5	2369.28069463812	0.051360102237723	0.120637276378881	0.425739902121284	0.670297382971283	0.923914631924763
Glrx3	1877.39690042958	0.0822532796966396	0.193109074429508	0.425942074134197	0.670150055580802	0.923914631924763
Zcchc12	15.745213986667	-0.252630350370377	0.593254736847284	-0.425837898426101	0.670225969228609	0.923914631924763
Bccip	585.9997578657	-0.0586356571403049	0.137946386022142	-0.425061205524391	0.67079205748863	0.923927696452588
Fkbp9	3174.67137496055	0.0729245087112799	0.171653837634096	0.424834712211496	0.670957171082145	0.923927696452588
Ogfr	1191.31006802823	-0.102675233012353	0.241567351908297	-0.425037705638839	0.670809188162986	0.923927696452588
Rps11	6719.52684196813	-0.0593942529412781	0.139790154398632	-0.424881517563152	0.670923048684661	0.923927696452588
Tmem42	655.543360877578	0.0530893572442591	0.124855542900582	0.425206250446824	0.670686328108958	0.923927696452588
Tmem66	2304.72697318675	0.0408303232168241	0.0959810242044199	0.425399953326856	0.670545140054595	0.923927696452588
Twf1	3837.90733550917	0.0964298307102775	0.226942919857827	0.424907861283745	0.670903843681283	0.923927696452588
Ubn2	100.4941335404	-0.101274256455823	0.238060103144488	-0.425414654190736	0.670534425219703	0.923927696452588
Yif1a	656.393715677984	0.0607797690352367	0.143108204804582	0.424711980128835	0.671046649391197	0.923938972377995
Creb3	552.827064862924	-0.0437959379850105	0.103346251502361	-0.423778679423218	0.671727228465944	0.924181288061465
Hdac8	233.173767489884	0.0693878962645779	0.163691847784063	0.423893414387453	0.671643547214167	0.924181288061465
Mrpl51	659.168489218051	0.0564566503619382	0.133249861230144	0.4236901250083	0.671791817874698	0.924181288061465
Mterfd2	431.616836296573	0.0517500280945933	0.122086574752957	0.423879760729708	0.67165350521349	0.924181288061465
Nnat	18.9615160992449	-0.286790322038021	0.675906494958781	-0.424304728800557	0.67134359098905	0.924181288061465
Nrd1	3365.06277500215	-0.0543379638262863	0.128179500955309	-0.423920856465433	0.671623533103749	0.924181288061465
RGD1304624	46.3721984948762	-0.131538643247769	0.310231658271495	-0.424001354280404	0.671564825621857	0.924181288061465
Gpank1	352.107930641777	-0.0827425591154304	0.195373087859261	-0.423510525538884	0.671922820744531	0.924249640636856
Ccdc125	134.553727099732	-0.0985718097190763	0.232919641826998	-0.423200932930727	0.672148666184938	0.924264879533446
Cir1	439.978873488003	-0.0599369554914678	0.141641045005054	-0.423160924076273	0.67217785449793	0.924264879533446
Ino80d	26.181956550024	-0.179753905019967	0.424679153240451	-0.423269905405956	0.67209834872905	0.924264879533446
Aldh5a1	293.961140610955	-0.126534837366814	0.299200017543035	-0.422910527899998	0.672360541332629	0.924404247343251
Adnp2	278.82716587515	-0.078834242085192	0.186718502321991	-0.422209053226254	0.672872434035361	0.924996139966849
Nfu1	438.785236900892	-0.0528712825942884	0.125361493581449	-0.421750579733936	0.673207081500048	0.925008666384132
Ppp1r1a	6655.63328584986	-0.0960204750674547	0.227616372279018	-0.421852233677419	0.673132877002092	0.925008666384132
Rnpep	1407.13531093085	-0.0507228459916522	0.120187743217049	-0.422030105849071	0.673003043013127	0.925008666384132
Tbca	2082.46972839831	-0.0802047541776125	0.190073964067709	-0.42196602028588	0.673049819776941	0.925008666384132
Akap7	96.0685729870254	0.107854383694944	0.256226990087259	0.420932953465263	0.67380404115427	0.925482431076785
Carhsp1	2321.15860697163	-0.0546703602656289	0.129910102207032	-0.420832247352891	0.673877582226946	0.925482431076785
Krt10	69.4982988230832	0.147951587409102	0.35142570606744	0.421003884618245	0.673752245245102	0.925482431076785
Taz	871.980872838197	-0.0382249518899779	0.0907724682071618	-0.421107331825996	0.673676707977362	0.925482431076785
Eif2c1	158.055663529808	-0.107035975538146	0.254555847341861	-0.420481307563131	0.674133881872523	0.925610739473677
Ezh1	1649.27207583306	-0.0661448304178214	0.157295430277957	-0.420513363299474	0.674110469238524	0.925610739473677
Bcdin3d	80.6323277462044	-0.137417908460099	0.327677430033733	-0.41936946479943	0.674946136608494	0.925651839569056
Ccdc94	379.665336222888	-0.0543372490661137	0.129445041143148	-0.419770804553449	0.67465289483674	0.925651839569056
Emc2	1293.06067238664	0.057797100124365	0.137687153768707	0.419771188105575	0.674652614615248	0.925651839569056
Grb14	86.0647764489053	-0.127679786935519	0.304488443987891	-0.419325558839917	0.674978219808933	0.925651839569056
Havcr1	51.9372668964827	-0.168139907264901	0.400705131704178	-0.419610067257712	0.674770332767397	0.925651839569056
Keap1	1073.80759280458	0.0522832964321537	0.12465219069903	0.419433434253799	0.674899393559819	0.925651839569056
Mrpl19	386.13507143788	0.066574810924908	0.158657092406265	0.419614464851235	0.674767119690911	0.925651839569056
Ostf1	1689.06169222935	0.0396755538411095	0.094598170040762	0.419411430728665	0.674915471589642	0.925651839569056
Slc10a3	218.848296619405	-0.0844717449940553	0.201165805724443	-0.419911051432692	0.674550434093029	0.925651839569056
Vwa5a	277.033928907567	0.085030112587227	0.202540956261894	0.419816881269581	0.67461923171387	0.925651839569056
Cideb	929.76163774414	0.0638783827911252	0.152418302514659	0.419099161565466	0.67514366342556	0.925767025997697
Aip	1019.31079209495	0.0551621542352573	0.131738722612222	0.418723919144328	0.675417912706344	0.92580800721422
Inpp1	577.657935507704	-0.0681249073296857	0.162683401015376	-0.418757580088006	0.675393309544533	0.92580800721422
Prpf40a	809.495325253185	0.051941791072564	0.12399751121885	0.418893819416174	0.675293734231931	0.92580800721422

Arhgap20	20.3348604141372	-0.225534038294075	0.53952514543701	-0.418023219494977	0.675930140657815	0.926024002955389
Atxn1	43.1879523161226	-0.161422745077102	0.385864611075324	-0.418340372358196	0.675698275841699	0.926024002955389
Ccdc22	719.512603192812	-0.0477909835690876	0.114345868448099	-0.417951118109527	0.675982856987583	0.926024002955389
Slc9a1	594.353037313179	-0.0632778939980369	0.151316724686588	-0.418181758355529	0.675814231873332	0.926024002955389
Trip12	3798.6113475848	0.0329988254105707	0.0789441752209767	0.418002028879293	0.675945633835809	0.926024002955389
Ddx41	1619.31831145579	0.0508248719566803	0.121772664945408	0.417375048656984	0.676404102421014	0.926489398423384
Arl11	29.3284725132189	-0.21630385252619	0.518957921738739	-0.416804221431819	0.676821614441425	0.926503007315577
Egln2	1346.58970520272	0.0482049503825749	0.115626428466854	0.41690252844222	0.676749704061537	0.926503007315577
Idh1	6437.59986558836	0.123723189053308	0.296816655704517	0.416833714265937	0.676800040483322	0.926503007315577
RGD1311458	153.646191265583	-0.0960786993235919	0.230411753632516	-0.416986971406102	0.676687937415682	0.926503007315577
Rimbp2	2748.24008932103	-0.0626257814407449	0.150222125987351	-0.416887865413501	0.676760429701329	0.926503007315577
Afm	3870.11827602166	0.0670970269560602	0.161333757102268	0.415889570547397	0.677490811581213	0.926753796523356
Entpd2	661.417799139454	-0.0486734220240568	0.116963375207017	-0.416142420120044	0.677305790692929	0.926753796523356
Hsp90b1	9471.61241936967	-0.0721106107167046	0.173381287759949	-0.415907689049717	0.677477552847864	0.926753796523356
Nek4	249.397192279279	0.0753830085848028	0.181195117655481	0.416032228462876	0.677386420266809	0.926753796523356
Pask	50.7409926519393	-0.164425569281233	0.395414411972038	-0.415830997310388	0.677533674909646	0.926753796523356
Pcdhgc3	395.726239993998	-0.122155037207655	0.293631608445154	-0.416014603654196	0.677399317056247	0.926753796523356
Usp2	866.035331139673	0.233142744036984	0.560744282117021	0.415773734074972	0.677575580600835	0.926753796523356
Mob1b	183.245302300801	0.0980442961241748	0.236107055526063	0.41525356328605	0.677956291403886	0.927051396546748
Tram1	2865.84146125483	-0.0509949886236688	0.122780557172389	-0.415334396569563	0.677897124465799	0.927051396546748
Anapc13	1484.11998195372	0.0536285480441867	0.12946829979935	0.414221459054458	0.678711927532961	0.92723348139681
Ccdc28b	85.6264463339936	0.13878106493954	0.335096599176271	0.414152412410896	0.678762490301619	0.92723348139681
Ercc3	743.719795649146	-0.0411491792320467	0.0994561608441238	-0.413741882682755	0.679063150544694	0.92723348139681
Fxr1	1255.95307139489	-0.0555947264145909	0.134570681173493	-0.413126588420224	0.679513870105522	0.92723348139681
Klc4	3466.06213591311	0.0534496838469455	0.129397281491032	0.413066512920907	0.679557883163419	0.92723348139681
Klhl17	117.382630087836	-0.0908790673690618	0.219497680221113	-0.414032017456922	0.67885065883078	0.92723348139681
Med13	445.609749234384	0.0619079038506995	0.149400166438667	0.41437640483563	0.678598466199916	0.92723348139681
Ngdn	456.893979368474	0.0557737369695228	0.134712177612521	0.414021493512988	0.67885836601266	0.92723348139681
Panx1	61.4189766240279	0.137928797238381	0.33384909002998	0.413147141500354	0.679498812572076	0.92723348139681
Papola	1685.00446803709	0.0488050325915538	0.118000966975406	0.413598581795741	0.679168112047276	0.92723348139681
Ppil1	498.324335526397	-0.0675163653707104	0.163130946026002	-0.413878341390535	0.678963206417273	0.92723348139681
RGD1306119	158.369144168251	-0.0935662372356496	0.22632530109072	-0.413414836011395	0.679302706734089	0.92723348139681
Rnf186	109.584667664469	-0.157935974246676	0.380611925762852	-0.414952773563594	0.678176475665401	0.92723348139681
Rreb1	957.834242078501	0.0603139099203853	0.145999829725552	0.413109453851844	0.679526423277278	0.92723348139681
Spsb2	185.598376222196	-0.0756418158766065	0.18297984697615	-0.413388780932065	0.679321793035092	0.92723348139681
Tigd3	124.090060267972	-0.206222579615526	0.499096647937886	-0.413191674333166	0.679466187505686	0.92723348139681
Vegfc	100.162319769446	0.0946745373547773	0.22823849577841	0.414805298430876	0.678284440536777	0.92723348139681
Zfp597	24.2986572461979	0.192877172342425	0.466741832292791	0.413241665943993	0.679429564015352	0.92723348139681
Dnajb9	854.827042611411	0.0751853184017167	0.182297990261652	0.412430868238336	0.680023641833501	0.927556520704308
Napepld	53.6176748556947	-0.170906597515219	0.414324634762359	-0.412494414224836	0.679977074012196	0.927556520704308
Nsmce1	948.234681719851	0.0613787349226515	0.148829662991412	0.412409285144947	0.680039458651152	0.927556520704308
Accs	189.649236675794	-0.0750371491675603	0.183818258672302	-0.408213796113319	0.683116720419257	0.927567296156515
Asmt	14.3343059255998	0.260874500964092	0.638295128732695	0.408705141588729	0.68275606010183	0.927567296156515
Cbp	18.7619812130213	-0.232345549426101	0.56923649561127	-0.408170507719466	0.683148498691377	0.927567296156515
Ccdc90b	497.401950222122	0.0552901396182952	0.134495909744435	0.411091606602428	0.681005364302291	0.927567296156515
Ccm2	691.777110683195	0.053852010788068	0.131497019815537	0.409530275770592	0.682150553181679	0.927567296156515
Chordc1	822.09032775885	-0.0871106296802278	0.213216919004513	-0.40855402135505	0.682866978563147	0.927567296156515
Dcp1a	50.3167911254782	-0.125569531353729	0.304813132166702	-0.411955779139642	0.680371835672786	0.927567296156515
Espl1	23.7226139599047	0.174623015607678	0.425283189069139	0.410604087102276	0.681362866300832	0.927567296156515
Fam168b	1176.0632401339	0.0892212974719786	0.218421042036398	0.408483068481611	0.682919058555376	0.927567296156515
Figf	11.9749023153827	-0.369499000520403	0.899523650385302	-0.410771857262599	0.681239831002744	0.927567296156515
Ganab	5733.45606753717	-0.0362232000498852	0.08864320635237	-0.408640453571733	0.682803538645421	0.927567296156515
Gfm2	367.293828729999	0.0692337138522141	0.169416190145398	0.408660552411171	0.682788786727475	0.927567296156515
Gpr137	347.395917928401	0.0651823938999563	0.159679463197712	0.408207746911378	0.683121161141289	0.927567296156515
Hnrnpul1	2020.45894858742	-0.0463415373704516	0.112566891270912	-0.411679996198193	0.68057398886165	0.927567296156515
LOC24906	25.0894391245606	-0.168978031307659	0.411271232124575	-0.410867617544607	0.681169608400752	0.927567296156515
LOC691849	304.629183726899	0.0903270193646903	0.220600099452322	0.409460465289648	0.682201774180092	0.927567296156515
Mapk9	296.394193306861	0.0574260185354153	0.139677122363728	0.41113403228536	0.680974256592848	0.927567296156515
Mrpl41	1058.11888052485	-0.0651010061716133	0.159491876793482	-0.408177566660084	0.683143316641997	0.927567296156515
Nsd1	609.297148191047	-0.0847930283682789	0.207612739642058	-0.408419196791436	0.682965942191906	0.927567296156515
Pcdhga12	21.7067962202517	0.254992514785075	0.619960753672216	0.411304285432065	0.680849427633416	0.927567296156515
Psma3l	1469.52200221015	0.0738208532199029	0.179246306961027	0.411840302160053	0.680456479302229	0.927567296156515
Ptger3	257.411445511823	-0.151172013311753	0.367807217895559	-0.411008827332691	0.681066061963758	0.927567296156515
Runx1	14.5578811694528	-0.272391654938681	0.666853036712206	-0.408473291629078	0.682926234963818	0.927567296156515
Sdc2	6223.96120197447	0.0531808595664469	0.12907005877805	0.412030954893242	0.68031673465799	0.927567296156515
Slc37a1	293.911368054743	0.0830659230519533	0.203016087451788	0.409159313897623	0.682422750258507	0.927567296156515
Snapc2	372.713054248936	-0.0717231960437855	0.174848077884515	-0.410202942529102	0.681657082542609	0.927567296156515
Sprr1a	119.229240767416	-0.384666156771319	0.933811639193855	-0.411931208207466	0.680389845616822	0.927567296156515
Stag2	1863.46448259915	-0.0415956277306388	0.101026834454963	-0.411728507134229	0.680538427926446	0.927567296156515
Sult2b1	651.844586497081	-0.0965936390505087	0.236498867265003	-0.408431719642331	0.682956749966916	0.927567296156515
Tcp1	2350.11765995748	-0.0545956775405417	0.133008920261229	-0.410466286270995	0.681463929743819	0.927567296156515
Thbs1	614.768546753243	-0.207128321593881	0.50636373953852	-0.409050461991314	0.682502629302703	0.927567296156515
Tmem160	489.314606691707	0.0669183267573951	0.163254789983274	0.409901153676723	0.681878459051408	0.927567296156515
Toe1	175.804202326025	-0.0897083828490712	0.2196132100509	-0.408483546268821	0.682918707850589	0.927567296156515
Vamp8	1448.9998083124	0.0540493085663529	0.132090119620946	0.409185098184906	0.682403829436927	0.927567296156515
Vpreb3	17.1775309876119	-0.330429903587239	0.808353917914906	-0.408768852682202	0.682709299809884	0.927567296156515
Wwp2	617.615702188436	0.049133498384585	0.119904908175766	0.409770535102376	0.681974282494814	0.927567296156515
Zbtb44	212.126770392523	0.070972368699226	0.172785926893431	0.410753178660203	0.68125352865344	0.927567296156515
Zfp498	141.229801103722	-0.0883575559798202	0.215090008911513	-0.410793399595655	0.681224033412029	0.927567296156515
Nmral1	361.029420695362	-0.081944539420537	0.200816999308072	-0.408055790609771	0.683232715931954	0.927570837229167
Apoa1bp	833.403888969785	-0.0573621075523329	0.140723034331029	-0.407624152115691	0.683549629898591	0.92757520211593
Clta	3634.31902727732	0.0364815500984161	0.0894650225897656	0.407774446843871	0.683439275443086	0.92757520211593
Dpcd	815.641465400109	0.0572334856051537	0.140313933959233	0.407895951529535	0.683350065125655	0.92757520211593
Slu7	687.099463929437	-0.0501191696256049	0.12295960067476	-0.407606802157524	0.683562369604349	0.92757520211593
Gnpnat1	609.551364028551	0.0539152679124302	0.132329023726698	0.407433429145391	0.683689678672415	0.927637207567227
Cplx3	38.3067565890283	-0.175858721503573	0.4317915544555	-0.407276890177564	0.683804634104727	0.927682438676811
Tfpt	185.824238606543	0.0877571831779095	0.215568541171701	0.407096428360623	0.683937166535149	0.927751501949934
Mettl7b	4061.78594826828	-0.0625119939008296	0.153631711345293	-0.406895121804192	0.684085018977908	0.927841327649512
Arfrp1	309.374070885273	-0.0629109969922685	0.155092150345778	-0.40563624175697	0.685009895689069	0.928219715509367
Cmc1	240.7740883462	0.166396173400643	0.410050694526683	0.405794150873742	0.68489385674106	0.928219715509367
Fam53b	377.521116135213	-0.0846813740707507	0.208590633727751	-0.405969206562148	0.684765226386761	0.928219715509367
Gemin8	273.402601405016	-0.0707469479787318	0.174401720924463	-0.405655102505405	0.684996035544813	0.928219715509367
LOC301124	4566.28928661635	0.0540645160581189	0.133240036469232	0.405767796908429	0.684913222341092	0.928219715509367
Man2c1	1347.98735445967	0.0533924444839876	0.131585040382831	0.405763788411273	0.68491616791038	0.928219715509367
Tapbp	808.013022881005	0.0625612314308507	0.15423373286095	0.405626125169733	0.685017330080289	0.928219715509367
Ttc14	289.95330986553	-0.0685053152935503	0.168849514869382	-0.405718164760759	0.684949693935649	0.928219715509367
Rraga	1358.17967842865	-0.0580785299864907	0.143223779247652	-0.405508989439984	0.685103412006303	0.928225698040725
Ddx25	84.8045786750709	-0.154715397659722	0.381937701638949	-0.405080192386915	0.685418565728091	0.928542004537006
Fermt2	2650.91182597197	-0.0412328415574516	0.101966694507019	-0.404375583192148	0.685936552452786	0.929132982383311
Dusp19	67.9117806832176	-0.124548348910113	0.308393635521396	-0.403861605961941	0.686314491028449	0.929145153821509
H1f0	5901.27547329874	0.189016986020637	0.467904260553625	0.403965088492658	0.686238391774286	0.929145153821509
Mgea5	541.563586234394	-0.0630474009385064	0.156156740825316	-0.403744344338194	0.686400727031938	0.929145153821509
Rnf38	89.392729403619	0.134101768062341	0.332133255939378	0.403758930080817	0.686390000229888	0.929145153821509
Rpn2	5564.50267094815	0.0348434462075866	0.0863110263208588	0.403696349039542	0.686436024691115	0.929145153821509
Slc39a7	991.123085619521	-0.0416136905322794	0.103080623007467	-0.403700417383634	0.686433032641882	0.929145153821509

Tuba1b	2909.85241068192	0.0550974264729953	0.136556453339279	0.403477280829079	0.6865971448849	0.929252577847318
Dll4	168.362055581194	-0.118767021025702	0.294523043796305	-0.403252049465583	0.686762812800317	0.929366130526064
Pole2	18.4455805503346	-0.180430277991803	0.447771227840085	-0.402951924495339	0.6869835917545	0.929554226652577
Cyp2d4	842.521046730296	-0.0644084176859471	0.159889994108857	-0.402829570699067	0.687073605734082	0.929565361596664
Kat3	562.018577200679	0.0935742615150907	0.23242727792637	0.402595867188763	0.687245550473104	0.929687327621673
Arpc4	2304.83919362636	0.0522412522988673	0.129798991104672	0.402478107528118	0.687332196954392	0.929693889156684
Gtpbp8	150.280380493844	0.0801581678608581	0.19937849270128	0.402040193878663	0.687654445836268	0.930019089882797
Rabif	160.722258583274	-0.0828014129527342	0.206262087076871	-0.401437870265878	0.688097772271754	0.930507945227931
Atn1	755.172426482987	-0.0463920125155608	0.115753256731756	-0.400783648127229	0.68857941887996	0.930716336661865
Pex7	470.969499746199	0.0623010260392999	0.155382344253413	0.400953057689059	0.688454685293555	0.930716336661865
Tubb5	1428.19046037287	-0.0503513379699393	0.125579260796177	-0.400952654528384	0.68845498212434	0.930716336661865
Ube2g1	1036.78917547799	0.0628317385323233	0.156770071068307	0.400789118127954	0.688575391272433	0.930716336661865
Pdcd7	1202.88138956978	-0.0669704161526796	0.167178051336378	-0.40059335311864	0.688719540190828	0.930795040880969
Rpl14	10046.3346453371	-0.0588238608149672	0.146947502915884	-0.400305276698979	0.688931681924963	0.930971049430404
Phlda2	65.6346972570297	0.135166282035147	0.338067726602844	0.399820128923274	0.689289003838023	0.931343178509626
Msr1	7.3251916223629	-0.586563404829724	1.46990521469048	-0.399048454939481	0.689857501334679	0.932000518265774
Rhbdf1	889.347396022845	-0.0630405811601291	0.158022371925617	-0.398934533078664	0.689941443186818	0.932003142769358
Acss1	5240.42216212293	0.0602184265593997	0.151078269342616	0.398590921258411	0.690194652223024	0.932178497779852
Gnat1	55.0073831074527	-0.123493593838364	0.309957977621734	-0.398420440041302	0.690320293442992	0.932178497779852
Pmm1	752.150245873524	0.066852489694319	0.167885891858589	0.39820195106465	0.690481327890953	0.932178497779852
Sfxn3	674.45928066898	0.0997453266587839	0.250465177259127	0.39824029731522	0.690453064273654	0.932178497779852
Ube2v2	1153.17612243293	-0.0709545643359127	0.178120603561076	-0.398351245826444	0.690371290619371	0.932178497779852
Nr1h2	2092.6780074986	0.0358609411841558	0.0902506374813283	0.397348342182901	0.691110603413303	0.932799430867849
Nufip1	297.989482790085	0.0638828970400522	0.160815223221386	0.39724409020723	0.691187472001498	0.932799430867849
Utp23	216.392422796163	-0.107725618722282	0.271163627895925	-0.397271638376325	0.691167159473919	0.932799430867849
Hsf1	493.157758086999	-0.0479448968679487	0.121009088188334	-0.396209058226512	0.691950809471334	0.933497080172247
R3hdm4	1685.33656364371	0.0349214181951757	0.0881298980805205	0.396249388184581	0.691921060206572	0.933497080172247
Sin3b	1912.50097887689	-0.0510036222522457	0.128704687951617	-0.396284106383283	0.691895450818967	0.933497080172247
Kcnj6	11.7767638167854	-0.268372840308054	0.678324448931754	-0.395640818682999	0.692370020071322	0.933824105928257
LOC502894	118.043853808361	0.0907811009085392	0.229430776475849	0.395679700443743	0.692341332602759	0.933824105928257
Lpin3	1424.21450137457	-0.0638047676440939	0.161307926484135	-0.395546387798677	0.692439694242772	0.933824105928257
LOC500846	145.508850934981	-0.121759252370219	0.307926554894054	-0.395416538245984	0.69253550570701	0.933842514873161
Rapgef3	1711.09497275494	0.0721906237088354	0.182681128339242	0.395172858658811	0.692715321668371	0.933863402453174
RGD1306730	490.316818793324	0.0518747002217404	0.131244647262138	0.395251930679732	0.692656970970248	0.933863402453174
Appl2	342.863929137364	-0.0618500272773837	0.156626966332324	-0.394887475162822	0.692925933779089	0.933997294078151
Crip1	119.838367364244	-0.159924038201238	0.405975816565288	-0.39392503611239	0.693636385651388	0.933997294078151
Dos	247.465477715574	-0.265470537009737	0.672433165470662	-0.394790963089876	0.692997164753047	0.933997294078151
Fam20b	1654.93070616381	-0.041084391686115	0.104248269430427	-0.394101426436953	0.693506157916364	0.933997294078151
Higd1a	1039.25822095718	-0.0532510560914992	0.134932923217795	-0.394648354320071	0.693102422476051	0.933997294078151
Itih1	52.8410091428271	-0.167862615406599	0.425939019780945	-0.394100112013518	0.693507128312461	0.933997294078151
Mrpl10	1451.31772698348	0.035187434675087	0.0892245778753206	0.394369303985463	0.693308403122027	0.933997294078151
Mul1	685.38578422775	0.0648107585497401	0.164445570824392	0.394116778121986	0.693494824303533	0.933997294078151
Ncln	1601.03074801055	-0.0379708655414579	0.0963664301160777	-0.394025860413427	0.693561946671462	0.933997294078151
Wasf2	107.35095486839	0.140888116341056	0.357254925808636	0.394362977703275	0.693313073121937	0.933997294078151
Abcf1	1671.0518799202	0.0420630679875219	0.107963350766456	0.389605062170699	0.696828608310344	0.934021932259454
Acot1	1303.24133944526	0.176953493310366	0.454490297749419	0.389344930324405	0.697021003562739	0.934021932259454
Aktip	2905.30742714371	-0.0482231935315623	0.123431823866003	-0.390686874917388	0.696028701647154	0.934021932259454
Alox12	50.1793201000311	-0.11756039578602	0.299975228641523	-0.39190034563323	0.695131847593765	0.934021932259454
Aqp2	12325.4212492239	-0.0578962588920721	0.147328458488048	-0.392974035608801	0.6943386584559	0.934021932259454
Arrb1	122.725199864989	-0.11816613214958	0.300534342319121	-0.393186786034943	0.694181528640789	0.934021932259454
Atpbd4	232.771745339625	-0.0589943657041305	0.150482755055297	-0.392034061859463	0.695033046434113	0.934021932259454
Ccdc163	103.291437251658	-0.102286849181488	0.260380457683206	-0.392836121771995	0.694440523675746	0.934021932259454
Ccdc176	114.234204763513	0.101801668294532	0.258780367804388	0.393390229553595	0.694031284866413	0.934021932259454
Clasp2	490.109979056546	-0.0643591278112522	0.163493147340608	-0.393650308028945	0.693839233483081	0.934021932259454
Cubn	6053.45545393558	-0.0537218313816557	0.137903659456494	-0.389560593195163	0.696861496480538	0.934021932259454
Eif1a	819.395442778787	-0.0704982427997886	0.181014625298778	-0.389461584573213	0.696934722889902	0.934021932259454
Fam98a	704.316875905191	0.0472414390109981	0.12116213880155	0.389902650104043	0.696608534197625	0.934021932259454
Fas	28.1547153464176	-0.222253837727455	0.569587650902559	-0.390201292769033	0.696387705752798	0.934021932259454
Ftsjd2	1441.40265795754	0.0403100906021072	0.103197911488174	0.390609558089036	0.696085859527317	0.934021932259454
Ggt5	45.8935078736534	-0.211136825638636	0.538962493907858	-0.391746787624767	0.695245315972174	0.934021932259454
Gltscr2	2510.89822239407	0.0482658678184547	0.123376436075728	0.391208154114851	0.695643381536882	0.934021932259454
Gmppb	419.194981281221	0.0570754622182434	0.145240098700686	0.392973171519705	0.694339296667829	0.934021932259454
Itga8	157.710832378382	-0.350523122325875	0.898897801727272	-0.389947691108299	0.69657522741334	0.934021932259454
Nelfe	367.795656249633	0.070678973438814	0.18041495181333	0.391757848938948	0.695237142224463	0.934021932259454
Nsa2	1421.60925218081	-0.0544994672251834	0.139845059301053	-0.38971321187586	0.696748625803077	0.934021932259454
Pdia3	8458.96702440541	-0.0507135444523143	0.129917332915656	-0.390352413447697	0.696275970825058	0.934021932259454
Pfdn1	1809.41399975472	0.0552380277487051	0.140941699192135	0.391921113945159	0.695116501821749	0.934021932259454
Pitx2	78.8806116727623	-0.116485699652974	0.296905211311201	-0.392332957507031	0.694812215103714	0.934021932259454
Pknox1	85.41627984981	0.137156075556292	0.352487195072549	0.389109384606333	0.697195231561973	0.934021932259454
Plxdc1	8.87228965241456	0.352857008935242	0.905691887963611	0.389599392050003	0.696832801761685	0.934021932259454
Psma4	2220.28962050337	0.0491366677349379	0.125340255464662	0.3920262293449	0.695038833633248	0.934021932259454
Ptprj	1828.82321479127	-0.0619297920163835	0.158592862393801	-0.390495455354137	0.696170215215528	0.934021932259454
Rad51d	438.715547734596	0.0558011947686616	0.14341988998552	0.389075704731717	0.697220145131345	0.934021932259454
RGD1563015	16.393789228789	0.19523648649416	0.500884054832257	0.389783792497734	0.696696429465617	0.934021932259454
RGD1564719	72.5459740523518	0.0956800884474985	0.246029637782859	0.388896595181529	0.697352640960208	0.934021932259454
RGD1566265	116.635832590384	-0.0928961220414335	0.236458619183843	-0.39286418216461	0.694419797403359	0.934021932259454
Rnf5	3626.45536319705	0.0635786306236458	0.163417082200921	0.38905743369886	0.697233660658058	0.934021932259454
Sigirr	1370.67900331269	0.0475768292938419	0.12123880681467	0.392422447431123	0.694746102820632	0.934021932259454
Slc7a6os	280.757757839944	-0.0664041896931852	0.169240186374817	-0.392366559713659	0.694787390599983	0.934021932259454
Slmap	784.398166696421	0.0898044055555907	0.229742898466635	0.39089088783579	0.695877889653139	0.934021932259454
Sycp3	72.9113708588137	0.106908664204737	0.273989268952675	0.390192888259439	0.696393920034733	0.934021932259454
Themis2	12.5713046119666	-0.287562700450916	0.739424283627916	-0.388900806773639	0.69734952533981	0.934021932259454
Timm8a1	1010.44348708559	0.0668449343249424	0.171493392297036	0.389781398744292	0.696698199689495	0.934021932259454
Tns3	3194.1662891606	-0.0419390837728909	0.107825001993513	-0.388955093879006	0.697309365684176	0.934021932259454
Tp53inp1	50.705429307154	-0.151408777318335	0.385528470680703	-0.392730469557805	0.694518563712749	0.934021932259454
Ubr7	722.573833050952	-0.0467917729762611	0.1194759569474	-0.391641750957989	0.69532293448323	0.934021932259454
Ubxn1	2903.15197093251	0.0565597498838094	0.144009408681372	0.392750379309942	0.694503857121234	0.934021932259454
Ubxn8	637.157299711686	0.0440895708174764	0.11272840553968	0.391113230125101	0.695713541776752	0.934021932259454
Vwf	1570.88848984706	0.271450676002892	0.697890312144328	0.38895893993547	0.69730652054156	0.934021932259454
Capzb	3247.95160980632	-0.0301567404098738	0.077651935782302	-0.388357870361642	0.697751216872973	0.934124620062842
Mtx1	874.546484710882	0.0518031300431659	0.133355389552219	0.388459215762563	0.697676230039644	0.934124620062842
Naa50	1131.69289056942	-0.0641548322910443	0.165222430721536	-0.388293720234452	0.697798683945434	0.934124620062842
Nudt16	277.880605579801	0.0768037440408067	0.197842409516826	0.38820667534518	0.697863093597152	0.934124620062842
Phlpp2	119.642673848268	-0.0875736226215829	0.225631651787585	-0.388126497004182	0.697922424210246	0.934124620062842
Slc35c1	635.163865646711	0.0436701283828138	0.112457138369453	0.38832686849407	0.697774156163785	0.934124620062842
Sh3rf1	348.070340328606	-0.0744826182516416	0.192002312548534	-0.387925631014543	0.698071069743499	0.934213561604217
Ssu72	1695.5463150095	0.0560922697433246	0.144735174175547	0.387551057044995	0.698348294183164	0.934474536341634
Efhd2	508.113367128279	0.0577298134934249	0.149038761146918	0.387347647344682	0.698498855785597	0.93453789359433
Tbx3	80.789496454293	-0.155388534536978	0.401283106148399	-0.387229195937079	0.698586537668716	0.93453789359433
Urm1	509.045606056325	0.046241444758561	0.119439452834165	0.387153856295412	0.698642308804419	0.93453789359433
Ggcx	908.150029757302	-0.0875598557815917	0.226261262785452	-0.386985623184728	0.698766851393187	0.934594496697453
Ppil4	403.400712266392	-0.0478294229902278	0.123653202903284	-0.386802944583957	0.698902097127986	0.93466540015963
Isg20l2	125.595135578652	-0.0844901731973332	0.218535436432281	-0.386620012647308	0.699037539996267	0.934736550540891
Riok1	238.387660865991	-0.0606448339332812	0.156953070697531	-0.386388323998781	0.699209096099817	0.934783223244971
Tmem198b	68.2758900380363	-0.110833529933086	0.286955323626267	-0.386239671501744	0.699319175369698	0.934783223244971

Trit1	522.934428529305	-0.0418524657967573	0.10834063161711	-0.386304428653042	0.699271221009081	0.934783223244971
Lgals1	397.708938701726	-0.0902832117384763	0.233921165404137	-0.385955719665201	0.699529463273729	0.934830009567291
Peo1	456.528841772102	-0.0563427858624655	0.146019014945343	-0.385859238151658	0.69960092040994	0.934830009567291
Rtf1	614.522746562238	0.0455483818717373	0.117989545351934	0.386037438621172	0.699468941816824	0.934830009567291
Utp15	198.573500449477	0.0639249761601342	0.165733923648063	0.385708458190366	0.699712597951092	0.934869329765147
Znrd1	102.673330665759	-0.0858575716828053	0.22291173361537	-0.385163985270291	0.700115924409254	0.935298260088808
Bcl9l	661.476882161142	0.0432319175655377	0.112379849293024	0.384694567909706	0.700463720246457	0.935349175365403
Ccnf	29.2946668177805	0.213289336962288	0.554750852002918	0.384477709573966	0.700624413888254	0.935349175365403
Chrac1	590.221187447025	-0.0542763625861432	0.141443150550369	-0.383732703739619	0.701176570803393	0.935349175365403
Epm2aip1	1081.59204873026	-0.0508722048019182	0.132698570237816	-0.383366638470538	0.701447935895026	0.935349175365403
G0s2	1003.53150850171	0.106550062838754	0.278368548160292	0.382766169320968	0.701893147624361	0.935349175365403
Gas6	2553.41942087246	-0.0911362726796026	0.238261919957782	-0.382504567644512	0.702087141533105	0.935349175365403
Il10rb	623.210675748377	-0.0624927739947211	0.163439299725365	-0.382360754725031	0.702193796024891	0.935349175365403
Irf2	242.120599387255	0.0747962203468121	0.195671823082505	0.382253403522868	0.702273413612505	0.935349175365403
Jagn1	807.105345816864	-0.0531999100848101	0.139158278541101	-0.38229784560821	0.702240452508188	0.935349175365403
LOC100125367	189.313794423316	0.0729059638437977	0.189447663262036	0.384834326211547	0.70036016540702	0.935349175365403
Pafah1b3	250.59769521026	0.0756399234282198	0.196848473552928	0.384254559169249	0.70078978398748	0.935349175365403
Pion	492.207320556945	-0.0647532372161962	0.168534413437797	-0.384213739469271	0.700820035782252	0.935349175365403
Polh	99.6789545677607	0.0865123927013322	0.226338271517496	0.382226090715042	0.702293670822483	0.935349175365403
Rab30	29.5131006960864	-0.159517753283963	0.416700490339974	-0.382811532460202	0.701859510011905	0.935349175365403
Rcn2	383.004021832521	-0.070343279789109	0.182932248183696	-0.384531871704064	0.700584278090916	0.935349175365403
Rnf19a	2901.99425459295	0.0748738818722768	0.195753579389344	0.382490486793891	0.702097583910022	0.935349175365403
Rpl19	13038.0841894047	-0.0581794507350614	0.152038987928163	-0.382661391843457	0.701970844307435	0.935349175365403
Rpl37a	198.660321827141	0.108608451431539	0.283387473494258	0.383250713563173	0.701533879257852	0.935349175365403
Secisbp2l	468.409600272366	0.0949590107144065	0.247702865126995	0.383358548015671	0.701453933799559	0.935349175365403
Serpinh1	2109.96403428069	-0.0790867143536899	0.20640036872783	-0.383171381142141	0.701592696213757	0.935349175365403
Sumo3	2837.6888484941	-0.0558666352343925	0.145987152009824	-0.382681862515087	0.701955664242194	0.935349175365403
Thg1l	122.427880941829	-0.105242859266172	0.27497454758432	-0.382736730329195	0.701914977497464	0.935349175365403
Tor1aip1	909.277595469184	0.0452017109535076	0.118181415292006	0.382477319651504	0.702107348730622	0.935349175365403
Trmt6	280.99955211472	0.0537393779070768	0.139838381162647	0.384296338818254	0.700758821258444	0.935349175365403
Yme1l1	838.135881444735	0.0543168636712073	0.141589118205569	0.383623150984995	0.701257778536796	0.935349175365403
Zfp180	359.607679038887	-0.0577910030883661	0.150940759014986	-0.382872084819901	0.7018146102199	0.935349175365403
Ddx49	632.241961329171	-0.0464419608273013	0.121710049431601	-0.381578686757505	0.702773895642663	0.935572660569741
Ints10	439.269561159297	0.0462400755055224	0.121258694407419	0.381334103352291	0.702955351158331	0.935572660569741
Ppig	1224.26282049086	-0.0524138268140504	0.137416239877479	-0.381423817598145	0.702888790528143	0.935572660569741
Rnf151	22.8896789904843	-0.16378048730174	0.429282794101848	-0.381521201296698	0.702816542369559	0.935572660569741
Sos2	632.20143719395	-0.0549279390956441	0.143983089688544	-0.381488820766808	0.702840564918379	0.935572660569741
Tm7sf3	293.860417106347	0.0904939255377303	0.23702895891715	0.381784259405033	0.702621395160186	0.935572660569741
Atf1	1123.04319602499	-0.0589906074540173	0.154896275357747	-0.380839418622387	0.703322407637097	0.935813177846867
Clspn	18.5133513291262	0.252238421590518	0.662187385581554	0.380916983746216	0.703264849677331	0.935813177846867
Phf10	763.432919343635	0.0541697940012514	0.142320227677101	0.38061907913858	0.703485921983218	0.935813177846867
Rnf34	405.514245490096	0.0634609155047415	0.166741220155795	0.380595244807772	0.703503610307091	0.935813177846867
Slc5a5	70.8164164558475	-0.100192974843408	0.263294489360741	-0.380535783664403	0.703547739283959	0.935813177846867
Bclaf1	1043.56032897891	0.0471107548848754	0.123891117375229	0.380259342905038	0.703752912389013	0.93582932085735
Flcn	839.714293020631	-0.07108017884991	0.187011544300638	-0.380084444068555	0.703882732670189	0.93582932085735
Hdac3	777.758187966416	0.0358664109135316	0.0943664916783618	0.380075705641135	0.703889219075267	0.93582932085735
LOC498685	109.527612504298	-0.0963914996031569	0.253551553068763	-0.380165289608837	0.70382272324634	0.93582932085735
Abi3	101.749868726891	-0.14179346472997	0.374087673400257	-0.379038056616897	0.704659603601996	0.936087079773292
Epn3	801.710710854706	0.102031590303056	0.268985454909626	0.379320102409762	0.704450173571315	0.936087079773292
Fbxl20	47.7811965034161	0.160751360962724	0.423904358755132	0.379216107696552	0.704527391105851	0.936087079773292
Psma6	2436.26065936331	-0.0553174371953803	0.145883854404682	-0.37918820709199	0.704548108214564	0.936087079773292
Tbrg1	1002.20180281119	0.0455574696082448	0.120173168570302	0.379098513838331	0.704614709865054	0.936087079773292
Wbp5	1952.22176509919	-0.0881143774090165	0.232323904309226	-0.379273831812568	0.704484529763239	0.936087079773292
Zswim7	99.4643264140713	0.105354537815121	0.277817201767371	0.379222514462368	0.704522633904257	0.936087079773292
Eif4ebp3	279.648674890113	-0.0740965518562426	0.195735602190643	-0.378554289699805	0.705018871585822	0.936288297969499
Pigr	1518.74778153523	0.0441786559640724	0.116719948116067	0.378501333123804	0.70505820361789	0.936288297969499
Zc3hav1	150.085303713416	-0.093737132782597	0.247615258898251	-0.378559597658378	0.705014929289882	0.936288297969499
Narf	959.968928177625	-0.0442910848088566	0.117066976882693	-0.378339699104372	0.705178257690111	0.936338326741332
Cer1	11.6656600344057	0.235303811063746	0.622156719376939	0.378206653943065	0.705277082825382	0.936360159256312
Eif2c3	34.1169017127126	0.134535767167104	0.355936331967083	0.377977056805613	0.70544763787508	0.936477207672058
Rbpjl	39.8344738163857	-0.142876589469208	0.37830519925474	-0.377675458203255	0.705671701332931	0.936555880120282
Wscd1	136.875580129482	-0.129178927415525	0.341944733700358	-0.377777209836263	0.705596105215231	0.936555880120282
C1rl	83.5948092122949	0.110678882327931	0.293373852483135	0.377262259029351	0.705978716456748	0.936591499499093
Dad1	1799.17922975519	0.0399754604089601	0.105980680435127	0.377195732701774	0.706028151302853	0.936591499499093
Krcc1	1942.08757128076	-0.0540258077303222	0.143209220845657	-0.37725090194121	0.70598715567296	0.936591499499093
Rnf39	20.2055893419573	-0.184041492255388	0.487700797851705	-0.377365575504655	0.705901945791507	0.936591499499093
Jak2	297.268850259506	-0.0668757910133405	0.177459362639965	-0.376851297212311	0.706284116655005	0.936821714307479
Kif13a	654.94171857042	0.0517872815103468	0.137543293932282	0.376516222854484	0.706533157217968	0.937012444481331
Slc25a21	194.528375147447	-0.0806306002930838	0.214234778139319	-0.376365597562543	0.706645118152266	0.937012444481331
Ubash3b	185.939894549011	-0.177507221047034	0.471685856372442	-0.376325087235338	0.706675230872248	0.937012444481331
Wars	982.632648858725	0.0354322352053099	0.0941865075173381	0.376192260858461	0.706773968508182	0.93703405179797
Ift81	459.344633721362	-0.0521196211368341	0.138610325032291	-0.376015431207541	0.706905424014544	0.937099026049605
Cep78	222.353735140343	-0.0618919407747417	0.164753817505609	-0.375663166485563	0.70716732434091	0.937118317413872
Ighmbp2	235.263704951378	0.0716993251338558	0.190772465924525	0.375836862968592	0.707038180860805	0.937118317413872
Tep1	821.457507583901	0.0529687656841889	0.140980786179012	0.375716203035861	0.707127890727621	0.937118317413872
Ccdc17	441.535163848874	-0.0498569511101832	0.132941121975184	-0.375030316951063	0.707637919757355	0.937239654518371
Cdh5	185.804141529928	-0.156710629445823	0.417911076438072	-0.374985584927526	0.707671187319784	0.937239654518371
Hist3h2a	569.632900594076	-0.0824487404999368	0.219807853366824	-0.375094607572293	0.707590107287412	0.937239654518371
LOC360479	81.800588439325	-0.0964374571553725	0.257098104831971	-0.375099836766203	0.70758621842443	0.937239654518371
Sec62	2532.46312922111	-0.0450010027282829	0.11993299773116	-0.375217859801658	0.707498448720156	0.937239654518371
Dcaf7	1137.44987629598	0.0522101226243354	0.139430045535857	0.374453887780655	0.708066657446449	0.937255844966066
Dok7	8.43385250005321	0.483670285553703	1.29180326204835	0.374414819782053	0.708095718874152	0.937255844966066
Maoa	413.246621353922	0.0640172357938096	0.17090245965545	0.374583466632793	0.707970270949407	0.937255844966066
RGD1310769	380.049867499533	-0.0555713462981496	0.148373775452549	-0.374536174796751	0.708005448172386	0.937255844966066
Uxt	525.817126521419	0.0709450323720361	0.189384637464946	0.374608169499321	0.707951896392617	0.937255844966066
Aurkaip1	1290.70046834895	-0.0569035606800763	0.152227993276333	-0.373804840065005	0.708549518277463	0.937275779721657
Fam103a1	291.506389808214	0.0687587445453763	0.184372236747461	0.372934373191754	0.709197288405181	0.937275779721657
Fam129a	71.8309721357337	-0.140450365802951	0.376439737860115	-0.373101858484298	0.709072635523151	0.937275779721657
Gtl3	340.620296583157	-0.0499533753148841	0.133922339009554	-0.373002560172732	0.709146538509595	0.937275779721657
Herpud1	5523.52183211209	-0.0802294667571318	0.215182950756818	-0.372843045766207	0.709265263187222	0.937275779721657
Llgl1	190.804118911556	-0.0960621716273931	0.257645046279765	-0.372846957527309	0.709262351625441	0.937275779721657
Mff	393.724927069992	-0.0523853485634158	0.140094504017311	-0.373928648599539	0.70845740152956	0.937275779721657
Myo1c	1974.82182413194	-0.0796875920702851	0.213119460704671	-0.37391044349869	0.708470946327465	0.937275779721657
Nr3c2	526.306435990418	-0.0586782359230253	0.15732558955634	-0.372973246682238	0.709168355662606	0.937275779721657
Rnf31	337.254202955774	-0.0575078379220309	0.154116810878282	-0.373144484331752	0.709040911982461	0.937275779721657
Rom1	70.8390261283898	-0.110088070770436	0.294835694937903	-0.37338786537913	0.708859789565772	0.937275779721657
Rpp40	146.79179134382	0.0804929796147813	0.215789678456638	0.373015893023613	0.709136615346939	0.937275779721657
Tmem203	264.7122763021	-0.0600659914523933	0.161100514606986	-0.372847917953135	0.70926163677182	0.937275779721657
Upf3a	914.096535246792	-0.0500921616262625	0.134142204670452	-0.373425811431415	0.708831551870753	0.937275779721657
Bambi	522.796266937718	0.0709410661964767	0.191157396846045	0.371113372367231	0.710553091408101	0.937332503271896
Cdc123	697.561718013048	0.0391237270685935	0.105365824911432	0.371313251725404	0.710404228923179	0.937332503271896
Cers2	3169.59711905657	0.0326747492580976	0.0877337199923115	0.372430910953748	0.709572042915616	0.937332503271896
Chmp1b	1272.08979657726	0.0468277717092071	0.126264311665114	0.370871001407007	0.710733614822648	0.937332503271896
Dennd5a	781.145854334412	-0.0570326257262206	0.153304464510068	-0.372021949318215	0.70987650718389	0.937332503271896
Elf3	1226.74512912736	-0.0691300613966739	0.186576592694276	-0.370518404256371	0.710996266189234	0.937332503271896

Hrc	10.7061545469412	0.269232438813922	0.726978504391568	0.370344428600749	0.711125874163044	0.937332503271896
LOC680039	1094.01612785151	-0.0388697882474676	0.104877372942892	-0.370621299492629	0.710919615470177	0.937332503271896
Npat	344.685546616492	0.0592023789157791	0.159334905215756	0.371559382017475	0.710220935694179	0.937332503271896
Oxgr1	31.95529237309	0.162935476868221	0.439666756908424	0.370588574887772	0.710943993001841	0.937332503271896
Pdgfb	184.995198327946	-0.0805528010805076	0.217154476404782	-0.370946997796881	0.710677009232757	0.937332503271896
Rhoq	714.169099049687	0.043726895321766	0.118002389297039	0.370559406315878	0.71096572178796	0.937332503271896
Sh3kbp1	186.338541204677	-0.103680916081202	0.279231060640806	-0.371308678351415	0.710407634873356	0.937332503271896
Smc4	553.072994286006	0.0587766743316769	0.15810591742662	0.37175505691592	0.710075228556295	0.937332503271896
Snrpa	227.501977712472	0.0668857220654966	0.179789055330691	0.372023324459166	0.709875483339593	0.937332503271896
Tas1r1	526.85075140118	-0.188790743562668	0.509859727452716	-0.370279771861715	0.711174044116766	0.937332503271896
Tbcc	283.397551741264	0.0720892000165834	0.193469290981572	0.37261314005358	0.70943639169977	0.937332503271896
Tial1	744.362906520971	-0.040413553135898	0.108952763028441	-0.370927290071096	0.710691688295799	0.937332503271896
Timm9	80.9506908381726	-0.14058590624009	0.378100649164197	-0.371821382880086	0.710025842069314	0.937332503271896
Tomm22	1720.58762831031	-0.039924663878076	0.107577772445741	-0.371123727238477	0.710545379225619	0.937332503271896
Ttc7	1326.93266921131	0.0448860640119073	0.120542412740887	0.372367393279183	0.709619327592626	0.937332503271896
Unc50	637.833545866343	0.0604138758694605	0.162530102570872	0.371708839863167	0.710109642614985	0.937332503271896
Wdr12	528.434553443134	0.0589249851611983	0.159154219886846	0.370238283364978	0.71120495409263	0.937332503271896
Ascc2	576.726481900298	-0.0469991902646339	0.127062184467795	-0.369891250189754	0.711463521147043	0.937455875418189
Rap1gds1	707.925759685261	0.0469829450761049	0.126983326706123	0.369993024240397	0.711387688020646	0.937455875418189
Rbl2	1119.82591109045	-0.0473015927991129	0.127938371017803	-0.369721706027748	0.711589856975482	0.937513656471929
Nfya	87.5490115794393	-0.100347103571891	0.271691902057774	-0.369341532860822	0.711873171786167	0.93766954114284
Qpctl	244.801703465986	-0.0639622085067368	0.173166324588739	-0.369368632490432	0.711852975128695	0.93766954114284
Cdkl3	100.914263874791	0.0926220793727021	0.251166083883281	0.368768258598738	0.712300465709759	0.938034492196963
Map4k4	884.946600479974	0.0501982411004204	0.136172312871832	0.368637647710867	0.712397830076033	0.938034492196963
Plscr1	467.122018545995	0.0539014921672769	0.146203580401021	0.36867422821952	0.712370560528759	0.938034492196963
Nfatc2	24.3078096353093	0.174402165181849	0.473467392476098	0.368350952891973	0.712611564219171	0.938207232586019
Rer1	2006.70963203206	-0.0423806341669952	0.115102661665084	-0.368198559042107	0.712725184700771	0.938248140990152
Ephb3	89.9663318887181	0.13144140602586	0.357280699947248	0.367893944579899	0.712952315609332	0.938438450401351
Abhd12	687.990377352719	0.0627264755980858	0.170697268483909	0.367472052454072	0.713266934772822	0.93852650852372
Dpysl3	12.5467112257751	0.251706875996329	0.684946151987529	0.367484181443962	0.713257889095407	0.93852650852372
Pcdhb3	11.3922864887467	-0.251024645829476	0.683051859465617	-0.367504520119255	0.713242720808529	0.93852650852372
Mpv17l	540.936836595708	0.0534070313235076	0.14560271338092	0.366799698188221	0.713768432782433	0.938968982292261
Polr2f	740.535703777728	-0.0521325904989619	0.142115746712235	-0.366831907828787	0.713744405317822	0.938968982292261
Dcp2	22.4899897686836	-0.188216594530211	0.513971242358373	-0.366200633456792	0.714215369541826	0.939178232850198
Fbp2	50.7948212940728	0.149261553606619	0.408073397130857	0.365771340783469	0.714535706897783	0.939178232850198
Gripap1	806.827998655418	-0.0366634849714776	0.100316227524699	-0.365479104190304	0.714753802054744	0.939178232850198
Msto1	296.492348896271	-0.0784319218927528	0.214543423414734	-0.365575978253764	0.714681502694865	0.939178232850198
Myo9b	450.632602360557	-0.0720208107288066	0.197056211904148	-0.365483584774475	0.714750458034416	0.939178232850198
Nt5m	29.3229972326944	0.154463578149517	0.422410691592979	0.365671563773657	0.714610167514493	0.939178232850198
Rfk	4761.6694938059	0.0589618862250212	0.161321647366845	0.365492710912764	0.714743646887722	0.939178232850198
S100a16	1161.49638748847	0.05274109615873	0.144018228109483	0.366211255693522	0.714207443887677	0.939178232850198
Taf1a	50.7792879688621	0.107824750296567	0.294799053177015	0.365756772739097	0.714546578426885	0.939178232850198
Zfp518a	237.644164372471	-0.0810681904202006	0.221786638082282	-0.365523329634153	0.714720795262902	0.939178232850198
Abcg3l1	125.937160589805	0.120102686246684	0.329869956061928	0.364091012350748	0.715790048021295	0.939340759893125
Akap17a	1511.46153831781	-0.0513128404144495	0.141569383322407	-0.362457186788692	0.717010411503582	0.939340759893125
Cap1	2925.17893806128	0.0410122696523336	0.112623139163794	0.36415491485002	0.715742331659495	0.939340759893125
Cetn4	27.5591519423288	-0.160165738010079	0.440742004447894	-0.363400212354877	0.716305943165097	0.939340759893125
Cldn22	17.162665823972	0.169667860592255	0.468383117830366	0.36224162258065	0.717171478259882	0.939340759893125
Cntrob	62.2462728554235	-0.11462798060073	0.315578342628787	-0.363231455130514	0.716431992148513	0.939340759893125
Cops4	2003.06495173048	0.0469919955513027	0.129031992725234	0.36418871443278	0.715717093766635	0.939340759893125
Coq7	540.33259826182	-0.0503186553412312	0.138543779180095	-0.363196786164042	0.716457888221819	0.939340759893125
Des	199.532993994241	0.317563870361184	0.871835096203696	0.364247633232453	0.715673100287239	0.939340759893125
Dhh	41.32561088753	0.125797381454714	0.345976368553197	0.363601080561581	0.716155919763756	0.939340759893125
Etfb	5615.16267390387	0.0660746979297926	0.182101989185568	0.362844459993571	0.716721077647769	0.939340759893125
Galt	913.58867124018	0.051797177160205	0.142803607785162	0.362716166373962	0.716816921986995	0.939340759893125
Gls2	22.1204042052285	0.16520615229526	0.455178627548889	0.362947955585889	0.716643762426823	0.939340759893125
Il1b	48.5893218023058	-0.196062683005071	0.538399431498826	-0.364158413873619	0.715739718950618	0.939340759893125
Kdm4b	586.108444513487	-0.0414044629484135	0.113694590142579	-0.364172674324172	0.715729070755709	0.939340759893125
Maged1	2031.41248138454	-0.0329894143214653	0.0903710093060202	-0.365044216887679	0.715078400064372	0.939340759893125
Mapkbp1	317.619387172944	-0.081624134949535	0.224884641235274	-0.362960024753936	0.716634746479328	0.939340759893125
Mtrr	525.968885246506	-0.0449251451210834	0.123952979673073	-0.362436992152781	0.71702550013722	0.939340759893125
Ntpcr	729.702566086595	-0.0488529312536131	0.134125285237444	-0.364233568391882	0.715683602134239	0.939340759893125
Pold3	312.912711750024	0.0665247524386058	0.182712465288032	0.364095314097671	0.715786835847403	0.939340759893125
Rpl11	6928.90874738171	-0.0630334468850142	0.173231743115031	-0.363867763214494	0.715956758141829	0.939340759893125
Sart1	1230.79135313354	0.0473155189040899	0.130628354720634	0.362214765739646	0.717191546221409	0.939340759893125
Sdf4	2621.59145618665	0.0366351385276893	0.100327690622531	0.365154807215927	0.71499585103128	0.939340759893125
Selt	2043.13787432279	-0.0530448534667906	0.146225973425023	-0.362759448436765	0.71678458674936	0.939340759893125
Syngr4	17.5971608159576	0.178256988847609	0.491900768410314	0.362384042260568	0.717065062727908	0.939340759893125
Tbpl1	416.583454256254	-0.0578872121435374	0.159320709825962	-0.363337648989714	0.716352672402767	0.939340759893125
Tnfrsf1a	706.993028333444	0.0496169597713486	0.136866158313494	0.362521753972961	0.716962170196985	0.939340759893125
Tsc22d4	1671.29315588716	0.0504281421237128	0.138390704946984	0.364389661451837	0.715567054561696	0.939340759893125
Cdca7	26.8311144181447	0.153405465288832	0.42383121080415	0.361949430287991	0.7173898205962	0.939383951716177
Fam3d	103.565746198686	0.105812182587932	0.292331811080855	0.361959179867241	0.717382534795692	0.939383951716177
Prelid1	4159.81720964854	0.0525653197321559	0.145366831091058	0.361604633860587	0.717647501370514	0.939396694757258
RGD1564482	11.6222109986409	-0.31637352537318	0.87478819092088	-0.361657288766252	0.717608148080497	0.939396694757258
Rmrp	25.0530218222053	0.191256424046681	0.528849247976578	0.361646395033072	0.717616289791105	0.939396694757258
Sec63	2882.50955480283	-0.040664420284989	0.112565167675956	-0.361252251691665	0.717910884483884	0.939633246550418
Mrpl45	845.127689556402	0.0444904110160509	0.123373639002984	0.360615212257578	0.718387115761136	0.940040059606387
Nudt1	121.450798730225	0.0757756619935992	0.2100688385063	0.360718241374608	0.718310086910063	0.940040059606387
Hnrnpr	1116.33286589636	-0.0458520210217629	0.127305384790321	-0.360173460826373	0.718717419929557	0.940364014610262
Osbpl11	297.108031894717	0.0624236051958353	0.173420750963027	0.359954647002675	0.718881049774252	0.940469844812862
Pbx2	1018.03914643522	0.0337680338215985	0.0939332579045419	0.359489648021308	0.719228820644044	0.940816523816343
Ttll12	174.832054668862	-0.0682080447014264	0.189896121918035	-0.359186085595087	0.719455885133082	0.9410052463087
Vsig2	21.6182735217381	-0.274265801738154	0.763850868977062	-0.359056738529926	0.719552644173803	0.941023513252726
Slc6a19	99.6250008391341	-0.101295843576933	0.282271891893779	-0.358859123015518	0.719700480616292	0.941108566806807
Asmtl	760.968236097899	0.053017205723156	0.148158013812418	0.357842308754766	0.72046132643738	0.941111411920726
Ccnl2	540.227713874318	-0.0770137117250949	0.215102211346305	-0.358033100836449	0.72031854240818	0.941111411920726
Dr1	556.090937930853	-0.0667672243731289	0.186351412326118	-0.35828665605327	0.720128803110418	0.941111411920726
Gga1	2276.46413865393	0.0397732037238456	0.111190589195458	0.357702967594944	0.720565612041593	0.941111411920726
Hdac1	1400.4838485153	0.0316318845002306	0.0883999755887527	0.357826846552378	0.720472898390605	0.941111411920726
Lsm1	229.264805813043	-0.0741265314612893	0.207182313189735	-0.357784071043773	0.720504912028111	0.941111411920726
Msh5	53.8216843304437	-0.112585138157757	0.314496085839677	-0.357985816761963	0.720353927719804	0.941111411920726
Noc3l	267.913804542184	0.0616012298058599	0.171776350123728	0.358612985789309	0.719884630880441	0.941111411920726
Pskh1	333.180446773978	0.0528469234291797	0.147766094801091	0.357639034179778	0.720613462778997	0.941111411920726
Raph1	139.890146405095	0.0909362665274313	0.254077121893928	0.357908125885476	0.720412069438775	0.941111411920726
Stk36	147.112917818812	-0.0696859037024906	0.194688922261897	-0.357934611239712	0.72039224834517	0.941111411920726
Elp6	269.380838205213	0.0572018081749402	0.160123762754866	0.357234973690386	0.720915905718065	0.941232269824413
Qrsl1	306.112369513513	-0.0549201773091099	0.153747632816939	-0.357209904977861	0.720934671315456	0.941232269824413
Usp34	1032.28006772187	-0.0557040993434309	0.155953730927434	-0.357183499312052	0.720954437892956	0.941232269824413
Srek1ip1	611.416387611677	0.0562666544087941	0.157623756434876	0.356968109893011	0.721115679631689	0.941334651968965
Apbb3	428.824351719348	-0.0719256365763503	0.201888462348049	-0.356264225007336	0.721642698268569	0.941352405447387
Bcas2	878.272259096613	-0.054527371236092	0.15295777079079	-0.356486440369691	0.721476305017512	0.941352405447387
Fkbp7	34.0153011043253	-0.203241655350206	0.56999466414622	-0.356567645514079	0.721415502482606	0.941352405447387
Golph3	2308.93660571661	-0.0481148855529161	0.134911463384287	-0.356640454012895	0.721360988451862	0.941352405447387
Hat1	665.738483178134	-0.0460535741433451	0.129300178675281	-0.356175641945571	0.721709032295313	0.941352405447387

Mpc2	3206.08686251057	0.0785160725087882	0.220326823831498	0.356361840757238	0.721569602680467	0.941352405447387
Pcnp	606.224588705741	0.0496222060737948	0.139108989957775	0.356714588243771	0.721305483257922	0.941352405447387
Kdm1a	683.542540476037	-0.041914365310207	0.117767485697555	-0.355907787806979	0.721909623367049	0.941505998759597
R3hdm2	2001.34210367043	0.0307322061889305	0.086407888699619	0.355664357171893	0.72209194071091	0.941635727182217
Dcaf17	23.136975631488	-0.17723498837852	0.498761706501109	-0.355350032026017	0.722327377839431	0.941759512818352
Exosc9	427.642309568714	-0.0519491482525438	0.146309477392105	-0.355063453021035	0.722542055357601	0.941759512818352
Il34	1013.63235233706	-0.0340930476907369	0.0959841932933278	-0.355194397337366	0.72244396171721	0.941759512818352
Jak1	1388.84452822012	-0.0475615044243044	0.133981893687867	-0.354984566310928	0.72260115355348	0.941759512818352
Ppil3	268.226551143649	0.0758894858459822	0.213660013937725	0.355188059980664	0.722448709083507	0.941759512818352
Brms1l	599.928964110292	-0.0476048981057237	0.13431653319728	-0.354423219335203	0.723021735954347	0.941760545701424
Cuedc1	190.41977695837	-0.0811040181333493	0.229208358980147	-0.353844067878756	0.723455745853323	0.941760545701424
Drg1	831.274728911992	0.0387545705211536	0.109303467031641	0.354559389318684	0.722919704556486	0.941760545701424
Eif3g	2262.84267071874	0.0450165931984767	0.127254306424208	0.353753004227706	0.723523996074114	0.941760545701424
Gamt	3585.74046983709	0.0703388296831227	0.19883522671611	0.353754366591941	0.723522974995625	0.941760545701424
Igsf8	153.727606457744	-0.0679117516340541	0.19163820484752	-0.354374805838373	0.723058013100201	0.941760545701424
Leprel1	1222.96170927005	-0.0621527369039927	0.175644225680002	-0.353855850731045	0.723446915023135	0.941760545701424
Mefv	175.120649097165	-0.0714677977057745	0.202057074694587	-0.353701041222136	0.723562942199003	0.941760545701424
Pkp2	550.248617735686	0.0620319659710001	0.175412820025791	0.353634164035899	0.723613067513342	0.941760545701424
Rbm14	223.354219422821	-0.062449899818428	0.176549031752156	-0.353725529948512	0.723544587879045	0.941760545701424
Rsl24d1	563.576534264026	0.0464658977820865	0.130944857563756	0.354850878809522	0.722701309691566	0.941760545701424
Selp	15.8975300284953	0.189996156841346	0.537401409191239	0.35354607113383	0.723679096089762	0.941760545701424
Sgsm1	123.74829563757	0.104327859938691	0.294333958738073	0.354454037128391	0.722998643925168	0.941760545701424
Mvd	244.384909436217	0.0750201333506776	0.212367515185782	0.353256162012579	0.723896407190013	0.941827674464479
RGD1307554	176.68819480253	0.0628130094319479	0.177804754837705	0.353269570823805	0.723886355675047	0.941827674464479
Fbl	881.431742954075	-0.0538161113413087	0.152400184359553	-0.353123662989424	0.723995733685943	0.941849091115306
Arf5	2579.80567341774	-0.0555508284127371	0.158036946008928	-0.351505327175829	0.72520927664405	0.941918482269029
Ddx18	469.604769730691	-0.047920036338633	0.136264137097106	-0.351670199947648	0.725085611709785	0.941918482269029
Dusp22	325.067225543317	0.0564511768527496	0.160536817929773	0.351640063511439	0.725108215394342	0.941918482269029
Fxyd2	8297.71215043143	0.0492655303802528	0.139676446417281	0.352711796755429	0.724304514535641	0.941918482269029
Myl9	485.364717229739	-0.230516437750229	0.655060402626002	-0.35190104122633	0.72491247829984	0.941918482269029
Ndufb10	2719.71532598025	-0.0501823832021336	0.142296012600915	-0.35266190727969	0.724341920296004	0.941918482269029
Per1	2805.45380260122	-0.155403601089619	0.440732729396797	-0.352602815094557	0.724386226846841	0.941918482269029
Perp	2592.75709756896	0.0584090458442682	0.16570666294385	0.352484594201623	0.724474870103582	0.941918482269029
Plk2	183.872252559207	0.0758589406968689	0.215237134329611	0.35244355456201	0.724505642916566	0.941918482269029
Rnf167	1897.19518263866	0.0349548228242155	0.0994322553115547	0.351544101204285	0.725180193042703	0.941918482269029
St6gal1	7613.9117997897	-0.0467479616056884	0.132832534012127	-0.351931565209924	0.724889586040643	0.941918482269029
Stap2	128.274438040193	-0.0985869029845225	0.280362098008435	-0.35164133698827	0.725107260224512	0.941918482269029
Syncrip	466.197097481243	0.0523190200343194	0.148779596375665	0.351654536702836	0.725097359818999	0.941918482269029
Tmem132a	667.337333546778	-0.079086846958596	0.224434263313345	-0.352383124532899	0.724550956063895	0.941918482269029
Mfsd8	40.4381852528641	0.144726088771765	0.412366836753589	0.350964422627046	0.725615039340207	0.942337812744605
Stard5	327.565743973587	-0.0653004819927555	0.186122156956124	-0.3508474383743	0.725702805894253	0.94234412107781
Gemin7	118.444612385223	0.0795355136584302	0.226803079854998	0.350680924215313	0.725827738099939	0.942398683029918
Abce1	1527.70448112615	0.0349654493238543	0.0998961100470492	0.350018126905905	0.726325093798996	0.94256164051322
Aph1b	792.814467213035	0.0465438926136851	0.133103654195741	0.349681553785429	0.726577698699607	0.94256164051322
Arhgef3	637.298598774645	-0.054501144033913	0.15632703791222	-0.348635429685019	0.727363025597524	0.94256164051322
Cdk5rap3	1209.92914272979	0.0350738324722366	0.100397147341851	0.349350886961068	0.726825899759907	0.94256164051322
Cebpb	1245.40407557285	0.152098167792921	0.43597207416946	0.348871353934018	0.727185891872514	0.94256164051322
Gtf2h4	270.37730910613	0.0541832694356284	0.155204193214501	0.349109571806118	0.727007050869891	0.94256164051322
Gusb	351.021486404721	-0.0591593725256083	0.169507584257247	-0.349007230471925	0.727083881347356	0.94256164051322
Mllt1	798.636280861075	-0.053412765958925	0.152631815402021	-0.349945165876718	0.726379849994523	0.94256164051322
Mrps14	1044.74734504423	0.0613996219523364	0.17572315625378	0.349411103586502	0.726780698561881	0.94256164051322
Mrps36	1974.00594290607	0.0594397181436913	0.170473907090477	0.348673407902504	0.727334510278093	0.94256164051322
Pde5a	55.4979163445727	0.119091267759462	0.341462239963427	0.348768483953651	0.727263125646122	0.94256164051322
RGD1306820	383.576864582062	-0.0556952729752752	0.159370916665836	-0.349469490045384	0.726736872073979	0.94256164051322
Spint2	5708.78515436253	-0.0341591265889977	0.0979648855584964	-0.348687454634964	0.727323963615908	0.94256164051322
Supt16h	1306.0400646274	0.0369110729324002	0.105797902971639	0.348882840733573	0.727177267867943	0.94256164051322
Thap1	92.3049925407372	0.0839965332340846	0.239872880601089	0.350171028186265	0.726210348139025	0.94256164051322
Tyw1	2158.87032621698	0.0323909783342611	0.0927682727102977	0.349160088766702	0.726969127398609	0.94256164051322
Uxs1	616.707845239888	-0.0583981263452657	0.167463555328762	-0.34872140526707	0.72729847278717	0.94256164051322
Abhd14b	9506.83649205866	0.0459444005361394	0.132162042221704	0.347636883962998	0.728112902573854	0.942637442882923
C2cd3	202.891078577958	-0.0918280512951434	0.265001678570355	-0.346518753354855	0.728952893059661	0.942637442882923
Capza2	3281.18160340305	0.0442628753207485	0.127260259928158	0.347813805706007	0.727980020816092	0.942637442882923
Chek2	75.1414358794581	0.0943639007833395	0.270969426709808	0.348245563822953	0.727655771787689	0.942637442882923
Cntnap1	399.809763489256	-0.0555197328850149	0.160350921255345	-0.34623893926125	0.729163153072907	0.942637442882923
Eif2b3	225.707978538822	-0.0578162709465235	0.16645093430197	-0.347347229914823	0.728330472555397	0.942637442882923
Gan	17.9908896679456	0.182624978734661	0.526011557230521	0.347188148671469	0.72844997373693	0.942637442882923
Hnrnpab	2296.42694045233	-0.0595092336191169	0.171602587074568	-0.346785177505848	0.728752713563747	0.942637442882923
Mafg	275.630803029823	0.0526345347093958	0.151336488485972	0.347798044185984	0.727991858591152	0.942637442882923
Mdm2	720.509894123868	0.0459836534068427	0.132038416385722	0.348259655527157	0.727645189782053	0.942637442882923
Mrpl50	1159.45966773693	0.0593549853807753	0.171344368478014	0.346407564532192	0.729036440961976	0.942637442882923
Pde1b	39.3190184970173	0.173387958763848	0.499351043989619	0.347226587089008	0.728421098349526	0.942637442882923
RGD1308299	426.076680238306	0.0558331121345036	0.160495487187738	0.347879638940834	0.727930577109817	0.942637442882923
Slc46a1	73.4355326088513	-0.0859775915953229	0.248302771331897	-0.346261103467105	0.729146497535344	0.942637442882923
Slco3a1	128.649241440161	-0.150714242220194	0.434342325693808	-0.346994141037135	0.728595720413847	0.942637442882923
Spag9	2600.35941585125	0.0413253349977624	0.118882937728645	0.347613676001926	0.728130334133727	0.942637442882923
Spg11	788.780233781542	0.0351616710422553	0.101540528557757	0.346282135238791	0.729130693096154	0.942637442882923
Spred3	35.1959184692509	-0.144617592321693	0.417400397302152	-0.346472100305658	0.728987948029466	0.942637442882923
Tcf19	153.297383603759	0.069946529893069	0.201297952750215	0.347477601920094	0.728232542559454	0.942637442882923
Trim25	119.18286205104	0.116594496465	0.336386674617749	0.346608546838224	0.728885424084183	0.942637442882923
Tspan7	692.067604705775	-0.0864014151592118	0.249077087986511	-0.346886242559134	0.728676782524993	0.942637442882923
Kif15	41.1846639759774	0.165759970742361	0.479448180702825	0.345730732567121	0.7295450856631	0.942809487623286
P2rx3	29.6701102879118	-0.131356298679852	0.379831550732689	-0.345827771354085	0.729472152914415	0.942809487623286
Tmem68	319.675226144905	0.0604404073631055	0.174800238211466	0.345768449640138	0.729516737843574	0.942809487623286
Snip1	257.510462740434	-0.101265064582807	0.293117325676387	-0.345476216218646	0.729736387568329	0.942842307730092
Tmem127	1957.51408988636	-0.0460960545861433	0.133427103456774	-0.345477443427204	0.729735465122242	0.942842307730092
Minos1	3419.16950052843	0.0455436412197443	0.13203741740919	0.344929809393366	0.730147139670961	0.943265786485581
Atp11a	14858.0097081804	0.0399255799559401	0.11583841636442	0.344666141069615	0.730345375581096	0.943281586653505
Cript	966.685566253006	0.0364072743405387	0.105740860636455	0.344306582350504	0.730615734583738	0.943281586653505
Gfer	559.818060285945	0.0557381114742093	0.162040708325901	0.343975980172262	0.730864350153459	0.943281586653505
Nudcd2	449.125877213648	-0.0561282553071468	0.163138358572492	-0.344053083519323	0.730806365270401	0.943281586653505
Osgepl1	265.168883002532	0.0699767272846766	0.202989610991096	0.344730584698475	0.730296922737062	0.943281586653505
Pdcd10	770.074615095372	0.0502670083325589	0.146077565315589	0.344111761610765	0.730762237966879	0.943281586653505
Rfxank	392.964975199311	-0.0596071729731502	0.173017000967673	-0.344516276665131	0.730458057391661	0.943281586653505
Surf6	551.77031583972	0.0395979803100418	0.115086197673794	0.344072365847729	0.730791864408082	0.943281586653505
Taf12	280.206316376788	-0.068898449968029	0.200332659299187	-0.343920208562363	0.730906293652773	0.943281586653505
Nup93	277.727045356275	-0.0611605296307808	0.178075753490008	-0.343452314153553	0.731258209178986	0.943521490013435
Nupr1l	59.984640709851	0.107965496600408	0.314353110823065	0.343452928834469	0.731257746824402	0.943521490013435
Mb21d2	366.424159567227	-0.0563701937632796	0.164314186329393	-0.34306346288492	0.731550717343878	0.943791765417765
Tmem128	612.734731421514	-0.0458102705871617	0.13361685888731	-0.342847983171025	0.731712825882393	0.94389376676646
Cyp2j3	237.14978460724	0.151186580274047	0.441448872847113	0.342478120510249	0.731991106886251	0.94395889921659
Pola1	94.2431026347413	0.0933665369582015	0.272571750126315	0.342539301724899	0.731945072311884	0.94395889921659
Sox17	227.297664865892	-0.103383675642498	0.301894459488755	-0.342449728350674	0.732012470323335	0.94395889921659
Guk1	1918.60571196011	-0.0421306786231729	0.123087190353283	-0.342283209993257	0.732137769814984	0.944013373989463
Gar1	244.036532102182	-0.0764788667273228	0.223661528586821	-0.341940195126742	0.732395899528295	0.944131994333515
Mrpl53	1377.22362705614	-0.06170288214443	0.1803993503902	-0.342034946417313	0.732324593108511	0.944131994333515

Jmjd7	66.2080018171745	0.0966180147235945	0.282690773674756	0.341779865920762	0.73251656279484	0.944180455060802
Ranbp6	552.653026978095	-0.0472942938360578	0.138517028480175	-0.341433066785913	0.73277758537151	0.944302725094398
Yeats4	522.134178844126	0.055962135842067	0.163876339023387	0.341490029467162	0.732734709596074	0.944302725094398
Slc9a9	56.7546043533015	-0.162564052834197	0.476300141940634	-0.341305908857903	0.732873300074672	0.944319003361152
Cep57	378.064725325334	-0.0576309888692334	0.168953059961049	-0.341106511373751	0.733023399520609	0.944350713182009
Dhrsx	570.541399909742	0.0450063630128684	0.13208775303088	0.340730779199087	0.733306265289136	0.944350713182009
Fam117b	732.004436363743	-0.0809045753142408	0.237450828478304	-0.340721385697895	0.733313337545699	0.944350713182009
RGD1304978	37.0466335840633	0.127782945532257	0.374705984191725	0.341021896962487	0.733087097373117	0.944350713182009
Tbc1d24	53.2184164683292	-0.121148528567651	0.355391720682292	-0.340887312554906	0.733188416498543	0.944350713182009
Ccl24	229.000992571728	0.114107114878915	0.335180630328864	0.340434692681849	0.733529196230541	0.944404633064071
Ercc6l	13.7430859741919	-0.218294159117806	0.641410208755538	-0.340334712697104	0.733604478736819	0.944404633064071
Vrk1	232.272516423312	-0.0556258460468882	0.163356135875886	-0.340518865414099	0.733465818189073	0.944404633064071
Ccl21	37.4435740519927	0.24734286828107	0.727936364411123	0.339786388445043	0.734017399145798	0.944592700753561
Fam118b	216.111108974683	-0.0586853325378121	0.173235563742611	-0.338760305736097	0.734790306386274	0.944592700753561
Fig4	657.061996424326	-0.0525110008931316	0.154797126982412	-0.339224647877979	0.734440502614571	0.944592700753561
Golga2	1465.16635748467	-0.0295367392982451	0.087044598650045	-0.339328801055134	0.734362048261047	0.944592700753561
Katna1	287.110568094757	0.0648643895234696	0.19091045922742	0.339763414670752	0.734034701429049	0.944592700753561
Kctd1	427.316296392943	0.0958899344826856	0.283276111709073	0.338503426583197	0.734983845305783	0.944592700753561
Mrrf	1224.56695879087	0.0389134962038235	0.11476509764227	0.33907082382415	0.73455637708976	0.944592700753561
Ndufs3	4395.35435264553	0.0375715727397312	0.110626085243082	0.339626704291074	0.73413766517561	0.944592700753561
Papolb	12.1943767214647	0.2381219069116	0.70332447397036	0.338566217619829	0.734936535477084	0.944592700753561
Rhobtb2	145.01393339119	0.0869330645345145	0.256829325770712	0.338485740573588	0.73499717098931	0.944592700753561
Rpl36	4244.17717045782	-0.0558674385964891	0.164313440086682	-0.34000528847194	0.733852545319095	0.944592700753561
Tceb1	388.835583520434	0.0745700953288822	0.220187946558409	0.338665655838255	0.73486161593229	0.944592700753561
Tjp2	982.941054999864	0.042673882848971	0.125698671708357	0.339493506725211	0.734237987836215	0.944592700753561
Tm9sf2	3946.33813382879	-0.0403265640714014	0.119019504087666	-0.338823156595395	0.734742955604227	0.944592700753561
Zfp637	623.15282793607	-0.0393023091020603	0.115985027918266	-0.338856745628897	0.734717650603816	0.944592700753561
Mospd1	550.888542044952	-0.051733017043501	0.152919592261494	-0.338302085942244	0.735135551943033	0.944663729042907
Cklf	43.655677401506	-0.135339196308823	0.400249298400119	-0.338137248084638	0.73525976206759	0.944676337429262
Slc30a7	143.008942025167	0.0785174257957484	0.232253029562981	0.338068467582492	0.735311592225645	0.944676337429262
Vcpip1	194.65656400585	-0.0625501297945425	0.185084870082352	-0.337953770973885	0.735398025553862	0.944680601090099
Gatc	1068.98308916388	0.051063672456991	0.151259091186124	0.337590765993412	0.735671601740357	0.944832862057799
Rhbdl3	478.368185073675	0.0912929526464033	0.270436824377285	0.337575893581124	0.735682810945937	0.944832862057799
Crebl2	984.188434539394	0.0374455989701797	0.110982306278361	0.337401521250246	0.735814238026593	0.944894885257061
Kdm6b	1044.86299859988	-0.0507269325501863	0.150547664256071	-0.336949316356734	0.736155107759592	0.94489960391456
Mical3	260.633233975586	-0.08115786809006	0.240826609341707	-0.336997096425112	0.736119088933478	0.94489960391456
Radil	22.6724917250384	-0.181488517282193	0.538789052542313	-0.3368452206403	0.736233581934484	0.94489960391456
Sorbs2	327.770640853807	0.0703571939111574	0.208705708301576	0.337111976877472	0.736032489107204	0.94489960391456
Tmem115	874.976547107977	0.0443577327752612	0.131575785093265	0.337126871360252	0.736021261510316	0.94489960391456
Adrbk2	52.9320483416186	0.106632673854677	0.31876224439597	0.334521028538801	0.737986425498591	0.94501506446789
Btbd7	126.075889384483	-0.10760320692647	0.320268583578504	-0.33597802732997	0.736887435974237	0.94501506446789
Cdc25b	87.7356448932439	0.0958803404826104	0.285893839044319	0.335370432616239	0.737345669121529	0.94501506446789
Emc1	1183.61443243948	0.0468014150826024	0.139758254780638	0.334874066337341	0.737720085802283	0.94501506446789
Ergic3	2156.05043594948	0.0287865333000053	0.0855844478518857	0.336352386707266	0.736605149800497	0.94501506446789
Fbxo18	1779.11465446926	0.0346797968593942	0.103122138214941	0.336298271735891	0.736645953068099	0.94501506446789
Gla	968.681475573755	-0.0380946066619359	0.113453108279373	-0.335774023644461	0.737041280166772	0.94501506446789
Gtf3c3	227.459551026906	-0.060914307492336	0.181692586503524	-0.335260280370078	0.737428753268872	0.94501506446789
Map4	2512.65742175496	-0.0318363350584564	0.0948184127081871	-0.335761105350243	0.737051022524762	0.94501506446789
Mfsd4	132.206542689692	-0.154489149950979	0.459938936483597	-0.335890566543692	0.736953391011563	0.94501506446789
Pbxip1	2414.26261543546	0.0530794857140304	0.158169432066962	0.335586244575745	0.737182898291874	0.94501506446789
Pfkfb2	48.3203674579362	0.124232094987953	0.371205249745482	0.334672246885337	0.737872339106422	0.94501506446789
Polg2	353.11095537488	0.0459820243515077	0.136632002168451	0.336539197418899	0.736464298232379	0.94501506446789
Ppapdc3	47.8327615536821	0.171783930128316	0.511810290773754	0.335639851767368	0.737142468221701	0.94501506446789
Ptpn2	316.755900928533	-0.0554403459723039	0.165372823015784	-0.335244600420303	0.73744058037894	0.94501506446789
RGD1560880	28.3103898912059	-0.14081132089262	0.42072105721437	-0.334690452208273	0.737858604524712	0.94501506446789
Slc35f5	698.594573252108	0.0389217672895136	0.115978391972841	0.33559498995837	0.737176302551933	0.94501506446789
Smim8	125.895133854504	0.109738101654148	0.327853701727227	0.334716677213086	0.73783881982797	0.94501506446789
Tti2	231.594190431011	-0.0523457876870479	0.156175120565658	-0.335173665929979	0.737494085792955	0.94501506446789
Xpo1	1382.25260326272	-0.0359539524486465	0.107462782833592	-0.334571202239588	0.737948571406163	0.94501506446789
Dph3	1124.19327728895	0.0370692876658998	0.111057828222246	0.333783653608979	0.73854281924514	0.945246857384873
Gnb2l1	7382.54127761543	-0.0378367840374939	0.113266494370502	-0.334050985225405	0.738341085617238	0.945246857384873
LOC499770	623.539117543483	0.058340734384196	0.174814140392299	0.333730064703427	0.738583260640072	0.945246857384873
Timm10	567.480348964649	0.0514275991370744	0.15407385475151	0.333785373384841	0.738541521411341	0.945246857384873
Zdhhc15	48.2240669550992	0.109897162640832	0.329077684057438	0.333955074941058	0.738413459308386	0.945246857384873
Cirbp	917.038983276254	-0.0564774357348638	0.169946419790751	-0.332324951619473	0.739643900852682	0.94532695120787
Crtc2	723.437097322392	-0.0455159351872969	0.136723741010851	-0.332904401611455	0.739206446773987	0.94532695120787
Ggact	1398.69451382983	-0.0427205161372526	0.128495453318404	-0.332467142097189	0.739536546761774	0.94532695120787
Hmox2	2198.76634380031	-0.0354165394602206	0.106352784472813	-0.333009987804073	0.73912674387083	0.94532695120787
LOC691083	193.520280792166	0.0712339733652622	0.213979397554236	0.332901083840125	0.739208951275707	0.94532695120787
Mkks	1050.41133509846	-0.0495584909832719	0.148770814392473	-0.333119712933287	0.73904391961504	0.94532695120787
Ncor1	1729.99491303529	0.0504086085087895	0.151178532540679	0.333437609570828	0.738803977525329	0.94532695120787
Ppp2r5e	813.493130816503	-0.0362509155652648	0.108928299943488	-0.332796119870336	0.739288187351494	0.94532695120787
Rnf114	973.312518075866	-0.0440693100387206	0.132592417978882	-0.332366742461394	0.739612348158197	0.94532695120787
Satb1	76.8559272640148	0.118866946202905	0.357670204434469	0.332336730119445	0.739635007869256	0.94532695120787
Stx12	1254.548306797	-0.0301063620588323	0.0904631198014272	-0.332802606464577	0.739283290616957	0.94532695120787
Timm10b	313.156830773013	0.0536206439957255	0.161313511440983	0.33240020328578	0.739587085064236	0.94532695120787
Snapap	630.977537598184	-0.055644500326779	0.16750813141132	-0.33218984569855	0.739745910789408	0.945351025638904
Ap4e1	80.205954647993	0.0977102968274747	0.296329947908047	0.329734802429739	0.741600354527824	0.945568015613475
Bcor	323.638033117997	0.0708990950175502	0.21478299566644	0.330096406363832	0.741327118498584	0.945568015613475
Cox7b	6049.44478935604	-0.0533951996751907	0.162083044929156	-0.329431124017498	0.74182984590719	0.945568015613475
Efha2	75.7891913368083	-0.0832044298280253	0.251865698108462	-0.330352368158504	0.741133727746226	0.945568015613475
Emx2	910.720411901707	0.0434008841140369	0.131425303384079	0.330232329669438	0.741224420237067	0.945568015613475
Itgb3bp	136.825298225238	0.0796957457204531	0.240782698675383	0.330986180314795	0.74065492429819	0.945568015613475
LOC100361444	140.830763909809	0.270642508627502	0.821646155435586	0.32939058600478	0.741860482434842	0.945568015613475
Lrrc46	65.0069793950265	-0.0942108972072661	0.28505208135012	-0.330504154753215	0.741019053808088	0.945568015613475
Mettl4	57.4352152017072	-0.0949028052598813	0.287394079802842	-0.330218372365174	0.741234965610541	0.945568015613475
Myl6b	277.958699330219	0.0720484487955656	0.218437645237589	0.329835311661598	0.741524404230967	0.945568015613475
Nsl1	20.2757949319648	-0.149479033227736	0.453660468428596	-0.329495390562696	0.741781277423749	0.945568015613475
Pld3	693.866306407327	-0.0384612708502736	0.116587063896109	-0.32989312506014	0.741480718391783	0.945568015613475
Rabac1	2109.12480383484	-0.0347354817035965	0.105169006958087	-0.330282492041021	0.74118652070202	0.945568015613475
RGD1560010	44.9095424897115	-0.118129916919434	0.357286412592462	-0.330630868558044	0.740923326630537	0.945568015613475
Rmdn1	1355.68149135918	0.04769816297291	0.144802050614981	0.329402537949799	0.741851449732114	0.945568015613475
RT1-A1	3015.81820366511	0.179927741904579	0.546286154549458	0.329365370888765	0.741879538918202	0.945568015613475
Scfd2	210.83387644031	0.0605454486478162	0.18370375913091	0.329581979891171	0.741715840456008	0.945568015613475
Scmh1	243.189780927929	0.0679505269284547	0.204849489761039	0.331709524918613	0.740108607694849	0.945568015613475
Slirp	639.698083476699	-0.0507818916923344	0.15405828478106	-0.329627788369208	0.741681222997311	0.945568015613475
Sox18	78.3469799412779	0.132934658920501	0.401498580210107	0.331096211724922	0.740571813022421	0.945568015613475
Spast	468.364750783343	-0.0456991946917154	0.138026674430105	-0.331089587432296	0.740576816539025	0.945568015613475
Tmigd1	2522.48641819452	0.150831205937956	0.456144142246679	0.330665664574921	0.740897040342603	0.945568015613475
Wdr77	391.520100082297	0.0567583585382811	0.172168218506909	0.329668036473314	0.741650807943591	0.945568015613475
Wdr8	129.904726681009	-0.0738885820541924	0.224365777004665	-0.329321980565049	0.741912331800191	0.945568015613475
Doc2a	29.0274953482826	-0.228597055478227	0.69524656137456	-0.328799980004607	0.742306878193791	0.945704993534128
Golga7	1730.96615027031	-0.0372643290548255	0.113355205865678	-0.328739459032719	0.742352626457108	0.945704993534128
Med17	318.158402755044	-0.065413524309927	0.198836665330825	-0.32898119771367	0.742169899792664	0.945704993534128
Polk	111.273590088851	0.0889774950116452	0.270539095467547	0.328889600439722	0.742239135096912	0.945704993534128

Insig2	131.708604565145	0.0758564114304318	0.231068750051466	0.3282850295141	0.742696162254333	0.945718639948774
Lrrcc1	418.566739517949	0.178441433294884	0.543314180866074	0.328431393066969	0.74258550997079	0.945718639948774
Rmdn2	297.188134925222	0.0900408469071357	0.274246148380501	0.32832128158901	0.74266875483419	0.945718639948774
Zc3h12c	35.1176784156643	0.181049809447737	0.551446188936479	0.32831818059512	0.742671099245651	0.945718639948774
Bri3bp	531.41439720671	0.058067178604856	0.176955875762902	0.328144959044244	0.742802061884199	0.945747533928062
Nfatc1	120.648573600848	0.0812529334184321	0.247805520037575	0.327889925156275	0.742994891572272	0.945787484941132
Spry4	287.900447284011	0.0658407345362955	0.200805361768566	0.327883349111856	0.742999863895774	0.945787484941132
Kank1	2468.13586953804	-0.0365150384285093	0.111463516800514	-0.3275963245791	0.743216901284301	0.945874683887799
Tmed2	4959.46045005547	0.0434182034302706	0.132545265071341	0.327572647781129	0.74323480571898	0.945874683887799
Ndst1	501.861955006607	0.095702316537506	0.292471864272345	0.327218882320897	0.7435023402915	0.946109225229869
Arhgap1	1012.47118679242	-0.0333091498689914	0.101855466484553	-0.327023683839718	0.743649972096815	0.946191154466854
Usp7	1503.84456721605	0.0388211000083209	0.118903098125118	0.32649359537689	0.744050934207685	0.946595356861937
Chrm3	46.5882487183936	-0.187960397850738	0.576128633473058	-0.326247276962547	0.744237274565175	0.946664032878978
Crtc1	113.738314488939	0.0769184371201604	0.235840782036228	0.326145615936538	0.744314185694498	0.946664032878978
Ston1	36.2196985259329	-0.198716514539632	0.609387968216003	-0.326091956034805	0.744354782847038	0.946664032878978
Sept1	102.335079408165	-0.0747515145507068	0.229778989290132	-0.325319189459578	0.744939508983897	0.946886589823454
Atp6ap2	1427.23890983512	-0.0464782702220871	0.14288343755451	-0.325288018104658	0.744963098372881	0.946886589823454
Coq10b	2553.81322877067	0.147407377960473	0.453143506918259	0.325299547957692	0.7449543729563	0.946886589823454
Nhej1	126.918300818461	-0.0869504667800118	0.267544460452198	-0.324994457493345	0.745185266486084	0.946886589823454
Pcolce	337.382708406407	-0.235307391786557	0.723122182970407	-0.325404748088314	0.744874762441566	0.946886589823454
Rnf103	1082.23636996785	0.0381695578521921	0.117452039683589	0.324979948879724	0.745196247226007	0.946886589823454
Rpl3	10514.0265636388	-0.0434702215682542	0.133648988895752	-0.325256643745816	0.744986841629648	0.946886589823454
Ttc30b	268.809560874919	0.0648963506725748	0.199647920050798	0.325053978303719	0.745140219130547	0.946886589823454
Cgrrf1	672.408524291583	-0.0358971785415497	0.110648976322435	-0.324423955237906	0.745617086006273	0.946892044642156
Lats1	730.07924460469	0.034709511937761	0.106936651629997	0.324580126726397	0.74549887011406	0.946892044642156
Trib2	1538.61383840204	0.0726152296593402	0.223569535997059	0.324799303874278	0.745332971434614	0.946892044642156
Use1	2073.30429302487	0.0456093286188235	0.140523599423605	0.324567039315122	0.745508776557904	0.946892044642156
Wdr74	330.578153240869	0.0602385020370028	0.185627260811825	0.324513230295781	0.745549507440733	0.946892044642156
Leng1	328.046311915568	-0.0621246179140132	0.191801153095193	-0.323901170099745	0.746012858093239	0.947288810757206
Rnf139	642.827389625638	0.0363163849683672	0.112191835217352	0.32369900089441	0.746165927337378	0.947377338038052
Btg3	254.008889555059	-0.0735861430692765	0.227420543455362	-0.323568583344451	0.746264676256393	0.947396884879947
Abcd1	182.516505048543	0.0637871351702248	0.197528947636997	0.322925505012297	0.746751660050676	0.947406664106522
Dnase1	7632.14909118238	0.0704468168082829	0.2181397727299	0.32294347760007	0.746738048578219	0.947406664106522
Krt23	37.9701165606849	-0.167102374958166	0.517545626394337	-0.322874673142044	0.746790157806086	0.947406664106522
Nudc	1643.33146121966	0.0445430511106874	0.137994788673112	0.322787922203373	0.746855860495727	0.947406664106522
Sult2a1	66.7241461259489	0.436087703786561	1.34936503525691	0.323179934556057	0.746558976141322	0.947406664106522
Zbtb7a	406.784581549763	0.0468025864653504	0.144987128925424	0.322805112510532	0.746842840900611	0.947406664106522
Zfp593	218.818885407488	0.0759944519251745	0.235369493959956	0.322872988536499	0.746791433660816	0.947406664106522
Asf1b	22.1940614431356	0.17129658568704	0.532111927579735	0.321918334862682	0.747514562756268	0.947573236418764
Cabin1	564.411117320636	0.0539519173267035	0.167525466653274	0.322052034263945	0.747413275011633	0.947573236418764
Csnk1e	175.27989700761	-0.0842410986811884	0.261505643552098	-0.322138740628769	0.747347590495601	0.947573236418764
Dap3	678.830863034017	0.0392526397930133	0.121751369377969	0.322399986082752	0.747149694765637	0.947573236418764
Elovl7	231.326034769595	0.0817474225036654	0.25399690899623	0.321844162697582	0.747570755847884	0.947573236418764
Fermt1	993.678424076579	0.0637304215008612	0.197969953246288	0.321919667382941	0.747513553246529	0.947573236418764
RGD1310727	44.9444866762454	0.0994495106734134	0.308694664204601	0.322161417754532	0.747330411713021	0.947573236418764
Fbxo15	17.9537280102869	-0.146661440372352	0.455915097419439	-0.321685860377255	0.747690690725586	0.947619579704172
Gadd45gip1	1115.48724854832	0.0577332560167461	0.179846490074241	0.321014082581838	0.74819971889245	0.948070625695673
Scaf8	805.230582146858	-0.0343801276755629	0.107104526665729	-0.320996028327193	0.748213400711789	0.948070625695673
Smc6	724.532426156153	0.0471056566207271	0.146935972805423	0.320586277964116	0.748523937775732	0.948358385548876
Sirpa	289.969131946214	0.0712672783588709	0.222410141767901	0.320431783336764	0.748641035044736	0.948401025893721
Eif2a	937.712279709346	0.0467322856119902	0.145916080296348	0.320268235804302	0.748765000166326	0.948452356167443
Ccdc64b	121.158450246322	-0.0662763213590255	0.207351876082739	-0.31963212781629	0.749247216536548	0.948517665315912
Dynlrb1	2481.3039661154	0.0462468444611435	0.144557628114852	0.31991977915133	0.749029143634807	0.948517665315912
Manf	579.963503086218	-0.06233945636817	0.194915101728737	-0.319828765525453	0.749098140295124	0.948517665315912
Maz	305.05108897515	0.0571009524016265	0.178653804376013	0.319617892275308	0.749258009239979	0.948517665315912
Ogg1	93.9182110742597	-0.0900377394762511	0.281773226448304	-0.319539725655127	0.749317272292061	0.948517665315912
Slc39a13	630.927714909545	0.0447466151564981	0.140010127186286	0.319595561090819	0.749274939770412	0.948517665315912
Disp1	139.320323710437	0.0818893158964552	0.257165596899346	0.318430291157905	0.750158564107482	0.948613475883823
Eogt	115.497254404694	-0.0963510989171461	0.302638066408692	-0.318370719389379	0.750203746207258	0.948613475883823
Hsdl2	1830.51136625223	0.0649519180224563	0.203791057558364	0.318718195001538	0.749940216042436	0.948613475883823
Krr1	345.782770066053	-0.0593373336976298	0.186131517184997	-0.318792510774272	0.749883857750892	0.948613475883823
Mcm8	67.7226303550587	0.107986287966843	0.338758350427514	0.318770851937862	0.749900282861305	0.948613475883823
RGD1562218	1058.6492081964	0.0303448850011729	0.0952907740777404	0.318445151640986	0.750147293334608	0.948613475883823
Srms	18.8453043994886	-0.210470399643748	0.660360096299091	-0.318720650783268	0.749938353648455	0.948613475883823
Stom	1615.50813691547	-0.0831030193548352	0.260347556342442	-0.319200304862964	0.749574625798756	0.948613475883823
Trim21	61.7675825961798	0.114572453482559	0.359906707409344	0.318339311615687	0.750227567721246	0.948613475883823
Xab2	792.924879270301	0.035854793897601	0.112457493163611	0.318829745257054	0.749855621019329	0.948613475883823
Cln5	233.526074276401	-0.0575012273824702	0.180982858562095	-0.317716428170691	0.750700048555843	0.948697422708596
Dgka	1080.85768378774	-0.0444883343009803	0.140031823939286	-0.317701598461426	0.75071129859591	0.948697422708596
Dip2c	209.073757006425	0.0712228839787871	0.224085233861462	0.31783836333821	0.750607548717222	0.948697422708596
Hoxd10	353.898816041598	0.0878449833769342	0.276343623275711	0.31788315697551	0.750573569230167	0.948697422708596
Mtpap	533.765512798669	-0.0450543470067203	0.141796049686039	-0.317740494932534	0.750681791261839	0.948697422708596
Actl6a	578.982030697633	0.0447661188752855	0.14105759496685	0.317360570948384	0.750970021806575	0.948707932405639
Tmem184a	291.310105285869	-0.0466945183567127	0.147040468847441	-0.317562360367333	0.750816929289114	0.948707932405639
Whsc1l1	417.840707300323	0.0407732649416872	0.128471557229204	0.317371921233463	0.750961410372697	0.948707932405639
Ddx21	1040.95273851939	-0.0363472759448933	0.114604514126794	-0.317153963976323	0.751126779437687	0.948800508456185
Cdyl2	20.5171476860275	-0.136370359363961	0.430924874863684	-0.316459706363202	0.751653604443425	0.948940952128699
Defb42	20.5169799322745	0.25134117537082	0.794786592084786	0.316237311844344	0.751822389047944	0.948940952128699
Lsm14a	1244.4945266683	0.0354831343948594	0.112188930396413	0.316280173716622	0.751789858436918	0.948940952128699
Nnt	4775.12716632463	0.0419182763724985	0.132516242129578	0.316325574124789	0.7517554016499	0.948940952128699
RGD1566380	114.190449010161	0.453401688517644	1.43126395551628	0.316784117122612	0.751407416703643	0.948940952128699
Sec61b	1355.52373185474	0.0562065268430214	0.177694506413475	0.316309873487227	0.751767317643727	0.948940952128699
Smim12	1116.22103178157	0.0361383422033859	0.114098415654109	0.316729570662398	0.751448808968231	0.948940952128699
Aph1a	1573.43223304337	0.0360808456093214	0.114452468389669	0.315247422069389	0.752573802163604	0.949151557411398
Arv1	273.795402714469	-0.0526897485320431	0.167066329877679	-0.31538221118894	0.75247147159828	0.949151557411398
Bwk1	715.196388646759	-0.0358489268205792	0.113669665831106	-0.315378131522305	0.752474568776976	0.949151557411398
Hmgxb4	89.614136850777	-0.0783222594264089	0.248062484534839	-0.315736011325037	0.752202890735719	0.949151557411398
Iah1	11483.9732143026	0.0728348350445231	0.230652653359082	0.315777139277619	0.752171671153234	0.949151557411398
Mitd1	125.05644874262	-0.0696843662256441	0.22098125914455	-0.315340615287479	0.752503050330932	0.949151557411398
Ppp1r14a	98.0496575087669	0.0828822300712145	0.262702854170329	0.315498018980319	0.752383554943608	0.949151557411398
Wdr24	554.699995826227	0.0369541945448716	0.117275601980116	0.315105562631324	0.752681505141157	0.949182068948673
Cldnd1	718.020231328533	0.0455301901374096	0.144672721915972	0.314711643870598	0.752980602646279	0.949243292998847
Galnt16	13.2109477394944	-0.349572299493684	1.11013705487171	-0.314891119037623	0.752844324833342	0.949243292998847
Polr1e	169.702504603478	0.0574255695424519	0.182464619728073	0.314721668387183	0.752972990698328	0.949243292998847
Ap4b1	492.368543800008	-0.0402426503003196	0.128026283010217	-0.31433116196233	0.753269532926583	0.949291663293441
Dclre1a	373.670005316483	0.0434497662901594	0.13821884079661	0.314354874051475	0.753251525434656	0.949291663293441
Preb	1959.49164919144	0.0520357598533855	0.165500227271714	0.314415035623816	0.753205837990619	0.949291663293441
Ppp1r15b	1499.73329520333	-0.0423377439471834	0.134758271780441	-0.314175474260783	0.753387768989594	0.949335408241212
Zc3h10	216.911188255855	-0.0499334011302992	0.159037788618324	-0.313971928081411	0.753542359600953	0.949424948367635
Gemin4	172.532715293542	-0.0584449458800803	0.186212728321133	-0.313861175908926	0.753626478551151	0.949425687786786
Emc9	94.963325179011	-0.0796112936305457	0.253959517617558	-0.313480252196863	0.753915821629903	0.949491476788097
Heatr1	153.341871769982	0.0602565644071954	0.192272680547787	0.313391191278573	0.753983475751765	0.949491476788097
Sytl1	58.1416565914003	-0.118073821200992	0.376808269026219	-0.313352521445798	0.754012851441964	0.949491476788097
Tmem179b	350.558789047173	-0.0545660304030868	0.173978997003061	-0.313635733870375	0.75379771625798	0.949491476788097
Ptprm	365.838658129003	-0.0806989517491391	0.257807751993068	-0.313019880609753	0.754265558054708	0.949637211304125

Tmem151b	29.749303545453	0.168853611440619	0.539588837676548	0.312930141712526	0.754333737032935	0.949637211304125
Yrdc	515.414793436909	-0.0651369948033041	0.208191712487654	-0.312870258018396	0.754379234635311	0.949637211304125
Litaf	2557.53358049441	0.0449437734581068	0.143794986794473	0.312554522657631	0.75461913375992	0.949728831172101
Smg7	1071.66648046733	-0.0292286573120292	0.0934937081659685	-0.312626997959509	0.754564064151343	0.949728831172101
Clcn4	293.120384569366	-0.0752700117079766	0.241146824025773	-0.312133539440404	0.754939038163594	0.950026251967163
Itga5	124.951896926337	-0.123734610798661	0.396757334140492	-0.311864709613274	0.755143343471883	0.950072973422784
Uchl5	386.845237202667	0.0579839431573343	0.185888622151691	0.311928414370717	0.755094927577341	0.950072973422784
Dpp8	2612.41980908785	-0.0304524411214251	0.0977988992876796	-0.31137815806953	0.755513156490767	0.950117563252275
Noc2l	841.823229959203	-0.0343125185385168	0.110088223131302	-0.311682008870216	0.755282202170977	0.950117563252275
Orc4	272.287588382146	0.0699986788804873	0.224664004457293	0.311570511927706	0.755366947476518	0.950117563252275
Qsox2	343.667188116637	0.0410939138724019	0.131939066702027	0.311461304824968	0.755449955201116	0.950117563252275
Leo1	512.486958817573	-0.0595335440376072	0.191358691375529	-0.311109694624618	0.755717231376396	0.950269062044929
Arpc5l	3029.70804841136	0.0545657541477458	0.175605972354801	0.310728350613835	0.756007142731951	0.950269673235178
Dear	20.8202726397503	-0.135720291185285	0.436576995483312	-0.310873666247658	0.755896664536551	0.950269673235178
Otud6b	219.224701856677	0.0648969548030587	0.208722929825553	0.310923935656223	0.755858447693825	0.950269673235178
Prkrip1	349.874751525936	-0.0726553676340374	0.234033003184524	-0.310449238549283	0.756219355482332	0.950269673235178
Rock2	1665.25136886302	0.031002813075863	0.099811430895015	0.310613852520287	0.756094194985408	0.950269673235178
Thoc3	610.654978437796	-0.0376466238880742	0.121244099148636	-0.310502730874533	0.756178683105137	0.950269673235178
Asgr1	470.146342932097	0.0686276081327628	0.221891907671888	0.309283961063792	0.757105530496466	0.950379824107573
Cilp	29.7382330281904	0.124511534284361	0.401488832649967	0.310124526907863	0.756466261359499	0.950379824107573
Clstn1	2288.18402657623	0.0424978912778549	0.137083413073859	0.310014832027545	0.756549677326358	0.950379824107573
Cyp2d2	5624.42411756945	0.0378522765258406	0.12233073732438	0.309425720417829	0.756997707671768	0.950379824107573
Frmd8	705.968632264141	-0.0354702687656384	0.114713270768343	-0.309208067454277	0.757163257472311	0.950379824107573
Gsta4	6731.74821183671	0.0481721667031214	0.155758142025742	0.309275432260614	0.757112017694661	0.950379824107573
Mfap3l	322.508130115958	-0.0864329680784171	0.279281680682108	-0.309483127813168	0.756954044684907	0.950379824107573
Mrpl2	792.01829741417	0.0472401584974912	0.152656394392781	0.309454174424842	0.756976065991411	0.950379824107573
Slit3	62.3727231278321	-0.202174617391041	0.654023179764143	-0.309124544276779	0.757226789294227	0.950379824107573
Spg20	366.732218394541	-0.0402502203916028	0.13012793498486	-0.30931268060379	0.757083685898705	0.950379824107573
Trmt1	753.459853605446	-0.0477682572539543	0.154094099979258	-0.30999407024918	0.756565465655184	0.950379824107573
Cd200	308.793333356697	-0.195170598114679	0.631598909829584	-0.30901034672042	0.757313656206465	0.950383903769756
Cdip1	1760.32574940234	0.0406416946042505	0.13171243947042	0.308563828653236	0.757653339526052	0.950452677995833
Cep19	800.152413326297	-0.0403517045644286	0.130986936266701	-0.30805899973314	0.758037438503627	0.950452677995833
Chmp1a	1786.87698233501	0.0249938543212379	0.0810958416695041	0.308201429403707	0.757929064865358	0.950452677995833
Exosc4	652.502841901318	-0.0418788074746317	0.13578403230236	-0.308422181640454	0.757761105591463	0.950452677995833
Phc1	696.274879157554	-0.0368251070821155	0.119538902735395	-0.308059604358505	0.758036978438796	0.950452677995833
Slc36a4	106.141098693757	-0.0884334624389481	0.286876602585681	-0.308263070748462	0.757882163916175	0.950452677995833
Tdrd7	632.408224151345	0.0376740440293433	0.122260555612306	0.308145532634494	0.75797159571909	0.950452677995833
Zfp161	82.7848100079138	-0.100803342211695	0.32642233032921	-0.308812641923214	0.757464052006403	0.950452677995833
Pole	66.9331096176544	-0.104023765771744	0.33806364819558	-0.307704677290719	0.758307060342972	0.950685864533226
Rtcd1	971.132191043735	0.0435027991259648	0.141503815822791	0.307431986006967	0.758514584931546	0.950841157199951
Alg13	371.762231213419	-0.0426730218708899	0.138939728993238	-0.307133331697852	0.758741887964802	0.950916341229236
Rhoa	2417.10367523329	0.0406816066065282	0.132425746796177	0.307203150374853	0.758688747747168	0.950916341229236
Ccl28	68.6355176295643	0.0935874584476909	0.30495548580781	0.306888915934018	0.758927926058093	0.950939786966849
Slc35b2	810.045297186684	-0.0300294955961236	0.0978265600730638	-0.306966692621058	0.75886872448924	0.950939786966849
Klf16	350.744718199715	-0.072080549467059	0.235102023784042	-0.306592637132168	0.759153458632176	0.951012698204929
Nhs	44.5314729308159	-0.103183396117243	0.33654573682475	-0.30659546334106	0.759151307176028	0.951012698204929
Kynu	1216.27036658721	0.0422935503450556	0.138581275596858	0.305189500983454	0.760221828220846	0.951685708710385
Mad2l1bp	132.541714118716	-0.0684888449053482	0.224591361234622	-0.304948705635211	0.760405219583234	0.951685708710385
Mfge8	324.684366429302	0.0690011558375499	0.22638875885485	0.304790556680379	0.760525674222137	0.951685708710385
MGC93861	55.7485337933722	-0.124509162371858	0.407727715248452	-0.305373310950882	0.760081846378613	0.951685708710385
Mmaa	307.709609000192	0.0464961524571654	0.152552689181027	0.304787498055775	0.760528003890237	0.951685708710385
Nme6	259.294363774265	0.0823222595781409	0.269966480206195	0.304935114593725	0.760415571013752	0.951685708710385
Psmc2	2414.98736311577	0.0405855420796832	0.132998689674764	0.305157458159411	0.760246231474759	0.951685708710385
RGD1303271	165.075061256139	0.0553913794409934	0.181604714253267	0.305010691317981	0.760358009581358	0.951685708710385
Rgs12	69.2518258285032	-0.134426788761139	0.44057890221293	-0.305113994532973	0.760279332993211	0.951685708710385
Zdhhc20	206.341483762183	-0.0575348492894528	0.188667276642802	-0.30495404562595	0.760401152464198	0.951685708710385
Grk6	175.193739586966	-0.0534468664225586	0.17550985713455	-0.304523445549756	0.760729133421828	0.951772020025746
Nabp1	317.921883441558	-0.0770601932469404	0.253154206056087	-0.304400209056244	0.760823008539675	0.951772020025746
Ppp1r14b	949.446909631929	-0.0466501756995926	0.153269418723921	-0.304367147001593	0.760848194083576	0.951772020025746
Arl14ep	168.944694255055	0.0575689587875468	0.189947676306247	0.303077983932428	0.761830431835861	0.951870038431021
Foxo1	123.617255079593	0.0964670526369172	0.317869864910851	0.303479704387744	0.761524312174861	0.951870038431021
Fst	319.48888874588	-0.116475413188605	0.383128271239094	-0.304011533296424	0.761119104475505	0.951870038431021
Lamtor2	1466.85283743802	-0.0401042409663999	0.132352290264958	-0.303011310844071	0.761881241776466	0.951870038431021
Nap1l3	421.85606362406	-0.0871348725292832	0.287101670688639	-0.303498312358415	0.761510133403409	0.951870038431021
Phf7	260.530014503358	-0.0521937034670857	0.172211801064127	-0.303078552948008	0.761829998207333	0.951870038431021
Ptprg	303.867453650308	0.0532112822801921	0.175282708935257	0.303574052474545	0.761452422305343	0.951870038431021
Rnf121	215.44632230245	0.0607638183590283	0.200025798073865	0.303779907112729	0.761295575577683	0.951870038431021
Sass6	17.9245593371741	-0.199807789929063	0.657831084370924	-0.303737227802387	0.761328093398801	0.951870038431021
Slc16a4	6339.61345935602	-0.0568491197454196	0.187612611663354	-0.303013316862876	0.761879713023298	0.951870038431021
Suv39h2	31.0947719252448	-0.121444625572506	0.40073451259349	-0.303055069518558	0.761847894238109	0.951870038431021
Zfp260	272.645808416882	-0.0591406058755021	0.195218733014864	-0.302945342192129	0.761931515893492	0.951870038431021
Hes6	2017.20360965158	0.0521902829116429	0.172370501894593	0.302779665534408	0.762057781015844	0.951923149689645
Fbxo36	287.043381255419	0.1116329594038	0.369033865953769	0.302500582474415	0.762270489490716	0.951979604829301
Otub2	57.7693355242276	-0.1254813835885	0.414698977456554	-0.302584260897161	0.7622067104998	0.951979604829301
Sclt1	417.781283451366	0.0476140437219068	0.15761821710602	0.302084648564955	0.762587534771179	0.95227092069976
Evc2	275.20167504923	0.0585493016244827	0.194145707005037	0.301574021530972	0.762976814278204	0.952570522205873
Klhl26	216.36757329071	-0.0571657226168695	0.189572905051501	-0.301550069095262	0.762995076030465	0.952570522205873
Zfp787	518.336111701983	-0.0449905138782766	0.149269653119601	-0.30140429041011	0.763106223071435	0.952604649250954
Cldn9	36.2395702603136	0.127120904917139	0.422613799160714	0.300796862690223	0.763569400892581	0.95284618782465
Gucy1b2	1924.57203862679	-0.0637497747035692	0.2121084075424	-0.300552794876015	0.763755532144908	0.95284618782465
Hist1h1t	19.6732280051701	0.140656796796497	0.468432686105902	0.300271097573874	0.763970377410349	0.95284618782465
Igfbp6	26.973541347873	-0.187648544557315	0.624401860394047	-0.300525281008762	0.763776515655764	0.95284618782465
Phpt1	1295.1067545766	0.0417440147720102	0.138668646916372	0.301034269103275	0.763388362874866	0.95284618782465
Plcd1	175.831870388588	-0.0751231556647192	0.250114046743191	-0.300355604344978	0.763905923767154	0.95284618782465
Pptc7	834.433239377145	-0.0300727679787825	0.0999774277369929	-0.300795576156389	0.763570381994547	0.95284618782465
Stard8	892.646157621148	-0.0558867558042957	0.186009267626415	-0.300451458776451	0.763832817199466	0.95284618782465
Dnmt3a	104.655072714967	-0.066812802281771	0.222638262960717	-0.300095775960844	0.764104101102781	0.95287048605393
Kif3b	721.639297098121	0.0367292842771086	0.122420448010824	0.300025729965153	0.764157529507441	0.95287048605393
Aoc2-ps1	33.3001697040046	-0.107816163816641	0.35970408020618	-0.299735726530658	0.764378744973164	0.952923414742994
Lrrc20	135.017667100445	-0.0669077229121798	0.223293427455879	-0.299640359658146	0.764451495304119	0.952923414742994
Ptpdc1	144.817972870172	-0.0729289916539801	0.243306694642961	-0.299740998746456	0.764374723139903	0.952923414742994
Eapp	320.639193261311	0.05201962762664	0.174152466592534	0.298701641409138	0.765167704537759	0.952980071200544
Jmjd8	1622.2531388159	-0.0328842322822298	0.109909760066697	-0.299193013088869	0.764792780022682	0.952980071200544
Llgl2	1649.41244247948	-0.0334047618833801	0.111795881934451	-0.298801362853118	0.765091611014278	0.952980071200544
LOC497934	439.478788135057	-0.0396779201768988	0.13262785735118	-0.299167316500011	0.764812385569274	0.952980071200544
Mvp	2036.6182224499	0.045051946264333	0.150726528578603	0.298898585996682	0.765017426028698	0.952980071200544
Stard6	38.1432802265393	-0.119209067990178	0.398337441309428	-0.299266540444478	0.764736682203409	0.952980071200544
Supt6h	1711.0686619504	-0.0323886433915672	0.108303579184623	-0.299054229189922	0.764898668793977	0.952980071200544
Usf1	671.988139829937	-0.0321717815814004	0.107658009903497	-0.298833144047885	0.76506736050721	0.952980071200544
Nucb1	3399.09587857691	0.0450556840191256	0.150912765821364	0.298554491224807	0.765279993213003	0.953015492808042
Atp2b4	100.821205260103	0.128715528372678	0.43199250751707	0.297957779667261	0.765735387731155	0.953160267783486
Cry1	166.081152007948	0.0751879432359238	0.252169068176469	0.298164813708662	0.765577375631783	0.953160267783486
Kbtbd8	49.9467841226017	-0.117951993742093	0.395489392071159	-0.298243128910194	0.765517606605494	0.953160267783486
Man1a1	228.621464656839	-0.201285072419868	0.675787188594888	-0.297852749825554	0.765815552120253	0.953160267783486
Utp3	670.08187428202	0.0439368246868712	0.147498651630742	0.297879500599542	0.765795134261641	0.953160267783486

Stk38	906.662760900625	-0.0380221478492656	0.127771321296376	-0.297579671740812	0.766023991645508	0.95321093595827
Strap	2452.63250662945	0.0294633451172	0.0989830579712419	0.297660485754645	0.7659623048325	0.95321093595827
Mfsd11	862.431029766111	-0.0395550468869372	0.133015586278358	-0.297371518584006	0.766182885583088	0.953304288728777
Ndufaf7	326.796395551046	0.0533490310035122	0.179537947253397	0.297146268071208	0.766354841886337	0.953413871812621
Rai2	86.177473412987	0.114847953259691	0.386900788530706	0.296840835336206	0.766588027703264	0.953599597556671
Zdhhc6	866.095627071318	0.038788172644276	0.130738986967964	0.296684053807	0.766707732415006	0.953644132920656
Jtb	1087.24151832466	-0.0302595732749557	0.102098282700752	-0.296376907373123	0.766942258800109	0.953765884014219
Sumf1	1101.09287256581	0.0343759641423542	0.11600330863896	0.296336066149141	0.766973445353683	0.953765884014219
Brca1	27.9336805529599	0.128171600958186	0.433440584766206	0.295707429029335	0.76745352324724	0.953945400834223
Pfdn5	2529.99845626012	-0.0466479033147535	0.157601729472461	-0.295985986136685	0.767240783601227	0.953945400834223
Snrpa1	443.797539386828	0.0579437284934083	0.195815447809904	0.295909894451533	0.767298894605734	0.953945400834223
Ywhah	2318.35914422185	-0.033265687296683	0.112463272713553	-0.295791563717088	0.767389266052035	0.953945400834223
Tsr2	896.555651083602	0.0458091020001142	0.155012240411147	0.295519256276873	0.767597244468372	0.954019713573265
Adat3	95.1126637575243	-0.0762608677858249	0.258389676305924	-0.295138988817551	0.767887706783006	0.95406773888234
Rhob	2084.86648184061	-0.0377106900237664	0.127744494256857	-0.295204033983189	0.767838020573435	0.95406773888234
Tsc22d2	288.015908417527	0.0645135214102308	0.21849046116156	0.295269281172541	0.76778818100201	0.95406773888234
Camlg	260.523774479162	-0.0451385194260302	0.153148648607856	-0.294736648585188	0.768195064589557	0.954285866964719
Ptpre	47.2661105387798	-0.139205620155969	0.472380874988449	-0.294689365142847	0.768231187992889	0.954285866964719
Mrps31	962.286650368897	0.0522982630562663	0.177667223537421	0.294360783125825	0.768482230584308	0.954493392287318
Ndufb6	2501.49996641605	0.0499326097109465	0.169792425564093	0.294080313329984	0.768696533780795	0.954550945367914
Six4	81.3425128332674	-0.109011297657407	0.370675673594152	-0.29408808136884	0.768690598088765	0.954550945367914
Slc35b4	2346.21243265071	0.0604057428358568	0.205568775288956	0.293846878014173	0.768874912043365	0.954668150566406
Arf6	728.038172418215	0.0304191869479175	0.103674638404037	0.293410108934925	0.769208699497872	0.954682537979603
Bola1	517.26605134511	-0.0415649342365121	0.141739492109101	-0.29324878774448	0.769331995121162	0.954682537979603
Cabyr	62.922532658461	-0.0876223307171953	0.302456468091964	-0.289702287638144	0.772044006063667	0.954682537979603
Cdc7	17.0272849355983	0.156581757582267	0.534565016345812	0.292914337441367	0.769587629582786	0.954682537979603
Cldn12	605.656325531937	-0.0510882881664906	0.17499928465649	-0.291934268570142	0.77033688165373	0.954682537979603
Cml2	87.6826726703584	0.152426337695659	0.528223260801762	0.288564228436854	0.77291487301652	0.954682537979603
Cox7c	8380.90827707982	-0.0590498889719873	0.203491000467444	-0.290184277615926	0.77167526362366	0.954682537979603
Dhx9	1497.73422939633	0.0401560300830769	0.138100957729779	0.290773002180403	0.771224934745662	0.954682537979603
Eif3f	1952.79595696677	0.0396332637194926	0.137589537993016	0.288054341177483	0.773305142372384	0.954682537979603
Etf1	1802.09453177158	0.0446448559521427	0.153086360057062	0.291631834054331	0.770568132895624	0.954682537979603
Fam101b	123.389739575356	0.0914497635598956	0.316966569946849	0.28851548469364	0.772952179154407	0.954682537979603
Fam32a	1709.73978313199	0.0273389406972046	0.0948148009942098	0.28834043219554	0.773086160318106	0.954682537979603
Fat3	123.295896856259	-0.0811774783087079	0.281925631798866	-0.287939332762132	0.773393178111686	0.954682537979603
Fopnl	385.09049352882	-0.0457039547661247	0.157471677296053	-0.290236031970368	0.771635672448153	0.954682537979603
Foxp1	96.3702046360982	0.0737852000100292	0.253902248360159	0.290604752366609	0.771353625013967	0.954682537979603
Glmn	205.248514207354	-0.0557594928238435	0.191741974183485	-0.290804833220739	0.771200588650472	0.954682537979603
Hyal2	767.997723583105	-0.0432595815124545	0.15024225201741	-0.287932195714436	0.773398641419035	0.954682537979603
Igsf3	207.804007131461	-0.0653462072484178	0.225638222473628	-0.289606107210206	0.772117594268698	0.954682537979603
Kat5	823.161375467447	0.0281480543549625	0.0968318282189946	0.290690105440362	0.771288339697703	0.954682537979603
Lpcat4	586.939307028456	0.0450579091769219	0.155864326560759	0.289084168078431	0.772516968662755	0.954682537979603
Lrfn3	38.0495971456605	0.116254572100546	0.402334838411961	0.288949802506315	0.772619791499245	0.954682537979603
Lrrc66	38.0663075799273	-0.198710257993969	0.688856237197022	-0.288464047015858	0.772991547670363	0.954682537979603
Mapkapk5	542.084599119376	0.0453251185218531	0.155933753671347	0.290669065899499	0.771304432384009	0.954682537979603
Mettl3	716.86663102358	-0.0439501211625938	0.151459536211868	-0.290177312448089	0.771680591900571	0.954682537979603
Mgat2	562.987841175108	-0.0388249613801021	0.13390417095523	-0.289945870267798	0.7718576487498	0.954682537979603
Mme	8722.11473752901	0.0574181154558814	0.197574419076513	0.290615129854668	0.771345687338829	0.954682537979603
Mog	12.890680706341	-0.168347624753052	0.584859455300721	-0.28784287101333	0.773467019142338	0.954682537979603
Naa10	302.223098087618	-0.0496477057948548	0.170868535675126	-0.290560843157516	0.77138721115587	0.954682537979603
Ndufb2	2960.18831008892	0.0526595260062407	0.182422433788234	0.28866803776651	0.772835424052097	0.954682537979603
Nelfb	1173.97667616086	0.0262064927493564	0.0908033995908721	0.288606955988801	0.772882171816271	0.954682537979603
Nosip	746.185314809738	-0.045353133603508	0.156035482558079	-0.290659104326651	0.771312051808325	0.954682537979603
Pard6a	17.5498421292168	0.13967475526904	0.482525761292714	0.289465903115398	0.772224868909076	0.954682537979603
Pdzd7	38.5119307641851	-0.125818705669837	0.435177582728046	-0.289120374448296	0.772489262533758	0.954682537979603
Plac8	1018.35008818714	0.0529720727012468	0.181618738703403	0.291666339494595	0.770541747885441	0.954682537979603
Prpf4	521.260352619891	-0.0356900539643523	0.122714545277114	-0.290838008516081	0.771175214636443	0.954682537979603
Rbbp7	2637.79660230569	0.0260932537944908	0.0904154388308729	0.288592901078539	0.772892928588248	0.954682537979603
Rbm34	330.163467307428	-0.0459605303947286	0.157083283449045	-0.292587023810446	0.769837833433879	0.954682537979603
Rcc1	107.406078389676	-0.0688313762365554	0.236038261329756	-0.291611096645026	0.770583990138977	0.954682537979603
RGD1311703	1833.60095883427	0.0357063327141748	0.123896110362986	0.288195752147213	0.773196900210664	0.954682537979603
Rps18	10615.4157190617	-0.0579415388032618	0.199361485638653	-0.290635568939741	0.771330053681625	0.954682537979603
Rsph9	269.89176771796	-0.0597394225220921	0.206949116825943	-0.288667202055937	0.772836063641555	0.954682537979603
Rwdd2a	194.804332199894	-0.057842134386245	0.198959159755768	-0.290723656338563	0.771262677550438	0.954682537979603
Sdccag3	2622.17083218644	0.029282741761608	0.101116161639923	0.289595068549818	0.772126040143126	0.954682537979603
Sdf2	1757.98639860451	0.0538363821489525	0.184922355926947	0.291129657520805	0.7709521584071	0.954682537979603
Sdsl	672.840864125803	0.0689707826908333	0.239118866806332	0.288437226271628	0.773012075518068	0.954682537979603
Setmar	66.5652306666341	0.0885489967222746	0.30276286199438	0.292469809999082	0.769927439401806	0.954682537979603
Sfrp5	16.1129699504771	-0.140625681187035	0.486852276008558	-0.288846716174257	0.772698680700898	0.954682537979603
Snx2	2345.039100276	-0.0335224767382374	0.114239410395484	-0.293440561555651	0.769185425622785	0.954682537979603
Tcn2	699.028545689712	-0.0645450714919124	0.223511733025636	-0.288777106320898	0.772751952581083	0.954682537979603
Tjp3	416.394612417587	0.040742387505507	0.140286730123037	0.290422247847493	0.771493225480774	0.954682537979603
Tmem117	333.832202523826	0.0517069941158614	0.176400887607975	0.293122074480558	0.76942884446044	0.954682537979603
Txnl1	1895.67530852123	0.0489746156110525	0.170173837635453	0.287791685793477	0.773506202028345	0.954682537979603
Upk1b	113.969919189567	0.154673271204075	0.535476797794897	0.288851490561352	0.772695026938781	0.954682537979603
Xpo7	1514.07150901885	0.0311830663943664	0.108222269591225	0.288139091077562	0.773240270550279	0.954682537979603
Zbed5	1814.14883133949	0.0632776856146701	0.218243304355988	0.289941016982837	0.771861361715117	0.954682537979603
Tfe3	560.846210256095	0.0392891341697586	0.136736563368522	0.287334515376618	0.773856196932826	0.955010807202877
Nme3	611.148439868595	0.0617143105187284	0.214874019158274	0.287211598500748	0.773950305968416	0.955023252376183
Fam58b	258.606029429451	-0.0437601148183642	0.152440905822868	-0.287062810222424	0.77406422741024	0.955060139898479
Fastkd3	314.005443384115	-0.0435920413722854	0.151977879164675	-0.286831488976441	0.774241350822081	0.955077922965252
Rcan3	25.1956738546534	-0.154769257270486	0.539595658315262	-0.286824504395959	0.774246699120344	0.955077922965252
Fzd4	3814.39064392657	0.0371441658367992	0.129658650497937	0.286476572863837	0.774513134061542	0.955199249321125
Mypop	25.0086059918479	0.1310220608121	0.457346136335624	0.286483366541331	0.774507931424294	0.955199249321125
Abhd5	105.324744841163	0.0755601364588139	0.264900203423681	0.285240009189284	0.775460270056549	0.955330742408708
Ciao1	853.694526783249	0.0351825430503523	0.123286943934745	0.285371199313483	0.775359770169502	0.955330742408708
Ddx51	249.31017286434	-0.0417547744694768	0.146152994291361	-0.285692227326094	0.775113858390473	0.955330742408708
Ggps1	526.185756709752	-0.0410521626652093	0.143645382299033	-0.285788251652595	0.775040306844572	0.955330742408708
Ndufb5	3240.57852744023	0.0365895108447212	0.128249946250258	0.285298449742215	0.775415500452463	0.955330742408708
Ptpn1	240.91003938248	0.0503337120409419	0.176160793819813	0.285725960638132	0.775088019528387	0.955330742408708
Rcor1	545.156432238872	-0.0456005741661759	0.159785445104333	-0.285386282438934	0.775348215787795	0.955330742408708
Supt4h1	840.838338510805	-0.0451841390279318	0.158224124258633	-0.285570479467935	0.775207116235266	0.955330742408708
Tnpo2	1169.55641341186	0.0297096495733749	0.104092710631562	0.285415274452143	0.775326006685222	0.955330742408708
Vav2	853.311759839485	-0.0270561144338985	0.0946940263309801	-0.285721449200295	0.775091475161007	0.955330742408708
Eno4	29.4354866716104	-0.104954111448241	0.368378527189963	-0.284908331245158	0.775714372994435	0.955333141559729
Sdc1	319.902603153182	-0.110734580653165	0.388407504424117	-0.285098973093605	0.775568316771841	0.955333141559729
Wdr43	619.072847296491	0.0542734956439511	0.190432238244634	0.285001616030108	0.775642903836688	0.955333141559729
Mtmr1	769.42143033428	-0.0353369750494454	0.124298454682939	-0.284291346498098	0.776187117176602	0.955811784813571
Sh3bgrl3	710.446648548107	0.0317775426035537	0.111897803976846	0.283987187184918	0.776420199731337	0.955995232385048
Acot2	1659.61826771026	0.0667516591885929	0.235971325672354	0.282880383870358	0.77726853208683	0.95599771882541
Acvr1	174.239503229648	0.090181180619226	0.319141680674125	0.282574123281972	0.777503318830733	0.95599771882541
Arsg	39.6437636804497	0.140825177930286	0.498393818247924	0.282558034979946	0.777515653072505	0.95599771882541
Fuk	397.656388256986	0.036225057425323	0.127755248713681	0.283550443446036	0.77675491923647	0.95599771882541
Ggnbp2	1705.64131936242	-0.0288478418211198	0.101789396458375	-0.283407140869694	0.776864755072035	0.95599771882541
Kb15	49.0548958343281	0.112351447593961	0.397529199069637	0.282624390502393	0.777464781374387	0.95599771882541

Me2	290.224225097539	-0.0554455943162208	0.195977669817531	-0.282917917984455	0.777239758929621	0.95599771882541
Pelp1	551.25657746134	-0.0371998781148303	0.131384458645234	-0.283137583382507	0.777071372458248	0.95599771882541
S100pbp	117.401416680731	-0.0650181486996098	0.229336446087436	-0.283505521293468	0.77678934983419	0.95599771882541
Sec14l3	25.7879074192586	0.110017588728807	0.389133840035759	0.282724290230572	0.77738819469063	0.95599771882541
Smyd5	195.378982953956	-0.0582534624177214	0.205744097860171	-0.283135521376229	0.777072953058884	0.95599771882541
Stbd1	120.869973125645	-0.0730881906934478	0.258339609537545	-0.282915155071587	0.777241876931796	0.95599771882541
Usp39	576.859669984242	-0.0708119692860747	0.24949201421017	-0.283824592583646	0.77654480705266	0.95599771882541
Ylpm1	776.286449244035	-0.0311900131010395	0.110477686731394	-0.282319570800501	0.777698480358448	0.956119083586168
Arap2	193.294579876697	-0.0547271911325887	0.193965327565215	-0.282149350193469	0.777828993742998	0.956176113225494
Ppp1r12b	318.309554847859	-0.0552341866498466	0.195938572890457	-0.281895421789798	0.778023700123689	0.956208626254958
Timm21	1014.92810200563	0.0378933734948978	0.134390400073071	0.281964883461128	0.777970437147965	0.956208626254958
Klc1	237.651825249712	-0.0531373313941867	0.188654727201447	-0.281664457511559	0.778200810252723	0.956322889969992
Apoh	613.700473862022	0.0871288107514462	0.309795849875349	0.281245893986326	0.778521806298223	0.9564602967681
Parvb	150.499265974969	-0.0894005643621066	0.317758923445795	-0.281347140129511	0.778444157242024	0.9564602967681
Slc30a5	1028.14015817922	-0.0304526770407716	0.108299491174073	-0.281189474767005	0.778565077045439	0.9564602967681
Adck1	175.079287113416	-0.0601620291356682	0.214696377474993	-0.28021911614543	0.779309401382392	0.956667621772162
Blcap	1381.87612567602	-0.0434918330314257	0.155439695660922	-0.279798753120948	0.779631908375735	0.956667621772162
Emg1	526.641869347912	0.0453424446697833	0.161677305120737	0.280450274922151	0.779132070089552	0.956667621772162
Ext2	617.797250453843	-0.0399873920198056	0.142755937564805	-0.280110184570453	0.779392971203538	0.956667621772162
Gtdc2	80.1954504275166	-0.0779447786887657	0.278480257950634	-0.279893372917598	0.779559311748887	0.956667621772162
Il12rb1	35.6870830412992	-0.169437791862114	0.604971181666263	-0.280075806909404	0.779419345490835	0.956667621772162
Nudt7	441.591388847326	0.0327960085190104	0.116822061244751	0.280734718849895	0.778913877368625	0.956667621772162
Pin1	1613.90625243717	0.0387234584160175	0.13811576294977	0.280369579756804	0.779193973325042	0.956667621772162
Plekhm2	570.319786626501	0.0496854127914677	0.177486093684652	0.279939750546012	0.779523729417574	0.956667621772162
RGD1563325	49.8209136492574	0.096497555462606	0.344926664225426	0.279762527722531	0.779659702663693	0.956667621772162
Srpx	62.5225737122411	-0.088469975039862	0.315771606911286	-0.280170772493541	0.779346489215311	0.956667621772162
Mfsd5	365.26097730221	0.0425464040445983	0.15226651733434	0.279420615834911	0.779922051744042	0.9567829509037
Msi1	30.7985428160205	-0.117484594358455	0.420322521320843	-0.279510586273762	0.779853014971701	0.9567829509037
G6pc3	924.431799029774	0.0369705891295318	0.132551431569004	0.278915049742677	0.780310018662498	0.956932477532321
Gstcd	135.216044566207	-0.0661610990189755	0.23728714351697	-0.278822940165926	0.780380708626349	0.956932477532321
Msh3	224.00969208666	0.0484398877998903	0.173692124925585	0.278883615596525	0.780334142753976	0.956932477532321
Ptpn11	836.820635550841	0.0287919407224463	0.103237416462436	0.278890558375437	0.780328814509818	0.956932477532321
Flrt1	124.722461705712	-0.066854951562679	0.240139528203964	-0.278400445202405	0.780704977791943	0.957020359963678
Kcnj1	3890.10983247967	-0.0566732149038744	0.203426293421079	-0.278593361510868	0.780556907835125	0.957020359963678
Rasa1	259.213847134944	0.0696510318593925	0.250125100157079	0.278464783484951	0.78065559504481	0.957020359963678
RGD1306058	432.194384865312	-0.0538366102368179	0.193579988987913	-0.27811041067979	0.780927604310321	0.957190030258936
Atxn7l3b	1749.99621236192	-0.0347674726611523	0.12517031039712	-0.277761336141516	0.781195572946351	0.957312008852639
Calr	14531.6400285215	-0.0398673303783192	0.143474226081567	-0.277871025808175	0.781111366380438	0.957312008852639
Grina	11957.80203317	0.0358163648322667	0.129068670450455	0.277498518480637	0.78139734325076	0.957348662994763
Oxnad1	1291.99465802695	0.0388797758553943	0.140216676461698	0.277283535999477	0.781562400486399	0.957348662994763
Sh3bgrl2	42.0767701971695	0.101490251704221	0.365901292518062	0.277370574467734	0.781495573720721	0.957348662994763
Slc15a4	380.882646737098	-0.0421863863877879	0.152003143159511	-0.277536276625003	0.781368354672724	0.957348662994763
Veph1	229.278532878232	-0.0626494120941594	0.22617835445465	-0.276991192394234	0.781786869107603	0.957520426107437
Pop7	432.235805894935	-0.0490658410284655	0.17730054297288	-0.276738244597315	0.781981103325553	0.957655125568175
Brms1	753.921203959191	-0.0338426418337817	0.122791740047347	-0.27561008436506	0.782847563360454	0.957705035425873
Bspry	1775.4446042602	0.0318312126328547	0.115541822227383	0.275495158542781	0.782935844891106	0.957705035425873
Fam122b	30.1600301918788	0.112523310585358	0.407612612122206	0.276054536191884	0.782506179142781	0.957705035425873
Gtpbp6	323.519382633633	0.0463228079449174	0.168093202571386	0.275578115214058	0.782872120533681	0.957705035425873
Hs3st3b1	14.1416471381912	-0.226643091107247	0.823194669801767	-0.275321378309975	0.783069341391084	0.957705035425873
LOC689226	1692.53374697835	0.0231104018786458	0.0839394027748623	0.275322448274159	0.783068519434132	0.957705035425873
Parp2	318.89613434746	0.0544351053959316	0.197675525353499	0.275376050214545	0.783027342209168	0.957705035425873
Prorsd1	134.693499848064	0.058715499409917	0.213309990461594	0.275259022246727	0.783117244347006	0.957705035425873
Rab21	864.979507221102	-0.0339237348537042	0.122781510373723	-0.276293513171868	0.782322637821096	0.957705035425873
Rbms1	255.926164497784	0.0432144310571553	0.156775892106484	0.275644619057907	0.782821035703767	0.957705035425873
RGD1309676	1304.62194158885	-0.0415612527878655	0.150636298670871	-0.275904633574898	0.782621314924679	0.957705035425873
Rpusd2	54.1541984147233	-0.0880871823288035	0.319647787344204	-0.275575761248581	0.78287392874596	0.957705035425873
Zfp414	290.057748402957	-0.0496091899621518	0.18007012987038	-0.275499273521166	0.782932683877079	0.957705035425873
Jag1	884.448029180386	0.118823826165068	0.43190266361187	0.275117141374821	0.783226242635657	0.957735284970078
Sacm1l	2762.93944218234	0.0439189431030085	0.159725488792338	0.274965150741271	0.783343012348394	0.957775029943185
Ankrd16	88.5614187657546	0.0679454583877865	0.247714947273788	0.27428889187171	0.783862620293348	0.95808573424261
Cdk2ap1	1517.61165281935	-0.045180348226828	0.164818406777384	-0.274121981338238	0.783990881938034	0.95808573424261
Ctgf	8104.19347922804	0.146725173504199	0.534996297647388	0.274254558675292	0.783889003003296	0.95808573424261
Fam63a	658.612078428737	-0.0463007551947946	0.168719679964213	-0.274424152562495	0.783758684134312	0.95808573424261
March3	118.552604959456	-0.0737747522910401	0.269499308843239	-0.27374746379759	0.784278699601079	0.95808573424261
Pfdn2	1179.43637126524	-0.0588819730045231	0.215174788320109	-0.273647175230056	0.784355776625681	0.95808573424261
Rgs2	86.7039557942875	-0.115314244407262	0.421231061378043	-0.273755320963311	0.784272661046464	0.95808573424261
Sts	949.69590645826	-0.0336512654247031	0.12289871194509	-0.273813003343258	0.784228330165703	0.95808573424261
Trim23	408.463565171409	-0.0514086416706027	0.187625831517335	-0.27399554344335	0.784088046473383	0.95808573424261
Slc36a1	74.4589982486828	0.106697860584079	0.390276475950231	0.273390447949226	0.784553094643745	0.958120820212829
Snrnp35	605.153511074886	-0.0302052542424316	0.110471071431783	-0.273422298262795	0.784528614058803	0.958120820212829
Clca4	169.071546989289	-0.135541944899336	0.496028834632839	-0.273254164749645	0.784657846174863	0.958145797123281
Myadm	514.626481871442	-0.0770728788404541	0.28272741674817	-0.272604898834782	0.785156945801982	0.958652255450138
Mef2d	282.228642775304	0.0514798507646053	0.188984327225199	0.272402751701523	0.78531235712409	0.958739017301011
Zfand4	80.2880744776903	-0.0729111203619323	0.268609525621716	-0.271439071988137	0.786053354520928	0.95954058935505
Snx9	1123.19833554967	-0.040756158392614	0.150229429485032	-0.271292772210619	0.786165865037959	0.959574873498866
Ankrd37	152.009084885879	-0.0850434369932732	0.313929756330362	-0.27089957316368	0.78646827332204	0.959601447287471
Cacfd1	178.17233520469	0.0632083696226939	0.233391611409014	0.270825370462534	0.786525346025873	0.959601447287471
Mon2	1237.41666253476	-0.0281971545007842	0.104064607708631	-0.270958158798168	0.786423213231546	0.959601447287471
Rdh13	236.792215875943	0.0463114938781656	0.170807730541713	0.271132306080584	0.786289275195021	0.959601447287471
Eif2s1	1126.62436518752	-0.0389183857171902	0.143860914330048	-0.270527862960074	0.786754184218534	0.959708885030112
Ulk3	163.51850958574	0.0543226415752984	0.200829504966063	0.270491338334365	0.78678227966836	0.959708885030112
Aff1	238.893968919483	0.0769169254323872	0.288038859442968	0.267036626867414	0.789440960545151	0.959758900155758
Akap6	10.8797954189209	-0.250795569711169	0.939106975879679	-0.267057509051345	0.789424882608608	0.959758900155758
Anapc10	64.6820687689518	0.0729426962164354	0.272179134276722	0.267995180491225	0.788703028501253	0.959758900155758
Ankrd24	144.321928395938	-0.069085855797847	0.256115562011987	-0.269744857575713	0.787356547890791	0.959758900155758
Aprt	1193.50499806432	-0.0330480768028858	0.12285958156351	-0.268990634530219	0.787936889710711	0.959758900155758
Arfip1	356.688938904458	0.0501115423969411	0.187656686539682	0.267038405723658	0.789439590936931	0.959758900155758
Atp6v1g1	4809.72770437468	-0.0365608865900977	0.135381699551653	-0.270057819566288	0.787115771894668	0.959758900155758
Cited2	1847.05573115532	-0.0464874686749212	0.17290649474001	-0.268859008129346	0.788038182576299	0.959758900155758
Csnk2a2	665.202716313289	-0.0323495451542122	0.120069898969246	-0.269422606597664	0.787604491571366	0.959758900155758
Cth	14286.4383115387	0.0456474362547549	0.169665391985924	0.269043885264132	0.787895911720801	0.959758900155758
Cyp2s1	305.93064174877	0.0828216946814702	0.307883932474138	0.269002977894689	0.787927391080165	0.959758900155758
Eri2	981.864187371501	0.159104802853954	0.590236375536184	0.269561161338828	0.787497883289534	0.959758900155758
Etv3	51.237027241935	0.109443309220412	0.407712809892061	0.268432353767314	0.788366538324306	0.959758900155758
Gps2	787.58427601835	0.0342573546233712	0.127989896259814	0.267656710603392	0.788963574234457	0.959758900155758
Hnrnpl	2908.34763926869	-0.033802769364326	0.126461575392923	-0.267296759978665	0.789240681205555	0.959758900155758
Hus1	1124.08729835615	0.0364326418921279	0.136238606841323	0.26741789817743	0.789147420327311	0.959758900155758
Kctd3	897.3103956398	0.0367514259788901	0.13661191168742	0.269020655116668	0.787913787923335	0.959758900155758
Mina	180.40205619276	0.0533186685621643	0.199648019541968	0.267063348208952	0.78942038684941	0.959758900155758
Mmp17	40.9181953180203	-0.141506821014857	0.527598944287845	-0.268209067790807	0.788538395541463	0.959758900155758
Phyh	10142.8737051352	0.0386374084035577	0.142983497637493	0.270222851181858	0.786988813705772	0.959758900155758
Polr1b	203.874169905753	0.0542582412159397	0.202129142614549	0.268433539637616	0.788365625619286	0.959758900155758
Ppp6c	825.605830997061	0.033399199177326	0.12360396684557	0.270211385845365	0.786997633762195	0.959758900155758
Prex2	33.4032489049876	0.168135808941535	0.628432165082216	0.267548063711122	0.789047212926061	0.959758900155758
Rnf7	1661.695448241	0.0437184614499036	0.162131949220523	0.269647417798204	0.787431516925988	0.959758900155758

Sh3bp4	684.418672589915	-0.0411045694649424	0.153545118619812	-0.267703524764732	0.788927536445741	0.959758900155758
Spryd7	384.968439026551	0.0392330324879246	0.146152361881981	0.268439264222124	0.788361219697184	0.959758900155758
Srxn1	647.431205827301	0.0782179829916505	0.291741124174483	0.268107498430253	0.788616574155696	0.959758900155758
Sult1b1	3245.11439902253	-0.069443543725597	0.258599175841453	-0.26853737448944	0.788285710257226	0.959758900155758
Ucma	31.4985197083427	-0.119679847650307	0.44722351231929	-0.267606340797358	0.789002349681548	0.959758900155758
Ywhaq	4536.17069867072	-0.0217948623025697	0.0813518742417436	-0.267908545509407	0.788769715714407	0.959758900155758
Zfp259	618.075108782114	-0.0326554456504285	0.12202040845676	-0.267622818702501	0.78898966468061	0.959758900155758
Ptgfrn	863.438481460366	-0.0522904467429779	0.196010610612184	-0.26677355159327	0.789643519238054	0.959902485525104
Atp6v0a1	1068.84009138321	0.0306088777484187	0.115019924232238	0.26611804826628	0.790148295521107	0.959965109243078
Entpd4	2056.26039770679	-0.0211025614090125	0.0792616768227647	-0.266239149295308	0.790055033937534	0.959965109243078
Hibch	655.824677009473	-0.0355552977390479	0.133406248079769	-0.266518984311648	0.789839540610751	0.959965109243078
Nap1l1	1766.43902810785	0.0322419635116635	0.121213891781564	0.265992313568859	0.790245128737326	0.959965109243078
Phf21b	38.3173631441603	0.0993514204513214	0.373587366488553	0.265938919148021	0.790286250852321	0.959965109243078
Slc35a2	415.006075258581	-0.0305529360475953	0.114854110768365	-0.266015172144895	0.790227524211512	0.959965109243078
Tctn1	673.941299486066	-0.0407413734556335	0.152954824098555	-0.266362134674368	0.789960324267272	0.959965109243078
Actr10	1264.6449836634	0.029881847627625	0.113683239278421	0.262851831257566	0.792664784152008	0.960093946570779
Acvrl1	482.944764130993	-0.0685280209383522	0.26291174513941	-0.260650283622799	0.794362208876594	0.960093946570779
Adprh	331.500932489182	-0.0341870767933728	0.130370122438899	-0.262230917282412	0.793143418620269	0.960093946570779
Alg5	603.102085144962	-0.036360049101553	0.139014778825454	-0.261555277854353	0.793664327002089	0.960093946570779
Atp2a3	238.515231317305	-0.115016017771694	0.433216355223902	-0.265493249238593	0.790629509653682	0.960093946570779
Atp5h	5953.63490649408	-0.0384393963523094	0.146687403674274	-0.262049742441866	0.793283092797494	0.960093946570779
Bles03	372.426868766561	0.0323419003858818	0.124238336757804	0.260321421148213	0.794615850377557	0.960093946570779
Cap2	133.92283304362	0.0614329054538485	0.232798683174679	0.263888543595209	0.79186580372468	0.960093946570779
Chchd2	3193.78483600463	0.0558996536405377	0.213919149227562	0.261312060385361	0.793851866755406	0.960093946570779
Coasy	1941.780631664	-0.035267718353821	0.132880326692549	-0.265409630090852	0.790693918368518	0.960093946570779
Dph5	188.864724839315	0.0552623684406495	0.212210263728431	0.260413268753907	0.794545008960693	0.960093946570779
Eif1ax	1920.78670047858	0.0251899812967861	0.0967306965143415	0.260413521296741	0.794544814178504	0.960093946570779
Enkd1	207.189709982709	0.0604412805585248	0.229405014657519	0.263469744324282	0.792188540493324	0.960093946570779
Etaa1	305.785423133421	-0.0466915863349461	0.176556310967722	-0.264457192603454	0.79142764625338	0.960093946570779
Fbxl6	318.612447752076	0.0341135066108206	0.130401832355725	0.261602969793874	0.793627554173125	0.960093946570779
Gen1	32.3623838287013	-0.106401665582365	0.40622977242607	-0.261924833689361	0.79337939329336	0.960093946570779
Gna13	55.4134230868709	0.0998674046573744	0.378630545721035	0.263759503257178	0.791965241506719	0.960093946570779
Ing1	351.564345240341	-0.0451661149495446	0.172852585440134	-0.261298463280363	0.7938623515422	0.960093946570779
Josd1	678.913678123099	-0.0297930989124625	0.11319035519815	-0.263212345789595	0.792386915655066	0.960093946570779
Kitlg	392.191276415081	0.0460076293938124	0.174951814540457	0.262973147861655	0.79257127579152	0.960093946570779
LOC100365504	712.884371104607	-0.0486392926909099	0.186512726872303	-0.260782701033646	0.794260085542696	0.960093946570779
Mfhas1	73.0092235147223	-0.0832718319197289	0.316904963653481	-0.262765943959093	0.79273098613025	0.960093946570779
Mrpl22	832.207424851073	-0.0375936510864741	0.144170555031158	-0.260758176857608	0.794278998881774	0.960093946570779
Ndufb7	2940.88915157913	-0.0390652477346624	0.147371113371577	-0.265080766786124	0.790947243384595	0.960093946570779
Nup37	242.55751037246	-0.0462375366125992	0.175998312339497	-0.262715795384492	0.792769641370819	0.960093946570779
Pbx1	136.118800251495	-0.0742486410862624	0.280982716117502	-0.26424629284035	0.791590141814939	0.960093946570779
Psma7	3879.8923890063	-0.0353577352996506	0.135323372361046	-0.261283285235571	0.793874055442544	0.960093946570779
Ptdss1	657.71049042245	-0.0453778198184402	0.17188591579469	-0.263999639578626	0.791780196477039	0.960093946570779
Rab22a	774.313316059087	-0.0283444999151697	0.107824924522535	-0.262875212207969	0.792646762361	0.960093946570779
Rdh16	1584.00036948992	-0.269646417698302	1.02409102599656	-0.26330317408641	0.792316913418048	0.960093946570779
Renbp	430.581008515219	0.0405407902670119	0.153635780800391	0.263875967276686	0.791875494818377	0.960093946570779
Rnf166	374.67097015156	-0.0358820597058628	0.135504896600121	-0.264802679505758	0.791161472614303	0.960093946570779
Rngtt	374.765699525644	-0.0399163169201421	0.152147942360106	-0.262351999645632	0.793050075586345	0.960093946570779
Sit1	133.530506687642	-0.0544565672796918	0.205348360923511	-0.265191146570564	0.790862214831892	0.960093946570779
Slc25a30	1829.48080397449	0.137097769852852	0.518225204492253	0.264552493133131	0.791354221390493	0.960093946570779
Spa17	274.061129258872	-0.0403840286156528	0.154014584451302	-0.262209119737111	0.793160222778017	0.960093946570779
Tapbpl	223.192341576694	0.0539390429743647	0.206233853102592	0.261543108286555	0.793673710410338	0.960093946570779
Tesk2	177.587277358548	-0.0651526856039356	0.246882928209235	-0.263901137581769	0.791856099048528	0.960093946570779
Tfdp2	303.097301984955	0.0415729347216684	0.159685352231377	0.260342818804263	0.794599346363514	0.960093946570779
Tle3	1646.8258207893	-0.0412941240966652	0.157335197979328	-0.262459542600829	0.792967172639911	0.960093946570779
Tomm40l	126.915653681681	0.0562800634709828	0.215066644467103	0.261686620956192	0.793563056124469	0.960093946570779
Triap1	518.40184443334	0.0449321061200425	0.169930285159898	0.264414939795829	0.791460200773793	0.960093946570779
Trim14	167.937366206195	0.0573738525286059	0.217274486187657	0.264061618716948	0.791732438294688	0.960093946570779
Trim41	785.108796432039	0.0245278824994558	0.0939940294623682	0.260951494895491	0.794129912971241	0.960093946570779
Ttc22	116.460714652351	0.0825613181631581	0.313957124213917	0.262970042071428	0.792573669633988	0.960093946570779
Uvrag	181.441766157099	0.0644815370427701	0.247026095434582	0.261031276591773	0.794068387915415	0.960093946570779
Wdr83os	765.531831719783	0.0329113675457545	0.124131459076857	0.265133172448873	0.790906873565738	0.960093946570779
Wfdc1	130.154921668605	0.108156190985169	0.409507176792575	0.264113053725436	0.791692805511807	0.960093946570779
Zcchc9	428.270857745376	0.0423020428416658	0.16144940549415	0.262014237291191	0.79331046576778	0.960093946570779
Zfp330	721.998719514624	0.0305952908248001	0.117257104705102	0.260924836083462	0.79415047166723	0.960093946570779
Sspo	1600.09852375686	-0.0513770509932276	0.197590665116639	-0.260017602364461	0.794850195749088	0.960244573751963
Tchp	461.705620145	-0.036227700862865	0.139369075602792	-0.259940741561029	0.794909483937376	0.960244573751963
Mterfd1	922.57521982488	-0.0516841641462098	0.198927235588082	-0.259814419043313	0.795006928026571	0.960260227837407
Ddit3	308.322330755292	0.0780228965869274	0.300766600388652	0.259413433825783	0.795316265710461	0.960454111264482
Tmem14c	1513.36310627361	0.0341829474355728	0.13178344247174	0.259387270467632	0.795336450397792	0.960454111264482
Psmb11	27.3170352196778	-0.1118356539442	0.431545514521738	-0.259151468804263	0.795518374464551	0.960571745900785
Pnkp	384.300016332543	-0.0386110351570039	0.149334764854193	-0.258553560483407	0.795979718365478	0.960822586973452
Spg7	1575.50324263878	0.0282114584937584	0.109039634890313	0.258726641208377	0.795846162561304	0.960822586973452
Zdhhc1	421.680228197699	0.0426155133217044	0.164800910191888	0.258587851681672	0.795953257471261	0.960822586973452
Als2	559.44895393605	0.0607802938703999	0.23575984132215	0.257805966993962	0.796556659128418	0.961124167884746
Fkbp14	100.46190411715	0.0803595701101593	0.31197759712658	0.257581220094963	0.79673012493973	0.961124167884746
Hddc3	1033.67007221444	0.0506100773247388	0.196488737959986	0.257572407712472	0.796736926782516	0.961124167884746
Hivep3	12.751083960626	-0.161541738685506	0.626845276489862	-0.257705920016004	0.796633876885069	0.961124167884746
Pter	8320.75098296786	0.0611303223376394	0.23706517201959	0.257862940460052	0.796512687043596	0.961124167884746
RGD1359310	409.835917449271	-0.0376053906353472	0.145956618792377	-0.257647724005177	0.796678794354121	0.961124167884746
Mdp1	390.822483428899	-0.0342805860202562	0.133158280831436	-0.257442389659954	0.796837283089472	0.961143219738399
Bin2	474.730711119855	-0.0331930188283913	0.129301347086104	-0.256710541509575	0.797402233252112	0.96128711946367
Dhps	450.044978955491	0.0355601806820086	0.138434179842701	0.256874282943812	0.79727582382785	0.96128711946367
Fundc2	489.621389850309	0.0456722762067005	0.177969019429511	0.256630487447227	0.797464037429434	0.96128711946367
Lmcd1	405.611013392629	-0.0714747296876583	0.278226791729666	-0.256893770881366	0.797260779370621	0.96128711946367
Pex2	382.052510428732	-0.034045140947427	0.132559604561552	-0.256828926580108	0.797310838695622	0.96128711946367
Tlk1	1345.88355030828	-0.0257957364707025	0.100348107559358	-0.25706251067509	0.797130517396256	0.96128711946367
Adc	40.2114704001228	0.0867197886219295	0.339088213680898	0.255744036870412	0.798148489097524	0.961295823623161
Celsr2	655.067751984015	-0.0652804933208024	0.254883825460987	-0.256118618757919	0.797859245666078	0.961295823623161
Cox15	1670.6137947953	0.0249727250106702	0.0979761765324206	0.254885686444466	0.798811391794256	0.961295823623161
Gsta5	3781.3612246185	0.0371325806491682	0.145705717265841	0.254846421581519	0.798841719459518	0.961295823623161
Iqcb1	154.278800403706	0.046354554570751	0.182018802400014	0.254669044953277	0.798978726622622	0.961295823623161
Map3k13	172.337719413072	-0.0686411607262071	0.268906909433395	-0.255259936871234	0.798522340911965	0.961295823623161
Ndfip1	5848.45034350281	-0.0305434691695517	0.119820559420944	-0.25491008652571	0.798792545644368	0.961295823623161
Pam16	1024.26154287086	-0.0483459495752046	0.189304698198665	-0.255386950430929	0.798424248749868	0.961295823623161
Phgdh	223.798657766291	-0.0794208062679689	0.309793300911626	-0.256367087455597	0.797667399215098	0.961295823623161
Prdm11	20.2010790906015	0.153805103534766	0.602057796756254	0.255465678483746	0.798363448926094	0.961295823623161
Psmc3	2831.33837132898	-0.0288046284810183	0.112558421037291	-0.255908249383449	0.798021684596458	0.961295823623161
Rhot2	1443.82390549071	-0.0355068051591184	0.139366880474056	-0.254772188617139	0.798899056856978	0.961295823623161
Rpl13	7505.45578361175	0.03980038164318	0.155444879374756	0.256041767366468	0.797918586263962	0.961295823623161
Spred1	43.1729135333827	-0.0920177673059291	0.361006425361698	-0.254892325569372	0.798806263851234	0.961295823623161
Srp72	1889.80294913776	0.0252708576726798	0.0989719105212194	0.255333634963647	0.798465423725724	0.961295823623161
Sugt1	904.775910220201	-0.0344706183430861	0.135364825489437	-0.254649745371083	0.798993634151681	0.961295823623161
Tomm7	2175.86867680775	-0.0416211146214858	0.163067214289148	-0.255239011734658	0.798538501632563	0.961295823623161
Ube2e3	778.550071193301	-0.025321133273277	0.0992317492900992	-0.255171691060811	0.798590494738953	0.961295823623161

Atxn10	2612.97938564405	0.0324333486040944	0.127502365849847	0.254374484645167	0.799206261092551	0.961348215723601
Rbpms2	1746.96688821798	0.0341710904389373	0.134333852966675	0.254374379088302	0.799206342633496	0.961348215723601
Ptpmt1	881.667517771055	-0.0282094190835066	0.110958353449655	-0.254234297882817	0.799314554993402	0.9613766383127
Ncald	714.643292052273	0.0301399127077761	0.118753033066418	0.253803308677758	0.799647517172561	0.961675344427397
Eif3e	2336.98962444838	-0.0320119424753445	0.126203814411962	-0.253652733275155	0.799763853301571	0.961713495199498
Mmachc	417.479239744958	0.0376221827129658	0.148567097962257	0.253233611135916	0.800087694834078	0.962001136092683
LOC303566	29.2905812698292	0.0975386699409918	0.385563362783408	0.25297701845127	0.800285972308887	0.962035998018277
Nipal2	362.168960340826	0.0521196482163827	0.205949779349335	0.253069696802038	0.800214355261204	0.962035998018277
Cyp1a1	1111.4780207392	-0.246455100460289	0.976355692239464	-0.252423478880936	0.800713754072859	0.962041493529822
Osbp	1907.08039877355	-0.0255991532453866	0.10141171524369	-0.252427968345396	0.800710284322339	0.962041493529822
RGD1559904	427.598496134694	-0.0333303989373478	0.131928755193224	-0.252639380160389	0.800546895956655	0.962041493529822
RGD1563365	308.443785441886	0.060625283499937	0.239914461774945	0.252695410903605	0.800503594392114	0.962041493529822
Ttc21b	3814.93390469066	-0.0529913231573197	0.209789854360362	-0.252592401662547	0.800583202255837	0.962041493529822
Dhx15	2431.98352829644	-0.0276528255950266	0.109608475475524	-0.252287293250435	0.800819009065917	0.962066256954149
Htra2	762.796823334018	-0.028409907594309	0.112855110217089	-0.251737892414969	0.80124366605411	0.962372979855333
Pbx3	302.040398577965	0.043115859532947	0.171268753187861	0.251743874643929	0.801239041799718	0.962372979855333
Dtd1	447.464253841549	0.0342840141907313	0.13629818082086	0.251536843589949	0.801399080555855	0.962457940574582
Atg4b	657.688027842296	-0.0285829457289792	0.113735620836074	-0.251310411978808	0.801574125836467	0.962566456868176
Ate1	820.151296461257	0.027059935225125	0.107799148625354	0.251021789783976	0.80179726262559	0.962576784848575
Ttf2	214.115496855189	-0.0589214965651657	0.234947941832784	-0.250785327615685	0.80198008601058	0.962576784848575
Vps11	1095.07173179651	0.0291146725661137	0.116044589844614	0.250892114876692	0.801897520895533	0.962576784848575
Vps18	399.203175107629	0.0329166987411857	0.131329509960619	0.250642058674064	0.802090861279329	0.962576784848575
Wdr61	1017.51253188035	0.0334670407997969	0.13330214748362	0.251061527751527	0.801766539833815	0.962576784848575
Zfp691	233.151441701419	0.0523251345010804	0.208726721753462	0.250687281731394	0.802055894469061	0.962576784848575
Ada	92.7910095643949	-0.104359810465634	0.418764867688463	-0.249208609694717	0.803199419914679	0.962689858848568
Arsa	678.979837424999	-0.0544829700263101	0.220168633801762	-0.247460181250732	0.804552104087085	0.962689858848568
Arsb	499.803627085795	-0.0523608558033377	0.210228073443808	-0.249066905982627	0.803309028113508	0.962689858848568
Bloc1s2	637.995898524171	0.0378154400761185	0.15281696079766	0.247455778983778	0.804555510672845	0.962689858848568
Cdc25c	30.6896747390864	0.101968738028239	0.411033887294409	0.248078664996305	0.804073542601114	0.962689858848568
Churc1	1040.35515279519	-0.0374497538368531	0.151046339964723	-0.247935526578132	0.804184291670728	0.962689858848568
Csgalnact1	52.81896972884	0.175281883258563	0.706736344130029	0.248015946419637	0.80412206873989	0.962689858848568
Cstf3	146.507439010638	0.0511046885394983	0.204381763652524	0.25004524682732	0.802552357821046	0.962689858848568
Ctc1	1076.9980273165	0.0301874842619733	0.121359507977654	0.248744286830265	0.803558589314088	0.962689858848568
Cyp2u1	66.8903783117547	0.0728288786965057	0.293050342694856	0.248520025695176	0.803732077729176	0.962689858848568
Eef1a2	17.1091250050347	-0.127548449375706	0.515096616210519	-0.247620437334764	0.804428096393968	0.962689858848568
Ell3	728.511791032323	0.0576961006748311	0.23225098166242	0.248421342557308	0.803808422069136	0.962689858848568
Fancl	205.505177263488	0.0497255163420925	0.199930542618357	0.248713956811553	0.803582052050967	0.962689858848568
Fert2	98.7491944236634	-0.0589568944906476	0.23675863893105	-0.24901686695292	0.803347734360645	0.962689858848568
Hps6	119.721946508601	-0.0583110867577701	0.233171192038316	-0.250078434853085	0.802526692715041	0.962689858848568
Iqgap1	1081.25877418678	0.0534943512582659	0.216069855227612	0.247578965616994	0.804460187134661	0.962689858848568
Mrpl34	863.35162947649	0.0373134747650787	0.149735745926368	0.249195504615361	0.803209556566078	0.962689858848568
Mzt2b	259.933991163877	0.0369495923908753	0.149284807106615	0.247510735399128	0.8045129842716	0.962689858848568
Nagpa	286.457861505192	0.0436082865296039	0.176196325408949	0.247498274600164	0.804522626642679	0.962689858848568
Prdm9	215.966909864648	-0.0534232960290735	0.213984457586472	-0.249659702539307	0.802850524180219	0.962689858848568
Sf3a2	487.021664362782	0.0316278890689198	0.127398283369714	0.248259931235765	0.803933298913884	0.962689858848568
Slc44a3	814.276603453921	0.0350001921151663	0.141441035964397	0.247454296955069	0.804556657505063	0.962689858848568
Smarce1	1595.50619676304	-0.0258950109534462	0.10426841553192	-0.248349520047315	0.803863987370069	0.962689858848568
Timm8b	2047.99945095743	-0.0400081753219521	0.160157214130614	-0.249805639659316	0.802737658178781	0.962689858848568
Tmco1	965.198149103661	-0.032386927632875	0.129785655940769	-0.249541656958267	0.802941822211759	0.962689858848568
Topors	894.221746094393	-0.0355251462808698	0.142450865394875	-0.249385261243544	0.803062784891479	0.962689858848568
Trmt10c	550.784511465153	0.0496877217082053	0.198683995625248	0.250084167835667	0.80252225928201	0.962689858848568
Vps72	404.718940635557	0.0346709445953523	0.13921501763198	0.249046009439917	0.803325191972321	0.962689858848568
Dtx3	491.662993956738	-0.0495539822602739	0.201114316971273	-0.24639708901158	0.805374859424242	0.96316189522474
Gng3	121.879786314936	0.0589617294516539	0.239244248914925	0.246449934404153	0.805333955872339	0.96316189522474
Hspa8	69864.007120559	0.0537779724683077	0.218017094865693	0.246668604136006	0.805164706123699	0.96316189522474
LOC310926	511.94196100441	0.24099218009691	0.976419939188627	0.246812022598767	0.805053705599325	0.96316189522474
Trmt112	980.71330272122	-0.0414760765928798	0.168202072924802	-0.246584812372809	0.805229559630956	0.96316189522474
Ict1	1129.66361844216	0.0351381995471733	0.142892238045754	0.24590698576589	0.805754235994017	0.963412859910372
Mrps12	693.21377149393	0.0353669577167561	0.143793529871806	0.245956530507918	0.805715882578977	0.963412859910372
Fcf1	809.950704329206	-0.0330432544675676	0.134455615160112	-0.245755853544824	0.805871232865255	0.963451397154358
Aamp	4009.81301672852	0.0266761919558928	0.109564600058927	0.243474552378649	0.807637796326161	0.963453967075014
Aarsd1	1087.59422746348	0.0340834530171245	0.139778827964759	0.243838451884269	0.80735593867998	0.963453967075014
Abcg3l2	196.231473978227	0.0693460918841385	0.284310008155755	0.243910132935413	0.807300421210043	0.963453967075014
Azi1	482.232250383134	0.0312366086061554	0.127419439794498	0.245147904091667	0.80634191220872	0.963453967075014
Cdk2	325.75077130183	0.0435221759315528	0.177776319210451	0.244814248179092	0.806600261119651	0.963453967075014
Clip1	459.105441521181	-0.0426118187450564	0.174915951275649	-0.243613109234988	0.807530474439593	0.963453967075014
Ctdp1	1254.93935678035	0.0307299415788741	0.12562037611692	0.244625454315408	0.806746453071176	0.963453967075014
Dnajc3	751.351693174373	0.0266316940281767	0.109462295010253	0.243295593479766	0.807776417752159	0.963453967075014
Dock8	2774.95503921364	0.0380559580956675	0.155376774986787	0.244926940328782	0.806513001517807	0.963453967075014
Ggct	14164.6454435612	0.0611003926604955	0.248917597838333	0.245464335149895	0.806096919980222	0.963453967075014
Grpel2	360.734868001258	0.035522122694023	0.145023083437777	0.244941162827115	0.806501988947198	0.963453967075014
Gtpbp4	11402.2881770922	0.0777871000480823	0.318996608701418	0.243849300983921	0.807347535914929	0.963453967075014
Hgd	12469.4056109601	0.0461838119360122	0.19022822629809	0.242781067955929	0.808175002437107	0.963453967075014
Lipt2	116.47180084659	-0.0631218211002724	0.260055404661401	-0.24272451165727	0.808218817636146	0.963453967075014
LOC361985	1482.96914930061	0.0312247691985359	0.128399741248395	0.243184050800617	0.807862821679631	0.963453967075014
LOC619574	474.732543521465	-0.0265762580942113	0.109011989270529	-0.24379206610255	0.807391865305941	0.963453967075014
LOC682988	214.996362521601	-0.0572109020873254	0.235738155560104	-0.242688341865553	0.808246839350718	0.963453967075014
Med28	1546.46229280962	-0.0384018786525307	0.156816826114893	-0.244883662065671	0.80654651238722	0.963453967075014
Med9	390.802381498086	0.0361305847350789	0.148620433934533	0.243106440874706	0.807922941776254	0.963453967075014
Nolc1	1561.4533055601	-0.0277409590638802	0.114081896155981	-0.243167058040048	0.807875984929904	0.963453967075014
Prx	123.635053344698	-0.0494875778922693	0.201547579134544	-0.245537942478752	0.806039933284044	0.963453967075014
Ptn	542.153447020917	-0.126852285238502	0.522686286199849	-0.242692966292979	0.808243256668172	0.963453967075014
Qtrt1	125.947646694993	0.0591458615894229	0.242297375043972	0.24410442572351	0.807149944983577	0.963453967075014
Senp6	1716.36250241103	0.0304315050596338	0.124440207127639	0.244547206743397	0.80680704581887	0.963453967075014
Slc2a4	333.798594308788	0.156368723156278	0.641152380153992	0.243886988485829	0.807318346640246	0.963453967075014
Trim37	531.469223742391	0.0579978580961487	0.237031579709542	0.244684097229657	0.806701042386566	0.963453967075014
Uqcrb	3761.98080293699	-0.0320832012296011	0.131085155624232	-0.24475083450006	0.8066493645549	0.963453967075014
Wdr25l	93.8816729418489	0.0615098774069673	0.253077107769535	0.243047970435087	0.807968236325708	0.963453967075014
Eif5a2	152.610309658304	0.0685623211943393	0.282933129462072	0.242326946033834	0.808526835964072	0.963484589805791
Rassf3	923.744174950937	0.0264689087966685	0.109191126171059	0.242408973373919	0.808463281951215	0.963484589805791
Rbm45	704.941392514447	-0.0402891638784282	0.166169651674389	-0.242458014880931	0.808425285655677	0.963484589805791
Commd3	1728.31460024159	0.0342849904952372	0.14237317056464	0.240810753593994	0.809701794690967	0.963817845979354
Cse1l	1016.54280959187	-0.0310545819906951	0.12908446343079	-0.240575675533138	0.809884004654196	0.963817845979354
Cwf19l1	358.586855902351	-0.0449770996918979	0.186257639149742	-0.241477879228022	0.809184759094418	0.963817845979354
Ifih1	173.804318849467	-0.047811270798513	0.198944388844158	-0.240324801701071	0.810078469350762	0.963817845979354
Ift172	546.575519786612	-0.0373669113548865	0.155447286619599	-0.240383168902321	0.810033225003601	0.963817845979354
Il3ra	569.610192818021	0.0390617907621257	0.162047874339925	0.241050929678882	0.809515643881043	0.963817845979354
M6pr	2365.76342495708	0.025130558013229	0.104292905713429	0.240961337123749	0.809585082202353	0.963817845979354
Mepce	324.005305904692	-0.0343902545179069	0.142269084826733	-0.241726827439637	0.808991840620311	0.963817845979354
Mrpl32	661.713872080359	-0.0400446392601644	0.166544821579108	-0.240443616802239	0.809986368435968	0.963817845979354
Nop10	1678.62894718284	-0.0356100203346745	0.147386868418736	-0.241609179411452	0.809083008650541	0.963817845979354
Npc1	288.560066592747	-0.0335874227256112	0.139607021220094	-0.240585483681797	0.809876402112115	0.963817845979354
Paf1	2034.13044751847	-0.0230978280091767	0.0960755981643753	-0.240413054412201	0.810010058978621	0.963817845979354
Rnf113a2	457.625223728265	0.0398184481912394	0.165556468827734	0.240512789824428	0.80993274937082	0.963817845979354
Slc25a14	85.7740209907859	-0.0715160045980558	0.296635887297077	-0.241090197311269	0.809485210145494	0.963817845979354

Slc35e1	652.179016568541	0.0239574299050926	0.0996107996718127	0.240510366185445	0.80993462802538	0.963817845979354
Anapc2	1489.56257519762	0.0227456726581755	0.0956802699676524	0.237725841135956	0.812093739815966	0.964172021036557
Dennd1c	162.242898054721	0.0462019176585622	0.193603438421851	0.238642030509245	0.811383170925466	0.964172021036557
Dhx33	77.1308575089365	0.0938087968393672	0.394341895217983	0.237886965541695	0.81196876534594	0.964172021036557
Ephb6	18.2430099491103	0.116243346633021	0.486134743141103	0.239117545645736	0.811014436903608	0.964172021036557
Fibp	753.248393620956	-0.0298471203258885	0.1249750846327	-0.238824565821329	0.811241620573596	0.964172021036557
Gpn1	476.298749510422	-0.0323896152853819	0.135689692587054	-0.238703579231723	0.81133544115176	0.964172021036557
Gpr108	800.706409584598	0.0258488428241154	0.108356028621281	0.238554726977496	0.811450874228719	0.964172021036557
Gspt1	1026.44149321485	-0.025054303113826	0.105413654449687	-0.237676069998922	0.812132345248798	0.964172021036557
Hrsp12	52391.710313534	0.0569070470119321	0.238659935811977	0.238444072392465	0.811536688143298	0.964172021036557
Igf2	12.6206238497758	-0.146325634570286	0.615176109652694	-0.237859748248183	0.811989875820512	0.964172021036557
Lig3	284.970103941267	0.0436519142417975	0.182239348035164	0.239530676072078	0.810694112587302	0.964172021036557
LOC100362347	573.991245085697	0.0356180456982257	0.149384589520076	0.238431861095277	0.811546158284691	0.964172021036557
Pigc	333.056683092295	-0.0309320308162962	0.129939725686003	-0.23804906969746	0.811843035776882	0.964172021036557
Pvrl4	109.564240919041	0.084249757676837	0.351375040175707	0.239771605958999	0.81050732010583	0.964172021036557
Rsrc2	1174.60385060349	-0.031161653522919	0.130870761800706	-0.238110125547927	0.8117956815196	0.964172021036557
Slc35e2b	81.8284301657851	-0.0646848250832656	0.270266997655642	-0.239336750858805	0.810844470266025	0.964172021036557
Smg9	357.450438064002	0.0330374576633817	0.138046744821121	0.239320801850063	0.810856836456761	0.964172021036557
Spata7	456.534580690517	0.028410360877782	0.119127141470884	0.238487724350591	0.811502835272813	0.964172021036557
Sppl2a	156.706664080141	-0.0624158770418969	0.262737158269495	-0.237560143578457	0.812222266396352	0.964172021036557
Terf2	111.073553735105	-0.0513954051147302	0.216370557785745	-0.237534189682236	0.812242398506513	0.964172021036557
Thumpd1	2916.51643575511	0.0558614982149895	0.233074882711718	0.239671892419582	0.810584626514434	0.964172021036557
Zmat2	1718.47038348449	-0.0330742505330188	0.139015612987989	-0.23791752467312	0.811945063013698	0.964172021036557
Eif4a1	7052.89414059024	0.0399360774029807	0.168646052085514	0.236804104864137	0.812808766901355	0.964251984169468
Hmgn1	1563.89243485346	-0.0393591612370185	0.166346163343143	-0.236609973118692	0.812959382451331	0.964251984169468
Klrb1c	33.951022767373	-0.127867849512107	0.540751671724341	-0.236463160815321	0.813073290188297	0.964251984169468
Rab3d	187.21668816977	-0.058596245619674	0.24728424210019	-0.236959076413503	0.812688538443292	0.964251984169468
Sirt5	804.09379701935	0.0335656392513309	0.14150891209525	0.237198058796027	0.812503142206078	0.964251984169468
Tbl1xr1	1226.99476799758	0.032651805601922	0.137891360854022	0.236793700487797	0.812816838877331	0.964251984169468
Trib1	205.038113706974	-0.0745193528583662	0.314862333722307	-0.236672808644328	0.812910631257713	0.964251984169468
Zdhhc23	66.6531578609884	0.094245200481232	0.398224068590969	0.236663747660202	0.812917661213719	0.964251984169468
Zmym4	549.036811989134	-0.0251106055369979	0.106184317863276	-0.236481300085486	0.81305921619989	0.964251984169468
Pappa	71.6933436385618	0.107168082723639	0.454181789375717	0.235958563796544	0.813464823648988	0.964615668815274
Osbp2	24.3110596613798	0.124629020242962	0.528767015253484	0.23569741804567	0.813667473558783	0.964755320724924
Cdkn2aipnl	880.070495586915	0.0327341265830264	0.13908399821816	0.235355087590172	0.813933141941511	0.96486901244339
Pi4ka	1324.5049784048	-0.026681751737907	0.113363996749014	-0.23536354136296	0.813926581063289	0.96486901244339
Acot3	630.679795635973	-0.0461531054447649	0.196393509374834	-0.235003211621814	0.81420624050738	0.964974540073034
Dcun1d2	60.4128804754957	0.0804981071178989	0.342673130907012	0.234912223508247	0.814276862030659	0.964974540073034
Tmem192	610.537746028447	0.0320282422317131	0.136268690275266	0.235037426183632	0.814179684852635	0.964974540073034
Aldh3a2	707.227474717379	-0.0474887250168902	0.202750186008264	-0.234222843154159	0.814811981923928	0.964988286282616
Csde1	8147.21154413161	0.0317786411856528	0.135702128796354	0.234179385891157	0.814845717790511	0.964988286282616
LOC691921	183.167577171952	0.0718611754390303	0.306443737039448	0.234500388662797	0.814596531490531	0.964988286282616
Ngrn	356.152814574996	-0.0335761961671626	0.143407027815672	-0.234132152925725	0.814882385123672	0.964988286282616
Raly	1675.15861614134	0.0269936206594119	0.114982694572875	0.234762463687991	0.814393103210588	0.964988286282616
Trappc5	720.420414088588	0.028490866476272	0.121687374241621	0.234131656253023	0.814882770696863	0.964988286282616
Zc3h15	2648.59853937489	0.0355120829357901	0.151672610265438	0.234136426304272	0.814879067648651	0.964988286282616
Sept5	97.681359575964	0.0676086244658124	0.299324188435392	0.225870902111894	0.82130182976245	0.965011325253145
Acadl	6431.83776351243	-0.0446827515308462	0.193455717394838	-0.230971470538914	0.817336966015539	0.965011325253145
Acap2	224.004084684518	0.0563368047754075	0.242863121766701	0.23196936762398	0.816561805999422	0.965011325253145
Agpat6	1416.44508056191	-0.0321407928480776	0.140931049799129	-0.228060408929674	0.81959927814941	0.965011325253145
Akap11	1029.18517536006	0.0321996057898235	0.1423856944156	0.226143545684008	0.821089777162411	0.965011325253145
Antxr1	41.4597178604039	-0.115923410207161	0.498946988984684	-0.232336125412954	0.8162769559647	0.965011325253145
Aox3	13.6175208295793	0.14430499805419	0.642804022239662	0.224492991738604	0.822373719549706	0.965011325253145
Ccpg1	1090.67831960272	0.0266049650576274	0.114700719013308	0.231951162002225	0.816575946404379	0.965011325253145
Cct8	3001.7476405583	-0.0301341228681372	0.134053477884814	-0.224791802074915	0.822141243877012	0.965011325253145
Chmp6	880.708442722375	0.0202572826555756	0.0899366714281866	0.225239408284648	0.82179303362218	0.965011325253145
Cntd1	136.270667918529	-0.049786629012001	0.216024581771179	-0.23046742460419	0.81772857361233	0.965011325253145
Commd4	847.435319131754	0.0276481762743151	0.119894903498809	0.230603432401859	0.81762290080737	0.965011325253145
Comtd1	300.5511992068	-0.0455086526691878	0.201913379298185	-0.225387009158917	0.821678216862801	0.965011325253145
Cpd	409.697090637433	0.0647369744813589	0.283890001501997	0.228035415614676	0.819618708068733	0.965011325253145
Cstb	1825.42243884569	-0.0377435929021177	0.168002929270037	-0.224660326258069	0.822243530681588	0.965011325253145
Ctnnbip1	273.717368727861	0.0412013024800784	0.179852640673707	0.229083667194116	0.818803887632954	0.965011325253145
Cyp46a1	23.0962463345593	-0.131489610659024	0.563669083305976	-0.233274477088266	0.815548276271136	0.965011325253145
Dbi	5187.95985759212	0.0290818987629995	0.128755312006468	0.225869506351229	0.821302915369712	0.965011325253145
Depdc5	429.4003451644	-0.0279026925212491	0.123137154343286	-0.226598484186674	0.820735971080506	0.965011325253145
Dhx36	845.297605775661	-0.0299328510170257	0.129245897849325	-0.231596139723687	0.816851706060095	0.965011325253145
Dlgap5	64.0753408538369	0.0871750046106591	0.384963298652878	0.226450170485642	0.820851310775433	0.965011325253145
Dock7	483.837571800998	-0.0351457483583059	0.156488344561831	-0.224590198437554	0.822298090625046	0.965011325253145
Dpm3	315.013137966361	-0.0334437059095416	0.147099309088307	-0.227354609051662	0.820148012810341	0.965011325253145
Exoc6b	378.543936820605	-0.0306328231442726	0.134505737399869	-0.227743617011705	0.81984556185047	0.965011325253145
Fam135a	494.63948116974	0.0300163751276134	0.130896784632831	0.229313311337708	0.818625408162966	0.965011325253145
Fam174a	297.182770314984	0.03849234951807	0.166830002719975	0.230727979922649	0.817526135101925	0.965011325253145
Furin	4204.3746495386	-0.0313631534516729	0.134962671086043	-0.232383911783117	0.816239843476804	0.965011325253145
G3bp1	3116.19201167433	0.0193452235265975	0.0843874553185478	0.229242882767026	0.818680144262062	0.965011325253145
Gle1	202.422108222296	-0.0516196275852661	0.229704509747513	-0.224721872644144	0.822195647863429	0.965011325253145
Gmds	225.778328556206	-0.045365196984834	0.194059779250548	-0.233769187824663	0.815164171393001	0.965011325253145
Gnal	34.8200164483391	-0.107151120119286	0.459463022025765	-0.233209453171788	0.815598765644619	0.965011325253145
Gpr146	201.877564011625	0.0641445177176493	0.284371503177134	0.225565912902651	0.821539055137864	0.965011325253145
Grcc10	1120.15970066907	-0.0384875681752154	0.169608862134455	-0.226919558865414	0.820486292963456	0.965011325253145
Hdgf	1563.19296198745	0.0267461053019677	0.116520793892187	0.229539332925546	0.818449753321265	0.965011325253145
Hook2	1492.6932475976	-0.0311940290243561	0.135472627110112	-0.230260752225626	0.81788915640733	0.965011325253145
Hoxc8	25.0085801589347	-0.0917737226101884	0.407479710178926	-0.22522280328974	0.821805950666057	0.965011325253145
Idi1	742.015969865558	-0.0546580912565741	0.235253353976518	-0.232337139227481	0.816276168598267	0.965011325253145
Ints5	338.960610578418	-0.0294303570172725	0.126093213269755	-0.233401594376941	0.815449575227481	0.965011325253145
LOC499331	182.661705154876	-0.0587407242992806	0.251778351952694	-0.233303315569868	0.815525884188228	0.965011325253145
LOC64038	284.1504000334	-0.0315672832550416	0.136983169013716	-0.230446437196096	0.817744880312751	0.965011325253145
Lrguk	43.0361151350096	0.103135993298475	0.448597012904277	0.229907891340514	0.818163344168368	0.965011325253145
Marveld2	140.0814387458	0.0619995760564137	0.275333443352074	0.225179968338004	0.821839272245347	0.965011325253145
Mgmt	245.875769549086	-0.0831982683388714	0.360773647450386	-0.230610713744864	0.817617243578575	0.965011325253145
Mphosph10	448.95830896457	-0.0386786559521516	0.166007342959131	-0.232993645116492	0.815766340451388	0.965011325253145
Mphosph9	147.211698953079	0.0507833631598918	0.222664348347849	0.228071370817556	0.819590756361725	0.965011325253145
Mustn1	36.2107183402094	0.129649196034282	0.57715991355228	0.224633057476779	0.822264745885379	0.965011325253145
Myl12b	3480.65903060751	-0.0244198007003363	0.105195357758762	-0.232137626798482	0.816431121014833	0.965011325253145
Nckipsd	178.796578538189	-0.040155025664508	0.175293955939664	-0.229072505376793	0.818812562838753	0.965011325253145
Nfkb2	581.250450061444	-0.0343865289388479	0.150578466910445	-0.228362857215825	0.819364162227558	0.965011325253145
Nsdhl	349.222012720989	0.0293329408421202	0.12817856660547	0.228844350650341	0.818989894485181	0.965011325253145
Paip2	2457.83791852198	-0.0402269573571151	0.176064265663018	-0.228478829622975	0.81927401241239	0.965011325253145
Pde4dip	637.433818878736	-0.0580796897093005	0.25185761234186	-0.230605258142708	0.817621482299797	0.965011325253145
Ppp4r4	25.4059963846659	-0.10121131972178	0.436633005589085	-0.231799516816715	0.816693732360507	0.965011325253145
Pyroxd1	631.736841568104	-0.0401844502773826	0.178842743628386	-0.224691533254942	0.822219251692275	0.965011325253145
Rab19	29.6099396430047	0.0930844263338134	0.411482053418483	0.226217463338906	0.821032288857239	0.965011325253145
Rab28	1044.50160988178	-0.0319406293325847	0.139232746807825	-0.22940457661638	0.818554479381167	0.965011325253145
RGD1306227	324.967658455045	-0.042600981681224	0.183782196833998	-0.231801460724204	0.8166922224611	0.965011325253145
RGD1307254	289.612248661785	-0.0337620547249653	0.145172295962119	-0.232565411335611	0.816098888630512	0.965011325253145
RGD1311564	53.3922895364853	0.110012676668018	0.47322640730242	0.232473663705993	0.816170140255856	0.965011325253145

RGD1565784	557.165173938264	-0.0287843553975101	0.126720975048896	-0.227147521445471	0.820309032884975	0.965011325253145
Rgs4	23.9933473272369	-0.15137607673862	0.666565317304658	-0.227098639561279	0.820347041888321	0.965011325253145
Rnaset2	1671.79251606174	0.0210599343110611	0.0933352298019178	0.225637568533938	0.821483318799976	0.965011325253145
Rnf44	363.564658184862	0.0400880820794295	0.177680505561624	0.225618910486079	0.821497831616681	0.965011325253145
Rtn4ip1	562.053787618299	0.0315254468169574	0.138592380334586	0.227468831553722	0.820059202840188	0.965011325253145
Sec14l1	900.343288835736	0.140576402440475	0.614705962336546	0.228688854596648	0.819110757998553	0.965011325253145
Sec61g	1245.80602241475	-0.0355862153670736	0.15653853085575	-0.227331987674435	0.820165601595884	0.965011325253145
Sepw1	135.274584117129	-0.0724616593844147	0.321249343298822	-0.225562046727709	0.82154206241398	0.965011325253145
Slc27a5	22.2782008525781	0.444092619211255	1.91549977866491	0.231841644753822	0.816661010303842	0.965011325253145
Smad3	337.289123669526	-0.0524448618803113	0.231853024845449	-0.226198739116173	0.821046851239028	0.965011325253145
Spetex-2B	43.6171844185867	0.176713087266588	0.773145671886246	0.22856376707828	0.819207988774955	0.965011325253145
Ssbp3	532.50183153296	-0.0302126233045846	0.131499310616174	-0.229754993870427	0.818282158848214	0.965011325253145
Sytl5	18.2626813699655	0.118160816041006	0.515575546728635	0.229182351239785	0.818727189223748	0.965011325253145
Tbc1d17	1053.42632734495	0.0217655360583892	0.0950029069538585	0.229103895409857	0.81878816588277	0.965011325253145
Tcea1	1806.18637049438	0.0330501384033178	0.145832540392845	0.226630752740692	0.820710877181456	0.965011325253145
Thap3	484.088129039398	0.0383361960636138	0.169559252372706	0.2260932124149	0.821128923628746	0.965011325253145
Thnsl1	91.7669030556323	0.0606837432678298	0.26562507533006	0.228456380454388	0.819291462828663	0.965011325253145
Thra	668.932968537558	0.0357083056017008	0.158615783023663	0.225124542595951	0.821882388756882	0.965011325253145
Ttc30a1	61.2428385362753	-0.0669763863738174	0.291867189304116	-0.229475558844096	0.81849931503694	0.965011325253145
Ttyh3	829.406387918153	0.0396341526080206	0.172465407314184	0.229809288861146	0.81823996642325	0.965011325253145
Tuft1	233.993215187001	0.101200149995372	0.439864085043645	0.230071409411229	0.818036280976669	0.965011325253145
Ubn1	932.797182644776	-0.0250646531332877	0.108191173695434	-0.231670036262353	0.816794305865574	0.965011325253145
Upp2	475.773000415457	-0.111444144381792	0.487507772249162	-0.228599728508192	0.819180035599298	0.965011325253145
Wdr63	29.6062844431202	-0.0959889446053282	0.425923682650587	-0.22536653516887	0.821694143079573	0.965011325253145
Xkr8	98.1761498453455	-0.0550990941655521	0.242491695970962	-0.227220540253676	0.820252256560863	0.965011325253145
Zbtb22	589.389664218377	0.0323372218745247	0.141644610760306	0.228298286118675	0.819414356861147	0.965011325253145
Zbtb5	213.011436269321	0.0376594818465334	0.164641558853349	0.2287361836757	0.819073969736631	0.965011325253145
Zc3h8	192.16420438988	-0.0610860979547644	0.268060979486128	-0.227881350250478	0.819738481644219	0.965011325253145
Zfp329	193.024925667669	-0.0481764252144813	0.20909143523129	-0.230408410374103	0.817774426420011	0.965011325253145
Eny2	1370.93549000937	-0.0391476636857354	0.17451834087726	-0.224318335190158	0.822509610286987	0.965046923638955
Myh7b	2064.47043823206	-0.0585650811777121	0.26117861563348	-0.224233829541	0.822575361436407	0.965046923638955
Zfp64	120.090965103737	0.0590334108970496	0.263393119251773	0.224126625117419	0.822658775570811	0.965046923638955
Mgarp	15.4083406022807	0.118110215108425	0.528034763941946	0.223678862025475	0.823007194891894	0.965356014152865
Arhgef40	395.537107263295	0.0469574713544224	0.211905132555447	0.221596668226691	0.82462787638782	0.965482384346487
Atad2	148.912139941438	-0.0449907824079708	0.206128927812238	-0.218265252167579	0.82722244651919	0.965482384346487
Atpif1	3307.57588446394	0.0367625465747997	0.164876265664277	0.222970519296307	0.823558451120674	0.965482384346487
Calml4	2790.88608162655	0.0455214265720925	0.205898525753719	0.221086704751553	0.82502492220071	0.965482384346487
Cdkl2	56.3556725156334	0.0893734785460117	0.40008985252806	0.223383517430609	0.823237031427231	0.965482384346487
Cmtm8	149.76679896222	0.0604883011793847	0.276621454764945	0.218668147887458	0.826908562811303	0.965482384346487
Cnppd1	1985.91900866559	-0.0199876743927298	0.0912896038358889	-0.218947980414742	0.826690570135026	0.965482384346487
Commd2	319.936035537396	-0.0305470908284788	0.140384327177332	-0.21759616221184	0.827743774959655	0.965482384346487
Cops7b	207.49435592685	-0.0378423115358491	0.173841037765981	-0.217683419416715	0.827675783286585	0.965482384346487
Cox8a	15384.3068828919	0.0380121942168099	0.173535976371252	0.21904503614564	0.826614965751056	0.965482384346487
Cwc15	2447.72230163373	-0.0397135817982922	0.178667172750052	-0.222276880453299	0.824098348670145	0.965482384346487
Derl3	44.7475208484757	-0.0904648183871841	0.411107541087201	-0.220051469131274	0.825831070384166	0.965482384346487
Dnajb11	1993.88412889942	0.0306298281989594	0.1406567113937	0.217763005372891	0.827613770252147	0.965482384346487
Dnm1l	451.620043842829	0.0359154924516112	0.161364185725859	0.22257412504549	0.8238669765245	0.965482384346487
Fam216a	478.678201533594	-0.0371623854093235	0.170023974413558	-0.218571442865649	0.826983900209873	0.965482384346487
Fbxo30	256.929430941415	-0.0333995410814547	0.151427812290588	-0.220564112868258	0.825431846644949	0.965482384346487
Fen1	170.46755064678	0.0393711875702223	0.177696887271873	0.22156374360112	0.824653509388974	0.965482384346487
Fras1	602.047166833298	-0.0460301931105117	0.208048054468307	-0.221247890196079	0.824899422083681	0.965482384346487
Gnl2	800.254349042165	0.0308706328917296	0.139674793272245	0.221017924340568	0.825078476472443	0.965482384346487
Heatr6	182.865866405453	-0.0410373159915445	0.188404013787711	-0.217815508101565	0.827572860940324	0.965482384346487
Klf3	995.323242849787	-0.0336224602535653	0.154513130031218	-0.21760260922015	0.827738751343137	0.965482384346487
Lamc1	1219.32109585537	-0.0477927102561502	0.218954434713696	-0.218276968532945	0.827213318268078	0.965482384346487
Map4k2	352.771833976345	0.0345456032214284	0.15488982031539	0.223033399813402	0.823509511853604	0.965482384346487
Med22	125.449798029228	-0.0458819360260805	0.210610922351471	-0.217851645649754	0.827544703395115	0.965482384346487
Mrpl35	940.986690031878	0.0290723986844895	0.132289076679649	0.219764166582636	0.826054828304988	0.965482384346487
Mrps28	166.16377891766	0.0608097457662911	0.278555279806989	0.218304050127595	0.82719221901571	0.965482384346487
Myom1	49.0058951867575	0.074059114351847	0.335798604787702	0.22054622412344	0.825445776830251	0.965482384346487
Nme1	1044.05748270752	0.0403047744088995	0.181490116123968	0.222076966336663	0.824253968370179	0.965482384346487
Nol12	219.823831870464	-0.0446453511858845	0.20303193936803	-0.219893241057788	0.825954300346601	0.965482384346487
Nop2	515.025227558667	0.0258856314095108	0.116742218135438	0.221733249744148	0.824521544778206	0.965482384346487
Nprl2	571.236318898265	-0.0263439905525102	0.119247417427513	-0.220918751288882	0.825155696702093	0.965482384346487
Nudt21	476.857597481829	0.0292035863630105	0.134003545786013	0.217931444960754	0.827482526395224	0.965482384346487
Optn	1611.00787092378	-0.023913636894233	0.109927121686251	-0.21754082638938	0.827786893846253	0.965482384346487
Pfn1	6247.63654559904	0.0261655400952526	0.119682912271915	0.218623858649141	0.826943065847644	0.965482384346487
Pprc1	290.130985957276	-0.0536623376049826	0.24374094150221	-0.220161361789505	0.825745487159972	0.965482384346487
Psmb5	2374.67726250875	0.0285785401452497	0.130493964733588	0.219002773067664	0.826647887607135	0.965482384346487
Psmc4	2059.22354632658	0.0323942413212044	0.148740367639201	0.217790515348079	0.827592334847844	0.965482384346487
Rad21	3674.80212381337	-0.0341971224398319	0.156405126867222	-0.21864451073182	0.826926977031769	0.965482384346487
RGD1304587	296.852365418748	-0.0382713675274794	0.17192444560299	-0.222605734706606	0.823842372788182	0.965482384346487
RGD1564804	292.081351622568	0.0365670002731296	0.165394021559964	0.221090217942806	0.825022186757684	0.965482384346487
Rtn4rl1	179.445531778621	0.0712521362955716	0.327214168032293	0.217753823815293	0.827620924427466	0.965482384346487
Sh3rf2	10.4799100268091	-0.164286572234779	0.740821010076325	-0.221762841496428	0.824498507395472	0.965482384346487
Slc25a28	1231.70881060456	0.0431617635456588	0.19389722687815	0.222601242114632	0.823845869637272	0.965482384346487
Slc35d2	152.526023941567	-0.0478025256280644	0.21545158226182	-0.221871313852659	0.824414062212793	0.965482384346487
Tctex1d2	208.103449133069	0.0407741483535302	0.186520659701734	0.218603925263466	0.826958594838094	0.965482384346487
Tex15	30.1873604427046	-0.095761697322439	0.429028395896132	-0.223205965475588	0.823375209280303	0.965482384346487
Tm6sf2	97.4671399735	0.057479238343288	0.261662243315511	0.219669592429428	0.826128487951365	0.965482384346487
Tmem231	240.45959208068	-0.0350357547291237	0.157733120251914	-0.222120469519454	0.824220103478635	0.965482384346487
Trappc2l	446.101491568718	0.0292412267335142	0.131602113670648	0.22219420279749	0.824162706828755	0.965482384346487
Txndc5	893.31177403798	-0.0291138846152334	0.131496119999572	-0.221404894800913	0.824777181500591	0.965482384346487
Ube2n	1409.55624412685	-0.0355569075329409	0.161623286652632	-0.219998666462967	0.825872193272389	0.965482384346487
Usp21	551.117127459197	0.0307457340321546	0.139281620874453	0.220745090695552	0.825290920079535	0.965482384346487
Ywhae	8652.71361409348	0.0230194001392062	0.104504001390203	0.220272906615843	0.825658619362486	0.965482384346487
Zfat	147.892356117141	-0.0522187556267631	0.23900626003772	-0.218482794628568	0.827052962423303	0.965482384346487
Zfp775	457.778997728097	-0.0402102051045704	0.1840418806408	-0.218483993776665	0.827052028207171	0.965482384346487
Armc8	695.231522938387	-0.0280576820552259	0.130183451816428	-0.21552418271095	0.8293586599709	0.965523228242456
Bnipl	11.6295827219416	0.220353567604066	1.03108531517344	0.213710315103265	0.830772966888495	0.965523228242456
Bub3	1059.2676947994	0.0284260231572464	0.130981362634754	0.217023419099046	0.828190094055722	0.965523228242456
Ccdc152	64.0961101931729	0.0723040192208228	0.33397421879252	0.216495810611481	0.828601290381535	0.965523228242456
Ccr9	21.8858031453913	0.0958805406733076	0.446399297044965	0.214786495650888	0.82993378209763	0.965523228242456
Dagla	40.7372686377833	-0.0947874027959093	0.440993975680521	-0.214940357517669	0.829813819413202	0.965523228242456
Eprs	2186.37978654655	0.0251865454147682	0.116363289192236	0.216447520430255	0.828638928107933	0.965523228242456
Fabp3	2041.43492295008	-0.0594881088963191	0.273949514382476	-0.217149897237141	0.828091529195589	0.965523228242456
Fam115e	12.6678829798242	-0.139042475755124	0.642588394894131	-0.216378753273364	0.828692526420438	0.965523228242456
Fbxl7	35.4061597025665	-0.0851483751124859	0.395889361449066	-0.215081240882096	0.829703979258478	0.965523228242456
Fosl2	60.0407473128349	-0.0668957572791386	0.313467645588657	-0.213405620071302	0.831010597287698	0.965523228242456
Foxn4	62.949825683244	0.058582932112303	0.272787060085766	0.214757005313537	0.829956775512321	0.965523228242456
Fuca1	2927.84808004429	0.0238357209299711	0.111703139430329	0.213384521254552	0.831027052740176	0.965523228242456
Gtpbp10	327.684303050401	0.0372799721646116	0.172495670552593	0.216121204927547	0.828893271144473	0.965523228242456
Hsd17b7	63.0919039402502	-0.0623791763723497	0.28982096056161	-0.215233488466371	0.829585282696139	0.965523228242456
Leprotl1	295.39967919402	0.0296237687174988	0.137170379962085	0.215963305822197	0.829016350310385	0.965523228242456
Lmo4	1332.61068608273	0.0383942080156967	0.177987790938692	0.215712593617848	0.829211784035436	0.965523228242456

Mxd1	188.450776511862	0.0386953111665033	0.180533026316278	0.214339237291201	0.830282522021674	0.965523228242456
Nsmce2	456.618078267375	0.0305445714813243	0.143022864575761	0.213564254721975	0.830886876848118	0.965523228242456
Osbpl6	116.325226261218	0.0835947469198391	0.391099465197931	0.213742933341862	0.830747528975202	0.965523228242456
Parl	906.703839896196	-0.0288848977625499	0.135384105568015	-0.213355161902951	0.831049950897057	0.965523228242456
Phf5a	912.759139127489	-0.0288602691710293	0.134419483383743	-0.214703021054162	0.829998867048053	0.965523228242456
Phlda3	162.65950902489	0.0708130306848735	0.327577581088074	0.216171785778693	0.828853845279894	0.965523228242456
Pick1	555.957807005642	0.0254261009602185	0.118879272579935	0.21388170038744	0.830639311010038	0.965523228242456
Pklr	5171.70141777076	-0.12229176709543	0.568679886926573	-0.215045001426646	0.829732233145141	0.965523228242456
Rfc4	208.886245973185	-0.0423895974649268	0.198076934691097	-0.214005722226234	0.830542594863863	0.965523228242456
RGD1565166	40.5249181807089	-0.101522767629129	0.475411032800925	-0.213547352973697	0.830900058458054	0.965523228242456
Rtn4r	19.7469472395469	-0.125596120240441	0.588213385580833	-0.213521356907614	0.830920332785119	0.965523228242456
Skp1	21065.9401668202	-0.0412749451941526	0.190397976327538	-0.216782478418511	0.828377867464753	0.965523228242456
Sntb1	26.0881215019495	0.130855277818512	0.610215054387828	0.214441248011797	0.830202978447804	0.965523228242456
Snw1	1199.92000659665	-0.02356436436478	0.110207042452871	-0.213819043141976	0.830688174084168	0.965523228242456
Snx3	3559.73951944512	0.0319044814092846	0.149094909626477	0.213987730964215	0.830556624858957	0.965523228242456
Ssr3	2182.4377801627	-0.0376682271278461	0.175407087679964	-0.214747463321283	0.829964215369718	0.965523228242456
Sub1	1266.242696803	-0.0322453835872903	0.150227251848171	-0.214644035556741	0.830044858607649	0.965523228242456
Txlng	269.337112326101	0.0372169368522836	0.173561569402217	0.214430746279068	0.830211167167122	0.965523228242456
Usp26	75.8871675971602	-0.0585158086401562	0.27268287455149	-0.214592899302544	0.830084730508046	0.965523228242456
Wdr13	246.468708748222	-0.0323556499377055	0.149265862096116	-0.216765236761711	0.828391304860633	0.965523228242456
Wnt11	15.2797519535782	-0.174437678432867	0.808743896817626	-0.215689638115691	0.829229678703856	0.965523228242456
Aaas	261.917120196466	0.0312311620535952	0.149294882673786	0.209191108859614	0.834299052984849	0.965560194270311
Adi1	2432.58120612109	0.0290562018372031	0.136718451863493	0.212525825454887	0.831696832131349	0.965560194270311
Ces1e	335.877758323416	-0.198571924701587	0.938113159215101	-0.211671612055552	0.832363236555538	0.965560194270311
Cnot3	480.747139901309	-0.0276492931035446	0.132549587647726	-0.2085958439722	0.834763755238793	0.965560194270311
Cox4i1	12397.4740342469	0.0338205300611179	0.161606807114427	0.209276642890241	0.834232284341764	0.965560194270311
Csnk2b	861.165179513874	0.0244035408928423	0.115952180137541	0.210462113466907	0.833307018493932	0.965560194270311
Edem2	528.201951102962	-0.0238641027223279	0.113880146156315	-0.209554549478462	0.834015356181085	0.965560194270311
Eif3i	1446.06667439663	0.0267961801615026	0.127811914690043	0.209653225417098	0.833938334813377	0.965560194270311
Galnt3	1160.8839014063	0.121888258561142	0.578960537517625	0.210529475953153	0.833254448647875	0.965560194270311
Gtf2h5	988.154680075138	-0.0419623698695101	0.200761237224228	-0.209016294428604	0.834435518436918	0.965560194270311
Igsf9	94.8640740044303	0.0613869980869845	0.294351636311444	0.208549878832788	0.834799641000189	0.965560194270311
Kcng1	107.208911678997	0.107447820922087	0.505674050724144	0.212484347907942	0.831729187555726	0.965560194270311
Klhl41	27.4431237663881	-0.0964027540665436	0.455362116521547	-0.211705696562885	0.832336643621182	0.965560194270311
Kxd1	755.390569343482	-0.0329196592140626	0.156694754613569	-0.210087818799341	0.833599132520934	0.965560194270311
Lamtor4	1553.54980092312	-0.0253375303472937	0.121266256619073	-0.208941308602318	0.834494056177841	0.965560194270311
Lmbrd2	102.736123291131	-0.0479330285289059	0.227442813138644	-0.210747606694818	0.833084224010118	0.965560194270311
Lpl	1981.96877576094	0.0497251617998251	0.235194341387713	0.211421590785019	0.832558310494128	0.965560194270311
Macc1	20.7760200280153	-0.106503630472054	0.50010717177476	-0.212961613995853	0.831356903454179	0.965560194270311
Mfap3	90.0830643863658	0.0658898378035872	0.313454315391529	0.210205553307778	0.833507245481257	0.965560194270311
Mrpl52	1339.21536212983	0.0212470635253959	0.100994600368837	0.21037821277376	0.833372495838579	0.965560194270311
Nampt	1102.85004038805	0.0252168142840436	0.119972355164096	0.210188540931389	0.833520522814982	0.965560194270311
Ndnl2	605.947939038229	0.0457784665139014	0.216069957955162	0.211868725051547	0.832209450483861	0.965560194270311
Ophn1	117.109901522348	-0.0657054280930955	0.31311785957906	-0.209842479702137	0.833790617101632	0.965560194270311
Ppib	3635.70775553194	0.0221628172485321	0.105654359475531	0.209767181955847	0.833849388176992	0.965560194270311
Rab33b	253.829610120774	-0.0452797109673599	0.215410663987112	-0.210201807697269	0.833510168744313	0.965560194270311
Rabggta	490.571093546337	0.0250036069178132	0.119907422717388	0.208524262728461	0.834819640075167	0.965560194270311
Rala	358.912666035481	-0.0360929470705568	0.171354037784323	-0.210633770509602	0.833173058396305	0.965560194270311
Rbl1	44.202629234583	-0.0952167805962732	0.44970688993526	-0.211730757805336	0.832317090813907	0.965560194270311
RGD1304931	38.4072480879507	-0.0705905221639896	0.332056113071634	-0.21258612440832	0.831649795185559	0.965560194270311
RGD1305420	65.0937093219253	-0.0563370911746551	0.268009472568932	-0.210205596968835	0.833507211405983	0.965560194270311
Slc10a5	102.829619272071	0.0751298521143935	0.353688791779885	0.212417961384396	0.831780974339533	0.965560194270311
Slc16a3	86.0394220566738	0.0664300256203692	0.31398532261659	0.211570480641503	0.832442141012391	0.965560194270311
Slx4ip	21.0380897102404	-0.0914516779290809	0.43557365314911	-0.209956863248967	0.833701340721028	0.965560194270311
Sptlc2	975.316550257155	-0.0228921887418369	0.107432535767714	-0.213084319179931	0.831261195266024	0.965560194270311
Stk11	1545.83254604507	0.0316009072253946	0.151283933655078	0.208884753733622	0.83453820638478	0.965560194270311
Surf2	422.820691364725	-0.0325415785236046	0.153811543051977	-0.211567856858492	0.83244418815519	0.965560194270311
Tgif1	585.899466505026	0.0591657762907936	0.282363115588673	0.209537907128714	0.834028346501189	0.965560194270311
Tmem243	745.175555904038	0.0457836129688524	0.216874227912089	0.211106747950757	0.832803974787843	0.965560194270311
Ubl3	2430.22593253388	-0.0299545590558998	0.143090147248536	-0.20934047264534	0.834182459032478	0.965560194270311
Yipf5	1397.33882233344	0.0208342166683274	0.0994363619191032	0.20952311876893 8	0.834039889712502	0.965560194270311
Zfp358	264.266731985617	-0.0343255001277511	0.164027478279882	-0.209266767298471	0.834239993257595	0.965560194270311
Zfp428	65.5625659926297	0.0588175679341931	0.277604798106337	0.211875184922643	0.832204410649846	0.965560194270311
Zfp639	228.13642105778	0.0352106180170353	0.167633314857854	0.210045467673848	0.833632186423722	0.965560194270311
Zfp709	98.9035055535831	0.0633400995748395	0.303753939245167	0.20852437249782	0.834819554375504	0.965560194270311
Mfrp	9.39986215510068	-0.187158177082505	0.899821951563878	-0.207994678010718	0.835233122818598	0.96594013776518
Cyp39a1	238.322988209484	0.048458565126982	0.234020033226666	0.207070157451205	0.835955067884631	0.965996887234328
Gspt2	22.5101190231528	-0.097422932799478	0.469945997355468	-0.207306655121455	0.83577037700914	0.965996887234328
Ppp1r3c	29.0548053261055	0.104668936330807	0.505670622242414	0.206990344557983	0.836017399137132	0.965996887234328
Rbx1	1525.63571390075	0.0316358543898784	0.152629542068645	0.207272156891163	0.835797317549407	0.965996887234328
Rgs3	253.831091818315	0.0702247525205134	0.338125675689663	0.207688317006032	0.835472340457368	0.965996887234328
Rinl	172.34684235635	-0.0460588760435084	0.222675013695618	-0.206843485845556	0.836132093666313	0.965996887234328
Sele	12.3741796558265	-0.16689547323836	0.806030177005661	-0.207058591600582	0.835964100370904	0.965996887234328
Shisa3	308.755612182589	0.161348309370394	0.779776362973078	0.206916132665545	0.836075357115579	0.965996887234328
Stk19	276.520132475702	-0.0383282532964351	0.185290589530647	-0.206854829452068	0.836123234349588	0.965996887234328
Vps4b	1749.94947723148	0.0204526736877917	0.098678994878263	0.207264714370302	0.835803129627382	0.965996887234328
Gas8	98.476247619691	0.0523932929822954	0.253448234527525	0.206721869970672	0.836227076542042	0.966008430935751
Mrps21	654.857388909316	-0.0301439806386337	0.145965210186871	-0.206514830486265	0.836388781365421	0.966097041564977
Ptger4	19.693394962899	-0.098058958751109	0.47528942507499	-0.206314202626405	0.836545485090005	0.966157438030871
Timd2	745.480459574555	0.0537184417731488	0.260478022440462	0.206230227294617	0.836611077344698	0.966157438030871
Atp13a3	12.8098758144742	-0.132803956423125	0.645069354651596	-0.205875469769995	0.836888187273131	0.966379268164829
Por	3634.45209449013	0.0509083880665452	0.24752896623917	0.205666386605259	0.837051516816512	0.966469681037838
Fbxo5	48.6578952361415	0.0705520300775229	0.344021104883813	0.205080528711605	0.837509208951149	0.966588235821079
Gtpbp1	531.410238706467	0.0254468841733195	0.124192853683671	0.204898135589465	0.837651711901291	0.966588235821079
Rpf1	457.62132558418	0.0348589993004863	0.170141972267681	0.204881833893657	0.837664448604401	0.966588235821079
Spr	2425.65061632987	-0.027421405800417	0.133487196112645	-0.205423490783918	0.837241268619235	0.966588235821079
Tekt1	50.7768602006375	0.0612211843959755	0.298765733285691	0.204913675081451	0.83763957075643	0.966588235821079
Wash	540.203265444879	-0.0307759361456263	0.150145908144128	-0.204973525592745	0.837592809374846	0.966588235821079
Bace1	179.774330719036	0.0494314933943884	0.243825775330337	0.202732846137445	0.8393438487395	0.966609549019681
Cox5b	6627.99152000771	0.0261110964202591	0.127652920722446	0.204547583184815	0.837925611915227	0.966609549019681
LOC499339	642.439329294026	0.0235842632535292	0.11567970576811	0.203875546682369	0.838450754652343	0.966609549019681
Mxd4	355.461831234531	-0.0316770578564942	0.155329714586975	-0.20393430800233	0.838404834509983	0.966609549019681
Ndufaf5	394.485089682162	-0.0294733465052702	0.145363935853389	-0.202755561977878	0.83932609282736	0.966609549019681
Orc5	346.494007647664	0.028507806148657	0.140482315128787	0.202928077619752	0.839191248072311	0.966609549019681
Pank1	4466.87842634098	-0.0322570606157589	0.159236769606487	-0.202572940254151	0.839468842017708	0.966609549019681
Pfas	384.525364321733	0.0452838308547932	0.222780239726786	0.203266819850488	0.838926488062379	0.966609549019681
Pigx	402.354021060254	0.0353035985953972	0.173723367827689	0.203217327852024	0.838965169737867	0.966609549019681
Ppwd1	193.140883693794	-0.0364968082142855	0.17962708760023	-0.203180982901149	0.838993576266217	0.966609549019681
Rab11fip4	219.844197606887	-0.0530685680979954	0.259267136913174	-0.204686828920271	0.837816811538245	0.966609549019681
Slc6a6	443.0003890252	-0.142892684835072	0.704411331404646	-0.202854040621598	0.83924911761421	0.966609549019681
Timm17a	1600.03483105309	0.0252938710084746	0.123988312813	0.204002058215139	0.838351890510673	0.966609549019681
Tipin	202.84283601964	-0.0331268345915359	0.162769255906498	-0.203520218895425	0.838728443972549	0.966609549019681
Tmem106a	1343.54264651945	0.0416348822884394	0.204618970356312	0.203475182266526	0.838763641615699	0.966609549019681
Tnks	125.570626850101	0.0592769846049181	0.292019509966185	0.202989809180154	0.839142997494899	0.966609549019681
Ttc23	214.103328593662	-0.0353080609588528	0.172924879903703	-0.204181497645191	0.83821166952698	0.966609549019681

Yipf3	1087.71469778236	-0.0194282519053369	0.0958654758070915	-0.202661612449846	0.83939952931828	0.966609549019681
Zfp191	230.97914935091	0.0481592981975307	0.237296497343337	0.202949890692446	0.839174198485208	0.966609549019681
Zfp367	161.6915947003	-0.0388382112603482	0.191308795627418	-0.203013202466588	0.839124713009309	0.966609549019681
Zfp786	32.3261829621795	-0.0847855052507359	0.416941100146855	-0.20335127724485	0.83886047922974	0.966609549019681
Atl3	104.602616222494	0.0850635396700932	0.423093650381423	0.201051326564243	0.840658440566243	0.966720881486429
Ciz1	601.782539755151	0.0306938110831363	0.151848404194909	0.202134564705326	0.839811526819519	0.966720881486429
Fahd1	8385.72905288264	-0.0396739955918916	0.197322906594335	-0.201061277054139	0.840650660081715	0.966720881486429
Kcnc3	170.8949812598	-0.0532198091234466	0.26391429779556	-0.201655649458875	0.840185936823505	0.966720881486429
Maob	25.5864344037237	0.100973562509221	0.502540554483699	0.200926197116488	0.840756283080534	0.966720881486429
Ndufa4	16022.6214855448	-0.0332000068659988	0.16515710589143	-0.201020759517448	0.840682341641041	0.966720881486429
Nphp4	228.083572346128	0.037715110815664	0.186796459156229	0.201904848657332	0.839991111460909	0.966720881486429
Pcdhga1	79.0878774068616	-0.0684644445487771	0.340410054162985	-0.201123450119946	0.840602046086434	0.966720881486429
Ppp3r1	2169.52196688089	0.029453364833509	0.146403079434774	0.20117995432351	0.840557865181604	0.966720881486429
Sc5dl	916.802001616013	-0.0360231704713639	0.178272199071087	-0.202068357596236	0.839863284559826	0.966720881486429
Sh3pxd2a	51.3546648817018	0.0875698041885929	0.434882393151519	0.201364335663234	0.840413700031337	0.966720881486429
Speccl1	356.526872567446	0.0439372667638877	0.21831998645943	0.201251692419156	0.840501773547881	0.966720881486429
Tmem147	750.465810987587	0.0208656041408228	0.103724543608786	0.201163614848196	0.840570641043258	0.966720881486429
Vat1	2439.90590646697	0.02700422938609	0.133844890554738	0.201757641018401	0.840106198053107	0.966720881486429
Rce1	455.619658523536	-0.0337241982924101	0.167962338910455	-0.200784285996335	0.840867250670871	0.966750674805293
Arvcf	446.395801426743	-0.0349935708633933	0.174633700436792	-0.200382691175115	0.84118129540649	0.966916120913245
RGD1565498	145.0295382176	0.054540005590275	0.272159787989617	0.200397002044827	0.841170103957874	0.966916120913245
Arntl	426.227185377485	-0.140376924359154	0.701055943953191	-0.200236408477733	0.841295693878419	0.966949828761463
Stau2	219.534240254266	0.0375002768427673	0.187401784397098	0.200106295483854	0.841397449860874	0.966969000517562
Mcl1	1663.52965031844	0.0318099882015545	0.159102656046263	0.199933734558806	0.841532406698464	0.967026320345237
Gstt1	2355.71790776972	0.0303703280539589	0.151988527199956	0.199819872022338	0.841621459117914	0.96703088398041
Strada	466.380303797058	-0.0246484460242212	0.123534226151008	-0.199527262947282	0.841850319327916	0.967196070906812
Tnks2	572.956873940914	-0.024423135282026	0.122536143985783	-0.19931372481298	0.842017343711386	0.967290188864323
Akirin1	867.831457043168	0.0258386525539012	0.129816270312524	0.19904017032454	0.842231321826641	0.967377294721894
Man2a2	457.4970841162	0.0405375337801219	0.203745066829833	0.198962038251354	0.842292439957818	0.967377294721894
Parva	540.899810826279	0.0335377224795231	0.168637118714976	0.198875091884173	0.842360454110327	0.967377294721894
Pex11b	819.875904409263	-0.0294496240366669	0.148217591163114	-0.198691827370595	0.842503817379471	0.967377294721894
Zgpat	372.482862837053	0.0283423328878462	0.142658367015623	0.198672769643735	0.842518726064757	0.967377294721894
Canx	13559.7465232291	0.0260371576521141	0.1312751846241	0.198340285916719	0.842778834119473	0.96743345460327
Flad1	924.141727701933	0.0196480636919984	0.099199446354637	0.198066263613577	0.842993219636704	0.96743345460327
Ipo7	1770.30316690389	0.0232306121046316	0.117197043472289	0.198218414188277	0.842874180882375	0.96743345460327
Mrpl54	1859.22309210245	0.0310497504797845	0.156757324632237	0.198075276881826	0.842986167784023	0.96743345460327
Vps13a	1137.12722264386	-0.0224748539109588	0.11323595443527	-0.198478071943186	0.84267103952702	0.96743345460327
Hagh	8387.4089594389	-0.0349235258108501	0.176464578465439	-0.197906719379889	0.843118046855553	0.967479022772358
Bdkrb2	141.712679153734	0.15793926087399	0.800381121275593	0.197330067733578	0.843569251264135	0.967541745999149
Cds2	113.842752215335	-0.0652620444162302	0.330903521346234	-0.19722378338774	0.843652419672181	0.967541745999149
Dhx37	366.674688804218	-0.0369194522038066	0.18702090059786	-0.197408161792528	0.843508143107879	0.967541745999149
Dus4l	41.4525270851801	-0.0637865620003495	0.323677574079444	-0.197068215744516	0.843774155824337	0.967541745999149
Fbxo6	827.229474560736	0.0221475640868688	0.112445610980324	0.196962459395096	0.843856915338565	0.967541745999149
Itgb2	407.107368287691	-0.034559922496168	0.175158215637536	-0.197306888348787	0.843587389181942	0.967541745999149
LOC361914	10445.3707437951	0.111084083018363	0.563429982376801	0.197156854432489	0.843704793045723	0.967541745999149
LOC681989	820.330791626581	-0.0190547959341758	0.0966136475413713	-0.19722675231795	0.84365009643527	0.967541745999149
Snrnp40	401.867467146093	0.0314028373977927	0.159520436142505	0.196857770434751	0.843938841266546	0.967541745999149
Apc2	21.9507035268007	-0.134607957024578	0.685490597022024	-0.196367328172487	0.844322666739028	0.96775686715878
Bcar3	408.861736539528	0.0593567699573547	0.302068747506349	0.196500864281258	0.844218156255364	0.96775686715878
Cdk18	407.246198006557	0.0267127909812038	0.136087267433236	0.196291625844489	0.844381915503574	0.96775686715878
RGD1359108	188.586603468245	-0.0378498567126954	0.193202564414806	-0.19590763107798	0.844682464272354	0.968003719390964
Bag5	708.097725223626	-0.0330680903009294	0.169250652154761	-0.195379396651851	0.845095944881108	0.968015517903665
Crnkl1	385.822623929421	-0.0352181895795176	0.180112654804969	-0.195534231715439	0.844974741810573	0.968015517903665
Pomp	1308.53490145837	-0.0315522705904992	0.161516235359119	-0.195350458239354	0.845118597937539	0.968015517903665
Syce1	133.114609754573	0.208165192365931	1.06431534493295	0.195586010628312	0.844934210703882	0.968015517903665
Zfp667	135.631365194801	0.0394660783954393	0.201621965898971	0.195742950027652	0.844811365373034	0.968015517903665
Sipa1l2	424.529344868239	-0.0493789181580106	0.253187574071335	-0.195028995159527	0.845370248590605	0.968206191604274
Fgd4	114.668540373973	0.0479499327829049	0.246225115349439	0.19474021857947	0.84559632475402	0.968367539259868
Arid3b	53.5334605958084	0.0605516831841357	0.311338024700895	0.1944885570669	0.845793354802085	0.968495594467107
Acp5	483.755129346115	-0.0327740720840151	0.168750978349276	-0.194215597471561	0.846007070381144	0.968568539545569
Zfp830	194.166607617975	0.0395030120893431	0.203425049346884	0.194189517053928	0.84602749081545	0.968568539545569
Smyd3	85.7939866605328	0.056396377867404	0.290699057244437	0.194002617008774	0.846173832758703	0.968638511746945
Calcr	36.7857563104394	0.0959516206178702	0.496464297993216	0.193269931001527	0.846747573925666	0.968709906918884
Catsperg1	278.286398426275	-0.0479859788032071	0.248173386772138	-0.1933566665924	0.846679650027293	0.968709906918884
Exosc2	239.100106463971	0.0302790221880303	0.156304041527847	0.193718741320171	0.846396116368648	0.968709906918884
Nsun5	201.056486192845	-0.0346351557669483	0.179174867982176	-0.193303648871078	0.846721168837997	0.968709906918884
Rttn	102.842453997076	-0.0475457653924043	0.245717442214921	-0.193497722277353	0.846569189708784	0.968709906918884
Tbc1d9b	1266.93273497475	-0.0265953603731619	0.137302542642578	-0.193698964791891	0.846411602469177	0.968709906918884
Nop9	354.831851833094	0.033208020220948	0.172159321129486	0.192891212645822	0.847044167092771	0.968756691806846
Pms1	123.357098967463	-0.0481837241495116	0.24977698814692	-0.192906978769276	0.847031819425383	0.968756691806846
Tsc2	689.757934157427	0.0272525803092327	0.141238372548895	0.192954505333161	0.846994597934512	0.968756691806846
Dlgap4	498.360880297871	0.0353096643488961	0.183385553573567	0.192543325582793	0.847316633836466	0.968873325974373
Nif3l1	332.924136424216	-0.034677555266254	0.180087481831109	-0.192559498937165	0.847303966386351	0.968873325974373
Commd9	1394.43691395624	-0.0239867545821321	0.124987354951112	-0.19191345229695	0.847809999899685	0.968936086202032
Grk4	73.8133484520651	-0.0585369272991887	0.305507261070893	-0.191605682607999	0.848051090914688	0.968936086202032
LOC683613	74.7833461387879	-0.0675058738625511	0.351956689993203	-0.19180164998101	0.847897578463476	0.968936086202032
Lsm2	139.346050083861	-0.0434199712551653	0.226614734112173	-0.191602595591479	0.848053509197414	0.968936086202032
Pde3b	346.563717442301	-0.0700541374557761	0.365249550771659	-0.191798011271399	0.847900428820267	0.968936086202032
Psd4	135.343356379805	-0.051650064543428	0.269532889888883	-0.19162805906441	0.848033561864984	0.968936086202032
Svop	47.8431915124629	0.0791495429758792	0.412861948833733	0.191709464142829	0.847969792184523	0.968936086202032
Traf3ip2	415.068699029414	-0.0273146465958246	0.14243190138762	-0.191773376116699	0.847919726642403	0.968936086202032
Acsl4	1103.93916846118	0.0273393660878015	0.143616850639688	0.190363219678112	0.849024517080144	0.969122200247668
Dgcr6	1083.7675004541	-0.0388802426941849	0.203770999222084	-0.190803612106796	0.84867945862663	0.969122200247668
LOC500350	96.4896514684274	-0.0543533599079643	0.284766548830092	-0.190869890200462	0.848627530595423	0.969122200247668
Mcfd2	1982.50802524695	0.0233455770210549	0.122486807124876	0.190596665625009	0.848841602750283	0.969122200247668
Nipsnap3b	99.3641301899486	0.0808696360352427	0.42474294475071	0.190396655282189	0.848998318434681	0.969122200247668
Nop56	780.702306194269	-0.0319822347785894	0.167936889769837	-0.190441985810396	0.848962799724471	0.969122200247668
Prkcdbp	237.912472705517	0.0620895725154509	0.326191352536182	0.190347083185057	0.849037160974789	0.969122200247668
Ptpn9	201.030442048298	-0.0445206819157073	0.233942140186752	-0.190306380373229	0.849069054202559	0.969122200247668
RGD1307597	1024.4777151221	-0.0234415728561931	0.122630156552258	-0.191156673980137	0.848402846778005	0.969122200247668
Rps6kc1	356.462210987035	-0.0267037260839352	0.140229689768642	-0.19042847579562	0.848973385456977	0.969122200247668
Als2cr8	94.6283246434568	-0.05100635708819	0.268531273415342	-0.189945686546898	0.849351691420474	0.969184642464349
Srsf9	679.425160790711	-0.0299048798995751	0.157468606832977	-0.189910106535041	0.849379572724563	0.969184642464349
Zfp451	276.059841367395	-0.0274240643773016	0.144361194900369	-0.189968394181195	0.849333897301832	0.969184642464349
Dusp1	2379.43315910068	0.106655786245663	0.565433530638695	0.188626567874722	0.85038550844286	0.969254123226844
Dyx1c1	33.8554894980344	-0.0992851258328162	0.526134966723821	-0.188706571720661	0.85032280064438	0.969254123226844
Fuca2	370.27521251282	-0.0276600970027461	0.146374762517319	-0.188967664418745	0.850118160217185	0.969254123226844
Glis2	3464.04255600713	-0.0359450105708928	0.190937341070994	-0.18825553120868	0.850676342983115	0.969254123226844
LOC500948	26.514264321426	0.0882862134151255	0.4672493955897	0.188948801750085	0.850132944147568	0.969254123226844
Nhp2l1	621.058689522093	0.0256373151588851	0.135901138451898	0.188646802013063	0.850369648637516	0.969254123226844
Pde7b	58.4601665263183	-0.0709004202029561	0.376320232185508	-0.188404486761704	0.850559582753806	0.969254123226844
Plekhm1	408.981555604775	0.0341116541575824	0.180264536134786	0.189231087206619	0.849911703733487	0.969254123226844
Pum1	793.754599219457	-0.0256340519091816	0.135261497304497	-0.189514772644243	0.849689377994067	0.969254123226844
Rassf4	30.9946331524195	-0.0810446684369996	0.430629789920137	-0.18820032968929	0.850719614051645	0.969254123226844
Smagp	2770.64841358171	-0.0187071726521626	0.0993199246056897	-0.188352666662123	0.85060020206099	0.969254123226844

Taf9	364.236555166395	0.0318265992029541	0.16879890336695	0.18854742873398	0.850447539407968	0.969254123226844
Tm4sf1	583.13943283865	-0.0503754056097676	0.266209809936519	-0.189231965650628	0.849911015273959	0.969254123226844
Tmem141	618.463178151446	-0.0233008289084407	0.122931594432816	-0.189543046406796	0.849667220355354	0.969254123226844
Zbtb6	110.457882996616	-0.0417885420160779	0.220575259481426	-0.189452534768963	0.849738153114781	0.969254123226844
Ficd	70.64582017336	0.0555474415165087	0.295482179180268	0.187989142596042	0.850885162397003	0.969345570392336
Hmg20b	1295.84100245404	0.0288891100294424	0.15389341461793	0.187721548067311	0.851094937645311	0.969390225601423
Rab2b	227.483401865217	0.0290565236746941	0.154756030506733	0.187756971922525	0.8510671672343	0.969390225601423
Psmd6	1760.37857474393	0.027124688329535	0.144705589249679	0.187447412848258	0.851309851261136	0.969537852648705
Slc43a1	50.1285403942313	-0.0759422517864401	0.40541332232504	-0.187320563002006	0.851409301393996	0.96955396449696
Bloc1s1	1290.47452954925	-0.0271620782089121	0.14521872069477	-0.18704253886111	0.851627280262673	0.969666345162601
Entpd1	36.0052434442902	-0.136059778591776	0.727681639202658	-0.18697706697789	0.851678613739068	0.969666345162601
Ears2	120.691407569709	-0.038193920063804	0.204649388792123	-0.186630999922508	0.851949959350434	0.969878128803789
Atg3	830.195524255872	-0.0302802238646967	0.164297965740287	-0.184300662082219	0.853777594557341	0.969961891199372
Atp6v0b	1036.78069800358	-0.0251314883554778	0.135854212792085	-0.184988656876911	0.853237932526278	0.969961891199372
Blvra	5647.25106547702	-0.0499192803209952	0.269163403335306	-0.185460875075981	0.85286756504835	0.969961891199372
Cblb	114.305597291158	-0.0381150390640435	0.206908801585628	-0.184211782060271	0.853847316902829	0.969961891199372
Ciita	25.412009738268	-0.112518794650397	0.608416161865821	-0.184937221761725	0.85327827577718	0.969961891199372
Ext13	262.14929198689	-0.0503281054348806	0.271996225605064	-0.185032367018786	0.853203648677671	0.969961891199372
Fam214a	929.959798655266	0.0306328824193235	0.16626925272346	0.184236603686866	0.853827845347803	0.969961891199372
Gcnt1	598.014702571794	0.0394655913200738	0.212838179743297	0.185425337538937	0.852895436518597	0.969961891199372
Ilf3	682.070665372221	-0.0226914379888069	0.122512040548563	-0.185218023364914	0.853058033067846	0.969961891199372
Kdm1b	1556.38806327615	0.0307289254089853	0.166800059742747	0.184226105532444	0.853836080711469	0.969961891199372
Lemd3	189.856400954736	-0.0331346135065624	0.179870705861345	-0.184213506851441	0.853845963871532	0.969961891199372
LOC691807	2666.81731775162	0.0383818532893464	0.206406438813016	0.185952790572181	0.85248178319236	0.969961891199372
Lrrc56	86.3495319322641	0.0513386238414596	0.277498490300812	0.185005056372767	0.853225069624684	0.969961891199372
Med1	430.230333529725	0.0286530464020521	0.155451204002282	0.184321804298353	0.853761009615954	0.969961891199372
Ndufa6	3481.02872983194	-0.0338873064245049	0.183517589429628	-0.184654269543462	0.853500216859935	0.969961891199372
Rab25	387.004581639019	-0.0379227357086746	0.204249578633589	-0.18566861171697	0.852704644501882	0.969961891199372
Rcbtb1	240.007713526337	0.0336238762535375	0.182353665707624	0.184388266191742	0.853708874224883	0.969961891199372
Sra1	1208.75393331578	-0.028436823226164	0.152601017664155	-0.186347533335248	0.852172234000365	0.969961891199372
Traf3ip1	167.267843391834	-0.0388042367803656	0.210358412779682	-0.18446724458321	0.853646921218384	0.969961891199372
Trerf1	69.7101768465962	0.0677601712059959	0.366680714460712	0.1847933870906	0.853391094869052	0.969961891199372
Unc79	113.378968287378	0.0567140897950864	0.308170842123782	0.184034574472513	0.853986331623448	0.969961891199372
Usp30	313.008833508194	0.0290926208264347	0.158014041391866	0.184114149414649	0.853923906600253	0.969961891199372
Zfp703	261.844571989956	-0.0556452759162817	0.299076763682092	-0.186056834476886	0.852400191885521	0.969961891199372
Carm1	327.318701166958	-0.0254322697683417	0.138640808939167	-0.183439998388216	0.854452794121099	0.969991912513074
Dkk4	116.984837407201	-0.0414244946612902	0.226067575697431	-0.183239434197908	0.854610154135661	0.969991912513074
Gpr157	21.8482103407179	0.0880428754831553	0.480281315949139	0.183315220807963	0.854550692282332	0.969991912513074
Gpr39	19.0215072976462	0.0980101124274263	0.533823973663215	0.183600057814676	0.854327217762623	0.969991912513074
Pcm1	982.890387149863	-0.0197202799291567	0.107416863959668	-0.18358644259584	0.854337899586626	0.969991912513074
Prss54	34.3585359346741	-0.113081153751219	0.616755120634806	-0.183348544613344	0.854524546827197	0.969991912513074
Unc119	152.307138759168	0.0404069333721116	0.219853283636535	0.183790447446366	0.854177850318454	0.969991912513074
Cdc26	190.23617383184	-0.0415893431030385	0.227589658629416	-0.182738281490893	0.855003377196331	0.970147587622392
Fignl1	14.0123788519516	0.106380714327041	0.581516895274853	0.18293658394355	0.85484777740258	0.970147587622392
Pias3	246.461777642549	-0.0349565910086443	0.191282379924682	-0.182748620246196	0.854995264660102	0.970147587622392
Fam19a5	103.54709565982	0.0494226554730352	0.270633817265273	0.182618181173536	0.855097617726931	0.970157668505121
Tubg2	20.7376738642401	-0.134053793461366	0.734736695285169	-0.182451474550806	0.855228432801574	0.97020923916785
Cops5	1478.58717541449	-0.0258866909586441	0.142265883206512	-0.181959935686528	0.85561416764041	0.970453111406137
Gpr37l1	19.3757986267009	-0.091375619166689	0.501981733381101	-0.182029769392659	0.855559363575245	0.970453111406137
Shmt1	1931.62915805956	-0.0275591419473867	0.151558711207782	-0.18183805950688	0.85570981524313	0.970464753547536
Ap2s1	2987.48560884401	0.0350484509036059	0.193407382658509	0.181215682782334	0.85619828531544	0.970921850832614
Pomgnt1	792.153706324001	-0.022967576342882	0.126902845876484	-0.180985510484426	0.856378949158165	0.971029841992389
Mta2	1352.3012182661	-0.0177113029729159	0.0980092807345333	-0.180710467826904	0.856594841928464	0.971080887029615
Rabgap1	872.734792989455	0.0217448307031777	0.120280640658233	0.180784127721464	0.856537022067388	0.971080887029615
Klhl9	1330.66497028979	-0.0189774058019009	0.105121949619542	-0.180527528937429	0.856738444446926	0.971146819545603
Anks1a	832.83828842565	-0.0313842398135232	0.173971652439081	-0.180398584329783	0.856839665635007	0.971164702792928
Ros1	791.602989685766	0.0456403326723067	0.253702823061671	0.179896826221805	0.857233566862525	0.971514280681968
Rpl6	6694.53600740621	-0.0259322981745016	0.144288229521741	-0.179725666192295	0.857367942825879	0.971569694731699
Prune	200.045827711918	0.0376710412327921	0.209744842987414	0.179604135654733	0.857463357699818	0.971571570860966
Zfp68	350.725945244523	-0.0313582954489177	0.174692375056044	-0.179505805212491	0.857540559464826	0.971571570860966
Oaz2	3790.01425847166	0.0198141102903931	0.110496282720929	0.179319247693028	0.857687034327152	0.971640669008513
Bcl2l12	93.8640332214379	-0.0414509441351422	0.231307655233969	-0.179202647198228	0.85777858521057	0.971647538320046
Pigy	846.826348657209	0.0226535919655968	0.12656725292552	0.178984622340879	0.857949776307982	0.971684770953005
Zc3hc1	398.569413430206	0.0280961866749834	0.157011909135192	0.178943029415633	0.85798243544645	0.971684770953005
Nos3	210.382297741211	-0.058968760064267	0.329775993847497	-0.178814592827933	0.858083286542631	0.971702165472107
Abtb1	537.418357768054	0.0197965417287525	0.110899452859804	0.178508921534345	0.85832331535135	0.97187714706948
Ccdc92	167.155933473614	-0.036638228680817	0.205710968306953	-0.178105372709865	0.858640222683724	0.972139134477857
Srd5a1	187.704383352448	-0.0372825536545532	0.209515114624246	-0.177946845130565	0.858764720799155	0.972183248665657
Spata13	167.267230051015	0.0570169717555581	0.320651829850744	0.177815831526981	0.858867613728163	0.972202897882114
Rplp1	15432.4533700534	0.0293681945701298	0.165397485474388	0.17756131228898	0.859067509972764	0.972332336023744
Smpd13b	60.2594890815656	0.0714542708928174	0.402982591872877	0.177313542402491	0.859262114038618	0.97235893948257
Srgap1	11.9044263048587	0.10877814851551	0.613275137342702	0.177372506876508	0.859215801233084	0.97235893948257
Ppcs	1287.70082446815	-0.0474111492425855	0.26810034530423	-0.176841059972471	0.859633236099715	0.97268206685011
Rpn1	4988.71062382356	-0.0209468654375347	0.118686700490571	-0.176488733370752	0.859909999223263	0.972898372603187
Apobec1	64.9645757240524	0.110222228559452	0.629435093329045	0.175112938137105	0.860990892189049	0.973127691031535
Apold1	527.129294065958	0.11412494242157	0.651919416884096	0.175059891553836	0.861032573442827	0.973127691031535
Car15	605.301122880009	-0.147743120840505	0.841616728585344	-0.175546796804816	0.860650003094257	0.973127691031535
Dab1	22.5885259769663	-0.103155584154135	0.590036106825238	-0.174829273939143	0.861213785300825	0.973127691031535
Eif2c2	51.4610496928213	0.0521265159728933	0.299172962272494	0.174235384029841	0.86168047838323	0.973127691031535
Fam162a	903.449532237199	-0.0191473367938041	0.109092167781322	-0.175515228849292	0.86067480562315	0.973127691031535
Fzd2	36.5952193349878	0.102956369832955	0.592303387735812	0.173823705831778	0.862004013438555	0.973127691031535
Gba	742.226915328176	-0.0277853517619087	0.159653868652784	-0.174034941942663	0.861838001541054	0.973127691031535
Gpr180	278.110475476502	-0.0270873757058401	0.156019173029594	-0.173615685686926	0.862167503843706	0.973127691031535
Hook3	154.132735661525	0.038603196182903	0.222313854984511	0.173642781668252	0.862146207816945	0.973127691031535
Isoc2b	1234.95608050395	0.0257244155029198	0.148115132184723	0.173678510247267	0.862118127172239	0.973127691031535
Kcnd1	15.0801475611597	-0.103889269802552	0.595990273803853	-0.174313700019781	0.861618933012698	0.973127691031535
Maf1	2048.34480979374	0.0194585334807014	0.111466149689252	0.174568992783445	0.861418314585351	0.973127691031535
Map1s	247.278807851972	0.0317370999954042	0.182622781118573	0.173784999883438	0.86203443336786	0.973127691031535
Mrpl21	618.826707745038	-0.0270281099052566	0.154148140595225	-0.175338539932371	0.860813630325768	0.973127691031535
P2rx4	679.547248798948	0.0907302097555692	0.522517197892711	0.173640619144174	0.862147907444295	0.973127691031535
Rab40c	56.5545661020883	-0.0490131415671281	0.281297884982412	-0.174239282212138	0.861677414939485	0.973127691031535
Ras110a	134.412355462507	0.0377014425189522	0.216321254282064	0.174284504054293	0.861641876843508	0.973127691031535
Stpg1	13.638027243723	0.122682089128289	0.699643202656652	0.175349504808232	0.860805015083902	0.973127691031535
Supt5h	2392.32018820991	-0.0185260513526343	0.106241276345197	-0.1743771534939	0.861569068108651	0.973127691031535
Tcte1	64.2213410428427	-0.0586172422288923	0.335133751494926	-0.174907009417641	0.861152702460835	0.973127691031535
Terf2ip	536.494159569982	0.0260872290877643	0.149135156627451	0.174923402889715	0.861139820934808	0.973127691031535
Tsg101	1305.91958281906	0.0200016782113587	0.11369937316381	0.175917225001247	0.860358972791151	0.973127691031535
Tspyl1	1199.06833810556	0.0155050543524493	0.0892269531002643	0.173770971816401	0.862045458411331	0.973127691031535
Ptpn22	18.8162046488457	-0.109770388970028	0.633201155201156	-0.173357846978589	0.862370156618102	0.973259775605337
Lyar	223.650794686132	-0.0350849179520805	0.202610875792526	-0.173164040749755	0.862522487926291	0.973335047435487
LOC100366054	29.1515998391921	-0.0696273395830771	0.402883193326564	-0.172822646207134	0.862790835785005	0.973466063108613
LOC689959	607.435814842273	0.0256072688304316	0.148191571473796	0.172798416102629	0.862809882083069	0.973466063108613
Als2cr12	39.7661601407411	0.0573935746464058	0.333117009192235	0.172292537044499	0.863207551198576	0.973528182865947
C2	115.766887420399	-0.0361853754856756	0.209977381707785	-0.172329872824269	0.86317820053422	0.973528182865947
Fam222b	360.074864813318	-0.0238615806077291	0.138475769828367	-0.17231592673075	0.863189163912717	0.973528182865947

LOC691141	15.3961041053711	-0.0917434779420247	0.53195757197981	-0.172463900834382	0.863072839053406	0.973528182865947
Csf2ra	225.738938623703	-0.0300750790330445	0.174834984337653	-0.172019799967274	0.863421962902135	0.973551889519037
Helq	165.633185168325	-0.0394398406239574	0.229495932689539	-0.171854203086516	0.863552151465348	0.973551889519037
Myg1	710.960751951948	0.0242210477880328	0.141031824470271	0.17174171772229	0.863640587055722	0.973551889519037
Tmem54	11.8739858171983	-0.135676310276394	0.790097191603969	-0.171721038523069	0.863656845154008	0.973551889519037
Zmynd12	46.0157518169105	-0.0616790440408885	0.358539610116801	-0.17202853548258	0.863415095338361	0.973551889519037
LOC687090	1080.46868607014	0.0212402333029215	0.123931370313351	0.171387060832276	0.863919428212051	0.973751311092639
Rcan2	1950.4667592086	-0.0217239817726627	0.126849265291481	-0.171258238845485	0.864020715855649	0.973768909907319
Anxa11	4589.12329993712	0.0179494900243758	0.104957414428818	0.171016884534147	0.864210489238386	0.973789671922622
Sbk1	259.567964634129	0.0304696117162877	0.178109691824919	0.17107217133495	0.864167017339421	0.973789671922622
Nubpl	286.26557869686	0.0271179295834942	0.159292112265417	0.170240253568297	0.864821195967455	0.974153187643555
Pank2	310.034947107517	-0.0272541602111521	0.159943344773492	-0.170398838724729	0.864696485350874	0.974153187643555
Psmb4	2844.97437449945	-0.0265122882677911	0.155981603337902	-0.169970609997883	0.865033250123005	0.974153187643555
Slc13a5	35.6304766578211	-0.0668689616854929	0.392938058567907	-0.170176851611682	0.864871055908412	0.974153187643555
Ttc4	132.821769941296	0.0399287017789534	0.234940101929354	0.169952687732126	0.86504734496625	0.974153187643555
Vwa5b2	18.6035250962434	0.0848078457162743	0.498741561812461	0.170043670329132	0.864975792769839	0.974153187643555
Gin1	150.551503334426	0.0335309078965255	0.19803966151143	0.169314104258809	0.865549582552307	0.974257452739707
Hspa4	4840.71205471919	0.0170548598588345	0.100638531928124	0.169466500872798	0.865429719658951	0.974257452739707
Kbtbd4	175.044384193553	-0.0298464827775794	0.176015678847055	-0.169567182725318	0.865350533126679	0.974257452739707
Mmp28	36.8584280479437	-0.0659222193377706	0.389404022827478	-0.169290031620903	0.865568516431952	0.974257452739707
Recql	431.365879358856	-0.0219677244359243	0.129529905594869	-0.169595772767973	0.865328047232059	0.974257452739707
Btaf1	791.369982927244	0.0233408787289051	0.138476802426966	0.168554431643634	0.866147125883211	0.974349809930695
Dtymk	533.716269646335	-0.0199573502412708	0.11815770778644	-0.168904345007623	0.865871881482857	0.974349809930695
Lsm10	283.400421041388	-0.0253798584385406	0.150308038165036	-0.168852303232605	0.865912816893111	0.974349809930695
Mrps18a	1119.84271628558	0.0200441352231474	0.118933838589407	0.168531811138674	0.866164919896159	0.974349809930695
RT1-CE2	578.059871545977	0.0442396762974109	0.262259973568469	0.168686344681038	0.866043360219117	0.974349809930695
Tfcp2	508.836261740753	0.0266503982485457	0.157635662714625	0.169063255037613	0.865746887065277	0.974349809930695
Crtap	215.892709686504	-0.0264376212907226	0.15705689560967	-0.168331490241775	0.866322501682286	0.974361227872892
RGD1564093	1592.0967922126	0.0287612938618621	0.170892041157113	0.168300955779561	0.866346521985171	0.974361227872892
Bmf	245.924543167523	0.0597015517355762	0.356072581885246	0.167666803828262	0.866845413135166	0.974440210235812
Hsd3b	41.3528401171733	-0.0842353560949607	0.501327585363	-0.168024578248508	0.866563943315037	0.974440210235812
Rnls	784.996666722364	-0.0238218265904453	0.142037015140082	-0.167715623754493	0.866807004318666	0.974440210235812
Slc27a4	844.051282346414	-0.0240737819128253	0.143543525786041	-0.167710677169156	0.866810896003972	0.974440210235812
Tubb6	69.7322644893677	0.059199419525019	0.353057996403348	0.167676189544188	0.866838028948822	0.974440210235812
Fgfr1	1346.29070034204	0.0295139666610489	0.176228589026407	0.167475475029913	0.866995943076155	0.974513042820765
Ercc2	561.703205783415	-0.0233422597245747	0.139763840523242	-0.167012151620813	0.867360487605715	0.974826391983245
Bicc1	624.93315444609	0.0417145484693249	0.251043526147851	0.166164605434825	0.868027412947861	0.974926699498151
Capn12	32.0333565120165	-0.0639797681196541	0.385564714067551	-0.16593781999575	0.868205884017071	0.974926699498151
Cldn23	248.165720435894	0.0636747537935715	0.382232414159145	0.166586483602252	0.867695429603918	0.974926699498151
Ing2	355.934695901155	-0.0331156151241414	0.199389393523178	-0.166085138928375	0.868089949144848	0.974926699498151
Ltbp2	117.809638928988	0.0816638635614108	0.49219500922715	0.165917699347745	0.86822171848674	0.974926699498151
Med30	194.732538692735	-0.0308920237078329	0.185785074851452	-0.166278285446413	0.867937953855867	0.974926699498151
Tal2	16.2378672698105	0.0911885585499833	0.548823795092248	0.166152705778101	0.868036777334614	0.974926699498151
Tp53i13	212.537277199676	-0.0401226454390393	0.241510437317913	-0.166132138571816	0.86805296265811	0.974926699498151
Zfp398	51.3498334550574	0.0553325443040161	0.332567448307336	0.166379916572236	0.86785797791848	0.974926699498151
Ahr	164.064014130244	-0.0485791633928097	0.293003133411319	-0.165797419390099	0.868316377044443	0.974936673069954
Brd7	950.656069592581	0.0192393532861278	0.116510063056216	0.165130399739333	0.868841345923864	0.974948241462202
Mest	195.485959876979	-0.170867170014317	1.03323349533535	-0.16537130356857	0.868651739109852	0.974948241462202
Nek1	134.495915986596	-0.0344495182462227	0.20851619932583	-0.165212671042366	0.868776592266163	0.974948241462202
Pard6b	1355.28837648421	0.021930137097109	0.132647902924573	0.165325923845016	0.868687455295044	0.974948241462202
Ssbp4	366.780466689784	0.0312154200008438	0.188991377361743	0.165168487772303	0.868811367688952	0.974948241462202
Wdr5b	150.151690229116	-0.0308243740078783	0.18632420543223	-0.165434082685998	0.868602329145327	0.974948241462202
Dcaf5	694.089177907776	-0.0169304307563573	0.102750191910158	-0.164772740971236	0.869122860296051	0.975167860821808
Acyp1	170.524149107255	0.0448048697351672	0.272400682985267	0.16448148824058	0.869352118519356	0.975328810491659
Arl3	1185.77658806944	0.0221458484372889	0.134773423321925	0.164319106033172	0.869479941683534	0.975375939318501
Tbcb	435.252832674395	-0.0244769682400654	0.149150145985125	-0.164109582852883	0.869644878067713	0.975464688060558
Degs1	1762.9263083726	0.0143044435083177	0.0874212974638576	0.16362652949908	0.8700251586592	0.975767762830491
Lingo4	558.664197099355	-0.0269374797673537	0.164901566976814	-0.16335490475443	0.870239006670595	0.975767762830491
Lmbr1	243.39957301697	0.0247740153279958	0.151883369487486	0.163112099840773	0.870430173089772	0.975767762830491
Mep1b	8686.04119705184	0.0221197441279424	0.135598144606575	0.16312718873935	0.870418292999435	0.975767762830491
Sesn1	672.54481388688	-0.0385256652808301	0.236011363810078	-0.163236484289936	0.870332241136053	0.975767762830491
Zfp653	192.871003405634	-0.0361834936622452	0.22154672506609	-0.16332217798052	0.870264772841458	0.975767762830491
Tmem41a	416.354919213749	-0.0243083641707782	0.149169155877708	-0.162958381226657	0.870551203537927	0.975807197180678
C1H6orf35	815.418369605598	0.0217761902095186	0.134136421767073	0.162343604538168	0.871035278634554	0.975813649050855
Eif4a3	1915.24344212514	0.0197763581654227	0.121533533697656	0.162723468689894	0.870736167928392	0.975813649050855
Glrx	194.643830329662	-0.0459820783337318	0.282766325828373	-0.162615114084132	0.870821486080326	0.975813649050855
Nrm	55.4506025807578	0.0553807389504456	0.341627630657001	0.162108488836047	0.871220421494395	0.975813649050855
Pwp1	409.319812755039	0.027824885074772	0.171661602007416	0.162091491337532	0.87123380652055	0.975813649050855
Rad1	149.061628008659	-0.0379548486675592	0.233864401396085	-0.162294254452506	0.871074138983812	0.975813649050855
Rpl18a	6190.43005687057	-0.0236664260131486	0.14601801062841	-0.162078814190774	0.871243789421791	0.975813649050855
Zfp3	115.941140905709	-0.034576626710294	0.21252624631753	-0.162693442854272	0.870759810047398	0.975813649050855
Agfg1	692.711502848185	0.0163650391320837	0.101089687786518	0.161886335692752	0.871395363416171	0.975833075130367
Max	108.197685897088	0.0377319785223153	0.233145527515919	0.161838740482547	0.871432844674751	0.975833075130367
Hint1	6574.53400376296	-0.0313907478436118	0.194178384766348	-0.161659331348254	0.871574132077735	0.975875470452082
Impa2	1041.76794263837	-0.0154675094745261	0.0957310080966496	-0.161572616668887	0.871642422666684	0.975875470452082
MGC95210	237.344553392173	0.0281819572870294	0.17457497963833	0.16143182342289	0.871753303897611	0.975903481936398
Dnajb6	947.950552628858	0.0221684901882345	0.13829664680125	0.160296657229104	0.872647391972939	0.976058750899549
Dync2li1	481.712390779345	-0.0228621988995106	0.142000821985274	-0.161000468728846	0.872093031465194	0.976058750899549
Eef1e1	724.791745941438	0.030947560451278	0.193175844281002	0.160204090560414	0.872720307213864	0.976058750899549
Fbxo46	197.943782841927	0.0296112876235087	0.184879386143752	0.160165436726865	0.872750755357392	0.976058750899549
Hbs1l	3062.10788270334	0.0229186279193871	0.142854125926507	0.160433783558886	0.872539378846507	0.976058750899549
Jmjd6	538.796952252435	-0.0304299334158775	0.188860865331792	-0.16112355178728	0.871996090948498	0.976058750899549
Nrbf2	334.547181794594	-0.0328735836514336	0.20451257642294	-0.160741134977683	0.872297289645594	0.976058750899549
Pnpla7	717.393323213663	-0.0212938448250779	0.132684698131207	-0.16048455567967	0.872499386724889	0.976058750899549
Tcf20	501.328274083167	-0.0243329528489786	0.151914210331338	-0.160175620147097	0.872742733722294	0.976058750899549
Tfam	1040.54364152278	-0.0203182683983548	0.126281046204012	-0.160897213074477	0.872174357351973	0.976058750899549
Fv1	468.366828420788	-0.0362219352243649	0.226365993113568	-0.160014915341954	0.872869324868559	0.976095311536407
Myl6	17004.0084943183	0.0228168466803114	0.142744195358057	0.159844304863521	0.87300372252091	0.976149563224069
Daam1	453.246966753692	0.022398172919862	0.140294520760814	0.159651088284826	0.873155932454762	0.976223719091169
Hsf2bp	56.8953765821416	0.0702079378905167	0.440954437290917	0.159218123128212	0.873497025806318	0.976347626690516
Nanp	120.378876442551	-0.0321919738587199	0.201971270505326	-0.159388876339573	0.873362502273447	0.976347626690516
Slc4a1ap	369.567934045386	-0.0223314675417839	0.14028775292445	-0.15918330058227	0.873524460304052	0.976347626690516
Tacc3	88.23061185827	0.0450125038126588	0.2833379688732	0.158865061367059	0.873775188043557	0.97643582610393
Zbtb26	37.0140581412027	-0.0589399793846945	0.370850368536034	-0.158931969293614	0.873722472955164	0.97643582610393
Xrcc6bp1	343.714350385747	-0.0287381024066243	0.181205896588683	-0.158593638218389	0.873989041324847	0.976578789195656
Dctn5	867.736068503072	0.0193152843036644	0.12194434478677	0.158394260409851	0.874146136218338	0.976615421832874
Pou2f1	14.8683756338351	-0.0884275388612918	0.558487619847048	-0.158333928486202	0.874193674267786	0.976615421832874
Hps5	457.555804195756	-0.0181052610470034	0.114511416921571	-0.158108785427079	0.874371077921378	0.976621626538363
Tmem11	384.234525147711	0.0276320102984552	0.174721323690942	0.158149043944587	0.874339355368758	0.976621626538363
B3galt6	63.0481193607289	0.0480727137257751	0.304537311095212	0.157854922777411	0.874571119028901	0.976653108555953
Serinc1	4965.49681825641	0.0234844781417126	0.14869030283415	0.157942230892537	0.874502320244097	0.976653108555953
Adh6	98.5016925533411	-0.0673331940582669	0.432894969430482	-0.155541641305859	0.876394328367493	0.976799931118608
Aurka	314.345590329906	0.0214313538653813	0.136248075181321	0.157296562442149	0.875011129310295	0.976799931118608
Bhlhb9	304.340105655638	-0.0211135479051734	0.134241775044743	-0.157280011368564	0.875024172808837	0.976799931118608
Camkk2	36.5659157105495	0.0628553067741324	0.399165272105827	0.157466871911312	0.874876914532968	0.976799931118608

Cnbp	7127.33891189743	0.0254132896651994	0.163415036847125	0.155513777406993	0.876416293276102	0.976799931118608
Cpsf3	1042.50872614673	-0.0177320382236511	0.113846976353477	-0.155753264527605	0.876227510430796	0.976799931118608
Esco1	517.000299694769	-0.026581945166379	0.171049400218628	-0.155405076734576	0.87650198213451	0.976799931118608
Glb1l	482.903778646164	-0.0177945719446768	0.11367776300466	-0.156535205077419	0.875611171622457	0.976799931118608
Hyou1	1046.11143761136	-0.0198796910464048	0.127967504267171	-0.155349525336526	0.876545773921036	0.976799931118608
LOC683519	657.956426931394	0.0292861748150176	0.187459551686406	0.156226634234192	0.875854383406281	0.976799931118608
Lrrc29	108.868446771629	-0.0335448055401441	0.2155210283723	-0.155645162764338	0.876312723989447	0.976799931118608
Nt5dc1	626.731910764353	-0.0282201493286464	0.179528979361859	-0.157189939078113	0.875095157185332	0.976799931118608
Pcyox1	415.723990213671	0.0451663013747891	0.288240787004898	0.156696426776068	0.875484103312261	0.976799931118608
Psma5	1319.34340689553	-0.0238546545771631	0.152049303313609	-0.156887628271217	0.87533341032105	0.976799931118608
Ptger2	10.5078179133878	0.129540898516717	0.831811641629085	0.155733452182773	0.87624312783381	0.976799931118608
Rdm1	285.829110241598	-0.035462155888925	0.227159690006346	-0.156111129962954	0.875945425484407	0.976799931118608
Rtel1	688.057613005557	-0.0800586541607008	0.510250199234151	-0.156900779815203	0.875323045267454	0.976799931118608
Sirt2	1718.70459546612	0.0187546166412965	0.119816651094308	0.156527631760754	0.875617140677969	0.976799931118608
Stx17	67.1605852740015	0.0468768125391641	0.300645410800898	0.15592059900162	0.876095608233368	0.976799931118608
Tecta	17.3194042408877	0.0849265911694587	0.546892903920251	0.155289254186123	0.876593286768414	0.976799931118608
Tln1	3420.81595414263	-0.0225942575611671	0.144422190966171	-0.156445885566572	0.875681570966968	0.976799931118608
Vps25	2162.85385502049	-0.020114789389716	0.129225929586719	-0.155655985250372	0.876304192865587	0.976799931118608
Gpr183	30.7263432151912	-0.0765584848050525	0.493394825082505	-0.155166777017271	0.87668983912478	0.976811754876213
Itpripl1	30.7756933124101	-0.0746812973896691	0.481932490803908	-0.154962155104118	0.876851152693325	0.97689572647641
Tab1	619.673821530104	-0.020821989251806	0.134672020087099	-0.15461258573488	0.877126747336147	0.977106989142669
Acbd6	385.326455561857	0.0246101619847903	0.160731265484169	0.153113720038583	0.878308596429965	0.977192652371228
Arl10	21.7903351300809	-0.0715156225702423	0.465051118398416	-0.15378013242186	0.877783099513374	0.977192652371228
Atp5g1	4708.70303913901	0.0208637270684178	0.13633769543002	0.153029776560414	0.878374793543541	0.977192652371228
Fancd2os	158.602477314149	0.0322272495436971	0.210222840892753	0.153300418769139	0.878161370448678	0.977192652371228
Gabpa	562.837844940808	-0.0205610434996916	0.133896030734216	-0.153559768627536	0.877956860585996	0.977192652371228
Kcnma1	14.566620103432	-0.095008027484801	0.621456787420952	-0.152879539507622	0.878493271329334	0.977192652371228
Magi1	206.752439958439	-0.0330584719778672	0.21472032860929	-0.153960606301144	0.877640796788324	0.977192652371228
Mogat2	24.7599691583637	0.0948188186989087	0.61568182946857	0.154006199566994	0.877604847358278	0.977192652371228
Ppard	679.134294282226	-0.0396098633698745	0.25845473547609	-0.153256481437303	0.878196017958203	0.977192652371228
Rpl7	14134.3947072263	-0.0232901367264847	0.151875081969025	-0.153350611729939	0.878121790245078	0.977192652371228
Traf7	1121.35357297408	-0.0144561274336852	0.0936420203532386	-0.154376500839617	0.877312881147171	0.977192652371228
Trnt1	904.764588871036	-0.023035355739057	0.150672276781968	-0.152883836569288	0.878489882604491	0.977192652371228
Unc5cl	231.066950528455	0.0371015584421068	0.241081070654076	0.153896605575239	0.877691260579106	0.977192652371228
Wdr4	154.587545886869	0.0336553465930449	0.220138627886933	0.152882512787946	0.878490926557325	0.977192652371228
Zfyve20	360.087754853263	-0.0285314899998598	0.185113476475013	-0.154129729197275	0.877507447843733	0.977192652371228
Aspm	105.396825432724	-0.0352533601293882	0.230820997663249	-0.152730299610005	0.878610965447162	0.977227931624665
Exosc7	424.42188283423	-0.0274426658047658	0.180113472207223	-0.152363204531378	0.878900476730182	0.977358655563571
RGD1560289	22.1579016789123	-0.0688641358658202	0.451905520676157	-0.152386135408975	0.87888239170973	0.977358655563571
Cep41	24.2488015675104	-0.0709847149350244	0.468327565758457	-0.151570652946863	0.879525581484485	0.977900911248188
LOC100365112	32.0830427143208	-0.128854072939564	0.850984148949588	-0.151417712184904	0.879646218247534	0.977900911248188
Madd	297.37874489942	0.0335169663633575	0.221316127778905	0.151443849572685	0.879625601375364	0.977900911248188
Med12l	155.870392355581	-0.0301883004818372	0.199649940768403	-0.151206157966538	0.879813092794098	0.977990768967992
Cdhr5	4006.04047641939	0.0140916106349348	0.0942976073731571	0.149437626547311	0.881208323357263	0.978057334448739
Cstf2t	776.40540080918	0.0159920922025959	0.107278743054593	0.149070465846693	0.881498030304495	0.978057334448739
Dhx30	1892.28470821573	-0.0160484518916754	0.107636873033849	-0.149098087294199	0.881476235139825	0.978057334448739
Dtx4	30.4607465741274	-0.0733019410530779	0.492128296818012	-0.148948844289246	0.881593998893835	0.978057334448739
Kpna4	484.303326876005	-0.0246860262783523	0.16500520856304	-0.149607557805795	0.881074245033242	0.978057334448739
LOC689840	22.5590949454289	0.0638938712738109	0.426700096994938	0.149739528356772	0.880970120630872	0.978057334448739
Macrod1	2009.0619609944	-0.0216403870011392	0.144244381386633	-0.150025857458768	0.880744214833012	0.978057334448739
Nuak2	2104.93002312368	0.0713842864958359	0.476195069341827	0.149905555709554	0.88083912842821	0.978057334448739
Polr2g	689.065231884278	-0.0230386836801503	0.154674157388012	-0.148949792707491	0.881593250513521	0.978057334448739
Ptpn14	187.37959825034	0.0531962364374831	0.355280280520085	0.149730337860606	0.880977371840476	0.978057334448739
Rab18	1462.78329913839	-0.0148454599704343	0.0992680024910677	-0.149549296831778	0.881120213436009	0.978057334448739
Serpinb9	181.659255904977	0.0493976161676599	0.327389452190797	0.150883346537602	0.880067737617774	0.978057334448739
Sptlc1	1074.93595584169	-0.0158829119237835	0.105247368277919	-0.15091030002615	0.880046475298544	0.978057334448739
Stxbp1	549.197491081583	-0.0807287483611468	0.536156576597133	-0.150569352097692	0.880315439195409	0.978057334448739
Tpm3	1484.76739226013	-0.0182423898307011	0.121358976757871	-0.150317597577453	0.880514049872494	0.978057334448739
Trpm3	42.1827696335034	0.075840114758501	0.506588725934799	0.149707466581604	0.880995417094294	0.978057334448739
Txn1	5214.60763589901	0.0320014878283524	0.212460612659876	0.150623155170803	0.880272994598728	0.978057334448739
Zfp213	237.980081855256	-0.0311734902278976	0.207187989992737	-0.150459928825944	0.880401762957872	0.978057334448739
Zfpm1	125.267208275198	-0.0371572101157537	0.248650034167242	-0.149435773215143	0.881209785683052	0.978057334448739
Znhit2	325.65325074871	-0.0257701487650646	0.17299834287434	-0.148961824355642	0.881583756558762	0.978057334448739
Npepps	1536.97239052888	0.0193537558716646	0.130238833003935	0.148602036929185	0.881867665621604	0.9780745401498
Rpp38	87.7503010363666	0.0397879389191746	0.26751295320527	0.148732756460747	0.881764512789344	0.9780745401498
Smpd2	559.625718896433	0.0188275272283755	0.126694008113355	0.148606295662619	0.88186430495668	0.9780745401498
Nck2	317.946653147459	0.0243327067203969	0.163867212029546	0.148490393038539	0.881955767153051	0.978076812319404
Lzic	291.958485420592	0.0222132905187778	0.150137889140586	0.147952596416071	0.882380178988297	0.97845201115912
Tfg	884.022571410759	-0.0239461242900066	0.161999282062357	-0.147816237116343	0.882487794743198	0.978475882845692
Nmi	162.498253020774	0.0325774522084943	0.220596495008703	0.147678920316522	0.882596168356198	0.97848534703969
Recql4	35.8440076135365	-0.0697289134026041	0.472458884676771	-0.14758726243514	0.882668508111732	0.97848534703969
Emc4	1605.14837269402	-0.0226017994116756	0.153976084650025	-0.146787726568367	0.883299572522614	0.978803055600276
Hyal1	68.2875381043668	0.0833307162689177	0.566991905409455	0.146969851727848	0.88315581671253	0.978803055600276
Phyhipl	1495.00959266285	0.111342368259262	0.758069023531384	0.146876293322983	0.883229664152613	0.978803055600276
Pole3	1843.9092051439	0.0162762510516152	0.110715725910225	0.147009387490382	0.883124610683445	0.978803055600276
Dhx8	284.304696063968	0.0241433083013234	0.164609190532185	0.146670475829859	0.883392123411014	0.978810184703606
Ndufa2	2301.15953839764	0.023671959776464	0.161660240402811	0.146430314080198	0.8835816980441	0.978880846138999
Noa1	181.529874846423	0.0321894303402675	0.219916005791155	0.146371475893557	0.883628143708088	0.978880846138999
Bpnt1	1603.43871574091	0.0192307278286309	0.131905034880563	0.145792219728716	0.884085418034128	0.978910361556346
Chek1	58.2102085865278	0.0477625834978257	0.327178128532619	0.145983424112238	0.883934473877569	0.978910361556346
Cpsf2	491.109685732469	0.0195026899988693	0.133704382670778	0.145864253731241	0.884028551104266	0.978910361556346
Mis18a	227.50012333964	-0.0271050310094622	0.18548719859233	-0.146128849943087	0.883819671906659	0.978910361556346
Scd	1364.33523041221	-0.13294096023034	0.911095934099722	-0.14591324058723	0.883989878985271	0.978910361556346
Casc4	190.633950372627	-0.0440480782109901	0.303998718324557	-0.144895605000424	0.884793296518378	0.978931238024713
Cntln	37.4186846832383	0.0551808812042502	0.3802172821847	0.145129860713288	0.884608342432934	0.978931238024713
Coa3	1462.88811337346	0.0182788866769605	0.126137842210511	0.144911997515028	0.884780353779899	0.978931238024713
Dhx58	182.663650042754	0.0298932488704223	0.205886581488226	0.145192798162671	0.884558651916054	0.978931238024713
Dnajc24	113.728428974275	0.0345978417278765	0.238208703429748	0.145241719675789	0.884520027606404	0.978931238024713
Ldhd	4178.81253983757	-0.0239913100543472	0.165429778488039	-0.145024132133996	0.884691818520715	0.978931238024713
Ramp1	14.3636775398262	0.100240651182997	0.688414570670357	0.145610879626482	0.884228578832292	0.978931238024713
RGD1566239	257.14004898794	-0.029772297330472	0.205171674335246	-0.145109199049693	0.88462465537511	0.978931238024713
C2cd5	965.77321314908	-0.0251844257677238	0.174448404664551	-0.14436604230432	0.885211430186921	0.978975104077457
Cyp4f6	731.486390169961	-0.021292953188822	0.14722504035515	-0.144628611664545	0.885004105897628	0.978975104077457
Dcaf10	290.559173516229	0.0235952084798944	0.163314145012732	0.144477433219608	0.885123475162169	0.978975104077457
Ndufb11	4308.93941122948	0.0230541615536511	0.159765534942924	0.144299967836537	0.885263603704742	0.978975104077457
Trim59	166.776132295626	-0.0506342348164208	0.350778778510475	-0.144348056149322	0.885225632309967	0.978975104077457
Styxl1	154.657676197353	0.0360420504506849	0.250242034693296	0.144028761973819	0.88547775765194	0.979116664410152
Gpx7	69.5011695615179	-0.0366782739360845	0.255166359432584	-0.143742592156922	0.885703736748064	0.979176020997812
Tmco6	322.343094253212	0.0189254994182413	0.131589792234962	0.143821941632437	0.885641076088337	0.979176020997812
Krt75	138.120851476148	-0.0511829287981966	0.356636145329929	-0.143515819886531	0.885882818115751	0.979183517524421
Nfatc2ip	34.36445008214	-0.059734519197411	0.416189744410671	-0.143527129151142	0.885873887085951	0.979183517524421
Btf3	6715.54955512146	-0.0227856098343066	0.159190810284578	-0.143133952227354	0.886184391003258	0.979345663972876
Tax1bp1	3907.2793834981	-0.0163327195175248	0.114201483503558	-0.143016701854104	0.886276990616304	0.979345663972876
Zbtb20	87.9153442958259	-0.0325963946853025	0.22794242991018	-0.143002751607706	0.886288008061323	0.979345663972876
Daglb	434.797307615909	-0.0183807086352445	0.12926950915752	-0.142189049490757	0.886930681146804	0.97935931684567

Dnaja1	5722.82039746645	0.0343960569526103	0.240815025163979	0.142831855816259	0.886422977710508	0.97935931684567
Itgb5	2560.22465401664	-0.0269990057884938	0.189612863267467	-0.14239015920776	0.886771835001395	0.97935931684567
Nadk	3254.29554801078	-0.0158626315295454	0.111618793797836	-0.142114342843336	0.886989689214265	0.97935931684567
Oscar	76.9164313381321	-0.0443356255309056	0.310985385474641	-0.142564980869562	0.886633756124162	0.97935931684567
Rap1b	440.964164241932	0.0243706919156434	0.170961799488586	0.142550511216808	0.886645184516723	0.97935931684567
Zfp143	266.523508599353	-0.0235062810607498	0.165050118391724	-0.142419049981902	0.886749016049323	0.97935931684567
Zfp763	65.1009965291828	-0.0607215876861517	0.427263554140479	-0.142117405282331	0.886987270292613	0.97935931684567
Mtmr6	2024.19094560222	0.0141741893746713	0.100019180933396	0.141714711542279	0.887305354095679	0.979538093566946
Tbx1	16.7804614423806	-0.103051022027151	0.727293295547941	-0.141691148066356	0.887323967214964	0.979538093566946
Itfg2	229.607385820643	-0.0251515916966852	0.177656027538999	-0.141574660005071	0.887415983677032	0.979544534376337
Plgrkt	253.456662385057	0.0220055184431292	0.155594156251545	0.141428951917407	0.887531083811802	0.979576451602733
Fmnl1	1796.55600922393	-0.0216116610469125	0.153224223294864	-0.141045982039818	0.887833616994952	0.979595875381897
Hexim2	90.3931866868382	0.0398932251889475	0.282990342944793	0.140970270482799	0.887893428487093	0.979595875381897
Irs1	33.9046732768697	0.054635791460366	0.387305037664183	0.14106656549027	0.887817356352933	0.979595875381897
Slc6a18	20089.8970103597	0.0187191071653903	0.132644600100237	0.141122270723758	0.887773350223442	0.979595875381897
Ankrd46	1049.08880963229	0.016566062385719	0.119164218905067	0.139018763668618	0.889435321759151	0.979806418831346
Ccdc68	41.030687096085	0.0580229909893403	0.415697933218878	0.139579695621891	0.888992084101449	0.979806418831346
Cisd1	11604.2713907264	0.0236063060654745	0.168409134009726	0.140172361815667	0.888523808361283	0.979806418831346
Csda	897.482017168741	-0.0152693983618574	0.109273754244698	-0.139735277399406	0.888869152510022	0.979806418831346
Ddx5	12080.0546334971	0.0160817089143909	0.115629464692658	0.139079679709112	0.889387185398274	0.979806418831346
E2f4	588.804159162094	-0.0155562013585681	0.110953289391444	-0.140204958716326	0.888498054117282	0.979806418831346
Gatsl2	56.7310489934502	-0.0482971013692446	0.34740171785001	-0.139023783958652	0.889431354668876	0.979806418831346
Lgr4	1292.68882069488	0.0221240335324163	0.158319363161833	0.139743068002372	0.888862996902931	0.979806418831346
Mapk11	13.8339207678706	0.0805245160719228	0.577626195280274	0.139405928487802	0.889129387850352	0.979806418831346
Ndor1	514.150846357287	-0.0195643962787257	0.140312982731886	-0.139433970383981	0.889107230054979	0.979806418831346
Nol8	159.676791999853	-0.0287914737735278	0.207457012292684	-0.138782842070956	0.889621752809051	0.979806418831346
Otub1	1846.40458660547	-0.016512316601738	0.118781495477891	-0.139014217116094	0.889438914499047	0.979806418831346
Ptpn6	607.270727524213	-0.0140509781895909	0.10088448210494	-0.13927789384868	0.889230557763124	0.979806418831346
Rab11b	2266.61313099872	0.0132245579916501	0.0946066270881454	0.139784689494624	0.888830110530803	0.979806418831346
Rassf9	128.867595774408	0.0446118224386728	0.321492187694158	0.138764872511032	0.889635953047641	0.979806418831346
Smc1a	1411.33492612456	0.0170913278513206	0.123125212261797	0.138812575729656	0.889598256204136	0.979806418831346
Srcin1	282.977363137022	-0.0318335200751729	0.227123294069761	-0.14015964415079	0.88853385639892	0.979806418831346
Txndc11	942.879225252489	0.0216877314997514	0.1545340638375	0.140342724194176	0.888389209310696	0.979806418831346
Pih1d1	462.285005102604	-0.0170925920030723	0.12348626390846	-0.13841694988637	0.889910901884949	0.980014272921648
Amigo1	36.9575456066718	-0.0595519757371457	0.431354257313071	-0.138058161540118	0.89019445131972	0.980231557227276
Arfgap3	1702.77474675658	0.0312156057030934	0.227385962240456	0.137280267416348	0.890809267078394	0.980549998630358
Arhgap26	50.6207205349706	0.0509299257250223	0.371058865248875	0.137255649965034	0.890828724780669	0.980549998630358
Pja2	1797.29602168849	0.0136989668794087	0.0996932076378003	0.137411235970849	0.890705750272914	0.980549998630358
Prelid2	50.418512169621	0.056582145250304	0.412214097086806	0.137263974352601	0.890822145154128	0.980549998630358
Anp32a	4496.98486555688	0.0201859321777606	0.147245039975203	0.137090744660466	0.890959068088707	0.980598505654715
Actr6	326.942085257167	0.023888305866838	0.176170696772775	0.135597498928265	0.892139482757487	0.980782940399784
Ap4s1	341.823406605885	-0.0234331643441675	0.171723670562587	-0.136458557328746	0.891458784489761	0.980782940399784
Ccdc34	185.471743938319	0.0244985927398032	0.179934931781859	0.136152510783751	0.891700716380285	0.980782940399784
Ddrgk1	2951.62316186388	0.0134441398636134	0.0984022166248362	0.136624359945771	0.891327720603189	0.980782940399784
Gart	677.446682811583	-0.0242016714715926	0.179030929580843	-0.135181510414177	0.892468365450438	0.980782940399784
Hsd11b1	19143.4098856414	-0.0244942435926413	0.180796363412419	-0.135479736042958	0.892232584829949	0.980782940399784
Ift57	952.326909168002	0.0245067835409443	0.181334530746426	0.135146810924909	0.892495799884459	0.980782940399784
Ift74	276.108607989405	0.0211375577664924	0.156194276207151	0.135328632263444	0.892352048030907	0.980782940399784
Ik	2177.9050803864	0.0136999577966145	0.101381838997802	0.135132267593918	0.892507298306789	0.980782940399784
Lmo2	51.5195908679186	-0.092156992258738	0.680011861163005	-0.135522624709993	0.89219867733833	0.980782940399784
Mob4	976.519564229005	-0.0203289896097974	0.149282928235977	-0.136177591436729	0.891680889572749	0.980782940399784
Pgls	1062.82839795831	0.0245449222394114	0.181000049353862	0.135607268213641	0.892131759332145	0.980782940399784
Rab26	28.9175843015679	0.0587908337375912	0.42985969008987	0.136767496680835	0.891214576062693	0.980782940399784
Serp1	3680.12191381554	-0.0182422481001564	0.134065094428633	-0.136070079821317	0.891765880345068	0.980782940399784
Sil1	1619.52003636072	0.0159696241535443	0.117475550139745	0.135939981847689	0.891868727892768	0.980782940399784
Styx	72.6642697144197	-0.0347153017575575	0.256893706380852	-0.135134886123256	0.89250522801213	0.980782940399784
Dcun1d5	563.966318976858	0.0139765646396377	0.10380083097307	0.134647907040973	0.892890262135769	0.980933929087181
Zfp18	333.642257670615	-0.0209703875551735	0.155761923652026	-0.134631025757113	0.892903609918703	0.980933929087181
Zfp316	189.675813042855	-0.0225096433355201	0.167058741031484	-0.134740889321546	0.892816742957568	0.980933929087181
Gopc	1077.22836676185	0.0155206414139492	0.115521997609203	0.134352259614257	0.893124030592886	0.980986444889712
Xpa	111.997157349216	-0.0328107866865993	0.244054781556029	-0.134440253443946	0.893054452889317	0.980986444889712
Abcf3	1650.80990453452	0.0131197244023484	0.0996976535864803	0.131595117140525	0.8953045478948	0.981059203065123
Acn9	139.424638448985	0.0285570567857302	0.226917860428504	0.125847549998065	0.899852601500542	0.981059203065123
Actn1	270.657829200814	0.0340822231996493	0.265043207553906	0.128591196560725	0.897681132597953	0.981059203065123
Adra1a	17.3865110969036	-0.0650555665228119	0.495447735165406	-0.131306618045379	0.89553275667856	0.981059203065123
Arrdc2	201.797640278513	-0.0569016938389276	0.441722978766791	-0.128817599658924	0.897501979046929	0.981059203065123
Atp2b1	585.407532022249	0.0185309891601991	0.138676974113427	0.133627008223025	0.893697526245511	0.981059203065123
Atxn3	171.090923387875	0.0289161231610049	0.227417389254894	0.127150009309953	0.898821670927987	0.981059203065123
Bag6	1344.47884930875	0.0133463911881838	0.101841595935091	0.131050491360034	0.895735365458006	0.981059203065123
Btbd1	1791.38989601998	0.0126922752432891	0.0951640254101708	0.133372618366904	0.893898699299259	0.981059203065123
Btg2	3903.27472862824	0.0605643807978915	0.461938390240831	0.131109217327264	0.895688909734143	0.981059203065123
Ccdc126	192.921804448844	-0.0240132395591646	0.18677548878988	-0.128567403114544	0.897699960736862	0.981059203065123
Ccdc65	184.836945713948	-0.0245418566826156	0.189858328455281	-0.129264051160106	0.897148715755733	0.981059203065123
Cct2	4078.52679367222	-0.0196320745166965	0.15592587926147	-0.125906453820765	0.899805973924462	0.981059203065123
Cd99	1949.19706360006	-0.0212365956627538	0.162220086066056	-0.130912245072453	0.89584472788384	0.981059203065123
Cdk6	84.5036315631161	-0.0352126792577493	0.268877181299863	-0.130961947337876	0.895805409708072	0.981059203065123
Cpsf1	581.704468093575	0.0187561703025325	0.145449320106675	0.128953303382761	0.89739459872694	0.981059203065123
Creb1	21.5513089194333	-0.0607146483410968	0.480421461840215	-0.126377885177182	0.899432806857259	0.981059203065123
Ddhd1	335.321414044058	-0.0241337913185668	0.18821845333961	-0.128222237991836	0.897973101955705	0.981059203065123
Dexi	513.046909007528	-0.0174915781606823	0.130903049994749	-0.133622388182583	0.893701179740812	0.981059203065123
Dhcr24	474.924067084569	0.0803881411657675	0.630137999079133	0.12757227985496	0.89848746932254	0.981059203065123
Dkk1	37.5250665721905	0.0634126298595334	0.474308987865785	0.13369476750771	0.893643942941651	0.981059203065123
Eed	282.499169325679	-0.0266478822907016	0.201708008727059	-0.132111176243677	0.894896356034291	0.981059203065123
Eef1a1	113695.491536521	0.0178345802384878	0.140612627616874	0.126834840801649	0.8990711194447	0.981059203065123
Exosc5	1265.0979845452	0.0208561947189173	0.161574528644662	0.129080956595483	0.897293590370713	0.981059203065123
F12	12.0711673664968	-0.0979451164521795	0.776964299245869	-0.126061283056694	0.899683414564378	0.981059203065123
Fam120a	3799.37613613546	-0.0105879941650424	0.0810434680945016	-0.130645867137573	0.896055456931067	0.981059203065123
Get4	1017.25230404945	-0.01568974520321	0.117524980447191	-0.133501364080295	0.893796885547982	0.981059203065123
Gmeb2	74.6565685942818	0.0349716757132414	0.274553083922838	0.127376736088931	0.898642228156024	0.981059203065123
Gnmt	849.904546761675	0.0271908904558895	0.213513039761001	0.127350022679299	0.898663370198763	0.981059203065123
Gpcpd1	270.130511216898	0.0284920292324649	0.221806413951152	0.128454487518741	0.897789313459713	0.981059203065123
Hpx	19.757397524453	-0.0622171218644931	0.487785516666307	-0.127550162394542	0.898504973508945	0.981059203065123
Hspa4l	247.620625719189	-0.0351723689718228	0.267660424583347	-0.13140668452041	0.895453601035665	0.981059203065123
Igbp1	1157.00213474591	-0.017287676627176	0.133759943117843	-0.12924404888499	0.897164542502869	0.981059203065123
Kat8	507.112642058773	0.0213662963107522	0.162037251957859	0.131860396622308	0.895094713953544	0.981059203065123
Lamtor3	604.194719323099	0.0182190958230061	0.1418462561335	0.128442556889616	0.897798754518234	0.981059203065123
Laptm4b	912.80806351702	-0.0216623188610361	0.162183973407108	-0.133566334613471	0.893745506686427	0.981059203065123
Mrpl55	539.905968475636	-0.0202365313386841	0.152309604803588	-0.132864446498829	0.894300585143318	0.981059203065123
Msl3l2	10.991862275819	0.0983959256874734	0.739718174036295	0.133018126552945	0.894179044973472	0.981059203065123
Mycl1	11.0273065023839	-0.101284564374872	0.777928045149673	-0.130197856995097	0.896409890019738	0.981059203065123
Ndufb4	3182.76991314085	0.0205594675349468	0.154789630484776	0.132821995055857	0.894334158939631	0.981059203065123
Nedd1	183.048954833155	0.0320296666382991	0.253167550005145	0.126515687486995	0.899323732004163	0.981059203065123
Ntrk2	25.1651803778217	-0.0779477056782684	0.618515872209578	-0.126023775913476	0.899713104160583	0.981059203065123
Nudt13	218.391049580581	-0.0206656721048629	0.162881838988595	-0.126875238106257	0.899039145363507	0.981059203065123
Nudt5	309.225491643315	-0.0207262227445904	0.16418478399882	-0.126237171556284	0.89954418806463	0.981059203065123

Palm	2607.06802856405	0.0131732576924899	0.102926183535023	0.127987429826419	0.898158920770372	0.981059203065123
Palmd	82.872385487325	0.0361862831350149	0.286004772080827	0.126523354389305	0.899317663466186	0.981059203065123
Phospho2	432.549703504481	-0.0257827483203726	0.19399406637798	-0.132904829522658	0.894268647384297	0.981059203065123
Prom1	2899.81122350242	0.0676752531670093	0.509154209357288	0.132917006131476	0.894259017292168	0.981059203065123
Psmc5	2387.57994097838	0.0184152216406359	0.14631012752569	0.125864299020603	0.899839343135315	0.981059203065123
Ptgr2	1889.24698300944	0.020735054035948	0.15690587971655	0.132149630551805	0.894865940600801	0.981059203065123
Ptpn12	1031.67198360414	-0.0203322941707002	0.160521755136669	-0.126663791791892	0.899206504918859	0.981059203065123
Rab6b	65.2812279530061	-0.0531213423168384	0.405816429027119	-0.130899930404958	0.895854469738113	0.981059203065123
Rad17	357.214803659699	-0.0191519397086845	0.152291060075263	-0.125758791745356	0.899922862146487	0.981059203065123
Rasd1	643.51749251157	-0.0306773971355544	0.232375430767271	-0.132016526163123	0.894971220169884	0.981059203065123
RGD1311648	1758.88256121972	0.0136452186822394	0.107387064578972	0.127065757274749	0.898888353469067	0.981059203065123
RGD1359158	311.495507272754	-0.017923310133182	0.138982012899082	-0.128961365282546	0.897388219525749	0.981059203065123
RGD1561161	141.221169583197	-0.0690338760932872	0.545524893432643	-0.126545785397438	0.899299908816391	0.981059203065123
RGD621098	378.338775452217	0.0289443762952608	0.219065069727518	0.132126844006956	0.89488396359889	0.981059203065123
Ropn1l	97.4100954596157	-0.0376175822992429	0.285570971224574	-0.131727612711939	0.895199744044501	0.981059203065123
Rpl13a	13084.7457980221	-0.0232152041586656	0.177544812018315	-0.130756871432947	0.895967641598104	0.981059203065123
Rpl28	6439.52673325153	0.0219647820859251	0.172048981373022	0.127665865328797	0.898413404513293	0.981059203065123
Rwdd3	139.874785863204	0.0281635022398847	0.223747102354429	0.125872031161646	0.899833222456227	0.981059203065123
Scn1b	616.486258373399	0.0328958540608333	0.254294676847623	0.129361158749481	0.897071880214874	0.981059203065123
Sec22b	680.40574006837	0.0147126323207302	0.111727183707845	0.131683551240333	0.895234596416848	0.981059203065123
Snap23	1342.48973701262	0.0231058563129597	0.172240660022335	0.134148674941002	0.893285010419212	0.981059203065123
Spata25	46.3035347068627	0.0452429932257913	0.346417811329443	0.130602387481645	0.89608985396937	0.981059203065123
Stk11ip	357.182300983826	0.0167067825993618	0.132059517657119	0.126509492808686	0.899328635245658	0.981059203065123
Stx5	990.6770031013	-0.0160132694485926	0.123942082196837	-0.129199616181704	0.897199699907885	0.981059203065123
Tada3	483.994303617188	-0.0187847425555311	0.143405687767309	-0.1309902197604	0.895783044240557	0.981059203065123
Tmco3	412.105236507834	0.0173366857021182	0.132882541705325	0.130466240934519	0.896197561650467	0.981059203065123
Tpp2	1657.52266600399	-0.0214312414976438	0.168130180241684	-0.12746814085869	0.898569887371549	0.981059203065123
Unc13a	92.4941401412513	0.0423847642657298	0.33669776386235	0.125883711788052	0.899823976210195	0.981059203065123
Urb2	149.116429001579	-0.029838717858986	0.234540092436882	-0.127222248226137	0.898764496919532	0.981059203065123
Vps36	489.695744183844	-0.0191995463952877	0.15234434606492	-0.126027298624565	0.899710315675916	0.981059203065123
Wdr53	153.206905464714	-0.0247604877014378	0.186936775760798	-0.132453807447289	0.894625357347707	0.981059203065123
Zdhhc16	559.830542488809	0.0136248216441607	0.104310551766528	0.130617865723271	0.89607760900946	0.981059203065123
Zfp322a	103.116292096288	-0.0322984206680097	0.249148359290035	-0.129635293445424	0.896854978716568	0.981059203065123
Zfp707	110.813517507928	-0.0325851921567153	0.253538223546294	-0.128521813006888	0.89773603708754	0.981059203065123
Abcc8	395.267648584558	-0.0291718595067518	0.235817232553092	-0.123705376366776	0.901548554637276	0.981243920263701
Ap1g1	837.354004452749	0.0157896440397038	0.126555393416585	0.124764686936168	0.900709844635675	0.981243920263701
Apba1	7.75302349052711	0.13273130736544	1.06173641623718	0.125013426435766	0.900512920970139	0.981243920263701
Cpt1a	3895.55877285882	0.0220313435560363	0.178427008213984	0.123475385125634	0.901730664943253	0.981243920263701
Cul3	2414.57164803454	-0.0172270171456994	0.137566653201609	-0.12522669371372	0.900344085053484	0.981243920263701
Cyb5d1	27.6070340964014	0.052261381985544	0.423067352269965	0.123529697352291	0.901687659289975	0.981243920263701
Fcn1	10.4610910742062	0.107610930612868	0.869326980613242	0.123786484271956	0.901484333490191	0.981243920263701
Foxm1	92.9612666895227	0.0344940776893863	0.277042802409748	0.124508117118919	0.900912973859661	0.981243920263701
Ifit1	32.2927874852236	-0.178398937217141	1.44101327998333	-0.123801036184209	0.901472811370825	0.981243920263701
Nasp	451.411526846097	-0.0230711927793042	0.185762326280043	-0.124197372208419	0.901159002804998	0.981243920263701
Rela	923.686120124409	-0.0145175187173112	0.117279069937156	-0.123786100325407	0.901484637497113	0.981243920263701
Rilp	210.069829325254	0.0263697962669991	0.211537343919466	0.124657877320414	0.900794406226348	0.981243920263701
Rps15	6878.79959112937	-0.0209692409035806	0.168627067098647	-0.124352758215937	0.901035976414086	0.981243920263701
Sav1	876.888284215818	0.0282146396766964	0.2260709334809	0.124804366674941	0.90067843031827	0.981243920263701
Stim2	574.761623797836	-0.0185315085152454	0.150030210725169	-0.123518512875998	0.901696515388466	0.981243920263701
Syce2	380.574840115734	-0.0207206183725758	0.1678150567955	-0.12347293960533	0.901732601368499	0.981243920263701
Tasp1	94.4766160422029	-0.0339758112698174	0.271501236490216	-0.125140539722889	0.900412289479765	0.981243920263701
Trmt1l	441.638919566697	-0.0207636398735232	0.165640984129023	-0.125353275233801	0.900243877217931	0.981243920263701
Wee1	275.960494672647	-0.0303530641115021	0.244752140370435	-0.124015520622465	0.901302986250847	0.981243920263701
Ptov1	1939.25214873565	0.0153577465276373	0.124541806770069	0.123313985286813	0.901858466698902	0.981272809722163
RGD1562747	283.644923675014	-0.0220791887131548	0.179183139600751	-0.123221351977372	0.901931817980594	0.981272809722163
Nceh1	181.91391493253	-0.0327210563999803	0.265807086942487	-0.123100767463962	0.902027303555373	0.981282765334071
LOC688146	29.0359810972707	0.0513219015966586	0.417317931333104	0.122980341229798	0.902122665210389	0.981292584245505
Ccdc101	324.395654781631	0.0218123861788611	0.178132713337609	0.122450198900416	0.902542484616587	0.981373565498147
Cdc73	168.892626285478	-0.0227884744720198	0.186010944504307	-0.122511471208041	0.902493961716224	0.981373565498147
Copa	2552.9342314711	0.0106292011250865	0.0867582770521547	0.122515124622597	0.902491068507653	0.981373565498147
F8a1	750.533357049428	-0.0186198515520687	0.152023130961187	-0.122480384625301	0.902518579825562	0.981373565498147
Slc7a13	120309.703661399	0.0276749133168132	0.226533780048335	0.122166827882836	0.902766897147295	0.981523680215817
Apln	73.782148161564	-0.0392612883370156	0.322578407231509	-0.121710838223708	0.903128029396027	0.981679843776505
LOC654482	118.678904452767	0.029870807605657	0.245103618510321	0.12187012083789	0.903001879322495	0.981679843776505
Snrnp200	2400.88548374418	0.0117448191382384	0.0965393989867818	0.121658299735702	0.903169639835554	0.981679843776505
Tmem97	414.029340663513	-0.017759179797908	0.146158095142441	-0.121506645120138	0.90328975165852	0.98171651533283
Asb5	27.111386744827	0.0525658417440977	0.439500089173449	0.119603711214158	0.90479707970747	0.981957835819038
Cnksr3	424.631922303114	-0.0150291447180982	0.125641380724857	-0.119619385200889	0.904784662817648	0.981957835819038
Dhfr	645.189811256863	0.031227137011528	0.259958931030119	0.120123347514112	0.904385437663194	0.981957835819038
Dhrs11	830.567454219367	-0.0186800427150196	0.154706721640479	-0.120744868205726	0.903893119270039	0.981957835819038
Ehd3	444.32239520172	-0.0780291077039736	0.650173192159898	-0.120012803734276	0.904473005355057	0.981957835819038
Fam53a	233.643934175622	0.0198116642238159	0.166574184323089	0.11893598221312	0.905326074392724	0.981957835819038
Gpatch11	350.75105100866	0.0190529652577012	0.159912787694647	0.119145976581202	0.905159706096978	0.981957835819038
Melk	1071.98931450721	-0.0280027428621617	0.235104473039494	-0.119107656694637	0.905190064763853	0.981957835819038
Mesdc2	3036.00025667025	0.0131622085010606	0.109370870542992	0.120344735629463	0.904210067690449	0.981957835819038
Metap1	1100.29711053892	0.0139637813883828	0.115748522891981	0.120638959698985	0.903977008811869	0.981957835819038
Micb	19.2707193081356	0.0785502302375511	0.659617014528374	0.119084602894476	0.905208329045411	0.981957835819038
Ndrg3	1201.56731066371	0.0182541591469671	0.151609417082548	0.120402541598245	0.904164278146031	0.981957835819038
Ovca2	307.093224789736	-0.0165327681197411	0.136718298220048	-0.120925789268761	0.903749815179747	0.981957835819038
P2ry2	104.425289033924	-0.042500510859418	0.355398193610412	-0.119585613049027	0.904811417053807	0.981957835819038
RGD1310313	7013.26716767548	-0.0137149147657533	0.114775501653493	-0.119493398575234	0.904884469739386	0.981957835819038
Rpl15	4309.27621349353	0.0173467926933059	0.145567646921534	0.119166539132534	0.905143415614091	0.981957835819038
Rwdd2b	598.563088614204	0.0212060951643169	0.178138374351456	0.119042824105257	0.905241428252898	0.981957835819038
Sik1	652.572898957769	-0.0650528788890365	0.538122472471376	-0.120888612196914	0.903779262166715	0.981957835819038
Tmem218	264.4107989589	-0.0206444250175219	0.171664688060567	-0.120260172611842	0.904277052731991	0.981957835819038
Tusc3	1030.84493696726	0.0176856414567667	0.148682100646262	0.118949364986735	0.905315471749865	0.981957835819038
Ubqln4	1977.41045160461	-0.0124679796180018	0.103681652269166	-0.120252516671261	0.904283117278369	0.981957835819038
Cym	18.2302168503463	-0.0595951111662692	0.501900110523907	-0.118738987931405	0.905482147131583	0.982033404990227
Actr5	152.349563743952	-0.020989201940174	0.17957675800826	-0.116881506120122	0.906953953669994	0.982358238833483
Arih1	1241.52890276134	-0.0143807441217927	0.12217821521945	-0.11770301355248	0.906302978912002	0.982358238833483
Cox7a2l	2390.81824541686	0.0140055889760938	0.11966109479255	0.117043797738727	0.906825346399348	0.982358238833483
Gabrp	88.1344469993072	0.0613188175431007	0.527510815852501	0.116241820452543	0.907460893455947	0.982358238833483
Gas2	337.806252567207	-0.0391186716146718	0.335822882522808	-0.11648602180054	0.907267363667603	0.982358238833483
Il17re	61.2137376342826	-0.0463334173525253	0.399200686531648	-0.116065475125008	0.907600650703572	0.982358238833483
Mrpl1	231.194163107842	0.0196614375937605	0.168689925024921	0.116553715883482	0.90721371701992	0.982358238833483
Myeov2	1388.00448135555	0.01984288747265	0.168332336177921	0.117879237722197	0.90616334443251	0.982358238833483
Ndufab1	2497.23102536016	0.0185268257954779	0.159789874357937	0.115944929989599	0.907696186813815	0.982358238833483
Nek11	45.6908947066862	-0.0385914001444516	0.329463548493166	-0.11713405115969	0.906753826534524	0.982358238833483
Pms2	361.478542110256	0.0204394496135225	0.175213743571582	0.116654374233903	0.907133947417577	0.982358238833483
Prdx2	3762.38375371761	0.0137977023675566	0.117371393853614	0.117555921545629	0.906419532201451	0.982358238833483
Prkab2	33.8185252343905	-0.0414630376314462	0.355487916205244	-0.116636981853153	0.907147730442578	0.982358238833483
Rnaseh2a	1122.81650018841	-0.0126534275150523	0.108215192679284	-0.116928383175854	0.906916805905168	0.982358238833483
Rpl8	3969.34178964123	-0.0160082039618881	0.137061740630931	-0.116795568830501	0.9070220552807	0.982358238833483
Sh3d21	88.8518316656562	0.0330728058069346	0.283116634242019	0.116816893841224	0.907005156061905	0.982358238833483
Tm2d1	790.337360905703	0.0153460928320856	0.130338586316567	0.117740212363612	0.906273503504839	0.982358238833483

Tmem209	300.869258441951	0.0165903623448948	0.143202606544877	0.115852376888794	0.907769539193038	0.982358238833483
Tmem38a	107.710705806002	0.0389571311114968	0.329683144366871	0.11816537113631	0.905936627511157	0.982358238833483
Trim32	330.226709907058	-0.0232785013994274	0.200156486992533	-0.116301508630574	0.907413590013347	0.982358238833483
Trub1	152.231778880522	0.0349420407470563	0.301258880297971	0.115986757676639	0.907663036801158	0.982358238833483
Utp11l	554.034632393859	-0.0188964620772731	0.16026068400053	-0.117910778898277	0.906138352504324	0.982358238833483
Whsc2	462.415842512325	0.0133965494543756	0.115378302069708	0.116109781597252	0.907565536656734	0.982358238833483
Efnb2	38.2874511898491	-0.0746257502899937	0.646747522685047	-0.115386217453414	0.908139002872498	0.982462301679507
Mbtps1	1792.8339024522	0.0176213157000898	0.15283703050865	0.115294805463343	0.908211455546945	0.982462301679507
Tmem214	1163.0265986202	-0.0125613591785067	0.108873350409832	-0.115375885202595	0.908147192123203	0.982462301679507
Tmem41b	712.015147075048	0.012613903637738	0.109176075934869	0.115537250535208	0.908019296501249	0.982462301679507
Dpm1	1145.16265895385	-0.0190369949302157	0.165484710654434	-0.115037787206631	0.908415170988737	0.982589154307002
Psmg4	366.959258134559	0.0228061874581519	0.199043189826958	0.114579089482935	0.908778754709537	0.982795368794348
RGD1311345	2075.15775572613	0.0146467517032945	0.127788610708959	0.114617035290044	0.908748676490119	0.982795368794348
Zmynd15	140.492726364095	-0.0357592782847009	0.313467924213595	-0.114076355258393	0.909177265618428	0.983132794312534
Amotl2	2388.33321493076	-0.0303197996714109	0.270455064870485	-0.112106607010412	0.910738878815895	0.983151664505522
Anp32b	2530.64051053622	-0.014007678325382	0.123355717177035	-0.113555161008701	0.909590433678436	0.983151664505522
Bicd2	455.981407390541	-0.0136813319269003	0.122035657147231	-0.11210929859946	0.910736744691817	0.983151664505522
Ebna1bp2	388.649663107396	-0.0152586643623311	0.136036648129095	-0.112165835987454	0.910691917120382	0.983151664505522
Emp2	162.210716882934	-0.0542847731414201	0.479790814115069	-0.113142585360963	0.90991751349689	0.983151664505522
Epha3	45.9667730713389	-0.0679424407391157	0.597306922069618	-0.113747954742766	0.909437596801394	0.983151664505522
Ero1lb	40.5290805816098	0.0475220297243441	0.421243547447446	0.112813668036714	0.910178281996606	0.983151664505522
Klc3	64.6604017301715	-0.039565260895388	0.348575587995817	-0.113505541575283	0.90962976993438	0.983151664505522
Lmln	51.6674437785827	-0.0390871399622637	0.348350480959977	-0.112206361405181	0.91065978535353	0.983151664505522
LOC362863	39.6761965657517	-0.0548001879833082	0.483316276689699	-0.113383700542101	0.90972636145838	0.983151664505522
LOC690349	536.638889898845	-0.0206279155854157	0.184029229181548	-0.112090430836212	0.910751704696343	0.983151664505522
Pkm	5372.3502611077	0.0184616700300515	0.163864221642922	0.112664435500029	0.910296598035251	0.983151664505522
Plxnb2	4540.16957059176	-0.0163133097039987	0.144629411083885	-0.112793861094664	0.91019398542076	0.983151664505522
Rapgef6	158.606391037834	-0.0314274610179928	0.277649344564849	-0.113191194696491	0.9098789764176	0.983151664505522
RGD1565641	486.263519580426	0.0178921404742528	0.158930903960438	0.11257810802301	0.910365041961053	0.983151664505522
Snrnp27	540.771127123482	-0.0135539865837743	0.119581830517671	-0.113344866231759	0.909757148290191	0.983151664505522
Tspan14	440.838348894965	0.0258246133849107	0.228418109476633	0.113058519939955	0.909984160367758	0.983151664505522
Ubxn11	119.019090393805	-0.0271035162581808	0.239060301146652	-0.113375228459844	0.909733077893417	0.983151664505522
Acvr1b	253.868961618031	0.0199306712647449	0.183751697327198	0.108465236265303	0.913626655228064	0.983202435797579
Aldoa	5712.38606350844	0.0220730560325219	0.20344476549575	0.108496554230504	0.913601813706842	0.983202435797579
Alox15	24.3126855496535	-0.0644256994380507	0.59086488463419	-0.109036263811713	0.913173727432085	0.983202435797579
Cct5	4688.0451231318	-0.0143186670379789	0.131855834812766	-0.108593351657977	0.913525034177332	0.983202435797579
Cdc42bpg	2424.79644966993	0.0129479073085373	0.118519950615687	0.109246647853593	0.913006862057308	0.983202435797579
Cep70	257.450043513857	-0.019206612582295	0.174170596226243	-0.110274713404243	0.912191510719611	0.983202435797579
Chmp3	1633.60022819768	-0.0105224411860153	0.0968248352770006	-0.108675022848345	0.913460253377124	0.983202435797579
Ckmt1b	1173.94722401911	0.024756314674705	0.223972439836435	0.110532861510926	0.911986789812342	0.983202435797579
Ctdnep1	1217.52371436386	-0.0115311317143346	0.1060217272219	-0.108761968103012	0.91339128987187	0.983202435797579
Eif3h	3519.7115756867	-0.0152948398021078	0.141197243016416	-0.108322510237183	0.913739867088671	0.983202435797579
Fjx1	772.638317671436	-0.0244495556474632	0.224499221999	-0.108907084085895	0.913276187781824	0.983202435797579
Haus3	97.26268902807 6	0.0269745278183177	0.242477475339165	0.111245499321482	0.911421672279042	0.983202435797579
Igf2bp2	79.635146082357	-0.0316140250847332	0.291632357634585	-0.108403694779115	0.913675470399279	0.983202435797579
Inmt	3056.3713328767	-0.0954530441210028	0.854356867525725	-0.111725027034009	0.911041434860513	0.983202435797579
Itpkb	381.252481361233	-0.0319496254515981	0.291432988655996	-0.109629406056399	0.912703288554215	0.983202435797579
Lcmt1	488.687200271018	-0.015869893828907	0.143891349921949	-0.11029081204336	0.912178743738402	0.983202435797579
Mre11a	106.271975970869	0.0248703729586384	0.227547916731228	0.109297300172669	0.912966687918272	0.983202435797579
Pgm2	372.770074626929	0.0140849145095323	0.127556308867895	0.110421151525476	0.912075379207741	0.983202435797579
Prdx5	6102.64819840779	0.0147740961055756	0.132445656983846	0.111548362113357	0.911181517440394	0.983202435797579
Prrg2	182.884433828503	0.0202383821725904	0.18416982624987	0.109889782624501	0.912496785752006	0.983202435797579
Psen2	135.244239774956	0.0246294445869576	0.22676850695289	0.108610516151056	0.913511419419331	0.983202435797579
Rad9b	106.367748561574	0.0282556914839348	0.258066130005979	0.109490119773874	0.912813757934088	0.983202435797579
Reep4	310.907415428166	0.0171480876043028	0.153872285525366	0.111443640066528	0.911264555797506	0.983202435797579
Rnf11	724.499321240932	0.0187514050980679	0.172249715416294	0.108861747914935	0.913312147016584	0.983202435797579
Rwdd1	541.587497941928	0.0120823692188805	0.111535524519903	0.10832754201756	0.913735875795167	0.983202435797579
Sel1l	672.319128477315	0.0216853887533645	0.196733521177998	0.110227218135054	0.912229176843487	0.983202435797579
Slc38a6	135.795288699822	0.0218661850610194	0.200966655500732	0.108805040351282	0.913357125934668	0.983202435797579
Spin1	298.375671843177	0.0257011152111638	0.234483775930781	0.109607221690045	0.912720883089919	0.983202435797579
Taf2	462.983093582509	0.015777942146069	0.142525024199156	0.110702960653575	0.911851898151476	0.983202435797579
Tatdn3	162.728459674022	-0.0208492595309883	0.188761655530988	-0.110452832554044	0.912050255083328	0.983202435797579
Tef	3348.92305910115	-0.0573878568043014	0.517835402273298	-0.110822582914124	0.911757037081478	0.983202435797579
Tm7sf2	519.18908673237	-0.0229230605309878	0.207370033876478	-0.110541817940012	0.91197968714068	0.983202435797579
Uqcc	821.113083026031	0.00984123325927873	0.0896771337496082	0.109740720379812	0.912615005234749	0.983202435797579
Zfp61	439.712637865061	0.0267757828905499	0.243778397885091	0.10983656928934	0.912538988389308	0.983202435797579
Abca2	2814.42621698925	0.0185088592070581	0.171438461128596	0.107962117048954	0.914025742727498	0.983416943566901
Armc5	746.608502380146	-0.0142968152608643	0.132975620320905	-0.107514559634032	0.914380775355571	0.983426560625608
Ccdc148	136.56709081539	-0.0255890218774744	0.237470731147917	-0.107756529631163	0.914188826361783	0.983426560625608
Men1	674.086667895318	0.0120967005833909	0.112378471393954	0.107642508688205	0.914279275817588	0.983426560625608
RGD1311188	263.36890223625	-0.0188237801915707	0.175031897240435	-0.107544856042514	0.914356741667835	0.983426560625608
Rsbn1l	192.43867806236	0.021239755455781	0.197843338040959	0.107356434975757	0.914506214552137	0.98346841088084
Cox5a	6822.25468561729	0.0172070989238716	0.161169280324278	0.106764135753726	0.914976100325781	0.983694509676771
Fnbp1l	168.819788045156	0.0334503761599401	0.31301132358326	0.106866345207612	0.91489501288596	0.983694509676771
Tiprl	759.231128740361	-0.0170372732713757	0.159532939458927	-0.106794705401652	0.914951847931547	0.983694509676771
Gpr155	29.0074436226467	0.0426620579241855	0.400540833677169	0.106511132791446	0.915176822964747	0.983817248634949
Ube2d3	5608.65679892452	-0.0179839775045594	0.169246360977316	-0.106259168000485	0.915376727335102	0.983939084820386
Fam18b2	1627.60944826506	-0.0140511477069845	0.132586808006108	-0.1059769664742	0.915600627345468	0.984086688454712
B9d2	170.115798527535	-0.0188361131147878	0.178383429718888	-0.105593401497389	0.915904960324042	0.984320705280168
Llph	538.569677193757	-0.0168020914462819	0.160133752704986	-0.104925358723319	0.916435036680491	0.984614110125377
Mageh1	420.165489329121	-0.0157228437494745	0.149779509921487	-0.104973262081818	0.916397025243604	0.984614110125377
Mrpl47	562.375491964894	0.0160407316459247	0.152882725665001	0.104921805757659	0.916437855975397	0.984614110125377
Nat2	80.2867183161431	-0.0378696718431143	0.361406902103866	-0.104784030472752	0.916547182120007	0.98463849451569
Yipf4	1693.82475950377	-0.0156580863605377	0.149591424718632	-0.104672352643135	0.916635800816318	0.984640630571616
Chrna1	148.382877946394	0.0483337617332492	0.463036992227202	0.104384233969654	0.916864433798015	0.984655878124142
LOC500077	86.4679734463029	0.0260110636053928	0.249142088405762	0.10440252697501	0.916849917407024	0.984655878124142
Msh2	444.597794107218	-0.0155728355090121	0.14926953466956	-0.104326951534256	0.916909890380602	0.984655878124142
Slc22a22	6508.99347456344	0.0276333760603879	0.265643743909033	0.104024192904956	0.917150149559741	0.984820840802647
LOC361016	20.9163253201672	-0.0463624753592854	0.447609685671695	-0.103577909154742	0.917504319293109	0.985108076051906
Aes	6013.70238387834	0.0177731574565188	0.172173984370521	0.103227891957653	0.917782103575596	0.985119944061265
Atp12a	39.5899331906447	0.0775537125568708	0.75886067019567	0.102197564853208	0.918599860602406	0.985119944061265
Car8	157.886736472358	-0.0280838057011783	0.279003564764209	-0.100657515702039	0.919822337578642	0.985119944061265
Cd36	571.509729971383	0.0548610350761313	0.53609962766302	0.102333656367722	0.918491841608699	0.985119944061265
Cd63	11636.3196298769	-0.0142402849201686	0.140937195609171	-0.101039933841581	0.919518759746932	0.985119944061265
Cox4nb	543.101570620944	-0.0122974228001757	0.121910379495014	-0.100872648015001	0.91965155605629	0.985119944061265
Crim1	520.420627443722	-0.0316632901984575	0.313195401220756	-0.101097557866565	0.919473016655074	0.985119944061265
Dnajc25	99.9931824971891	-0.022843379236635	0.221460957704141	-0.103148561595008	0.917845063958178	0.985119944061265
Foxk2	802.032653656493	0.0113094141097722	0.112144896203942	0.100846445024171	0.919672356948518	0.985119944061265
Ggh	6536.93501945751	-0.0247083433926194	0.245571444136364	-0.100615702609539	0.919855531085888	0.985119944061265
Gltpd1	317.407098322209	0.0138938538906687	0.136847738749051	0.101527829525536	0.919131466927683	0.985119944061265
Gtf2i	4107.17268399646	-0.0104674172222237	0.102833858082408	-0.101789599431691	0.918923681220535	0.985119944061265
Iyd	4073.36813156275	-0.0175316610211921	0.173339964596304	-0.101140328844661	0.919439064378187	0.985119944061265
Nkiras1	105.007873679476	0.0278506995025569	0.272864831917665	0.102067750200072	0.918702898905602	0.985119944061265
Pi4kb	897.176185172296	0.0123351741363388	0.122513281681782	0.100684382680878	0.919801009183132	0.985119944061265
RGD1561328	458.547770292978	0.0204330751648923	0.20240820673851	0.100949835454496	0.919590282164053	0.985119944061265

RGD1564036	70.3786406368058	0.031586344672658	0.307053168199603	0.102869300642177	0.918066702947433	0.985119944061265
Sdr39u1	920.248917446171	0.0102198953334778	0.0997749508233041	0.102429470013738	0.918415792996324	0.985119944061265
Sft2d2	1754.62924248869	0.0171476641022227	0.16784312274942	0.10216482999916	0.91862584324005	0.985119944061265
Snf8	1963.45816518955	-0.0130113937636553	0.127305906240315	-0.102205735365441	0.918593375434801	0.985119944061265
Tmem18	141.819359375718	0.0254346221368617	0.249660519411271	0.101876829371498	0.918854441737523	0.985119944061265
Tmem205	1173.25480304313	-0.0163831944717738	0.160935817518035	-0.10179955416039	0.918915779541572	0.985119944061265
Tmem230	759.398150421824	0.0119259300685081	0.118370080684858	0.100751220236632	0.919747950329474	0.985119944061265
Ttc9c	547.863631238439	-0.0178088188504314	0.174130353433563	-0.102272915084998	0.918540053188823	0.985119944061265
Uqcrh	4259.70628052287	0.0148253910853435	0.14363724085868	0.10321411770872	0.917793035443549	0.985119944061265
Zdhhc21	76.6123574886093	-0.0311347489939419	0.306892596704788	-0.101451613131912	0.919191966430332	0.985119944061265
Zfand2b	346.667432937416	0.0143665394561583	0.140240904367218	0.102441862600513	0.918405956883767	0.985119944061265
Fam195b	744.486548023817	0.0183260728122609	0.182592731508443	0.10036583965235	0.920053888801179	0.98523680014011
Mrps2	814.757268540702	0.0166278418626642	0.166391036987242	0.099932317051063	0.920398059465747	0.98523680014011
Sec14l2	113.759208995888	0.0277022113546497	0.276531851509688	0.100177289536136	0.920203575628336	0.98523680014011
Uck1	774.37945032285	0.0116118056887032	0.116058829675073	0.100051032060314	0.920303810936647	0.98523680014011
Zfp35	127.311607526835	0.0225787067039021	0.225730453919225	0.100025079965425	0.920324414358581	0.98523680014011
Angel2	563.512172848998	-0.0129979083094838	0.130609997154301	-0.0995169481102452	0.920727832050036	0.985411253760206
Bbs5	280.492259071187	-0.0260023887378133	0.263760322956823	-0.0985833974053403	0.921469052749904	0.985411253760206
Car5b	4715.05276092583	0.0181482361397513	0.184355119251641	0.0984417260199832	0.921581542991017	0.985411253760206
Elmod2	559.740390781756	-0.0115797686266185	0.117085739742086	-0.0988999057624457	0.921217743731142	0.985411253760206
Gli4	183.695556653367	-0.0187663053125702	0.189895959771	-0.0988241420997101	0.921277899695254	0.985411253760206
Idh2	9740.24568044015	-0.0141970631803676	0.144634567242659	-0.0981581612959	0.921806704419602	0.985411253760206
Imp3	783.260146285048	-0.0121048470580043	0.123156216161877	-0.0982885593212259	0.921703162532352	0.985411253760206
LOC501110	352.436351611458	-0.027486362521908	0.280218179855559	-0.098089148020575	0.921861504595484	0.985411253760206
Mcam	289.781461632976	0.0642039687872504	0.651294541886716	0.0985790063605629	0.921472539313854	0.985411253760206
Pigz	1219.3905182701	0.0419908769598851	0.425742491272838	0.0986297534792579	0.921432245333892	0.985411253760206
Polr3k	650.698725272955	0.0245200278835581	0.247999540241095	0.0988712634697658	0.921240485513348	0.985411253760206
Ppm1g	1547.74224585558	0.00906649157019715	0.091243370022912	0.0993660313940671	0.92084765227302	0.985411253760206
Psd2	20.229552161729	0.053985597712935	0.549628580587258	0.0982219622845183	0.921756043404147	0.985411253760206
RGD1302996	934.058534503647	-0.0115809903354723	0.117506904025819	-0.098555828965826	0.921490942582972	0.985411253760206
Tmem57	770.874844454769	0.0106771545720801	0.107329423175908	0.0994802194602383	0.920756992570859	0.985411253760206
Ankle2	226.714586547481	0.0272033773465786	0.277654418245311	0.0979756688854276	0.921951613812679	0.985414899623369
Arf4	5712.82716891497	-0.0157765125318398	0.161229049656243	-0.0978515507315644	0.922050172193854	0.985427574720766
Rasl11a	23.2290412422279	0.0680145390715608	0.699140033497935	0.097283141878274	0.922501543326852	0.985817275428075
Acot4	390.892275210756	-0.0287448794432619	0.296058237737924	-0.0970919764398092	0.92265335263144	0.985886810755754
Car13	23.7096587467169	0.0550926696643003	0.568202030757722	0.0969596493536496	0.922758438564496	0.985906412175602
Sept6	28.477414478821	-0.052569776035915	0.544330102019275	-0.0965770142803043	0.923062311096285	0.985957901872377
Dnajc28	98.4204076207237	0.0254957922594517	0.264372618243824	0.096438853724018	0.92317203510663	0.985957901872377
Nup160	219.202866616509	0.0166795606356091	0.173109210139262	0.0963528204085205	0.923240361572031	0.985957901872377
Polr1a	529.808194928327	-0.0119660182903965	0.124023049124246	-0.0964822133860694	0.923137599679493	0.985957901872377
RT1-CE11	433.817686034569	-0.0149554047098314	0.155126437960152	-0.0964078393502019	0.923196666226651	0.985957901872377
Phc3	74.446895543201	-0.0309208160334708	0.322342616583847	-0.0959253118969075	0.923579891290166	0.986227832055997
Araf	1079.33903038129	0.0113447303250957	0.118738880651443	0.0955435175306905	0.923883126343271	0.986241561021436
Mis18bp1	39.3165355775883	0.0402039353157882	0.421594458246055	0.0953616313721183	0.924027590877277	0.986241561021436
Rabep1	624.344995809634	0.0124965262151702	0.130699323151388	0.095612784472461	0.923828111181416	0.986241561021436
Rpp21	158.643582219348	-0.0215272352704435	0.225999113639528	-0.0952536269889087	0.924113375407205	0.986241561021436
Sdcbp	4243.58270111459	-0.0087584223639622	0.0916432157194352	-0.0955708755438703	0.923861397239907	0.986241561021436
Syt5	31.0589975772538	0.0381296997564948	0.400126215113573	0.0952941804767076	0.924081164926797	0.986241561021436
Spsb1	151.888800289953	-0.0405224799417858	0.42638353238189	-0.0950376289520765	0.924284938591289	0.986332045068875
Clns1a	697.371949542301	0.00932609790515963	0.0984668493049769	0.0947130731915097	0.924542733755805	0.986355156712322
Narg2	169.651066755792	0.0203392459409763	0.214893377646794	0.0946480815914514	0.924594357641838	0.986355156712322
Prkar1a	1772.49617940731	-0.0089328537554001	0.0942673393270403	-0.0947608558719313	0.9245047793975	0.986355156712322
Zbtb38	189.429180104162	-0.03247629365218	0.343397952818933	-0.0945733467121282	0.924653721165739	0.986355156712322
MGC116197	56.0833336033528	-0.0413575111806958	0.43791973177	-0.094440848813858	0.92475896813053	0.986374852831419
Nfx1	481.261253321694	-0.0113042270759186	0.119867114727851	-0.0943063249798243	0.924865825702824	0.986396263013822
Atp5j2	6159.36551552143	0.0138358360006003	0.148617367810076	0.0930970330350734	0.925826474495903	0.986467063858546
Cd2ap	570.784560514827	0.0168996250233624	0.182283814929207	0.0927105076768645	0.926133549048869	0.986467063858546
Cpsf3l	425.546092380992	0.0139635395660275	0.150094806998685	0.0930314635479016	0.925878565310151	0.986467063858546
Ints2	308.557031187559	-0.0207100615008895	0.222189277241934	-0.0932090952271249	0.925737448901717	0.986467063858546
Mrps35	994.594286289546	0.0106752002667587	0.114963662250771	0.0928571694547518	0.926017032497911	0.986467063858546
Nutf2	1116.73336190481	0.0110281493243517	0.118695251569243	0.0929114617354195	0.925973899990064	0.986467063858546
Padi4	121.008336092087	-0.0309154483617719	0.332979515733852	-0.0928448955595285	0.926026783525349	0.986467063858546
Paqr4	34.106508407997	0.0475584693682362	0.507566599505123	0.0936989735230917	0.925348285701353	0.986467063858546
Pex5	908.415694738023	0.0112007917756519	0.11935695310202	0.0938428091916697	0.925234024894598	0.986467063858546
Ppia	19864.2237674853	0.0153760463812504	0.164042608247369	0.0937320281939434	0.925322027451155	0.986467063858546
Rab4a	482.79463369374	-0.0119786083462986	0.127537948575163	-0.093921914850615	0.925171185252871	0.986467063858546
RGD1311863	97.2215438895028	0.0261314132822358	0.279567383781316	0.0934708939533392	0.925529471702364	0.986467063858546
Thumpd2	54.1103258725144	-0.0264459564473999	0.284892701653982	-0.0928277779453964	0.926040382676211	0.986467063858546
Wbscr22	506.34049064854	-0.0166584825866966	0.179716303670924	-0.0926932184027094	0.926147284747013	0.986467063858546
Rab7a	7329.44939506459	0.0097375992582637	0.105204658914224	0.0925586315165279	0.926254209874884	0.986488507256179
Ermp1	2101.29885879378	0.01492883283221	0.161714575330848	0.0923159387560917	0.92644702514909	0.986501966829081
Fkbp3	1442.11722088358	0.01599274936755	0.173428958687598	0.0922149881344682	0.926527229975404	0.986501966829081
mrpl24	761.109852092343	-0.01366650741214	0.148025849069025	-0.0923251411702239	0.926439713908567	0.986501966829081
Rapsn	187.135493856109	-0.0207962234761597	0.226513578195542	-0.0918100523678408	0.926848957162777	0.986600523600487
RGD1304567	806.472346508902	-0.0118373993333239	0.129331890316771	-0.0915273047067564	0.927073611269003	0.986600523600487
RGD1564419	46.1072408953759	0.045344797994997	0.494878622163286	0.0916281204404814	0.926993508546667	0.986600523600487
Sirt7	659.542092288046	-0.011125743816826	0.121538540867353	-0.0915408703891603	0.92706283266936	0.986600523600487
Tmem248	1615.25502442273	-0.0123283379991744	0.134819950116815	-0.0914429799780553	0.927140611699525	0.986600523600487
Trabd	1971.09218579454	0.00960428208875214	0.104483700662667	0.0919213430213413	0.92676053397215	0.986600523600487
Dnm1	651.792930372523	0.0421009286884887	0.461598074203541	0.0912068984714273	0.92732819361551	0.986641144814194
Slc9a3r2	897.118420975021	0.0429720204738964	0.471871604562065	0.0910671887404157	0.927439203870742	0.986641144814194
Swap70	556.942225432779	0.0135135509016831	0.148299606940523	0.0911233089586198	0.927394611819563	0.986641144814194
Amer1	23.1499763378392	-0.0409036965360444	0.450281777917305	-0.0908402217056993	0.927619549855303	0.986716757242516
Msmo1	757.615788199506	-0.0117283863274331	0.129346793639699	-0.0906739625885357	0.927751660229247	0.986716757242516
Ribc1	200.44690031804	-0.0162008526021069	0.178718769538043	-0.0906499784213115	0.927770718339853	0.986716757242516
Ahcyl1	3402.9986021611	-0.00779435136478583	0.0862732004241715	-0.090344989248852	0.928013070050795	0.986747134967764
Cilp2	19.2021883339525	0.0514299130430032	0.569631589564731	0.0902862727158477	0.928059728383371	0.986747134967764
Vim	1433.59034742329	0.0420209658851325	0.465407150738056	0.0902886124944459	0.928057869103875	0.986747134967764
Sdr42e1	30.0658035580634	-0.0487514766392789	0.540767867563564	-0.0901523177753317	0.928166174867732	0.986768005195645
Ankrd1	40.4417390840716	0.053644256982287	0.596654842321754	0.0899083576922663	0.92836003957527	0.986826620348005
Zscan21	356.141344662289	-0.0174731517905857	0.1944390397012	-0.0898644213499368	0.928394954371038	0.986826620348005
Akr1c13	303.905094428549	-0.0239044061036088	0.269896416606594	-0.08856881615606	0.929424591761955	0.986971574229849
B9d1	189.348668594175	0.0168826360376267	0.189067311257662	0.0892943149470136	0.928848012038003	0.986971574229849
Dazap2	6155.29602881246	0.0133512316675195	0.149908915883727	0.0890622921846425	0.929032404741369	0.986971574229849
Dpp7	755.079424386036	-0.0183688052189201	0.205595088727707	-0.0893445720546758	0.928808072304773	0.986971574229849
Eno3	129.147498816802	0.0230413626684341	0.260279845233968	0.0885253433577309	0.9294591423224	0.986971574229849
Gss	23383.3783012622	0.0133670879109048	0.15074340708477	0.0886744446699935	0.929340642708921	0.986971574229849
Plekha1	975.999068105675	-0.0121274899300648	0.136657841134643	-0.0887434619877838	0.929285791103065	0.986971574229849
RGD1359508	163.996340123744	0.0249699906497914	0.280445140071133	0.0890369882803388	0.929052514444316	0.986971574229849
Smg6	549.860214068889	0.0122667940632433	0.137960759289629	0.0889150953242504	0.929149386735877	0.986971574229849
Tprkb	381.003344127643	-0.0142572477646872	0.161115407755898	-0.0884909020389161	0.929486515093816	0.986971574229849
Wdr26	2371.21929732517	0.0103960799272444	0.117199403196002	0.0887042053435904	0.929316990332492	0.986971574229849
Fez2	189.264249494821	0.0185628545905733	0.210758443075628	0.0880764458101082	0.929815917132145	0.987136905858767
Rab6a	1900.04037568427	0.0101732718926339	0.115465602136388	0.088106515744985	0.929792017706571	0.987136905858767
Arhgap35	2041.16832300555	-0.0115412000963326	0.135291416391549	-0.0853062256583305	0.932017942217636	0.987187810523909

Atp6v0e1	2974.60561964361	-0.0107466078709987	0.122277137191222	-0.087887303529136	0.929966247869413	0.987187810523909
Baiap2l1	328.227990319652	0.0151883014446522	0.178781663113092	0.084954470051241	0.932297587427084	0.987187810523909
Cenpq	17.4726247032607	-0.0471234513294742	0.555219048901811	-0.0848736213620219	0.932361863202872	0.987187810523909
Dctd	50.9383500958929	0.0369962140352411	0.428630087545696	0.0863126857171381	0.931217854366821	0.987187810523909
Ddx23	1380.92700354459	0.00912920575624043	0.108670196874053	0.0840083667725477	0.933049779563963	0.987187810523909
Dpp6	17.0487149789218	0.0613006020261422	0.7187472307095	0.0852881227321465	0.932032333813796	0.987187810523909
Ebf1	38.8960009258498	-0.0357106035042997	0.414198993184216	-0.0862160557894387	0.931294667557568	0.987187810523909
Emc3	1848.37433466987	-0.00768033256417011	0.0904354736790365	-0.0849261053403478	0.932320137696637	0.987187810523909
Esrrg	253.178273037935	-0.0207658893999214	0.239981145118394	-0.0865313372418348	0.931044045971804	0.987187810523909
Fam149b1	1034.36871430368	-0.00775989655763984	0.0916736187849315	-0.0846469972549544	0.932542034710814	0.987187810523909
Gdpd2	17.7696589623104	0.0455184222046211	0.536794755051819	0.0847966970173301	0.932423019482396	0.987187810523909
Grid2ip	68.8314489832739	-0.0336746952155148	0.391319791903107	-0.0860541580372018	0.931423364968187	0.987187810523909
Ihh	175.296238589339	0.0341892900368028	0.404754518512115	0.084469199164183	0.932683390804668	0.987187810523909
Mrpl46	648.934566817038	0.0113375688950285	0.130497452051803	0.0868796188490164	0.930767200055952	0.987187810523909
Mrps17	775.839223495393	0.00931025281349864	0.108381944627073	0.0859022491756715	0.931544123538594	0.987187810523909
Ndufc2	1948.68497920621	-0.0131650494288321	0.157390425289444	-0.0836458088515956	0.933338044333093	0.987187810523909
Nup205	298.462687848722	-0.013352296682532	0.158047892104602	-0.0844825989434577	0.932672737407828	0.987187810523909
Ppp2r2a	1108.66315092119	0.0100025858571267	0.119423354401389	0.0837573681233853	0.933249344172121	0.987187810523909
RGD1305938	638.305374357341	0.0100726600470518	0.120207196447513	0.083794151637584	0.933220097984735	0.987187810523909
Rnf219	59.3319024188907	0.0251686592513014	0.288974236891175	0.087096550620115	0.930594767174597	0.987187810523909
Rpl35	8159.66582775246	0.0153321983140715	0.181340528169545	0.0845492095387338	0.93261977931861	0.987187810523909
RragB	45.8577380818621	-0.0286256057927966	0.339020521518862	-0.0844362036390883	0.932709623707785	0.987187810523909
Ruvbl2	495.3060822463	0.0111657798405198	0.131111242082969	0.0851626425249934	0.932132089593699	0.987187810523909
Slc16a1	89.0397679086778	-0.0376398280615651	0.434270282411637	-0.0866737365783804	0.930930853010256	0.987187810523909
Slc22a17	738.712027014156	-0.0126921140611824	0.144798090237649	-0.0876538774810603	0.930151778895236	0.987187810523909
Smyd4	124.672856306777	-0.0185775386574177	0.218570981555534	-0.0849954487334244	0.932265009002061	0.987187810523909
Sp4	64.3906828443461	-0.0311218075942065	0.357899390849162	-0.0869568610339516	0.930705802071896	0.987187810523909
Surf1	370.190526053056	-0.0150227090221919	0.17115410823691	-0.0877729969612977	0.930057100214725	0.987187810523909
Tacr1	38.9901146230036	0.0323200769110284	0.386170291215973	0.083693846073086	0.933299850102012	0.987187810523909
Tmem60	269.683872710362	0.0179578287737282	0.209364231785092	0.0857731457786041	0.931646754350895	0.987187810523909
Trappc3	1359.34562577925	0.00892835815069975	0.103848879937105	0.0859745252534948	0.931486668129982	0.987187810523909
Tulp4	142.695610734592	-0.0348828338391843	0.398895365060296	-0.0874485814943262	0.930314954803754	0.987187810523909
Txndc9	1147.42229082513	0.00965600454020195	0.114824266431693	0.0840937620615772	0.932981884231421	0.987187810523909
Uap1	598.411468732483	0.0106630580914554	0.12573645674477	0.0848048240543326	0.932416558318523	0.987187810523909
Uba52	7043.13861538073	-0.0136742499294115	0.162712165719066	-0.0840395053988839	0.933025022083376	0.987187810523909
Usp20	3019.3080688635	-0.0101147182294573	0.120494479042964	-0.0839434164103958	0.933101420050671	0.987187810523909
Xylb	6540.26544909002	0.0141036536063869	0.164005272639855	0.0859951230797172	0.931470294084325	0.987187810523909
Zfp26	215.67788257596	0.0173717370930004	0.201051475800755	0.0864044246569774	0.931144929719472	0.987187810523909
Zfp90	35.2506780582148	-0.0302900796219483	0.362038856419284	-0.0836652726216459	0.933322568737366	0.987187810523909
Blzf1	283.099784694986	-0.0152308520639229	0.182382285444453	-0.0835105889083818	0.933445558072801	0.987209659724152
Acp1	1242.528620623	0.0186493619320682	0.236701142716443	0.0787886434262351	0.937200737198908	0.987231843038534
Acvr2a	146.860544439076	-0.0177424540433965	0.219242728754642	-0.0809260774310668	0.935500741186151	0.987231843038534
Ap3m2	55.3387229996742	0.0279234917580062	0.338866802207834	0.0824025592831019	0.934326600959683	0.987231843038534
Aurkc	74.039896795795	-0.0293145265378138	0.354492795166344	-0.0826942802153654	0.934094633023295	0.987231843038534
Cacul1	805.895428091331	-0.00898007591312886	0.112481710185988	-0.0798358764129774	0.93636779012893	0.987231843038534
Cdh16	12773.476068659	0.0149689107642746	0.184978330803837	0.080922509675734	0.935503578537281	0.987231843038534
Cdhr1	10.1406892700578	0.0843928447425357	1.03367836529154	0.0816432340815542	0.934930420205873	0.987231843038534
Chchd5	166.421379963186	0.0186166758869547	0.237121760100247	0.078511039556573	0.937421549033932	0.987231843038534
Cnot6l	513.387926587264	-0.00998605907299901	0.12367326809633	-0.080745493563094	0.935644356277818	0.987231843038534
Cry2	176.125806086914	-0.0266549598886391	0.336752195370883	-0.0791530396981749	0.936910896232865	0.987231843038534
Ddx46	1440.93099663504	-0.00754232999556394	0.0927932001120259	-0.0812810635526996	0.935218433251426	0.987231843038534
Dffa	440.685955617608	0.0114348006645518	0.141302398143482	0.080924321276845	0.935502137813817	0.987231843038534
Dnajc14	904.258610017993	-0.00798811805406048	0.0959075154328397	-0.0832898028690384	0.933621108253825	0.987231843038534
Dync1li2	1444.96497084517	0.00778137851497312	0.0998823535868202	0.0779054381033318	0.937903273580069	0.987231843038534
Foxp2	354.734323111421	-0.020969481459045	0.262749062233831	-0.0798080163665186	0.936389948525708	0.987231843038534
Gnb1	1297.14008262089	0.0113680024063157	0.13754644441705	0.0826484643386865	0.934131064095875	0.987231843038534
Gpr107	738.043431188142	-0.0112433946727077	0.137148656844595	-0.0819796192787196	0.934662920354328	0.987231843038534
Haus1	53.3940682184579	-0.0221621398741677	0.283446069617347	-0.0781882066810334	0.937678343003217	0.987231843038534
Hsd3b6	11.4879708542347	0.063524251711235	0.817002788108167	0.0777527967295317	0.93802469546924	0.987231843038534
Ildr1	445.665227398498	0.0133546338108332	0.168672265817674	0.0791750424771607	0.936893395479358	0.987231843038534
Klf15	1863.29739564587	-0.0123854577656769	0.157170488898672	-0.078802692874881	0.937189562106368	0.987231843038534
Lama5	2534.37338774151	0.0151327135162767	0.187805189512575	0.0805766526236668	0.935778634324678	0.987231843038534
Mblac1	82.1004802311499	-0.0260400375572214	0.334580619496692	-0.0778288879863792	0.937964166849453	0.987231843038534
Mcm6	149.029032648694	-0.0221798836363302	0.283010360927889	-0.0783712778698646	0.93753272018032	0.987231843038534
Mdk	21.1341793222441	-0.0357961565831665	0.443800295305479	-0.0806582531868915	0.935713737725302	0.987231843038534
Mink1	3187.39411744268	-0.00837145218034436	0.105813690724824	-0.0791150192664097	0.936941137312209	0.987231843038534
Naa15	1024.30277208419	0.00794177815203773	0.102227012234562	0.0776876676569118	0.938076504240458	0.987231843038534
Nck1	335.395739609963	0.0123474235499674	0.148803124336335	0.0829782546908012	0.933868830217126	0.987231843038534
Nol3	12.5404053123177	0.0476574862931095	0.585016444792573	0.0814634985346561	0.935073352315106	0.987231843038534
Pdilt	25.6068324397131	0.0366500087453002	0.452671829490838	0.0809637498019787	0.935470781293772	0.987231843038534
Plk4	46.7701085871496	0.034442600564645	0.417141857165837	0.0825680759985497	0.934194986232434	0.987231843038534
Prodh	1343.79303753913	-0.0204557857047098	0.261615992564521	-0.0781901194349384	0.937676821504399	0.987231843038534
RGD1305178	685.25085298072	-0.0118635052417546	0.144222081145739	-0.0822585913856444	0.93444108206898	0.987231843038534
RGD1559786	672.217874082543	0.0110488072162823	0.136250971622557	0.0810915847770223	0.935369118023534	0.987231843038534
Rpl4	14814.396164827	-0.0118155126273237	0.149071205136054	-0.0792608647427245	0.936825133742821	0.987231843038534
Scamp3	2975.30627507857	-0.0121281320546322	0.14906438994595	-0.0813616991893893	0.935154307709285	0.987231843038534
Sdc4	12045.1511456568	0.014936368157113	0.191893745640669	0.0778366595911997	0.937957984759709	0.987231843038534
Serhl2	1153.55823887045	-0.0477733899233906	0.612975945771218	-0.0779368101684387	0.937878318169699	0.987231843038534
Spag1	215.861787921646	0.0132380190139073	0.168580063784664	0.0785265986778655	0.937409172860957	0.987231843038534
Sptan1	6849.18252400922	0.0104871896002651	0.128841331448031	0.0813961597757566	0.935126903034017	0.987231843038534
Sptssa	899.166677798834	-0.00833954876592432	0.105451242704043	-0.0790844048118989	0.936965487815836	0.987231843038534
Ssc5d	27.703443647594	-0.0338738219163217	0.419693879725978	-0.0807107836274339	0.935671960703409	0.987231843038534
Stoml2	1545.62100126531	0.00976895794926489	0.124243131178782	0.0786277507382496	0.937328713969108	0.987231843038534
Strbp	1588.44432547081	-0.00819916381871687	0.104024897720413	-0.0788192442231834	0.937176396990029	0.987231843038534
Tcea3	1431.54497271859	-0.0148295585474129	0.188656907863626	-0.078605966329803	0.93734604178122	0.987231843038534
Thoc7	800.151928157996	-0.0133119411320111	0.166166913828357	-0.0801118635793026	0.936148287304482	0.987231843038534
Thsd7a	37.0205880607416	0.0314268076208878	0.387138749692854	0.0811771171080679	0.935301097348679	0.987231843038534
Tmem33	583.696706185194	0.0150115932215502	0.18564703313624	0.0808609379204768	0.935552545221774	0.987231843038534
Tssk1b	35.4477687517662	0.0297647902216421	0.381507561850509	0.0780188735375714	0.937813039840348	0.987231843038534
Uri1	631.609497423024	-0.0121960992406414	0.155804666749009	-0.0782781382298935	0.937606807258913	0.987231843038534
Vapa	3059.2893086572	0.00970506831500186	0.125086875894558	0.0775866232616019	0.938156883394264	0.987231843038534
Ythdf3	1365.45578736935	-0.0101803091340129	0.124774836986784	-0.0815894404661989	0.934973198585258	0.987231843038534
Zbtb45	162.078902953187	0.0176094803548725	0.221247516738128	0.0795917649810977	0.936561945031508	0.987231843038534
Zdhhc12	293.245220513523	0.0108277406142857	0.136091939511246	0.0795619538759752	0.936585655659551	0.987231843038534
Bmi1	232.411417629268	-0.0179655756261458	0.235457613281529	-0.0763006783928664	0.939179886139735	0.987532276691215
Drap1	1563.59118153228	0.0093068236962792	0.121509296742519	0.0765935113261381	0.938946921002346	0.987532276691215
Ltbr	1601.85825171956	-0.00746616938634065	0.0973444229300556	-0.0766984811415985	0.938863412856716	0.987532276691215
Nt5dc3	38.2550373714013	0.0425814206549458	0.554083468689805	0.0768501914623702	0.938742721753538	0.987532276691215
Ogfod2	325.820156220841	-0.0103669892846504	0.136341861276258	-0.0760367299346501	0.939389876506849	0.987532276691215
Pop4	444.343305396792	0.0106394289159534	0.139387527881395	0.0763298487150622	0.939156679267151	0.987532276691215
Rassf2	220.421404062022	0.0205829110468589	0.270451462859038	0.0761057486222098	0.939334966668763	0.987532276691215
Rb1cc1	940.301916185824	-0.00948795568546586	0.12497773811451	-0.0759171659577692	0.939484999811905	0.987532276691215
Rprd1b	530.300626263132	-0.00945857395608039	0.122922336669541	-0.0769475606496838	0.938665261748667	0.987532276691215
Tpm2	362.176501385349	0.052980618664207	0.687593139094116	0.0770522793959337	0.938581955604815	0.987532276691215
Utp20	282.060104954727	-0.014343404181978	0.188800481774135	-0.0759712265943114	0.939441989874034	0.987532276691215

Zfp507	141.659386221566	-0.0167801657193783	0.220762134136956	-0.076010162634903	0.939411012977005	0.987532276691215
Acot7	256.039061257762	-0.0204071607451573	0.271424763381251	-0.0751853312532608	0.940067255282616	0.987596304976635
Cdk19	658.89079757662	-0.0108404415299614	0.144145327558683	-0.0752049456861389	0.940051649412086	0.987596304976635
Cers5	463.700579044741	0.0117565489315097	0.155288037531987	0.0757080140773112	0.939651399983556	0.987596304976635
Dnajb13	33.986012734729	-0.0258508131251739	0.343111394718554	-0.0753423334902027	0.939942339923936	0.987596304976635
Rpf2	277.868151745688	-0.0176204386182557	0.233465786355825	-0.0754733226366643	0.939838122436105	0.987596304976635
Smek1	722.564906386318	-0.00849819899152007	0.112918837491764	-0.0752593560143578	0.940008358934148	0.987596304976635
Cebpz	1034.55342771108	0.00783115336188077	0.104620469626817	0.0748529746598789	0.940331692318953	0.98760026010878
Dut	596.678850065703	-0.00955160900184572	0.127513344597545	-0.0749067404042481	0.940288913559997	0.98760026010878
Hsdl1	173.994867958802	0.0160201706362259	0.214001608680325	0.0748600477118692	0.940326064629452	0.98760026010878
Dhrs3	1000.89430676349	0.0144053817646469	0.193490550488486	0.0744500531332363	0.940652282635762	0.987634413006055
Gpld1	204.379579951527	-0.0197199142522027	0.264029131110885	-0.0746884033940969	0.940462634652099	0.987634413006055
Ifi35	465.678814832421	0.0132486340039363	0.178668618312831	0.0741519922695061	0.940889445246957	0.987634413006055
LOC684871	603.962783119112	0.0204763301638274	0.276529086970874	0.0740476540393166	0.940972466869837	0.987634413006055
Pif1	23.8490676981138	-0.0307035603178968	0.414466943515566	-0.0740796360198595	0.940947018833771	0.987634413006055
Rest	88.1189892425169	0.0252623750511491	0.338798023415827	0.0745647061232796	0.940561056351452	0.987634413006055
Rnps1	1284.05142995073	0.00809696394115784	0.108901513338576	0.0743512527322212	0.940730896068278	0.987634413006055
Ankrd44	473.201866951845	-0.00940200215661179	0.127314213424805	-0.0738488021383789	0.941130694517929	0.98770927736757
Ccdc167	37.4880102052409	-0.0326601627688601	0.447270241612482	-0.0730210949226464	0.941789330999355	0.988060750130835
Hnmt	11082.6201811865	-0.0152851268211755	0.208685929912984	-0.0732446448476378	0.941611440319394	0.988060750130835
Map3k2	48.8454694479482	-0.0232173321916005	0.317936101669438	-0.0730251521286496	0.941786102436766	0.988060750130835
Zfyve16	454.295306731254	-0.00931734368429093	0.127650666578934	-0.0729909520568738	0.941813317518694	0.988060750130835
Nfatc4	106.453233664128	0.0246968530196327	0.339842548968268	0.0726714565159958	0.9420675628744	0.988236263925283
Aimp1	850.632494571682	-0.0108530997359357	0.150241931675831	-0.0722374879960467	0.942412911981855	0.988269022746238
Bod1	203.833123209043	-0.0142738214229571	0.198379743388405	-0.0719520107202204	0.9426400987052	0.988269022746238
C2cd2	165.459590704067	-0.0242828027642285	0.337883444167688	-0.0718673944621483	0.942707438378912	0.988269022746238
Ctf1	570.25791873143	-0.0103223778041957	0.142831294898293	-0.0722697208027558	0.942387260967126	0.988269022746238
Hjurp	113.005214292905	-0.0204796937251497	0.284951873794983	-0.0718707108411031	0.942704799116263	0.988269022746238
Pigk	1324.98874012705	0.0104453747677449	0.144853091391468	0.0721101266628554	0.94251426729623	0.988269022746238
Wwc2	2338.20253999608	-0.00744523665091013	0.103331980674293	-0.0720516204405088	0.942560827395454	0.988269022746238
Fgfr4	2135.82527067931	0.00731873812727603	0.102093513180157	0.0716866125897848	0.942851310356029	0.988328690722736
RGD1309077	210.602790727436	0.0149247392074521	0.208969118602384	0.0714207884268783	0.943062865084166	0.988368144264327
Tgm4	193.877013607961	0.0132871514175173	0.185925379797776	0.0714649685372126	0.943027704302642	0.988368144264327
Ptpra	961.1460859387	0.00944632649403244	0.13301320960226	0.0710179577072018	0.94338346339274	0.988430719480262
Scgb1c1	51.6080466801246	-0.0356840287471083	0.502100238542406	-0.0710695315554894	0.943342417130895	0.988430719480262
Sco1	327.931819260247	-0.0109741994232454	0.154431507141539	-0.0710619201118551	0.943348474868118	0.988430719480262
Ubr1	195.230084831875	-0.019642644569901	0.277667485270753	-0.0707416086213606	0.94360340487971	0.988570034091878
Ptar1	30.1966505092423	-0.0283926821206647	0.403326964451991	-0.0703961912371577	0.943878322682124	0.988675791687865
Tp53	887.825317366101	0.00781768724755554	0.110948349004573	0.0704624027098712	0.943825624431712	0.988675791687865
Cul4b	708.105630709682	-0.00783509487310907	0.112790075130381	-0.069466173012581	0.944618557612023	0.988907029105378
Etfa	8910.06128889354	0.0126241749272431	0.181942734518792	0.0693854303148295	0.944682825892465	0.988907029105378
Fam76a	480.119242176939	0.0117681003635924	0.169682496979805	0.0693536491568311	0.944708122648794	0.988907029105378
Mettl25	269.258983980385	-0.0130902909893004	0.187636579671601	-0.0697640673913947	0.944381447533092	0.988907029105378
Nop14	391.500353699461	0.0112460091544472	0.161118641214311	0.0697995531099867	0.944353202878573	0.988907029105378
Plscr3	311.572514283348	-0.0134156166557574	0.193437715324739	-0.0693536761082789	0.944708101196305	0.988907029105378
Psmb2	1148.61645907286	0.0106394416640608	0.152625202029775	0.0697095992179928	0.944424801299845	0.988907029105378
Pdzrn3	493.428120206027	0.0144071115510123	0.208877657750047	0.0689739233300508	0.945010376221729	0.988950182868628
Puf60	2240.72315073808	0.00611703456879076	0.0884648988291826	0.0691464597795129	0.944873039943142	0.988950182868628
Tyw5	99.3560020856347	0.0184835286404497	0.267919825835497	0.0689890290231774	0.944998352264127	0.988950182868628
Ube2k	1814.55903297516	-0.00715522037198202	0.104170611725186	-0.0686875141989018	0.945238356956123	0.989097695190876
Krt4	44.2552905308196	0.0289681015015995	0.42270992625967	0.0685295038087286	0.945364134632347	0.98912365132818
Sssca1	361.314484496615	0.010695491562865	0.156280697914789	0.0684377002763103	0.945437211686552	0.98912365132818
Snrk	234.310127347082	0.0174404897410867	0.255582956699011	0.0682380780249975	0.945596115718818	0.989198845030841
RGD1305537	108.891898460096	0.0166797909981583	0.245082654286219	0.068057819296664	0.945739607788916	0.989257903748281
Sf3b4	711.877787920604	0.00835456136015683	0.123203412088582	0.0678111199887062	0.945935991669294	0.989372272137038
Alkbh2	211.551961705641	-0.0174425249303341	0.260710643232282	-0.0669037700727605	0.946658311681639	0.98972274817443
Asb2	44.0005965669748	-0.0308710946888618	0.461373478268846	-0.0669112901866304	0.94665232491413	0.98972274817443
Cox7a2	6023.80354808192	0.00953623691974994	0.145171083956973	0.0656896446579979	0.947624916972268	0.98972274817443
F2rl1	238.857228415989	0.0204908733596914	0.306052213064586	0.066952214311769	0.946619745244482	0.98972274817443
Gnaz	14.7335288222668	0.0409183779651176	0.617442103971608	0.0662707931673529	0.947162235768203	0.98972274817443
Kcnmb4	13.4517698605774	-0.0427481143415956	0.648989121939999	-0.0658687686687418	0.94748230555676	0.98972274817443
Mettl10	169.697020955776	0.012557254377335	0.191387613702532	0.0656116356456188	0.947687025171141	0.98972274817443
Nfkbie	110.666686200571	-0.021674927238258	0.327191105357567	-0.0662454659779666	0.947182399631879	0.98972274817443
Polr2j	1176.63452538018	0.00871517303462422	0.131058877731623	0.0664981509491541	0.946981229781918	0.98972274817443
Prkag1	1282.79451678716	0.00664738541074124	0.099932192474755	0.0665189589673069	0.946964664064336	0.98972274817443
Psmb1	3604.28657755618	-0.0109664624345444	0.167348260161218	-0.065530782477091	0.947751398128672	0.98972274817443
Rps6ka6	16.5861906831567	0.0314732997452076	0.480053164999247	0.0655621127823561	0.947726453804023	0.98972274817443
RT1-A2	6691.6196688026	0.0172537544780979	0.258585215471774	0.066723669590388	0.946801690691109	0.98972274817443
Sec22a	375.59056129318	0.0103226885218869	0.153952403955385	0.0670511681316676	0.94654096854122	0.98972274817443
Vdac3	3910.8756689937	-0.00700863860833414	0.106898228383386	-0.0655636553975238	0.94772522561771	0.98972274817443
Vta1	917.438366852117	-0.00729108295461096	0.109846078889333	-0.0663754503422605	0.947078914880024	0.98972274817443
Zbtb12	158.81838289903	0.0219571311443425	0.332744320398014	0.0659879967840723	0.947387381804208	0.98972274817443
Cyb561d2	356.842208781507	0.0092435465127608	0.14141240553171	0.0653658812888806	0.947882688748676	0.989752574077569
Tgfb3	57.2839325485056	0.036548628150693	0.55990771124934	0.0652761650114459	0.9479541194271	0.989752574077569
Bud13	252.171377935719	-0.0106154091204875	0.163314754335025	-0.0649996943859154	0.948174243586436	0.989891471718879
Agl	997.806406756042	-0.00929722647795353	0.143430901580741	-0.0648202470701189	0.948317120519732	0.989949705347839
RGD1311946	60.5732411588632	-0.0233465265165297	0.363704169251751	-0.0641909785212539	0.948818160678553	0.990059175562274
Rnf25	589.204025282089	-0.00952723225211356	0.148137802463938	-0.0643133089167624	0.948720756411241	0.990059175562274
Tmed4	1791.71109358647	-0.010688531393174	0.166639757640938	-0.0641415442778356	0.948857522382531	0.990059175562274
Uba3	1206.23486126677	0.00984309406069526	0.153003565627478	0.0643324488571755	0.948705516507961	0.990059175562274
Zfp799	152.462261445429	0.0171218598519806	0.266496965188757	0.064247860533246	0.948772868890461	0.990059175562274
Tsr3	482.976618426445	-0.0103433626942763	0.162137604560798	-0.063793730777599	0.949134470207316	0.990257241451841
Adtrp	480.683742709563	-0.0355079916205319	0.558655947952436	-0.0635596770260381	0.94932083985977	0.990269884897774
Eef1g	8068.32109363377	0.00801961772660062	0.126163109377148	0.0635654730308448	0.949316224649632	0.990269884897774
Azin1	2462.52439512335	0.00905414567749058	0.143691720534197	0.0630109072661273	0.949757818839855	0.990341811319434
Drosha	942.955917891933	-0.00900543744109155	0.142243199400658	-0.0633101440282275	0.94951953820331	0.990341811319434
Lsm6	405.478608436884	-0.0119136044585519	0.188514542833385	-0.063197269979757	0.949609418676579	0.990341811319434
Nipa2	664.823546444388	0.00707774770435926	0.112477364877287	0.0629259737021852	0.949825451803022	0.990341811319434
Pot1	184.06060689234	-0.0138527952617611	0.220005256470321	-0.0629657467462822	0.949793780311491	0.990341811319434
Cpxm2	239.91047899579	-0.0185350398524925	0.296618845746634	-0.0624877350791285	0.950174428841641	0.990583123249056
Igf1r	283.355814271284	0.013447844793017	0.215831991770997	0.0623070040853145	0.950318350862898	0.990583123249056
RGD1308106	167.178924700324	0.0137819396476298	0.22082351187858	0.0624115590336585	0.950235090128557	0.990583123249056
Zc2hc1a	60.98271791598	0.020842420406811	0.336781718951731	0.0618870301858581	0.950652796111701	0.990750016560205
Zcrb1	507.474405213986	0.00904891078970753	0.145994150929836	0.0619813241289125	0.950577704588207	0.990750016560205
Retsat	5328.39786535299	0.0417312308833153	0.676211349304303	0.0617132955935286	0.950791151799812	0.990803358518076
Adap1	356.994941032193	0.0124401267667221	0.216008759944791	0.0575908438616177	0.954074543109021	0.990906843924575
Aebp2	172.451327475493	0.0122685031245385	0.203526720019341	0.0602795697949276	0.951932973293948	0.990906843924575
Ankib1	1466.62742139694	0.00835700525900412	0.139543710224714	0.0598880827057447	0.95224477148188	0.990906843924575
Ankrd39	121.390171934086	-0.013689328995039	0.23757426496067	-0.0576212621232577	0.954050313084216	0.990906843924575
Armc6	48.4569407255946	0.0180685978119087	0.300671471167928	0.0600941543995613	0.952080645664159	0.990906843924575
Arpc5	2798.85022992394	0.0081070331975172	0.135459201055707	0.0598485236464906	0.952276278529875	0.990906843924575
Atg9a	1404.82360369844	0.00611354496667845	0.105215940810961	0.0581047407793703	0.953665198179505	0.990906843924575
Bag4	105.039768018622	-0.0153218251417727	0.264957504964804	-0.0578274812174427	0.95388604795539	0.990906843924575
C8g	198.233451960588	-0.0119012572942103	0.204895216548007	-0.0580846029239624	0.953681238772144	0.990906843924575
Ccdc25	805.980599622657	-0.00838563427727482	0.14200309806328	-0.0590524741476975	0.952910312674244	0.990906843924575

Ccnt1	38.118085584403	0.0245701093604697	0.405112727148486	0.060650055438678	0.951637908391468	0.990906843924575
Cct3	3915.8376066176	-0.00787949301428782	0.135311276138691	-0.0582323457374796	0.953563556255917	0.990906843924575
Cep57l1	65.7315926644419	-0.0211198078214572	0.351290034251889	-0.0601207144018024	0.952059492095874	0.990906843924575
Cfl2	1626.2126123514	0.00899939967254466	0.154981102639502	0.0580677225756867	0.953694684646886	0.990906843924575
Chchd1	861.249346630583	0.00900679902765557	0.150659317333088	0.0597825556831823	0.952328819272443	0.990906843924575
Cnnm3	987.279119485066	-0.00841931683249672	0.145372598066126	-0.0579154320999822	0.953815990716945	0.990906843924575
Ctcf	1122.13296547662	0.00758299493420073	0.127829852364276	0.0593210020503779	0.952696433352575	0.990906843924575
Eif4e	1824.11997656504	-0.0103492486511397	0.176026502518058	-0.0587936958531457	0.953116429756774	0.990906843924575
Fam203a	366.29694246019	0.010213232559526	0.168968906187138	0.060444449751095	0.951801657569892	0.990906843924575
Fgf9	214.240660704814	0.0162700594212307	0.273106291306908	0.0595740923556645	0.952494853014472	0.990906843924575
Gpt	586.113074434662	0.0183185745786084	0.307637500042787	0.0595459739988155	0.952517248459254	0.990906843924575
Kcnk5	608.872224359045	-0.0322676095976438	0.541313222281816	-0.059609867761266	0.952466359009645	0.990906843924575
Lss	460.084583614682	-0.0090384545055503	0.151436058100883	-0.0596849562706466	0.952406553530392	0.990906843924575
Oacyl	350.766954992086	0.0166150491225358	0.282730200896776	0.0587664461378214	0.953138134355766	0.990906843924575
Pmvk	482.053399617221	-0.00805945383405892	0.138431359252895	-0.0582198562345646	0.953573504559402	0.990906843924575
Ppp3cb	461.151879804798	-0.00907250560379728	0.155334696229936	-0.0584061759799472	0.953425095451774	0.990906843924575
Qars	2603.33893199832	-0.00510675686515614	0.088550327325355	-0.0576706718021798	0.95401095531268	0.990906843924575
RGD1562890	17.2907404239583	0.0316209989300546	0.534664982654971	0.0591417054714059	0.952839240595575	0.990906843924575
RT1-CE10	1033.74939565558	0.0169171336883986	0.280882651393009	0.0602284747900939	0.951973667271643	0.990906843924575
Sbds	2593.20018302337	-0.00689293458628563	0.114705724590801	-0.0600923328881394	0.95208209639819	0.990906843924575
Serf2	9182.2802242643	0.00948418997412988	0.161859251033939	0.0585952913629953	0.953274461188834	0.990906843924575
Serpinb6	758.868318909063	0.00766418117224699	0.133200750622335	0.0575385734422572	0.954116179826724	0.990906843924575
Sgpp2	59.1185390943387	0.0222949743023606	0.380186766943165	0.0586421628549042	0.953237127346671	0.990906843924575
Tmem55a	580.823836236852	0.0124874517098687	0.204391322736079	0.0610958016353422	0.951282912716342	0.990906843924575
Uchl3	304.934411609872	0.00957149021802227	0.166021436464575	0.0576521346992716	0.954025721213368	0.990906843924575
Uhrf1bp1l	1732.60586155037	-0.00822060186114754	0.13993529024547	-0.0587457377386879	0.953154628771469	0.990906843924575
Vegfb	843.289108733428	0.00977647897385637	0.163040069588079	0.0599636580047879	0.952184579293082	0.990906843924575
Cd276	274.207235027044	0.00930994867480879	0.164665723259695	0.0565384737667964	0.954912846893673	0.991190814410364
Cxadr	111.479578024177	-0.0166225990843075	0.293093750267882	-0.0567142733992613	0.954772803788742	0.991190814410364
Gnl3	848.361474382176	0.0110845479764757	0.195999882382968	0.056553850143733	0.954900597918433	0.991190814410364
Pak1ip1	196.367612082293	0.0149710981601649	0.262673673191966	0.0569950462801949	0.954549141224259	0.991190814410364
RGD1308706	934.50680059749	0.0114894723693907	0.202532485913632	0.056729034443839	0.954761045110264	0.991190814410364
Tmod3	523.062955436061	-0.00891206627417046	0.157264415865003	-0.0566693121590878	0.954808620065808	0.991190814410364
RGD1305587	761.345086344224	-0.00764853754602311	0.135618322629903	-0.0563975235624738	0.955025129728192	0.991216840881255
Samhd1	43.4337543287021	-0.0173457539191168	0.309753982987181	-0.0559984854814109	0.955343013680548	0.991456235709744
Asf1a	356.61089300003	0.0105344735627239	0.192047691235128	0.0548534246622432	0.956255237710147	0.991521585464356
B3galt4	95.6644456761215	-0.016991418711525	0.305341741766237	-0.0556472187956971	0.95562284759658	0.991521585464356
Ccl9	115.037102249851	0.0645071550766427	1.16705958602226	0.0552732318462891	0.955920787448048	0.991521585464356
Csrp2	3790.13985859418	0.00996863051479079	0.183292579147989	0.0543864381260203	0.956627283670783	0.991521585464356
Ebf4	27.3101039709	-0.0277748865514147	0.502494115713164	-0.0552740533329336	0.955920132997033	0.991521585464356
Fam136a	469.474503039127	-0.0127738551586622	0.232266390663734	-0.0549965714891384	0.956141195216093	0.991521585464356
Fyco1	829.381728367498	-0.00853996962933604	0.153252063092507	-0.0557249896478138	0.955560891569948	0.991521585464356
Ndufa8	1469.69557737584	-0.00685033934641526	0.124542387664879	-0.0550040791320644	0.956135214037199	0.991521585464356
Rhbg	1937.23727725383	0.00899418670143478	0.164006047845711	0.0548405794760451	0.956265471282031	0.991521585464356
Rpl41	19251.6301309834	-0.0105537792422405	0.193324787025438	-0.0545909265160696	0.95646436757168	0.991521585464356
Rufy1	677.361981707809	-0.00514012205301291	0.0943174388495052	-0.0544981088938875	0.956538315236494	0.991521585464356
Srpk2	505.485838454362	0.00607546303480426	0.111090804089488	0.0546891624792836	0.956386103530586	0.991521585464356
Syngap1	120.987340145754	0.0132179103794447	0.242726472805411	0.0544559900149054	0.95657187140964	0.991521585464356
Wdr67	365.30401898392	0.00684848943455071	0.12444678758069	0.0550314682097376	0.95611339376449	0.991521585464356
Arl6ip6	398.597914156795	0.0107676105532675	0.19838660905393	0.0542758939457473	0.956715355081566	0.991522451523396
LOC314140	273.017812983252	0.00790513570240392	0.146697400692271	0.053887360410609	0.957024906965349	0.991752834843924
Lin37	230.71486818273	-0.0103510477439549	0.194063287492765	-0.0533385158918368	0.957462192620477	0.991780012300312
Mks1	232.204866680523	-0.0111744023018714	0.209624148266378	-0.0533068465359805	0.957487425213033	0.991780012300312
Pacsin3	516.692696875494	-0.00646016304773387	0.120836332683893	-0.0534620912787349	0.957363734208993	0.991780012300312
Psmg2	443.292319973408	0.0110460805371332	0.207206774201858	0.0533094566028631	0.95748534563789	0.991780012300312
Ptprs	133.869065063281	-0.0163334193837171	0.305096817787015	-0.053535200734605	0.957305484720672	0.991780012300312
Ap2b1	2462.53981943904	0.00623990694177826	0.119807553642083	0.0520827506454188	0.95846275730111	0.992428505009056
Ezh2	157.636682966672	0.00976860217939098	0.186820826339227	0.0522886145554955	0.958298725176134	0.992428505009056
Grn	1372.83618792796	-0.00828131076675262	0.158698694530322	-0.052182601698531	0.95838319587763	0.992428505009056
Spata5	138.656171193147	-0.0135195166058596	0.258803142609602	-0.0522386106657657	0.958338568055432	0.992428505009056
Acadvl	4768.84192128527	-0.00610499259953796	0.121418574431609	-0.0502805491508771	0.95989882369067	0.992545523484454
Adprm	198.354202202248	-0.00886908714807204	0.171299759400996	-0.0517752457976919	0.958707780080931	0.992545523484454
Arhgap28	54.4547851361537	0.0259048046286095	0.524690088981433	0.0493716294106067	0.960623137030935	0.992545523484454
Arl5b	57.1992763718867	0.0170559090947013	0.3374137916501	0.0505489387712651	0.959684951723157	0.992545523484454
Atp6v1c2	543.417357320517	0.00778190214599692	0.156722343601989	0.0496540695292294	0.960398058483375	0.992545523484454
Ccrn4l	1437.13083820113	-0.029011901317996	0.572345230424003	-0.0506895135589816	0.959572932875748	0.992545523484454
LOC100363289	39.712240494025	-0.0169932935954831	0.33270305287316	-0.0510764582673117	0.959264595082818	0.992545523484454
Pcdhga10	77.5217285667814	-0.0188877644928813	0.373049012568674	-0.0506307853834736	0.959619731089062	0.992545523484454
Pdcl3	940.046403963284	0.00625353262752251	0.123360051309407	0.050693336790511	0.959569886295132	0.992545523484454
Pinx1	93.6837197755388	-0.0120381013960531	0.23349322310553	-0.0515565344293197	0.958882053749442	0.992545523484454
Plin2	1147.29322326458	-0.00767451216063103	0.150519223242842	-0.0509869237648752	0.959335940319611	0.992545523484454
Pogk	486.973819653778	-0.00637159643727973	0.129500708318327	-0.0492012477770984	0.960758916890371	0.992545523484454
Ppp1r11	1157.43354353347	-0.00439087859812466	0.0890769212090324	-0.0492931113752888	0.960685709172328	0.992545523484454
Prmt6	73.8937531346275	0.0135712064281204	0.271873889375528	0.0499172850298066	0.960188303012439	0.992545523484454
Psma1	2236.41914860661	0.0078148014588567	0.158720694166539	0.0492361849845299	0.960731074775908	0.992545523484454
Ptprf	3061.32644520455	-0.00712750974502228	0.142177575605818	-0.0501310401070773	0.960017964399313	0.992545523484454
Rad51ap1	21.6538096545663	-0.0225261790845722	0.454426809020832	-0.0495705328941972	0.960464629096472	0.992545523484454
RGD1311164	197.912269832485	0.00968637641388266	0.194620226081543	0.0497706564672481	0.960305150439258	0.992545523484454
RGD1565096	19.0369712057379	-0.0236193322229015	0.476030882243089	-0.0496172267471507	0.960427418580885	0.992545523484454
Robo3	240.444956021985	-0.0622174834698155	1.23941198613559	-0.0501991945904977	0.959963653370176	0.992545523484454
Rxrb	831.601241981359	-0.00682717266506368	0.134206932299323	-0.0508704919194262	0.959428719091679	0.992545523484454
Sirt4	124.777293029666	0.0111833806298824	0.220231451931647	0.0507801248722337	0.959500728495547	0.992545523484454
Tubgcp2	281.908505595278	0.0103240244702747	0.205544876608287	0.0502275933150576	0.959941023014214	0.992545523484454
Usp32	883.287482385563	-0.00620782076984718	0.123579652776338	-0.0502333566277491	0.959936430353527	0.992545523484454
Zfp354a	2517.6532273153	0.0226502707313541	0.455784145356472	0.0496951703171666	0.960365305234616	0.992545523484454
Arpp19	779.845427700857	0.00719606985916487	0.147384236509682	0.0488252341605893	0.961058572200975	0.992764857914777
Eci2	3079.1090741473	0.0109894927761821	0.227446463763422	0.0483168328684712	0.961463739532004	0.993093135543508
Calm2	1975.35318841359	0.00554202247653256	0.116505426322844	0.0475687927288062	0.962059903656981	0.993257595146266
Gltpd2	21.2421907469225	-0.0213513879299761	0.447138903670161	-0.0477511300285476	0.961914584678607	0.993257595146266
Rbm12	176.025463047671	0.00896393505688887	0.18769797009692	0.0477572296187341	0.961909723455848	0.993257595146266
Set	1971.89327425632	0.00640282057929723	0.134174897618158	0.0477199587475648	0.961939427441932	0.993257595146266
Thap11	352.620759790203	-0.0068734862712733	0.144310644197481	-0.0476297941118419	0.962011286702025	0.993257595146266
Mettl9	2058.52583429933	0.00617477832266236	0.13098795894935	0.047140045330808	0.9624016112393	0.993520137451942
Tspan31	1124.79959787869	0.00446608532253762	0.095411926387438	0.0468084598187677	0.962665886465562	0.993702703257432
Mrp63	776.227488824887	0.0064538760528552	0.138292502524718	0.0466683004142009	0.962777595411003	0.993727765114554
Fbxo42	143.858262124486	0.0110971956473924	0.242114742977099	0.0458344482080632	0.963442201953027	0.993872215229452
Ift20	916.547685771662	-0.00645098577938267	0.140341632921476	-0.045966301268506	0.963337109197254	0.993872215229452
LOC683077	2347.94882917481	0.00422391627404858	0.0921346033825619	0.0458450584142638	0.963433745123078	0.993872215229452
RGD1561676	21.1008245129292	0.0200216212461085	0.435810890874673	0.0459410759697286	0.963357214833525	0.993872215229452
Slc35e3	567.988431439516	0.00762773668232646	0.165756170637535	0.0460178143171894	0.963296051178391	0.993872215229452
Ttc39a	40.4673815468473	0.0268101747270152	0.581697036840858	0.0460895844899241	0.963238847560642	0.993872215229452
Gcfc2	102.348650931677	0.0100990530361778	0.221965602364878	0.0454982795918822	0.963710146167051	0.994058401064952
Cav2	732.374688715336	-0.00731127208998814	0.161680396621135	-0.0452205229748453	0.963931536013478	0.994196537053465
Otud3	52.6723842399789	0.0132412931995687	0.29417322710769	0.0450118908840109	0.964097831006777	0.99427782843871
Ccdc19	9.69804210673148	-0.0329464717037519	0.748665431451253	-0.0440069359685631	0.964898875121933	0.994309556389085

Cdc42ep5	65.9131258632182	0.0186662024894658	0.418668222076158	0.0445847129187424	0.964438327866407	0.994309556389085
Cdkn1c	94.9888021596942	-0.0221907165825993	0.500568803092853	-0.0443310019431694	0.964640559981397	0.994309556389085
Hdac4	84.399775967677	-0.0112642028977766	0.254433401668984	-0.0442717144207005	0.964687818182738	0.994309556389085
Myh9	2071.6673242988	0.0111048229393596	0.251503541158843	0.0441537438725209	0.964781853108665	0.994309556389085
Ociad1	3341.01028750889	0.00525371838407044	0.119441927094821	0.0439855460461525	0.964915925301039	0.994309556389085
Pcnx	1147.46920389837	-0.0067349300613802	0.150869918566788	-0.0446406422523436	0.964393747101067	0.994309556389085
Vps33a	459.709847509521	-0.00729122766999826	0.164931319574177	-0.0442076598236337	0.964738876368061	0.994309556389085
Vps39	768.702025524313	-0.00540180365928132	0.122091901241736	-0.0442437508495016	0.964710108043671	0.994309556389085
Pik3ap1	79.4290427344357	0.0167326772426113	0.387275851880945	0.0432060949871854	0.965537246530354	0.994679266207197
Tgfbr2	346.080091098705	-0.0261553058542933	0.60285746810423	-0.0433855550243781	0.965394192280074	0.994679266207197
Zfp598	1163.01429130254	-0.00540316681194206	0.124946624031134	-0.0432437999332876	0.965507190427467	0.994679266207197
Ccdc132	649.910694425865	-0.00563484276975645	0.13310651256627	-0.0423333363718851	0.966232970515605	0.994795155705679
Elk1	365.218547529696	0.00567378042716848	0.134703898424865	0.0421203877060262	0.966402727552974	0.994795155705679
Galk1	1076.03495301102	-0.00688298224049832	0.161254714396498	-0.0426839132502771	0.965953503248816	0.994795155705679
Ly6g6c	26.4151825574294	0.0196398032909116	0.462854746268333	0.0424318934811694	0.966154403915523	0.994795155705679
Nudt2	1079.62764345876	0.00640508539645502	0.152929861900053	0.0418825029780059	0.966592364758945	0.994795155705679
Ppic	22483.2339645667	0.0139096735686877	0.329805601207044	0.0421753709390629	0.966358896229472	0.994795155705679
Prph	29.5405821539736	0.0190327180283901	0.448145947454588	0.0424699099400395	0.966124098488629	0.994795155705679
Psmd8	3103.63485851886	0.0063492859586323	0.150413759502821	0.042212135243606	0.966329588658427	0.994795155705679
RGD1307752	1973.58365187035	0.00643201022700452	0.15366540808706	0.0418572423493024	0.966612502165539	0.994795155705679
Sp1	1041.3270071968	-0.00501970165688303	0.11964406772647	-0.0419552908244398	0.966534339463324	0.994795155705679
Tmem25	583.518814228122	0.00611338303253164	0.142588469902934	0.0428743154105888	0.96580172325124	0.994795155705679
Dnajc21	763.933496064091	0.0073608194103935	0.177138921308736	0.0415539360633471	0.966854295193808	0.994888661895875
Gpatch4	140.384992330547	0.0120754432336599	0.290808670691342	0.0415236698580988	0.966878423306514	0.994888661895875
Rab1a	4998.3983975552	-0.00527739015058435	0.127715565534738	-0.0413214327360038	0.967039646808352	0.994964481363603
Mak16	283.101219238623	-0.00652108989041888	0.160135896670439	-0.0407222242233378	0.967517343920969	0.995005621697949
Sec16a	2074.90970737604	0.00376231249587421	0.0921794914671644	0.0408150710748323	0.967443324390524	0.995005621697949
Stard4	282.056574720716	0.00947287402411248	0.232159952731642	0.0408032217126713	0.967452770944291	0.995005621697949
Tigd5	33.1186811019504	-0.0168881103649981	0.412463120436104	-0.0409445342583406	0.967340113991779	0.995005621697949
Zfhx2	71.7794275560186	-0.0118306097096632	0.288661499175285	-0.0409843700786688	0.967308356263061	0.995005621697949
Acd	572.235961913052	0.00478555661583989	0.120026549334083	0.0398708172682672	0.968196117041965	0.995277390636655
Fam83f	129.108225639861	0.00982799625533566	0.246446781663695	0.0398787770284097	0.968189771119252	0.995277390636655
Fndc3b	113.669615041137	-0.0154522966542456	0.385142781731909	-0.0401209561419268	0.967996694666032	0.995277390636655
Med10	438.33320482237	0.00542227214178958	0.136095851276324	0.0398415682104841	0.968219435885051	0.995277390636655
Pdik1l	304.299496445907	-0.00999779642745264	0.250701127875783	-0.0398793436318577	0.96818931939445	0.995277390636655
Nudt22	334.553435322399	0.00616429446319869	0.155238372077991	0.0397085745018106	0.968325465606132	0.995296368428224
Fbxl4	283.652859495144	-0.00646669834908166	0.16368603471372	-0.0395067200472639	0.968486395878204	0.995319188277318
Zfyve19	256.221720666475	-0.00699710026569207	0.177316626043007	-0.0394610501104107	0.968522806822729	0.995319188277318
Mrps10	201.212784978703	0.0130255674480706	0.334705612162096	0.0389164895202367	0.968956969791666	0.99540536141107
Ndufs4	3071.6044566689	0.0053509589994043	0.137133476868061	0.0390200782596109	0.968874380666609	0.99540536141107
Slc28a1	310.022331764436	-0.00558352462158101	0.14286863050973	-0.0390815296658195	0.968825386909499	0.99540536141107
Vom2r31	34.1721676547381	-0.0146708447954432	0.375973366516357	-0.0390209682440497	0.968873671102164	0.99540536141107
Ccdc58	574.24576522598	-0.00713783927357129	0.186255302960184	-0.0383228781147625	0.969430250084591	0.995621597764658
Cd22	24.7552018097762	0.0162495084375736	0.421703099730407	0.0385330542933211	0.969262677530583	0.995621597764658
Klf2	177.576028453334	-0.0108592156377091	0.282693088786873	-0.038413445777219	0.969358040714879	0.995621597764658
Cdk16	1054.63089773694	-0.0040648087868156	0.107888271171095	-0.0376760953039039	0.969945935645235	0.995682890582436
Ckap4	481.627158402249	0.00772131639469636	0.208301717142404	0.0370679440410841	0.970430831403583	0.995682890582436
Gdap2	290.270238330745	0.00557528239300613	0.149839935969301	0.0372082539740832	0.970318957451441	0.995682890582436
Pmel	30.9124395479865	0.0145499689718362	0.384662075164954	0.0378253275049191	0.969826950390303	0.995682890582436
Psma8	59.5359609497981	-0.0108032824248448	0.288535794256833	-0.0374417408164911	0.970132791629295	0.995682890582436
RGD1559442	657.17453441624	0.00613434175135747	0.165912334795311	0.0369733191754901	0.970506221532972	0.995682890582436
Rims2	19.2793565404339	0.0184189909284051	0.494296665603614	0.0372630288855229	0.970275283682474	0.995682890582436
Rnf20	645.666152560814	-0.00546396056727042	0.143464310896478	-0.0380858523846613	0.969619231288392	0.995682890582436
Sema6d	1035.56143878058	-0.0237586278123854	0.639428172120339	-0.0371560541876063	0.97036057807468	0.995682890582436
Sparc	4771.37980408819	0.0210129296275125	0.55922182061255	0.0375753034895808	0.970026298972657	0.995682890582436
Stat3	2585.73587642405	-0.00550635707563089	0.149104235959091	-0.0369295817802363	0.970541152979307	0.995682890582436
Ttll13	82.5702869685814	-0.00934597208454677	0.252677938776246	-0.036987685311233	0.970494824720635	0.995682890582436
Ccl7	12.8148054651678	0.0336104913333622	0.919246906673871	0.0365630725426922	0.970833387671917	0.995892805440343
Psmd7	1674.85930254991	0.00422083183006457	0.116564223776916	0.0362103542004665	0.971114629947005	0.996091407271696
Cmklr1	61.6459638727063	0.0165501332796015	0.460450882383291	0.0359433197172695	0.971327554028212	0.996219904266799
Btc	72.9660358676809	0.0104249933821857	0.296865616615151	0.0351168771279443	0.971986543710284	0.99638564176914
Cnn3	5506.30621222502	0.00495883244231649	0.140248594641809	0.0353574483579049	0.971794714770497	0.99638564176914
Psenen	1616.35198298421	-0.00444297805794849	0.125411819192614	-0.0354271079596154	0.971739169249219	0.99638564176914
RGD1306941	255.263922883422	0.00539618028898357	0.15382048823156	0.0350810243227172	0.972015132494829	0.99638564176914
Slc16a12	838.392050399677	0.0100065876973137	0.281159780448658	0.0355903951886211	0.971608967000392	0.99638564176914
Slc23a3	4216.90583764148	-0.00561330719062778	0.159214508539482	-0.0352562542328598	0.971875405691212	0.99638564176914
Acsl5	984.820321394262	0.00686082753169362	0.196488558870342	0.0349171858714732	0.97214577662545	0.996429695835951
Fam50a	560.487868562452	-0.00427810151275912	0.123234313175947	-0.0347151812064802	0.972306855371852	0.996504934008699
Krt80	98.9315520274398	0.013116065430411	0.380195757759941	0.0344981898474853	0.972479885783081	0.996592406618925
Aifm1	3925.28866958218	0.00283094694596583	0.0985233565806215	0.0287337647053191	0.977076927137794	0.996751364279479
Alg2	660.764217291615	0.0032496592663022	0.133107963898287	0.024413710277962	0.980522612365306	0.996751364279479
Ankrd54	248.230041339988	0.00520765518474351	0.206872735697415	0.0251732311035929	0.979916788672919	0.996751364279479
Apopt1	1190.91351185724	0.0054704723553053	0.19834186132988	0.0275810276188084	0.977996313675921	0.996751364279479
Arhgef28	1417.65738613326	0.00355659040204915	0.115993752665476	0.030661913424823	0.975539165554584	0.996751364279479
Arrdc1	806.76990984226	0.00341593009532826	0.11969961822072	0.0285375187164713	0.977233444596851	0.996751364279479
Atp5o	10163.192056208	0.00476320210461539	0.174104292647749	0.0273583266223791	0.978173936334103	0.996751364279479
Atp6v0a2	164.911122404338	0.00655423664776343	0.226469621460413	0.02894090874307	0.976917118815748	0.996751364279479
Bdh2	2644.99502680537	0.00506095419111072	0.163845260444898	0.0308886212354781	0.97535836453318	0.996751364279479
Birc5	86.3194588092101	-0.00735819530983119	0.249798117368018	-0.0294565683174891	0.976500457358451	0.996751364279479
Btbd6	1003.32097706911	0.00270897170773498	0.0918440663895022	0.0294953371973589	0.976469537702673	0.996751364279479
Ccndbp1	661.065561916587	0.00426957432419946	0.135939384338967	0.0314079274741542	0.974944219061475	0.996751364279479
Cd82	4737.88951922745	-0.00261405396833801	0.083805956929395	-0.0311917441684999	0.975116623911569	0.996751364279479
Cd93	195.526574481107	-0.0115987839914427	0.45677137140742	-0.0253929749487235	0.979741514481589	0.996751364279479
Cenpl	52.2261602595532	0.00894809972757011	0.317772482012492	0.0281588250527569	0.977535477038732	0.996751364279479
Clcf1	148.318494086437	0.0103010414516875	0.318348203008525	0.0323577810532565	0.974186730681682	0.996751364279479
Copz2	141.782292177021	0.00868871501630772	0.346499126883492	0.0250757197989397	0.97999456688517	0.996751364279479
Csnk1g2	1512.36741458431	0.00514291529332093	0.155776354254298	0.0330147365300729	0.973662835996094	0.996751364279479
Ctxn1	20.122588422847	-0.0123474141254789	0.452530045430915	-0.0272852913307032	0.978232188319646	0.996751364279479
Cxcl14	39.6307007137446	0.0179976796210639	0.595849879482416	0.0302050570803129	0.975903515406257	0.996751364279479
Dcaf4	262.97699074853	-0.00502402639172479	0.176651018217357	-0.0284404043770814	0.977310899190589	0.996751364279479
Dom3z	277.577045932421	0.00392123027053471	0.173058444434458	0.0226584162555546	0.981922746332552	0.996751364279479
Eif2ak2	612.555240223917	-0.00371303263224726	0.151749390197855	-0.0244681881581606	0.980479158286599	0.996751364279479
Eif2ak3	567.441465563899	0.00477826165602057	0.184389843760717	0.0259139091316836	0.979326005889917	0.996751364279479
Fiz1	305.384674442872	0.00434544716179253	0.133737293144493	0.0324924115003405	0.974079367581535	0.996751364279479
Fkbpl	327.572558358709	0.00431208419501247	0.18027487793977	0.0239194958515137	0.980916823290616	0.996751364279479
Flrt2	27.4449769863851	-0.0117545168799703	0.491387319257564	-0.0239210830628071	0.980915557241487	0.996751364279479
Gnptg	627.678176754908	0.00284038492072153	0.111333364935431	0.0255124321659445	0.979646232281164	0.996751364279479
Gramd1c	21.6366175774935	-0.0120046405693049	0.506565914657636	-0.0236980819710665	0.981093435942732	0.996751364279479
Gtf2b	639.464811411315	-0.00450351957696357	0.169712774357549	-0.0265361260754338	0.978829719295315	0.996751364279479
Hes1	656.806715317853	0.0043615061583247	0.162198006107662	0.026890010937802	0.978547460754928	0.996751364279479
Hey1	22.7337472418994	0.0156477512538324	0.505417281003549	0.03096006377693	0.975301388882231	0.996751364279479
Inppl1	1028.71125953842	-0.00221150050071107	0.092986305608657	-0.0237830773707519	0.981025638534119	0.996751364279479
Itpr3	722.791221462413	-0.00592614543316261	0.184696813544705	-0.0320858022368004	0.974403625755311	0.996751364279479
Kif26a	65.2390066000517	0.0116717982399944	0.496939380647907	0.0234873682676884	0.981261514368959	0.996751364279479
Limd1	355.416457388574	0.0058035631044044	0.200407803568526	0.0289587680772119	0.976897475098801	0.996751364279479

LOC500475	35.2559419993652	0.0105604847232312	0.342115608915565	0.0308681756927305	0.975374669940819	0.996751364279479
LOC691496	34.5645914496766	-0.0114361426365877	0.442384135829237	-0.0258511590049471	0.979376056479828	0.996751364279479
Lrp6	241.285914328048	0.00626174042955285	0.274793493821014	0.0227870767334522	0.981820116622194	0.996751364279479
Lrrfip2	542.254012601912	0.0036283359648631	0.158524438640932	0.0228881804974028	0.981739468523977	0.996751364279479
Mdfic	358.234698385006	-0.00492287455183306	0.163301707684834	-0.0301458853163619	0.975950706153269	0.996751364279479
Mea1	2298.28311128761	-0.00398974048172312	0.121790160584695	-0.0327591363913885	0.973866665144358	0.996751364279479
Meaf6	347.277792904957	-0.0047501019682901	0.170973373826737	-0.0277827000893357	0.977835463965835	0.996751364279479
Mfsd3	420.628043023404	0.00447788635787847	0.189517308558559	0.0236278490441671	0.98114945802417	0.996751364279479
Morn5	41.3422882262369	-0.0116015770241875	0.367841362520616	-0.0315396206252833	0.974839195160642	0.996751364279479
Mrpl30	388.199070402416	-0.00556944905986177	0.222991339890406	-0.0249760778270537	0.980074044783886	0.996751364279479
Nadsyn1	1018.67554848042	0.00419085977942918	0.129225584842908	0.0324305731293364	0.974128681473951	0.996751364279479
Nhp2	287.460211843689	0.00522107070423562	0.22838530878505	0.0228607992870047	0.981761309854107	0.996751364279479
Nmt2	79.8768495819273	0.00734022095003237	0.298422299635848	0.0245967575445579	0.980376605623176	0.996751364279479
Nup107	186.325405309518	-0.00465431534629789	0.195797015489753	-0.0237711250840872	0.981035172383751	0.996751364279479
Panx2	1390.26934412998	-0.00677396927014668	0.236898514115818	-0.028594393238089	0.977188083805448	0.996751364279479
Pcna	1044.04554978926	0.00457132172739559	0.151823845105895	0.0301093792230539	0.975979820585285	0.996751364279479
Pdap1	1974.77657400852	0.00289189179977872	0.12208916493601	0.0236867194668293	0.981102499365293	0.996751364279479
Plcd3	149.326770616873	0.00582294160187088	0.239742175819172	0.0242883488563268	0.980622606656749	0.996751364279479
Pls1	1989.75485063401	-0.00295850304521919	0.127653225911599	-0.0231760930763157	0.981509808446235	0.996751364279479
Ppp1cc	3084.398014514	-0.00310460847006832	0.133223459344817	-0.0233037671093106	0.981407966812321	0.996751364279479
Psmb6	2159.99169053026	0.00470434919310571	0.158243031145964	0.0297286342345552	0.976283475193209	0.996751364279479
Qser1	141.867224269913	-0.00604568837195105	0.251113371982873	-0.0240755333904058	0.980792359190952	0.996751364279479
Rab10	1772.36850198745	0.00336210820293051	0.11991096704928	0.0280383711820852	0.977631547389615	0.996751364279479
Rbbp4	1552.66891643474	-0.00436815941934125	0.138063794692875	-0.0316387031738357	0.974760178159328	0.996751364279479
Rbck1	1176.28214329337	-0.00256339421021158	0.0974092299137769	-0.0263157219544863	0.979005514947842	0.996751364279479
Rbm4	627.661469485069	-0.0033160671216904	0.128992272141441	-0.0257074867093922	0.979490652301168	0.996751364279479
Rfc5	337.773054054051	0.00362554019930216	0.144450493684722	0.0250988425641193	0.979976123392649	0.996751364279479
Rfx3	23.9428473510461	0.0093089605757635	0.413026120651563	0.0225384306471424	0.982018456554778	0.996751364279479
RGD1310429	100.606519116018	-0.00851718594811208	0.310929197041294	-0.0273926862744284	0.978146531568961	0.996751364279479
RGD1563945	47.9567238006684	0.0127128237407795	0.42022879112889	0.0302521483752413	0.975865959151869	0.996751364279479
RGD1565367	13.4200648634917	-0.01841188681883	0.584919494781762	-0.0314776426210578	0.974888621911937	0.996751364279479
Rhog	718.096004720081	0.00254024726176381	0.109316976378824	0.023237445325609	0.981460869613641	0.996751364279479
Rock1	476.994733923818	-0.00778818129564324	0.233767202448636	-0.033315970820819	0.973422617956364	0.996751364279479
Scfd1	855.345658934835	-0.002943214756653	0.122748203309064	-0.0239776605873601	0.980870427852506	0.996751364279479
Sec11c	730.593773029615	0.00431126216288692	0.185484125229986	0.0232432945813627	0.981456203842979	0.996751364279479
Selk	1386.10199548592	-0.00418339927383753	0.158944615917302	-0.0263198551878858	0.979002218246614	0.996751364279479
Sgsm3	373.255934996966	0.00409594140545525	0.123848773456644	0.0330721192559014	0.97361707619376	0.996751364279479
Sharpin	876.343880084729	-0.00355191666944177	0.105152153566536	-0.0337788295243449	0.973053517902507	0.996751364279479
Shbg	58.3546337040986	0.0125701995632432	0.5123367150924	0.0245350356376005	0.980425837722178	0.996751364279479
Slc22a13	1109.10909653936	-0.0163495172552393	0.518901862765878	-0.0315079178326559	0.974864477764023	0.996751364279479
Slc25a33	655.183084585997	0.00720357117071403	0.275653971306772	0.0261326587698504	0.979151528020487	0.996751364279479
Slc27a3	26.0175957345566	-0.0149990440578438	0.593733221849288	-0.025262261746322	0.979845775081266	0.996751364279479
Snx11	305.61012192298	-0.00419390835459105	0.145680993938429	-0.0287883013508514	0.977033431183914	0.996751364279479
Soat1	173.472691669505	0.00971827094917844	0.294710205425456	0.0329756851655297	0.973693977520686	0.996751364279479
Sod1	24569.1367512783	-0.00522001776376461	0.188335229482427	-0.0277166294278027	0.977888160433382	0.996751364279479
Sox15	13.0004787512528	-0.0186432827157743	0.628165986358794	-0.0296789114989198	0.976323130698124	0.996751364279479
Spryd3	678.274852754617	-0.00321667330894025	0.0980652565762011	-0.0328013551511055	0.973832997541335	0.996751364279479
Srsf10	292.034842590141	0.00428719428383169	0.18816734960994	0.0227839436157165	0.981822615839607	0.996751364279479
Stat5b	356.940201152021	0.00478836735080622	0.190692827169239	0.0251103694978342	0.979966929127964	0.996751364279479
Stk16	1439.69866642612	-0.00287910437035936	0.0967434163657829	-0.0297602098263058	0.976258292658398	0.996751364279479
Stx2	92.0024492776064	0.007815576532909	0.295735305932635	0.0264276073100629	0.978916274387319	0.996751364279479
Tbc1d10b	1639.64367753753	-0.00394588916407571	0.121168536980373	-0.0325652951039169	0.974021245637833	0.996751364279479
Tceanc2	247.941698936649	-0.00377202119982926	0.144530966815126	-0.0260983599774446	0.979178885166751	0.996751364279479
Tcof1	277.082653171236	0.00425950158432473	0.155476181193386	0.0273964896206618	0.978143498076198	0.996751364279479
Tmc5	55.9667689379674	-0.0111183596963762	0.423035154804124	-0.0262823540079649	0.979032129511763	0.996751364279479
Tmem170a	146.459691303745	-0.0067511012716887	0.270316301231514	-0.0249748211296612	0.980075047170653	0.996751364279479
Tmem38b	1187.13252828001	-0.0031476838441764	0.139165616564989	-0.0226182581723156	0.981954779637663	0.996751364279479
Trdmt1	84.1615353846647	0.00660567037410666	0.233690033072507	0.0282668040534579	0.977449356542405	0.996751364279479
Trip10	703.843432695305	0.0041463782167944	0.146117829376827	0.0283769491682031	0.977361508695652	0.996751364279479
Tspan3	1957.95978392401	0.00295159836331381	0.120548147131204	0.0244848090456448	0.980465900708984	0.996751364279479
Unc5b	54.1938879184725	-0.0148698906515034	0.474975952907024	-0.0313066178624292	0.975025012705402	0.996751364279479
Vkorc1	1271.44624013346	0.00377754364028164	0.112581959680173	0.0335537207827349	0.973233026933339	0.996751364279479
Vps37b	382.935055782833	-0.00423246168030253	0.149396713705659	-0.0283303532943925	0.977398671882664	0.996751364279479
Vps37d	581.033086518119	0.00681846445312675	0.211301149804644	0.0322689415529953	0.974257577350258	0.996751364279479
Vps52	1127.77839469727	0.00515784262018799	0.193708658083489	0.0266268047655616	0.978757393724702	0.996751364279479
Wdr89	299.073618282296	-0.0085632003381851	0.252577784182681	-0.0339032206094247	0.972954324991153	0.996751364279479
Ypel5	2321.79896673997	0.00321052882969794	0.129866982466071	0.0247216711186518	0.980276969308911	0.996751364279479
Zdhhc14	101.195301600769	0.00900401676822075	0.277572860772555	0.0324383902055853	0.974122447629353	0.996751364279479
Zfp2	62.8250984684449	0.00983693838485593	0.336253928960025	0.0292544935170866	0.976661620265742	0.996751364279479
Zfp251	67.7597226201734	0.0100391162930594	0.37433780461686	0.026818334053475	0.978604630016861	0.996751364279479
Zfp672	468.392441018483	0.00452408187003579	0.159819572465276	0.028307433190129	0.9774169521485	0.996751364279479
Ddx3x	6304.27107842581	-0.00306118293477009	0.139380633836921	-0.0219627565932277	0.982477664299709	0.996847594086481
Irx5	244.065558413693	-0.00561307218706271	0.252707569281249	-0.0222117295616725	0.982279061062479	0.996847594086481
Mical2	49.3181380453425	-0.00702028736757529	0.318026338072821	-0.0220745470646138	0.982388490027993	0.996847594086481
Pim3	8490.23894584824	0.0049319062233687	0.225511797698075	0.0218698368498297	0.982551785725044	0.996847594086481
Psmd14	979.157913669577	0.00349626341344523	0.159077539456489	0.0219783598953737	0.98246521767022	0.996847594086481
Rps6ka2	219.471108048567	-0.00428542279442965	0.198612176498537	-0.0215768381877695	0.982785509678931	0.996995725009883
Med6	396.361735266999	0.00404556534651049	0.190079336832905	0.0212835619795268	0.983019456510085	0.997055072523079
Rbm38	353.942359422451	-0.00448994429159686	0.210605189098412	-0.0213192481667619	0.982990989511438	0.997055072523079
Tk2	554.159005157752	-0.00363335114839817	0.172277399473361	-0.0210901206978109	0.983173765684736	0.99712260379876
Ccdc130	229.490925691445	-0.00360474985756219	0.17208186986211	-0.0209478770799661	0.983287234603234	0.997148707039647
Cd151	1465.72935589016	-0.00309947309339081	0.150010559244285	-0.0206616994763914	0.983515521882805	0.997202267771629
Hs2st1	442.829159736422	-0.00263076771244864	0.126771816277766	-0.0207519919623494	0.983443494345731	0.997202267771629
Arid4a	372.095398481103	-0.0027344407834302	0.134669236192571	-0.0203048659125094	0.983800174151383	0.997232134517116
Pcmt1	894.814500610339	0.0025811076514263	0.1271806544679	0.0202948134071582	0.983808193237763	0.997232134517116
Pdlim7	253.278079444474	-0.00382058254027416	0.186948799928224	-0.020436518136201	0.983695152667166	0.997232134517116
Plekha7	54.7621574427568	0.00661906669512146	0.328206215169821	0.0201674020453775	0.983909831993746	0.99724622350998
Alg6	248.162301099422	-0.00304127346462055	0.153273933308594	-0.0198420788125624	0.984169350443843	0.997420315394089
Adam10	718.843411466442	0.0040625147878647	0.244566738703648	0.0166110682482765	0.986746894591962	0.997605953298919
Bad	560.862350304219	-0.00283075406562508	0.167232209764993	-0.0169270864123787	0.986494784028424	0.997605953298919
Bend5	754.242502602565	-0.00298292504052244	0.184931390080477	-0.0161299011445507	0.987130758951606	0.997605953298919
Btbd3	72.7530592803265	-0.00493109798723795	0.288166813711919	-0.0171119565217096	0.986347300384552	0.997605953298919
Cage1	26.594206543784	-0.00740434858895318	0.450122697470571	-0.0164496228040962	0.986875691820873	0.997605953298919
Ccdc104	1653.21364436343	-0.0033833340690339	0.176571233359297	-0.0191612982741606	0.984712431433268	0.997605953298919
Coq2	1718.19176242544	-0.00284963108611628	0.167392151007996	-0.0170236840195701	0.986417721393193	0.997605953298919
Ctnnb1	2710.33598678746	0.00260175428777221	0.158137062139782	0.0164525270203417	0.986873374905113	0.997605953298919
Dpm2	489.070310340194	0.00208744222705402	0.111891811477886	0.0186558980454667	0.985115610386725	0.997605953298919
Enah	171.025600986095	0.00359283114848068	0.211001611297553	0.0170275057445609	0.986414672539746	0.997605953298919
Fbxo4	516.774863520497	-0.00311488354467363	0.187473563043114	-0.0166150549128748	0.986743714132754	0.997605953298919
Gpc3	680.725718496785	-0.00747781803935577	0.465266762907214	-0.0160721088105042	0.987176864586014	0.997605953298919
Herc4	1131.48864995957	0.00269575471287819	0.159732538261784	0.0168766786167271	0.986534997885885	0.997605953298919
Higd2a	1665.30618641705	-0.0030215868346053	0.164287881402576	-0.0183920250770116	0.98532611443468	0.997605953298919
Hltf	422.575827692439	-0.00253329712263458	0.155577221126989	-0.0162832135982606	0.987008449375445	0.997605953298919
Hnrpd	613.211161429763	0.00301208646841419	0.159834957928024	0.018844979267743	0.984964771917104	0.997605953298919
Maml3	28.6994817829351	-0.00764828467771995	0.44500143407864	-0.0171871011911579	0.986287352429172	0.997605953298919

MGC94199	210.748856061133	-0.00290162202497459	0.174490996966173	-0.0166290643954376	0.986732537727	0.997605953298919
Oxsm	365.087754665914	0.00205334237928649	0.127322401291947	0.0161271100643022	0.987132985621815	0.997605953298919
Pcif1	738.924898149953	-0.00273989964759878	0.146708732199567	-0.018675777552707	0.985099751597771	0.997605953298919
Rad18	27.4934914648211	0.00632805094556971	0.391308993788372	0.0161714937454058	0.987097577185474	0.997605953298919
Rasa2	96.6124856660199	-0.00493369526376285	0.269076523226656	-0.0183356585873788	0.98537108080384	0.997605953298919
Rb1	1213.23553755803	-0.00182118052403354	0.110321578391377	-0.0165079266503305	0.986829178397923	0.997605953298919
RGD1310262	301.312228810117	0.00503094094014529	0.289322193413644	0.0173887142247417	0.986126512539736	0.997605953298919
Slc34a2	1033.55598632752	0.00362627469141178	0.222981652694679	0.0162626594950263	0.987024847005817	0.997605953298919
Smad2	392.490431544198	0.00211483924175638	0.12379338914978	0.0170836201858695	0.986369906204957	0.997605953298919
Tonsl	19.3401158467199	0.00781896583498879	0.492635341361843	0.0158717111390629	0.987336738400452	0.997605953298919
Tp53rk	291.914179056479	0.00275165697494111	0.164502721512103	0.0167271212880126	0.986654310526913	0.997605953298919
Trak1	1887.07871542054	-0.00155800941192385	0.0976564938527215	-0.0159539765401933	0.987271108416913	0.997605953298919
Ttc12	261.32671183148	0.00290313280387317	0.179442244258193	0.0161786474298436	0.987091870117736	0.997605953298919
Ube2j2	967.200990192378	0.00218912587877772	0.123566522880644	0.0177161728576943	0.985865278598026	0.997605953298919
Vimp	1216.49000202981	0.0025249659178425	0.130391455934176	0.0193645043668901	0.984550326507562	0.997605953298919
Zbtb3	21.6932361101611	0.00716043733227846	0.446538571441642	0.0160354284942533	0.987206127472925	0.997605953298919
Zbtb9	199.202596093575	-0.00279508350414348	0.17348875873217	-0.0161110352311558	0.987145809816872	0.997605953298919
Ppp1r35	83.9622671680716	0.00403468394767028	0.257828996205585	0.0156486819056346	0.987514667883272	0.997697041347668
Ech1	4793.76318129293	0.00342896906030344	0.221835182568464	0.0154572823868693	0.98766736413464	0.997762622056201
Cttn	1605.36158866546	-0.0014146207325144	0.0993184789369765	-0.0142432782665959	0.988635892418429	0.997797300204334
Gtf3c5	289.794913241279	-0.00208493203662808	0.144517979625032	-0.0144268003333403	0.988489478039376	0.997797300204334
Mat2b	1572.8057062877	0.00162935173509832	0.112788788497163	0.0144460434127219	0.988474125883292	0.997797300204334
Nfatc3	458.76269700618	0.00260871618187828	0.179666009886857	0.0145198091921843	0.988415275479168	0.997797300204334
Orai2	603.758855919668	-0.0036748044655065	0.251164044398338	-0.0146310928951215	0.988326493361877	0.997797300204334
Prdm2	491.597874412461	-0.00203376509610477	0.141007994095336	-0.0144230482048396	0.988492471493324	0.997797300204334
Psmc6	1787.06442350151	0.00212407914789977	0.146479427999394	0.0145008700328114	0.988430385151272	0.997797300204334
Rhbdd3	447.736609474184	0.0020853054532395	0.14383214777071	0.0144981875440238	0.988432525242686	0.997797300204334
Rsrc1	204.92673397567	-0.00239656786360916	0.165967509430109	-0.0144399820894967	0.988478961615117	0.997797300204334
Slc5a10	3802.07611730117	0.0025858149812429	0.183471799088837	0.014093800758943	0.988755146243306	0.997797300204334
Tbl3	433.075370543904	0.00186974143588605	0.131906740824028	0.0141747224152889	0.988690586552195	0.997797300204334
Usp9x	1563.05152215324	-0.00178426889451663	0.11875576206141	-0.0150246932320973	0.988012480254269	0.997797300204334
Crb3	860.941049420143	-0.00232267359676586	0.166683909722891	-0.0139345999300548	0.988882157653615	0.997836878896572
Aptx	212.767408087504	0.0024882073970135	0.182035360452932	0.013668813525144	0.9890942043249	0.997856923663973
Atg5	849.611757093416	-0.00182716898168883	0.138093447637616	-0.013231395210609	0.989443182074507	0.997856923663973
Cask	151.355269930917	0.00306160830501973	0.236095809281467	0.0129676520491296	0.989653600616035	0.997856923663973
Khk	16942.8523656468	-0.00236972502021816	0.177625943606109	-0.013341097432665	0.989355660090508	0.997856923663973
Lmnb1	75.6959927307178	-0.00413245408585072	0.30641244605339	-0.0134865738617245	0.989239597136034	0.997856923663973
Mrpl14	290.87367563275	0.00246426539235537	0.190685125045089	0.0129232177484881	0.989689051087888	0.997856923663973
Psmd4	2097.06918432937	-0.0018037978948257	0.138308730369625	-0.0130418223781327	0.989594426259757	0.997856923663973
Rasgrp3	347.50554895073	-0.00345240865628065	0.26722835925892	-0.0129193198875108	0.989692160871368	0.997856923663973
Sv2a	182.839740306313	0.00362286158779555	0.272148008973275	0.0133121002849274	0.989378794412596	0.997856923663973
Zfp362	241.31464706471	0.00241695561212667	0.189220151780046	0.0127732463450098	0.989808701079031	0.99788590530994
Dpy30	306.491537878933	0.00244885340786289	0.193429273555475	0.0126602006141566	0.989898891229448	0.997888319087708
Ccdc12	738.605840632443	0.00168122356370181	0.135046541426357	0.0124492159957951	0.990067219332174	0.997969494051923
Fem1c	524.346329465048	0.00127739943014811	0.106087213406384	0.0120410310454176	0.990392879383626	0.998209228258782
Nup43	145.181658740777	0.00235562043397751	0.202161124715882	0.0116521929588991	0.990703105517881	0.998294939327029
Thap7	384.897154972564	-0.00178462197615947	0.153791347915635	-0.0116041767000993	0.990741414359395	0.998294939327029
Txnrd3	96.5819368117438	-0.0027790351918471	0.236501635008372	-0.0117505961079243	0.990624596539583	0.998294939327029
Camk2g	86.0787746791886	0.00331469086595113	0.311341518705149	0.0106464787598414	0.991505499442658	0.998296323975112
Galnt13	21.7366045668738	-0.0107045164567343	1.01932451276746	-0.0105015785676257	0.991621106605579	0.998296323975112
Klhl12	216.175040942194	-0.00201401958281309	0.185637807353621	-0.0108491885975392	0.991343769734561	0.998296323975112
Lnx2	283.933038757924	-0.00226583713009024	0.198937761554111	-0.0113896784219819	0.990912547912961	0.998296323975112
Map2k7	142.720261841693	-0.0021614416461648	0.200807819738338	-0.0107637324531547	0.991411949891629	0.998296323975112
Nqo1	292.275442548644	0.00399671996099513	0.365322611681066	0.0109402479704277	0.991271119178595	0.998296323975112
Olfml2a	250.584034970551	0.00235858984677665	0.214507394255008	0.0109953778282008	0.991227134561843	0.998296323975112
P2rx7	38.8311472143422	-0.00708925771575125	0.674741489932423	-0.0105066278293651	0.991617078099845	0.998296323975112
Psmd13	2300.98800887834	0.00139745120760704	0.13038052257929	0.0107182513151624	0.991448236496204	0.998296323975112
Zc3h14	634.28729792017	0.00122481045126604	0.115471316720823	0.0106070536480266	0.991536954354558	0.998296323975112
Abca4	1214.92261687688	0.00202598456865768	0.24989890363225	0.00810721671528061	0.993531447811587	0.998361832198658
Adamtsl5	9.56524825979627	0.0061712864888076	0.809091497158381	0.00762742719517117	0.993914252611458	0.998361832198658
Agxt2l2	172.751649022813	0.00173082404286097	0.21847231211245	0.00792239541077452	0.993678909140429	0.998361832198658
Apom	9680.4862414181	-0.00134884121655566	0.16047622146336	-0.00840524037926474	0.993293667436545	0.998361832198658
B3gnt1	456.464779289548	-0.00152557659106363	0.170833504602236	-0.00893019548252983	0.992874829603143	0.998361832198658
Camsap3	718.540770707269	-0.00101939790338555	0.107853746305931	-0.00945166893409521	0.99245877156524	0.998361832198658
Ccdc174	212.432856890852	0.00238112947174287	0.239887278226719	0.00992603479994653	0.992080300132956	0.998361832198658
Ccdc41	549.009486263427	-0.00120156419705427	0.12411106093047 1	-0.00968136270889998	0.992275510835054	0.998361832198658
Cdc14b	262.057224160907	0.00157187418930488	0.189599450236931	0.00829049972107303	0.993385214046305	0.998361832198658
Elac1	119.892147867247	-0.00203713335597009	0.204257309318966	-0.00997336821268378	0.992042535403062	0.998361832198658
Ext1	511.805293637814	-0.00097039364963179	0.122324537163062	-0.0079329435625677	0.993670493197435	0.998361832198658
Gpaa1	1081.4229456038	0.000698712828371151	0.0924515067667347	0.00755761428674257	0.993969953647722	0.998361832198658
mrpl11	992.207384807811	-0.00102579257963681	0.127883242702555	-0.00802132130808339	0.993599980202362	0.998361832198658
Mrpl23	607.688562378389	-0.00166362996404528	0.173949103331783	-0.00956388927669343	0.99236923673349	0.998361832198658
Pdcd5	342.574313913417	-0.0014994249463966 1	0.193111002922219	-0.00776457541883591	0.99380482740193	0.998361832198658
Pdgfc	96.3630575208681	-0.00275076253395071	0.327950397742015	-0.00838773958772456	0.9933076305557	0.998361832198658
Prps2	308.301306231231	-0.00188631039306919	0.234886210039056	-0.00803074132259846	0.993592464360297	0.998361832198658
Rfc3	149.930186730507	-0.00166267146096089	0.181098263903576	-0.00918104583181516	0.992674688189294	0.998361832198658
S100g	108.615426901298	0.00378575833332223	0.494424186257221	0.00765690360332152	0.993890734527258	0.998361832198658
Sat2	1276.40581993474	-0.00101723099177052	0.132816589509392	-0.00765891516660718	0.99388912957903	0.998361832198658
Ssrp1	1984.93217140456	-0.00102414426127588	0.113677466921995	-0.00900921078738183	0.992811787047465	0.998361832198658
Tars	1004.14960719997	-0.00106171783141059	0.123240297259836	-0.00861502166918744	0.993126292245438	0.998361832198658
Tpcn1	4518.09151422043	0.00147550852379381	0.166749595839559	0.00884864827626627	0.992939892289243	0.998361832198658
Tssc4	646.998787933329	0.00115685287790531	0.132993576585387	0.00869856204793898	0.993059639164587	0.998361832198658
Tymp	539.918338176642	-0.00115827403608405	0.127353787460725	-0.00909493199361074	0.992743394222614	0.998361832198658
Ube2e2	458.354229558754	-0.00133061554734861	0.145458183303599	-0.00914775310077508	0.992701250829906	0.998361832198658
Anapc1	807.343760808923	-0.00127371532680104	0.180475650731807	-0.00705754666425238	0.994368939225704	0.998589336575108
Fam213a	399.592106313228	0.00101015047802354	0.143212771049703	0.00705349439592196	0.994372172387566	0.998589336575108
Ap3s1	374.590332796452	0.00117821861877104	0.195475983111621	0.00602743416360385	0.995190832459102	0.999024967034108
Cela1	1707.46024040054	0.00144111567463108	0.232370966815226	0.00620178886537495	0.995051720135255	0.999024967034108
Fga	256.526146446577	0.00138205801207978	0.231928264654284	0.00595898914752756	0.995245442699912	0.999024967034108
Ndufa5	2289.19549815352	0.00109248693528897	0.179959606771808	0.00607073417688816	0.995156284679104	0.999024967034108
Pramef8	46.9850851624905	-0.00191037635005754	0.316744514730787	-0.00603128471437379	0.995187760219935	0.999024967034108
Lrp12	225.547578422326	-0.00117743071746	0.201942815715385	-0.00583051550157375	0.995347948057469	0.999039630662416
Bmpr1a	472.867054609133	0.00095415593836745	0.176938942426251	0.00539257172719422	0.995697371129146	0.999072242539945
Icam2	154.521305411589	0.00154274088164941	0.309177938433123	0.00498981553945226	0.996018719740982	0.999072242539945
Jrkl	185.575088848349	-0.00119402311184128	0.241498053911181	-0.0049442349224082	0.996055087362968	0.999072242539945
LOC685046	2102.38893696648	0.00106669553340614	0.19445893863528	0.00548545384898347	0.995623263014395	0.999072242539945
Pex11a	1343.02892841455	0.0023448369349206	0.474348234598363	0.00494328167344399	0.996055847936305	0.999072242539945
Rpl10	10576.105743663	-0.000837836798138976	0.163075274857977	-0.00513773040621057	0.995900702265689	0.999072242539945
Snx1	2503.98404868342	0.000612447422907085	0.124774122726024	0.00490844903996545	0.99608364001959	0.999072242539945
Tmem107	182.05774811582	0.00129928530412938	0.249694252462348	0.00520350505194468	0.995848222392912	0.999072242539945
Ergic2	743.870347480804	-0.000585558638568797	0.126871107357102	-0.00461538210524666	0.996317470950076	0.999130437164158
Plekhj1	646.243938453496	-0.000817544881057056	0.175637190060522	-0.00465473673756306	0.996286070833855	0.999130437164158
Abcb4	30.070073400088	-0.00188219011084498	0.425659751009427	-0.00442181838048224	0.99647191088069	0.999197153865895
Map3k14	264.881720783994	-0.000694519466799572	0.164184918779095	-0.00423010512758497	0.996624874493761	0.999262378572343
Arrdc3	436.69834900185	0.000607839864379485	0.257872902281718	0.0023571296518602	0.998119284384532	0.999281763246856

Cdk4	956.914937184779	0.000304713563445541	0.108106707144028	0.00281863698835613	0.997751056042353	0.999281763246856
Ftcd	770.642518651362	-0.000909449171609591	0.252630683359145	-0.00359991573278809	0.99712768902052	0.999281763246856
Fut11	64.2489101432876	-0.00117734598034455	0.3203208567777	-0.00367552082679904	0.997067365282438	0.999281763246856
Galnt4	59.049910770364	-0.000823293822378436	0.352944780415461	-0.00233264201105145	0.998138822641348	0.999281763246856
LOC361646	316.305985465106	-0.000418401244833295	0.163980558112072	-0.00255152958161869	0.997964176149367	0.999281763246856
Myct1	24.5036534610122	0.00212697592669279	0.705463219999901	0.00301500612135823	0.997594376809667	0.999281763246856
Pop5	517.076834737037	0.000399328382947269	0.14051082410119	0.00284197595097505	0.997732434319	0.999281763246856
Prickle1	159.288872101897	0.00111388427515838	0.288999433497444	0.00385427840351884	0.99692473838287	0.999281763246856
Rplp0	17846.6754203904	-0.000432019520195424	0.133574728336282	-0.00323429083911585	0.99741941377342	0.999281763246856
Snurf	129.908104336002	-0.000910609275197654	0.244694786931028	-0.00372140856214615	0.997030752417285	0.999281763246856
Sugp2	261.815137241329	-0.000468052054232067	0.196763418449587	-0.0023787554511917	0.998102029541506	0.999281763246856
Tmem177	140.39932390832	0.000671787339836721	0.266748678445027	0.0025184279965427	0.997990587308175	0.999281763246856
Tmem208	773.63478159593	0.000359483595250629	0.145555854414468	0.00246972955293851	0.998029442923609	0.999281763246856
Tmem8a	2458.56216121573	-0.000493884497597588	0.124675936306253	-0.00396134580761773	0.996839311606494	0.999281763246856
Trpv1	139.894227830048	0.00409487903797025	1.31058042164767	0.00312447749892537	0.997507031699241	0.999281763246856
Xpo5	446.990626057562	0.000391420357010442	0.144621662022193	0.00270651264504468	0.997840517983345	0.999281763246856
Bco2	37.0957060389079	-0.000775937211038648	0.35103297876099	-0.00221043963953873	0.998236325775257	0.999291358002605
Fdxacb1	133.789768138288	0.000469293627349633	0.249805853854567	0.00187863342715278	0.998501068274754	0.999468352444813
Apbb1	203.17768839159	-0.000409860900552205	0.24977303488591	-0.00164093334070037	0.998690725209721	0.999494064404055
Bbs10	150.358781267642	-0.000422683182314084	0.273530753729056	-0.0015452857733605	0.998767040830106	0.999494064404055
Fam168a	244.839150811134	0.000355161924384387	0.240333762537955	0.00147778622792666	0.998820897613734	0.999494064404055
Fam45a	610.107806608278	-0.00022605018251054	0.160822603727766	-0.00140558713309473	0.998878504096926	0.999494064404055
Cdc37l1	404.015007590061	0.000159213341981518	0.140291056867264	0.001134878769444	0.999094497945851	0.99953420153618
Yars2	270.014323216673	-0.000234875599809004	0.205658827663499	-0.00114206427449499	0.999088764746025	0.99953420153618
Got1	3146.14266811164	0.000243414178786855	0.264117340369517	0.000921613773810922	0.999264658702949	0.999616450520834
Hmga1	69.1199302001994	-0.000152292327511295	0.342386683321847	-0.000444796292991742	0.999645103916822	0.999909025003837
Pgam5	524.432900619504	3.37521533698981e-05	0.146710470236211	0.000230059608667027	0.9998164389918	0.999913898316074
Rcl1	314.430271112094	4.505593508891e-05	0.206515971595649	0.000218171673313132	0.99982592419164	0.999913898316074
Lzts2	716.889411453729	5.51772172339798e-06	0.107836467663715	5.11674931768431e-05	0.999959174247197	0.999959174247197
